# Engineered non-covalent π interactions as key elements for chiral recognition

**DOI:** 10.1038/s41467-022-31026-8

**Published:** 2022-06-07

**Authors:** Ming Yu Jin, Qianqian Zhen, Dengmengfei Xiao, Guanyu Tao, Xiangyou Xing, Peiyuan Yu, Chen Xu

**Affiliations:** Department of Chemistry and Shenzhen Grubbs Institute, Guangdong Provincial Key Laboratory of Catalysis, Southern University of Science and Technology, 518055 Shenzhen, China

**Keywords:** Stereochemistry, Asymmetric catalysis

## Abstract

Molecular recognition and self-assembly are often mediated by intermolecular forces involving aromatic π-systems. Despite the ubiquity of such interactions in biological systems and in the design of functional materials, the elusive nature of aromatic π interaction results in that they have been seldom used as a design element for promoting challenging chemical reactions. Described here is a well-engineered catalytic system into which non-covalent π interactions are directly incorporated. Enabled by a lone pair-π interaction and a π-π stacking interaction operating collectively, efficient chiral recognition is successfully achieved in the long-pursued dihydroxylation-based kinetic resolution. Density functional theory calculations shed light on the crucial role played by the lone pair-π interaction between the carbonyl oxygen of the cinchona alkaloid ligand and the electron-deficient phthalazine π moiety of the substrate in the stereoselectivity-determining transition states. This discovery serves as a proof-of-principle example showing how the weak non-covalent π interactions, if ingeniously designed, could be a powerful guide in attaining highly enantioselective catalysis.

## Abstract (web summary)

Non-covalent π interactions have been rarely used as a design element for promoting chemical reactions. Here the authors report a Sharpless asymmetric dihydroxylation (SAD)-based kinetic resolution in which a-priori-designed non-covalent forces play a central role in differentiating the enantiomeric substrates.

## Introduction

**Fig. 1:**
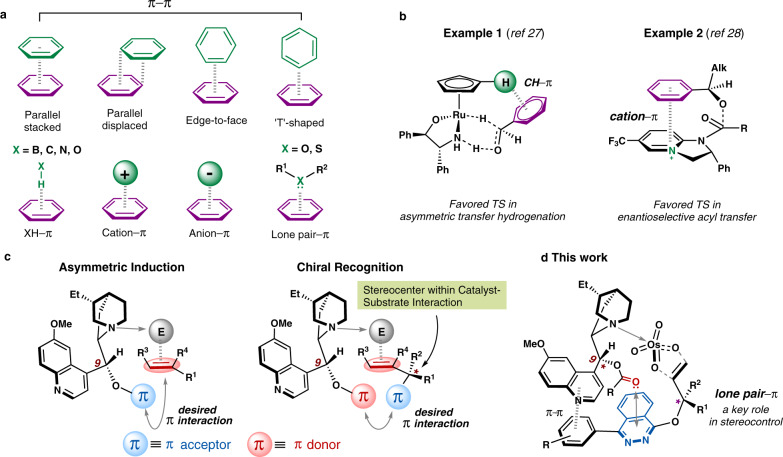
Orchestrated non-covalent π interactions as a crucial design element in asymmetric catalysis. **a** Non-covalent interactions involving π systems. **b** Representative examples using non-covalent π interactions to rationalize the observed enantioselectivities. TS, Transition State. **c** Designing cinchona alkaloids-based, *bi*-functional catalysis modes using non-covalent π interactions for both asymmetric induction (left) and chiral recognition (right). E: electrophiles. **d** Lone pair–π interaction as a key stereocontrol element in asymmetric dihydroxylation-based kinetic resolution.

Attractive non-covalent interactions (NCIs), such as hydrogen bond, ion pair, and van der Waals forces, have been integral to biocatalysis, and their application to chemical catalysis has been accelerated over the past two decades^1–6^. Prominent examples of the latter include thiourea-catalyzed asymmetric Strecker reaction^7^, chiral ion pair-catalyzed fluorination reaction^8^, and chiral phosphoric acid-catalyzed Ugi reaction^9^. Particularly, NCIs involving π systems have drawn increasing attention as they underpin many important structural phenomena in molecular biology and materials science^10–12^. These include π–π^13–15^, XH–π^16,17^, cation–π^18–21^, anion–π^22,23^ and lone-pair–π interactions^24–26^ (Fig. 1a). Recent advances in theoretical and computational chemistry have now reached the stage at which non-covalent π interactions can be modeled accurately, thus often attributed to when rationalizing the observed reactivity and selectivity in a variety of chemical transformations^4,27–29^ (Fig. 1b). Although in some early work the use of π-effects in chiral catalysts could be seen^30,31^, the direct incorporation of specific π interactions into the design of catalysts as a key stereocontrol element still remains a challenge^32,33^. This is ascribed to the potential competition and synergetic cooperation between the relatively weak and unpredictable π interactions and other types of intermolecular forces operating simultaneously, which makes the precise control of such interactions hardly possible and highly context-dependent. Moreover, the detailed understanding of the reactivity- and selectivity-determining transition states at the molecular level for individual catalytic process is still lacking. These obstacles, even though having been overcome gradually through the synergistic efforts from experimental, theoretical, and computational chemists, still hamper the exquisite design of catalytic systems with precise control of reactivity and selectivity.

We envisioned if the weak, yet important non-covalent interactions were purposely utilized in the design of catalysts, it might open up a new avenue in enabling challenging chemical transformations that were previously considered difficult or even hardly possible to occur. We initiated out studies aimed at identifying appropriate catalytic systems in which non-covalent π interactions are presumably essential for stereocontrol. The cinchona alkaloid and its derivatives, as privileged catalysts and ligands used in asymmetric catalysis^34,35^, contain versatile π-character groups with varying steric and electronic properties. In cinchona alkaloid-catalyzed asymmetric reactions, the corresponding stereochemical outcomes are mainly governed by the absolute configuration of C9^36,37^. An effective *bi*-functional catalysis mode may operate through the interaction between the cinchona alkaloid and a combination of electrophiles and nucleophiles connected by its quinuclidine *N*–C9–π scaffold (Fig. 1c). Taking the enantioselective functionalizations of alkenes as an example, the quinuclidine nitrogen coordinates to the electrophile, which subsequently reacts with the double bond of the substrate. An efficient asymmetric induction requires a π acceptor component (in blue) at C9 stereocenter in the cinchona alkaloid^14^. This would engage in an effectively operating π–π interaction between the π acceptor and alkene’s double bond that was a prototype of electron-rich π systems (in red). Such a “sandwich-like” mode would significantly facilitate enantio-face control of the alkene double bond (Fig. 1c, left). In comparison, to achieve efficient chiral recognition of an alkene substrate bearing an existing stereocenter at the allylic position, a π donor moiety (in red) at C9 in the cinchona alkaloid and a π acceptor moiety (in blue) adjacent to the stereocenter in the alkene substrate are needed^14^. This would enable a desirable non-covalent π interaction that incorporates the substrate’s stereocenter into the corresponding catalyst–substrate interaction framework, thus rendering a kinetic resolution process with potentially superior stereoselectivity (Fig. 1c, right).

We chose the Sharpless asymmetric dihydroxylation (SAD)-based kinetic resolution as a platform to investigate our proposed *bi*-functional catalysis strategy that utilizes non-covalent π interactions as key stereocontrol elements. The classic SAD reaction is widely applied to convert prochiral alkene substrates into chiral vicinal diols with excellent stereoselectivities. In stark contrast, the application of the powerful SAD reaction for the kinetic resolution of racemic olefin substrates such as allylic alcohol and its derivatives has been considered as an unsolved problem^36^. Despite of some sporadically reported cases^38–44^, SAD-based kinetic resolution has been proven to be generally ineffective. The reasons for such orthogonal stereochemical scenarios had not yet been well understood^36^. Thus, further investigation into such a catalytic system would not only help shed light on the intricate origin of enantioselectivity in this important catalytic reaction, but also stimulate understanding on the underlying principles of π interactions as well as developing catalysts with assembling properties based on these non-covalent forces. To the best of our knowledge, very few examples exist that use such weak yet essential non-covalent π interactions as the control elements in asymmetric catalytic reactions^45,46^.

In this work, we report the use of well-orchestrated non-covalent π interactions as crucial design elements in asymmetric catalysis. Relying on the favorable interaction between the lone-pair electrons on the C9 carbonyl of the cinchona alkaloid and the electron-deficient phthalazine π-system in the substrate, a highly efficient SAD-based kinetic resolution of racemic allylic substrates is realized (Fig. 1d). Density functional theory calculations indicate that the favored transition state is stabilized by this lone-pair–π interaction, which is absent in the disfavored transition state.

## Results and discussions

### Experimental studies on the development of effective non-covalent π interactions

**Fig. 2:**
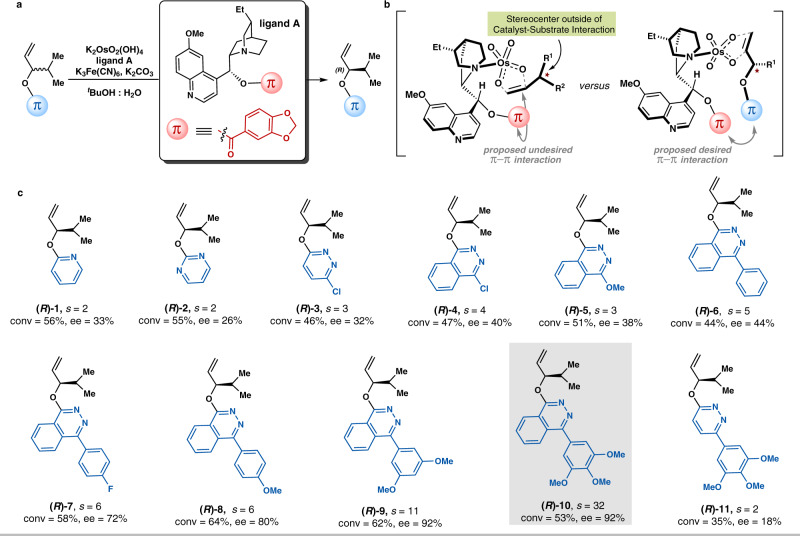
Development of effective non-covalent π interactions between the catalyst and the alkene substrate for AD-based kinetic resolution. **a** Using cinchona alkaloid ligand **A** with the piperonylate moiety as a large, delocalized π system in AD-based kinetic resolution. **b** Proposed possible π–π interactions in the racemic alkene substrates/Ligand **A**-OsO_4_ complexes. **c** Screening an appropriate π moiety in the alkene substrate that could establish desired π–π interactions with the piperonylate moiety in ligand **A**. General condition: K_2_OsO_2_(OH)_4_ (0.4 mol%), K_3_Fe(CN)_6_ (3.0 equiv), K_2_CO_3_ (3.0 equiv), ligand **A** (1.0 mol%) and alkene (0.1 mmol) in 1.0 mL ^*t*^BuOH-H_2_O (*v*/*v* = 1:1), 0 °C. *s*: selectivity factor; *s* = In[(1 − c)(1 − ee)]/In[(1 − c)(1+ee)]. Conversion (conv) was determined by ^1^H NMR analysis of the crude mixture.

Guided by the above analysis, our investigations started to focus on cinchona alkaloid ligands with a large, delocalized π-system, such as piperonylate in ligand **A**, to test the validity of our hypothesis (Fig. 2a). As the aromatic ring in piperonylate is flanked with both electron-donating and withdrawing substituents, its π-electron distributions are considerably polarizable thus conducive for potential π–π interactions^13,14,47,48^. Considering the potential undesired π–π interaction between the piperonylate moiety and the alkene’s double bond that would preclude the stereocenter outside of the catalyst–substrate interaction (Fig. 2b, left), an appropriate electron-poor π-character group that could compete in the π–π interaction with the piperonylate should be introduced in the racemic substrate (Fig. 2b, right). Thus under the catalysis of K_2_OsO_2_(OH)_4_ and cinchona alkaloid **A**, AD-based kinetic resolutions of racemic alkene substrates with a variety of electron-poor π systems were conducted (Fig. 2c). Racemic **1** bearing electron-deficient pyridine as the π acceptor was recovered in 33% ee at 56% conversion, and the corresponding selectivity factor was only obtained as 2. Then substrates including more electron-deficient aryl rings, such as pyrimidine and pyridazine, were explored. However, racemic **2** and **3** were only kinetically resolved with selectivity factors of 2 and 3, respectively. Subsequently, racemic **4** with larger aromatic π-system (i.e., 4-chorophthalazine) was examined, and its selectivity factor was slightly improved. Replacing the chloride with a methoxy group in the substrate **5** resulted in a less effective kinetic resolution. Considering that the extended arenes could probably engage in better π–π interactions^1,49^, we attached the phthalazine ring with a phenyl group. To our delight, the selectivity factor was increased to 5, presenting a promising prospect for further optimization. *Para*-substituted fluoride and methoxy group in the phenyl ring enable slightly more efficient kinetic resolution of racemic **7** and **8**, respectively. Gratifyingly, increasing the number of the methoxy groups in the phenyl ring led to a dramatic increase in the efficiencies for the kinetic resolution. With 3,4,5-tri-methoxyl-substitution in the extended phenyl ring, racemic **10** underwent catalytic kinetic resolution with a selectivity factor of 32, yielding the recovered (*R*)-**10** in 92% ee at 53% conversion. Finally, retaining the 3,4,5-tri-methoxyl-substituted phenyl ring in the conjugated π-system while changing the phthalazine to a pyridazine ring in racemic **11** resulted in a drastically decreased selectivity factor of 2, indicating the importance of the phthalazine in the non-covalent π interaction with the piperonylate of the catalyst.

**Fig. 3:**
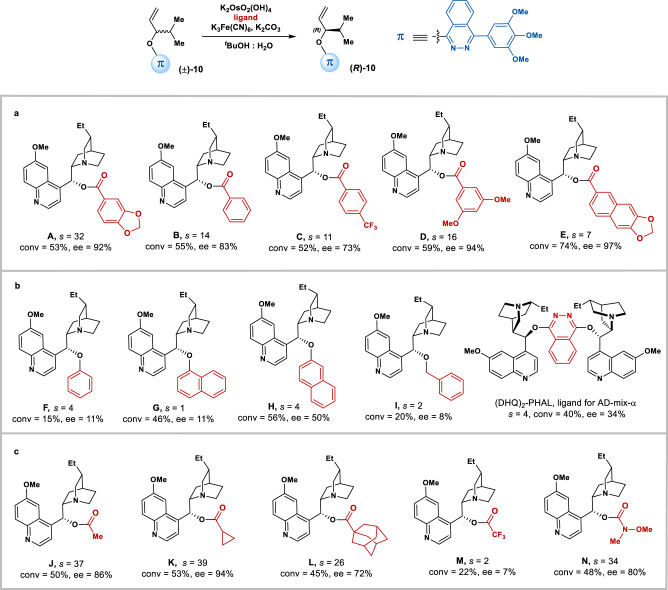
Exploring the nature of the non-covalent π-interaction. **a** The π-component at C9 of the cinchona alkaloid ligands with benzoate moiety. **b** The π-component at C9 of the cinchona alkaloid ligands with aromatic moiety. **c** The π-component at C9 of the cinchona alkaloid ligands only with carbonyl moiety. General condition: K_2_OsO_2_(OH)_4_ (0.4 mol%), K_3_Fe(CN)_6_ (3.0 equiv), K_2_CO_3_ (3.0 equiv), ligand (1.0 mol%), and (±)-**10** (0.1 mmol) in 1.0 mL ^*t*^BuOH-H_2_O (v/v = 1:1), 0 °C. Conversion was determined by ^1^H NMR analysis of the crude mixture.

We further investigated the nature of the non-covalent π interaction through varying the π-components at C9 of the cinchona alkaloids (Fig. 3). In addition to ligand **A**, it was found that benzoate moieties at C9 of **B**, **C**, and **D** (Fig. 3a) all lead to successful kinetic resolutions (*s* > 10). However, the benzoate moiety with extended conjugation in **E**, fails to lead to an efficient kinetic resolution (Fig. 3a). Unexpectedly, replacing the benzoate moiety with sole aryl rings, such as phenyl, naphthyl, benzyl or electron-deficient phthalazine groups, all leads to dramatic decreases in selectivity factors (ligands **F**, **G**, **H**, **I** and (DHQ)_2_PHAL, Fig. 3b), hinting on the critical role played by the carbonyl group in stereocontrol. Then we used aliphatic esters in the absence of aromatic rings, and high selectivity factors were surprisingly obtained (Fig. 3c). For instances, with simple ester groups, such as acetate in **J** and cyclopropanecarboxylate in **K**, (*R*)-**10** was kinetically resolved in 86% and 94% ee’s at around 50% conversions, respectively. These unexpected results imply that a lone-pair–π interaction between the carbonyl oxygen of the ligand and the π-auxiliary of the substrate^50,51^ might be involved in the stereoselectivity-determining transition state, which has not yet been reported as a controlling factor in asymmetric catalysis. Then increasing the steric hindrance of the ester group, for instances, using the adamantyl substituent in **L**, still results in a successful kinetic resolution. In contrast, when the trifloroacetate **M** was examined, the corresponding selectivity factor drastically decreased to 2. This remarkable electronic effect supports the lone-pair–π interaction as a key element in the AD-based kinetic resolution, since the electron-deficient trifloromethyl group might withdraw the lone-pair electrons of the carbonyl thus disrupt its interaction with π system in the ligand. A further evidence of the central role played by the lone-pair–π interaction was appreciable from the result obtained with ligand **N**, where the more electron-rich Weinreb amide moiety allows racemic **10** to be resolved with the selectivity factor of 34.

### Investigation on the origins of stereoselectivity

**Fig. 4:**
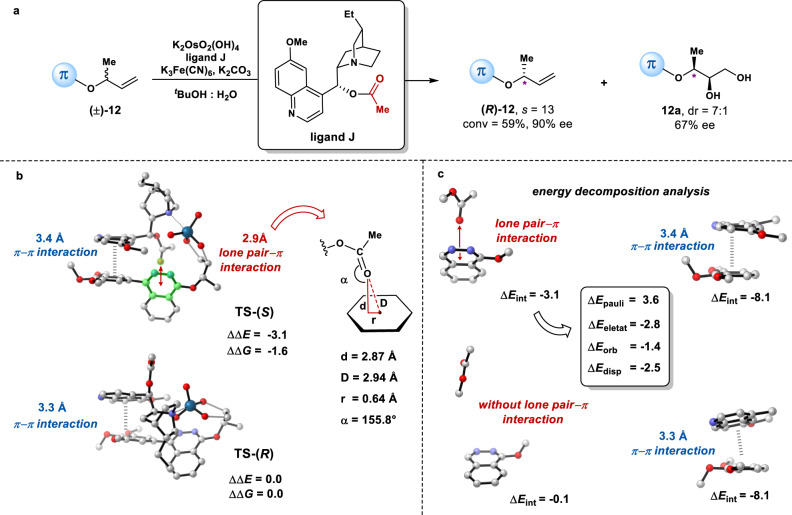
Combined experimental and computational investigation on the origins of stereoselectivity for the AD-based kinetic resolution using a model system. **a** Experimentally observed stereoselevtivitits for both recovered (***R***)**-12** and the diol product **12a** from the AD-based kinetic resolution. **b** Representative DFT-computed transition state structures TS-(*S*) and TS-(*R*) for this reaction. **c** Energy decomposition analysis for truncated structures of TS-(*S*) and TS-(*R*). Relative energies are given in kcal/mol. TS: transition state.

To understand the origins of chiral recognition for this kinetic resolution, we chose to use both the structurally simplest substrate (racemic **12**) and ligand (**J)** to perform the AD-based kinetic resolution (Fig. 4a). At 59% conversion, the recovered **12** was obtained in 90% ee with a selectivity factor of 13. The diol product **12a** was obtained with a diastereomeric ratio of 7:1 and the enantio-purity of the major diastereomer was found to be only modest (67% ee), as expected based on the proposed *bi*-functional catalysis modes in Fig. 1c. The absolute stereochemistry of the major enantiomer of the recovered **12** was confirmed to have (*R*)-configuration at its allylic carbon (for details, please see the Supplementary Information), indicating that the more reactive enantiomer of **12** has the (*S*)-configuration. The corresponding stereoselectivity-determining transition states (TSs) for this reaction were explored using density functional theory (DFT) calculations (See Supplementary Information for more details about computational methods, and supplementary data 1 for XYZ coordinates). Owing to the flexibility of the substrate and the ligand, thorough conformational searches for the TSs were performed (for details, please see Supplementary Information). The corresponding lowest energy TSs for the (*S*)- and (*R*)-substrates are shown in Fig. 4b. TS-(*S*) is lower in energy than TS-(*R*) (ΔΔ*G* = − 1.6 kcal/mol, ΔΔ*E* = − 3.1 kcal/mol), which supports the experimental result that (*R*)-**12** was kinetically resolved with good selectivity. In the [3 + 2] cycloaddition TSs, two C-O bonds are being formed in a concerted fashion^52^. There are no obvious steric clashes in the TSs. Distal to the forming bonds, two important sets of non-covalent interactions were identified. In both TSs, parallel displaced π–π interactions between the quinoline moiety of the ligand and the tri-methoxyphenyl group of the substrate are present (3.4 Å and 3.3 Å, respectively). In the favored transition state TS-(*S*), a lone-pair π interaction between the carbonyl group of the ligand and the phthalazine moiety of the substrate is evidenced clearly by the short distances of the carbonyl oxygen to the centroid of and to the plane of the diazine ring (2.94 Å and 2.87 Å, respectively)^25,53^. However, this type of interaction is absent in the disfavored transition state TS-(*R*), which may contribute to the difference in energy of these two TSs. Further calculations using truncated model show that the lone-pair π interaction is −3.1 kcal/mol, which is the main contributor to the stereoselectivity (Fig. 4c). The π–π interaction is relatively strong in its magnitude (−8.1 kcal/mol), which may contribute to the binding of substrate with the catalyst but plays a much smaller role in controlling the stereoselectivity, as both TSs have such an interaction in a similar magnitude. To unravel the nature of the lone-pair–π interactions, we performed energy decomposition analysis (EDA) (Fig. 4c, center box) to separate Δ*E*_int_ (−3.1 kcal/mol) into chemically meaningful energy terms, including Pauli repulsions (Δ*E*_pauli_ = 3.6 kcal/mol), electrostatic interactions (Δ*E*_eletat_ = −2.8 kcal/mol), orbital interactions (Δ*E*_orb_ = −1.4 kcal/mol) and London dispersion forces (Δ*E*_disp_ = −2.5 kcal/mol)^54^. These results indicate that electrostatic interactions and London dispersion forces are the two largest components, contributing 42% and 37% to the total stabilizing interactions, respectively.

### Reaction scope

**Fig. 5:**
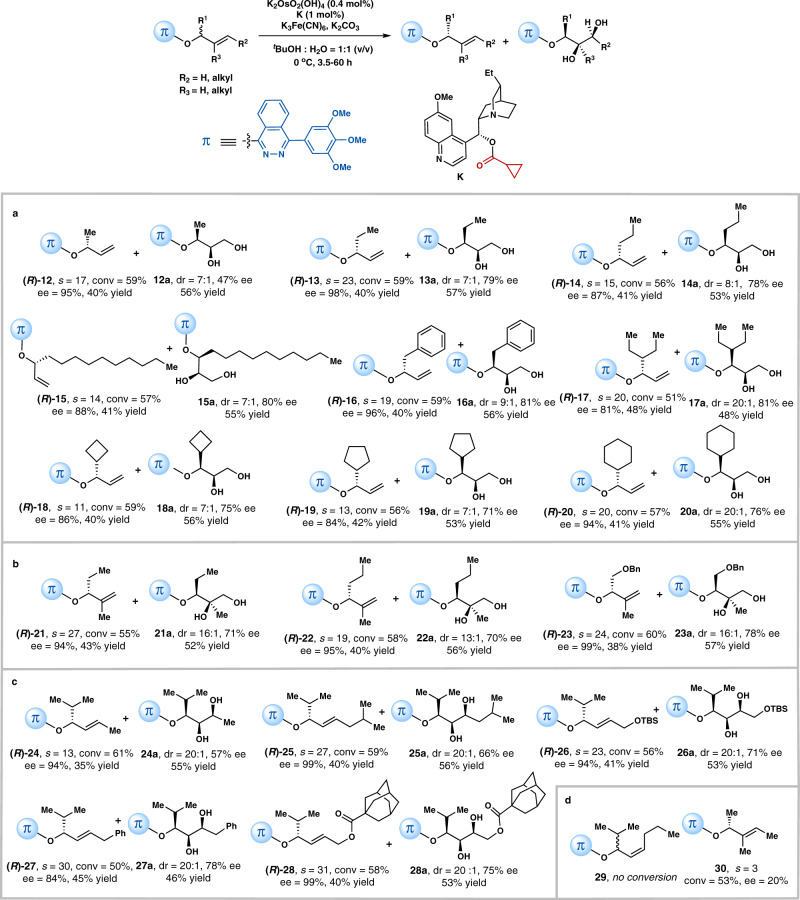
Scope for AD-based kinetic resolution controlled by the lone-pair–π interaction. **a** Racemic substrates with mono-substituted terminal alkenes. **b** Racemic substrates with 1,1-disubstituted terminal alkenes. **c** Racemic substrates with 1,2-(*E*)-disubstituted alkenes. **d** Unsuccessful substrates. General condition: K_2_OsO_2_(OH)_4_ (0.4 mol%), K_3_Fe(CN)_6_ (3.0 equiv), K_2_CO_3_ (3.0 equiv), ligand **K** (1.0 mol%) and allylic ether (0.1 mmol) in 1.0 mL ^*t*^BuOH-H_2_O (v/v = 1:1), 0 °C. Conversion (conv) and diastereomeric ratio (dr) were determined by ^1^H NMR analysis of the crude mixture. Isolated yields were given.

**Fig. 6:**
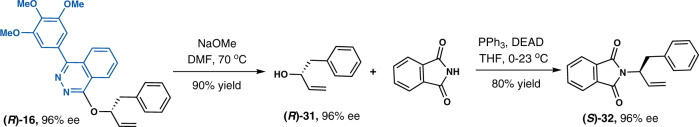
Synthetic application. DEAD: diethyl azodicarboxylate.

Having identified the crucial role of the lone-pair–π interaction between the cinchona alkaloid ligand and the alkene substrate in stereocontrol, we finally demonstrated the generality of the reaction scope (Fig. 5). A number of alkene substitution patterns in the racemic allylic ethers were found to be well accommodated, including 1-substituted, 1,1-disubstituted and (*E*)-1,2-disubstituted alkenes, and their corresponding diol products were also obtained. For mono-substituted alkenes, R^1^ group varying among methyl, ethyl, *n*-propyl, decyl, benzyl, 3-pentyl, cyclobutyl, cyclopentyl and cyclohexyl substituents were all compatible with this strategy. The resolved chiral allylic eithers were obtained with 84% to 97% ee’s at practical conversions (Fig. 5a). It is worth mentioning that these simple allylic ethers or their corresponding alcohols have been hardly accessed with high ee’s either by direct asymmetric reduction of prochiral ketones or by asymmetric 1,2 addition to aldehydes. Racemic allylic ethers bearing 1,1-disubstituted alkenes (Fig. 5b) and (*E*)-1,2-disubstituted alkenes (Fig. 5c) were also successfully resolved under identical conditions. The moderate stereoselectivities of the corresponding diols are not unexpected: the π-partner in the cinchona alkaloid ligand can only have this non-covalent π interaction with the introduced 3,4,5-tri-methoxyl-substituted phthalazine moiety instead of alkene’s double bond in the substrate, so that the “sandwich-like” mode that facilitates enantio-face control of the alkenes could not be formed (Fig. 1c, left). Unsuccessful substrates include (*Z*)-disubstituted alkenes and trisubstituted alkenes (Fig. 5d). Allylic ether **29** gave no conversion even with increased reaction time or catalyst loading, as *cis*-double bonds are challenging substrates in OsO_4_-catalyzed dihydroxylations^55^. *Tri*-substituted alkene **30** provided practical conversion but with low ee. As shown in Fig. 6, we are pleased to find that the π-moiety in (*R*)-**16** can be easily removed to reveal its alcohol version (*R*)-**31**, which can be further converted to chiral allylic amine (*S*)-**32** via Mitsunobu reaction without loss of the stereochemical fidelity.

In conclusion, we have developed a highly efficient catalytic system based on modified cinchona alkaloids. A *bi*-functional catalysis strategy was proposed and subsequently tested in the Sharpless asymmetric dihydroxylation-based kinetic resolution of racemic olefins. Rationally designed and well-engineered non-covalent π interactions are incorporated as key elements for stereocontrol to tackle this persistent challenge in asymmetric catalysis. A relatively weak lone-pair–π interaction between the ligand and the substrate was discovered to play a crucial role in differentiating the enantiomeric substrates bearing 1-substituted, 1,1-substituted and (*E*)-alkenes during chiral recognition. Fully integrated experimental and computational studies provide strong support of such non-covalent π interactions in the stereodetermining transition states. The search of a rigid system bearing a π-auxiliary to facilitate AD-based kinetic resolution of the less reactive *Z*-alkenes is underway. Further investigations and applications exploiting the lone-pair–π interaction that was originally discovered in biological systems are expected to reveal whether nature also utilizes this type of non-covalent force in the biosynthesis of natural products and guide the development of new chiral catalysts in the laboratory. We anticipate that this discovery will stimulate wider utilization and manipulation of non-covalent π interactions at the outset of rational catalyst design to solve other challenging problems in asymmetric catalysis.

## Methods

### Representative procedure for Sharpless asymmetric dihydroxylation-based kinetic resolution of racemic allylic ether 13

To a 20 mL glass tube containing a magnetic stir bar was charged with K_3_Fe(CN)_6_ (98.7 mg, 3.0 equiv.), K_2_CO_3_ (41.2 mg, 3.0 equiv.) and racemic allylic ether **13** (0.1 mmol, 1.0 equiv.). To a 50 mL round bottom flask, stock solution of K_2_OsO_2_(OH)_4_ (2.9 mg) and ligand **K** (7.9 mg) was prepared with 20 mL of ^*t*^BuOH-H_2_O (*v*/*v* = 1:1). 1.0 mL of the above stock solution was also added to the glass tube. Then the reaction mixture was cooled to 0 °C and stirred at this temperature for 13 h, which was quenched with saturated aqueous Na_2_S_2_O_3_ (2.0 mL) at room temperature and extracted with ethyl acetate (2.0 mL × 3). The combined organic layer was dried over Na_2_SO_4_ and concentrated in vacuo. The selectivity factor (*s*) was calculated through the equation: *s* = In[(1 − *c*)(1 − ee)]/In[(1 − *c*)(1+ee)]. The conversion (c) of the reaction (*c* = 59%) and diastereomeric ratio (dr = 7:1) of the diol product **13a** were determined by ^1^H NMR analysis. Enantiomeric excesses (ees) of both (*R*)-**13** (98% ee) and **13a** (79% ee) were determined by chiral high-performance liquid chromatography (HPLC) analysis. The residue was purified by column chromatography (petroleum ether/ethyl acetate = 3:1 to 1:2) on silica gel to give the allylic ether (*R*)-**13** (15.2 mg, 40% yield) and diol product **13a** (23.6 mg, 57% yield).

## Supplementary information

Supplementary Information

Description of Additional Supplementary Files

Supplementary Data 1

## Data Availability

The data that support the findings of this study are available within the paper and its supplementary information files. Raw data are available from the corresponding author on request. Materials and methods, experimental procedures, characterization data, ^1^H, ^13^C, NMR spectra and mass spectrometry data are available in the Supplementary Materials.
