# Supplementary material for: Engineered non-covalent π interactions as key elements for chiral recognition
Source: Nat Commun. 2022 Jun 7;13:3276. doi: 10.1038/s41467-022-31026-8 (PMC9174283; doi:10.1038/s41467-022-31026-8)
Supplement: Supplementary file 1 — Supplementary Information [file 41467_2022_31026_MOESM1_ESM.pdf]

Supplementary Information

Engineered Non-Covalent Interactions as Key Elements for Chiral Recognition

Ming Yu Jin[§], Qianqian Zhen[§], Dengmengfei Xiao[§], Guanyu Tao, Xiangyou Xing, Peiyuan Yu* and Chen Xu*

Department of Chemistry and Shenzhen Grubbs Insitute, Guangdong Provincial Key Laboratory of Catalysis, Southern University of Science and Technolgy, Shenzhen, 518055, China

[§]These authors contributed equally to this work.

E-mail: xuc@sustech.edu.cn; yupy@sustech.edu.cn.

1. Supplementary Notes 3

2. Supplementary Methods 4

2.1 Preparation of Racemic Allylic Ethers.. 4

2.2 Preparation of Cinchona Alkaloid Ligands. 19

2.3 General Procedure for AD-Based Kinetic Resolution. 28

2.4 Screening an appropriate π in the racemic allylic ethers for AD-based kinetic resolution.. 29

2.5 Screening and optimization of *O*-substituent in cinchona alkaloids.. 62

2.6 Substrate scopes.. 81

2.7 Unsuccessful substrates 166

2.8 Synthetic Application 171

3 Supplementary Discussion 175

3.1 Determination of absolute configuration of **(±)-12** 175

3.2 Computational studies. 182

3.2.1. Computational methods 182

3.2.2. Conformational analysis 184

3.2.3. Stereoselectivity-determining transition structures 189

3.2.4. DFT-computed free energy profile 190

3.2.5. Calculations of stabilizing non-covalent interaction 191

4 Supplementary Figures.. 193

4.1 NMR spectra 193

5 Supplementary References 268

1. Supplementary Notes

All reactions were carried out under an argon atmosphere with dry solvents under anhydrous conditions, unless otherwise noted. All the chemicals were purchased commercially, and used without further purification. Anhydrous THF was distilled from sodium-benzophenone. The dichloromethane, triethylamine and N,N-dimethylformamide were distilled from calcium hydride. Thin-layer chromatography (TLC) was conducted with 0.25 mm Tsingtao silica gel plates (60F-254) and visualized by exposure to UV light (254 nm). Flash column chromatography was performed using Tsingtao silica gel (60, particle size 0.040–0.063 mm). Reagents were purchased at the highest commercial quality and used without further purification, unless otherwise stated. ^{1}H NMR (400 MHz and 600 MHz), ^{13}C NMR (101 MHz and 151 MHz), ^{19}F NMR (565 MHz and 376 MHz) spectra were recorded on a Bruker AV III HD spectrometer, and were reported in terms of chemical shift relative to residual $CDCl_3$ (δ 7.26 and δ 77.0 ppm, respectively). Data for ^{1}H NMR spectra are reported as follows: chemical shift (δ ppm) (multiplicity, coupling constant (Hz), integration). Abbreviations are used as follows: s = singlet, br = broad singlet, d = doublet, t = triplet, q = quartet, m = complex multiplet. Data for ^{13}C NMR spectra are reported in terms of chemical shift. High-resolution mass spectra (HRMS) data was obtained by using Thermo Scientific™ Q Exactive™ Quadrupole-Orbitrap Mass Spectrometer. HPLC analysis was conducted on Agilent 1260 instrument and Waters ACQUITY UPC2 instrument using chiral column described below in detail. Specific optical rotation was measured on a Rudolph-Autopol I.

2. Supplementary Methods

2.1 Preparation of Racemic Allylic Ethers.

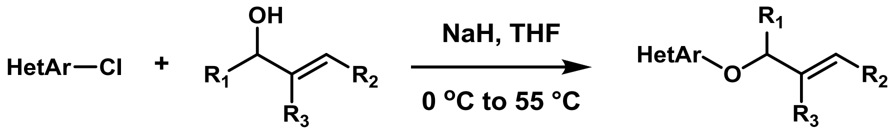

General Procedure I: A dry round-bottom flask equipped with a magnetic stir bar was charged with dry THF (20.0 mL) and the flask was cooled to 0 °C at under N_2. The NaH (60% oil suspension, 288 mg, 20.0 mmol, 2.0 equiv.) was added in one portion and the suspension was stirred for 10 minutes. Next, a solution of allylic alcohol (11.0 mmol, 1.1 equiv.) in dry THF (10.0 mL) was added to the suspension at 0 °C. After 10 minutes, the aryl chloride (10.0 mmol, 1.0 equiv.) was added to reaction mixture. The reaction mixture was warmed to 55 °C and stirred for 10 h. Then the mixture was quenched with the addition of H_2O (10 mL) and the resulting layers were separated, and the aqueous phase was extracted with EtOAc (3 x 10 mL). The organic phase was washed with brine, dried over Na_2SO_4 and filtered. The crude residue was purified by flash column chromatography on silica gel with petroleum ether/EtOAc (10:1) to afford the corresponding products. Characterization of compounds **(±)-1-30** were described below in detail:

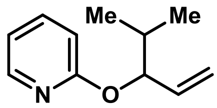

(±)-**1**

2-((4-methylpent-1-en-3-yl)oxy)pyridine

^{1}H NMR (400 MHz, CDCl$_3$): δ 8.13 – 8.11 (m, 1H), 7.57 – 7.52 (m, 1H), 6.83 – 6.80 (m, 1H), 6.75 – 6.73 (m, 1H), 5.93 – 5.85 (m, 1H), 5.39 – 5.36 (m, 1H), 5.28 – 5.16 (m, 2H), 2.04 – 1.99 (m, 1H), 0.99 (d, J = 8.0 Hz, 6H) ppm.

^{13}C NMR of (101 MHz, CDCl$_3$): δ 163.8, 146.9, 138.6, 138.4, 135.9, 116.7, 116.5, 111.4, 79.7, 32.3, 18.3, 18.1 ppm.

HRMS (ESI) m/z: $[M + H]^+$ Calcd for $C_{11}H_{16}NO$ 178.1226; Found 178.1228.

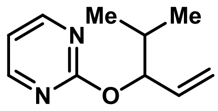

(±)-**2**

2-((4-methylpent-1-en-3-yl)oxy)pyrimidine

^{1}H NMR (400 MHz, CDCl$_3$): δ 8.47 – 8.45 (m, 2H), 7.26 – 6.84 (m, 1H), 5.92 – 5.80 (m, 1H), 5.36 – 5.25 (m, 2H), 5.20 – 5.16 (m, 1H), 2.04 – 2.01 (m, 1H), 0.99 (d, *J* = 8.0 Hz, 6H) ppm.

^{13}C NMR (101 MHz, CDCl$_3$): δ 165.1, 159.2, 135.2, 117.3, 114.7, 82.0, 32.1, 18.2, 18.0 ppm.

HRMS (ESI) m/z: [M + H]$^+$ Calcd for $C_{10}H_{15}N_2O$ 179.1179; Found 179.1180.

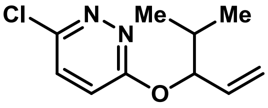

(±)-**3**

3-chloro-6-((4-methylpent-1-en-3-yl)oxy)pyridazine

^{1}H NMR (600 MHz, CDCl$_3$): δ 7.35 – 7.33 (m, 1H), 6.95 – 6.93 (m, 1H), 5.85 – 5.80 (m, 1H), 5.61 – 5.59 (m, 1H), 5.34 – 5.31 (m, 1H), 5.25 – 5.23 (m, 1H), 2.10 – 2.04 (m, 1H), 0.99 (d, *J* = 8.0 Hz, 6H) ppm.

^{13}C NMR (151 MHz, CDCl$_3$): δ 164.2, 150.6, 134.4, 130.8, 120.4, 118.6, 82.3, 32.0, 18.2, 17.9 ppm.

HRMS (ESI) m/z: [M + H]$^+$ Calcd for $C_{10}H_{14}ClN_2O$ 213.0789; Found 213.0780.

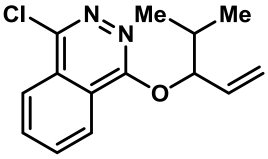

(±)-**4**

1-chloro-4-((4-methylpent-1-en-3-yl)oxy)phthalazine

^{1}H NMR (400 MHz, CDCl$_3$): δ 8.29 – 8.26 (m, 1H), 8.19 – 8.17 (m, 1H), 7.95 – 7.91 (m, 2H), 5.99 – 5.86 (m, 1H), 5.85 – 5.83 (m, 1H), 5.43 – 5.38 (m, 1H), 5.28 – 5.25 (m, 1H), 2.24 – 2.16 (m, 1H), 1.07 (d, *J* = 8.0 Hz, 6H) ppm.

^{13}C NMR (151 MHz, CDCl$_3$): δ 160.0, 149.7, 134.5, 133.0, 132.8, 127.8, 125.2, 123.6, 122.0, 118.3, 82.0, 32.1, 18.14, 18.06 ppm.

HRMS (ESI) m/z: [M + H]$^+$ Calcd for $C_{14}H_{16}ClN_2O$ 263.0946; Found 263.0950.

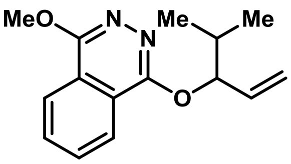

(±)-**5**

1-methoxy-4-((4-methylpent-1-en-3-yl)oxy)phthalazine

^{1}H NMR (400 MHz, CDCl$_3$): δ 8.20 – 8.12 (m, 1H), 8.11 – 8.05 (m, 1H), 7.87 – 7.74 (m, 2H), 5.95 (ddd, J_1 = 17.1 Hz, J_2 =10.6 Hz, J_3 = 6.4 Hz, 1H), 5.75 (t, J = 5.8 Hz, 1H), 5.37 (dd, J = 17.2, 1.0 Hz, 1H), 5.22 (d, J = 10.6 Hz, 1H), 4.16 (s, 3H), 2.17 (dq, J = 13.4, 6.6 Hz, 1H), 1.04 (dd, J = 12.9, 6.8 Hz, 6H) ppm.

^{13}C NMR (151 MHz, CDCl$_3$): δ 157.8, 157.4, 135.2, 131.8, 123.08, 123.07, 122.8, 122.5, 117.3, 80.9, 54.5, 32.1, 18.2, 18.1 ppm.

HRMS (ESI) m/z: $[M + H]^+$ Calcd for $C_{15}H_{19}N_2O_2$ 259.1441; Found 259.1445.

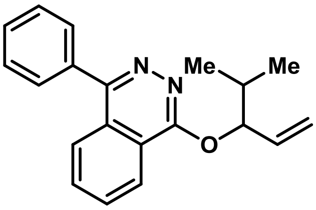

(±)-**6**

1-((4-methylpent-1-en-3-yl)oxy)-4-phenylphthalazine

^{1}H NMR (600 MHz, CDCl$_3$): δ 8.36 – 8.34 (m, 1H), 7.98 – 7.97 (m, 1H), 7.87 – 7.84 (m, 1H), 7.81 – 7.78 (m, 1H), 7.72 – 7.71 (m, 2H), 7.54 – 7.50 (m, 3H), 6.03 – 5.97 (m, 2H), 5.47 – 5.45 (m, 1H), 5.28 – 5.27 (m, 1H), 2.27 – 2.23 (m, 1H), 1.10 (d, J = 6.0 Hz, 6H) ppm.

^{13}C NMR (151 MHz, CDCl$_3$): δ 159.4, 156.3, 136.6, 134.9, 131.9, 131.5, 130.0, 128.9 128.4, 127.9, 126.2, 123.2, 120.6, 118.0, 81.3, 32.2, 18.2, 18.1 ppm.

HRMS (ESI) m/z: $[M + H]^+$ Calcd for $C_{20}H_{21}N_2O$ 305.1648; Found 305.1642.

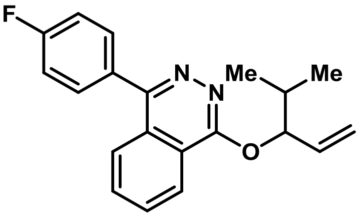

(±)-**7**

1-(4-fluorophenyl)-4-((4-methylpent-1-en-3-yl)oxy)phthalazine

¹H NMR (600 MHz, CDCl₃): δ 8.36 – 8.34 (m, 1H), 7.94 – 7.92 (m, 1H), 7.86 – 7.85 (m, 1H), 7.82 – 7.81 (m, 1H), 7.71 – 7.69 (m, 2H), 7.23 – 7.20 (m, 2H), 6.02 – 5.95 (m, 2H), 5.47 – 5.43 (m, 1H), 5.29 – 5.27 (m, 1H), 2.26 – 2.22 (m, 1H), 1.10 (d, J = 6.0 Hz, 6H) ppm.

^{13}C NMR (151 MHz, $CDCl_3$): δ 163.3 (d, J_{CF} = 248.4 Hz), 159.4, 155.3, 134.9, 132.7 (d, J_{CF} = 3.2 Hz), 132.1, 131.7, 131.7, 131.6, 124.6 (d, J_{CF} = 383.6 Hz), 116.7 (d, J_{CF} = 392.4 Hz), 115.5, 81.4, 32.2, 18.2, 18.1 ppm.

^{19}F NMR (376 MHz, $CDCl_3$): δ –112.7 ppm.

HRMS (ESI) m/z: $[M + H]^+$ Calcd for $C_{20}H_{20}FN_2O$ 323.1554; Found 322.1550.

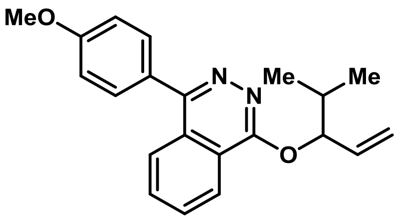

(±)-**8**

1-(4-methoxyphenyl)-4-((4-methylpent-1-en-3-yl)oxy)phthalazine

^{1}H NMR (400 MHz, $CDCl_3$): δ 8.35 – 8.33 (m, 1H), 8.02 – 8.00 (m, 1H), 7.86 – 7.80 (m, 2H), 7.69 – 7.65 (m, 2H), 7.07 – 7.05 (m, 2H), 6.04 – 5.94 (m, 2H), 5.47 – 5.43 (m, 1H), 5.29 – 5.26 (m, 1H), 3.90 (s, 3H), 2.28 – 2.20 (m, 1H), 1.08 (d, J = 8.0 Hz, 6H) ppm.

^{13}C NMR (151 MHz, $CDCl_3$): δ 160.3, 159.2, 155.9, 135.0, 131.9, 131.4, 131.3, 129.1, 127.9, 126.3, 123.2, 120.7, 117.9, 113.9, 81.3, 55.4, 32.2, 18.2, 18.1 ppm.

HRMS (ESI) m/z: $[M + H]^+$ Calcd for $C_{21}H_{23}N_2O_2$ 335.1754; Found 335.1761.

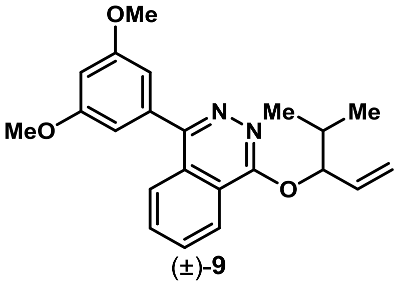

(±)-**9**

1-(3,5-dimethoxyphenyl)-4-((4-methylpent-1-en-3-yl)oxy)phthalazine

^{1}H NMR (600 MHz, $CDCl_3$): δ 8.30 – 8.29 (m, 1H), 8.10 – 7.99 (m, 1H), 7.82 – 7.74 (m, 2H), 6.82 – 6.81 (m, 2H), 6.57 – 6.56 (m, 1H), 5.98 – 5.91 (m, 2H), 5.42 – 5.38 (m, 1H), 5.24 – 5.21 (m, 1H), 3.80 (s, 6H), 2.21 – 2.18 (m, 1H), 1.05 (d, J = 12.0 Hz, 6H) ppm.

^{13}C NMR (151 MHz, CDCl$_3$): δ 160.7, 159.5, 156.1, 138.4, 134.9, 132.0, 131.6, 127.8, 126.2, 123.2, 120.5, 118.0, 108.1, 101.4, 81.4, 55.6, 32.2, 18.2, 18.1 ppm.

HRMS (ESI) m/z: [M + H]$^+$ Calcd for $C_{22}H_{25}N_2O_3$ 365.1859; Found 365.1860.

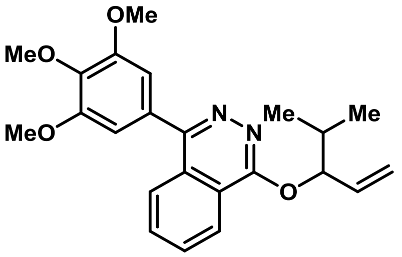

(±)-**10**

1-((4-methylpent-1-en-3-yl)oxy)-4-(3,4,5-trimethoxyphenyl)phthalazine

^{1}H NMR (600 MHz, CDCl$_3$): δ 8.36 – 8.34 (m, 1H), 8.06 – 8.04 (m, 1H), 7.88 – 7.81 (m, 2H), 6.96 – 6.90 (m, 2H), 6.02 – 5.95 (m, 2H), 5.46 – 5.25 (m, 1H), 3.93 (s, 3H), 3.89 (s, 6H), 2.26 – 2.21 (m, 1H), 1.09 (d, J = 12.0 Hz, 6H) ppm.

^{13}C NMR (151 MHz, CDCl$_3$): δ 159.4, 156.1, 153.2, 138.8, 134.9, 132.1, 132.0, 131.6, 127.8, 126.1, 123.3, 120.6, 117.9, 107.3, 81.4, 61.0, 56.3, 32.2, 18.2, 18.1 ppm.

HRMS (ESI) m/z: [M + H]$^+$ Calcd for $C_{23}H_{27}N_2O_4$ 395.1965; Found 395.1960.

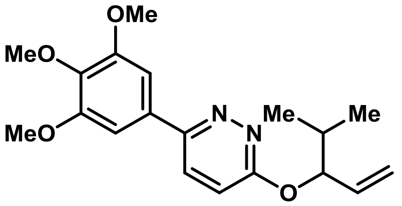

(±)-**11**

3-((4-methylpent-1-en-3-yl)oxy)-6-(3,4,5-trimethoxyphenyl)pyridazine

^{1}H NMR (600 MHz, CDCl$_3$): δ 7.76 – 7.75 (m, 1H), 7.27 – 7.26 (m, 2H), 7.04 – 7.02 (m, 1H), 5.91 – 5.88 (m, 1H), 5.77 – 5.75 (m, 1H), 5.38 – 5.34 (m, 1H), 5.26 – 5.24 (m, 1H), 3.94 (s, 6H), 3.90 (s, 3H), 2.13 – 2.12 (m, 1H), 1.02 (d, J = 6.0 Hz, 6H) ppm.

^{13}C NMR (151 MHz, CDCl$_3$): δ 164.0, 154.4, 153.6, 139.4, 134.9, 131.8, 127.0, 118.1, 118.0, 103.8, 81.7, 61.0, 56.3, 32.0, 18.2, 17.9 ppm.

HRMS (ESI) m/z: [M + H]$^+$ Calcd for $C_{19}H_{25}N_2O_4$ 345.1809; Found 345.1808.

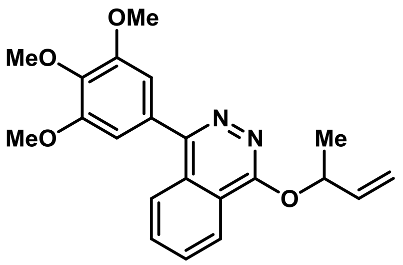

(±)-**12**

1-(but-3-en-2-yloxy)-4-(3,4,5-trimethoxyphenyl)phthalazine

^{1}H NMR (600 MHz, CDCl$_3$): δ 8.32 – 8.30 (m, 1H), 8.03 – 8.02 (m, 1H), 7.85 – 7.9 (m, 2H), 6.92 (m, 2H), 6.20 – 6.17 (m, 1H), 6.14 – 6.09 (m, 1H), 5.45 – 5.42 (m, 1H), 5.21 – 5.19 (m, 1H), 3.91 (s, 3H), 3.88 (s, 6H), 1.60 (d, *J* = 6.0 Hz, 3H) ppm.

^{13}C NMR (151 MHz, CDCl$_3$): δ 159.0, 156.1, 153.2, 138.7, 138.3, 132.98, 132.96, 131.5, 127.7, 125.9, 123.3, 120.5, 115.7, 107.3, 73.1, 60.9, 56.2, 20.1 ppm.

HRMS (ESI) m/z: $[M + H]^+$ Calcd for $C_{21}H_{23}N_2O_4$ 367.1652; Found 367.1655.

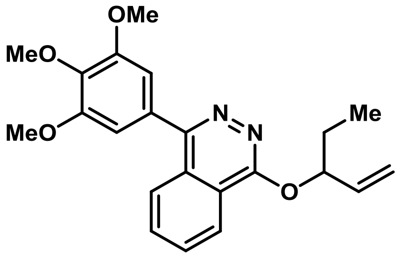

(±)-**13**

1-(pent-1-en-3-yloxy)-4-(3,4,5-trimethoxyphenyl)phthalazine

^{1}H NMR (600 MHz, CDCl$_3$): 8.33 – 8.32 (m, 1H), 8.04 – 8.02 (m, 1H), 7.85 – 7.79 (m, 2H), 6.92 (s, 2H), 6.05 – 5.99 (m, 2H), 5.45 – 5.41 (m, 1H), 5.24 – 5.21 (m, 1H), 3.91 (s, 3H), 3.87 (s, 6H), 2.01 – 1.88 (m, 2H), 1.03 (t, *J* = 12.0 Hz, 3H) ppm.

^{13}C NMR (151 MHz, CDCl$_3$): δ 159.2, 156.0, 153.2, 138.7, 136.7, 132.0, 131.5, 127.7, 126.0, 123.2, 120.5, 116.9, 107.3, 78.0, 60.9, 56.2, 27.3, 9.4 ppm.

HRMS (ESI) m/z: $[M + H]^+$ Calcd for $C_{22}H_{25}N_2O_4$ 381.1809; Found 381.1809.

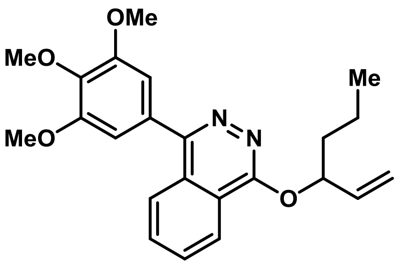

(±)-**14**

1-(hex-1-en-3-yloxy)-4-(3,4,5-trimethoxyphenyl)phthalazine

^{1}H NMR (600 MHz, CDCl$_3$): δ 8.32 – 8.30 (m, 1H), 8.03 – 8.02 (m, 1H), 7.84 – 7.78 (m, 2H), 6.92 (s, 2H), 6.12 – 6.09 (m, 1H), 6.04 – 5.99 (m, 1H), 5.43 – 5.40 (m, 1H), 5.21 – 5.19 (m, 1H), 3.91 (s, 3H), 3.87 (s, 6H), 1.99 – 1.79 (m, 2H), 1.55 – 1.46 (m, 2H), 0.96 (t, *J* = 12.0 Hz, 3H) ppm.

^{13}C NMR (151 MHz, CDCl$_3$): δ 159.2, 156.0, 153.1, 138.7, 137.0, 132.0, 131.9, 131.5, 127.7, 125.9, 123.2, 120.4, 116.6, 107.3, 76.7, 60.9, 56.2, 36.5, 18.4, 13.9 ppm.

HRMS (ESI) m/z: $[M + H]^+$ Calcd for $C_{23}H_{27}N_2O_4$ 395.1965; Found 395.1972.

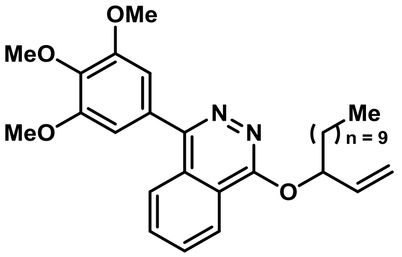

(±)-**15**

1-(pent-1-en-3-yloxy)-4-(3,4,5-trimethoxyphenyl)phthalazine

^{1}H NMR (600 MHz, CDCl$_3$): δ 8.27 – 8.26 (m, 1H), 7.98 – 7.97 (m, 1H), 7.80 – 7.73 (m, 2H), 6.87 (s, 2H), 6.05 – 6.02 (m, 1H), 5.99 – 5.93 (m, 1H), 5.38 – 5.35 (m, 1H), 5.16 – 5.14 (m, 1H), 3.86 (s, 3H), 3.82 (s, 6H), 1.95 – 1.88 (m, 1H), 1.82 – 1.76 (m, 1H), 1.47 – 1.36 (m, 2H), 1.31 – 1.26 (m, 2H), 1.23 – 1.16 (m, 14H), 0.78 (t, *J* = 6.0 Hz, 3H) ppm.

^{13}C NMR (151 MHz, CDCl$_3$): δ 159.3, 156.0, 153.2, 138.7, 137.1, 132.0, 132.0, 131.5, 127.7, 126.0, 123.3, 120.5, 116.7, 107.3, 77.0, 61.0, 56.3, 34.5, 31.9, 29.62, 29.59, 29.55, 29.5, 29.3, 25.2, 22.7, 14.1 ppm.

HRMS (ESI) m/z: $[M + H]^+$ Calcd for $C_{30}H_{41}N_2O_4$ 493.3061; Found 493.3065.

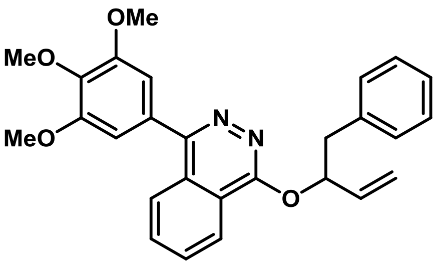

(±)-**16**

1-((1-phenylbut-3-en-2-yl)oxy)-4-(3,4,5-trimethoxyphenyl)phthalazine

^{1}H NMR (600 MHz, $CDCl_3$): δ 8.32 – 8.31 (m, 1H), 8.05 – 8.04 (m, 1H), 7.88 – 7.82 (m, 2H), 7.36 – 7.35 (m, 2H), 7.29 – 7.26 (m, 2H), 7.21 – 7.18 (m, 1H), 6.93 (s, 2H), 6.36 – 6.33 (m, 1H), 6.09 – 6.04 (m, 1H), 5.43 – 5.40 (m, 1H), 5.24 – 5.22 (m, 1H), 3.94 (s, 3H), 3.90 (s, 6H), 3,38 – 3.34 (m, 1H), 3.20 – 3.16 (m, 1H) ppm.

^{13}C NMR (151 MHz, $CDCl_3$): δ 159.1, 156.4, 153.3, 138.8, 137.3, 136.2, 132.1, 132.0, 131.7, 129.8, 128.3, 127.8, 126.5, 126.1, 123.3, 120.5, 117.3, 107.3, 61.0, 56.3, 41.1 ppm.

HRMS (ESI) m/z: $[M + H]^+$ Calcd for $C_{27}H_{27}N_2O_4$ 443.1965; Found 443.1966.

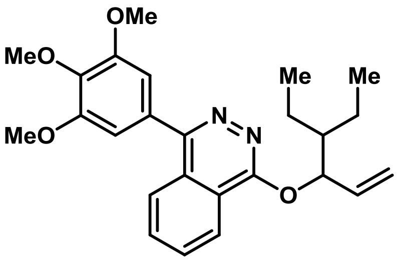

(±)-**17**

1-((4-ethylhex-1-en-3-yl)oxy)-4-(3,4,5-trimethoxyphenyl)phthalazine

^{1}H NMR (400 MHz, $CDCl_3$): δ 8.31 – 8.28 (m, 1H), 8.03 – 8.01 (m, 1H), 7.85 – 7.77 (m, 2H), 6.92 (s, 2H), 6.22 – 6.18 (m, 1H), 6.00 – 5.92 (m, 1H), 5.42 – 5.17 (m, 1H), 5.23 – 5.20 (m, 1H), 3.90 (s, 3H), 3.86 (s, 6H), 1.78 – 1.72 (m, 1H), 1.65 – 1.49 (m, 3H), 1.46 – 1.37 (m, 1H), 1.00 – 0.93 (m, 6H) ppm.

^{13}C NMR (101 MHz, $CDCl_3$): δ 159.2, 155.9, 153.1, 138.6, 135.2, 131.92, 131.88, 131.5, 127.6, 125.9, 123.1, 120.4, 117.3, 107.2, 78.4, 60.8, 56.2, 45.1, 22.00, 21.97, 11.7, 11.6 ppm.

HRMS (ESI) m/z: $[M + H]^+$ Calcd for $C_{25}H_{31}N_2O_4$ 423.2278; Found 423.2288.

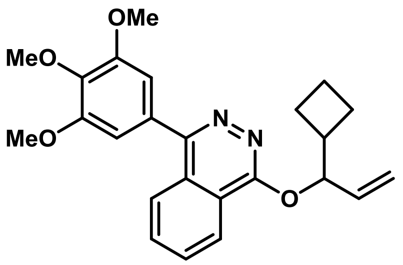

(±)-**18**

1-((1-cyclobutylallyl)oxy)-4-(3,4,5-trimethoxyphenyl)phthalazine

^{1}H NMR (400 MHz, $CDCl_3$): δ 8.34 – 8.32 (m, 1H), 8.04 – 8.02 (m, 1H), 7.87 – 7.78 (m, 2H), 6.93 (s, 2H), 6.11 – 6.07 (m, 1H), 5.98 – 5.89 (m, 1H), 5.43 – 5.38 (m, 1H), 5.21 – 5.18 (m, 1H), 3.91 (s, 6H), 3.87 (s, 3H), 2.87 – 2.78 (m, 1H), 2.12 – 2.01 (m, 3H), 1.99 – 1.85 (m, 3H) ppm.

^{13}C NMR (101 MHz, $CDCl_3$): δ 159.6, 156.0, 153.1, 138.7, 134.9, 132.0, 131.5, 127.7, 126.0, 123.2, 120.4, 117.2, 107.3, 79.7, 60.9, 56.2, 38.8, 24.3, 24.0, 18.2 ppm.

HRMS (ESI) m/z: $[M + H]^+$ Calcd for $C_{24}H_{27}N_2O_4$ 407.1965; Found 407.1969.

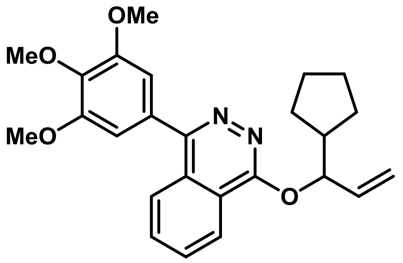

(±)-**19**

1-((1-cyclopentylallyl)oxy)-4-(3,4,5-trimethoxyphenyl)phthalazine

^{1}H NMR (600 MHz, $CDCl_3$): δ 8.34 – 8.33 (m, 1H), 8.05 – 8.04 (m, 1H), 7.87 – 7.81 (m, 2H), 6.94 (s, 2H), 6.05 – 5.98 (m, 2H), 5.47 – 5.43 (m, 1H), 5.23 – 5.21 (m, 1H), 3.93 (s, 3H), 3.89 (s, 6H), 2.47 – 2.43 (m, 1H), 1.91 – 1.86 (m, 1H), 1.84 – 1.75 (m, 1H), 1.69 – 1.55 (m, 2H), 1.65 – 1.56 (m, 3H), 1.51 – 1.43 (m, 1H) ppm.

^{13}C NMR (151 MHz, $CDCl_3$): δ 159.5, 156.0, 153.2, 138.7, 136.1, 132.1, 132.0, 131.6, 127.8, 126.1, 123.3, 120.6, 117.5, 107.3, 80.4, 61.0, 56.3, 44.0, 28.8, 28.7, 25.7, 25.6 ppm.

HRMS (ESI) m/z: $[M + H]^+$ Calcd for $C_{25}H_{29}N_2O_4$ 421.2122; Found 421.2128.

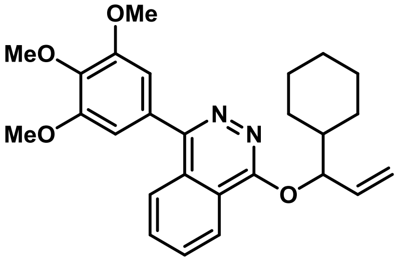

(±)-**20**

1-((1-cyclohexylallyl)oxy)-4-(3,4,5-trimethoxyphenyl)phthalazine

^{1}H NMR (600 MHz, CDCl$_3$): δ 8.35 – 8.34 (m, 1H), 8.06 – 8.04 (m, 1H), 7.88 – 7.81 (m, 2H), 6.94 (s, 2H), 6.02 – 5.96 (m, 2H), 5.44 – 5.40 (m, 1H), 5.27 – 5.24 (m, 1H), 3.93 (s, 3H), 3.89 (s, 6H), 1.98 – 1.87 (m, 3H), 1.79 – 1.67 (m, 4H), 1.30 – 1.17 (m, 5H) ppm.

^{13}C NMR (151 MHz, CDCl$_3$): δ 159.4, 156.0, 153.2, 138.7, 135.3, 132.1, 132.0, 131.6, 127.8, 126.1, 123.3, 120.6, 117.8, 107.3, 80.9, 61.0, 56.3, 41.9, 28.64, 28.60, 26.5, 26.10, 26.08 ppm.

HRMS (ESI) m/z: $[M + H]^+$ Calcd for $C_{26}H_{31}N_2O_4$ 435.2278; Found 435.2280.

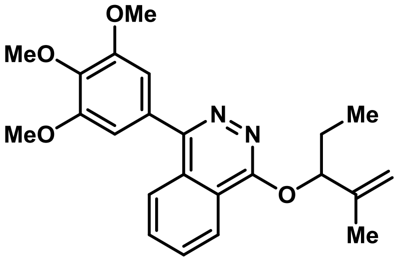

(±)-**21**

1-((2-methylpent-1-en-3-yl)oxy)-4-(3,4,5-trimethoxyphenyl)phthalazine

^{1}H NMR (400 MHz, CDCl$_3$): δ 8.36 – 8.34 (m, 1H), 8.06 – 8.04 (m, 1H), 7.87 – 7.82 (m, 2H), 6.94 (s, 2H), 5.98 – 5.94 (m, 1H), 5.14 (s, 1H), 4.95 (s, 1H), 3.93 (s, 3H), 3.89 (s, 6H), 2.04 – 1.89 (m, 2H), 1.87 (s, 3H), 1.03 (t, J = 8.0 Hz, 3H) ppm.

^{13}C NMR (101 MHz, CDCl$_3$): δ 159.3, 156.0, 155.9, 153.2, 143.3, 132.0, 131.6, 127.8, 126.1, 123.3, 120.6, 112.8, 107.3, 80.7, 61.0, 56.3, 25.9, 18.5, 9.8 ppm.

HRMS (ESI) m/z: $[M + H]^+$ Calcd for $C_{23}H_{27}N_2O_4$ 395.1965; Found 395.1966.

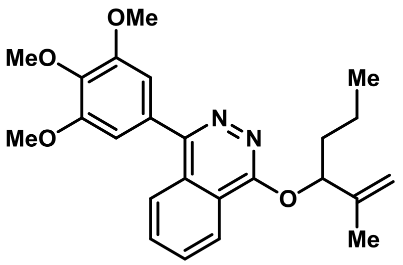

(±)-**22**

1-((2-methylhex-1-en-3-yl)oxy)-4-(3,4,5-trimethoxyphenyl)phthalazine

^{1}H NMR (400 MHz, $CDCl_3$): 8.30 (d, *J* = 7.8 Hz, 1H), 8.00 (d, *J* = 7.9 Hz, 1H), 7.89 – 7.61 (m, 2H), 6.91 (s, 2H), 6.00 (t, *J* = 6.5 Hz, 1H), 5.10 (s, 1H), 4.88 (s, 1H), 3.89 (s, 3H), 3.85 (s, 6H), 2.02 – 1.89 (m, 1H), 1.87 – 1.71 (m, 4H), 1.56 – 1.35 (m, 2H), 0.94 (t, *J* = 7.4 Hz, 3H) ppm.

^{13}C NMR (101 MHz, $CDCl_3$): δ 159.1, 155.9, 153.1, 143.6, 138.6, 131.9, 131.5, 127.6, 125.9, 123.1, 120.4, 112.4, 107.2, 79.2, 60.8, 56.1, 35.1, 18.7, 18.3, 13.9 ppm.

HRMS (ESI) m/z: $[M + H]^+$ Calcd for $C_{24}H_{29}N_2O_4$ 409.2122; Found 409.2122.

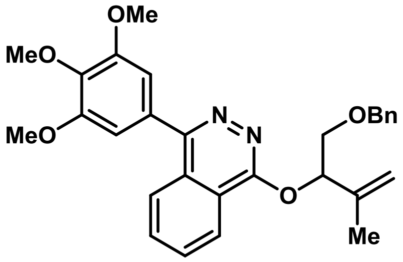

(±)-**23**

1-((1-(benzyloxy)-3-methylbut-3-en-2-yl)oxy)-4-(3,4,5 trimethoxyphenyl)phthalazine

^{1}H NMR (400 MHz, $CDCl_3$): δ 8.40 – 8.35 (m, 1H), 8.08 – 8.02 (m, 1H), 7.92 – 7.80 (m, 2H), 7.37 – 7.23 (m, 5H), 6.93 (s, 2H), 6.23 (dd, *J* = 6.7, 3.7 Hz, 1H), 5.22 (s, 1H), 5.01 (s, 1H), 4.67 (q, *J* = 12.3 Hz, 2H), 4.02 – 3.82 (m, 11H), 1.92 (s, 3H) ppm.

^{13}C NMR (151 MHz, $CDCl_3$): δ 159.0, 156.3, 153.2, 141.4, 138.7, 138.1, 132.1, 131.9, 131.6, 128.3, 127.7, 127.6, 127.5, 126.0, 123.3, 120.3, 113.2, 107.3, 77.9, 73.0, 70.9, 60.9, 56.2, 19.5 ppm.

HRMS (ESI) m/z: $[M + H]^+$ Calcd for $C_{29}H_{31}N_2O_5$ 487.2227; Found 487.2240.

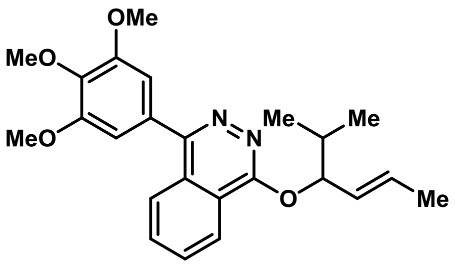

(±)-**24**

(*E*)-1-((2-methylhex-4-en-3-yl)oxy)-4-(3,4,5-trimethoxyphenyl)phthalazine

^{1}H NMR (400 MHz, CDCl$_3$): δ 8.39 – 8.29 (m, 1H), 8.06 (d, *J* = 7.5 Hz, 1H), 7.91 – 7.74 (m, 2H), 6.96 (s, 2H), 6.04 – 5.86 (m, 2H), 5.63 (ddd, *J* = 15.3, 7.6, 1.6 Hz, 1H), 3.95 (s, 3H), 3.91 (s, 6H), 2.21 (dq, *J* = 13.4, 6.7 Hz, 1H), 1.72 (dd, *J* = 6.5, 1.4 Hz, 3H), 1.13 – 0.98 (m, 6H) ppm.

^{13}C NMR (101 MHz, CDCl$_3$): δ 159.4, 155.8, 153.2, 140.0, 138.7, 132.1, 131.9, 131.5, 130.4, 127.7, 125.9, 123.3, 120.7, 107.3, 81.5, 61.0, 56.3, 32.3, 18.4, 18.1, 17.9 ppm.

HRMS (ESI) m/z: $[M + H]^+$ Calcd for $C_{24}H_{29}N_2O_4$ 409.2122; Found 409.2129.

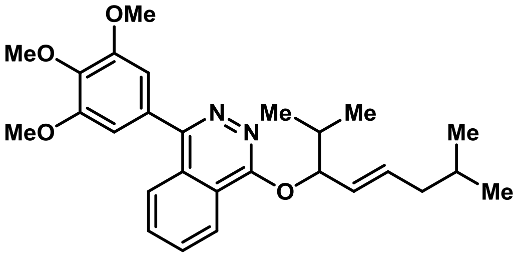

(±)-**25**

(*E*)-1-((2,7-dimethyloct-4-en-3-yl)oxy)-4-(3,4,5-trimethoxyphenyl)phthalazine

^{1}H NMR (600 MHz, CDCl$_3$): δ 8.32 – 8.31 (m, 1H), 8.03 – 8.02 (m, 1H), 7.85 – 7.77 (m, 2H), 6.93 (s, 2H), 5.92 – 5.87 (m, 2H), 5.58 – 5.54 (m, 1H), 3.91 (s, 3H), 3.87 (s, 6H), 2.22 – 2.18 (m, 1H), 1.95 – 1.87 (m, 2H), 1.62 – 1.58 (m, 1H), 1.05 (d, *J* = 6.0 Hz, 6H), 0.82 (t, *J* = 6.0 Hz, 6H) ppm.

^{13}C NMR (151 MHz, CDCl$_3$): δ 159.4, 155.7, 153.2, 138.7, 134.5, 132.1, 131.8, 131.4, 127.7, 127.5, 125.9, 123.33, 123.29, 120.7, 81.6, 60.9, 56.2, 41.8, 32.3, 28.1, 22.24, 22.22, 18.4, 18.0 ppm.

HRMS (ESI) m/z: $[M + H]^+$ Calcd for $C_{27}H_{35}N_2O_4$ 451.2591; Found 451.2599.

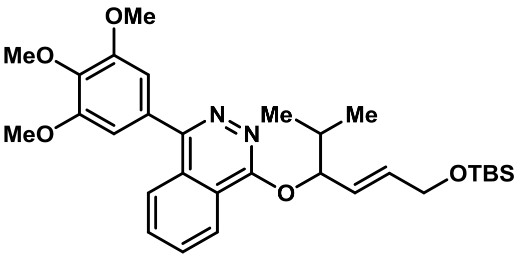

(±)-**26**

(*E*)-1-((6-((tert-butyldimethylsilyl)oxy)-2-methylhex-4-en-3-yl)oxy)-4-(3,4,5-trimethoxyphenyl)phthalazine

^{1}H NMR (400 MHz, CDCl$_3$): δ 8.35 – 8.33 (m, 1H), 8.06 – 8.04 (m, 1H), 7.89 – 7.80 (m, 2H), 6.94 (s, 2H), 6.04 – 5.98 (m, 2H), 5.89 – 5.83 (m, 1H), 4.19 (m, 2H), 3.94 (s, 3H), 3.90 (s, 6H), 2.32 – 2.18 (m, 1H), 1.08 (dd, *J* = 8.0 Hz, 6H), 0.87 (s, 9H), 0.03 (d, *J* = 8.0 Hz, 6H) ppm.

^{13}C NMR (101 MHz, CDCl$_3$): δ 159.4, 155.9, 153.3, 133.7, 132.1, 132.0, 131.5, 127.8, 126.1, 126.0, 123.4, 107.4, 80.8, 63.1, 61.0, 56.3, 32.4, 25.9, 18.4, 18.1, – 5.17, – 5.21 ppm.

HRMS (ESI) m/z: $[M + H]^+$ Calcd for $C_{30}H_{43}N_2O_5Si$ 539.2936; Found 539.2939.

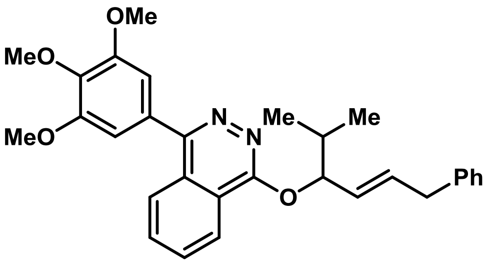

(±)-**27**

(*E*)-1-((2-methyl-6-phenylhex-4-en-3-yl)oxy)-4-(3,4,5-trimethoxyphenyl)phthalazine

^{1}H NMR (600 MHz, CDCl$_3$): δ 8.35 – 8.34 (m, 1H), 8.07 – 8.06 (m, 1H), 7.88 – 7.81 (m, 2H), 7.28 – 7.23 (m, 2H), 7.17 – 7.15 (m, 3H), 6.97 (s, 2H), 6.13 – 6.08 (m, 1H), 6.01 – 5.99 (m, 1H), 5.73 – 5.69 (m, 1H), 3.96 (s, 3H), 3.91 (s, 6H), 3.42 – 3.41 (m, 2H), 2.27 – 2.24 (m, 1H), 1.10 (dd, *J* = 12.0 Hz, 6H) ppm.

^{13}C NMR (151 MHz, CDCl$_3$): δ 159.5, 156.0, 153.4, 140.2, 138.9, 133.5, 132.2, 132.1, 131.7, 128.7, 128.5, 128.4, 127.9, 126.10, 126.09, 123.4, 120.8, 107.5, 81.3, 61.1, 56.4, 38.9, 32.5, 18.5, 18.3 ppm.

HRMS (ESI) m/z: $[M + H]^+$ Calcd for $C_{30}H_{33}N_2O_4$ 485.2435; Found 485.2438.

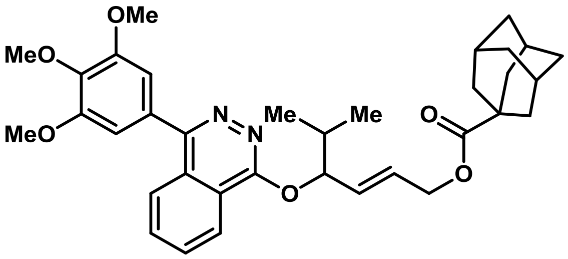

(±)-**28**

(*E*)-5-methyl-4-(4-(3,4,5-trimethoxyphenyl)phthalazin-1-yloxy)hex-2-enyl adamantanecarboxylate

^{1}H NMR (600 MHz, CDCl$_3$): δ 8.36 (d, *J* = 6.0 Hz, 1H), 8.07 (d, *J* = 12.0 Hz, 1H), 7.92 – 7.81 (m, 2H), 6.95 (s, 2H), 6.04 – 5.96 (m, 2H), 5.94 – 5.88 (m, 1H), 4.57 (d, *J* = 6.0 Hz, 1H), 3.95 (s, 3H), 3.90 (s, 6H), 2.30 – 2.21 (m, 1H), 1.98 (s, 3H), 1.86 (s, 6H), 1.74 – 1.64 (m, 6H), 1.11 (d, *J* = 6.0 Hz, 3H), 1.07 (d, *J* = 6.0 Hz, 1H) ppm.

^{13}C NMR (151 MHz, CDCl$_3$): δ 177.3, 159.3, 156.1, 153.2, 138.8, 132.0, 132.16, 131.6, 130.3, 128.1, 127.8, 126.1, 123.3, 120.6, 107.3, 80.3, 63.7, 61.0, 56.3, 40.7, 38.8, 36.5, 32.3, 27.9, 18.2, 18.1 ppm.

HRMS (ESI) m/z: [M + H]$^+$ Calcd for $C_{35}H_{43}N_2O_6$ 587.3116; Found 587.3123.

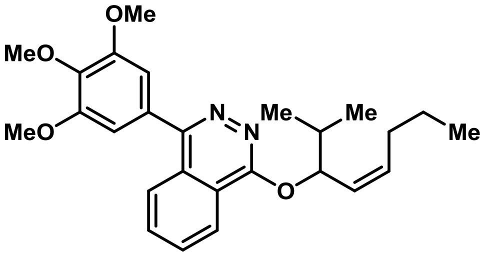

(±)-**29**

(*Z*)-1-((2-methyloct-4-en-3-yl)oxy)-4-(3,4,5-trimethoxyphenyl)phthalazine

^{1}H NMR (600 MHz, CDCl$_3$): δ 8.31 – 8.31 (m, 1H), 8.01 – 8.00 (m, 1H), 7.86 – 7.78 (m, 2H), 6.92 (s, 2H), 6.27 – 6.24 (m, 1H), 5.69 – 5.66 (m, 1H), 5.58 – 5.54 (m, 1H), 3.93 (s, 3H), 3.89 (s, 6H), 2.36 – 2.31 (m, 2H), 2.24 – 2.23 (m, 1H), 1.45 – 1.38 (m, 2H), 1.08 – 1.05 (m, 6H), 0.92 – 0.90 (m, 3H) ppm.

^{13}C NMR (151 MHz, CDCl$_3$): δ 159.5, 156.0, 153.2, 138.7, 135.3, 132.2, 131.9, 131.5, 127.8, 126.5, 126.0, 123.4, 120.6, 107.3, 77.0, 61.0, 56.3, 32.6, 30.3, 22.8, 18.4, 17.9, 13.9 ppm.

HRMS (ESI) m/z: [M + H]$^+$ Calcd for $C_{26}H_{33}N_2O_4$ 437.2435; Found 437.2438.

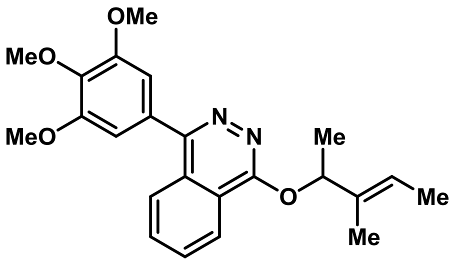

(±)-**30**

(*E*)-1-((3-methylpent-3-en-2-yl)oxy)-4-(3,4,5-trimethoxyphenyl)phthalazine

^{1}H NMR of (400 MHz, CDCl$_3$): δ 8.30 – 8.28 (m, 1H), 8.03 – 8.00 (m, 1H), 7.84 – 7.76 (m, 2H), 6.92 (s, 2H), 6.12 – 6.07 (m, 1H), 5.75 – 5.70 (m, 1H), 3.91 (s, 3H), 3.87 (s, 6H), 1.77 (s, 3H), 1.60 (d, *J* = 4.0 Hz, 3H), 1.56 (d, *J* = 8.0 Hz, 3H) ppm.

^{13}C NMR (101 MHz, CDCl$_3$): δ 159.1, 155.8, 153.3, 153.1, 138.6, 135.4, 132.0, 131.9, 131.4, 127.6, 125.9, 123.4, 121.5, 120.6, 107.3, 106.7, 77.6, 60.9, 56.2, 19.2, 13.1, 11.8 ppm.

HRMS (ESI) m/z: $[M + H]^+$ Calcd for $C_{23}H_{27}N_2O_4$ 395.1965; Found 395.1969.

2.2 Preparation of Cinchona Alkaloid Ligands.

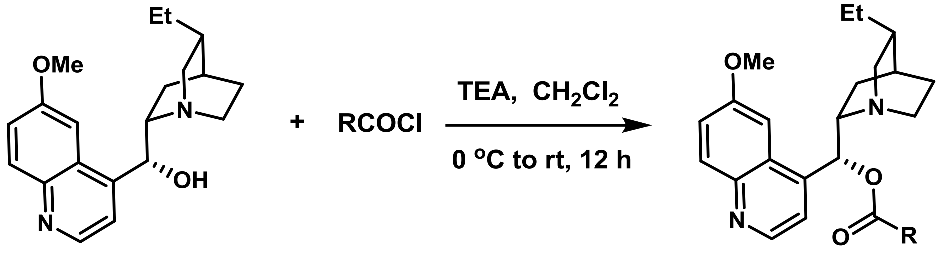

General Procedure II: Hydroquinine (1.96 g, 6.0 mmol, 1.2 equiv.) and triethylamine (1.39 mL, 10.0 mmol, 2.0 equiv.) were dissolved in anhydrous CH_2Cl_2 (30 mL) under N_2. The acyl chloride (5.0 mmol, 1.0 equiv.) was added dropwise to reaction mixture at 0 ℃. Then the reaction mixture was stirred at room temperature for 12 h. After the reaction was terminated by water, the reaction mixture was washed with 2N $NaHCO_3$. The organic layer was dried over anhydrous Na_2SO_4 and concentrated. The crude residue was purified by flash column chromatography on silica gel plate eluting with CH_2Cl_2/MeOH (20:1) to afford the corresponding products.

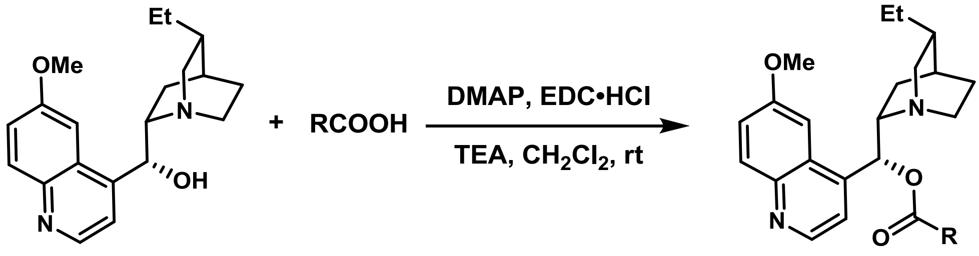

General Procedure III: A solution of the carboxylic acid (5 mmol, 1.5 equiv.) and 4-(dimethylamino) pyridine (DMAP) (30.5 mg, 0.25 mmol, 0.5 equiv.) in dried CH_2Cl_2 (20 mL) was treated with EDC•HCl (1.44 g, 7.5 mmol, 1.5 equiv.) and triethylamine (1.04 mL, 7.5 mmol, 1.5 equiv.) at room temperature under argon atmosphere. The resulting mixture was stirred at room temperature for 30 min and then was treated with the hydroquinine. After completion (monitored by TLC analysis), the reaction mixture was quenched with saturated $NaHCO_3$ (aq.) and extracted with CH_2Cl_2. The combined organic layer was washed with brine, dried over anhydrous Na_2SO_4, and concentrated in vacuo to give crude product, which was purified by flash chromatography with CH_2Cl_2/MeOH (20:1) to afford the corresponding products.

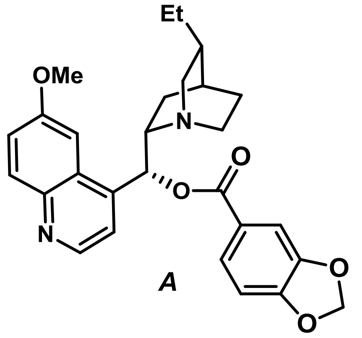

A

^{1}H NMR (600 MHz, $CDCl_3$): δ 8.71 (d, *J* = 4.5 Hz, 1H), 8.01 (d, *J* = 9.2 Hz, 1H), 7.71 (dd, *J* = 8.2, 1.7 Hz, 1H), 7.50 (m, 2H), 7.40 (d, *J* = 4.5 Hz, 1H), 7.37 (dd, *J* = 9.2, 2.7 Hz, 1H), 6.87 (d, *J* = 8.2 Hz, 1H), 6.70 (d, *J* = 6.4 Hz, 1H), 6.05 (s, 2H), 3.98 (s, 3H), 3.45 – 3.43 (m, 1H), 3.21 – 3.16 (m, 1H), 3.08 – 3.04 (m, 1H), 2.69 – 2.65 (m, 1H), 2.38 – 2.35 (m, 1H), 1.84 – 1.74 (m, 3H), 1.69 – 1.66 (m, 1H), 1.52 – 1.44 (m, 2H), 1.37 – 1.30 (m, 2H), 0.85 (t, *J* = 7.3 Hz, 3H) ppm.

^{13}C NMR (151 MHz, $CDCl_3$): δ 164.9, 158.0, 152.1, 148.0, 147.5, 144.8, 143.9, 131.8, 127.0, 125.6, 123.8, 122.0, 118.6, 109.5, 108.2, 102.0, 101.4, 74.5, 59.3, 58.5, 55.7, 42.8, 37.4, 28.6, 27.8, 25.4, 23.8, 12.1 ppm.

$[\alpha]_D^{25}$ = + 132.00 (*c* 1.00, $CHCl_3$).

HRMS (ESI) m/z: $[M + H]^+$ Calcd for $C_{28}H_{31}N_2O_5$ 475.2227; Found 475.2229.

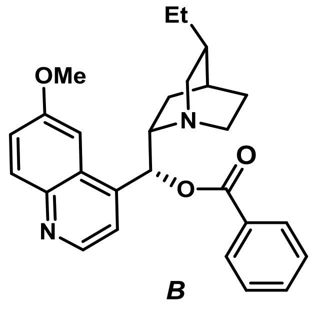

B

^{1}H NMR (400 MHz, $CDCl_3$): δ 8.71 (d, *J* = 4.5 Hz, 1H), 8.11 – 8.09 (m, 2H), 8.02 (d, *J* = 9.2 Hz, 1H), 7.62 – 7.57 (m, 1H), 7.53 (d, *J* = 2.6 Hz, 1H), 7.50 – 7.46 (m, 2H), 7.42 (d, *J* = 4.6 Hz, 1H), 7.38 (dd, *J* = 9.2, 2.7 Hz, 1H), 6.78 (d, *J* = 6.0 Hz, 1H), 3.99 (s, 3H), 3.50 – 3.44 (m, 1H), 3.26 – 3.19 (m, 1H), 3.12 – 3.06 (m, 1H), 2.74 – 2.66 (m, 1H), 2.41 – 2.38 (m, 1H), 1.87 – 1.71 (m, 4H), 1.55 – 1.48 (m, 2H), 1.38 – 1.29 (m, 2H), 0.85 (t, *J* = 7.3 Hz, 3H) ppm.

^{13}C NMR (101 MHz, $CDCl_3$): δ 165.5, 158.1, 147.4, 144.8, 143.7, 133.5, 131.9, 129.8, 129.7, 128.7, 126.9, 122.0, 118.5, 101.4, 74.5, 59.3, 58.4, 55.8, 42.8, 37.4, 28.5, 27.7, 25.4, 23.7, 12.1 ppm.

$[\alpha]_D^{25}$ = + 98.80 (*c* 1.00, $CHCl_3$).

HRMS (ESI) m/z: $[M + H]^+$ Calcd for $C_{27}H_{31}N_2O_3$ 431.2329; Found 431.2335.

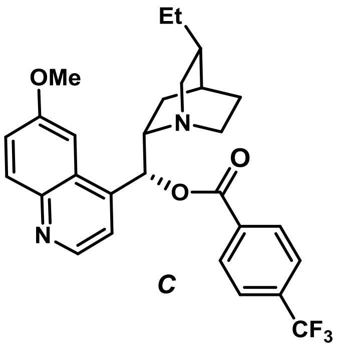

C

^{1}H NMR (600 MHz, CDCl$_3$): δ 8.73 (d, *J* = 4.5 Hz, 1H), 8.20 (d, *J* = 8.1 Hz, 2H), 8.03 (d, *J* = 9.2 Hz, 1H), 7.74 (d, *J* = 8.2 Hz, 2H), 7.50 (d, *J* = 2.5 Hz, 1H), 7.41 (d, *J* = 4.5 Hz, 2H), 6.75 (d, *J* = 6.7 Hz, 1H), 3.98 (s, 3H), 3.49 (q, *J* = 7.8 Hz, 1H), 3.19 – 3.14 (m, 1H), 3.06 (dd, *J* = 13.6, 9.9 Hz, 1H), 2.71 – 2.66 (m, 1H), 2.39 – 2.36 (m, 1H), 1.95 – 1.86 (m, 3H), 1.78 – 1.74 (m, 1H), 1.67 – 1.64 (m, 1H), 1.53 – 1.42 (m, 2H), 1.38 – 1.32 (m, 2H), 0.86 (t, *J* = 7.3 Hz, 3H) ppm.

^{13}C NMR (151 MHz, CDCl$_3$): δ 164.5, 158.1, 147.5, 144.9, 143.4, 135.0 (q, J_{CF} = 32.9 Hz), 133.1, 132.0, 130.1, 127.0, 125.7 (q, J_{CF} = 3.3 Hz), 123.5 (d, J_{CF} = 273.1 Hz), 121.9, 118.6, 101.4, 75.3, 59.3, 58.5, 55.7, 42.8, 37.4, 28.7, 27.8, 25.3, 24.1, 12.2 ppm.

^{19}F NMR (376 MHz, CDCl$_3$): δ -63.19 ppm.

$[\alpha]_D^{25}$ = + 94.50 (*c* 1.00, CHCl$_3$).

HRMS (ESI) m/z: $[M + H]^+$ Calcd for $C_{28}H_{30}F_3N_2O_3$ 499.2203; Found 499.2208.

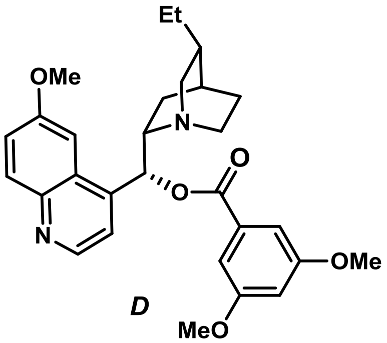

D

^{1}H NMR (600 MHz, CDCl$_3$): δ 8.70 (d, *J* = 4.5 Hz, 1H), 8.01 (d, *J* = 9.2 Hz, 1H), 7.53 (s, 1H), 7.39 – 7.37 (m, 2H), 7.24 (d, *J* = 2.3 Hz, 2H), 6.78 (s, 1H), 6.68 (t, *J* = 2.1 Hz, 1H), 4.00 (s, 3H), 3.82 (s, 6H), 3.44 (q, *J* = 8.2 Hz, 1H), 3.25 (s, 1H), 3.12 – 3.08 (m, 1H), 2.73 – 2.70 (m, 1H), 2.41 (d, *J* = 13.0 Hz, 1H), 1.87– 1.80 (m, 4H), 1.54 – 1.48 (m, 2H), 1.35 – 1.29 (m, 2H), 0.85 (t, *J* = 7.3 Hz, 3H) ppm.

^{13}C NMR (151 MHz, CDCl$_3$): δ 165.2, 160.9, 158.2, 147.4, 144.8, 131.9, 131.6, 126.8, 122.1, 118.4, 107.5, 105.6, 101.4, 59.2, 58.4, 55.9, 55.6, 42.9, 37.3, 27.7, 25.3, 12.1 ppm.

$[\alpha]_D^{25}$ = + 120.90 (*c* 1.00, CHCl$_3$).

HRMS (ESI) m/z: $[M + H]^+$ Calcd for $C_{29}H_{35}N_2O_5$ 491.2540; Found 491.2542.

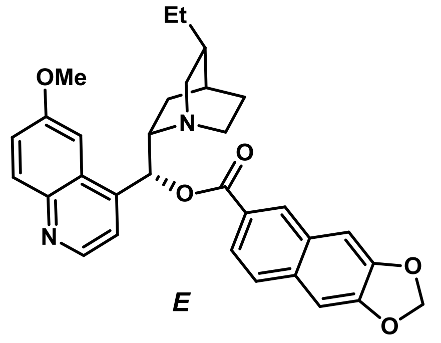

E

^{1}H NMR (600 MHz, CDCl$_3$): δ 8.77 (d, *J* = 4.5 Hz, 1H), 8.49 (s, 1H), 8.07 (d, *J* = 9.2 Hz, 1H), 8.01 (d, *J* = 8.5 Hz, 1H), 7.76 (d, *J* = 8.5 Hz, 1H), 7.61 (d, *J* = 2.1 Hz, 1H), 7.51 (d, *J* = 4.5 Hz, 1H), 7.43 (dd, *J* = 9.2, 2.2 Hz, 1H), 7.26 (s, 1H), 7.20 (s, 1H), 6.86 (d, *J* = 6.0 Hz, 1H), 6.13 (s, 2H), 4.05 (s, 3H), 3.54 (dd, *J* = 15.2, 7.7 Hz, 1H), 3.29 (t, *J* = 13.6 Hz, 1H), 3.13 (dd, *J* = 13.4, 10.1 Hz, 1H), 2.81 – 2.65 (m, 1H), 2.45 (d, *J* = 13.6 Hz, 1H), 2.09 – 1.76 (m, 7H), 1.63 – 1.46 (m, 2H), 1.46 – 1.33 (m, 2H), 0.91 (t, *J* = 7.3 Hz, 3H) ppm.

^{13}C NMR (151 MHz, CDCl$_3$): δ 165.9, 158.1, 149.9, 148.5, 147.5, 144.8, 143.9, 133.7, 131.8, 130.0, 129.7, 127.3, 127.0, 125.6, 124.0, 122.0, 118.6, 105.0, 1039, 101.6, 101.5, 74.5, 59.3, 58.5, 55.8, 42.8, 37.4, 28.6, 27.8, 25.4, 23.8, 12.1 ppm.

$[\alpha]_D^{25}$ = + 119.00 (*c* 1.00, CHCl$_3$).

HRMS (ESI) m/z: $[M + H]^+$ Calcd for $C_{32}H_{33}N_2O_5$ 525.2383; Found 525.2389.

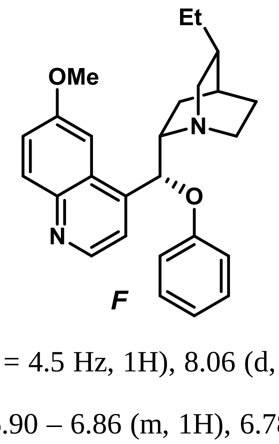

F

^{1}H NMR (400 MHz, CDCl$_3$): δ 8.67 (d, *J* = 4.5 Hz, 1H), 8.06 (d, *J* = 9.2 Hz, 1H), 7.43 – 7.40 (m, 2H), 7.37 (d, *J* = 2.5 Hz, 1H), 7.19 – 7.14 (m, 2H), 6.90 – 6.86 (m, 1H), 6.78 (d, *J* = 7.9 Hz, 2H), 6.00 (s, 1H), 3.99 (s, 3H), 3.47 – 3.39 (m, 1H), 3.27 – 3.10 (m, 2H), 2.75 – 2.68 (m, 1H), 2.44 – 2.39 (m, 1H), 2.04 – 1.84 (m, 3H),

1.54 – 1.47 (m, 3H), 1.29 – 1.22 (m, 2H), 0.82 (t, *J* = 7.4 Hz, 3H) ppm.

^{13}C NMR (101 MHz, $CDCl_3$): δ 158.2, 157.1, 147.7, 144.7, 143.8, 132.2, 129.6, 126.4, 121.9, 121.3, 118.6, 115.5, 100.9, 78.9, 59.0, 56.0, 43.6, 37.5, 28.3, 27.6, 25.7, 21.1, 12.1 ppm.

$[\alpha]_D^{25}$ = + 131.00 (*c* 1.00, $CHCl_3$).

HRMS (ESI) m/z: $[M + H]^+$ Calcd for $C_{26}H_{31}N_2O_2$ 403.2380; Found 403.2380.

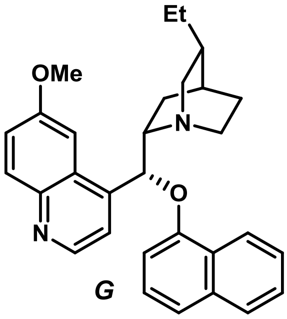

G

^{1}H NMR (400 MHz, $CDCl_3$): δ 8.62 – 8.54 (m, 2H), 8.07 (d, *J* = 9.2 Hz, 1H), 7.82 (d, *J* = 7.9 Hz, 1H), 7.62 – 7.52 (m, 2H), 7.48 – 7.40 (m, 3H), 7.35 (d, *J* = 8.3 Hz, 1H), 7.09 (t, *J* = 8.0 Hz, 1H), 6.43 (d, *J* = 7.7 Hz, 1H), 6.28 (s, 1H), 4.02 (s, 3H), 3.37 – 3.33 (m, 1H), 3.19 – 3.13 (m, 1H), 2.78 – 2.65 (m, 1H), 2.53– 2.45 (m, 1H), 2.30 – 2.20 (m, 1H), 2.11 – 1.88 (m, 2H), 1.76 – 1.63 (m, 1H), 1.62 – 1.48 (m, 2H), 1.36 – 1.21 (m, 2H), 0.84 (t, *J* = 7.3 Hz, 3H) ppm.

^{13}C NMR (101 MHz, $CDCl_3$): δ 158.3, 152.1, 147.6, 144.6, 143.5, 134.6, 132.1, 127.8, 126.5, 126.4, 125.82, 125.81, 125.5, 121.9, 121.5, 120.7, 118.0, 106.5, 100.7, 78.6, 60.2, 59.0, 56.0, 43.6, 37.5, 28.6, 27.8, 25.7, 21.4, 12.1 ppm.

$[\alpha]_D^{25}$ = + 232.30 (*c* 1.00, $CHCl_3$).

HRMS (ESI) m/z: $[M + H]^+$ Calcd for $C_{30}H_{33}N_2O_2$ 453.2537; Found 453.2539.

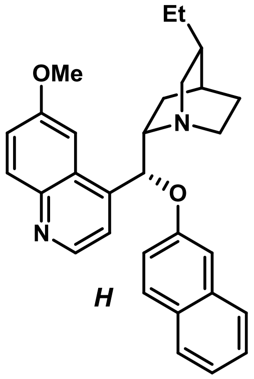

H

^{1}H NMR (400 MHz, $CDCl_3$): δ 8.66 (d, *J* = 4.5 Hz, 1H), 8.17 – 7.99 (m, 1H), 7.78 – 7.64 (m, 2H), 7.51 –

7.42 (m, 4H), 7.40 – 7.18 (m, 3H), 6.88 (d, *J* = 2.0 Hz, 1H), 6.11 (s, 1H), 4.02 (s, 3H), 3.52 – 3.35 (m, 2H), 3.27 (t, *J* = 8.4 Hz, 1H), 3.14 (dd, *J* = 13.4,10.0 Hz, 1H), 2.79 – 2.63 (m, 1H), 2.44 – 2.41 (m, 1H), 2.06 – 2.03 (m, 1H), 1.97 – 1.92 (m, 1H), 1.86 (d, *J* = 2.7 Hz, 1H), 1.63 – 1.38 (m, 3H), 1.35 – 1.16 (m, 2H), 0.83 (t, *J* = 7.3 Hz, 3H) ppm.

^{13}C NMR (101 MHz, $CDCl_3$): δ 158.2, 154.9, 147.7, 144.7, 143.8, 134.3, 132.2, 129.7, 129.1, 127.5, 126.8, 126.5, 126.4, 123.9, 121.9, 118.6, 118.5, 109.0, 101.0, 79.4, 60.1, 59.1, 55.9, 43.6, 37.5, 28.5, 27.6, 25.8, 21.2, 12.1 ppm.

$[\alpha]_D^{25}$ = + 197.40 (c 1.00, $CHCl_3$).

HRMS (ESI) m/z: $[M + H]^+$ Calcd for $C_{30}H_{33}N_2O_2$ 453.2537; Found 453.2538.

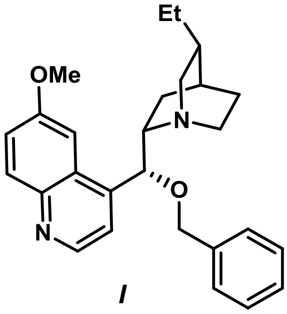

I

^{1}H NMR (600 MHz, $CDCl_3$): δ 8.77 (d, *J* = 4.5 Hz, 1H), 8.04 (d, *J* = 9.2 Hz, 1H), 7.71 (s, 1H), 7.55 (d, *J* = 4.4 Hz, 1H), 7.42 (dd, *J* = 9.2, 2.5 Hz, 1H), 7.39 – 7.30 (m, 5H), 6.42 (s, 1H), 4.67 (d, *J* = 11.1 Hz, 1H), 4.52 (d, *J* = 11.1 Hz, 1H), 4.15 (s, 3H), 4.03 (s, 1H), 3.38 (dt, *J* = 17.8, 10.4 Hz, 2H), 3.06 (s, 1H), 2.73 (d, *J* = 10.5 Hz, 1H), 2.22 (s, 1H), 2.02 (d, *J* = 6.5 Hz, 2H), 1.90 – 1.82 (m, 1H), 1.76 – 1.70 (m, 1H), 1.51 (t, *J* = 11.9 Hz, 1H), 1.30 – 1.22 (m, 3H), 0.82 (t, *J* = 7.4 Hz, 3H) ppm.

^{13}C NMR (151 MHz, $CDCl_3$): δ 159.2, 147.0, 144.8, 137.0, 131.8, 128.7, 128.2, 127.7, 127.0, 123.3, 118.3, 101.0, 71.6, 59.7, 57.7, 56.9, 35.6, 29.7, 27.1, 25.3, 24.8, 11.5 ppm.

$[\alpha]_D^{25}$ = – 13.20 (*c* 1.00, $CHCl_3$).

HRMS (ESI) m/z: $[M + H]^+$ Calcd for $C_{27}H_{33}N_2O_2$ 417.2536; Found 417.2521.

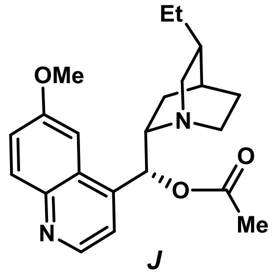

J

^{1}H NMR (600 MHz, $CDCl_3$): δ 8.74 – 8.72 (m, 1H), 8.01 – 7.99 (m, 1H), 7.44 – 7.43 (m, 1H), 7.37 – 7.33 (m, 2H), 6.49 – 6.47 (m, 1H), 3.95 – 3.93 (m, 3H), 3.36 – 3.33 (m, 1H), 3.10 – 2.98 (m, 2H), 2.65 – 2.61 (m, 1H), 2.32 – 2.30 (m, 1H), 2.12 – 2.10 (m, 3H), 1.80 – 1.70 (m, 3H), 1.54 – 1.26 (m, 5H), 0.89 – 0.81 (m, 3H) ppm.

^{13}C NMR (151 MHz, $CDCl_3$): δ 170.1, 157.9, 147.5, 144.8, 143.7, 131.8, 127.1, 121.8, 118.9, 101.5, 73.9, 58.9, 58.4, 55.7, 42.6, 37.4, 28.5, 27.7, 25.3, 24.1, 21.1, 12.2 ppm.

$[\alpha]_D^{25}$ = + 3.20 (*c* 1.00, $CHCl_3$).

HRMS (ESI) m/z: $[M + H]^+$ Calcd for $C_{22}H_{29}N_2O_3$ 369.2173; Found 369.2176.

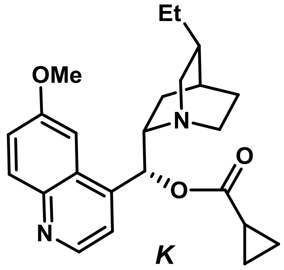

K

^{1}H NMR (600 MHz, $CDCl_3$): δ 8.72 (s, 1H), 7.98 (d, *J* = 8.3 Hz, 1H), 7.41 – 7.36 (m, 3H), 6. 49 – 6.49 (m, 1H), 3.92 (s, 3H), 3.34 – 3.31 (m, 1H), 3.09 – 2.98 (m, 2H), 2.63 - 2.59 (m, 1H), 2.30 (d, *J* = 11.7 Hz, 1H), 1.79 (s, 2H), 1.69 (d, *J* = 16.9 Hz, 2H), 1.50 – 1.2 (m, 5H), 1.00 – 0.83 (m, 7H) ppm.

^{13}C NMR (151 MHz, $CDCl_3$): δ 173.9, 157.8, 147.4, 144.8, 143.8, 131.7, 127.1, 121.7, 118.8, 101.4, 73.6, 59.0, 58.3, 55.6, 42.5, 37.4, 28.4, 27.7, 25.2, 24.0, 13.0, 12.1, 8.6, 8.5 ppm.

$[\alpha]_D^{25}$ = + 0.30 (*c* 1.00, $CHCl_3$).

HRMS (ESI) m/z: $[M + H]^+$ Calcd for $C_{24}H_{31}N_2O_3$ 395.2329; Found 395.2330.

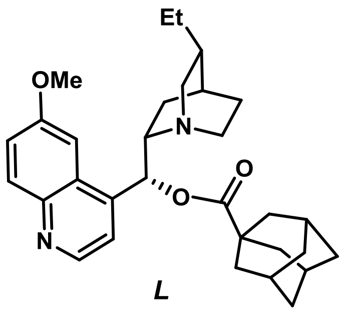

L

^{1}H NMR (400 MHz, CDCl3): δ 8.69 (d, *J* = 4.6 Hz, 1H), 7.99 (d, *J* = 9.2 Hz, 1H), 7.53 (s, 1H), 7.36 (dd, *J* = 9.2, 2.6 Hz, 1H), 7.26 (d, *J* = 3.8 Hz, 1H), 6.70 (s, 1H), 4.02 (s, 3H), 3.42 (s, 1H), 3.36 – 3.29 (m, 2H), 3.22 – 3.11 (m, 1H), 2.83 (t, *J* = 9.3 Hz, 1H), 2.45 (d, *J* = 12.7 Hz, 1H), 2.05 – 2.02 (m, 3H), 1.97 – 1.90 (m, 7H), 1.85 (d, *J* = 2.8 Hz, 1H), 1.81 – 1.68 (m, 8H), 1.32 – 1.24 (m, 2H), 0.82 (t, *J* = 7.3 Hz, 3H) ppm.

^{13}C NMR (101 MHz, $CDCl_3$): δ 175.9, 158.4, 146.0, 144.6, 131.5, 126.6, 122.5, 101.2, 58.8, 57.6, 56.3, 50.4, 42.6, 40.8, 39.1, 38.7, 36.7, 36.6, 36.3, 28.11, 27.8, 27.5, 25.1, 11.8 ppm.

$[\alpha]_D^{25}$ = + 14.50 (*c* 1.00, $CHCl_3$).

HRMS (ESI) m/z: $[M + H]^+$ Calcd for $C_{31}H_{41}N_2O_3$ 489.3112; Found 489.3116.

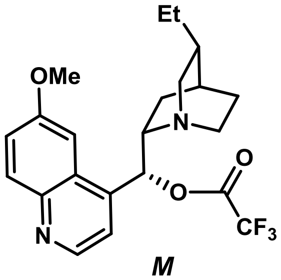

M

^{1}H NMR (600 MHz, $CDCl_3$): δ 8.79 (s, 1H), 8.10 – 8.06 (m, 2H), 7.36 – 7.28 (m, 2H), 6.38 (s, 1H), 4.29 (s, 1H), 3.83 (s, 3H), 3.52 – 3.47 (m, 1H), 3.25 (t, *J* = 8.7 Hz, 1H), 3.12 (s, 1H), 2.69 (d, *J* = 11.3 Hz, 1H), 2.20 – 2.05 (m, 3H), 1.83 (d, *J* = 59.2 Hz, 2H), 1.31 – 1.21 (m, 3H), 0.78 (t, *J* = 7.4 Hz, 3H) ppm.

^{13}C NMR of M (151 MHz, $CDCl_3$): δ 162.4 (q, J_{CF} = 30.2 Hz), 160.4, 154.7, 140.3, 134.3, 126.9, 126.7, 124.2, 119.5, 116.46 (d, J_{CF} = 291.4 Hz), 100.5, 66.4, 59.9, 57.0, 56.5, 43.9, 35.2, 26.7, 24.5, 24.2, 17.6, 11.3 ppm.

^{19}F NMR (**376** MHz, $CDCl_3$): δ -75.6 ppm.

$[\alpha]_D^{25}$ = – 44.00 (*c* 1.00, $CHCl_3$).

HRMS (ESI) m/z: $[M + H]^+$ Calcd for $C_{22}H_{26}F_3N_2O_3$ 423.1890; Found 423.1899.

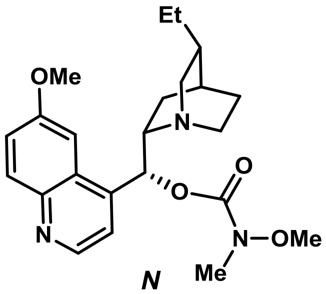

N

^{1}H NMR (600 MHz, $CDCl_3$): δ 8.73 (d, J = 4.5 Hz, 1H), 8.00 (d, J = 9.2 Hz, 1H), 7.46 (d, J = 2.5 Hz, 1H), 7.35 (dd, J = 10.4, 3.4 Hz, 2H), 6.45 (d, J = 6.4 Hz, 1H), 3.95 (s, 3H), 3.71 (s, 3H), 3.35 (q, J = 7.7 Hz, 1H), 3.18 – 3.15 (m, 4H), 3.02 (dd, J= 13.5, 9.9 Hz, 1H), 2.67 – 2.62 (m, 1H), 2.32 (d, J = 13.6 Hz, 1H), 1.78 – 1.72 (m, 3H), 1.64 – 1.56 (m, 1H), 1.49 – 1.42 (m, 2H), 1.38 – 1.26 (m, 2H), 0.82 (t, J = 7.4 Hz, 3H) ppm.

^{13}C NMR (151 MHz, $CDCl_3$): δ 156.0, 156.3, 147.4, 144.8, 143.8, 131.7, 126.9, 121.9, 118.7, 101.5, 75.7, 61.9, 59.2, 58.3, 55.7, 42.6, 35.5, 28.4, 27.7, 25.3, 23.8, 12.1 ppm.

$[\alpha]_D^{25}$ = + 21.40 (c 1.00, $CHCl_3$).

HRMS (ESI) m/z: $[M + H]^+$ Calcd for $C_{23}H_{32}N_3O_4$ 414.2387; Found 414.2390.

2.3 General Procedure for AD-Based Kinetic Resolution.

<u>General Procedure IV:</u> To a 20 mL glass tube containing a magnetic stir was charged $K_3Fe(CN)_6$ (98.7 mg, 3.0 equiv.), K_2CO_3 (41.2 mg, 3.0 equiv.), and racemic allylic ether (0.1 mmol, 1.0 equiv.). To a 50 mL round bottom flask, stock solution of ($K_2OsO_2(OH)_4$ (0.4 mol% x 20) and ligand (1.0 mol% x 20) was prepared with 20 mL of ^{t}BuOH-H_2O (v/v = 1:1). The stock solution (1.0 mL) was added to the glass tube. The reaction mixture was cooled to 0 ^oC for 3 - 60 h, which was quenched with saturated $Na_2S_2O_3$ (2.0 mL) at room temperature and extracted with ethyl acetate (2.0 mL x 3). The combined organic layer was dried over Na_2SO_4 and concentrated in vacuo. The residue was purified by column chromatography (petroleum ether/ethyl acetate = 3 : 1 to 1 : 2) on silica gel to give the allylic ether and diol product.

The selectivity factor (*s*) was calculated through the equation: *s* = In[(1-c)(1-ee)]/In[(1-c)(1+ee)], where the conversion [c = **diol** $(H_b)^{Integrals}$/(**substrate** $(H_a)^{Integrals}$ + **diol** $(H_b)^{Integrals}$)% or **diol** $(H_b)^{Integrals}$/(**substrate** $(H_c)^{Integrals}$ + **diol** $(H_b)^{Integrals}$)%)] of the reaction and diastereomeric ratio of the diol product were determined by ^{1}H NMR analysis, and enantiomeric excess (ee) was determined by chiral HPLC analysis.

2.4 Screening an appropriate π in the racemic allylic ethers for AD-based kinetic resolution.

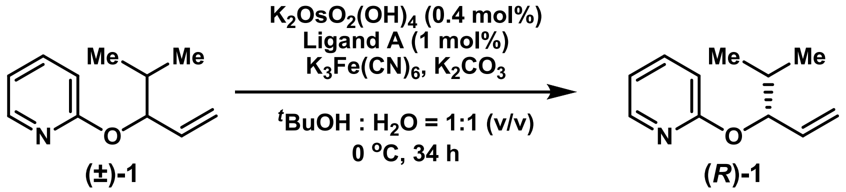

The general procedure **IV** was followed. The conversion of **(±)-1** was determined by crude ^{1}H NMR (56% conversion, 44% yield, 33% ee).

Conversion (%) = [1.27 / (1.00 + 1.27)] % = 56%

S = In [(1 – conv)(1 – ee)] / In [(1 – conv)(1 + ee)] = 2

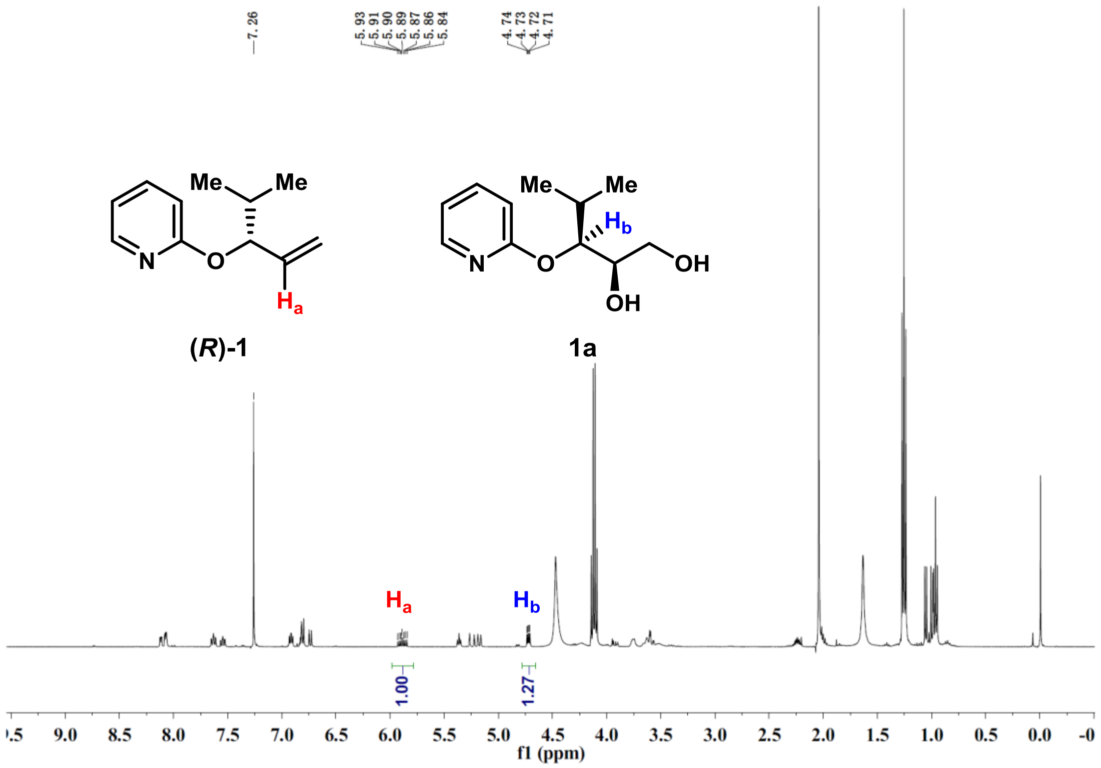

Supplementary Figure 1. ^{1}H NMR spectrum of crude mixture of compound **(*R*)-1** and **1a**.

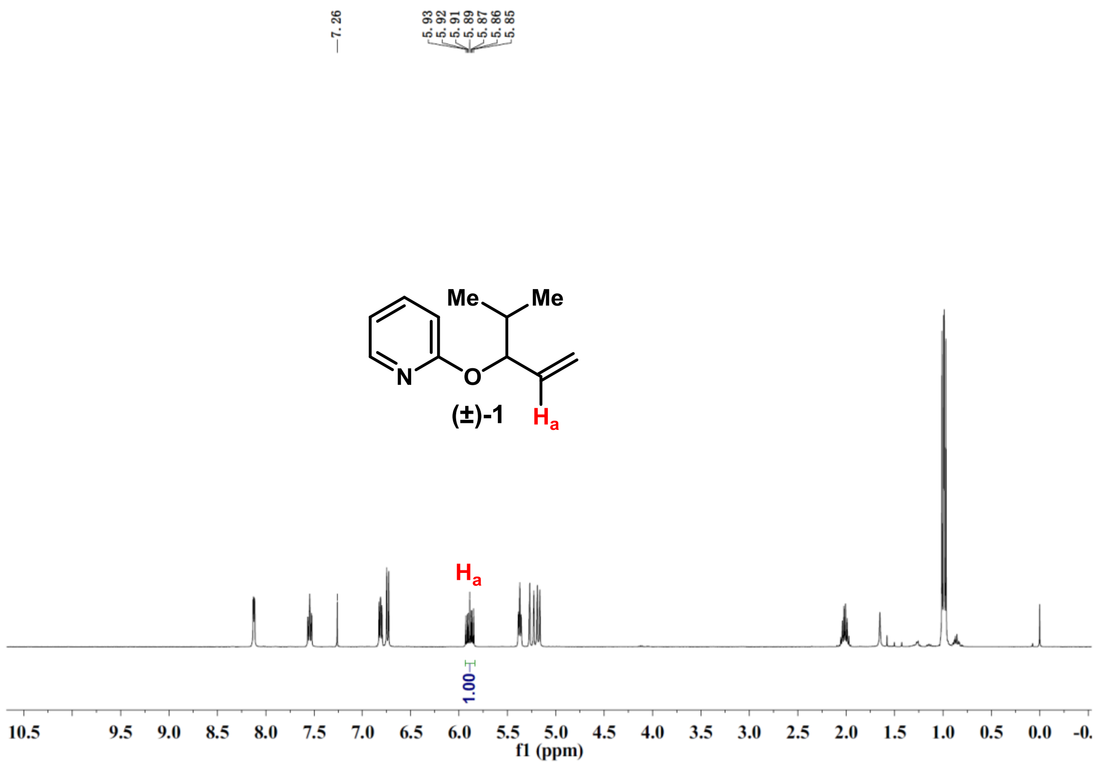

Supplementary Figure 2. ^{1}H NMR spectrum of starting material **(±)-1**.

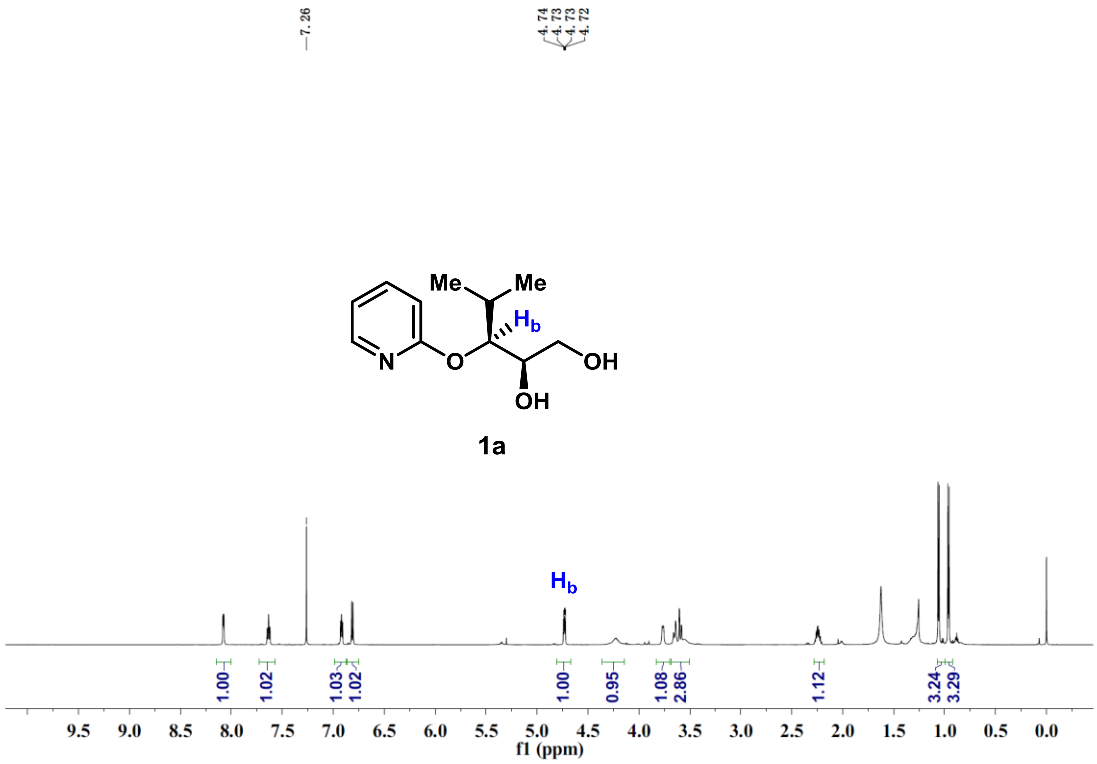

Supplementary Figure 3. ^{1}H NMR spectrum of product **1a**.

HPLC (OD-H, 0.46*25 cm, 5μm, isopropanol/hexane = 0.1%, flow = 1.0 mL/min, detection at 210 nm), retention time = 4.797 min (minor) and 5.118 min (major).

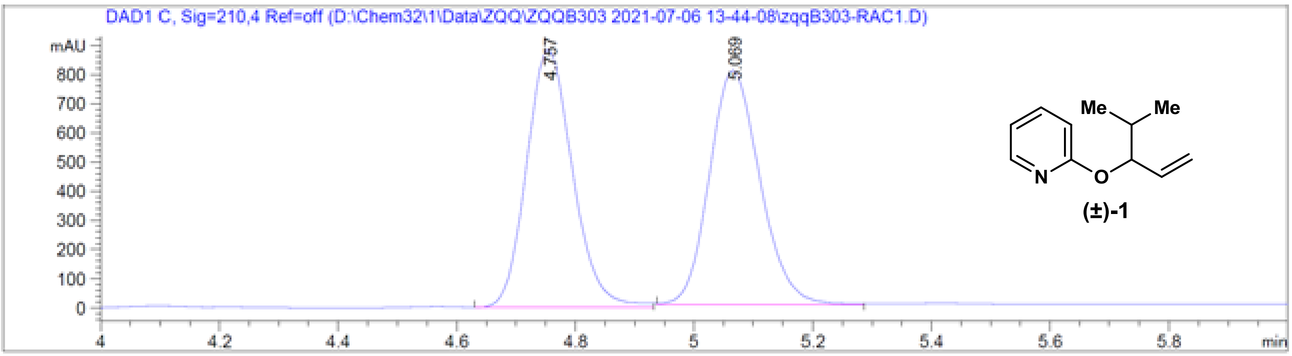

Signal 2: DAD1 C, Sig=210,4 Ref=off

Peak #	RetTime [min]	Type	Width [min]	Area [mAU*s]	Height [mAU]	Area %
1	4.757	BV	0.0848	4762.57617	884.27991	49.9691
2	5.069	MM R	0.0989	4768.45752	803.93396	50.0309

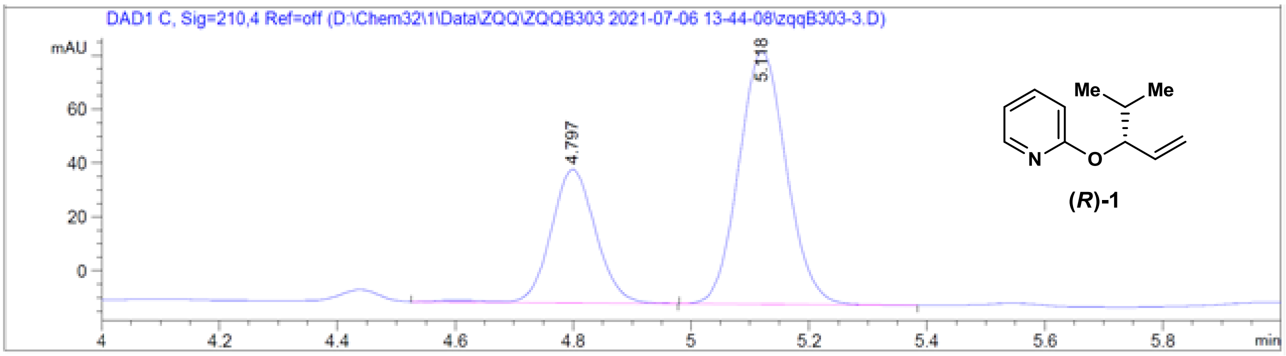

Signal 2: DAD1 C, Sig=210,4 Ref=off

Peak #	RetTime [min]	Type	Width [min]	Area [mAU*s]	Height [mAU]	Area %
1	4.797	VB R	0.0814	261.33008	49.64353	33.2246
2	5.118	BB	0.0871	525.22607	94.08320	66.7754

Supplementary Figure 4. HPLC chromatogram for **(*R*)-1**.

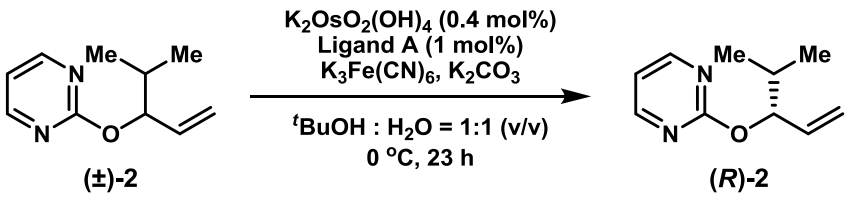

The general procedure **IV** was followed. The conversion of **(±)-2** was determined by crude ^{1}H NMR (55% conversion, 45% yield, 26% ee).

Conversion (%) = [1.20 / (1.00 + 1.20)] % = 55%

S = In [(1 – conv)(1 – ee)] / In [(1 – conv)(1 + ee)] = 2

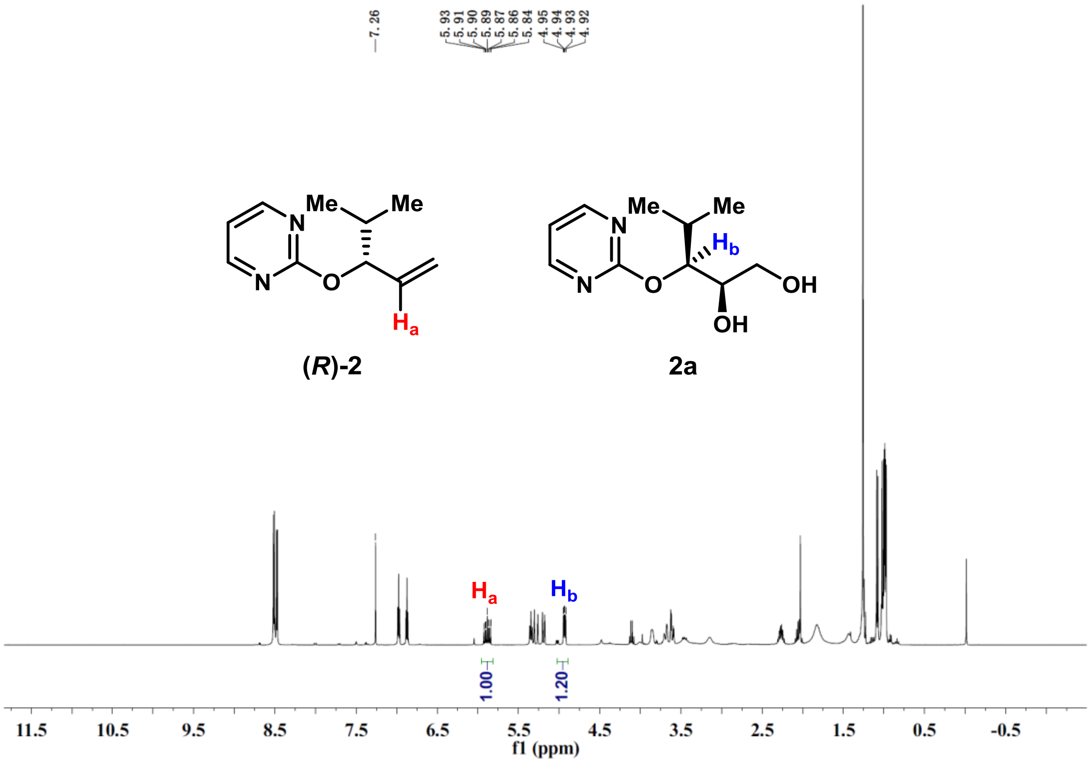

Supplementary Figure 5. ^{1}H NMR spectrum of crude mixture of compound **(*R*)-2** and **2a**.

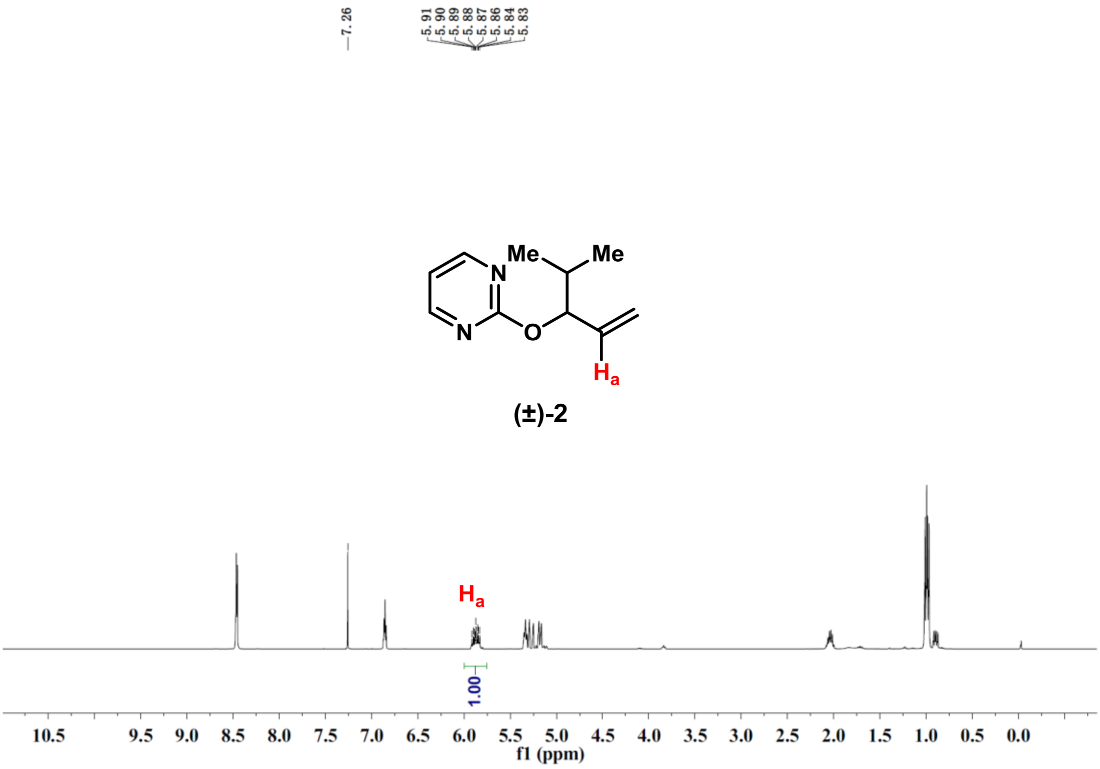

Supplementary Figure 6. ^{1}H NMR spectrum of starting material **(±)-2**.

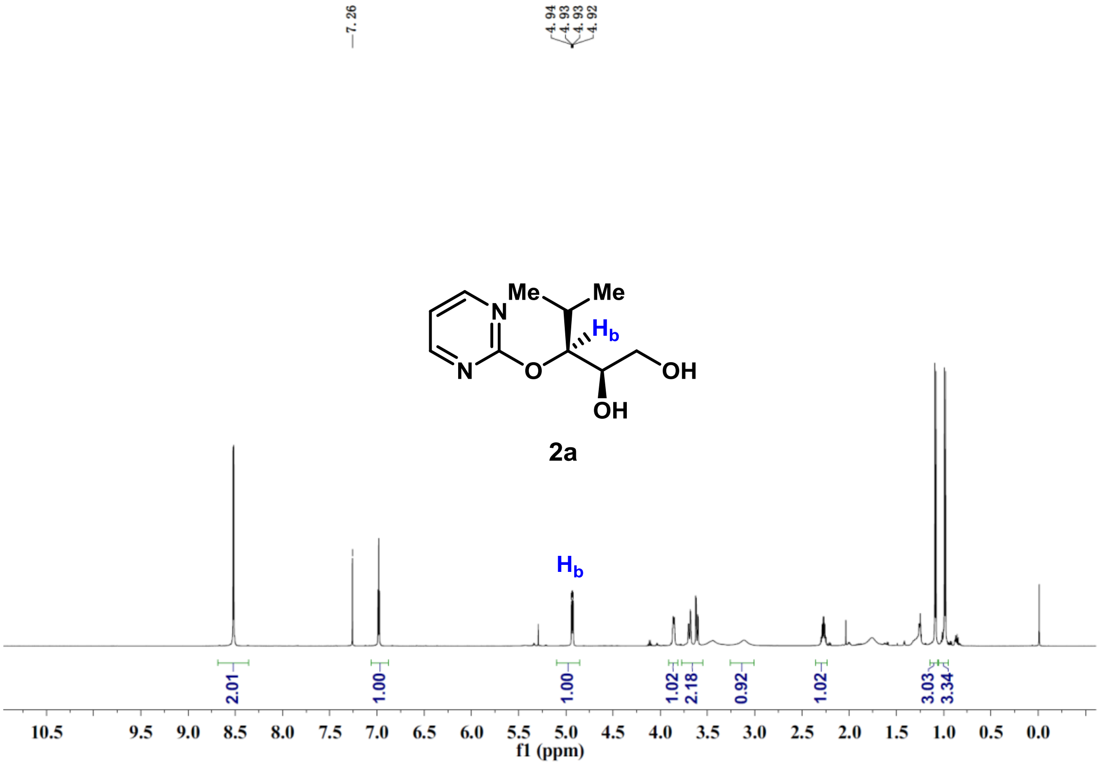

Supplementary Figure 7. ^{1}H NMR spectrum of dihydroxylated product **2a**.

HPLC (IA-H, 0.46*25 cm, 5μm, hexane/isopropanol = 99/1, flow = 1.0 mL/min, detection at 210 nm), retention time = 6.593 min (minor) and 6.932 min (major).

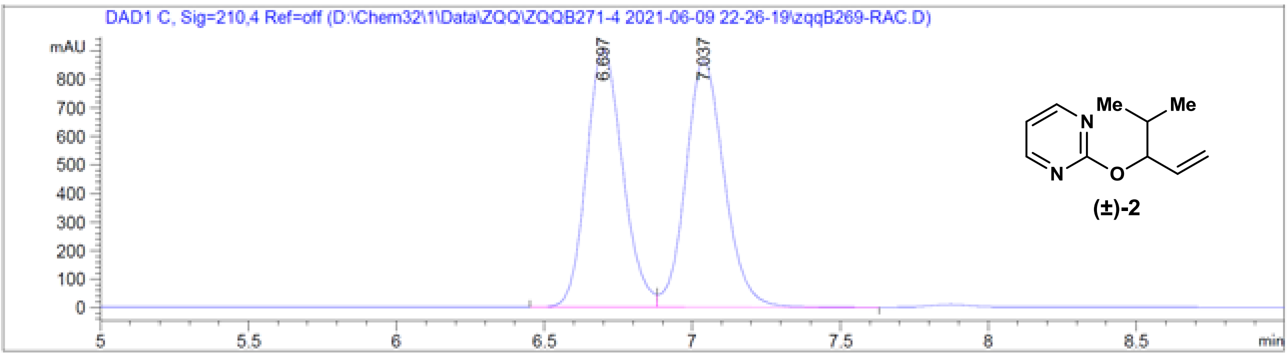

Signal 2: DAD1 C, Sig=210,4 Ref=off

Peak #	RetTime [min]	Type	Width [min]	Area [mAU*s]	Height [mAU]	Area %
1	6.697	BV	0.1334	7691.30078	903.05518	49.4933
2	7.037	VB	0.1397	7848.78516	866.64679	50.5067

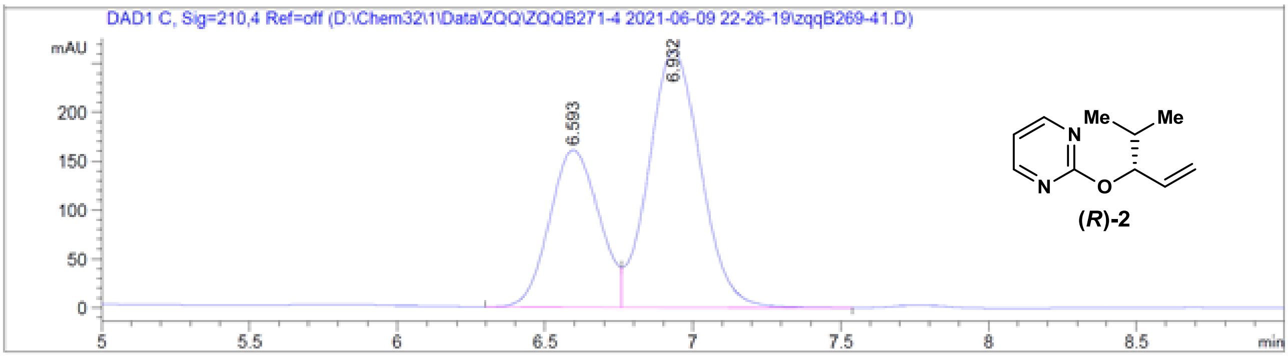

Signal 2: DAD1 C, Sig=210,4 Ref=off

Peak #	RetTime [min]	Type	Width [min]	Area [mAU*s]	Height [mAU]	Area %
1	6.593	BV	0.1847	1878.12769	160.28806	36.8843
2	6.932	VB	0.1912	3213.81055	261.90347	63.1157

Supplementary Figure 8. HPLC chromatogram for ***(R)*-2**.

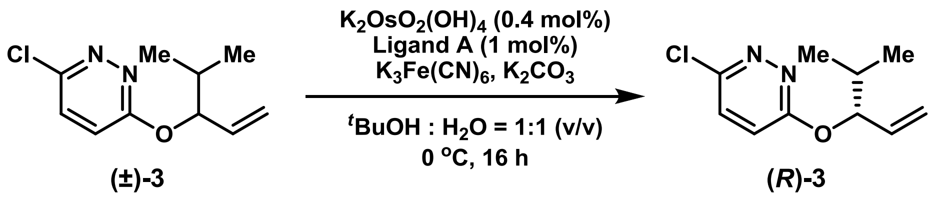

The general procedure **IV** was followed. The conversion of **(±)-3** was determined by crude ^{1}H NMR (46% conversion, 54% yield, 32% ee).

Conversion (%) = [0.84 / (1.00 + 0.84)] % = 46%

S = In [(1 – conv)(1 – ee)] / In [(1 – conv)(1 + ee)] = 2

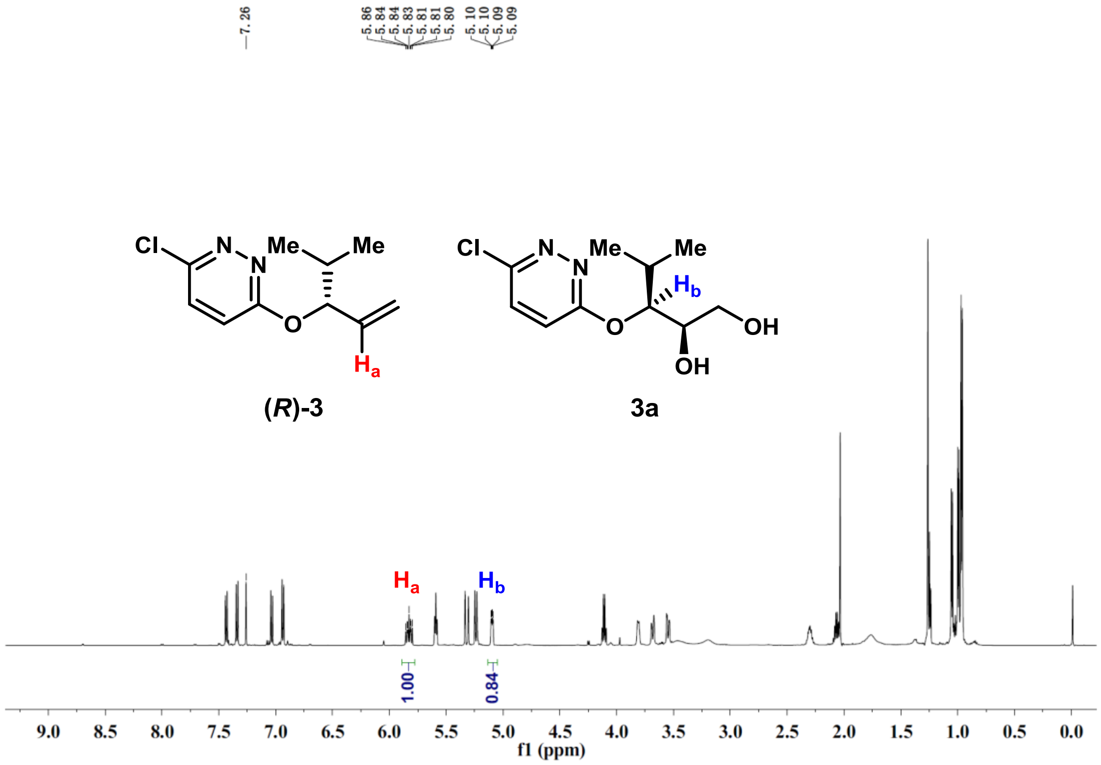

Supplementary Figure 9. ^{1}H NMR spectrum of crude mixture of compound **(*R*)-3** and **3a**.

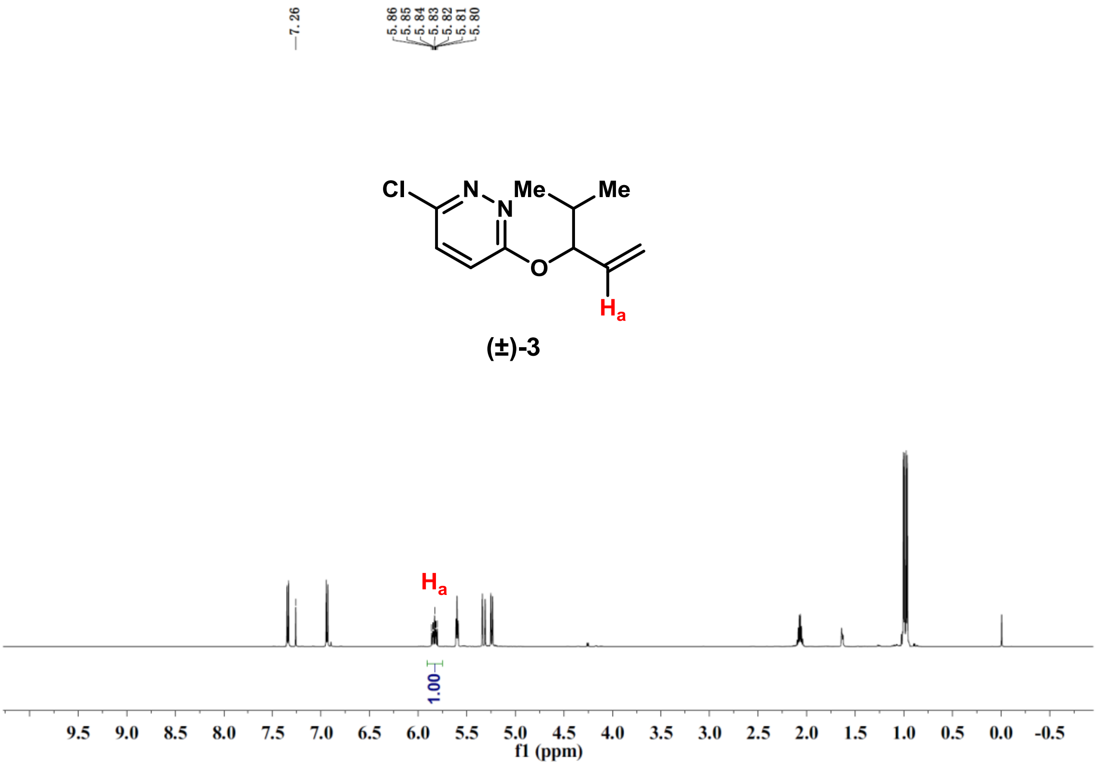

Supplementary Figure 10. ^{1}H NMR spectrum of starting material **(±)-3**.

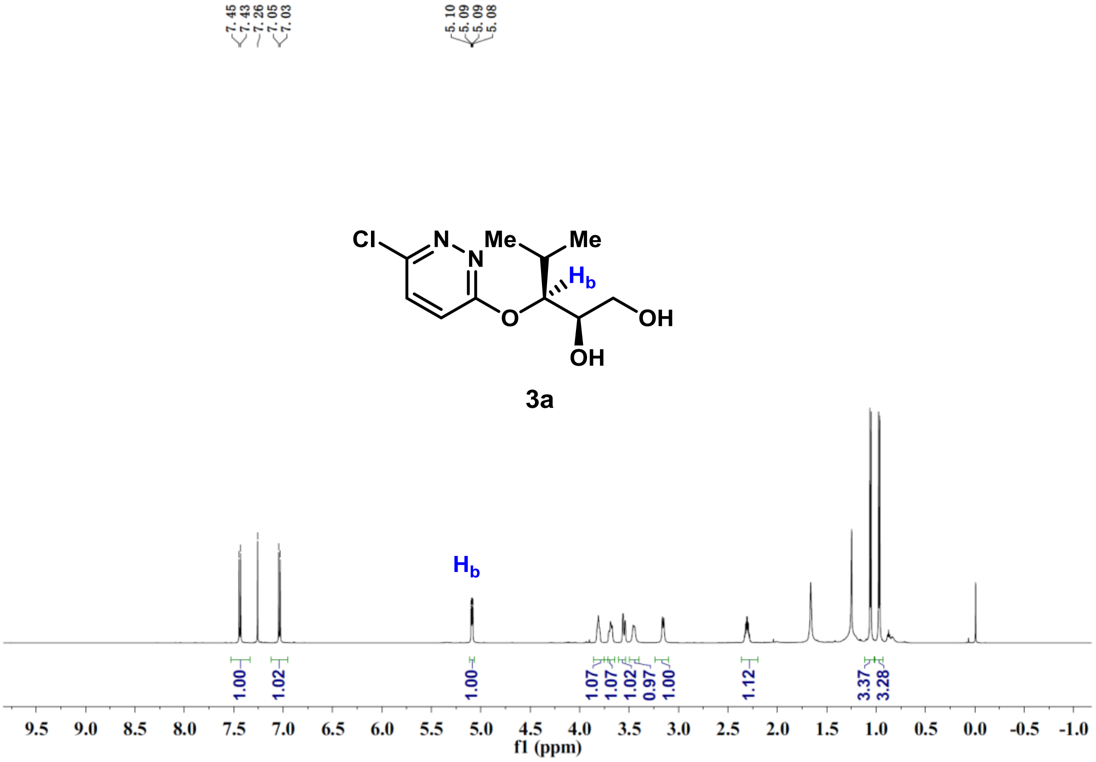

Supplementary Figure 11. ^{1}H NMR spectrum of dihydroxylated product **3a**.

HPLC (OD-H, 0.46*25 cm, 5μm, hexane/isopropanol = 95/5, flow = 1.0 mL/min, detection at 210 nm), retention time = 4.992 min (minor) and 5.351 min (major).

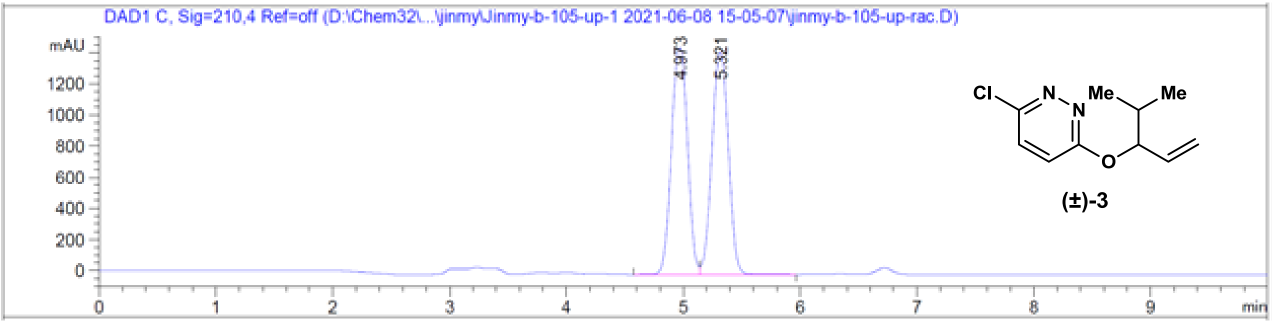

Signal 3: DAD1 C, Sig=210,4 Ref=off

Peak #	RetTime [min]	Type	Width [min]	Area [mAU*s]	Height [mAU]	Area %
1	4.973	BV	0.1531	1.39184e4	1461.04492	50.0033
2	5.321	VB	0.1554	1.39166e4	1431.83972	49.9967

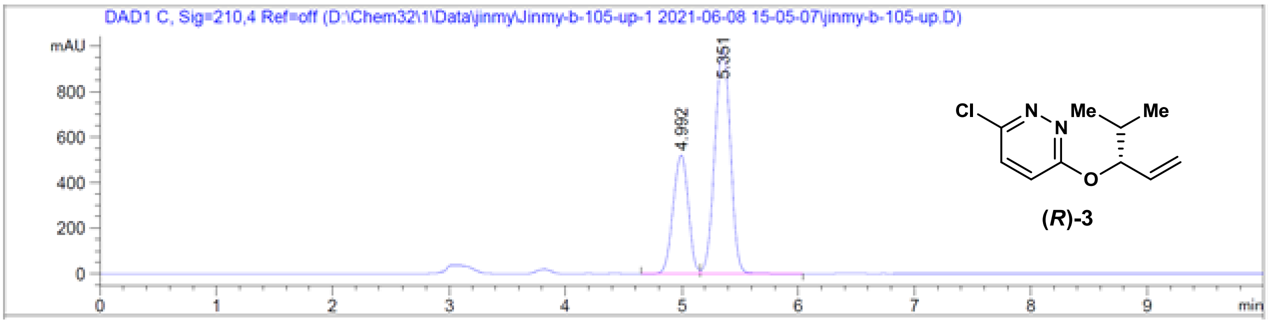

Signal 3: DAD1 C, Sig=210,4 Ref=off

Peak #	RetTime [min]	Type	Width [min]	Area [mAU*s]	Height [mAU]	Area %
1	4.992	BV	0.1461	4742.46143	521.29865	33.8453
2	5.351	VB	0.1469	9269.72852	993.31122	66.1547

Supplementary Figure 12. HPLC chromatogram for ***(R)*-3**.

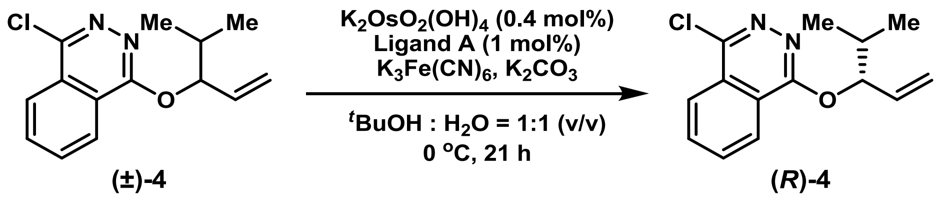

The general procedure **IV** was followed. The conversion of **(±)-4** was determined by crude ^{1}H NMR (47% conversion, 53% yield, 40% ee).

Conversion (%) = [(1.88 – 1.00) / 1.88] % = 47%

S = In [(1 – conv)(1 – ee)] / In [(1 – conv)(1 + ee)] = 4

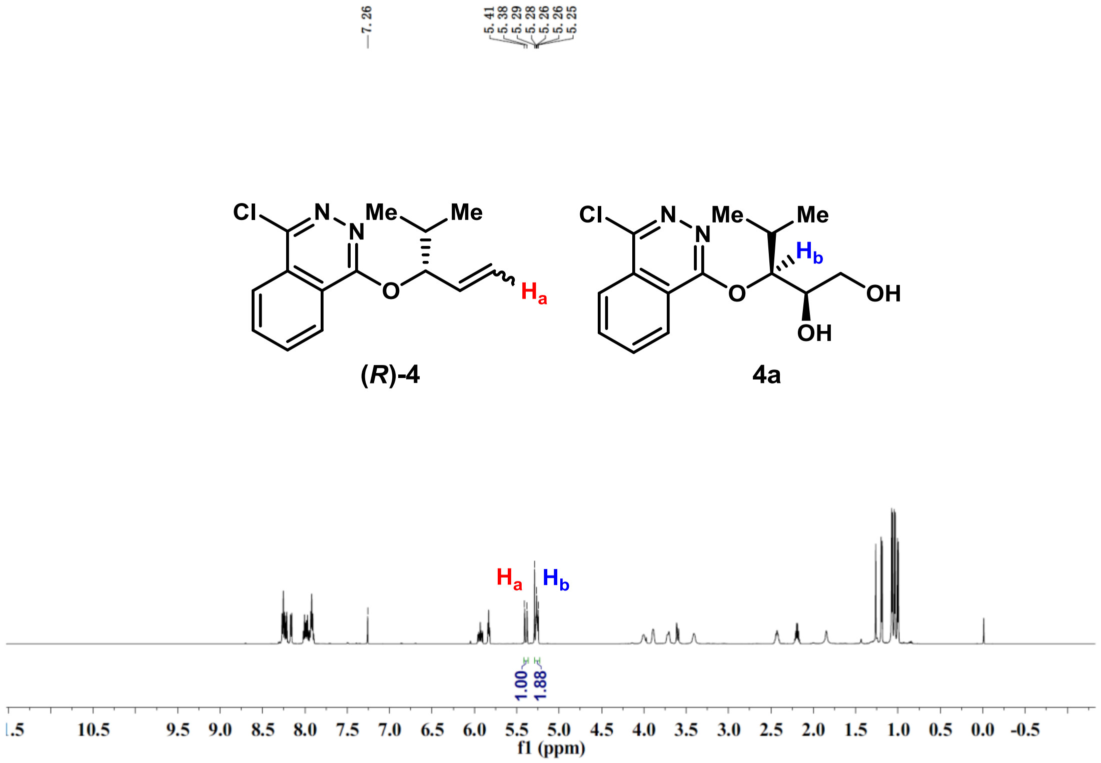

Supplementary Figure 13. ^{1}H NMR spectrum of crude mixture of compound **(*R*)-4** and **4a**

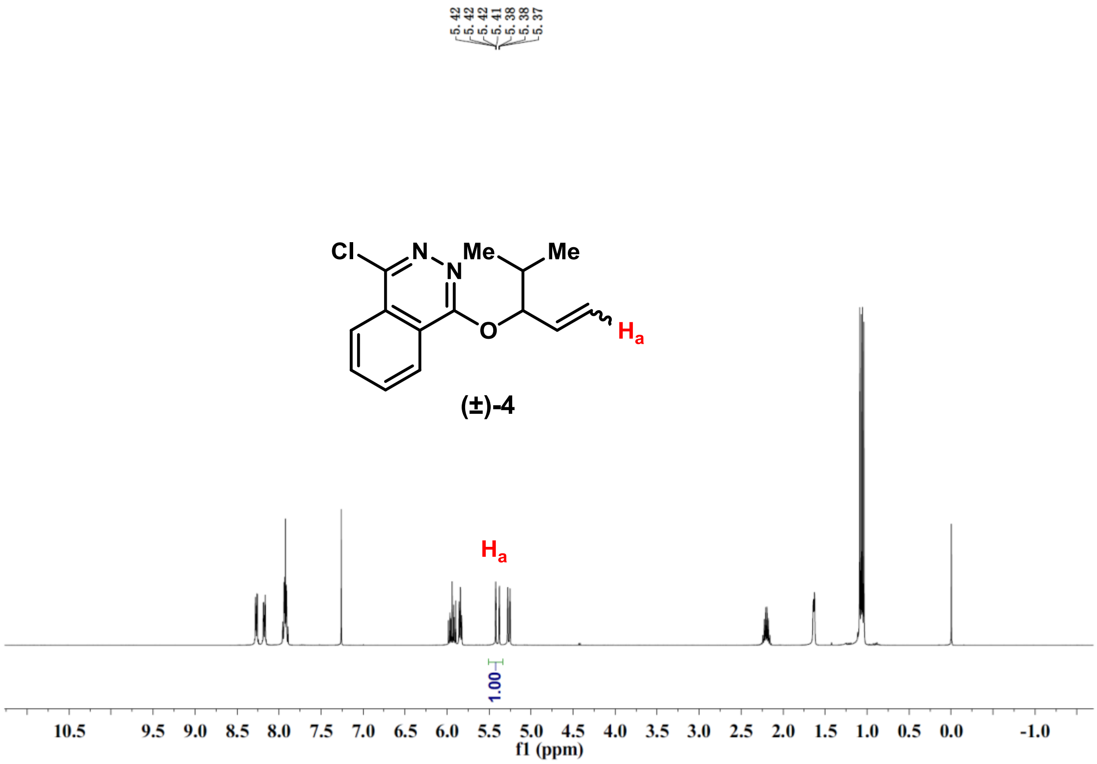

Supplementary Figure 14. ^{1}H NMR spectrum of starting material **(±)-4**.

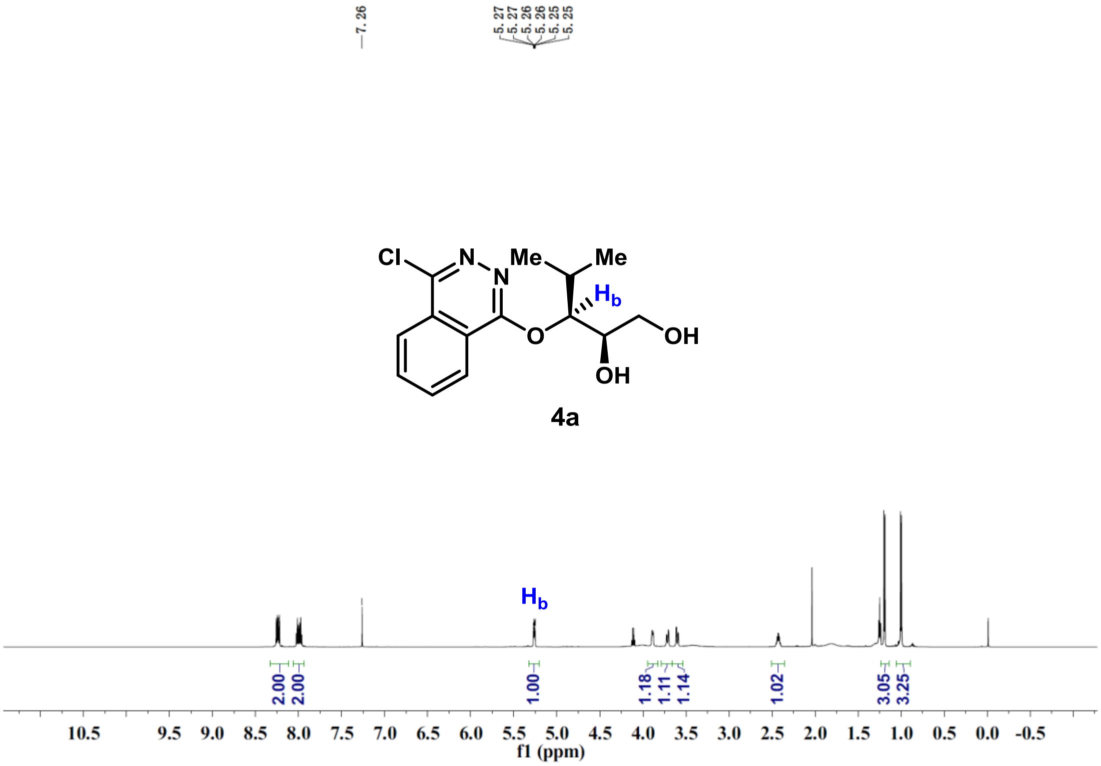

Supplementary Figure 15. ^{1}H NMR spectrum of dihydroxylated product **4a**.

HPLC (AD-H, 0.46*25 cm, 5μm, hexane/isopropanol = 98/2, flow = 1.0 mL/min, detection at 210 nm), retention time = 6.688 min (major) and 7.429 min (minor).

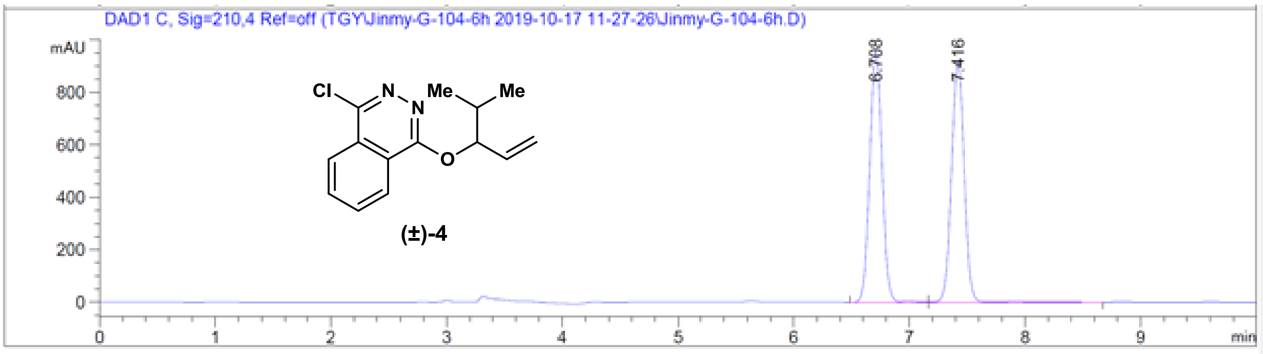

Signal 2: DAD1 C, Sig=210,4 Ref=off

Peak #	RetTime [min]	Type	Width [min]	Area [mAU*s]	Height [mAU]	Area %
1	6.708	BV R	0.1127	7007.53223	958.41815	49.8104
2	7.416	VV R	0.1187	7060.88672	920.27832	50.1896

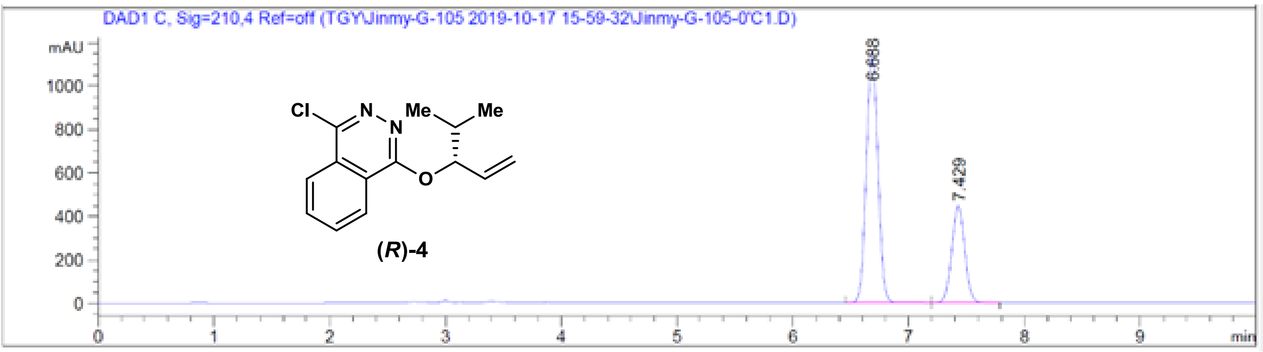

Signal 2: DAD1 C, Sig=210,4 Ref=off

Peak #	RetTime [min]	Type	Width [min]	Area [mAU*s]	Height [mAU]	Area %
1	6.688	BB	0.1069	8058.50244	1161.45093	69.9321
2	7.429	BB	0.1207	3464.81372	445.53326	30.0679

Supplementary Figure 16. HPLC chromatogram for ***(R)*-4**.

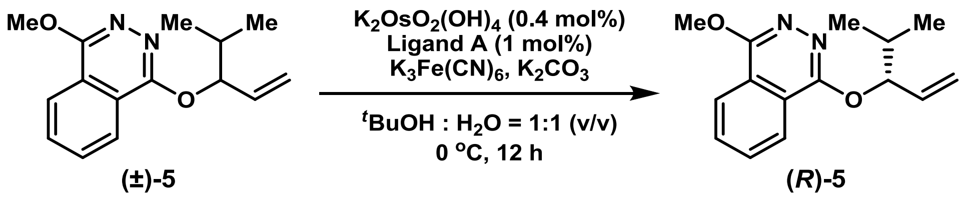

The general procedure **IV** was followed. The conversion of **(±)-5** was determined by crude ^{1}H NMR (51% conversion, 49% yield, 38% ee).

Conversion (%) = [1.03 / (1.00 + 1.03)] % = 51%

S = In [(1 – conv)(1 – ee)] / In [(1 – conv)(1 + ee)] = 3

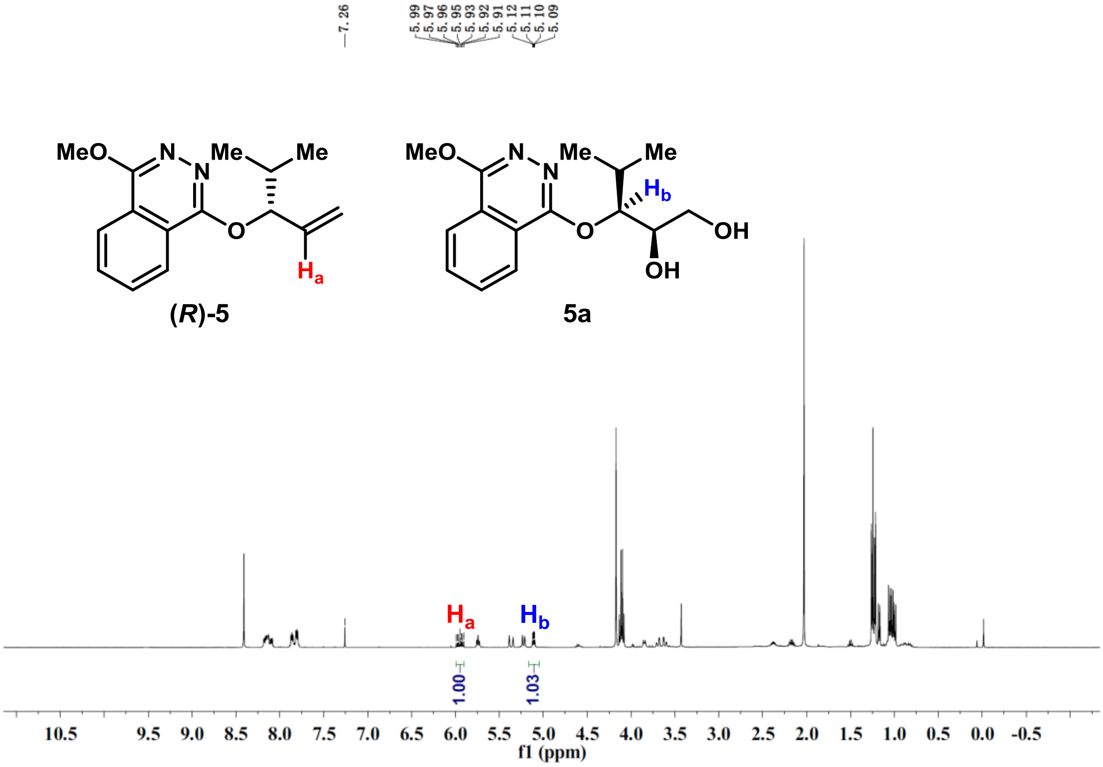

Supplementary Figure 17. ^{1}H NMR spectrum of crude mixture of compound **(*R*)-5** and **5a**

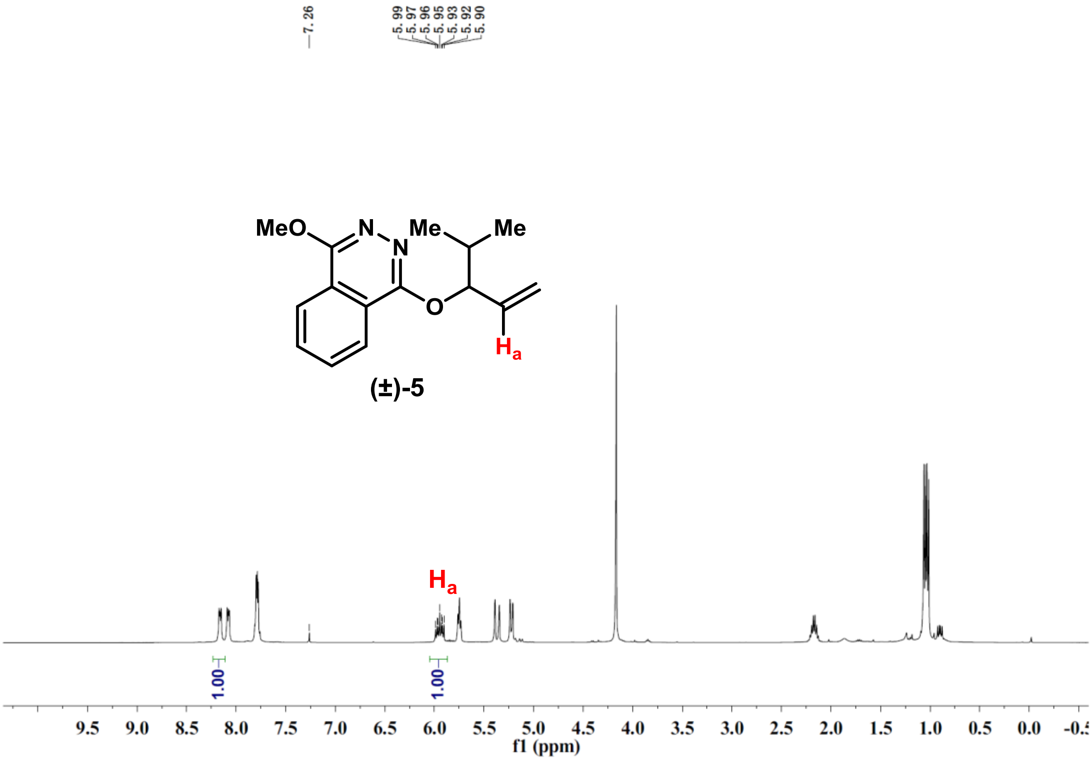

Supplementary Figure 18. ^{1}H NMR spectrum of starting material **(±)-5**.

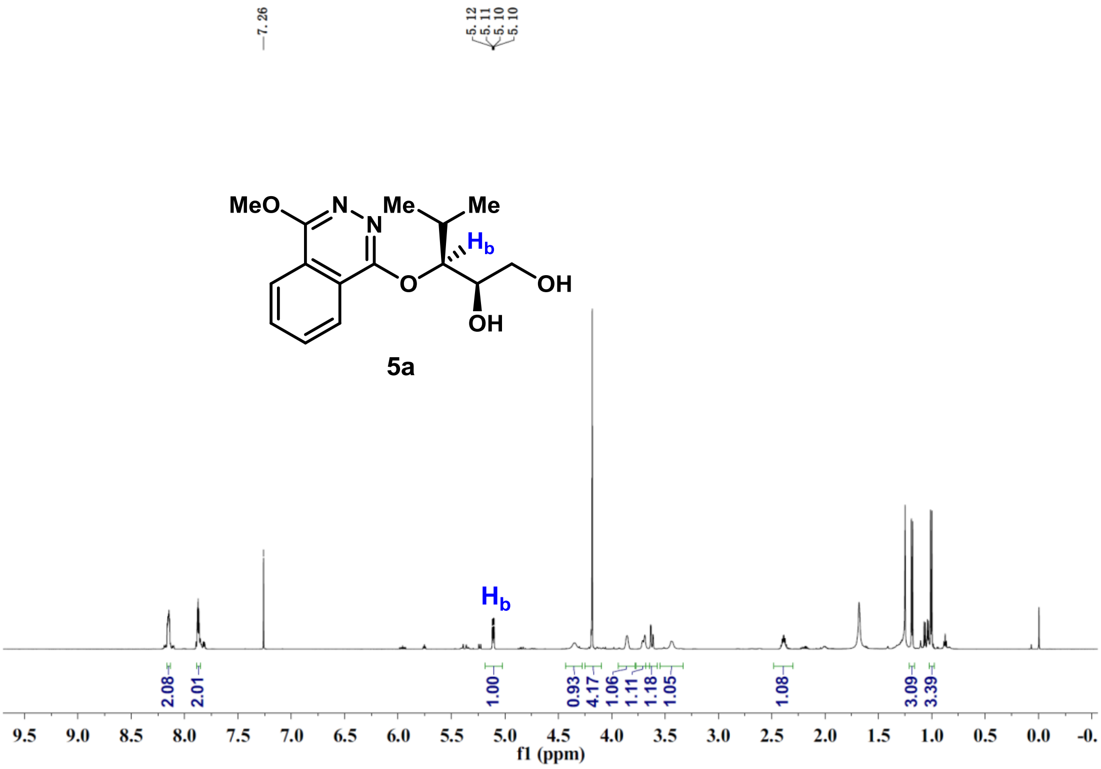

Supplementary Figure 19. ^{1}H NMR spectrum of dihydroxylated product **5a**.

HPLC (OD-H, 0.46*25 cm, 5μm, hexane/isopropanol = 95/5, flow = 1.0 mL/min, detection at 210 nm), retention time = 4.942 min (minor) and 7.955 min (major).

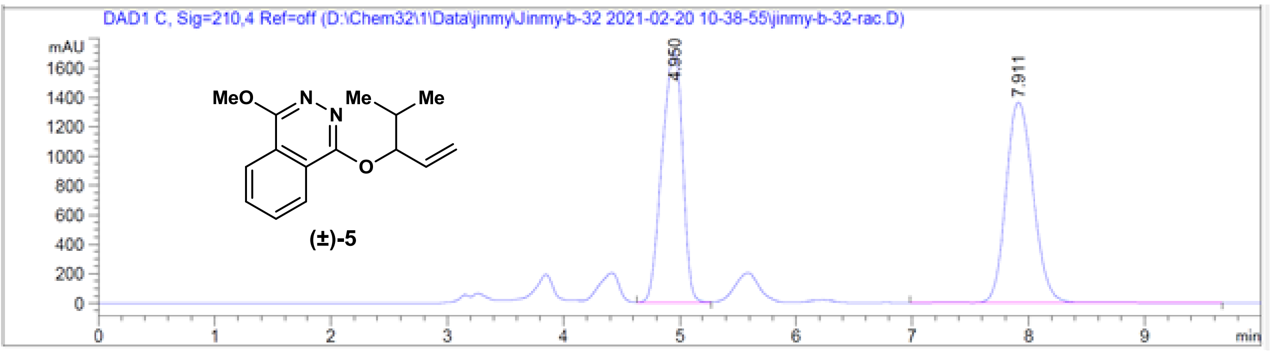

Signal 2: DAD1 C, Sig=210,4 Ref=off

Peak #	RetTime [min]	Type	Width [min]	Area [mAU*s]	Height [mAU]	Area %
1	4.950	BV	0.2018	2.08431e4	1714.91040	48.2155
2	7.911	VV R	0.2554	2.23860e4	1366.96814	51.7845

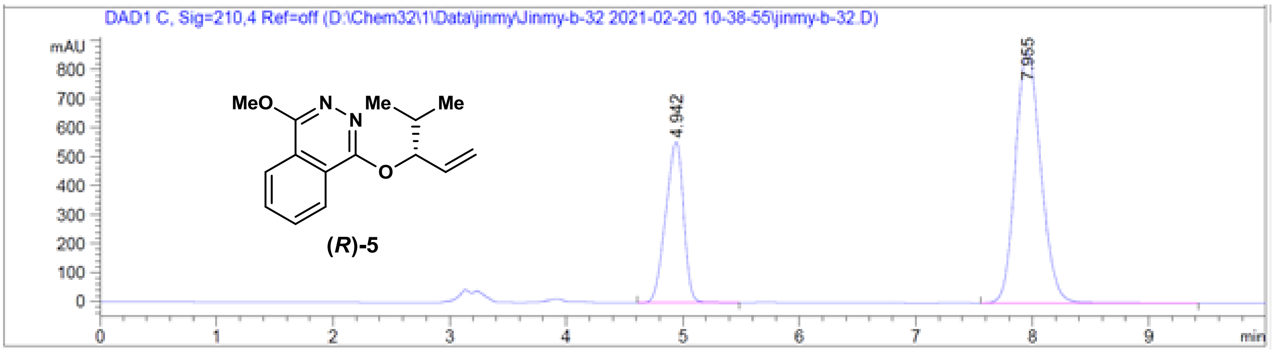

Signal 2: DAD1 C, Sig=210,4 Ref=off

Peak #	RetTime [min]	Type	Width [min]	Area [mAU*s]	Height [mAU]	Area %
1	4.942	BB	0.1625	5923.92480	555.09937	30.9739
2	7.955	BB	0.2342	1.32016e4	874.23737	69.0261

Supplementary Figure 20. HPLC chromatogram for ***(R)*-5**.

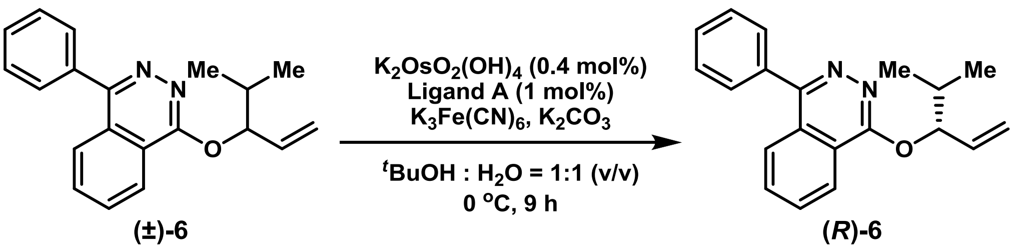

The general procedure **IV** was followed. The conversion of **(±)-6** was determined by crude ^{1}H NMR (44% conversion, 56% yield, 44% ee).

Conversion (%) = [(1.77 – 1.00) / 1.77] % = 44%

S = In [(1 – conv)(1 – ee)] / In [(1 – conv)(1 + ee)] = 5

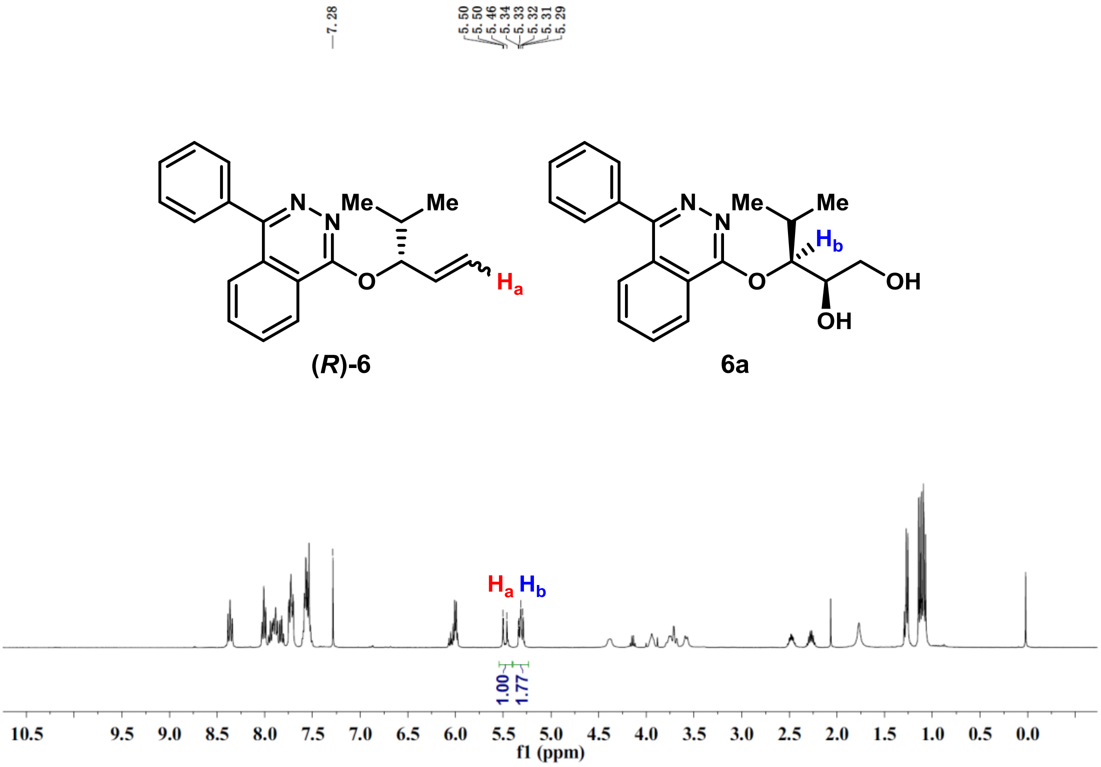

Supplementary Figure 21. ^{1}H NMR spectrum of crude mixture of compound **(*R*)-6** and **6a.**

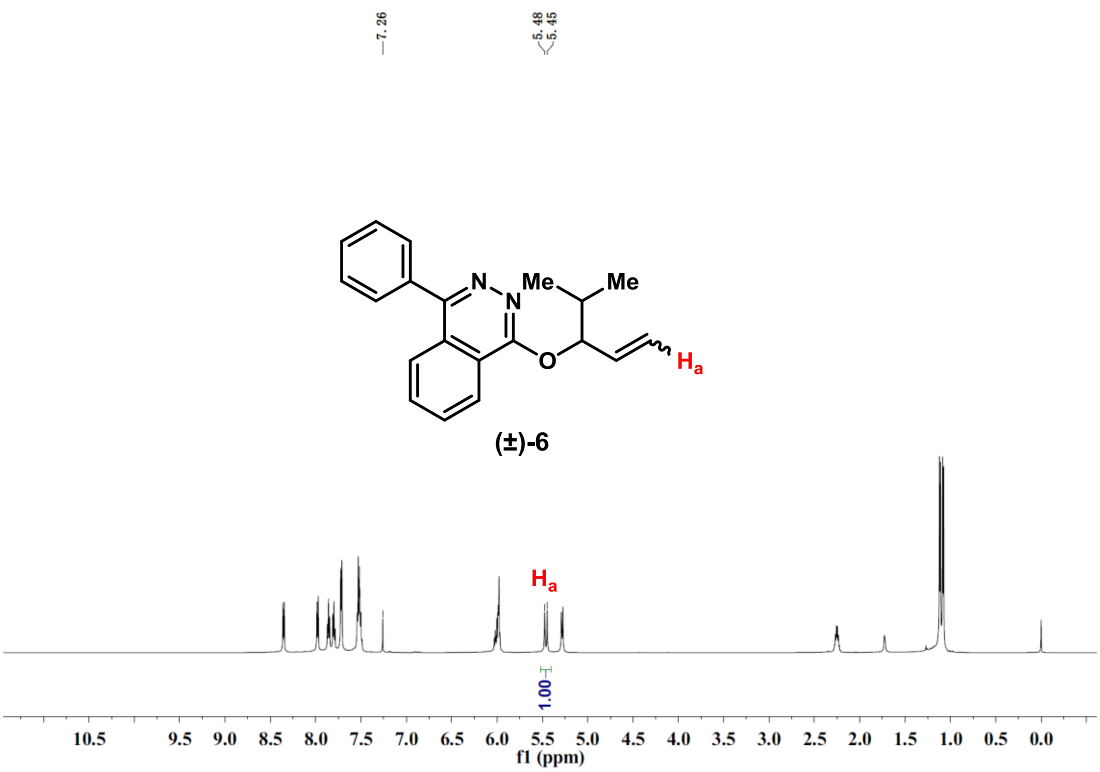

Supplementary Figure 22. ^{1}H NMR spectrum of starting material **(±)-6**.

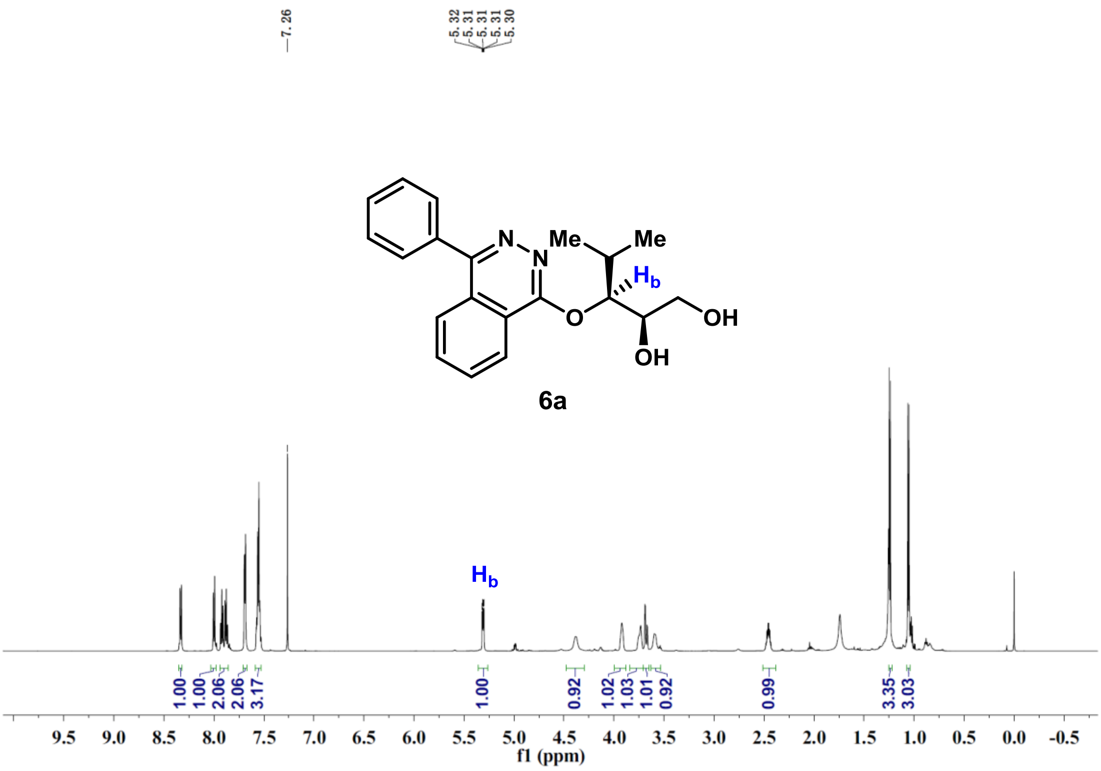

Supplementary Figure 23. ^{1}H NMR spectrum of dihydroxylated product **6a**.

HPLC (AD-H, 0.46*25 cm, 5μm, hexane/isopropanol = 80/20, flow = 1.0 mL/min, detection at 210 nm), retention time = 6.106 min (major) and 8.544 min (minor).

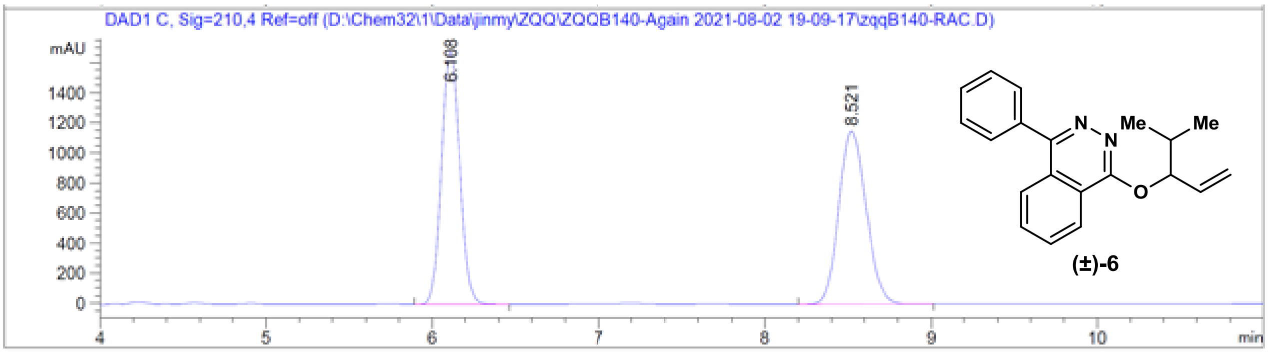

Signal 2: DAD1 C, Sig=210,4 Ref=off

Peak #	RetTime [min]	Type	Width [min]	Area [mAU*s]	Height [mAU]	Area %
1	6.108	VB	0.1193	1.28742e4	1681.15039	49.4995
2	8.521	BB	0.1772	1.31346e4	1150.34070	50.5005

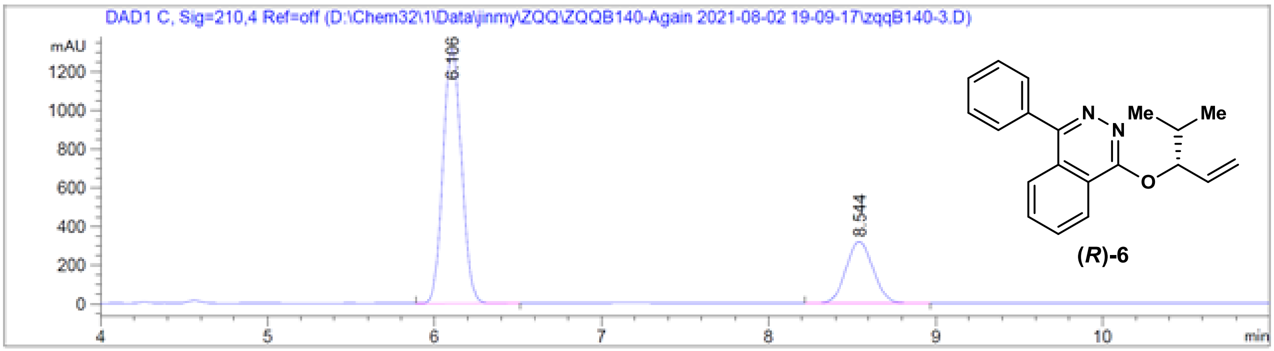

Signal 2: DAD1 C, Sig=210,4 Ref=off

Peak #	RetTime [min]	Type	Width [min]	Area [mAU*s]	Height [mAU]	Area %
1	7.374	VB R	0.2292	3.48489e4	2343.67236	72.1621
2	10.926	BB	0.3091	1.34437e4	650.49036	27.8379

Supplementary Figure 24. HPLC chromatogram for **(*R*)-6**.

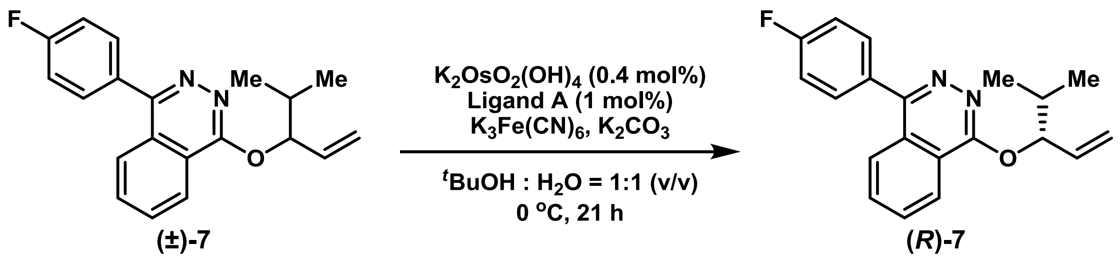

The general procedure was **IV** followed. The conversion of **(±)-7** was determined by crude ^{1}H NMR (58% conversion, 42% yield, 72% ee).

Conversion (%) = [(2.40 – 1.00) / 2.40] % = 58%

S = In [(1 – conv)(1 – ee)] / In [(1 – conv)(1 + ee)] = 6

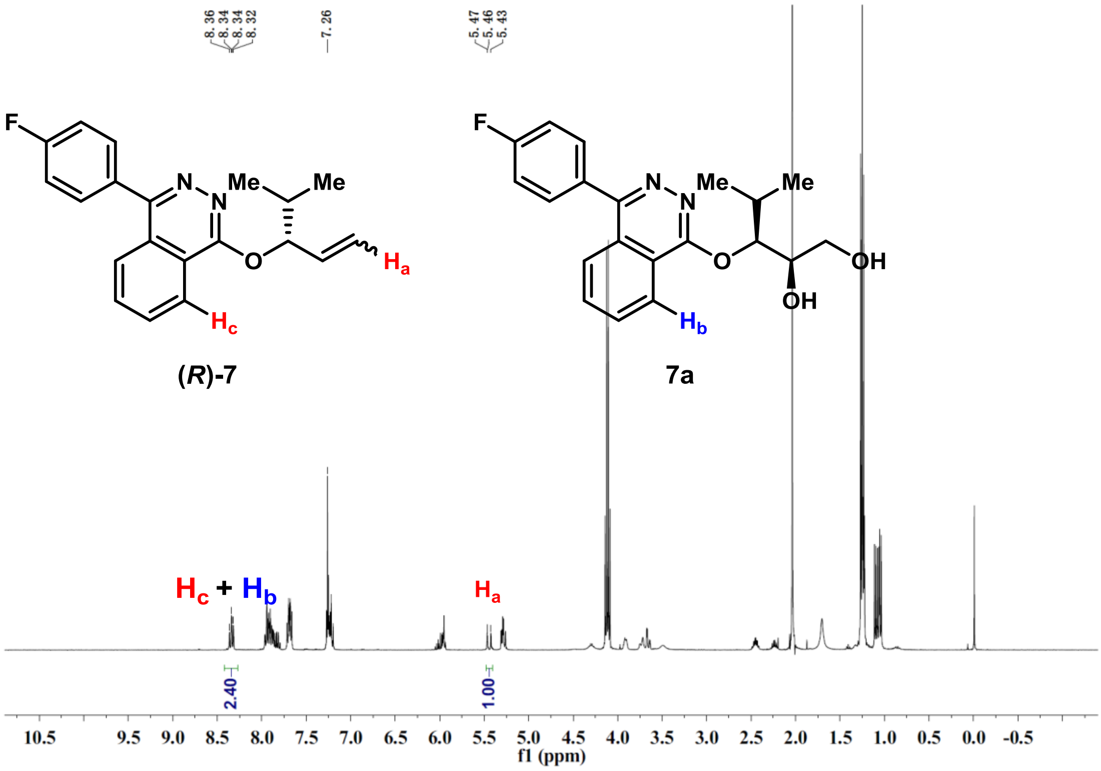

Supplementary Figure 25. ^{1}H NMR spectrum of crude mixture of compound **(*R*)-7** and **7a.**

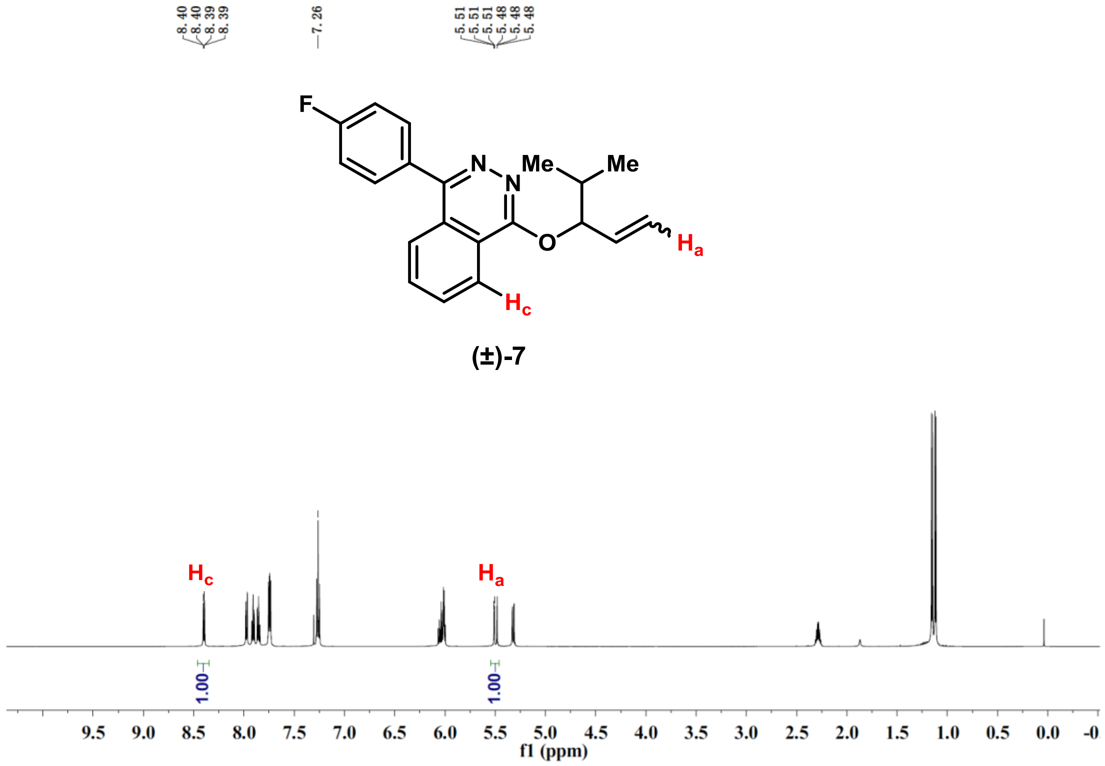

Supplementary Figure 26. ^{1}H NMR spectrum of starting material **(±)-7**.

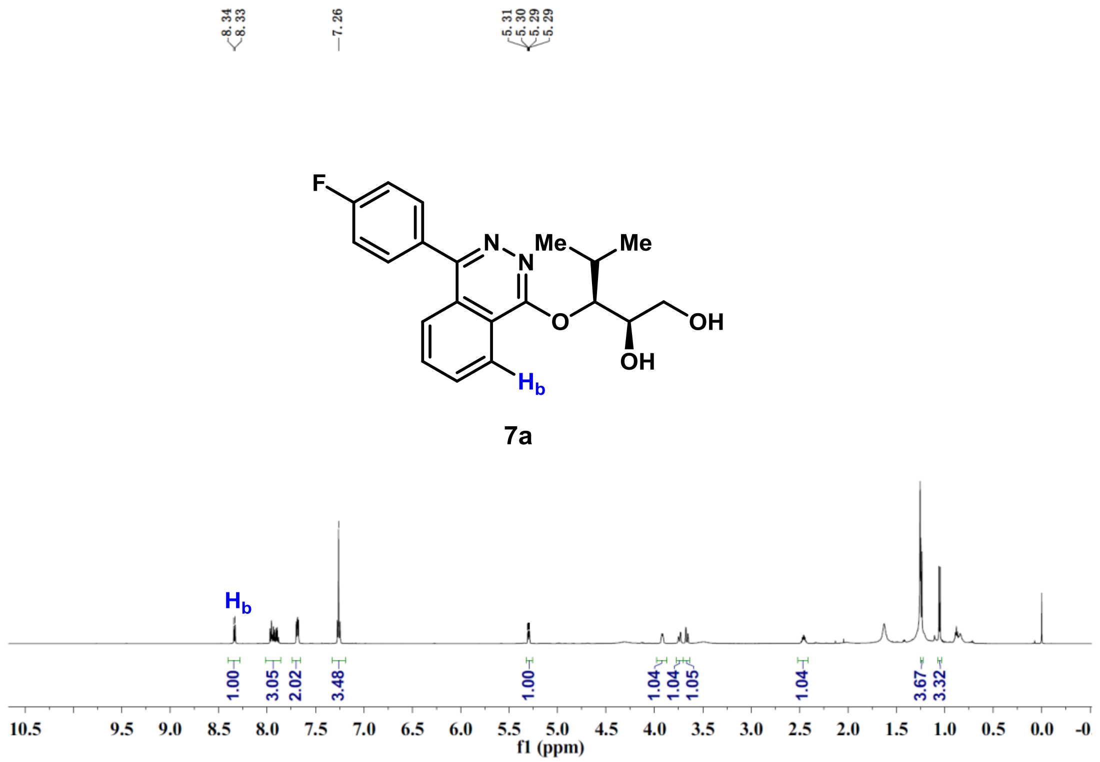

Supplementary Figure 27. ^{1}H NMR spectrum of dihydroxylated product **7a**.

HPLC (AD-H, 0.46*25 cm, 5μm, hexane/isopropanol = 80/20, flow = 1.0 mL/min, detection at 210 nm), retention time = 8.875 min (major) and 11.895 min (minor).

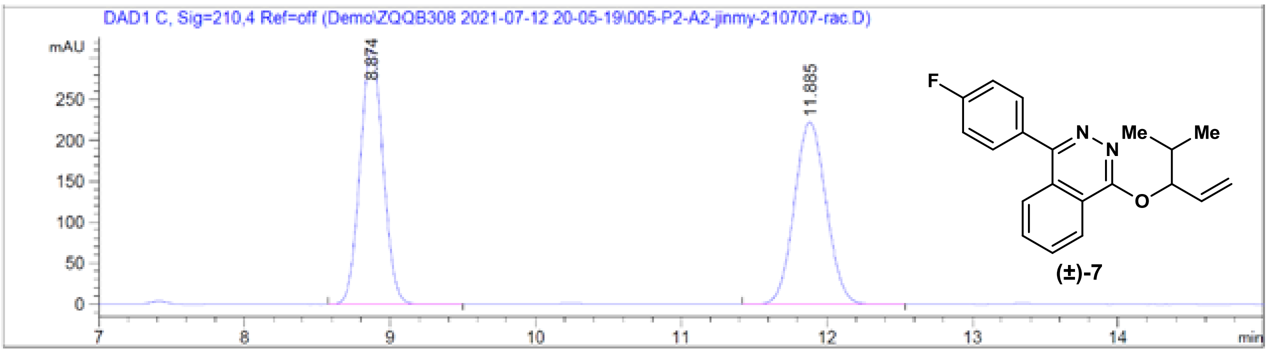

Signal 2: DAD1 C, Sig=210,4 Ref=off

Peak #	RetTime [min]	Type	Width [min]	Area [mAU*s]	Height [mAU]	Area %
1	8.874	BB	0.1690	3381.80151	310.54916	50.0489
2	11.885	BB	0.2367	3375.19287	222.91100	49.9511

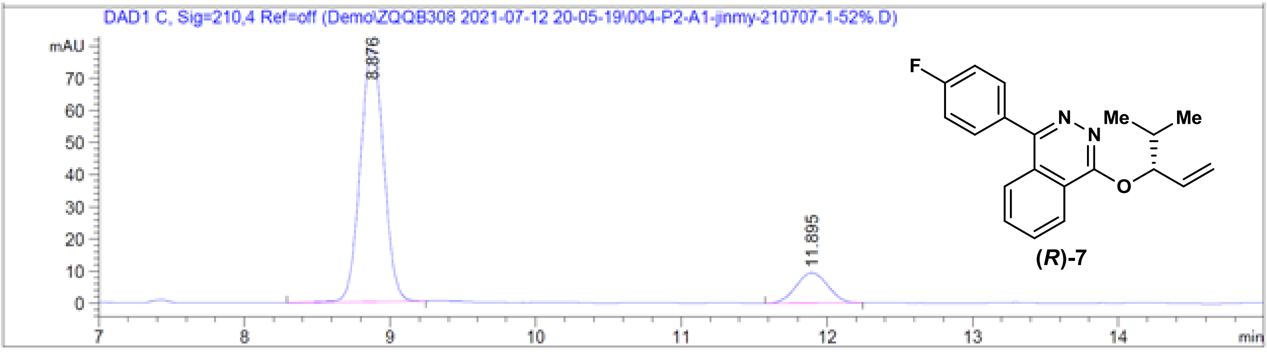

Signal 2: DAD1 C, Sig=210,4 Ref=off

Peak #	RetTime [min]	Type	Width [min]	Area [mAU*s]	Height [mAU]	Area %
1	8.876	BB	0.1734	872.70239	78.69175	85.8399
2	11.895	BB	0.2339	143.95990	9.55091	14.1601

Supplementary Figure 28. HPLC chromatogram for ***(R)*-7**.

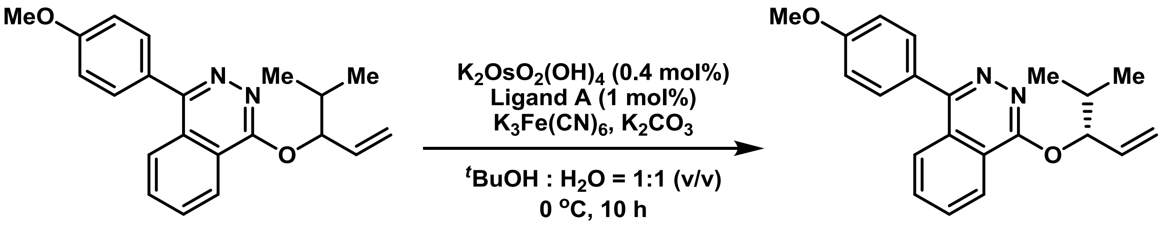

(±)-8 **(*R*)-8**

The general procedure was **IV** followed. The conversion of **(±)-8** was determined by crude ^{1}H NMR (64% conversion, 36% yield, 80% ee).

Conversion (%) = [(2.83 – 1.00) / 2.83] % = 64%

S = In [(1 – conv)(1 – ee)] / In [(1 – conv)(1 + ee)] = 6

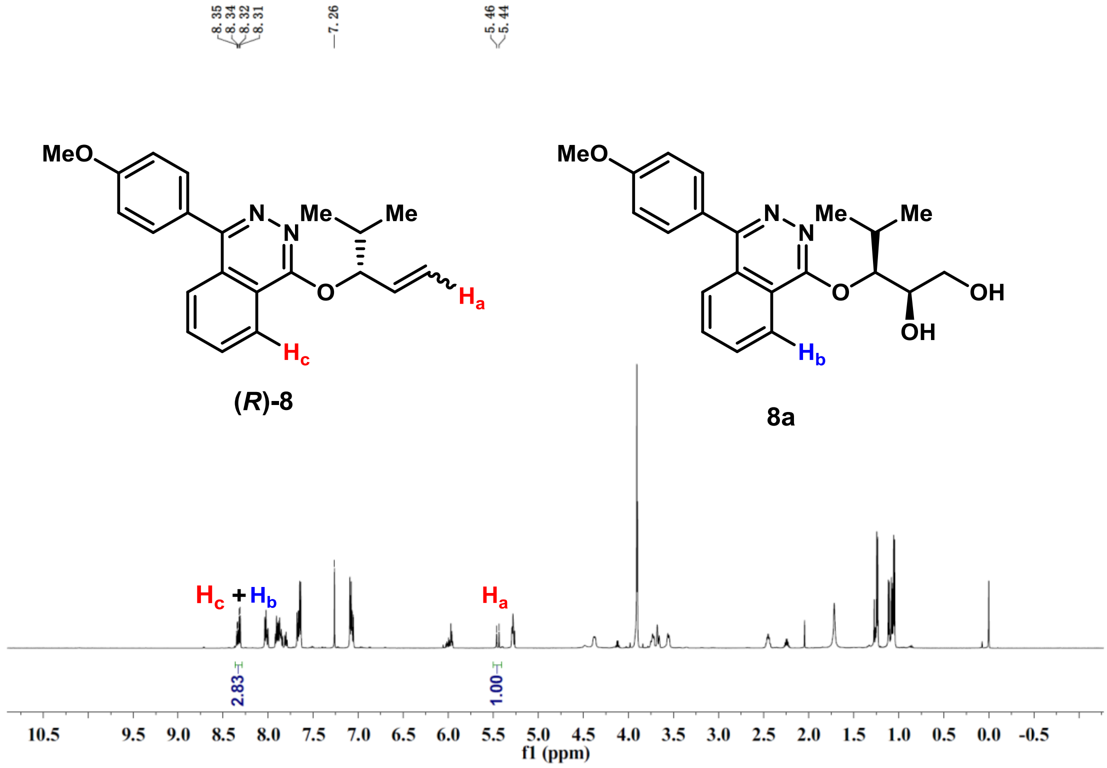

Supplementary Figure 29. ^{1}H NMR spectrum of crude mixture of compound **(*R*)-8** and **8a.**

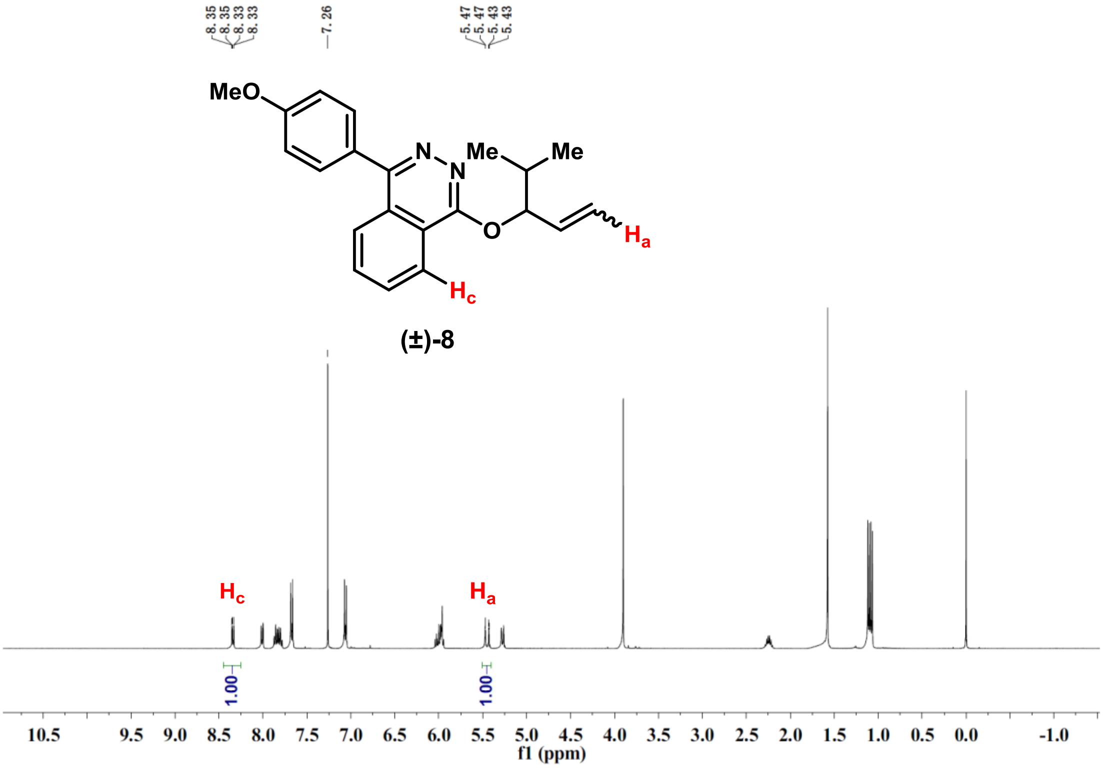

Supplementary Figure 30. ^{1}H NMR spectrum of starting material **(±)-8**.

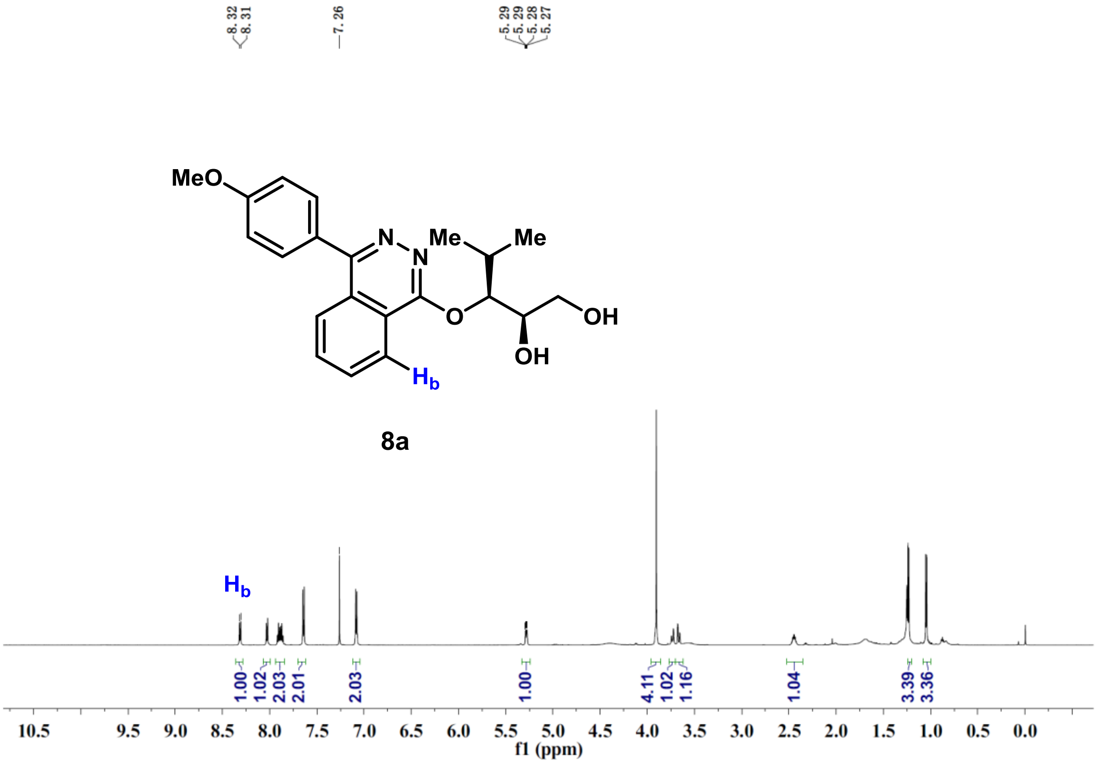

Supplementary Figure 31. ^{1}H NMR spectrum of dihydroxylated product **8a**.

HPLC (AD-H, 0.46*25 cm, 5μm, hexane/isopropanol = 80/20, flow = 1.0 mL/min, detection at 210 nm), retention time = 8.821 min (major) and 13.044 min (minor).

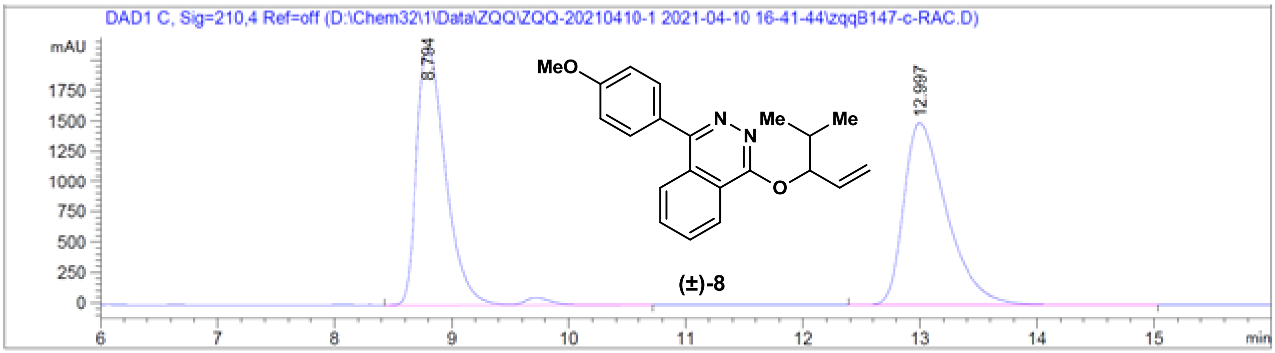

Signal 2: DAD1 C, Sig=210,4 Ref=off

Peak #	RetTime [min]	Type	Width [min]	Area [mAU*s]	Height [mAU]	Area %
1	8.794	BV R	0.2678	3.73682e4	2105.31226	49.3652
2	12.997	BB	0.3876	3.83293e4	1502.67896	50.6348

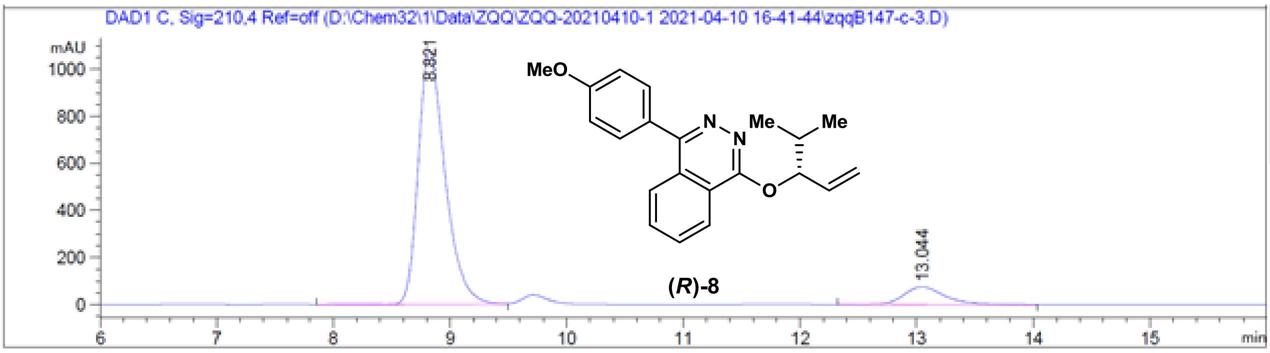

Signal 2: DAD1 C, Sig=210,4 Ref=off

Peak #	RetTime [min]	Type	Width [min]	Area [mAU*s]	Height [mAU]	Area %
1	8.821	MM R	0.2677	1.73639e4	1081.02441	90.1524
2	13.044	MM R	0.4151	1896.70959	76.14605	9.8476

Supplementary Figure 32. HPLC chromatogram for **(*R*)-8**.

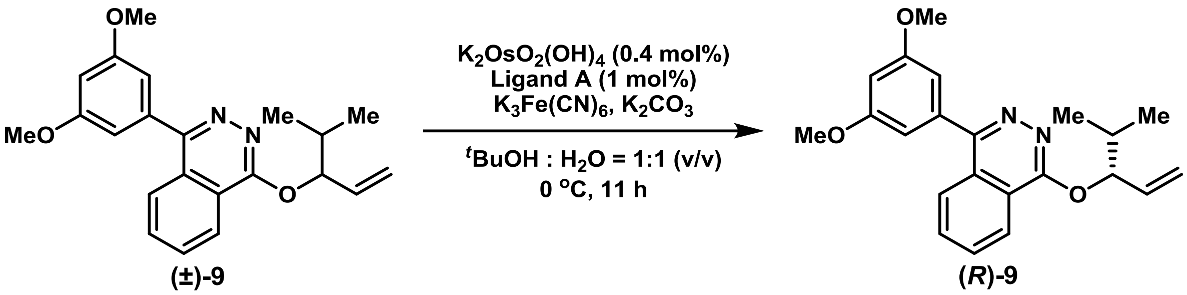

The general procedure was **IV** followed. The conversion of **(±)-9** was determined by crude ^{1}H NMR (62% conversion, 38% yield, 92% ee).

Conversion (%) = [(2.67 – 1.00) / 2.67] % = 62%

S = In [(1 – conv)(1 – ee)] / In [(1 – conv)(1 + ee)] = 11

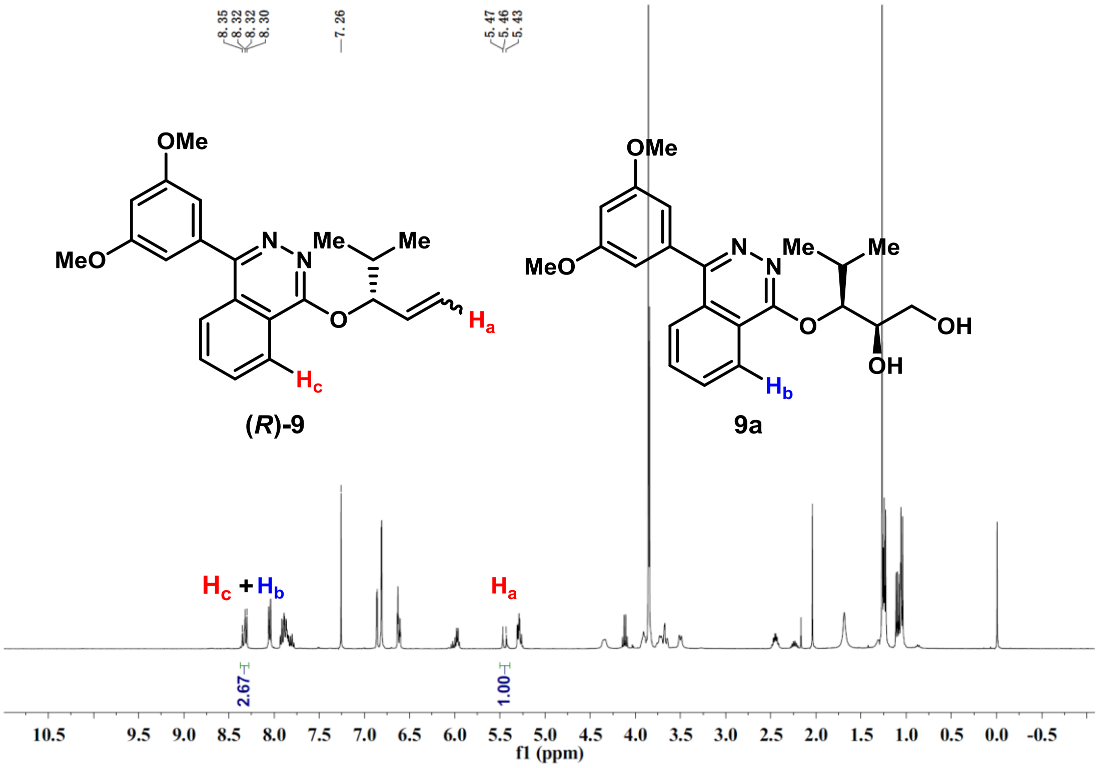

Supplementary Figure 33. ^{1}H NMR spectrum of crude mixture of compound **(*R*)-9** and **9a.**

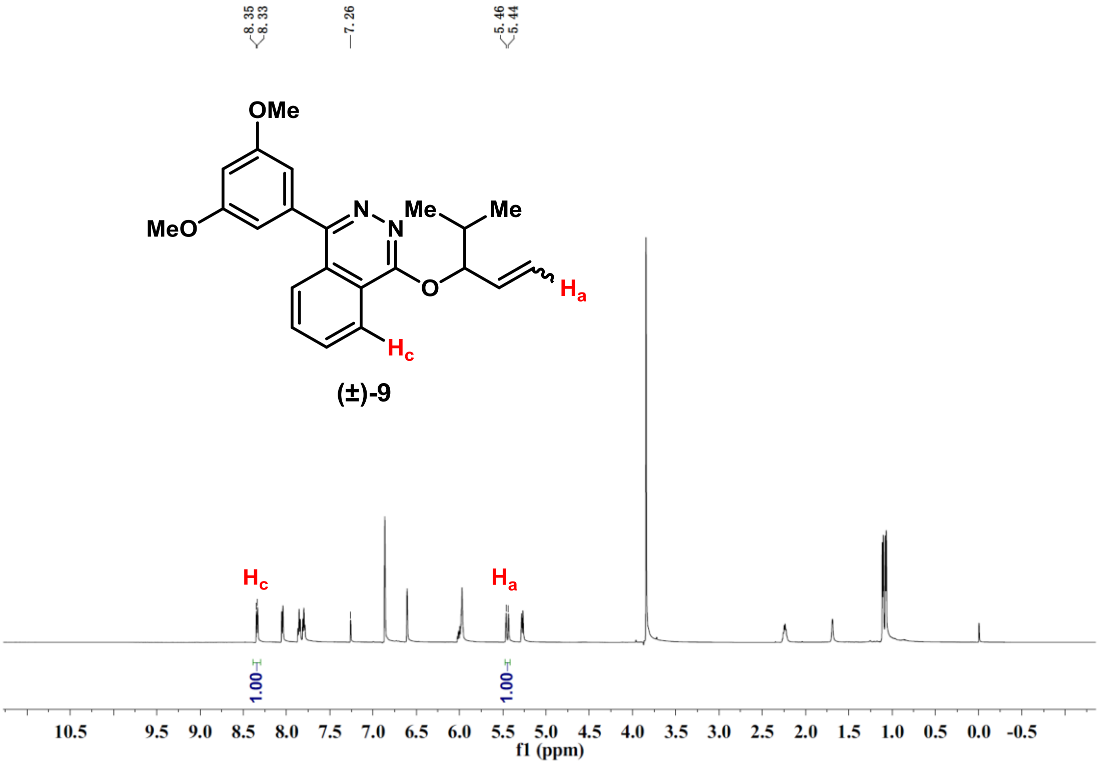

Supplementary Figure 34. ^{1}H NMR spectrum of starting material **(±)-9**.

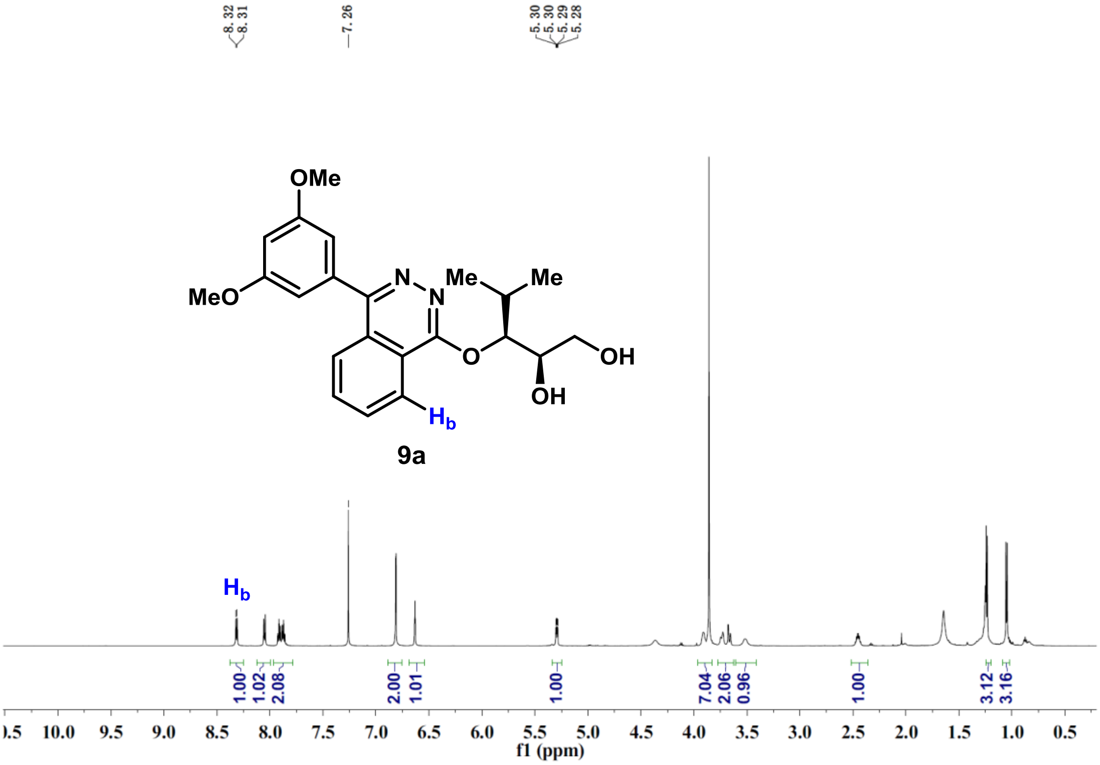

Supplementary Figure 35. ^{1}H NMR spectrum of dihydroxylated product **9a**.

HPLC (AD-H, 0.46*25 cm, 5μm, hexane/isopropanol = 90/10, flow = 1.0 mL/min, detection at 210 nm), retention time = 7.431 min (major) and 8.752 min (minor).

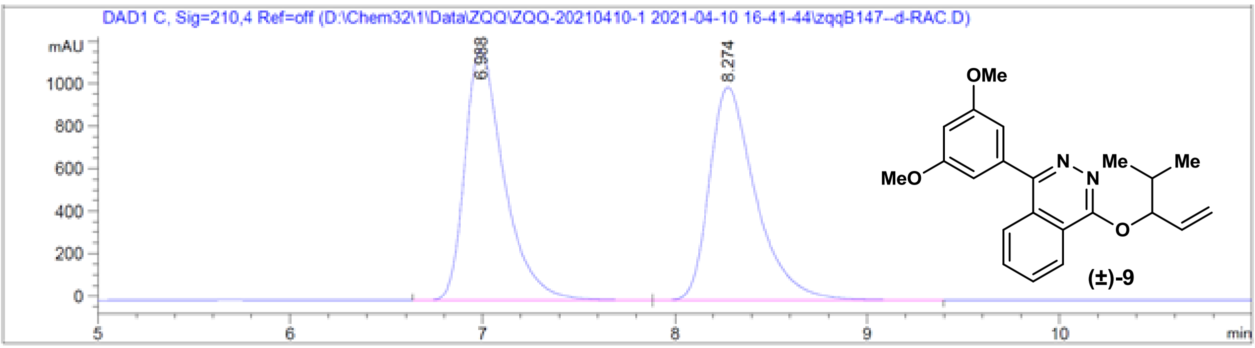

Signal 2: DAD1 C, Sig=210,4 Ref=off

Peak #	RetTime [min]	Type	Width [min]	Area [mAU*s]	Height [mAU]	Area %
1	6.988	BB	0.2128	1.67049e4	1182.80225	49.8085
2	8.274	BB	0.2515	1.68334e4	1004.55255	50.1915

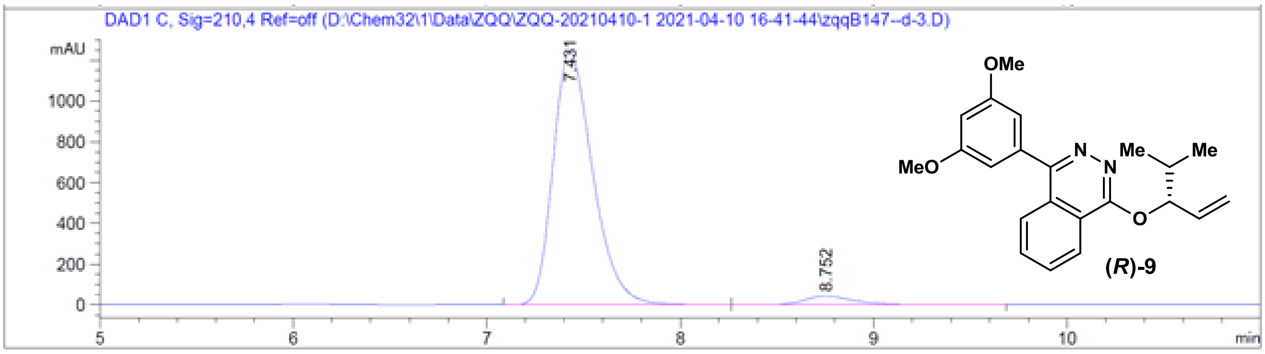

Signal 2: DAD1 C, Sig=210,4 Ref=off

Peak #	RetTime [min]	Type	Width [min]	Area [mAU*s]	Height [mAU]	Area %
1	7.431	BB	0.2209	1.77845e4	1243.00720	96.0022
2	8.752	BB	0.2660	740.60132	41.93873	3.9978

Supplementary Figure 36. HPLC chromatogram for ***(R)*-9**.

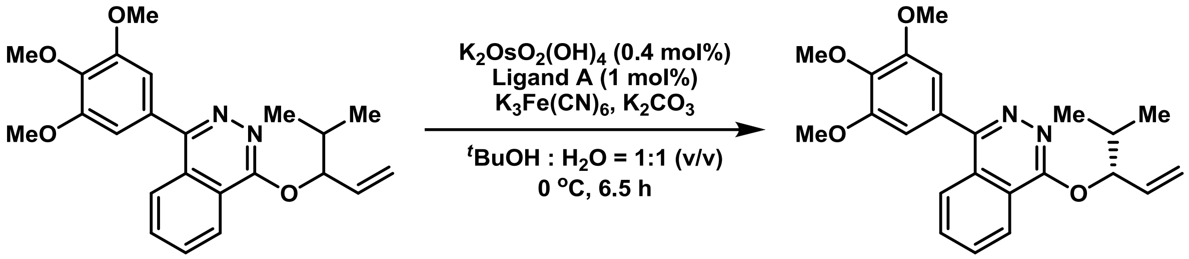

(±)-10 (*R*)-10

The general procedure was **III** followed. The conversion of **(±)-10** was determined by crude ^{1}H NMR (53% conversion, 47% yield, 92% ee).

$[\alpha]_D^{25}$ = – 6.00 (*c* 0.60, $CHCl_3$)

Conversion (%) = [(2.12 – 1.00) / 2.12] % = 53%

S = In [(1 – conv)(1 – ee)] / In [(1 – conv)(1 + ee)] = 32

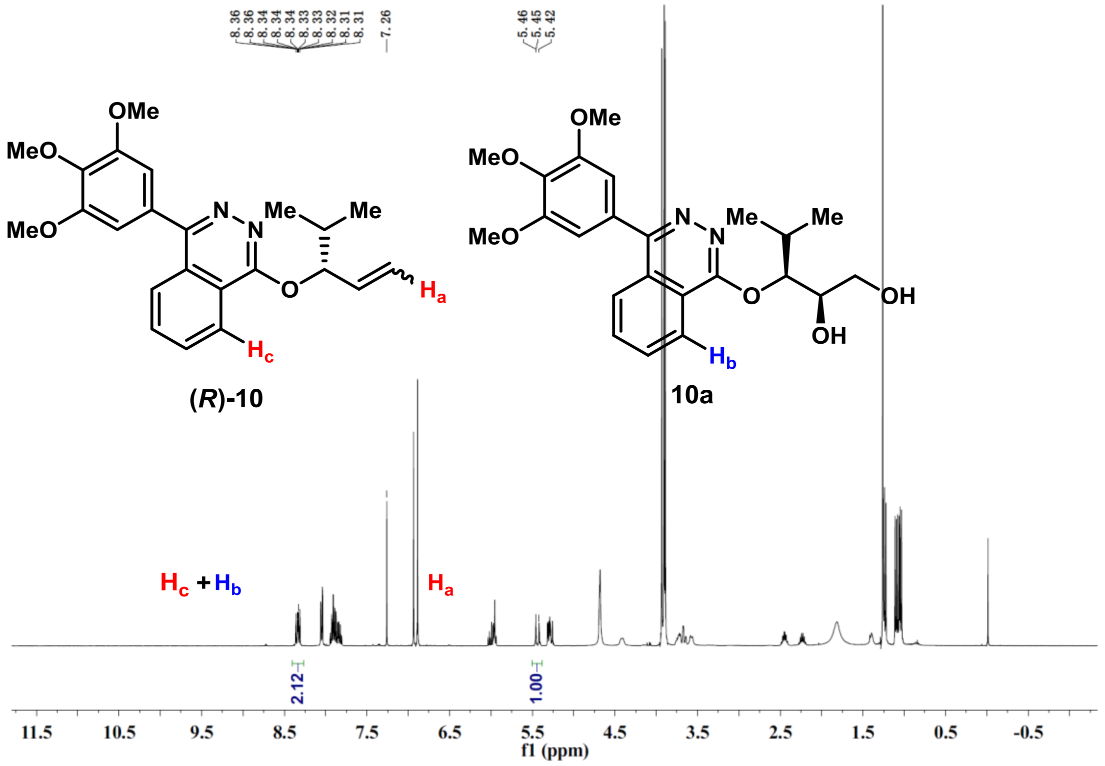

Supplementary Figure 37. ^{1}H NMR spectrum of crude mixture of compound **(*R*)-10** and **10a.**

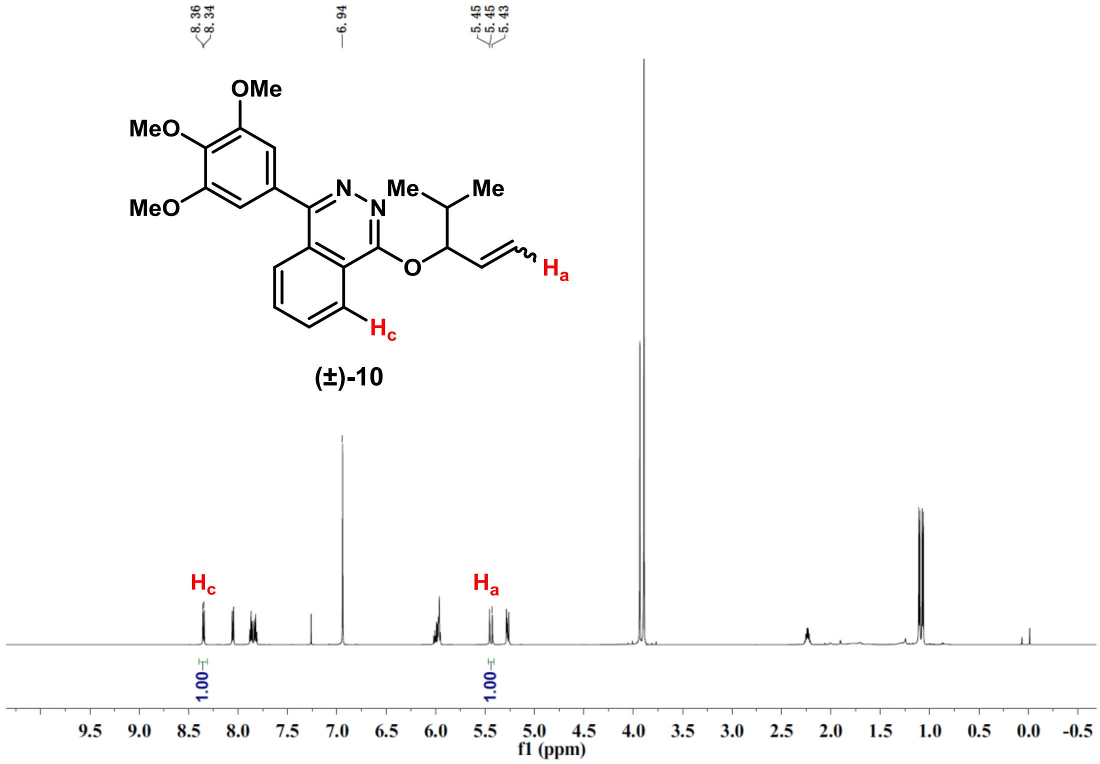

Supplementary figure 38. ¹H NMR spectrum of starting material **(±)-10**.

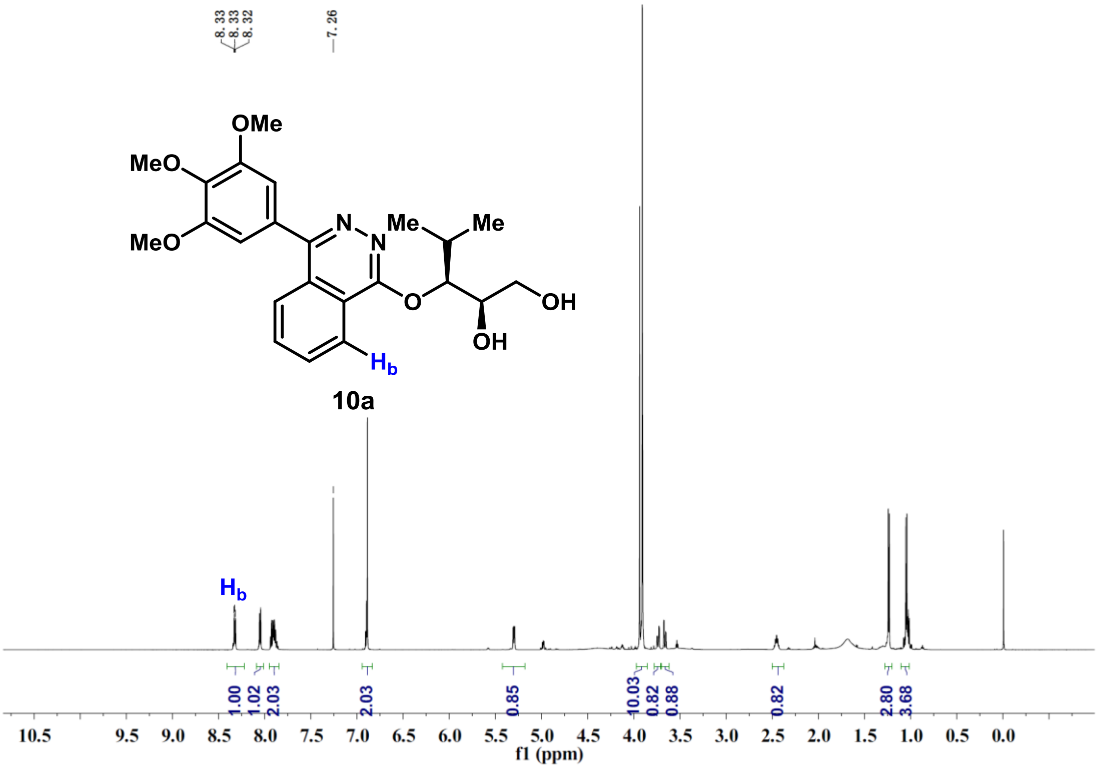

Supplementary Figure 39. ¹H NMR spectrum of dihydroxylated product **10a**.

HPLC (AD-H, 0.46*25 cm, 5μm, hexane/isopropanol = 90/10, flow = 1.0 mL/min, detection at 210 nm), retention time = 14.603 min (major) and 18.811 min (minor).

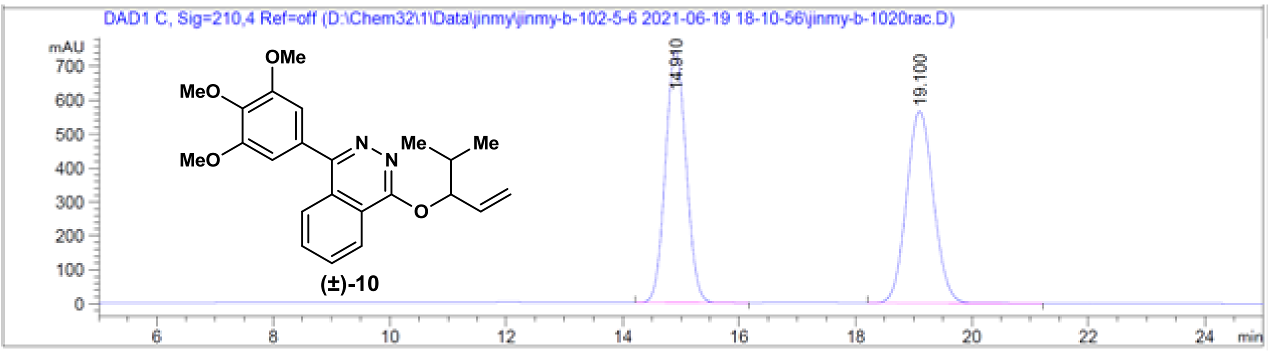

Signal 2: DAD1 C, Sig=210,4 Ref=off

Peak #	RetTime [min]	Type	Width [min]	Area [mAU*s]	Height [mAU]	Area %
1	14.910	BB	0.3837	1.81943e4	742.90076	49.9093
2	19.100	BB	0.5050	1.82604e4	565.13336	50.0907

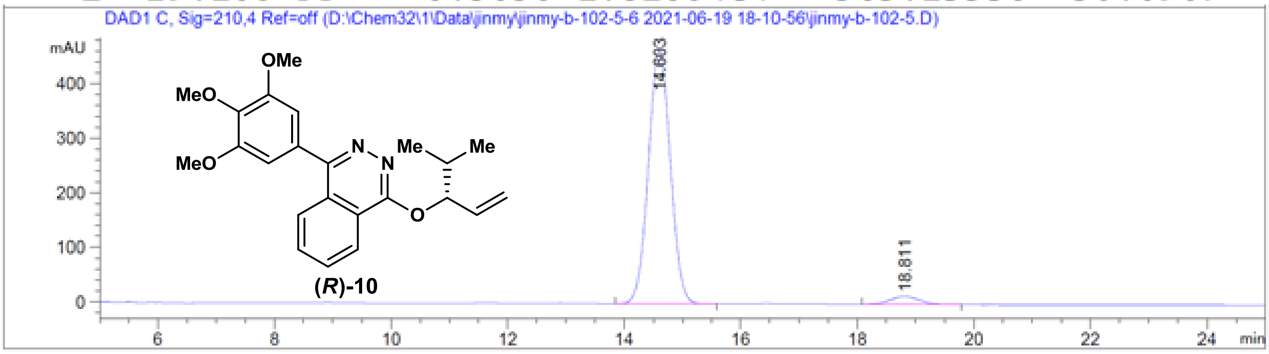

Signal 2: DAD1 C, Sig=210,4 Ref=off

Peak #	RetTime [min]	Type	Width [min]	Area [mAU*s]	Height [mAU]	Area %
1	14.603	BB	0.4126	1.22245e4	465.23315	96.0266
2	18.811	BB	0.5188	505.83209	14.79656	3.9734

Supplementary Figure 40. HPLC chromatogram for **(*R*)-10**.

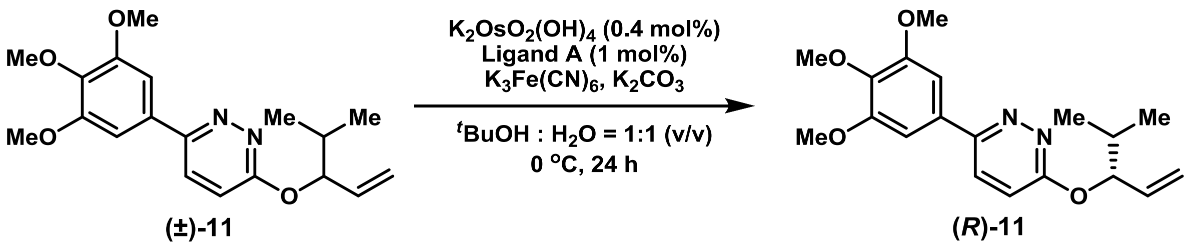

The general procedure was **III** followed. The conversion of **(±)-11** was determined by crude ^{1}H NMR (35% conversion, 65% yield, 18% ee).

Conversion (%) = [0.55/ (1.00 + 0.55)] % = 35%

S = In [(1 – conv)(1 – ee)] / In [(1 – conv)(1 + ee)] = 2

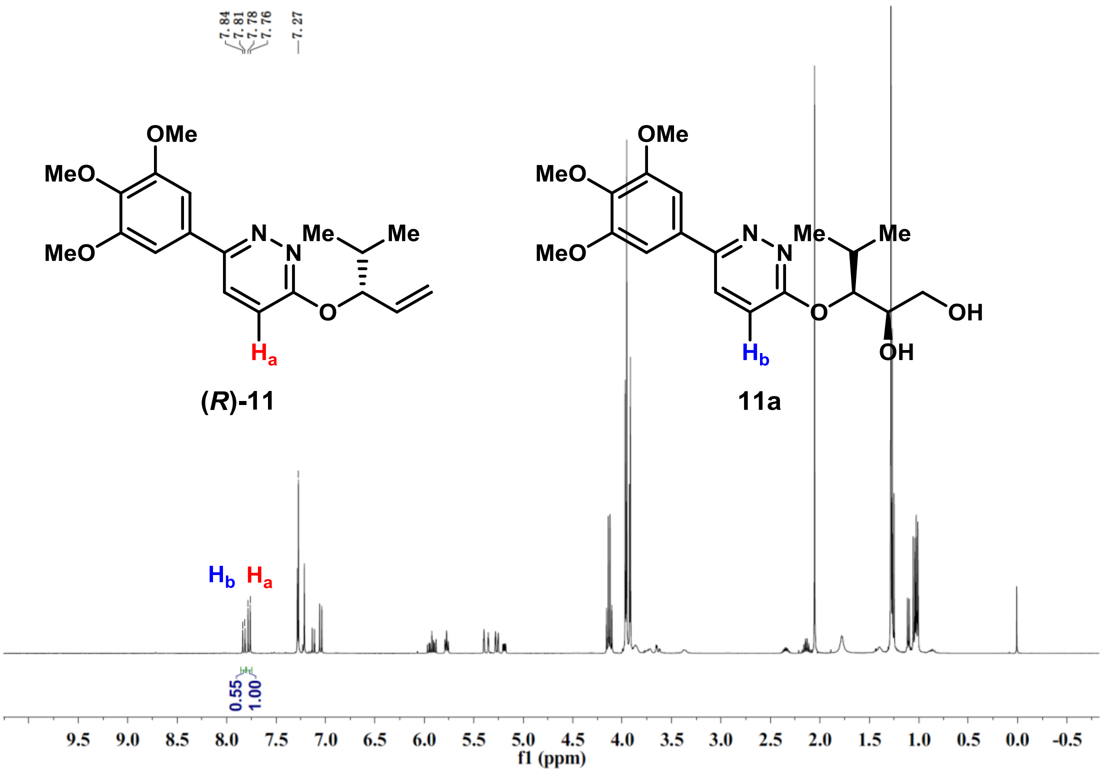

Supplementary Figure 41. ^{1}H NMR spectrum of crude mixture of compound **(*R*)-11** and **11a.**

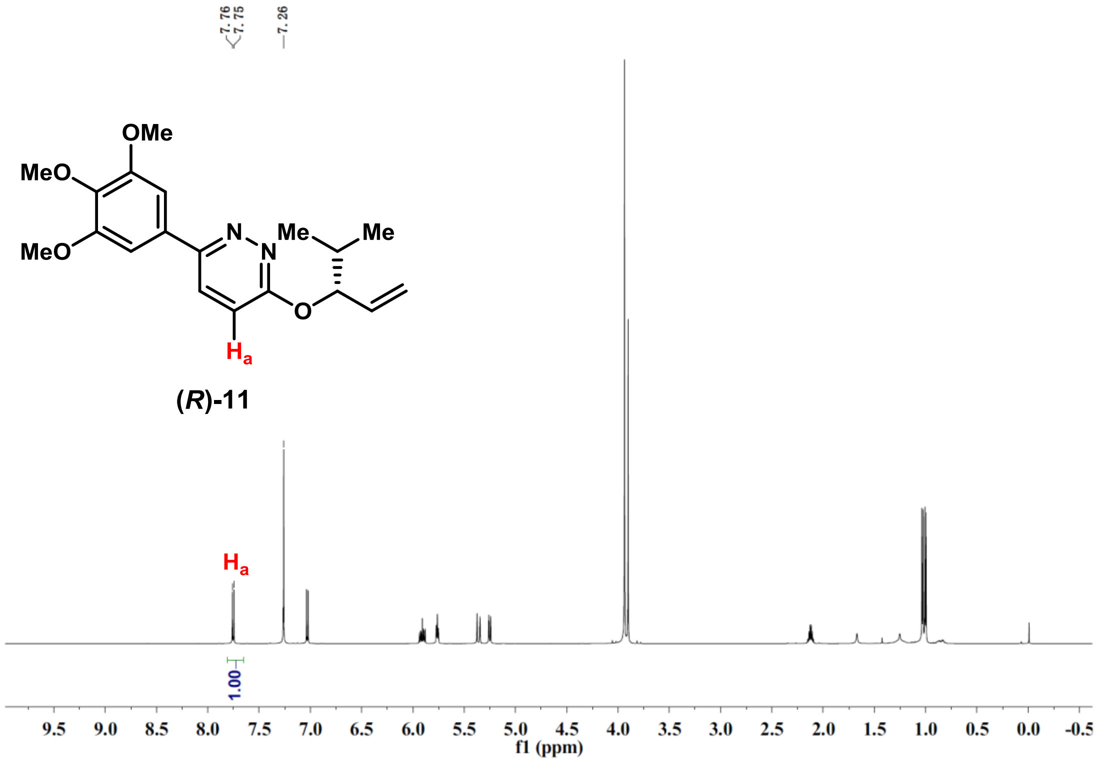

Supplementary Figure 42. ^{1}H NMR spectrum of starting material **(±)-11**.

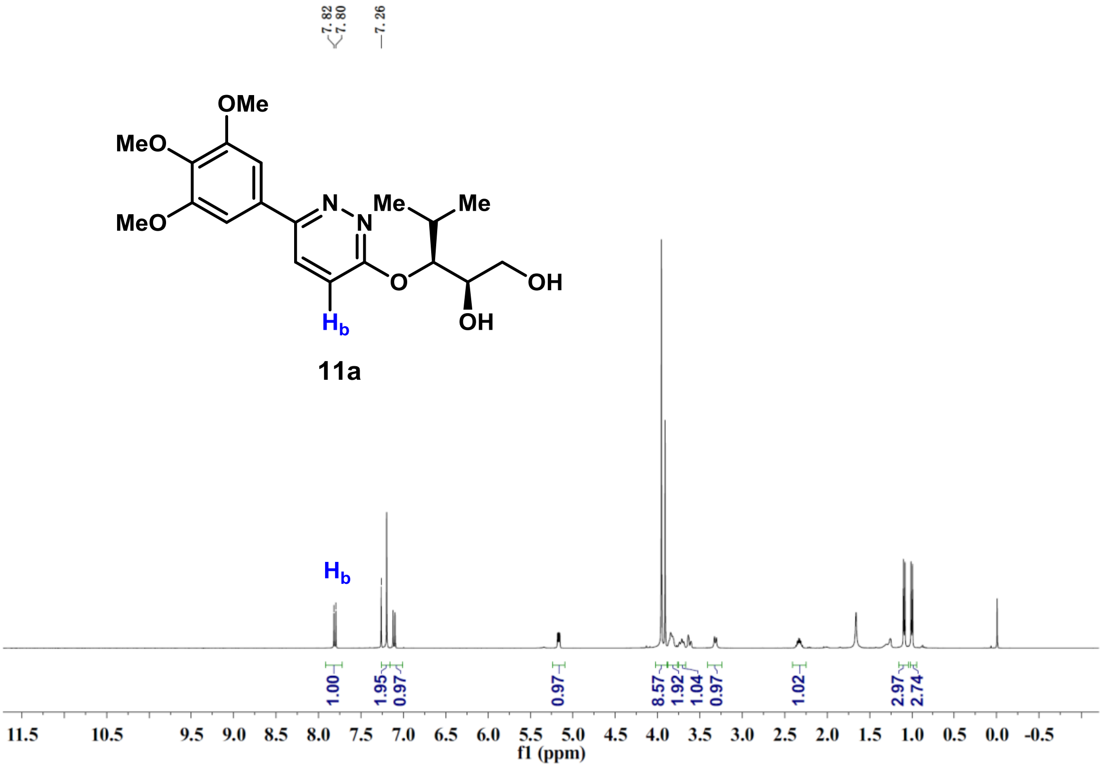

Supplementary Figure 43. ^{1}H NMR spectrum of dihydroxylated product **11a**.

HPLC (AD-H, 0.46*25 cm, 5μm, hexane/isopropanol = 80/20, flow = 1.0 mL/min, detection at 210 nm), retention time = 5.968 min (major) and 6.555 min (minor).

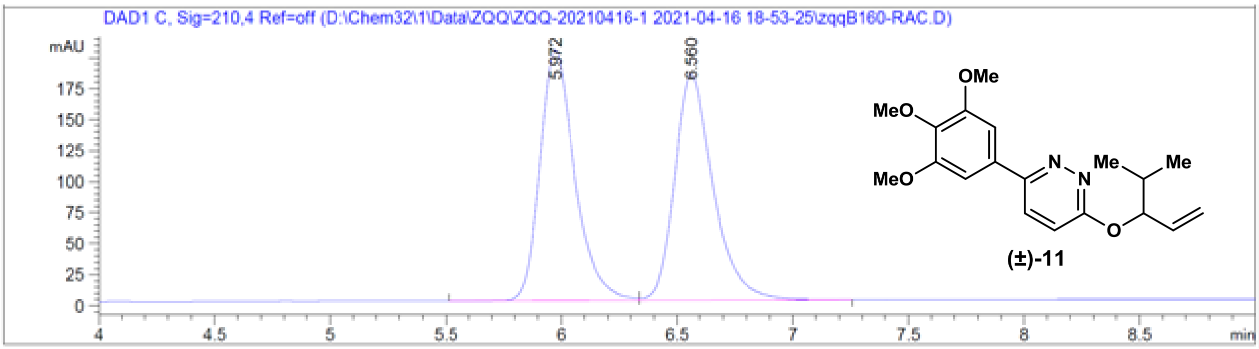

Signal 2: DAD1 C, Sig=210,4 Ref=off

Peak #	RetTime [min]	Type	Width [min]	Area [mAU*s]	Height [mAU]	Area %
1	5.972	BV	0.1578	2113.76221	202.46143	49.8360
2	6.560	VB	0.1760	2127.67310	182.50626	50.1640

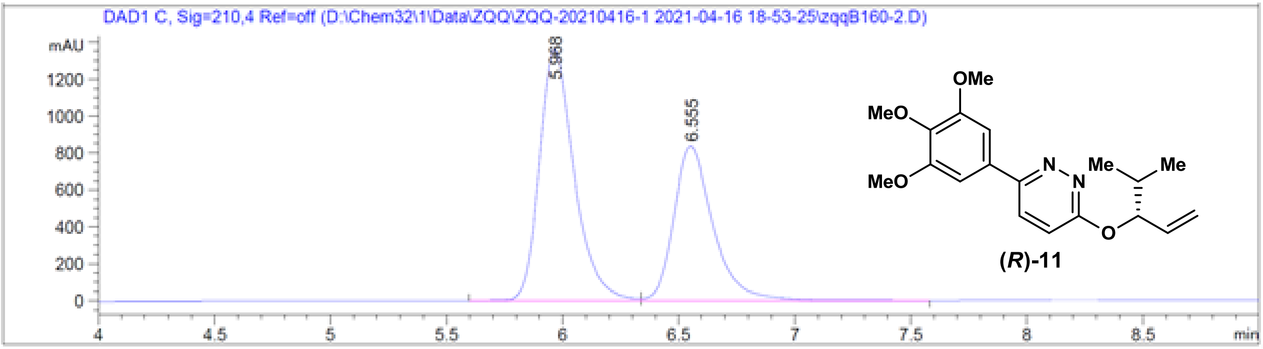

Signal 2: DAD1 C, Sig=210,4 Ref=off

Peak #	RetTime [min]	Type	Width [min]	Area [mAU*s]	Height [mAU]	Area %
1	5.968	BV	0.1572	1.41673e4	1364.02930	59.1135
2	6.555	VB	0.1768	9798.97363	835.41986	40.8865

Supplementary Figure 44. HPLC chromatogram for ***(R)*-11**.

2.5 Screening and optimization of *O*-substituent in cinchona alkaloids.

Supplementary Table 1. Exploring the nature of the non-covalent π-interaction[a].

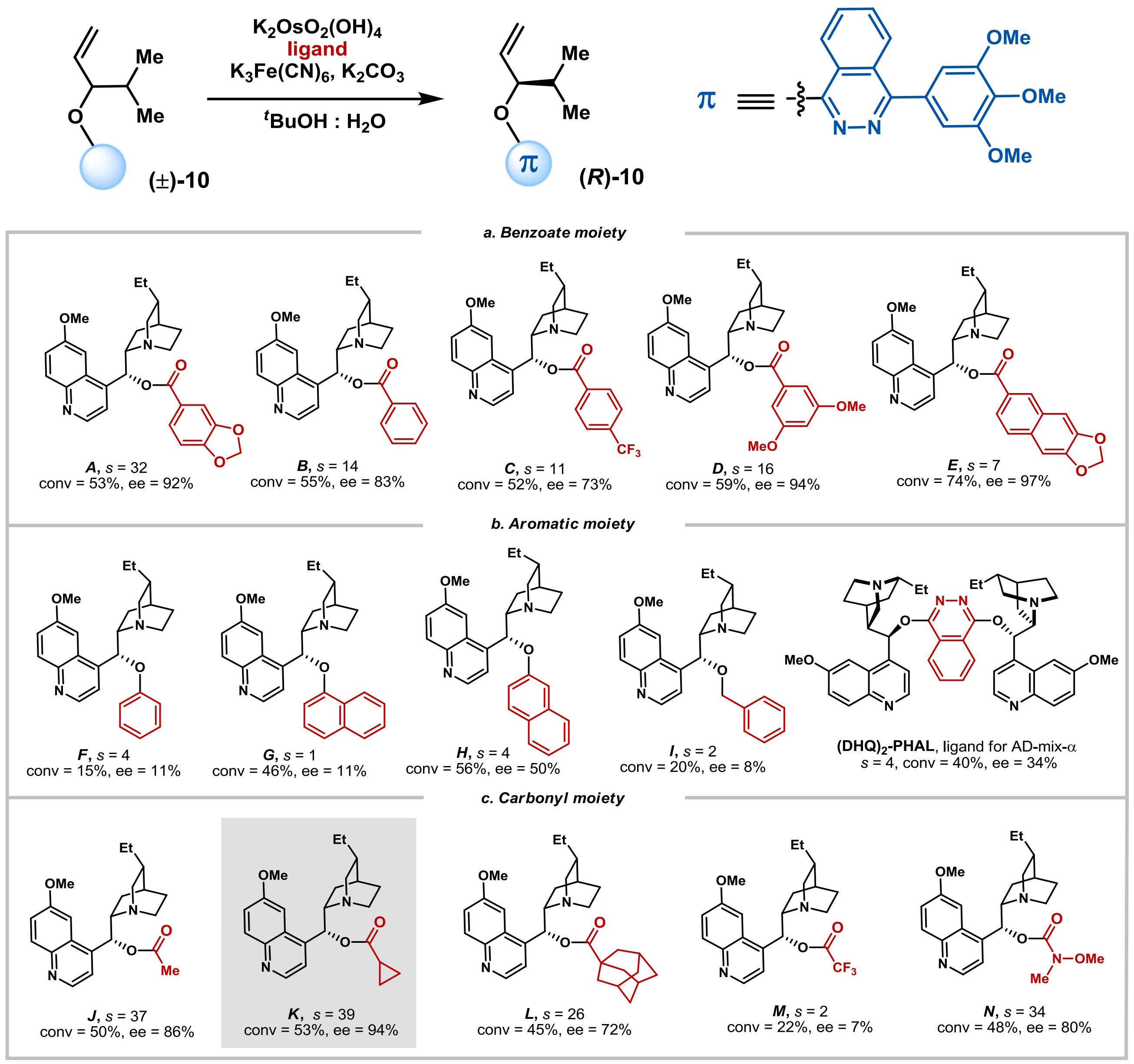

[a] General condition: $K_2OsO_2(OH)_4$ (0.4 mol%), $K_3Fe(CN)_6$ (3.0 equiv), K_2CO_3 (3.0 equiv), cinchona alkaloid ligand (1.0 mol%) and (±)-**10** (0.1 mmol) in 1.0 mL ^tBuOH-H_2O (v/v = 1:1), 0 °C. Conversion was determined by ^1H NMR analysis of the crude mixture.[b] The ee values were determined by HPLC. [c] s = In[(1 – c)(1 – ee)] / In[(1 – c)(1 – ee)].

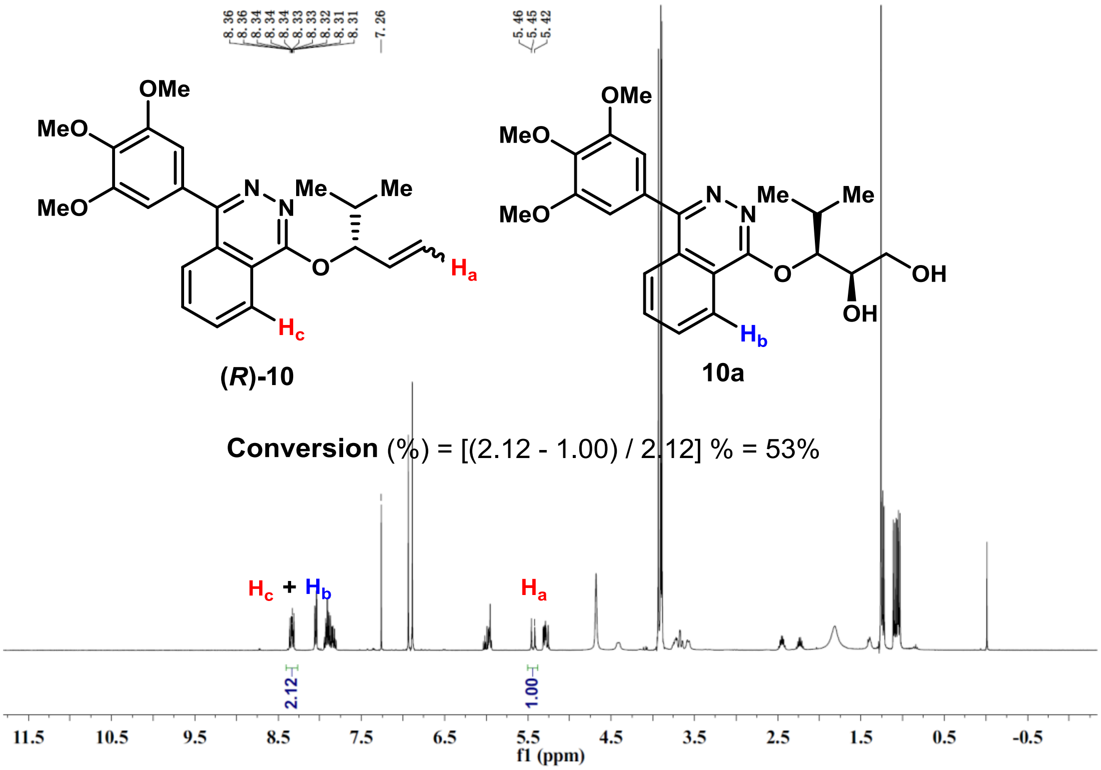

Supplementary Figure 45. ^{1}H NMR spectrum of crude mixture with ligand **A**.

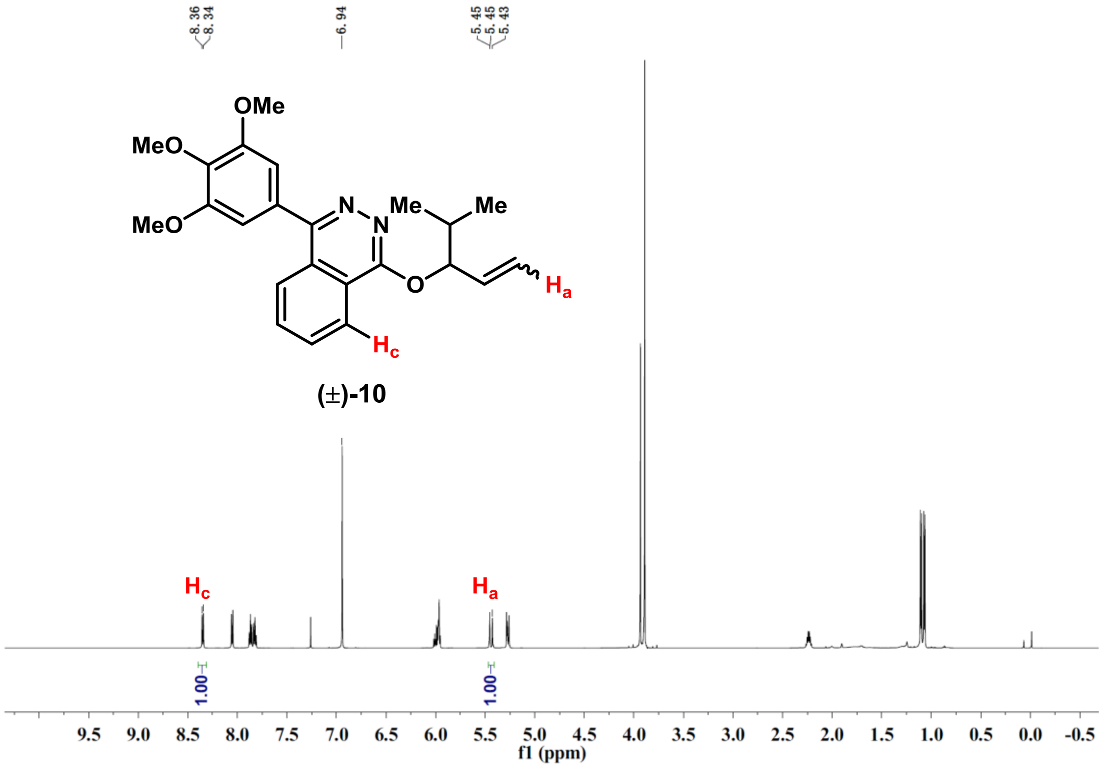

Supplementary Figure 46. ^{1}H NMR spectrum of starting material **(±)-10**.

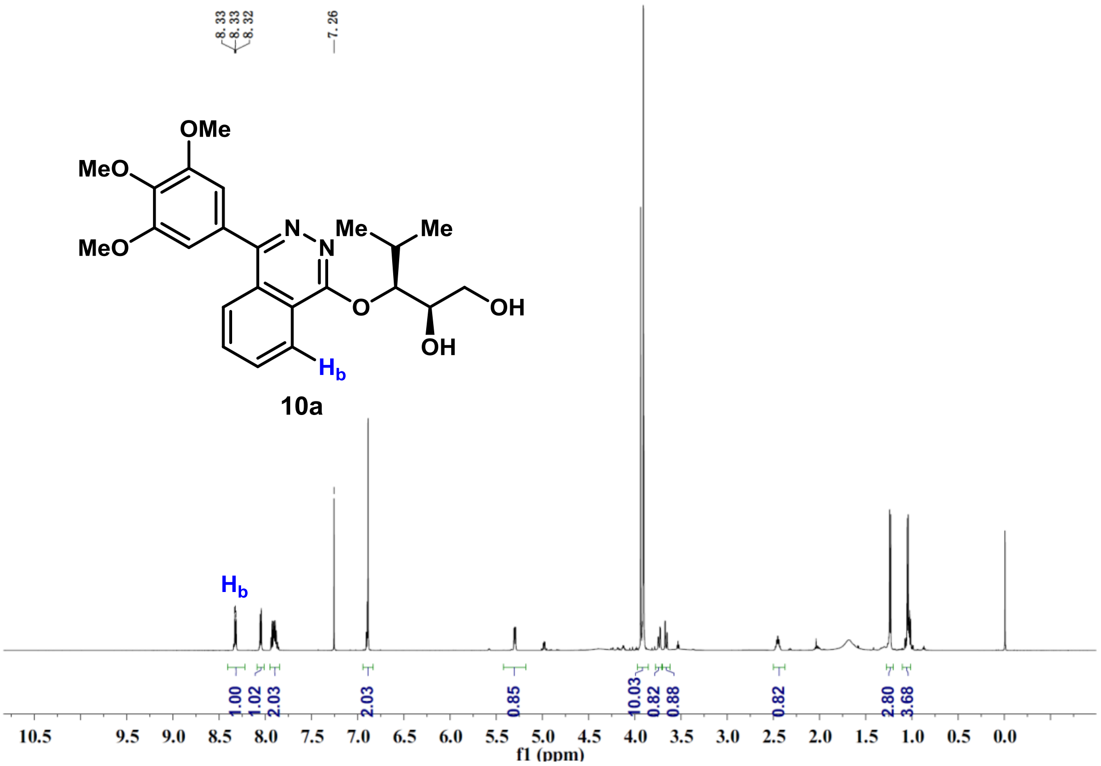

Supplementary Figure 47. ^{1}H NMR spectrum of dihydroxylated product **10a**.

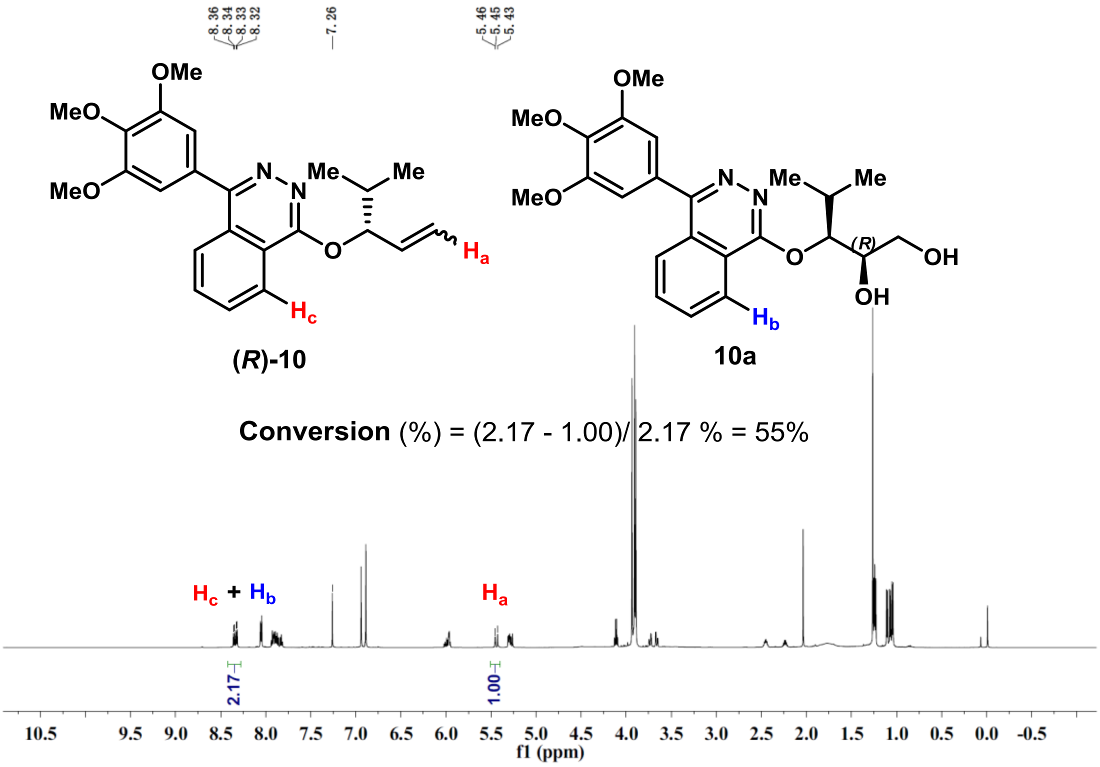

Supplementary Figure 48. ^{1}H NMR spectrum of crude mixture with ligand **B**.

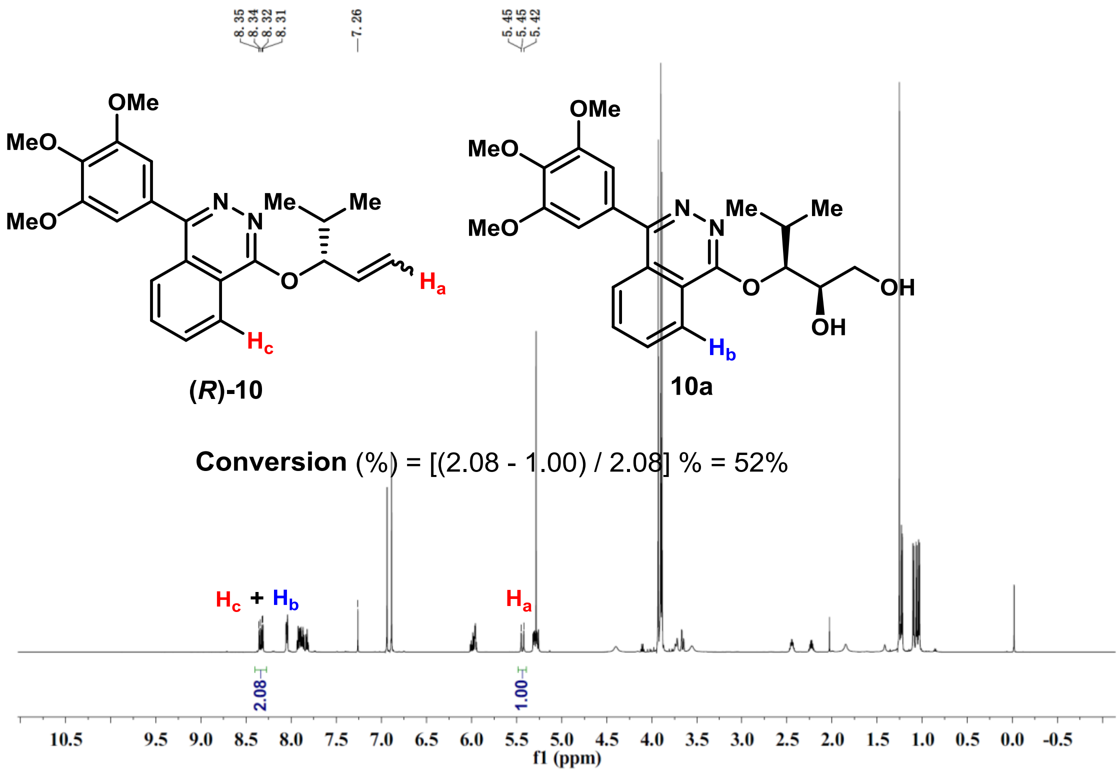

Supplementary Figure 49. ^{1}H NMR spectrum of crude mixture with ligand **C**.

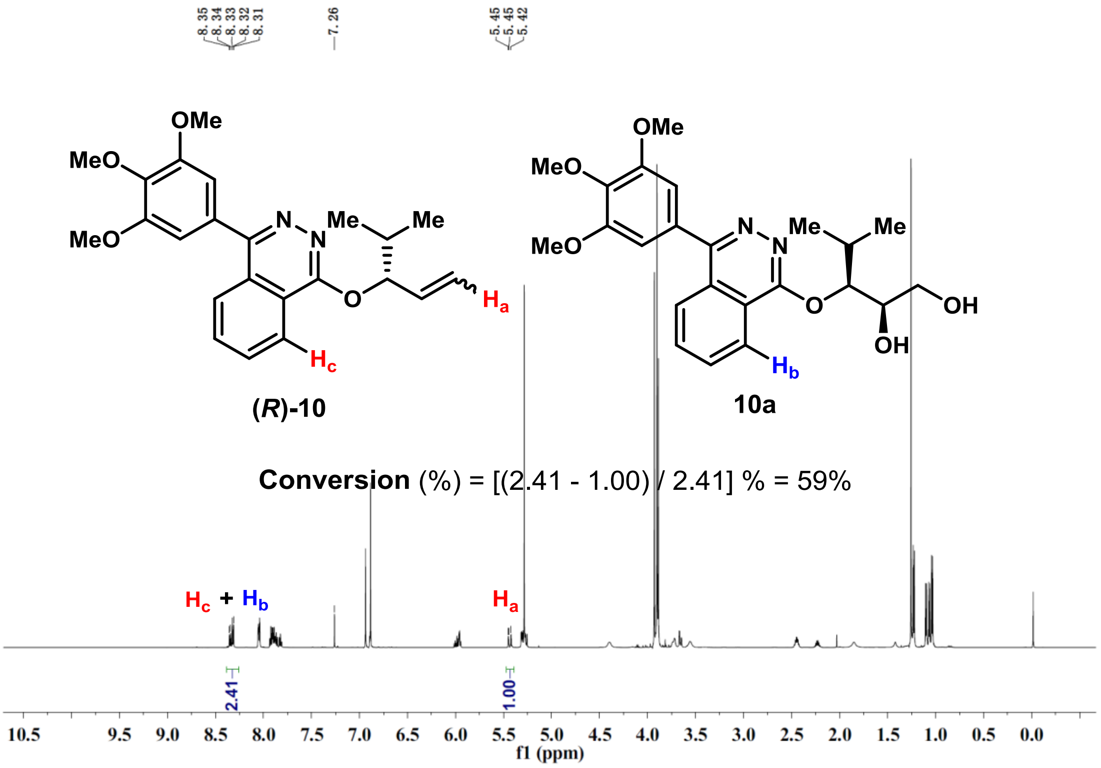

Supplementary Figure 50. ^{1}H NMR spectrum of crude mixture with ligand **D**.

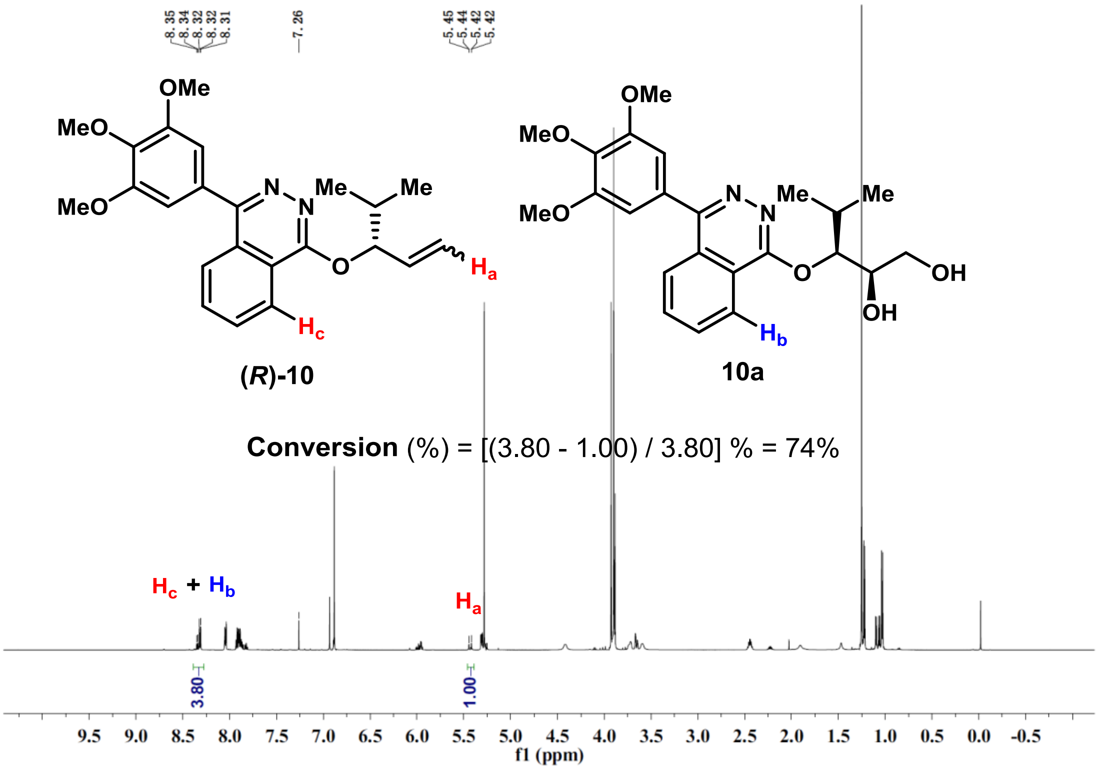

Supplementary Figure 51. ^{1}H NMR spectrum of crude mixture with ligand **E**.

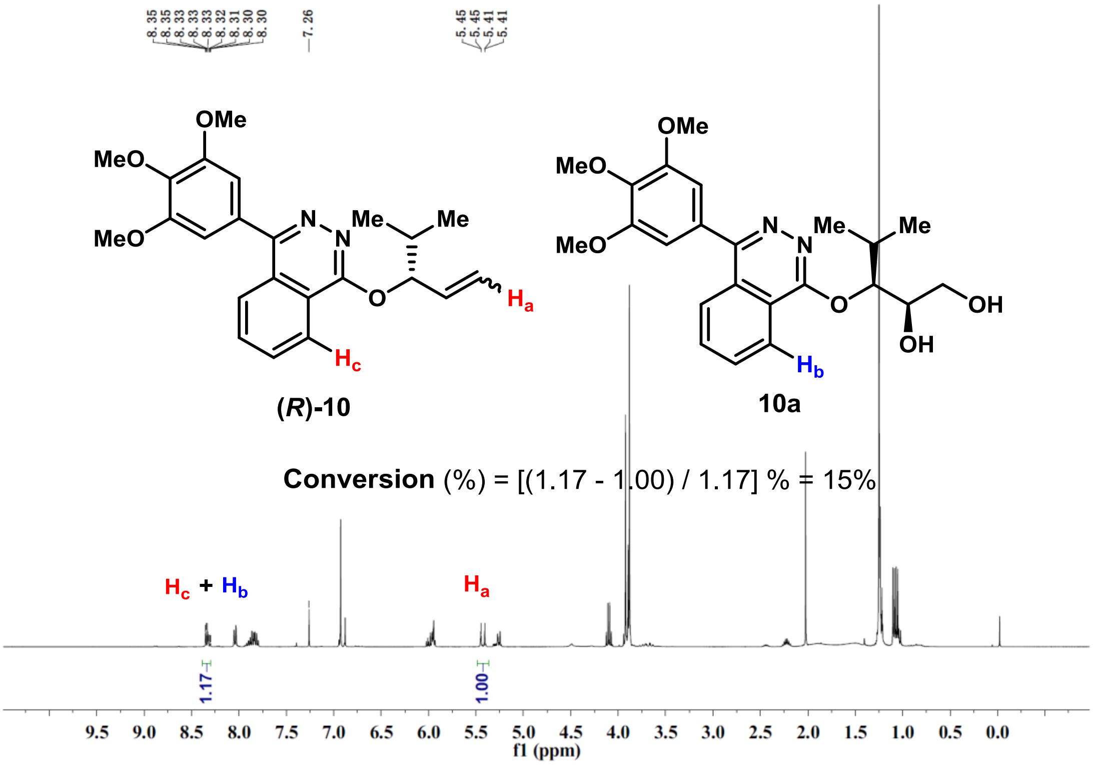

Supplementary Figure 52. ^{1}H NMR spectrum of crude mixture with ligand **F**.

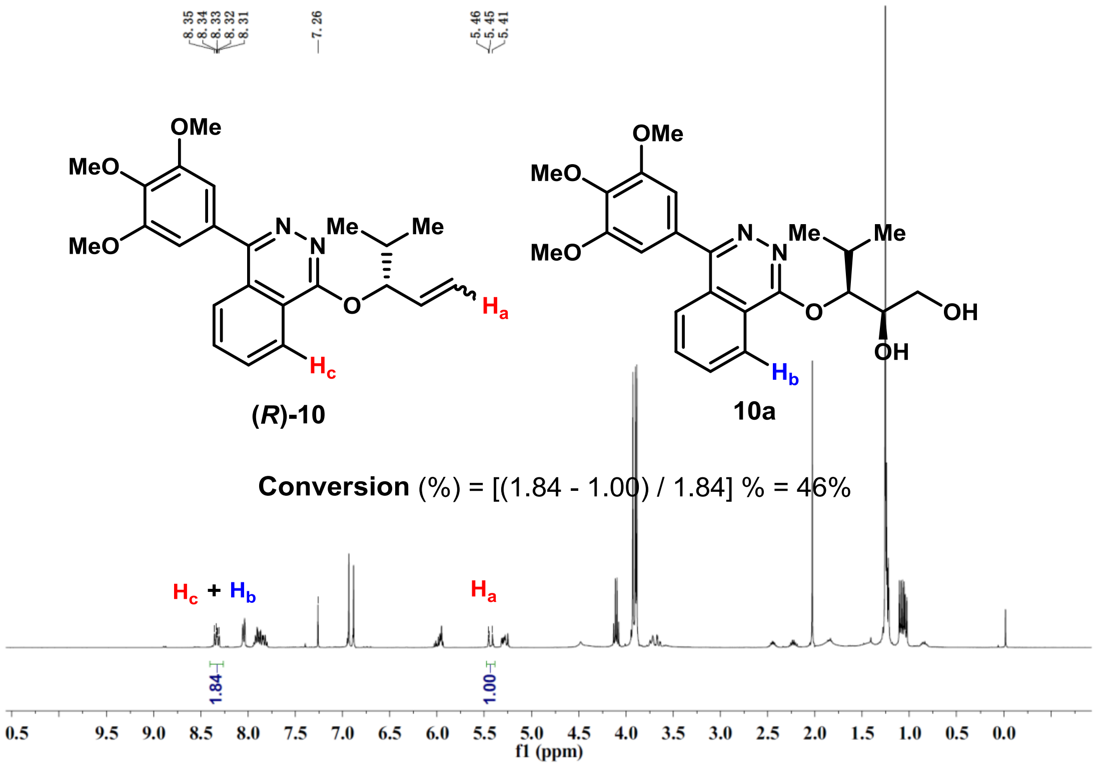

Supplementary Figure 53. ^{1}H NMR spectrum of crude mixture with ligand **G**.

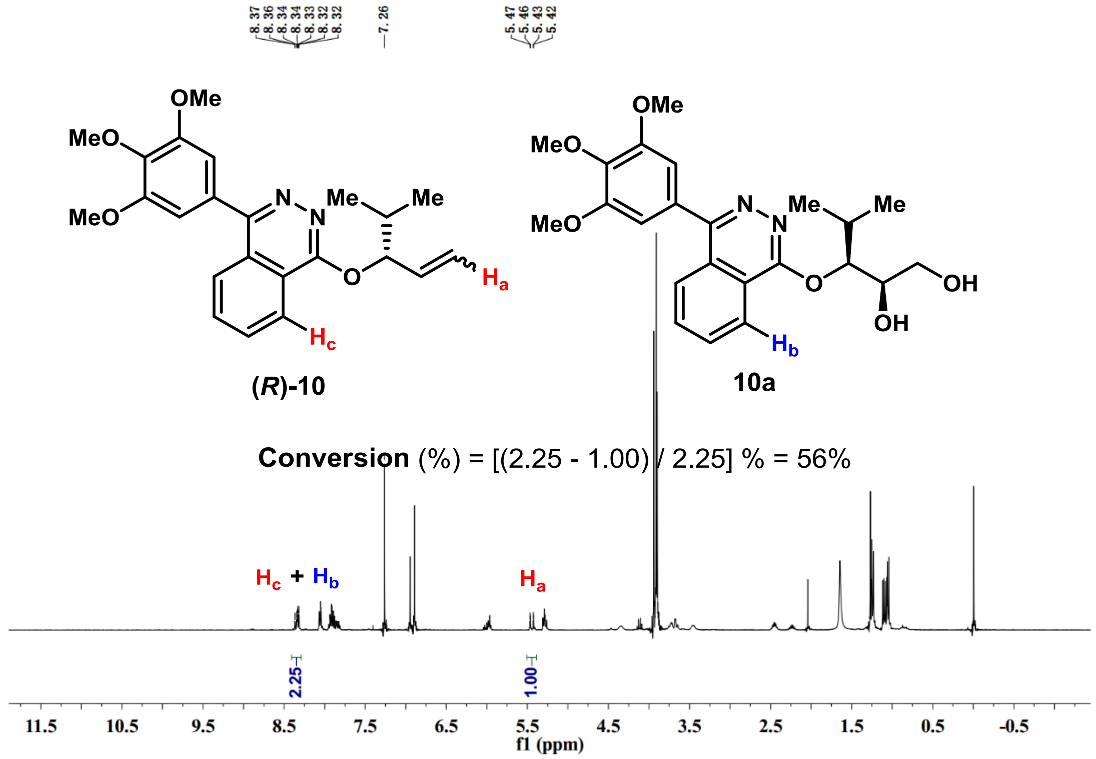

Supplementary Figure 54. ^{1}H NMR spectrum of crude mixture with ligand **H**.

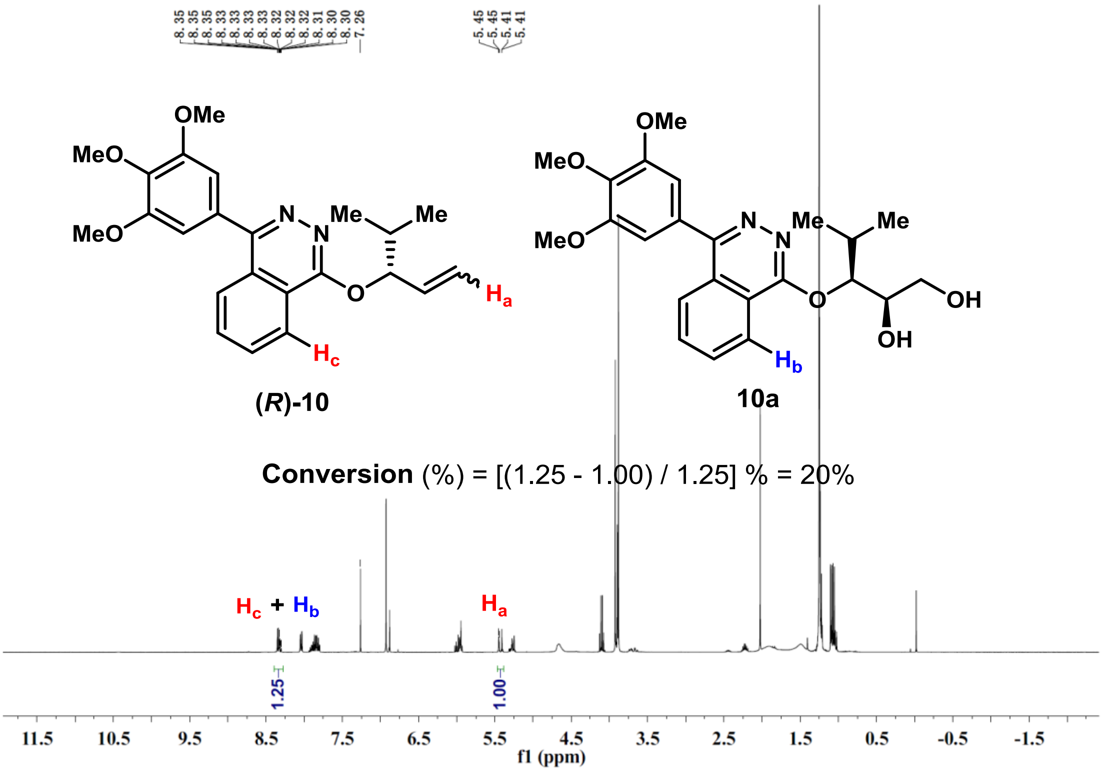

Supplementary Figure 55. ^{1}H NMR spectrum of crude mixture with ligand **I**.

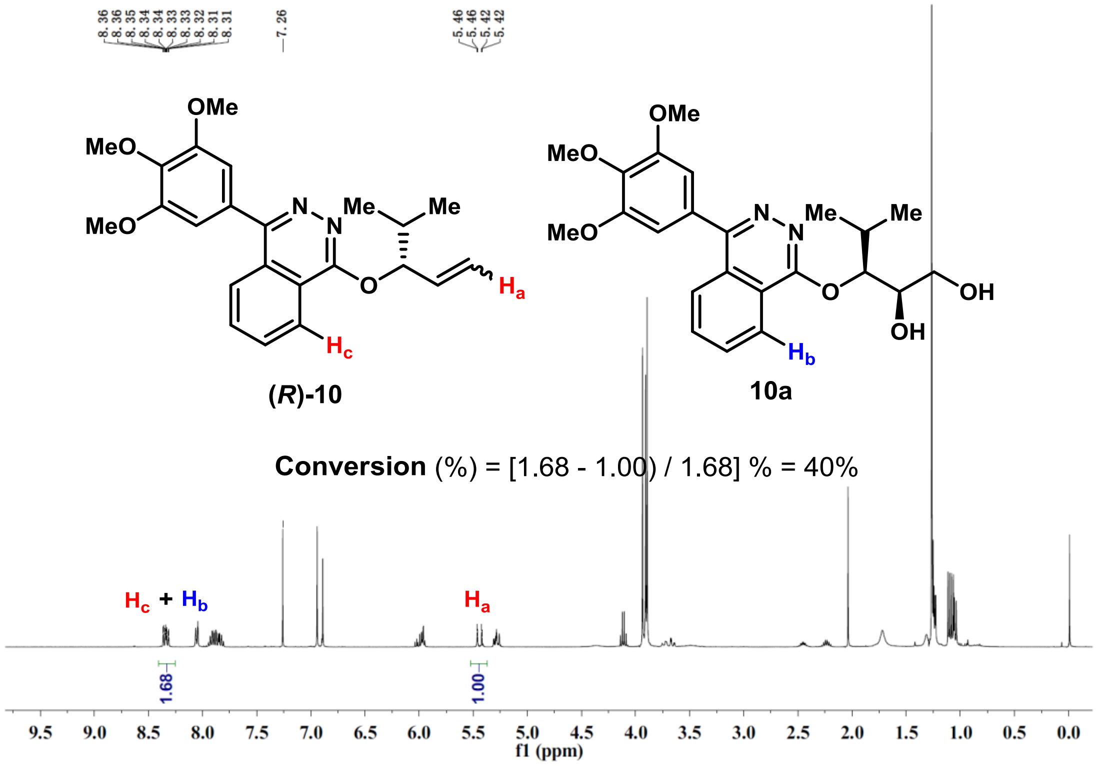

Supplementary Figure 56. ^{1}H NMR spectrum of crude mixture with ligand **(DHQ)$_2$-PHAL**.

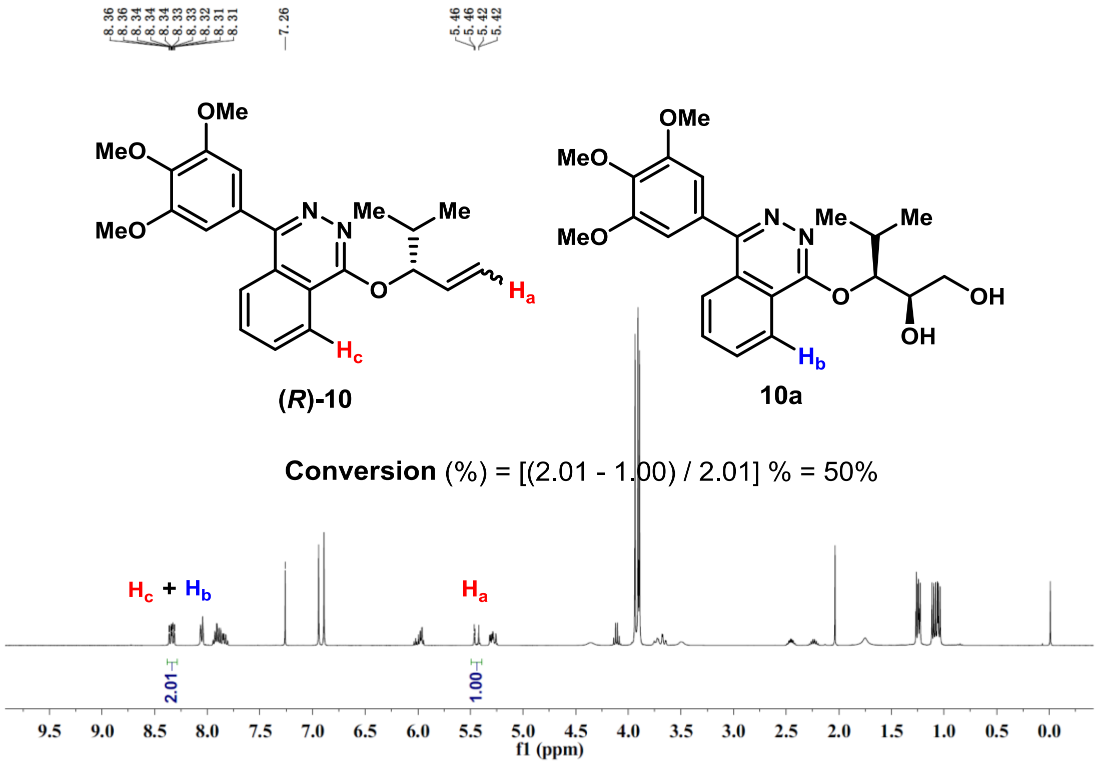

Supplementary Figure 57. ^{1}H NMR spectrum of crude mixture with ligand **J**.

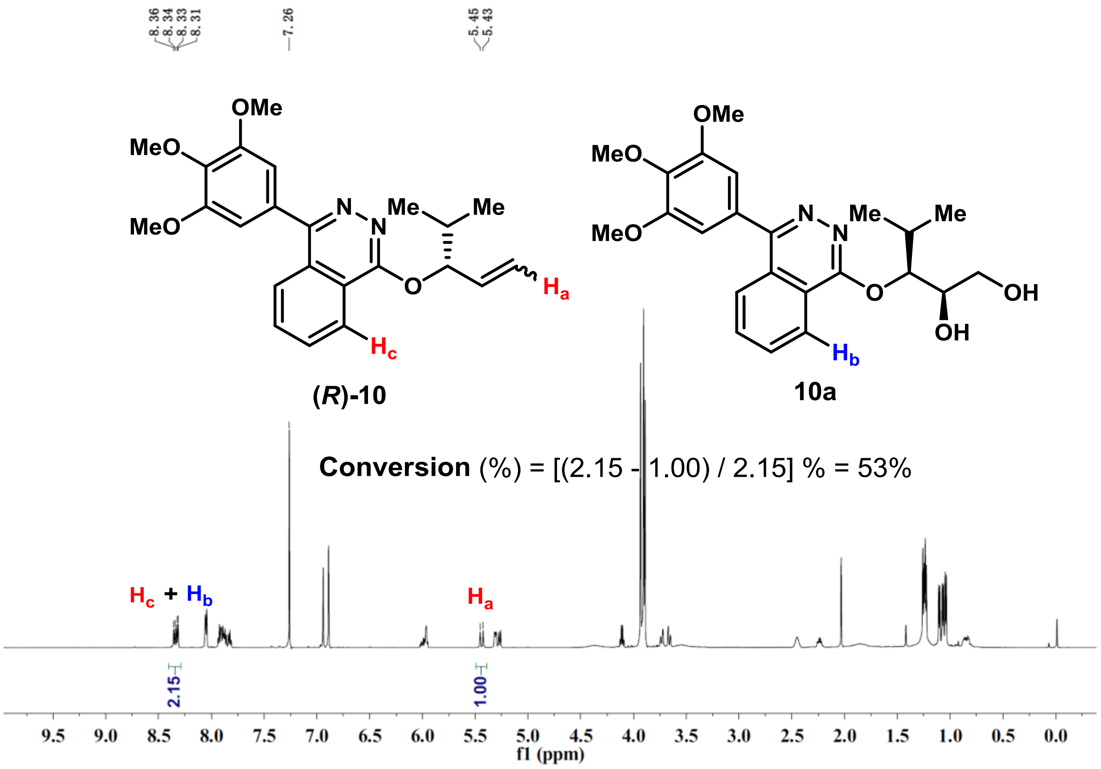

Supplementary Figure 58. ^{1}H NMR spectrum of crude mixture with ligand **K**.

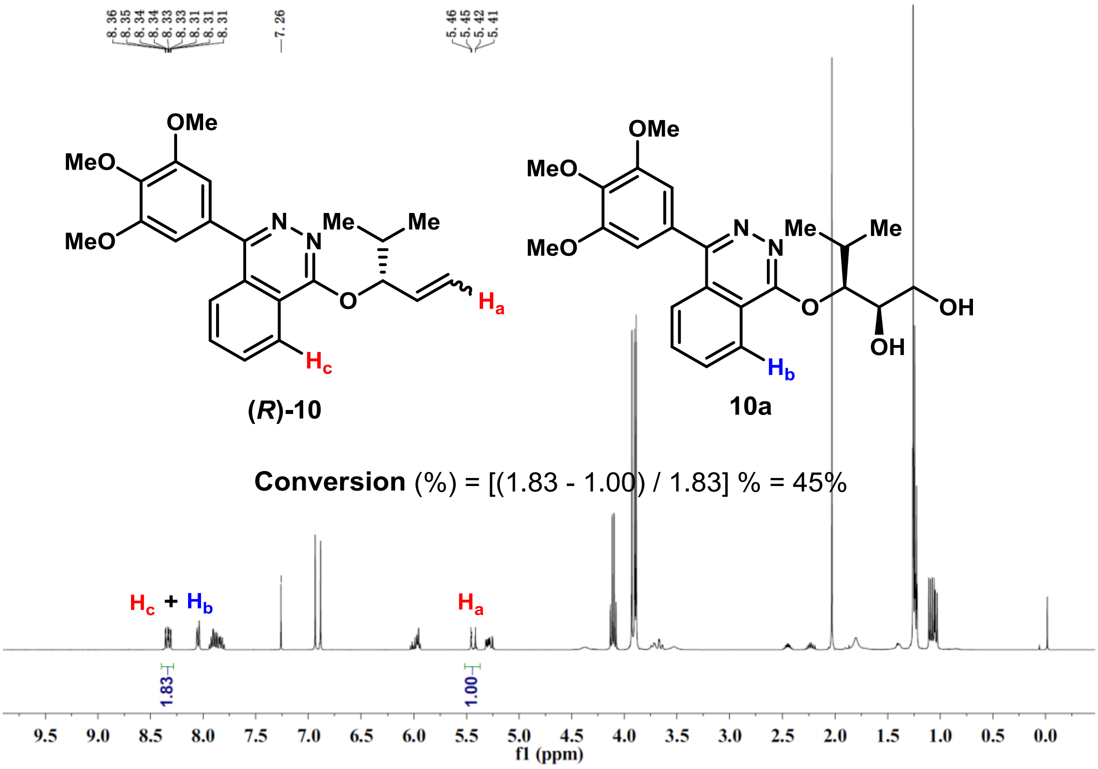

Supplementary Figure 59. ^{1}H NMR spectrum of crude mixture with ligand **L**.

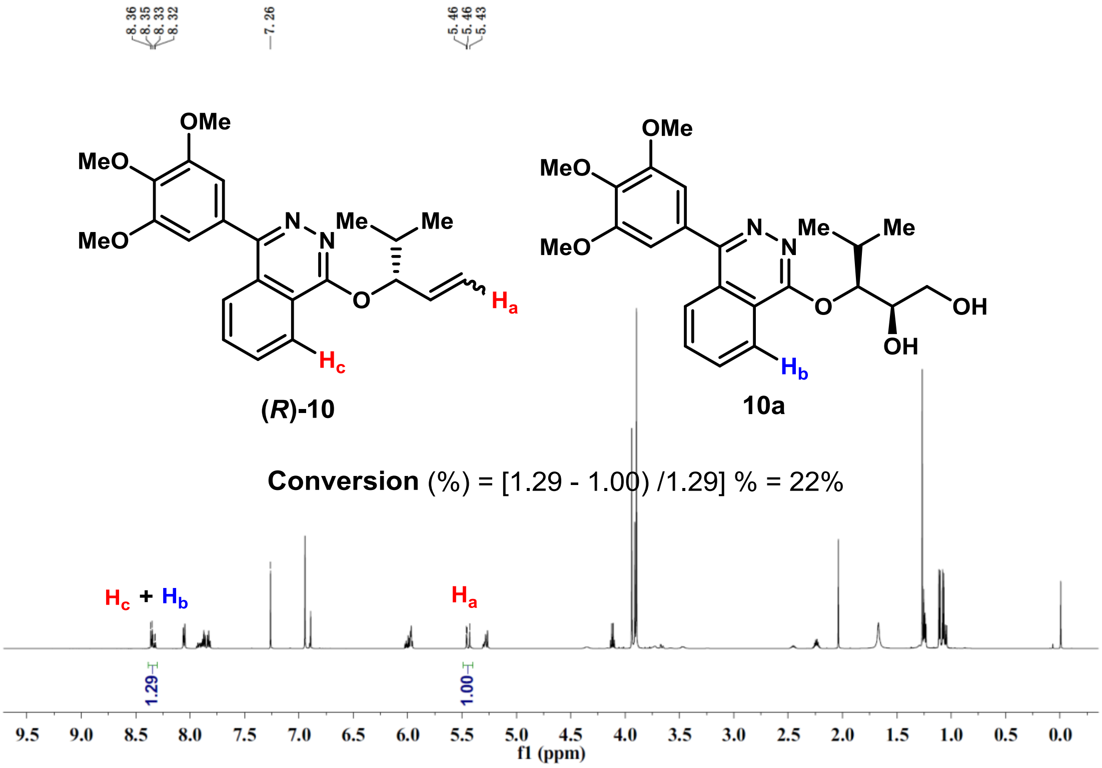

Supplementary Figure 60. ^{1}H NMR spectrum of crude mixture with ligand **M**.

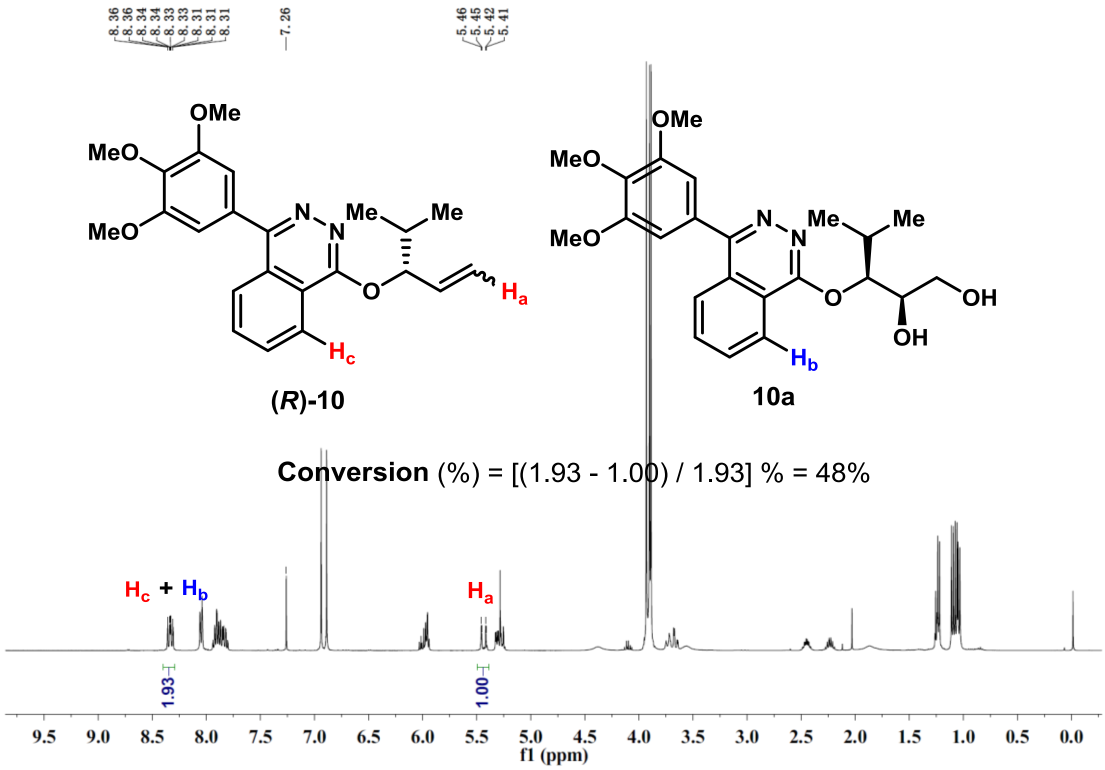

Supplementary Figure 61. ^{1}H NMR spectrum of crude mixture with ligand **N**.

HPLC (AD-H, 0.46*25 cm, 5μm, hexane/isopropanol = 90/10 or 80/20, flow = 1.0 mL/min, detection at 210 nm), retention time = 14.910 min (major) and 19.100 min (minor) or 6.834 min (major) and 8.078 min (minor).

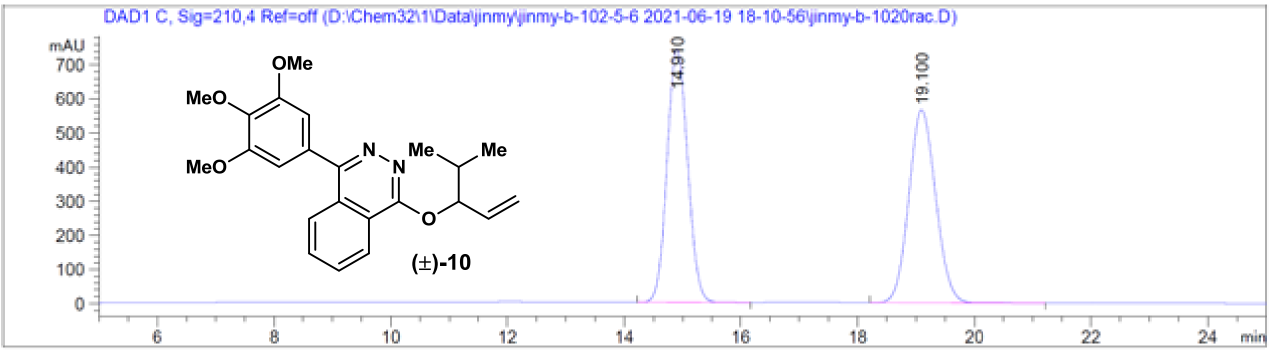

Signal 2: DAD1 C, Sig=210,4 Ref=off

Peak #	RetTime [min]	Type	Width [min]	Area [mAU*s]	Height [mAU]	Area %
1	14.910	BB	0.3837	1.81943e4	742.90076	49.9093
2	19.100	BB	0.5050	1.82604e4	565.13336	50.0907

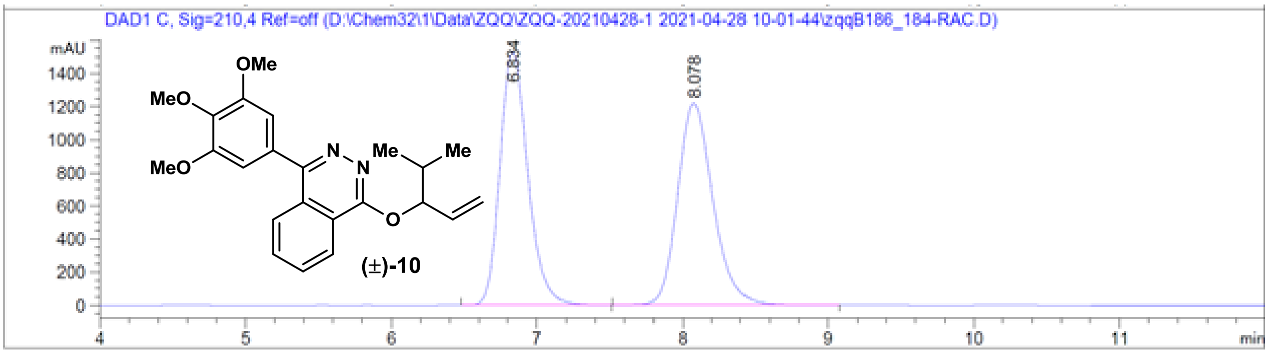

Signal 2: DAD1 C, Sig=210,4 Ref=off

Peak #	RetTime [min]	Type	Width [min]	Area [mAU*s]	Height [mAU]	Area %
1	6.834	BB	0.2062	2.05013e4	1530.77563	49.6386
2	8.078	BB	0.2610	2.07998e4	1219.70654	50.3614

Supplementary Figure 62. HPLC chromatogram for **(±)-10**.

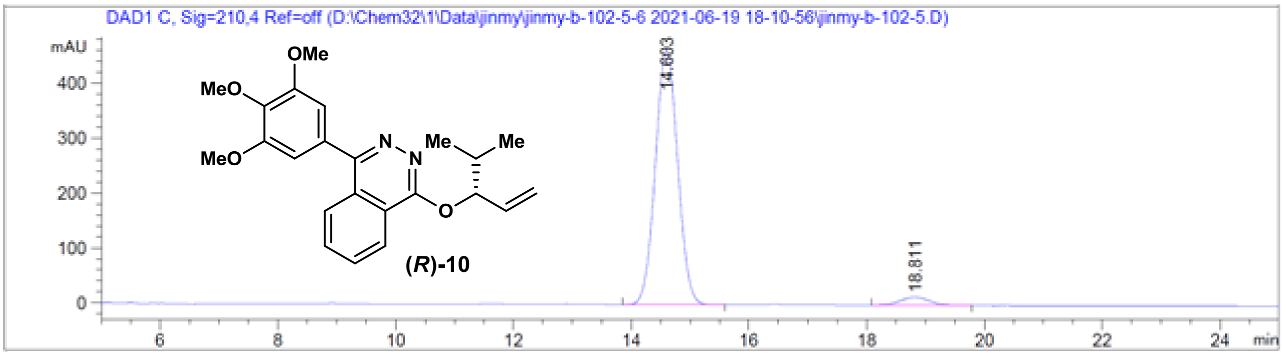

Signal 2: DAD1 C, Sig=210,4 Ref=off

Peak #	RetTime [min]	Type	Width [min]	Area [mAU*s]	Height [mAU]	Area %
1	14.603	BB	0.4126	1.22245e4	465.23315	96.0266
2	18.811	BB	0.5188	505.83209	14.79656	3.9734

Supplementary Figure 63. HPLC chromatogram for **(*R*)-10** with ligand **A**.

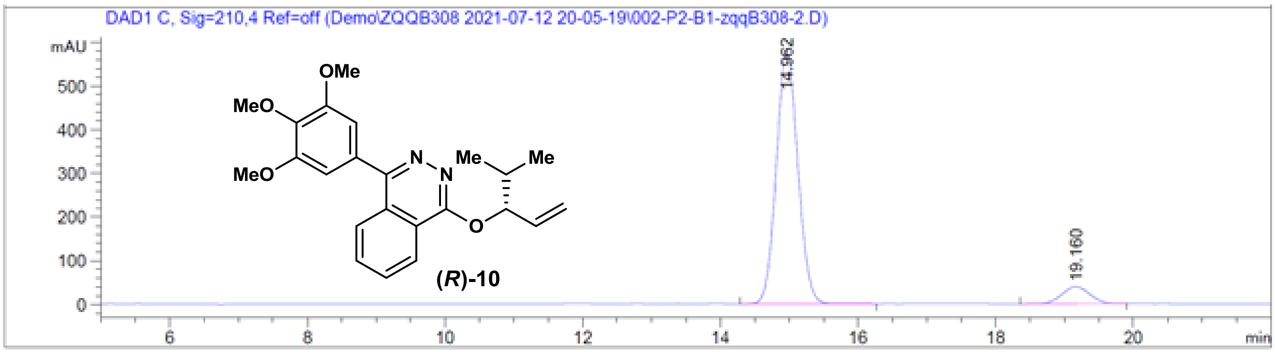

Signal 2: DAD1 C, Sig=210,4 Ref=off

Peak #	RetTime [min]	Type	Width [min]	Area [mAU*s]	Height [mAU]	Area %
1	14.962	BB	0.3411	1.27028e4	581.00037	91.9962
2	19.160	BB	0.4375	1105.16113	39.13092	8.0038

Supplementary Figure 64. HPLC chromatogram for **(*R*)-10** with ligand **B**.

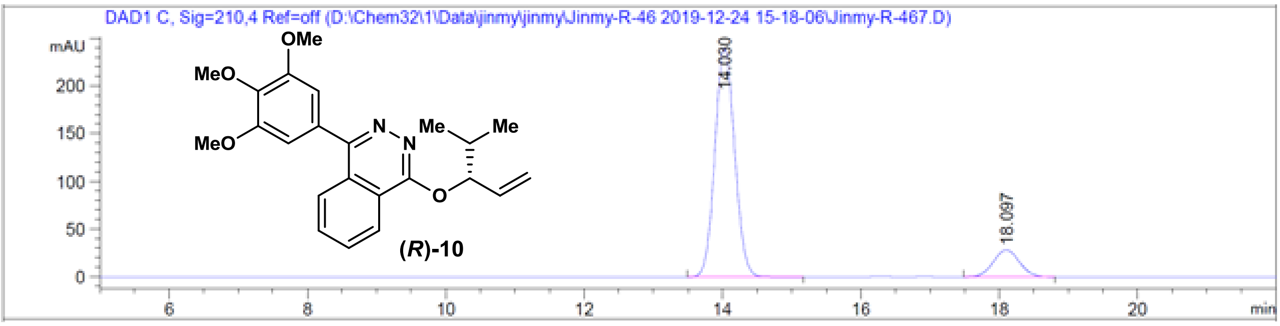

Signal 3: DAD1 C, Sig=210,4 Ref=off

Peak #	RetTime [min]	Type	Width [min]	Area [mAU*s]	Height [mAU]	Area %
1	14.030	BB	0.3106	4772.96436	239.44289	86.6051
2	18.097	BB	0.4158	738.21600	27.98224	13.3949

Supplementary Figure 65. HPLC chromatogram for **(*R*)-10** with ligand **C**.

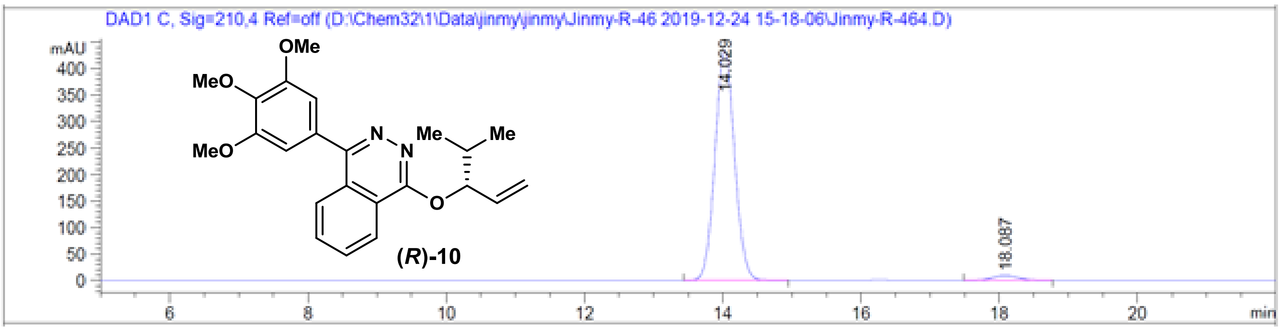

Signal 3: DAD1 C, Sig=210,4 Ref=off

Peak #	RetTime [min]	Type	Width [min]	Area [mAU*s]	Height [mAU]	Area %
1	14.029	BB	0.3091	8619.40332	435.08618	97.1542
2	18.087	BB	0.3937	252.47650	9.57326	2.8458

Supplementary Figure 66. HPLC chromatogram for **(*R*)-10** with ligand **D**.

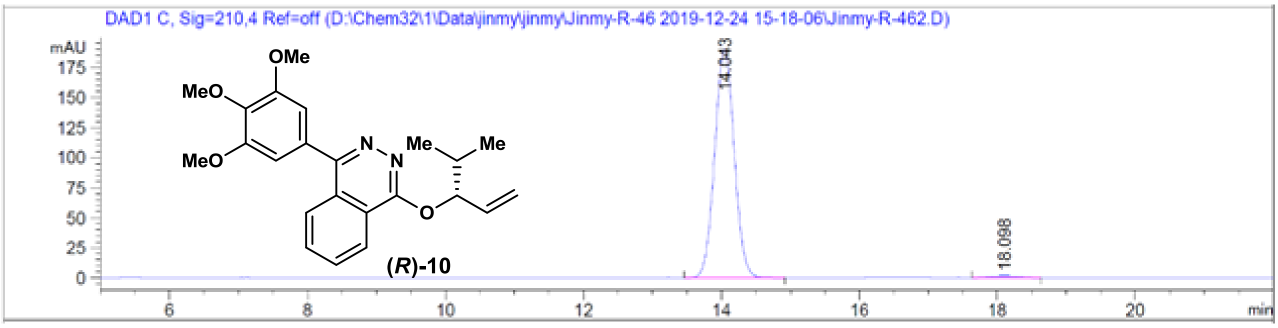

Signal 3: DAD1 C, Sig=210,4 Ref=off

Peak #	RetTime [min]	Type	Width [min]	Area [mAU*s]	Height [mAU]	Area %
1	14.043	BB	0.3091	3766.75488	190.12476	98.6838
2	18.098	MM R	0.4869	50.24034	1.71987	1.3162

Supplementary Figure 67. HPLC chromatogram for **(*R*)-10** with ligand **E**.

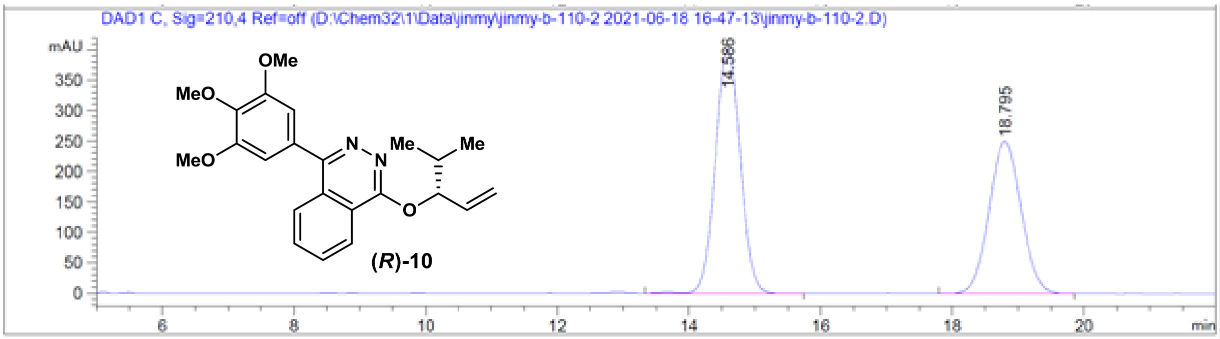

Signal 2: DAD1 C, Sig=210,4 Ref=off

Peak #	RetTime [min]	Type	Width [min]	Area [mAU*s]	Height [mAU]	Area %
1	14.586	VB R	0.4119	1.05183e4	400.29117	55.4633
2	18.795	BB	0.5287	8446.14355	249.62489	44.5367

Supplementary Figure 68. HPLC chromatogram for **(*R*)-10** with ligand **F**.

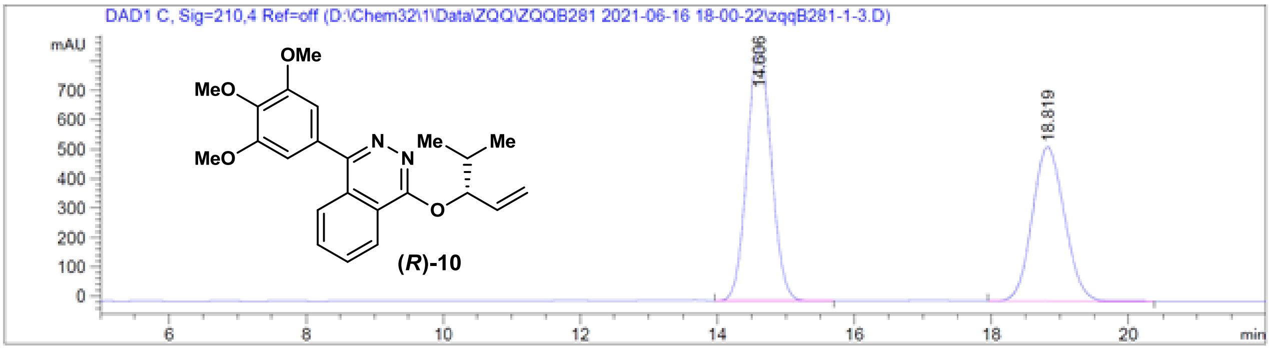

Signal 2: DAD1 C, Sig=210,4 Ref=off

Peak #	RetTime [min]	Type	Width [min]	Area [mAU*s]	Height [mAU]	Area %
1	14.606	BB	0.3824	2.11053e4	859.76343	55.2786
2	18.819	BB	0.5084	1.70746e4	523.67108	44.7214

Supplementary Figure 69. HPLC chromatogram for **(*R*)-10** with ligand **G**.

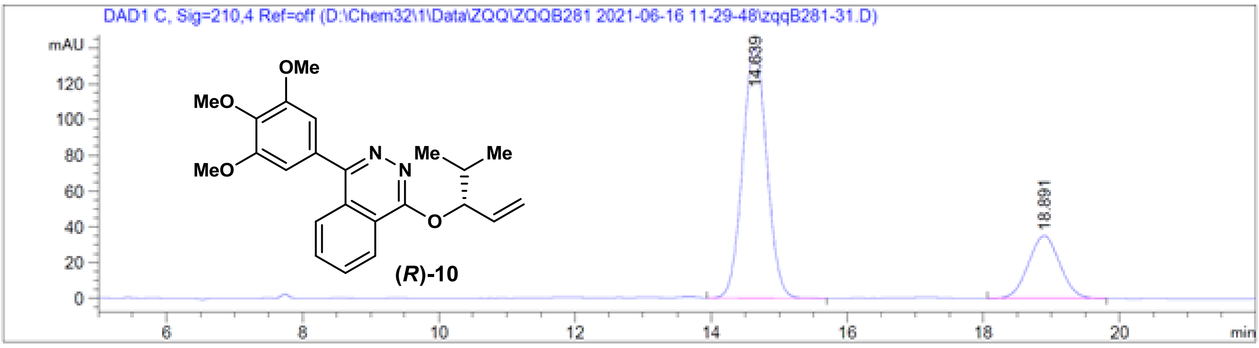

Signal 2: DAD1 C, Sig=210,4 Ref=off

Peak #	RetTime [min]	Type	Width [min]	Area [mAU*s]	Height [mAU]	Area %
1	14.639	BB	0.3818	3433.95361	140.19353	75.0031
2	18.891	BB	0.5043	1144.46362	35.29756	24.9969

Supplementary Figure 70. HPLC chromatogram for **(*R*)-10** with ligand **H**.

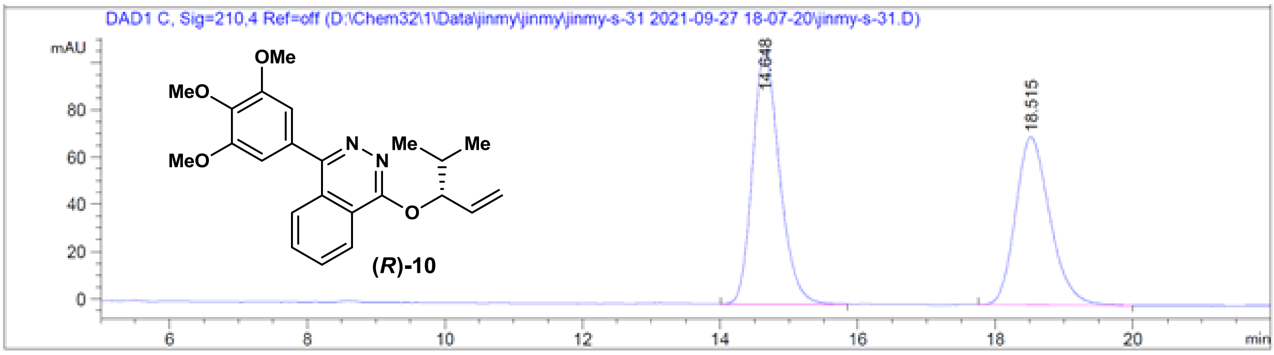

Signal 2: DAD1 C, Sig=210,4 Ref=off

Peak #	RetTime [min]	Type	Width [min]	Area [mAU*s]	Height [mAU]	Area %
1	14.648	BB	0.4172	2916.31006	107.27245	54.1795
2	18.515	BB	0.5371	2466.37280	71.03211	45.8205

Supplementary Figure 71. HPLC chromatogram for **(*R*)-10** with ligand **I**.

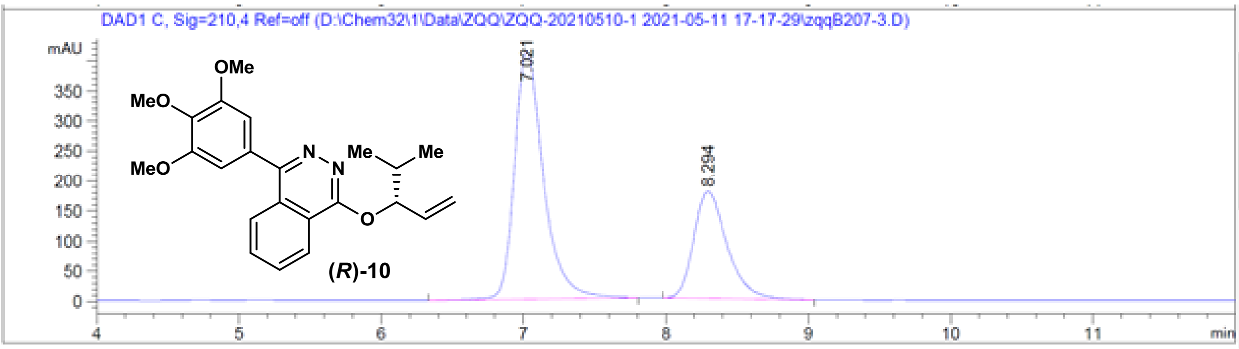

Signal 2: DAD1 C, Sig=210,4 Ref=off

Peak #	RetTime [min]	Type	Width [min]	Area [mAU*s]	Height [mAU]	Area %
1	7.021	BB	0.2130	5835.74268	412.52567	66.9211
2	8.294	BB	0.2474	2884.58813	177.64429	33.0789

Supplementary Figure 72. HPLC chromatogram for **(*R*)-10** with ligand **$(DHQ)_2$-PHAL**.

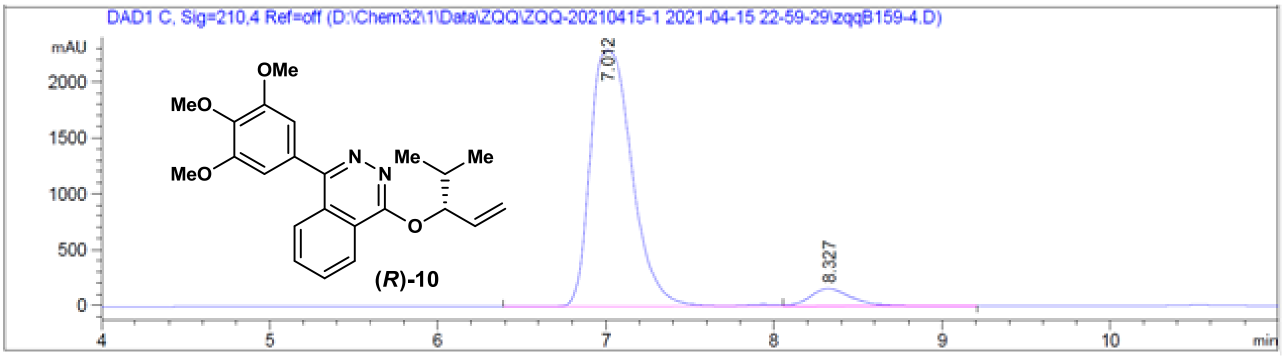

Signal 2: DAD1 C, Sig=210,4 Ref=off

Peak #	RetTime [min]	Type	Width [min]	Area [mAU*s]	Height [mAU]	Area %
1	7.012	BV R	0.2706	3.96641e4	2293.13208	93.6037
2	8.327	VB E	0.2634	2710.39722	155.48566	6.3963

Supplementary Figure 73. HPLC chromatogram for **(*R*)-10** with ligand **J**.

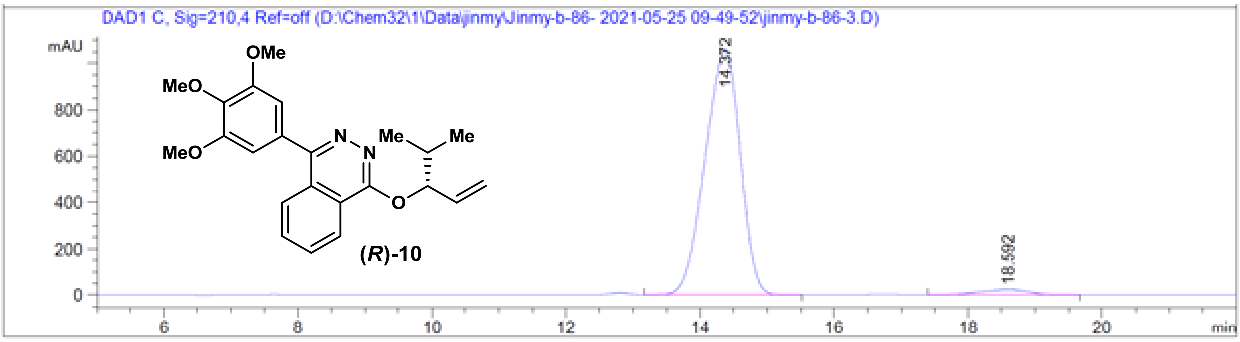

Signal 2: DAD1 C, Sig=210,4 Ref=off

Peak #	RetTime [min]	Type	Width [min]	Area [mAU*s]	Height [mAU]	Area %
1	14.372	BB	0.5925	3.91626e4	1061.37207	97.0946
2	18.592	BB	0.6997	1171.89673	22.95232	2.9054

Supplementary Figure 74. HPLC chromatogram for **(*R*)-10** with ligand **K**.

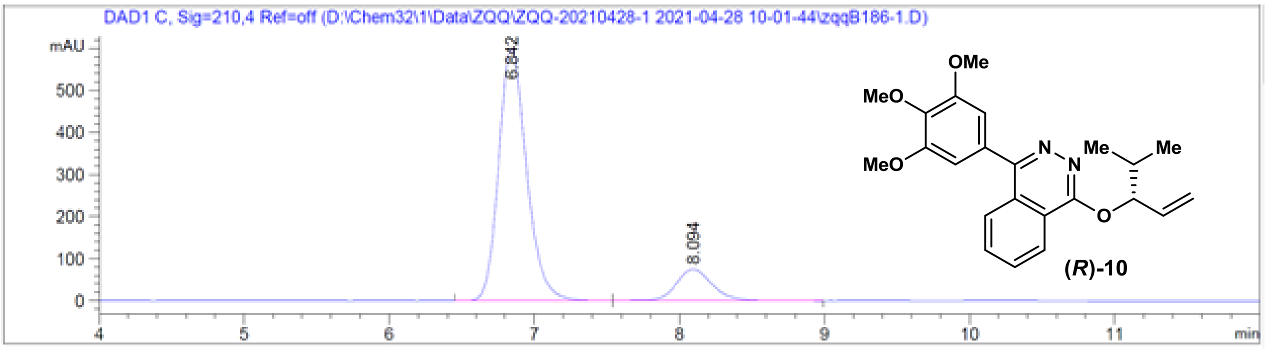

Signal 2: DAD1 C, Sig=210,4 Ref=off

Peak #	RetTime [min]	Type	Width [min]	Area [mAU*s]	Height [mAU]	Area %
1	6.842	BV	0.2041	8021.88818	599.68988	85.9594
2	8.094	VB	0.2649	1310.29370	75.35191	14.0406

Supplementary Figure 75. HPLC chromatogram for **(*R*)-10** with ligand **L**.

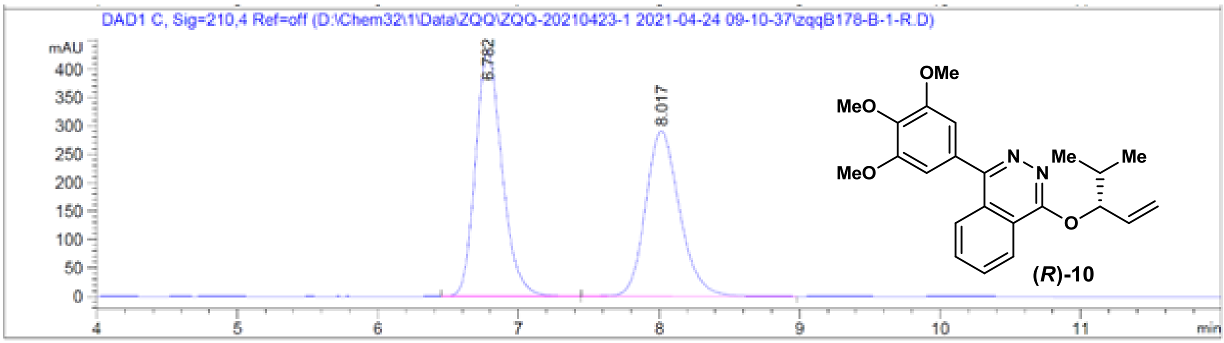

Signal 2: DAD1 C, Sig=210,4 Ref=off

Peak #	RetTime [min]	Type	Width [min]	Area [mAU*s]	Height [mAU]	Area %
1	6.782	BB	0.2008	5664.52344	432.54727	53.6844
2	8.017	BB	0.2581	4887.01074	290.81107	46.3156

Supplementary Figure 76. HPLC chromatogram for **(*R*)-10** with ligand **M**.

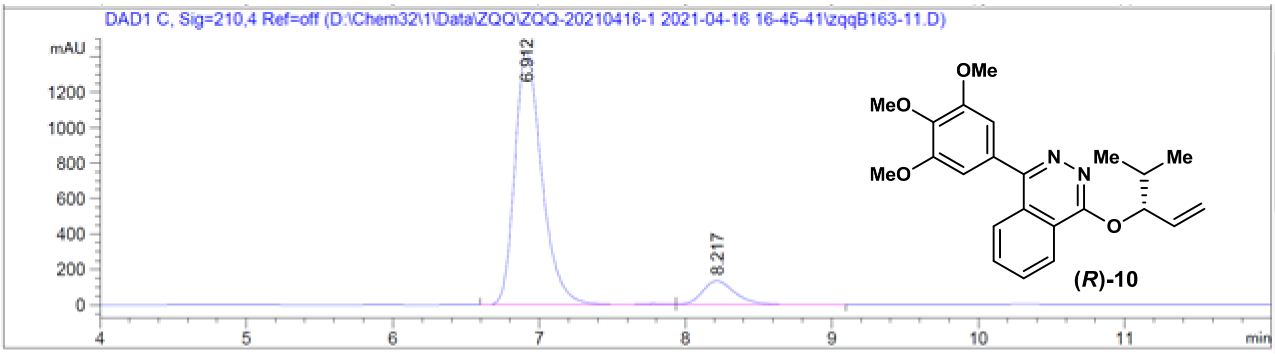


```
Signal 2: DAD1 C, Sig=210,4 Ref=off

Peak RetTime Type  Width     Area       Height      Area
  #   [min]        [min]   [mAU*s]     [mAU]        %
----|-------|----|-------|----------|----------|--------|
   1   6.912 BV R  0.1954 1.84025e4  1429.72888  89.7853
   2   8.217 VB    0.2346 2093.62109  135.22546  10.2147
```

Supplementary Figure 77. HPLC chromatogram for ***(R)*-10** with ligand **N**.

2.6 Substrate scopes.

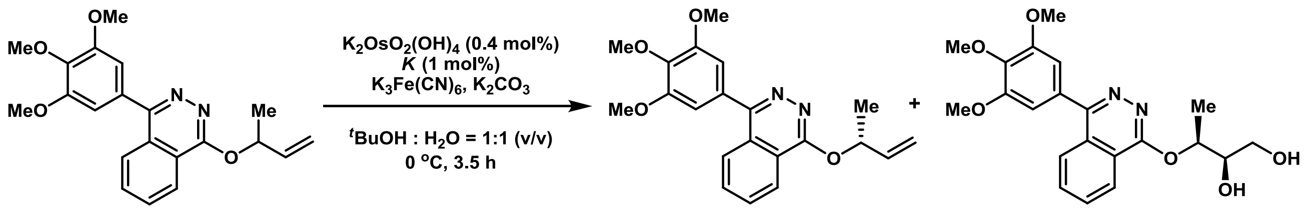

(±)-12 | (*R*)-12 s = 17, conv = 59% ee = 95%, 40% yield | 12a dr = 7:1, 47% ee 56% yield

The general procedure **IV** was followed. The conversion of **(±)-12** was determined by crude ^{1}H NMR. **Conversion** (%) = [(2.43 – 1.00) / 2.43] % = 59%.

S = In [(1 – conv)(1 – ee)] / In [(1 – conv)(1 + ee)] = 17.

The recovered alkene **(*R*)-12** (14.7 mg, 40% yield, 95% ee) was purified by chromatography on silica gel (eluted with petroleum ether : ethyl acetate = 3:1). $[\alpha]_D^{25}$ = –19.84 (*c* 0.63, $CHCl_3$).

The dihydroxylated product **12a** (dr = 7:1, 22.4 mg, 56% yield, 47% ee) was purified by chromatography on silica gel (eluted with petroleum ether : ethyl acetate = 1:2). $[\alpha]_D^{25}$ = – 3.08 (*c* 1.20, $CHCl_3$).

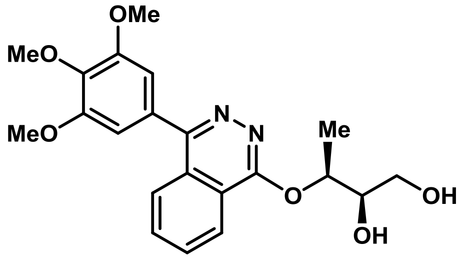

12a

(2*R*,3*S*)-3-(4-(3,4,5-trimethoxyphenyl)phthalazin-1-yloxy)butane-1,2-diol

^{1}H NMR (600 MHz, $CDCl_3$): δ 8.32 (d, *J* = 6.0 Hz, 1H), 8.05 (d, *J* = 12.0 Hz, 1H), 7.94 – 7.85 (m, 2H), 6.90 (s, 2H), 5.63 – 5.54 (m, 1H), 3.94 (s, 3H), 3.91 (s, 6H), 3.85 – 3.82 (m, 1H), 3.81 – 3.76 (m, 1H), 3.74 – 3.68 (m, 1H), 1.64 (d, *J* = 6.0 Hz, 3H) ppm.

^{13}C NMR (151 MHz, $CDCl_3$): δ 160.3, 157.1, 153.4, 139.0, 132.8, 132.1, 131.4, 128.1, 126.4, 123.5, 120.5, 107.2, 75.2, 74.8, 62.3, 61.0, 56.3, 29.7, 17.9 ppm.

HRMS (ESI) m/z: $[M + H]^+$ Calcd for $C_{21}H_{25}N_2O_6$ 401.1707; Found 401.1702.

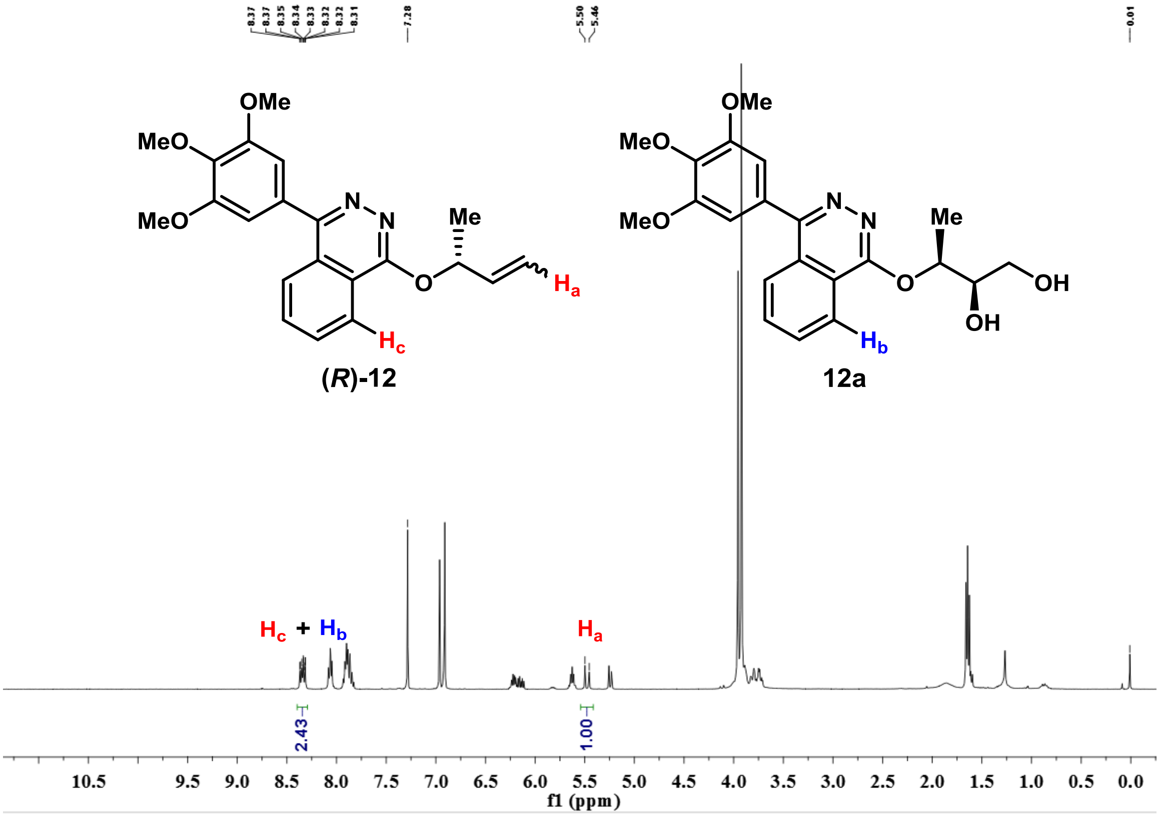

Supplementary Figure 78. ^{1}H NMR spectrum of crude mixture of compound **(*R*)-12** and **12a.**

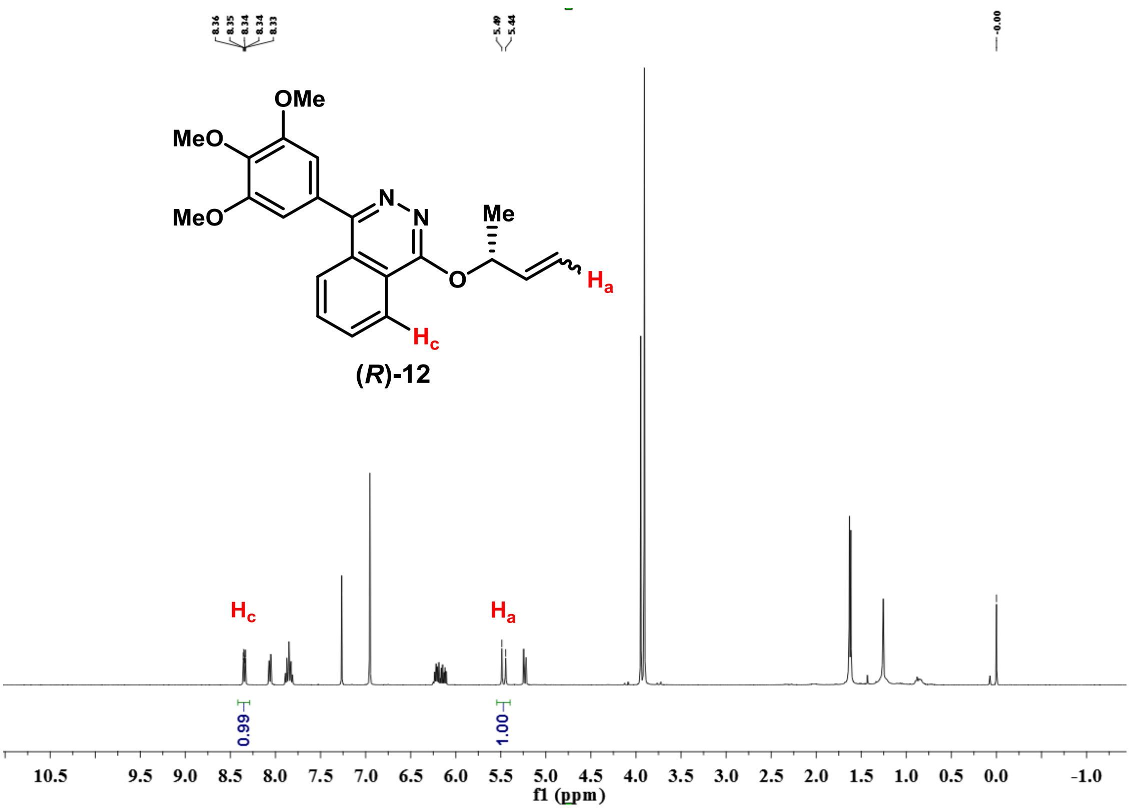

Supplementary Figure 79. ^{1}H NMR spectrum of recovered alkene **(*R*)-12**.

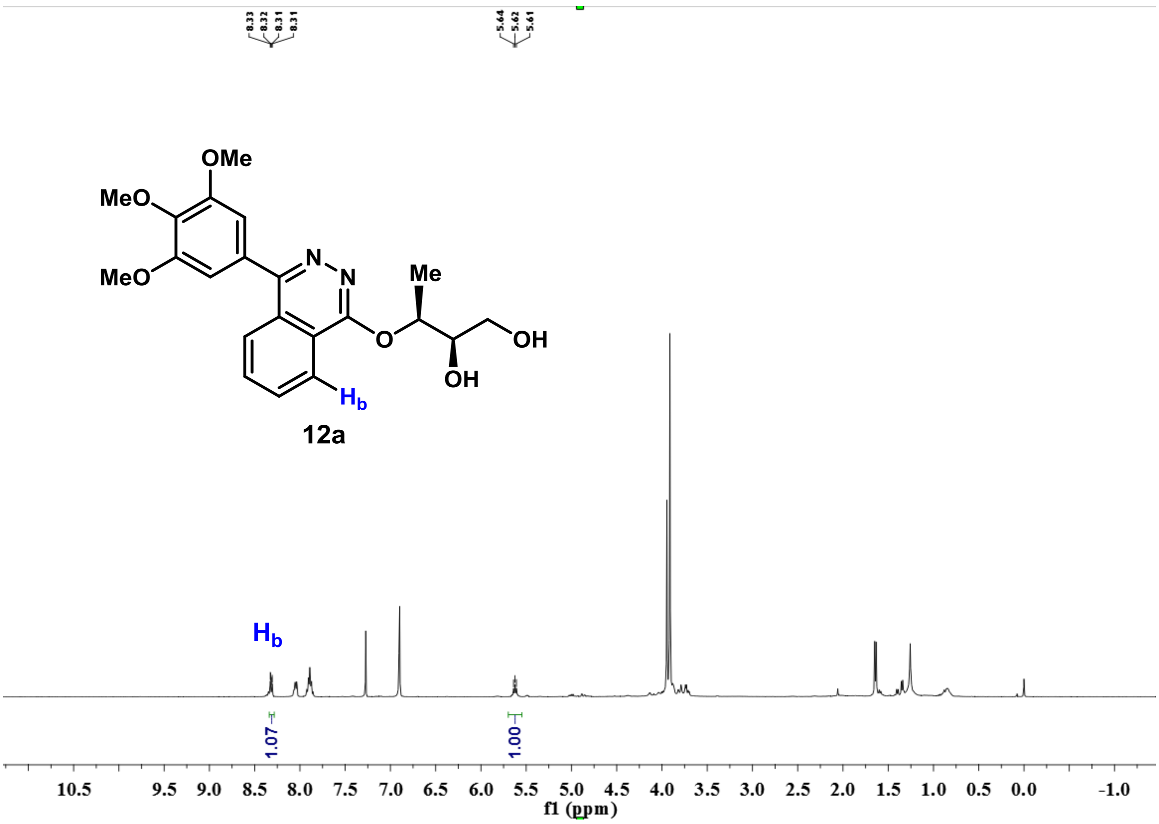

Supplementary Figure 80. ^{1}H NMR spectrum of dihydroxylated product **12a**.

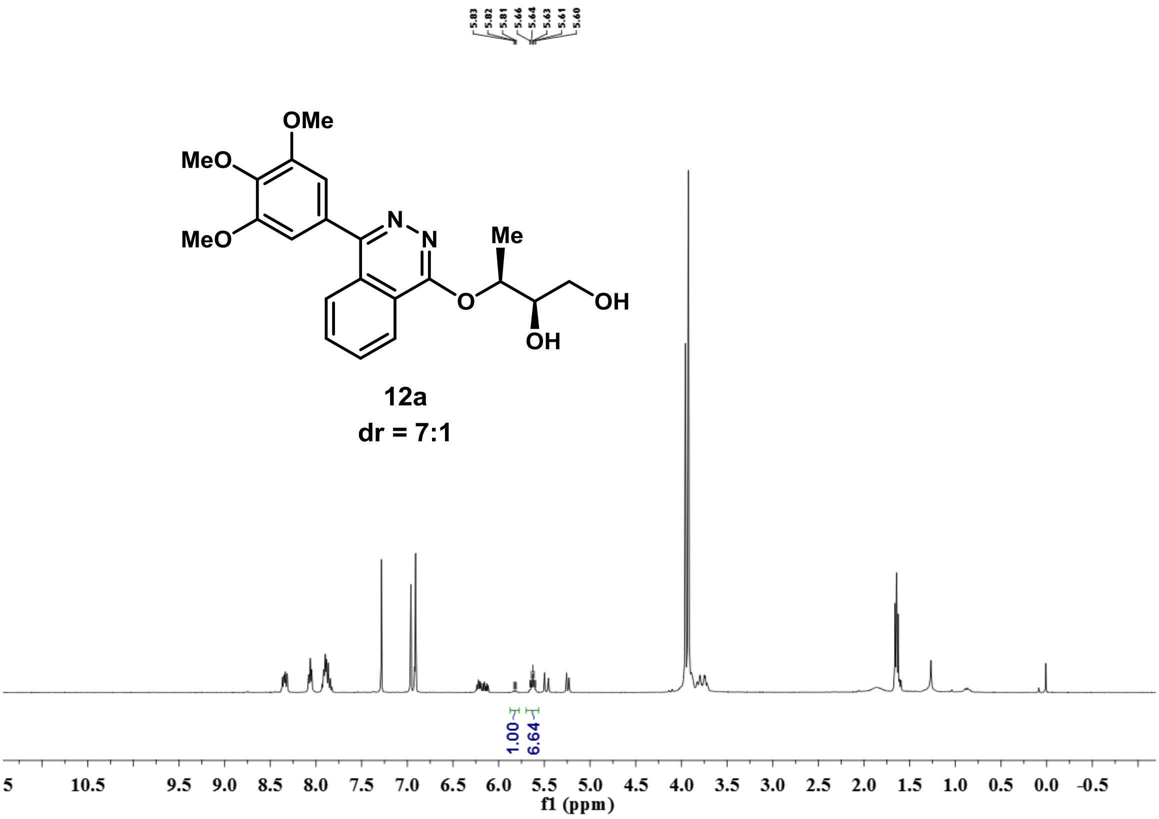

Supplementary Figure 81. ^{1}H NMR spectrum of crude mixture for diastereomeric ratio (dr).

HPLC (AD-H, 0.46*25 cm, 5μm, hexane/isopropanol = 90/10, flow = 1.0 mL/min, detection at 254 nm), retention time = 9.496 min (major) and 11.773 min (minor).

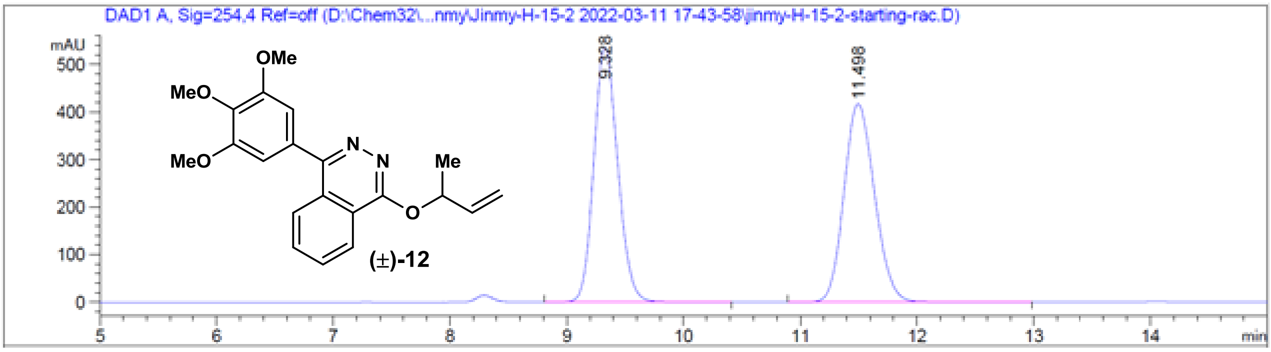

Signal 1: DAD1 A, Sig=254,4 Ref=off

Peak #	RetTime [min]	Type	Width [min]	Area [mAU*s]	Height [mAU]	Area %
1	9.328	BB	0.2219	7598.33398	534.38043	50.0310
2	11.498	BB	0.2822	7588.91846	417.26175	49.9690

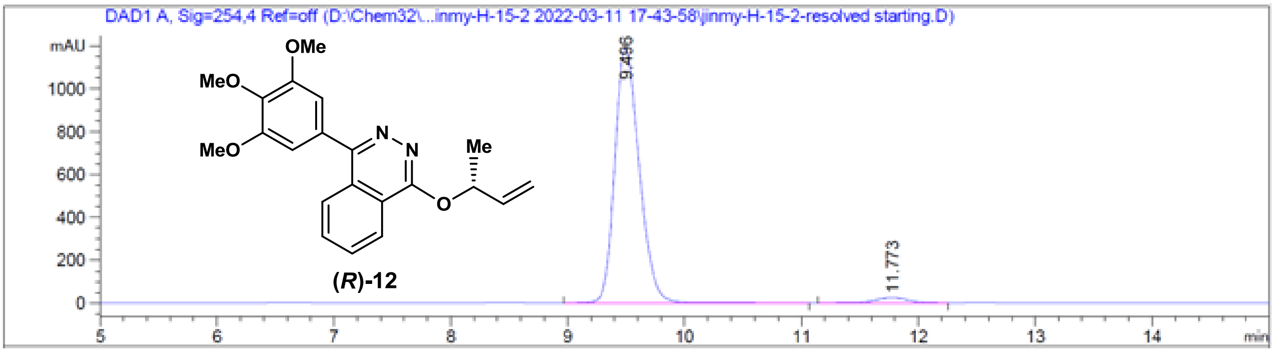

Signal 1: DAD1 A, Sig=254,4 Ref=off

Peak #	RetTime [min]	Type	Width [min]	Area [mAU*s]	Height [mAU]	Area %
1	9.496	BB	0.2261	1.72932e4	1186.43420	97.3931
2	11.773	BB	0.2880	462.88434	24.76650	2.6069

Supplementary Figure 82. HPLC chromatogram for ***(R)*-12**.

HPLC (AD-H, 0.46*25 cm, 5μm, hexane/isopropanol = 85/15, flow = 1.0 mL/min, detection at 210 nm), retention time = 29.092 min (minor) and 31.143 min (major).

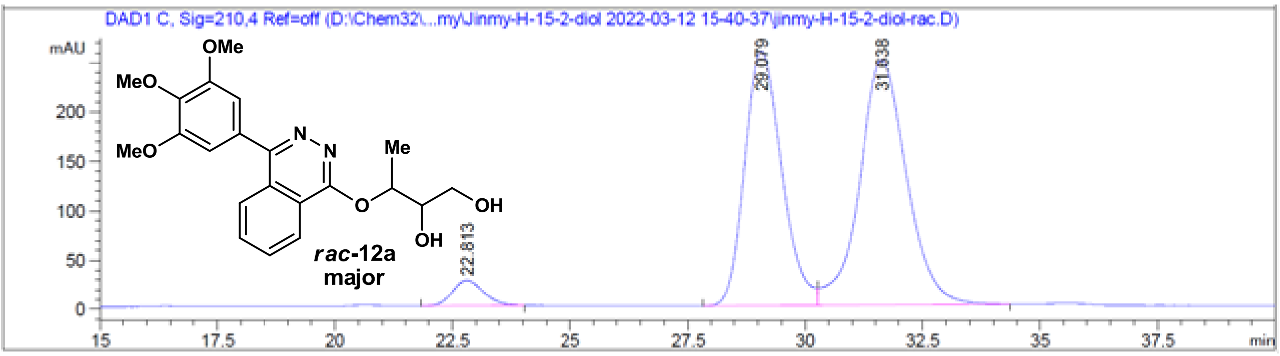

Signal 2: DAD1 C, Sig=210,4 Ref=off

Peak #	RetTime [min]	Type	Width [min]	Area [mAU*s]	Height [mAU]	Area %
1	22.813	BB	0.6825	1197.31787	26.03769	3.6616
2	29.079	BV	0.8347	1.40843e4	258.07257	43.0725
3	31.638	VB	1.0374	1.74174e4	248.60435	53.2659

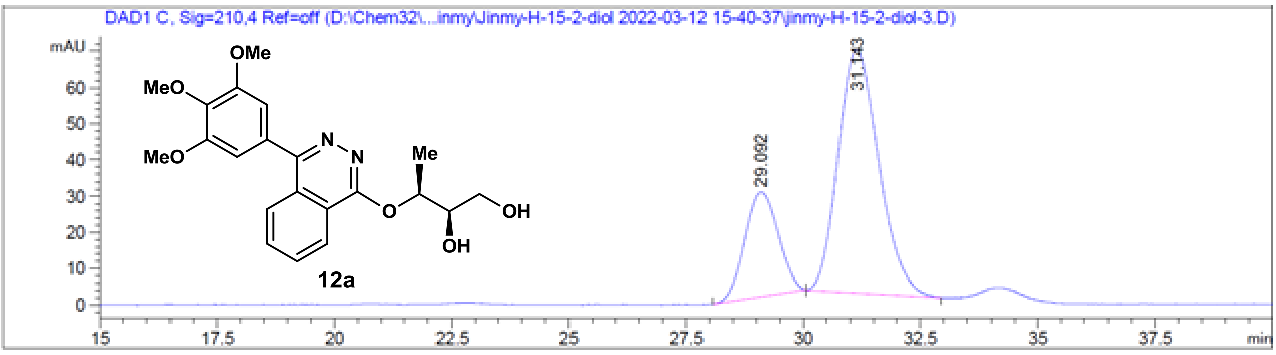

Signal 2: DAD1 C, Sig=210,4 Ref=off

Peak #	RetTime [min]	Type	Width [min]	Area [mAU*s]	Height [mAU]	Area %
1	29.092	BB	0.6578	1449.76685	29.07092	26.2659
2	31.143	BB	0.8754	4069.80811	67.12224	73.7341

Supplementary Figure 83. HPLC chromatogram for **12a**.

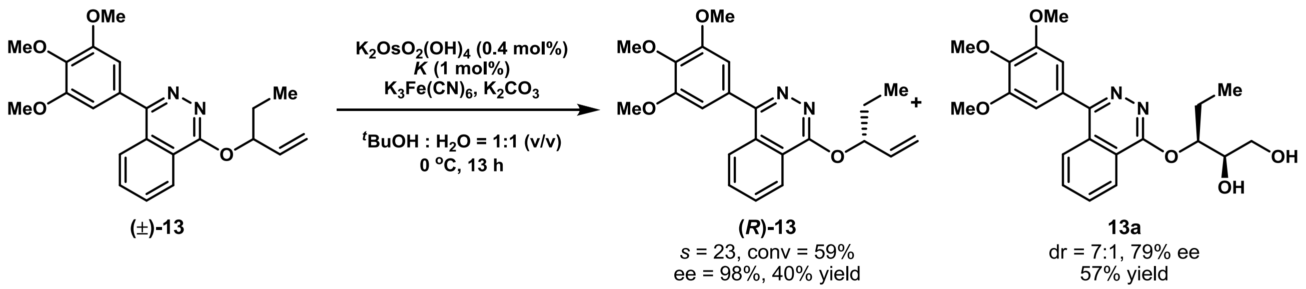

The general procedure **IV** was followed. The conversion of **(±)-13** was determined by crude ^{1}H NMR. **Conversion** (%) = [(2.42 – 1.00) / 2.42] % = 59%.

S = In [(1 – conv)(1 – ee)] / In [(1 – conv)(1 + ee)] = 23.

The recovered alkene **(*R*)-13** (15.2 mg, 40% yield, 98% ee) was purified by chromatography on silica gel (eluted with petroleum ether : ethyl acetate = 3:1). $[\alpha]_D^{25}$ = –8.02 (*c* 0.67, $CHCl_3$).

The dihydroxylated product **13a** (dr = 7:1, 23.6 mg, 57% yield, 79% ee) was purified by chromatography on silica gel (eluted with petroleum ether : ethyl acetate = 1:2). $[\alpha]_D^{25}$ = – 8.37 (*c* 0.77, $CHCl_3$).

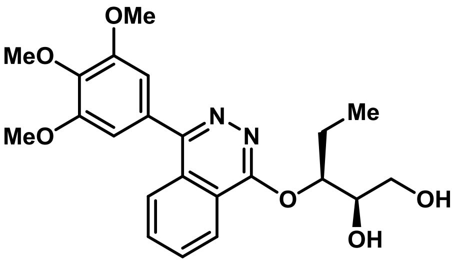

13a

(2*R*,3*S*)-3-(4-(3,4,5-trimethoxyphenyl)phthalazin-1-yloxy)pentane-1,2-diol

^{1}H NMR (600 MHz, $CDCl_3$): δ 8.35 (d, *J* = 6.0 Hz, 1H), 8.08 (d, *J* = 6.0 Hz, 1H), 7.95 – 7.87 (m, 2H), 6.90 (s, 2H), 5.40 – 5.32 (m, 1H), 4.15 – 3.97 (m, 2H), 3.95 (s, 3H), 3.91 (s, 6H), 3.86 (s, 1H), 3.78 – 3.73 (m, 1H), 3.72 – 3.67 (m, 1H), 2.19 – 2.09 (m, 1H), 2.06 – 1.97 (m, 1H), 1.09 (t, *J* = 6.0 Hz, 3H) ppm.

^{13}C NMR (151 MHz, $CDCl_3$): δ 161.0, 157.1, 153.4, 139.0, 132.9, 132.2, 131.3, 128.2, 126.5, 123.4, 120.4, 107.2, 80.6, 73.4, 62.3, 61.0, 56.3, 29.7, 25.3, 10.3 ppm.

HRMS (ESI) m/z: $[M + H]^+$ Calcd for $C_{22}H_{27}N_2O_6$ 415.1864; Found 415.1864.

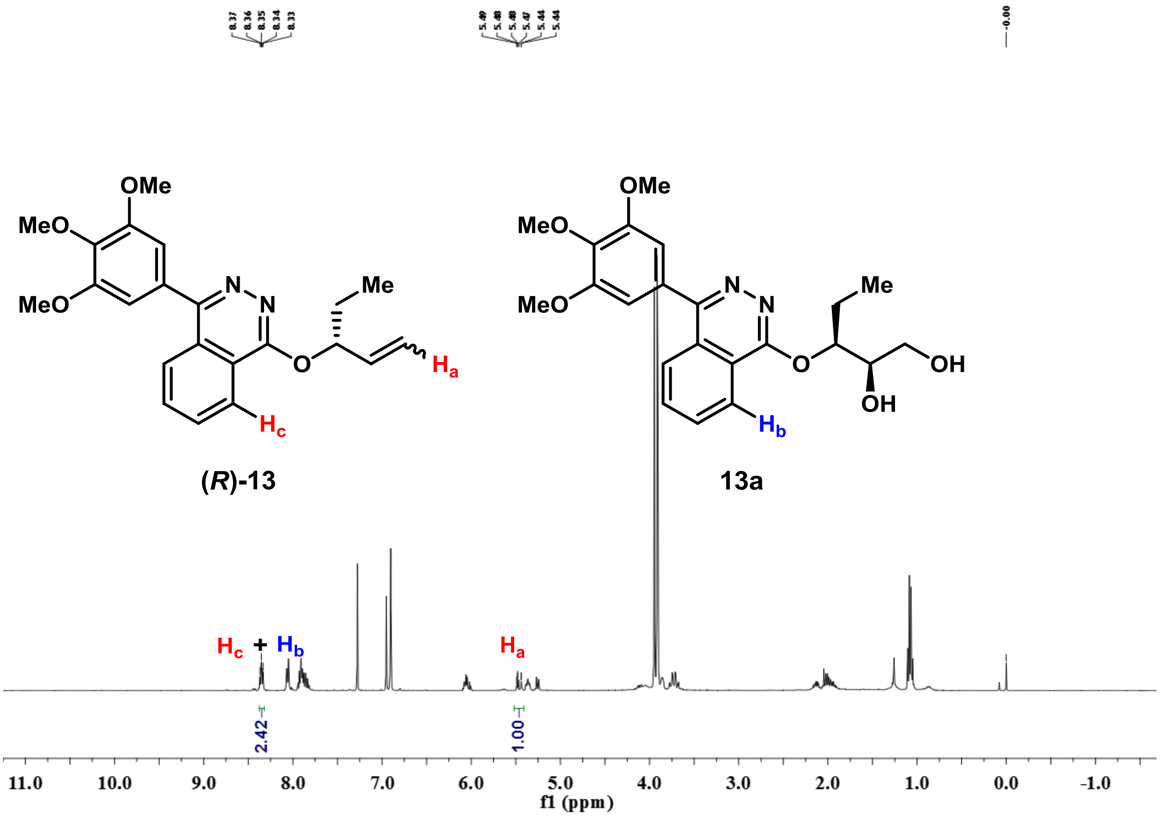

Supplementary Figure 84. ^{1}H NMR spectrum of crude mixture of compound **(*R*)-13** and **13a.**

:

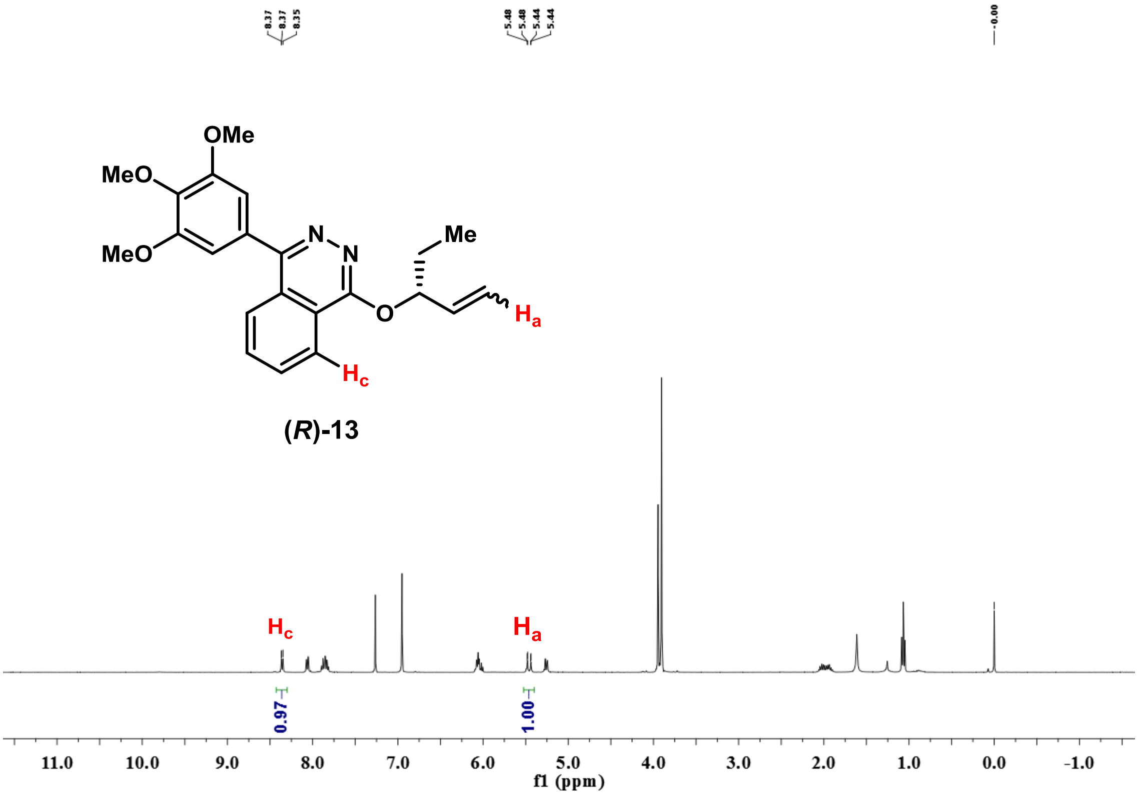

Supplementary Figure 85. ^{1}H NMR spectrum of recovered alkene **(*R*)-13**.

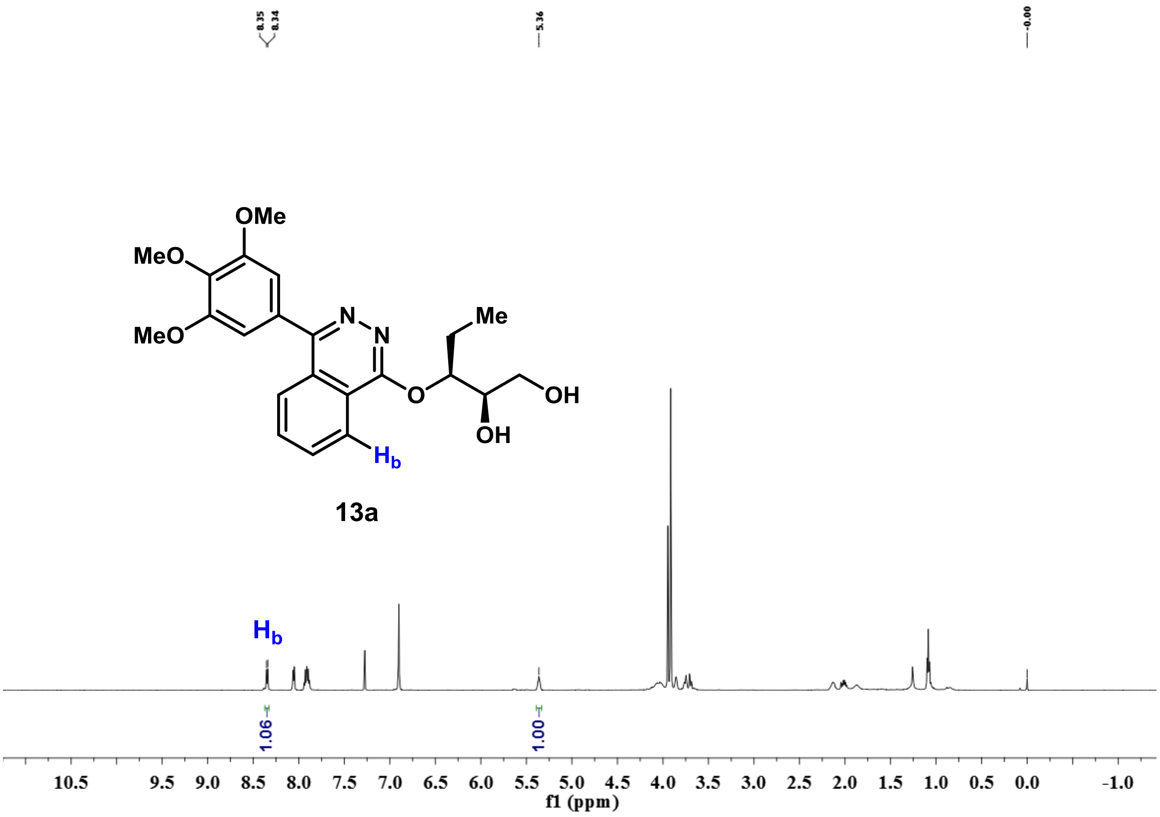

Supplementary Figure 86. ^{1}H NMR spectrum of dihydroxylated product **13a**.

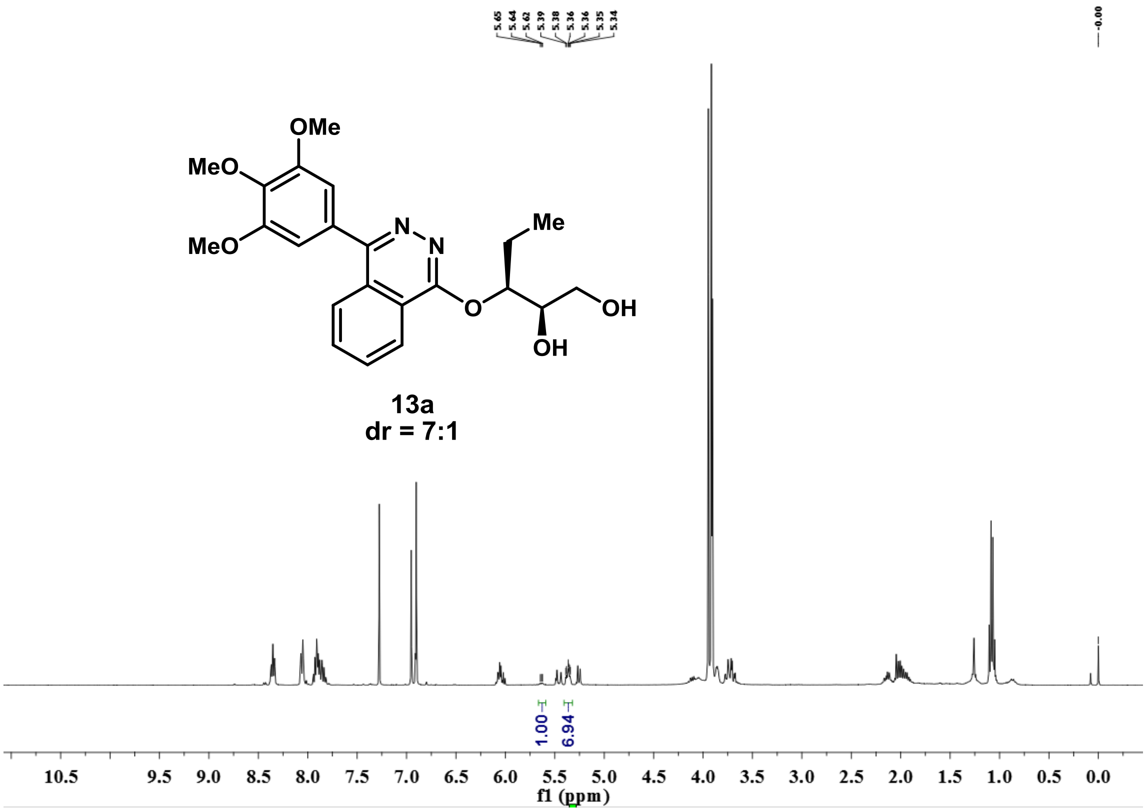

Supplementary Figure 87. ^{1}H NMR spectrum of crude mixture for diastereomeric ratio (dr).

HPLC (AD-H, 0.46*25 cm, 5μm, hexane/isopropanol = 90/10, flow = 1.0 mL/min, detection at 210 nm), retention time = 16.702 min (major) and 24.110 min (minor).

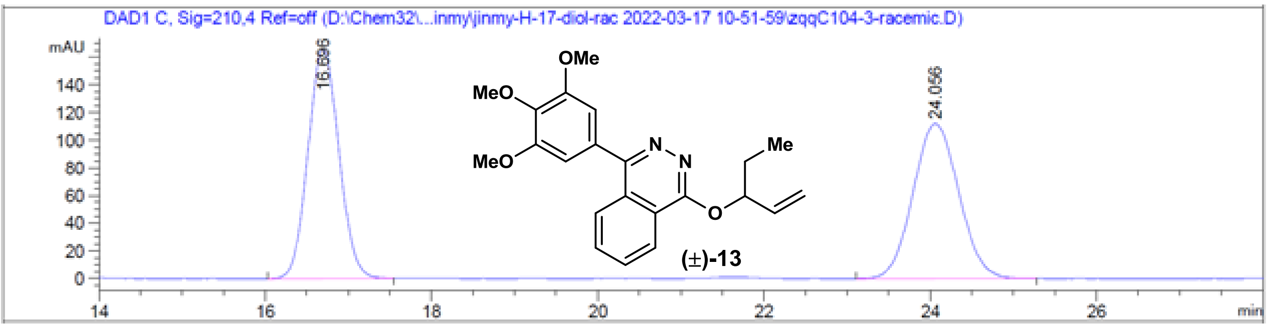

Signal 2: DAD1 C, Sig=210,4 Ref=off

Peak #	RetTime [min]	Type	Width [min]	Area [mAU*s]	Height [mAU]	Area %
1	16.696	BB	0.4035	4283.48047	165.75980	50.0437
2	24.056	BB	0.5994	4275.99463	112.00613	49.9563

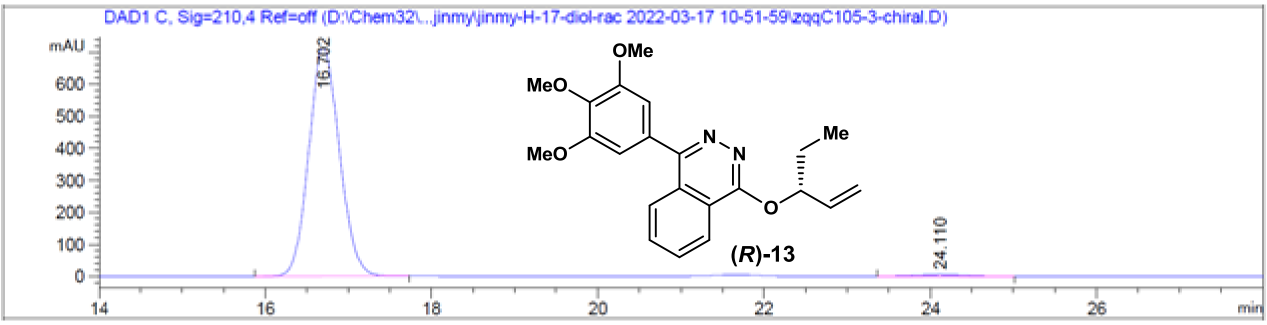

Signal 2: DAD1 C, Sig=210,4 Ref=off

Peak #	RetTime [min]	Type	Width [min]	Area [mAU*s]	Height [mAU]	Area %
1	16.702	BB	0.4044	1.84726e4	712.68005	98.8759
2	24.110	BB	0.4610	210.01492	5.50565	1.1241

Supplementary Figure 88. HPLC chromatogram for **(*R*)-13**.

HPLC (Waters ACQUITY UPC2, C2, 5% MeOH, flow = 0.6 mL/min, detection at 210 nm), retention time = 30.915 min (minor) and 32.463 min (major).

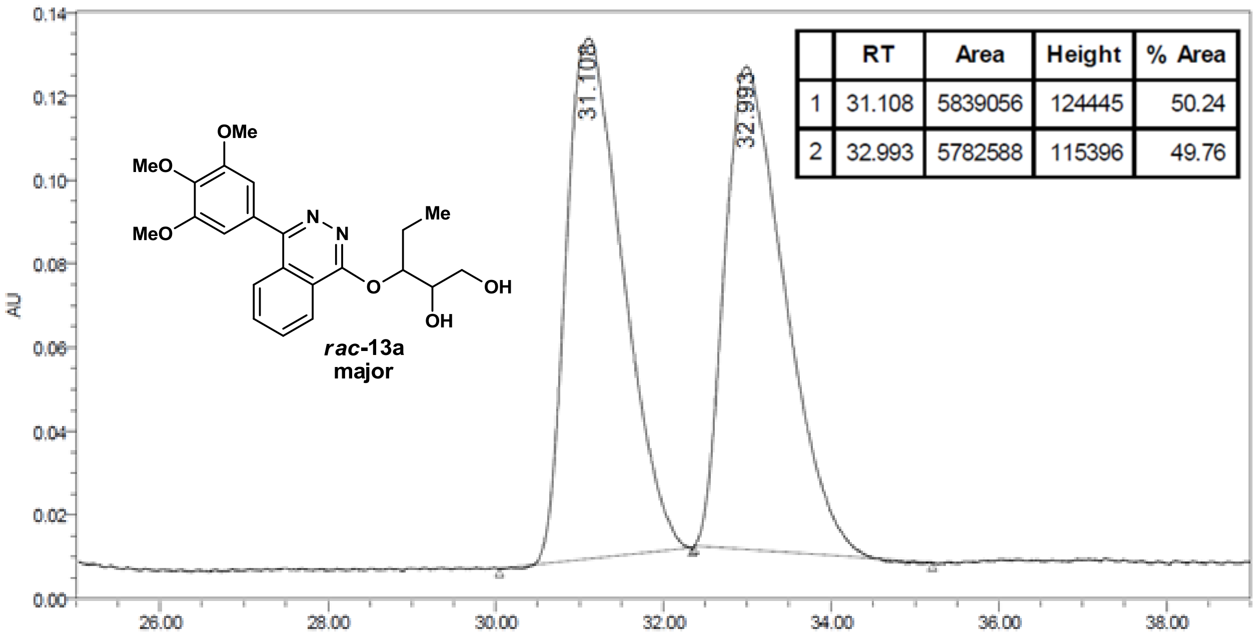

	RT	Area	Height	% Area
1	31.108	5839056	124445	50.24
2	32.993	5782588	115396	49.76

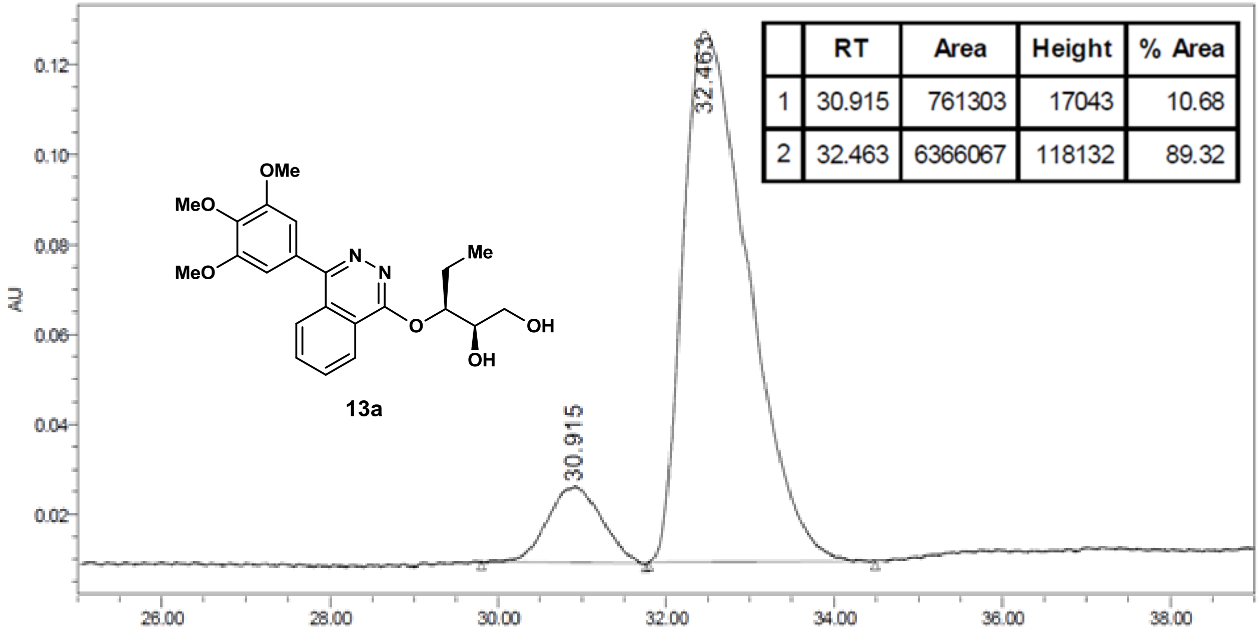

	RT	Area	Height	% Area
1	30.915	761303	17043	10.68
2	32.463	6366067	118132	89.32

Supplementary Figure 89. HPLC chromatogram for **13a**.

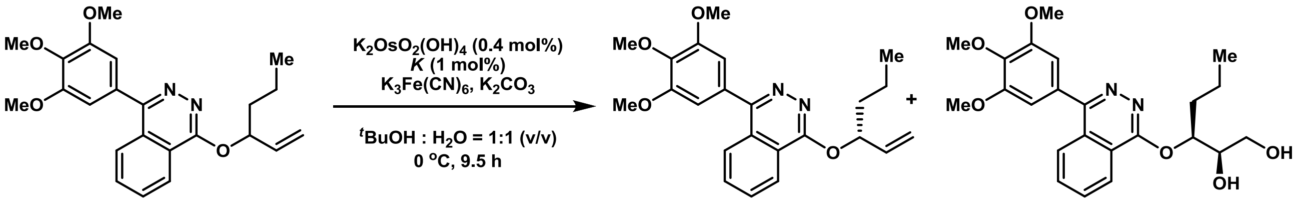

(±)-14

(*R*)-14
s = 15, conv = 56%
ee = 87%, 41% yield

14a
dr = 8:1, 78% ee
53% yield

The general procedure **IV** was followed. The conversion of **(±)-14** was determined by crude ^{1}H NMR. **Conversion** (%) = [(2.27 – 1.00) / 2.27] % = 56%.

S = In [(1 – conv)(1 – ee)] / In [(1 – conv)(1 + ee)] = 15.

The recovered alkene **(*R*)-14** (16.2 mg, 41% yield, 87% ee) was purified by chromatography on silica gel (eluted with petroleum ether : ethyl acetate = 3:1). $[\alpha]_D^{25}$ = –7.10 (*c* 0.67, $CHCl_3$).

The dihydroxylated product **14a** (dr = 8:1, 22.7 mg, 53% yield, 78% ee) was purified by chromatography on silica gel (eluted with petroleum ether : ethyl acetate = 1:2). $[\alpha]_D^{25}$ = – 5.90 (*c* 0.95, $CHCl_3$).

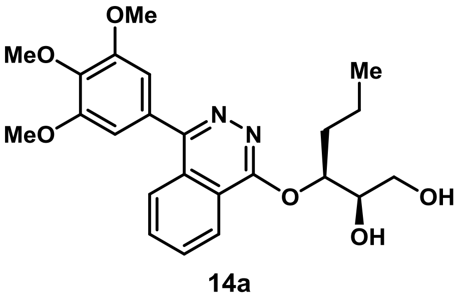

14a

(2*R*,3*S*)-3-(4-(3,4,5-trimethoxyphenyl)phthalazin-1-yloxy)hexane-1,2-diol

^{1}H NMR (600 MHz, $CDCl_3$): δ 8.34 (d, *J* = 6.0 Hz, 1H), 8.06 (d, *J* = 6.0 Hz, 1H), 7.95 – 7.87 (m, 2H), 6.90 (s, 2H), 5.45 – 5.39 (m, 1H), 3.95 (s, 3H), 3.91 (s, 6H), 3.85 – 3.80 (m, 1H), 3.77 – 3.73 (m, 1H), 3.71 – 3.66 (m, 1H), 2.09 – 1.95 (m, 2H), 1.65 – 1.53 (m, 2H), 1.52 – 1.41 (m, 2H), 0.97 (t, *J* = 6.0 Hz, 3H) ppm.

^{13}C NMR (151 MHz, $CDCl_3$): δ 161.0, 157.1, 153.4, 139.0, 132.9, 132.2, 131.3, 128.2, 126.5, 123.4, 120.4, 107.2, 79.2, 73.7, 62.2, 61.0, 56.3, 34.4, 19.2, 14.0 ppm.

HRMS (ESI) m/z: [M + H]$^+$ Calcd for $C_{22}H_{29}N_2O_6$ 429.2020; Found 429.2024.

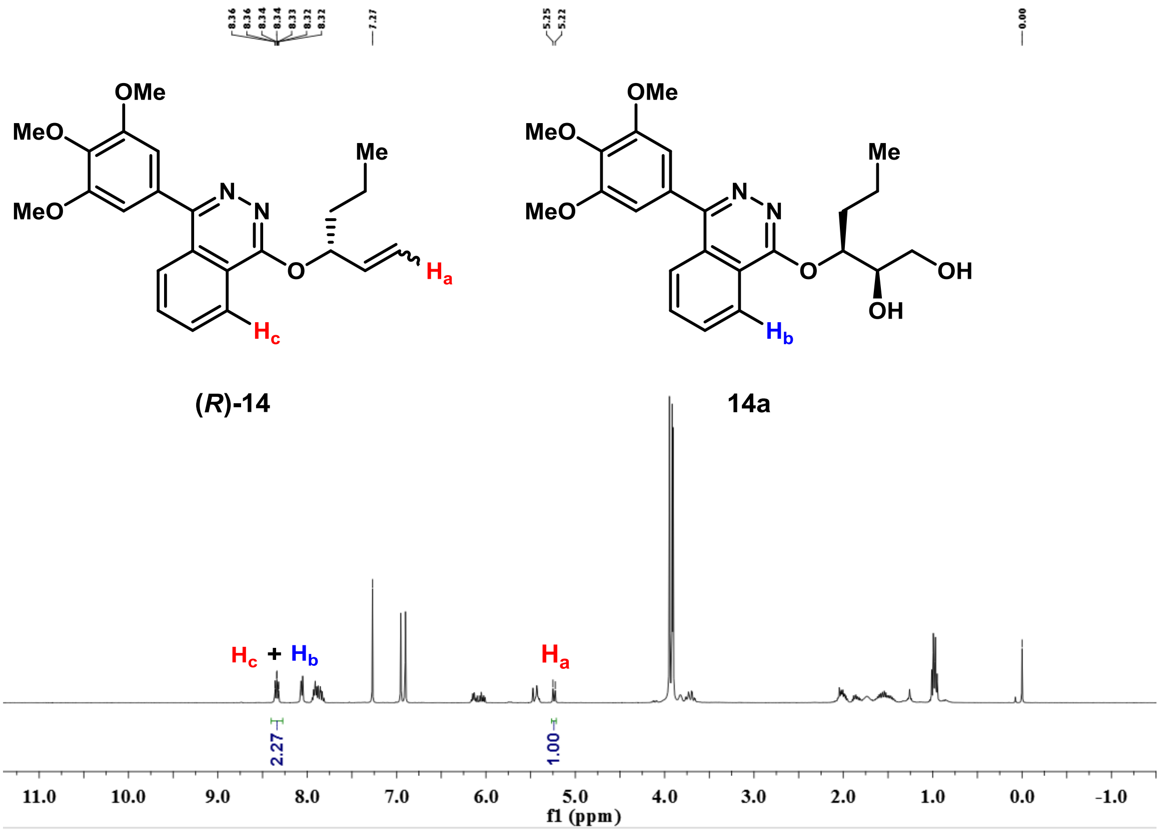

Supplementary Figure 90. ^{1}H NMR spectrum of crude mixture of compound **(*R*)-14** and **14a.**

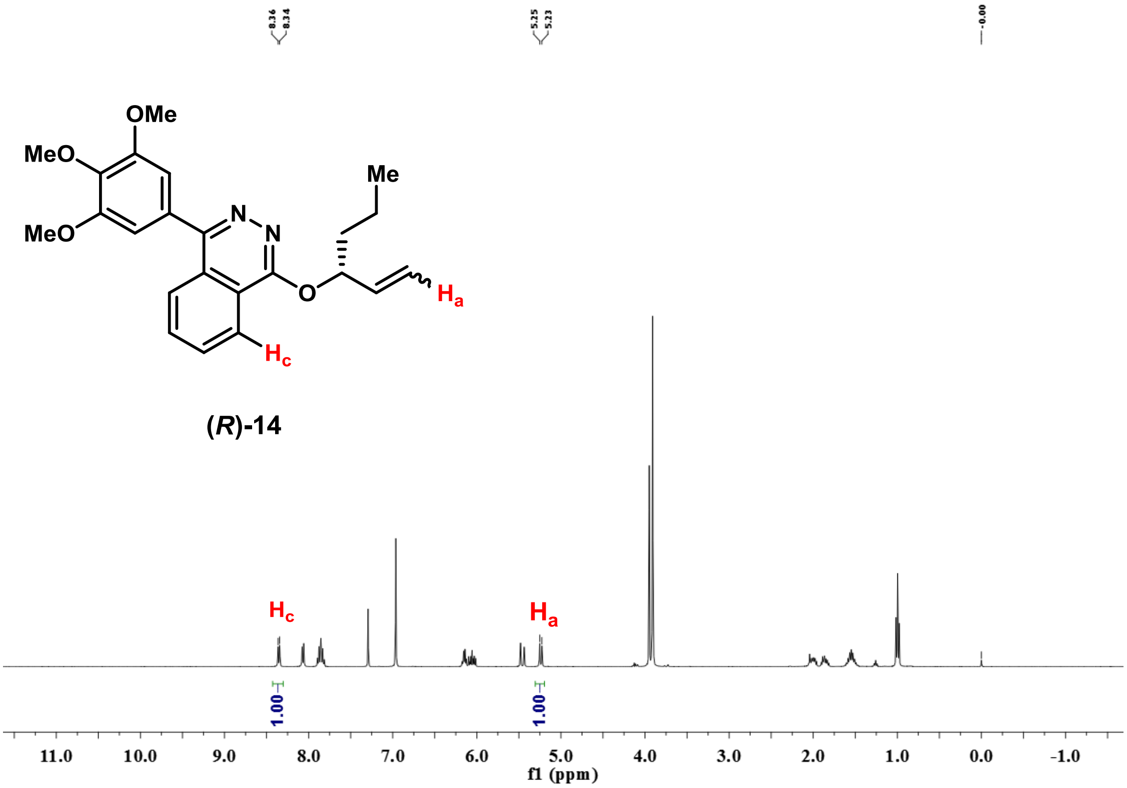

Supplementary Figure 91. ^{1}H NMR spectrum of recovered alkene **(*R*)-14**.

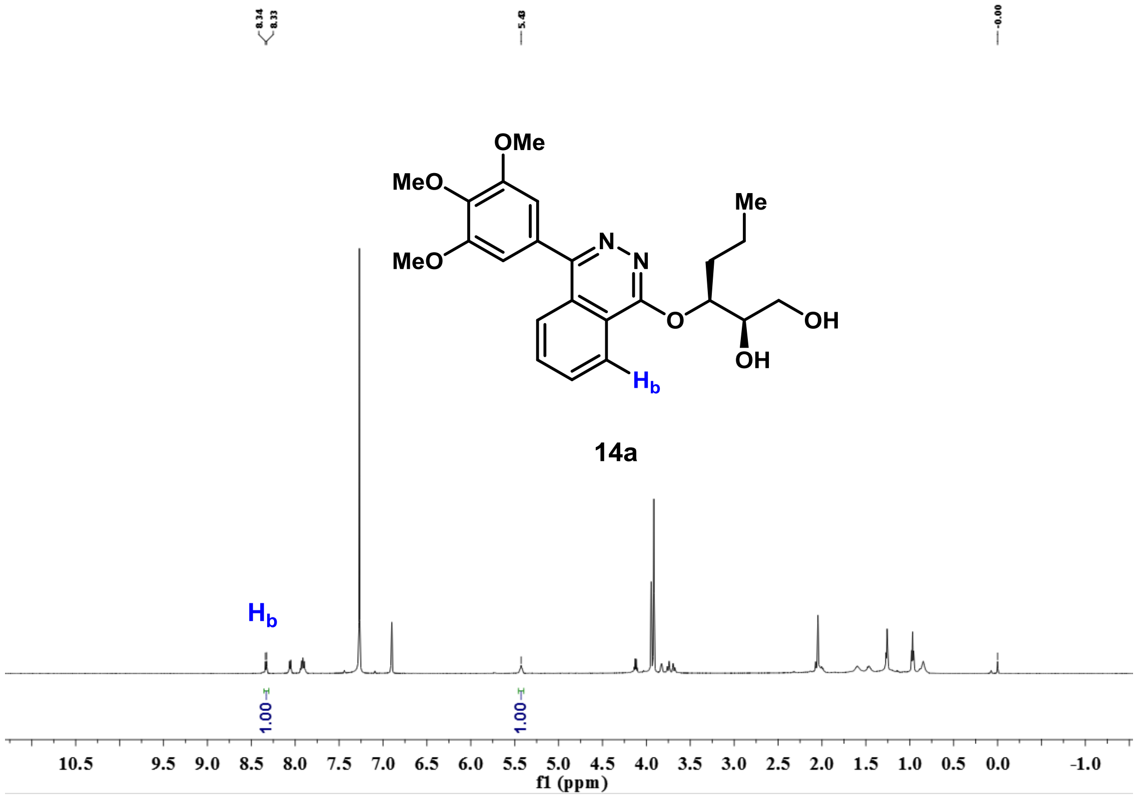

Supplementary figure 92. ^{1}H NMR spectrum of dihydroxylated product **14a**.

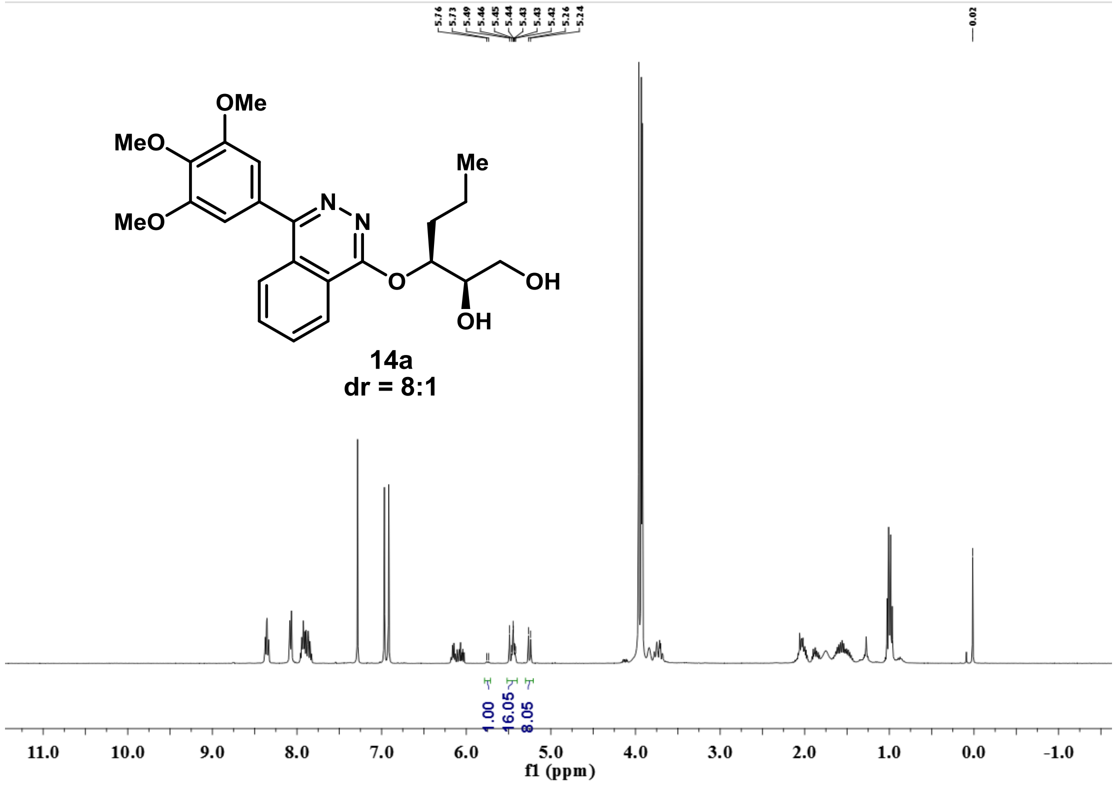

Supplementary Figure 93. ^{1}H NMR spectrum of crude mixture for diastereomeric ratio (dr).

HPLC (AD-H, 0.46*25 cm, 5μm, hexane/isopropanol = 90/10, flow = 1.0 mL/min, detection at 210 nm), retention time = 20.882 min (minor) and 23.447 min (major).

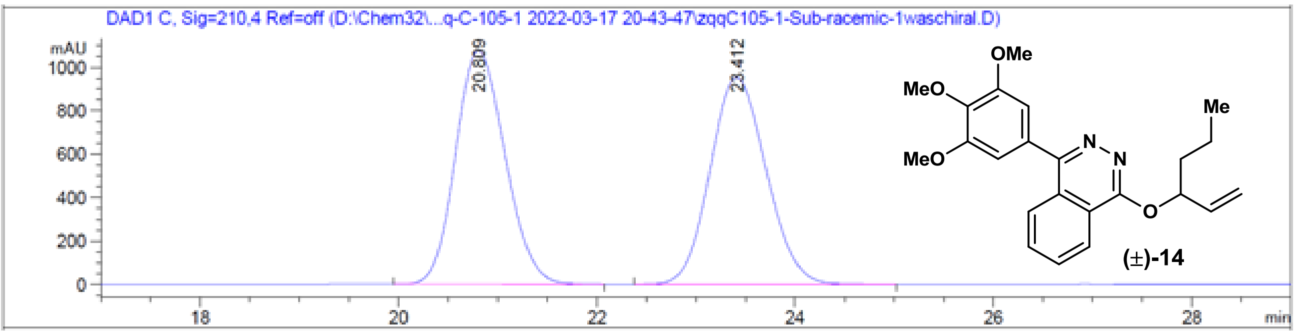

Signal 2: DAD1 C, Sig=210,4 Ref=off

Peak #	RetTime [min]	Type	Width [min]	Area [mAU*s]	Height [mAU]	Area %
1	20.809	BB	0.5442	3.73486e4	1078.07141	49.8999
2	23.412	BB	0.6193	3.74984e4	952.43567	50.1001

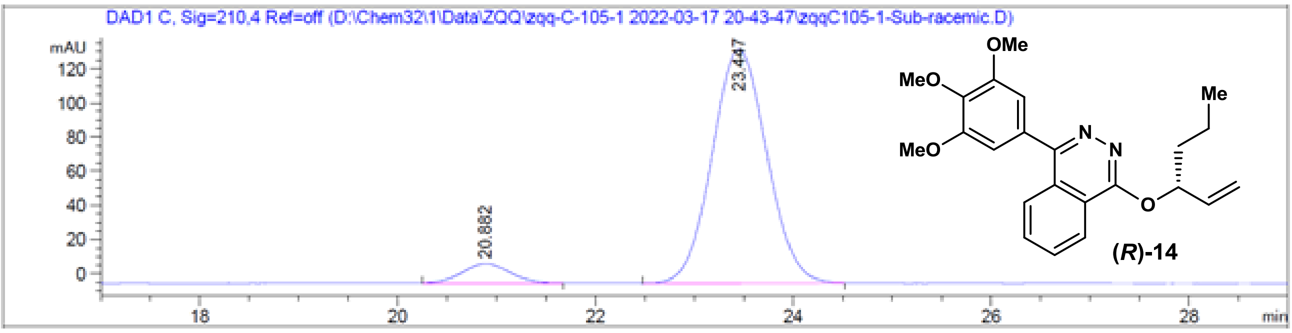

Signal 2: DAD1 C, Sig=210,4 Ref=off

Peak #	RetTime [min]	Type	Width [min]	Area [mAU*s]	Height [mAU]	Area %
1	20.882	BB	0.4269	376.28702	11.28706	6.6883
2	23.447	BB	0.6018	5249.74414	136.77281	93.3117

Supplementary Figure 94. HPLC chromatogram for ***(R)*-14**.

HPLC (AD-H, 0.46*25 cm, 5μm, hexane/isopropanol = 80/20, flow = 1.0 mL/min, detection at 210 nm), retention time = 17.324 min (major) and 24.137 min (minor).

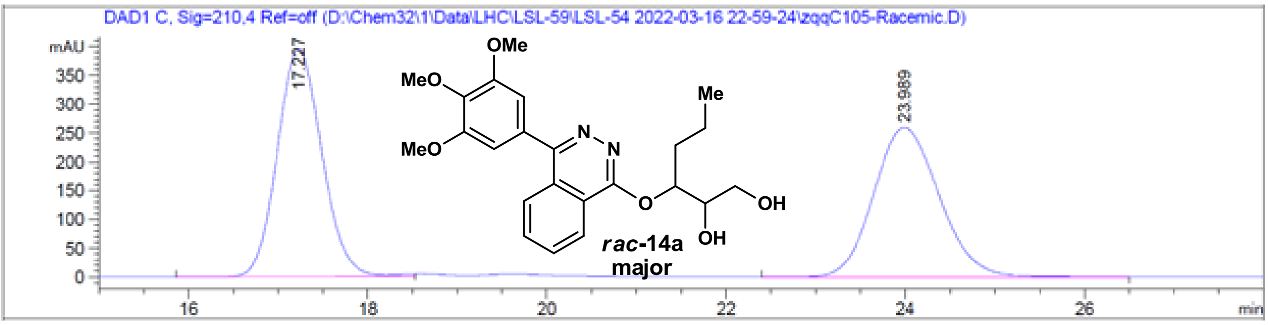

Signal 3: DAD1 C, Sig=210,4 Ref=off

Peak #	RetTime [min]	Type	Width [min]	Area [mAU*s]	Height [mAU]	Area %
1	17.227	MM R	0.5760	1.36958e4	396.31027	50.9415
2	23.989	BB	0.7971	1.31895e4	259.43689	49.0585

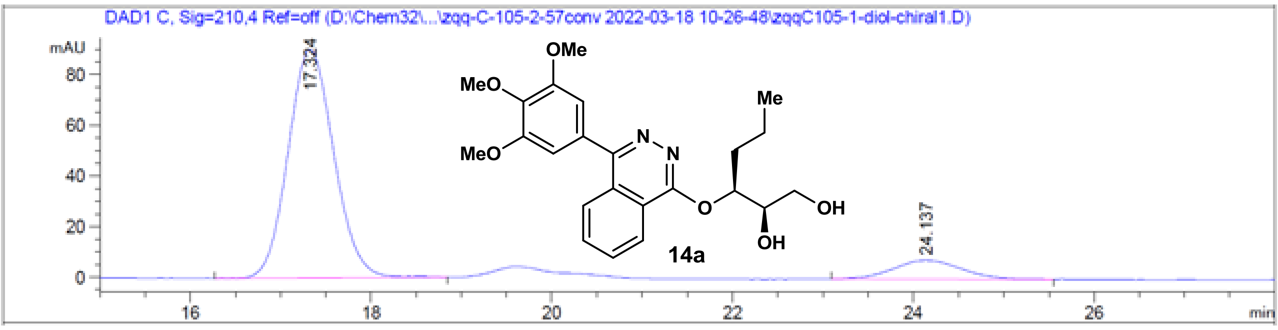

Signal 3: DAD1 C, Sig=210,4 Ref=off

Peak #	RetTime [min]	Type	Width [min]	Area [mAU*s]	Height [mAU]	Area %
1	17.324	BB	0.5481	3180.85474	90.48270	88.8329
2	24.137	BB	0.6394	399.86362	7.55539	11.1671

Supplementary Figure 95. HPLC chromatogram for **14a**.

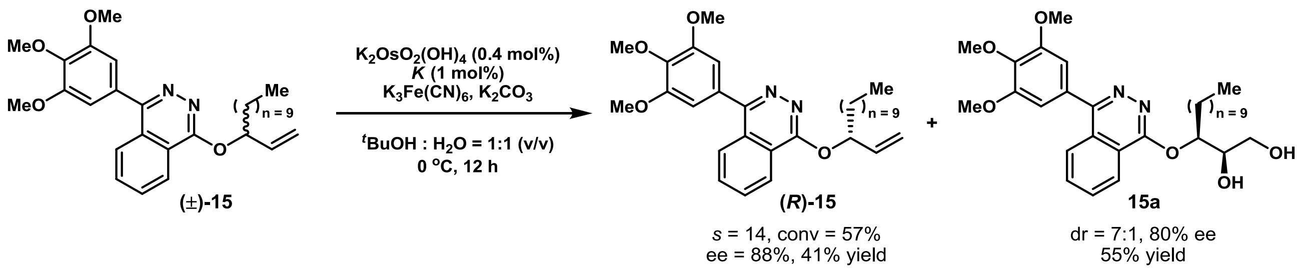

The general procedure **IV** was followed. The conversion of **(±)-15** was determined by crude ^{1}H NMR. **Conversion** (%) = [(2.33 – 1.00) / 2.33] % = 57%.

S = In [(1 – conv)(1 – ee)] / In [(1 – conv)(1 + ee)] = 14.

The recovered alkene **(*R*)-15** (20.2 mg, 41% yield, 88% ee) was purified by chromatography on silica gel (eluted with petroleum ether : ethyl acetate = 3:1). $[\alpha]_D^{25}$ = –5.48 (*c* 1.09, $CHCl_3$).

The dihydroxylated product **15a** (dr = 7:1, 22.7 mg, 55% yield, 80% ee) was purified by chromatography on silica gel (eluted with petroleum ether : ethyl acetate = 1:2). $[\alpha]_D^{25}$ = – 12.24 (*c* 0.85, $CHCl_3$).

`

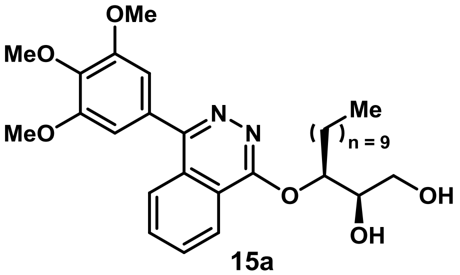

15a

(2*R*,3*S*)-3-(4-(3,4,5-trimethoxyphenyl)phthalazin-1-yloxy)tridecane-1,2-diol

^{1}H NMR (600 MHz, $CDCl_3$): δ 8.34 (d, *J* = 6.0 Hz, 1H), 8.07 (d, *J* = 6.0 Hz, 1H), 7.96 – 7.88 (m, 2H), 6.90 (s, 2H), 5.29 (d, *J* = 12.0 Hz, 1H), 5.20 – 4.80 (m, 1H), 3.95 (s, 3H), 3.92 (s, 6H), 3.88 – 3.83 (m, 1H), 3.67 – 3.60 (m, 1H), 2.84 – 2.60 (m, 1H), 2.55 – 2.46 (m, 1H), 1.31 – 1.22 (m, 18H), 1.02 (d, *J* = 6.0 Hz, 3H) ppm.

^{13}C NMR (151 MHz, $CDCl_3$): δ 161.0, 157.1, 153.4, 139.0, 132.9, 132.2, 131.4, 128.2, 126.5, 123.4, 120.4, 107.2, 79.5, 73.7, 62.2, 61.0, 56.3, 32.3, 31.9, 29.7, 29.55, 29.52, 29.47, 29.4, 29.3, 25.9, 22.7, 14.1 ppm.

HRMS (ESI) m/z: $[M + H]^+$ Calcd for $C_{30}H_{43}N_2O_6$ 527.3116; Found 527.3119.

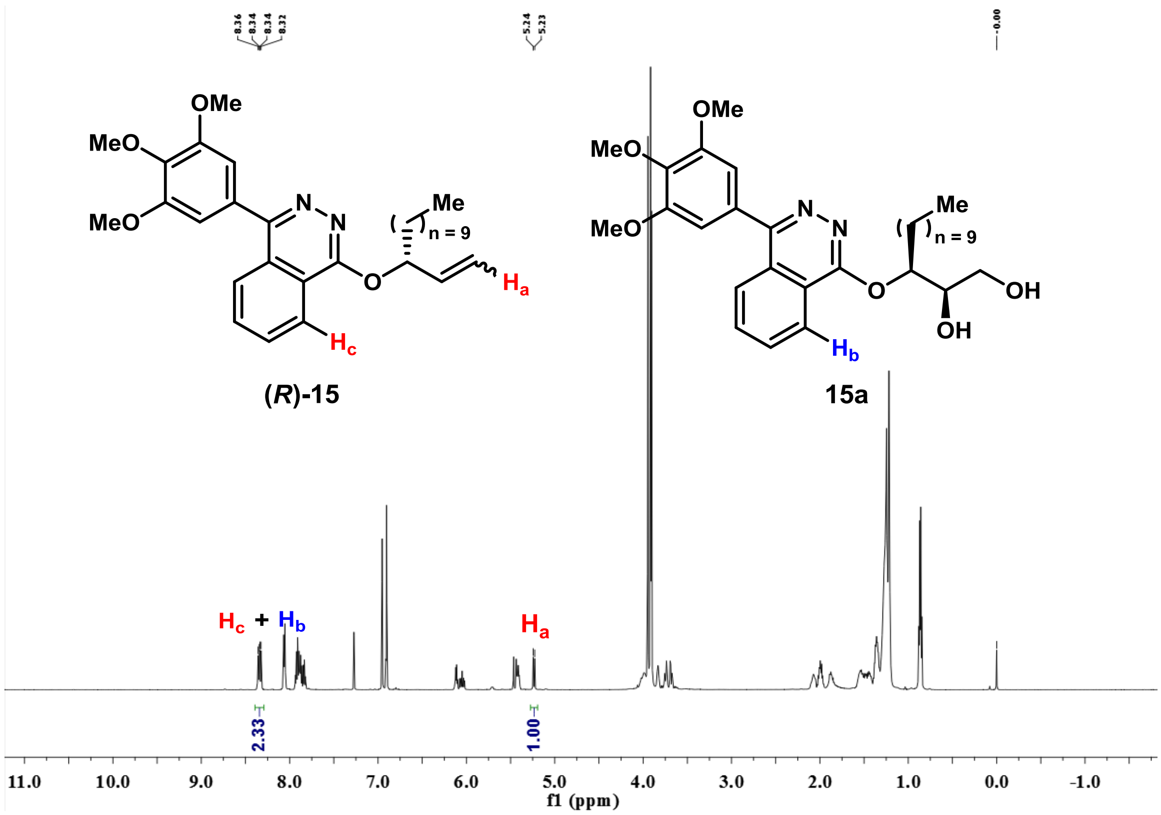

Supplementary Figure 96. ^{1}H NMR spectrum of crude mixture of compound **(*R*)-15** and **15a.**

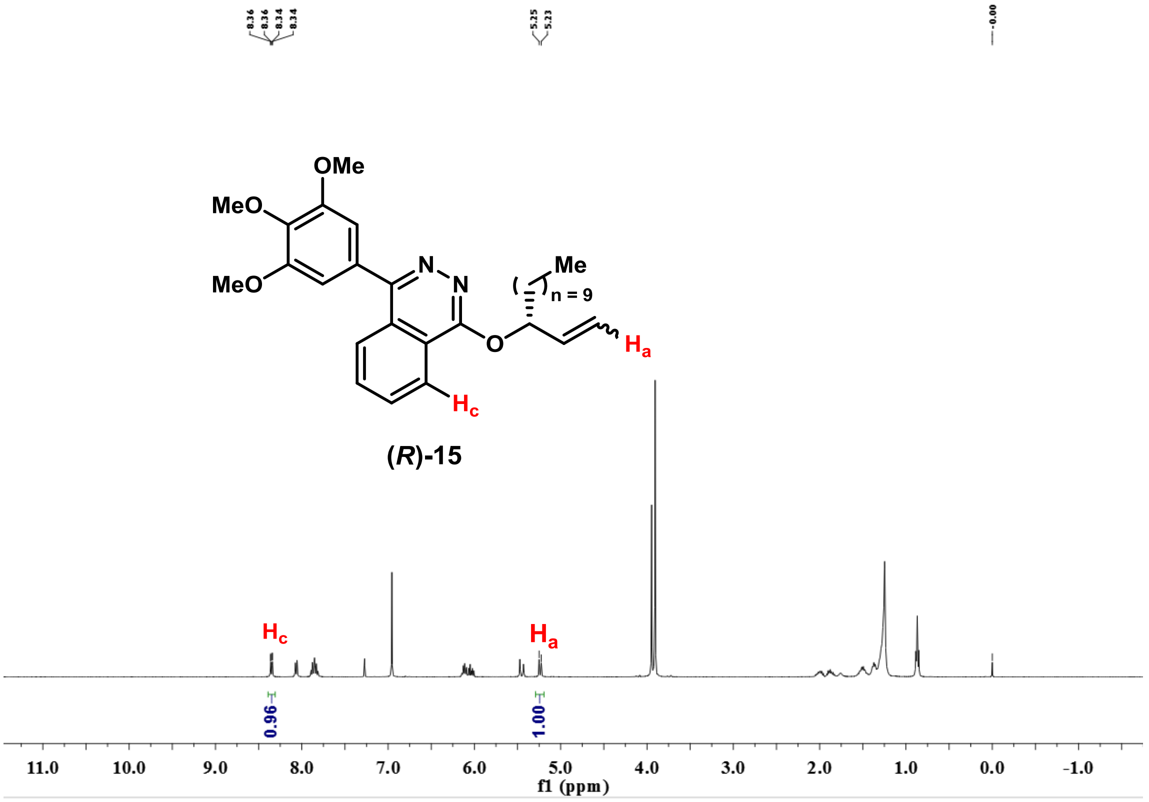

Supplementary Figure 97. ^{1}H NMR spectrum of recovered alkene **(*R*)-15**.

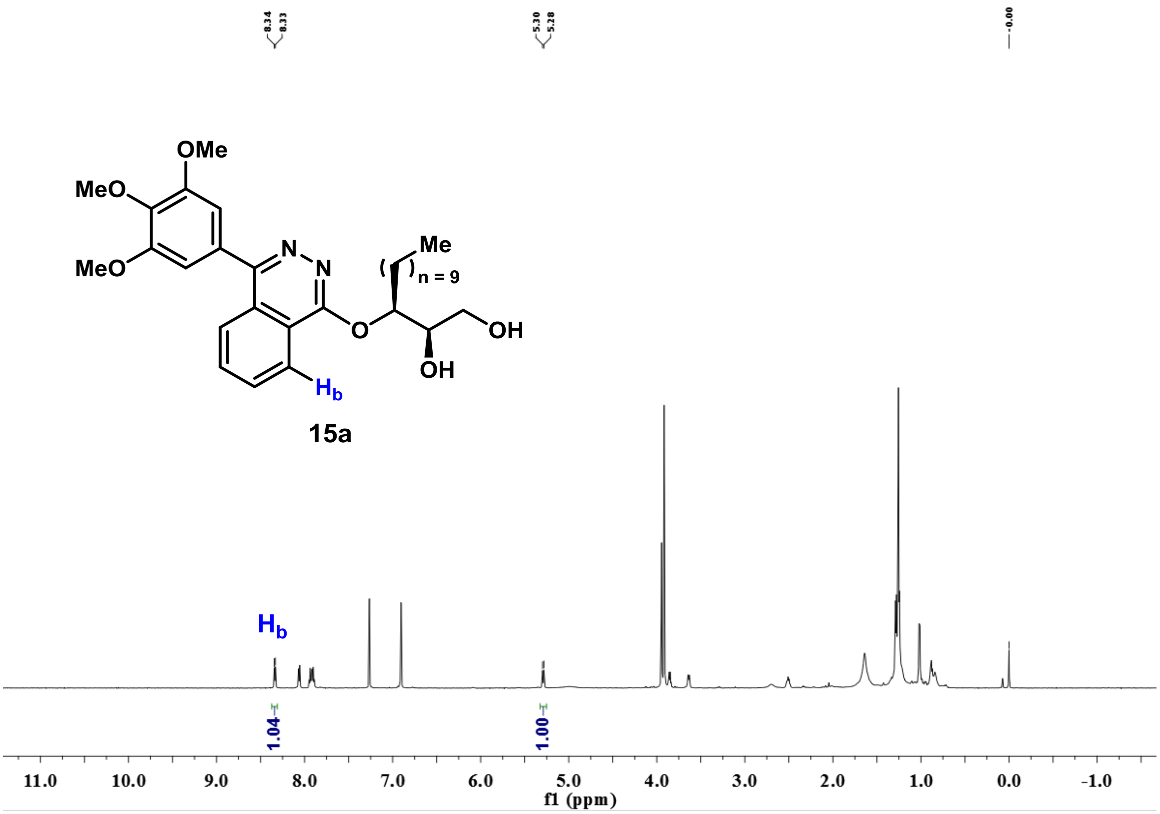

Supplementary Figure 98. ^{1}H NMR spectrum of dihydroxylated product **15a**.

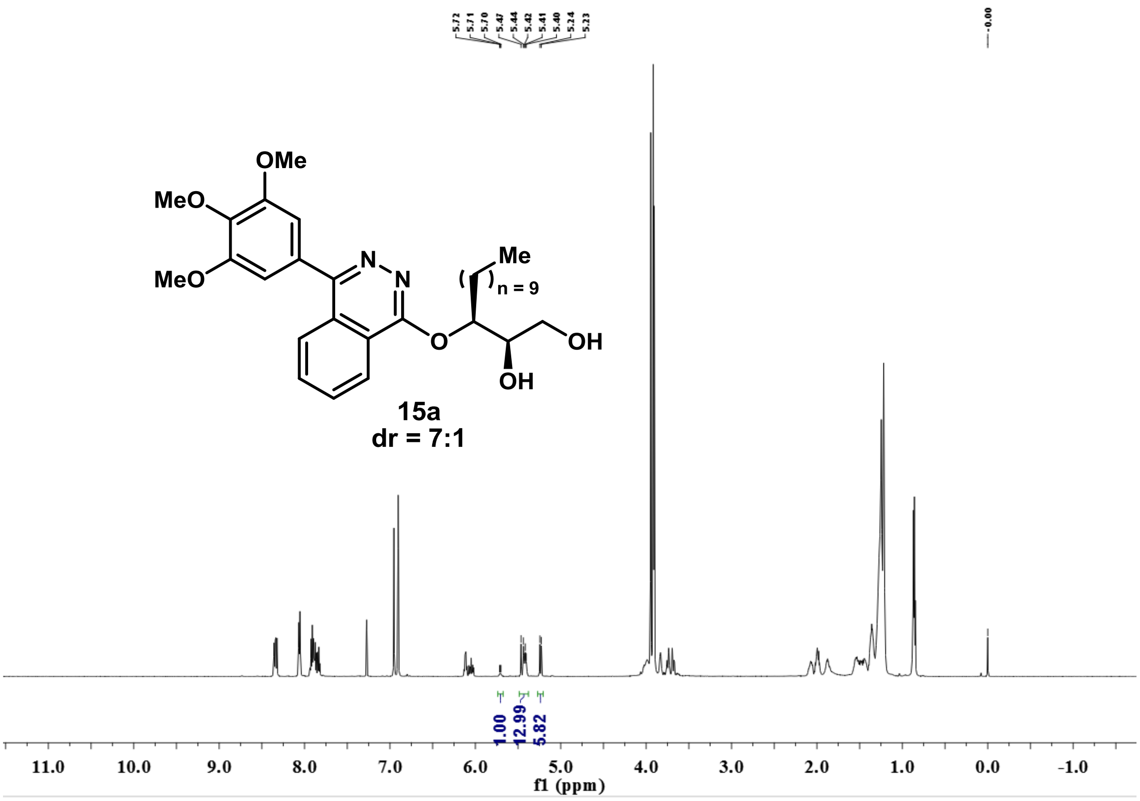

Supplementary Figure 99. ^{1}H NMR spectrum of crude mixture for diastereomeric ratio (dr).

HPLC (AD-H, 0.46*25 cm, 5µm, hexane/isopropanol = 90/10, flow = 1.0 mL/min, detection at 254 nm), retention time = 10.372 min (minor) and 13.023 min (major).

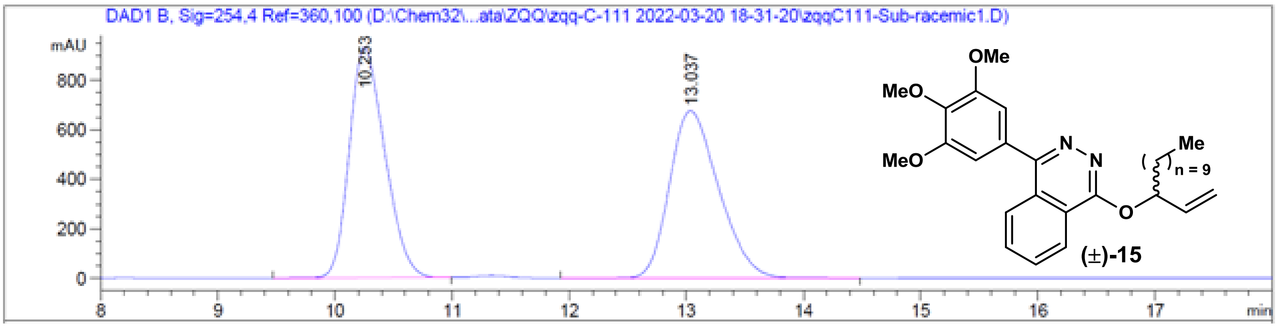

Signal 1: DAD1 B, Sig=254,4 Ref=360,100

Peak #	RetTime [min]	Type	Width [min]	Area [mAU*s]	Height [mAU]	Area %
1	10.253	BB	0.3254	1.96063e4	931.94543	49.8320
2	13.037	BB	0.4502	1.97385e4	676.94891	50.1680

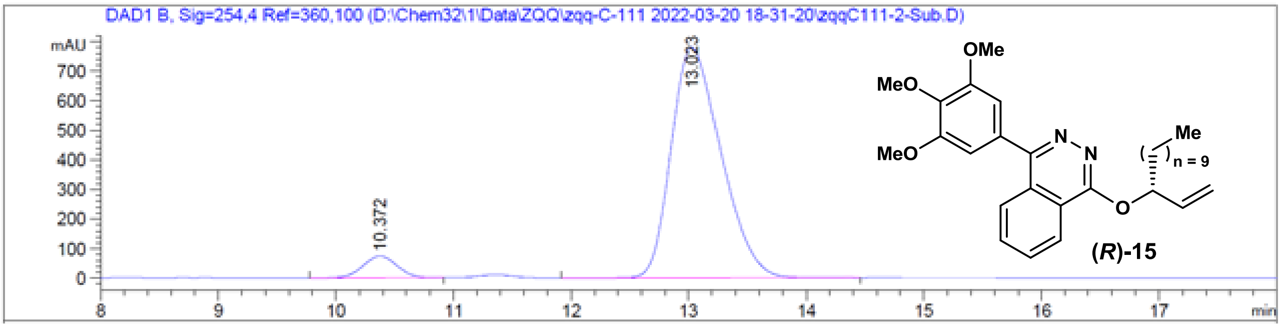

Signal 1: DAD1 B, Sig=254,4 Ref=360,100

Peak #	RetTime [min]	Type	Width [min]	Area [mAU*s]	Height [mAU]	Area %
1	10.372	BB	0.3195	1508.93506	74.13635	6.1525
2	13.023	BB	0.4555	2.30165e4	781.64874	93.8475

Supplementary Figure 100. HPLC chromatogram for ***(R)*-15**.

HPLC (AD-H, 0.46*25 cm, 5μm, hexane/isopropanol = 75/25, flow = 1.0 mL/min, detection at 254 nm), retention time = 8.049 min (major) and 9.761 min (minor).

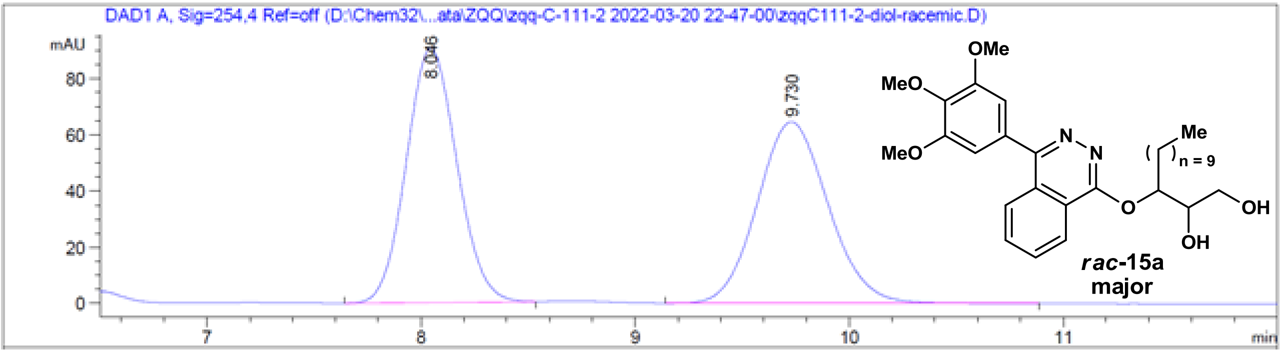

Signal 1: DAD1 A, Sig=254,4 Ref=off

Peak #	RetTime [min]	Type	Width [min]	Area [mAU*s]	Height [mAU]	Area %
1	8.046	BB	0.2549	1484.99805	90.73893	49.8443
2	9.730	BB	0.3600	1494.27771	64.57133	50.1557

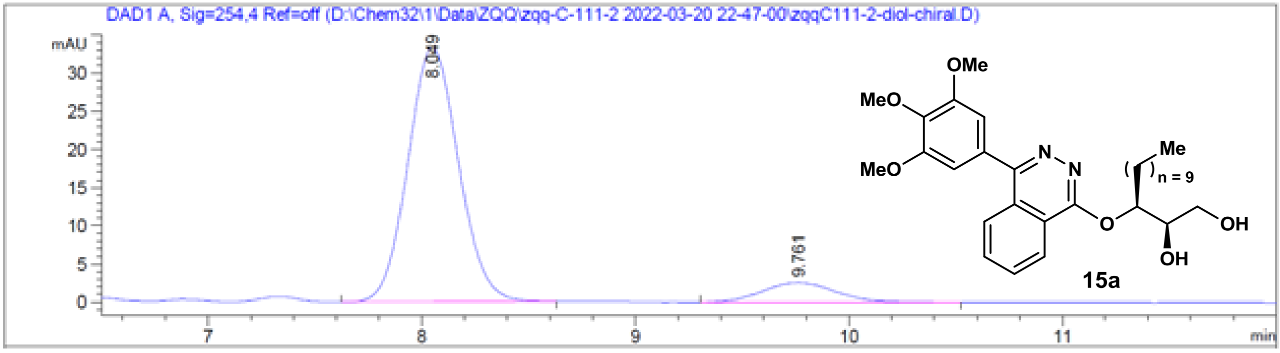

Signal 1: DAD1 A, Sig=254,4 Ref=off

Peak #	RetTime [min]	Type	Width [min]	Area [mAU*s]	Height [mAU]	Area %
1	8.049	BB	0.2562	549.29089	33.34343	90.0459
2	9.761	BB	0.3429	60.72121	2.55848	9.9541

Supplementary Figure 101. HPLC chromatogram for **15a**.

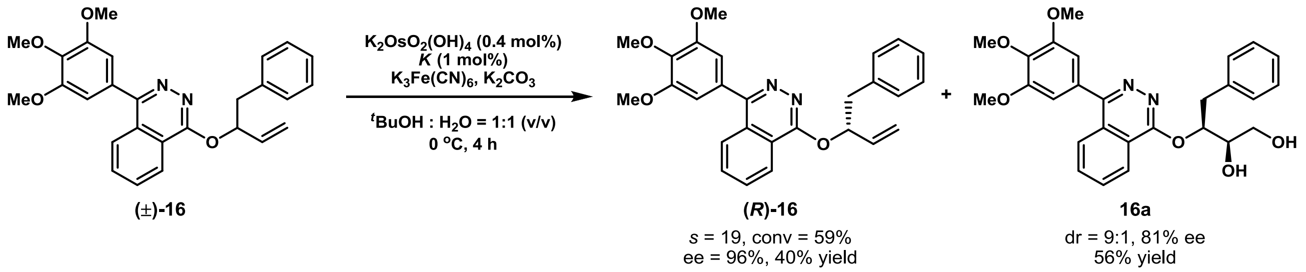

The general procedure **IV** was followed. The conversion of **(±)-16** was determined by crude ^{1}H NMR. **Conversion** (%) = [(2.43 – 1.00) / 2.43] % = 59%.

S = In [(1 – conv)(1 – ee)] / In [(1 – conv)(1 + ee)] = 19.

The recovered alkene **(*R*)-16** (17.7 mg, 40% yield, 96% ee) was purified by chromatography on silica gel (eluted with petroleum ether : ethyl acetate = 3:1). $[\alpha]_D^{25}$ = –15.08 (*c* 0.81, $CHCl_3$).

The dihydroxylated product **16a** (dr = 9:1, 26.6 mg, 56% yield, 81% ee) was purified by chromatography on silica gel (eluted with petroleum ether : ethyl acetate = 1:2). $[\alpha]_D^{25}$ = – 76.96 (*c* 0.56, $CHCl_3$)

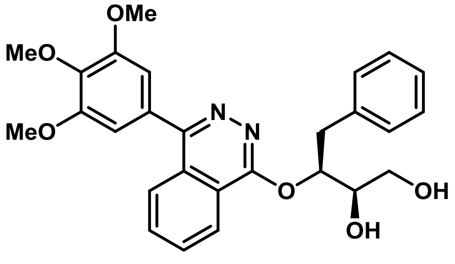

16a

(2*R*,3*S*)-4-phenyl-3-(4-(3,4,5-trimethoxyphenyl)phthalazin-1-yloxy)butane-1,2-diol

^{1}H NMR (600 MHz, $CDCl_3$): δ 8.23 (d, *J* = 12.0 Hz, 1H), 8.10 (d, *J* = 12.0 Hz, 1H), 7.91 – 7.83 (m, 2H), 7.35 (d, *J* = 12.0 Hz, 2H, 7.23 (t, *J* = 6.0 Hz, 2H), 7.14 (t, *J* = 6.0 Hz, 1H), 6.85 (s, 2H), 5.70 – 5.63 (m, 1H), 4.19 – 4.10 (m, 1H), 3.93 (s, 3H), 3.91 – 3.86 (m, 2H), 3.89 (s, 6H), 3.80 – 3.74 (m, 1H), 3.72 – 3.66 (m, 1H), 3.50 – 3.42 (m, 1H), 3.34 – 3.25 (m, 1H) ppm.

^{13}C NMR (151 MHz, $CDCl_3$): δ 160.5, 157.2, 153.4, 139.0, 137.7, 132.8, 132.1, 131.3, 129.6, 128.4, 128.2, 126.4, 126.38, 123.4, 120.3, 107.1, 79.1, 72.9, 62.1, 61.0, 56.3, 38.4 ppm.

HRMS (ESI) m/z: $[M + H]^+$ Calcd for $C_{27}H_{29}N_2O_6$ 477.2020; Found 477.2014.

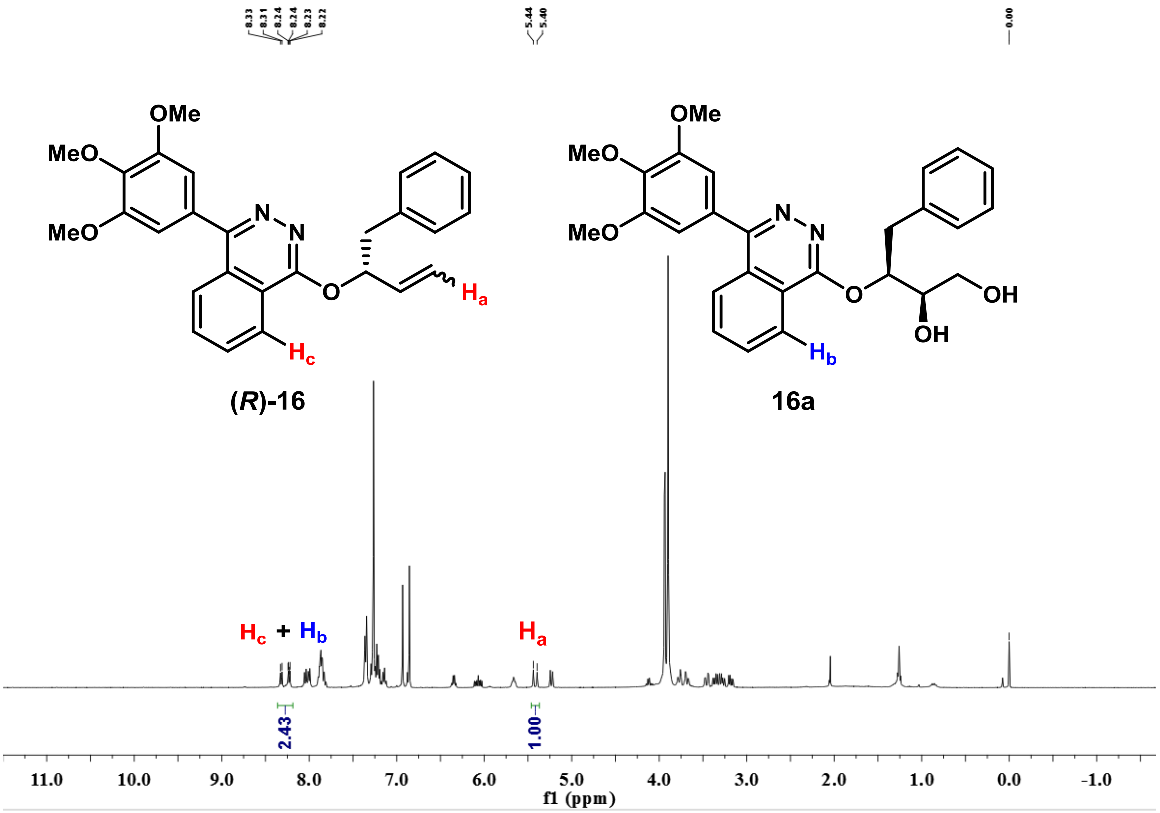

Supplementary Figure 102. ^{1}H NMR spectrum of crude mixture of compound **(*R*)-16** and **16a.**

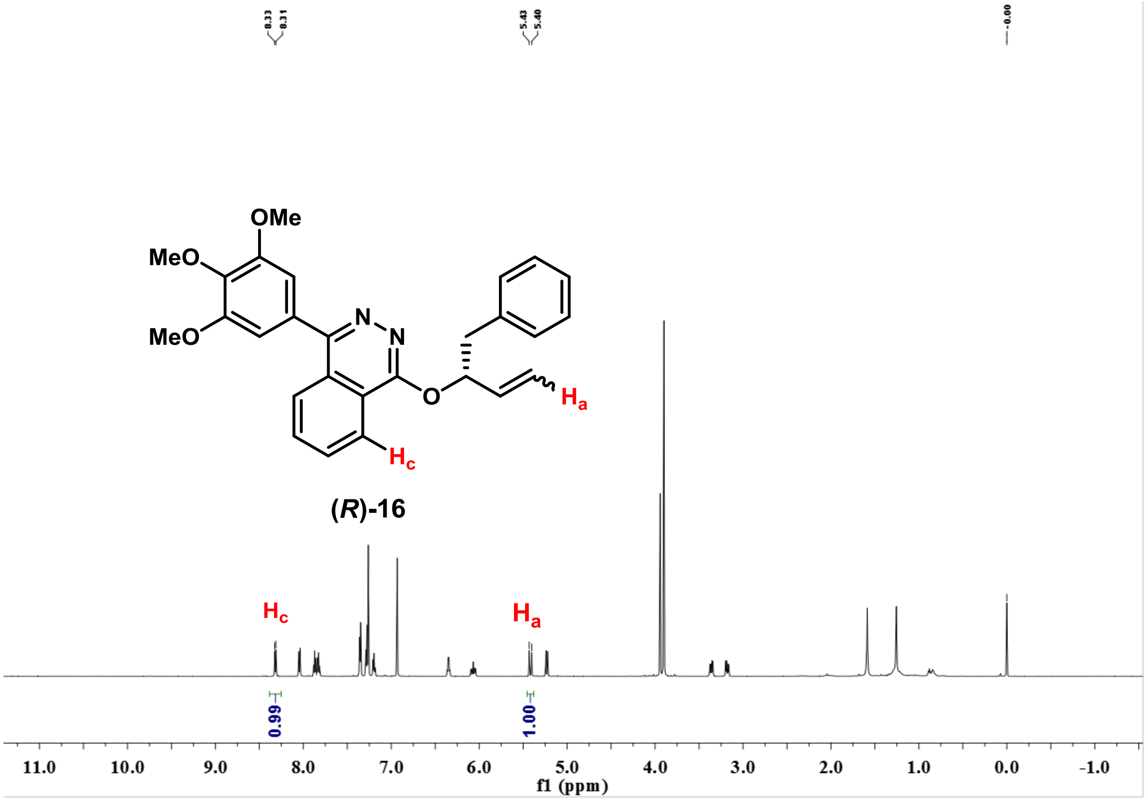

Supplementary Figure 103. ^{1}H NMR spectrum of recovered alkene **(*R*)-16**.

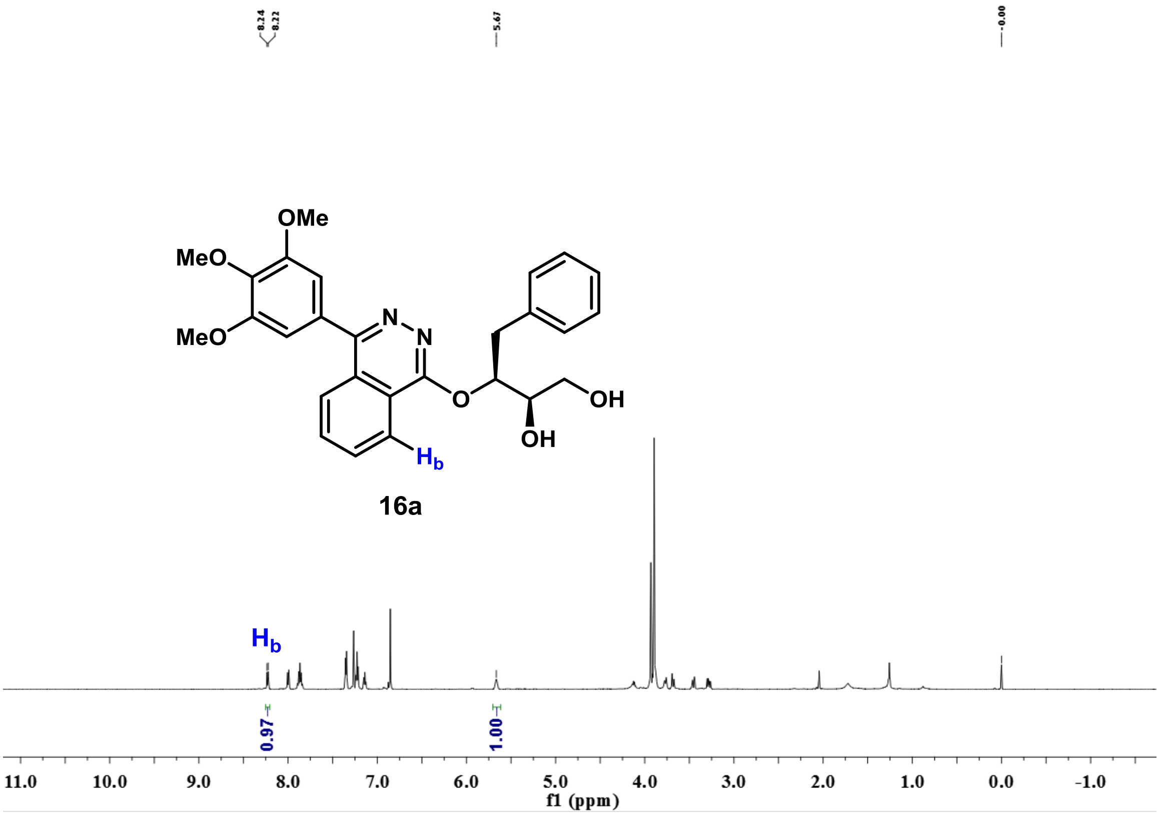

Supplementary Figure 104. ^{1}H NMR spectrum of dihydroxylated product **16a**.

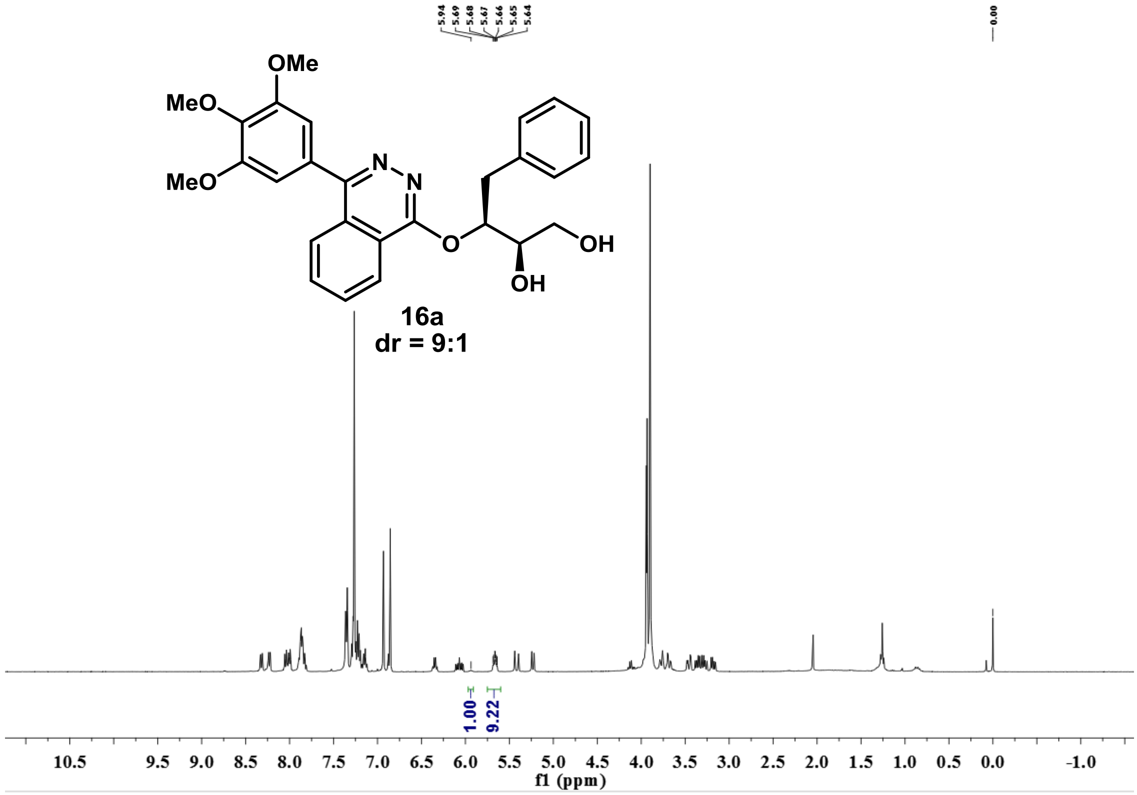

Supplementary Figure 105. ^{1}H NMR spectrum of crude mixture for diastereomeric ratio (dr).

HPLC (OD-H, 0.46*25 cm, 5µm, hexane/isopropanol = 90/10, flow = 1.0 mL/min, detection at 210 nm), retention time = 15.813 min (major) and 19.760 min (minor).

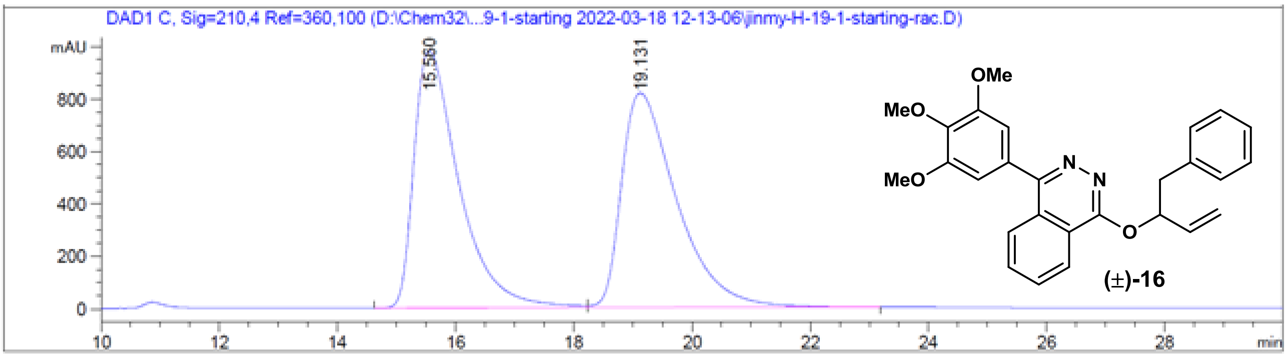

Signal 2: DAD1 C, Sig=210,4 Ref=360,100

Peak #	RetTime [min]	Type	Width [min]	Area [mAU*s]	Height [mAU]	Area %
1	15.560	BV	0.7929	5.20831e4	981.73993	49.5746
2	19.131	VB	0.9586	5.29770e4	819.11481	50.4254

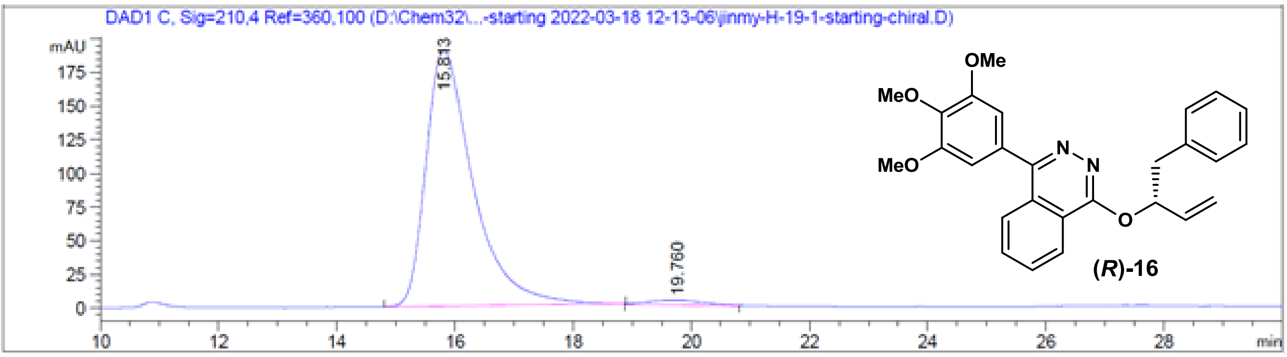

Signal 2: DAD1 C, Sig=210,4 Ref=360,100

Peak #	RetTime [min]	Type	Width [min]	Area [mAU*s]	Height [mAU]	Area %
1	15.813	BB	0.8229	1.05564e4	190.37024	97.8931
2	19.760	MM R	1.0368	227.19533	3.65214	2.1069

Supplementary Figure 106. HPLC chromatogram for **(*R*)-16**.

HPLC (OD-H, 0.46*25 cm, 5μm, hexane/isopropanol = 80/20, flow = 1.0 mL/min, detection at 230 nm), retention time = 14.991 min (minor) and 17.100 min (major).

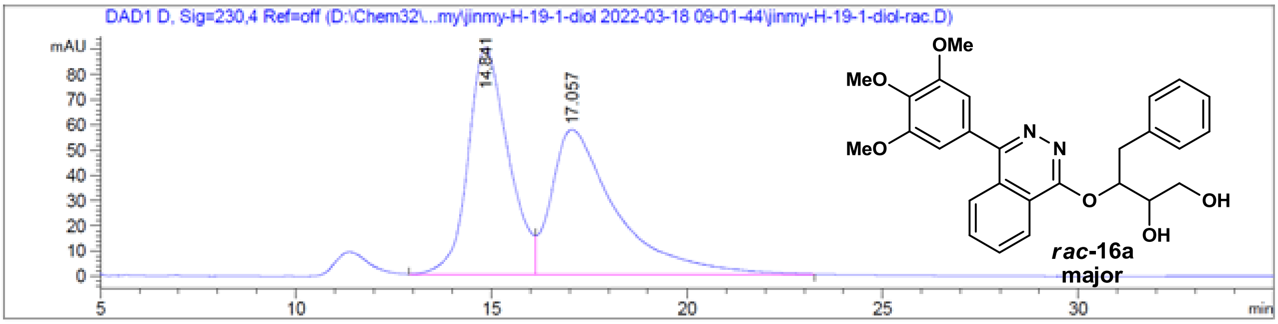

Signal 4: DAD1 D, Sig=230,4 Ref=off

Peak #	RetTime [min]	Type	Width [min]	Area [mAU*s]	Height [mAU]	Area %
1	14.841	BV	1.0390	6411.12549	89.78922	49.3094
2	17.057	VB	1.5690	6590.70801	57.25195	50.6906

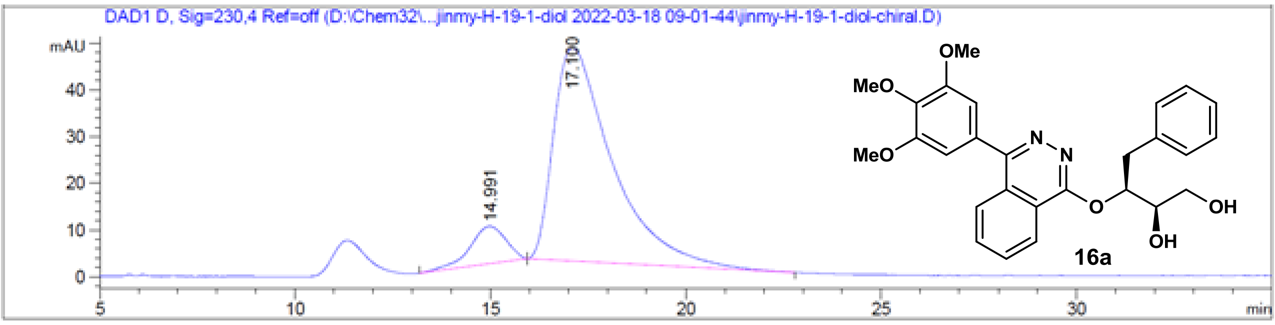

Signal 4: DAD1 D, Sig=230,4 Ref=off

Peak #	RetTime [min]	Type	Width [min]	Area [mAU*s]	Height [mAU]	Area %
1	14.991	BB	0.7675	514.47980	7.96534	9.7484
2	17.100	BB	1.3931	4763.08008	45.58289	90.2516

Supplementary Figure 107. HPLC chromatogram for **16a**.

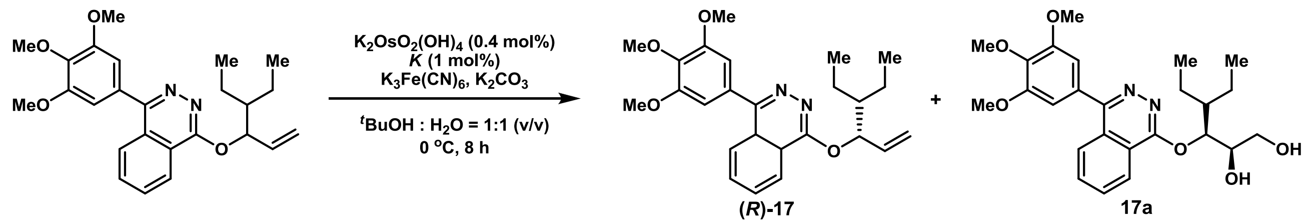

(±)-17

(*R*)-17
s = 20, conv = 51%
ee = 81%, 48% yield

17a
dr = 20:1, 81% ee
48% yield

The general procedure **IV** was followed. The conversion of **(±)-17** was determined by crude ^{1}H NMR. **Conversion** (%) = [(2.05 – 1.00) / 2.05] % = 51%.

S = In [(1 – conv)(1 – ee)] / In [(1 – conv)(1 + ee)] = 20.

The recovered alkene **(*R*)-17** (20.3 mg, 48% yield, 81% ee) was purified by chromatography on silica gel (eluted with petroleum ether : ethyl acetate = 3:1). $[\alpha]_D^{25}$ = –11.3 (*c* 0.81, $CHCl_3$).

The dihydroxylated product **17a** (dr = 20:1, 21.9 mg, 48% yield, 81% ee) was purified by chromatography on silica gel (eluted with petroleum ether : ethyl acetate = 1:2). $[\alpha]_D^{25}$ = – 25.14 (*c* .0.74, $CHCl_3$)

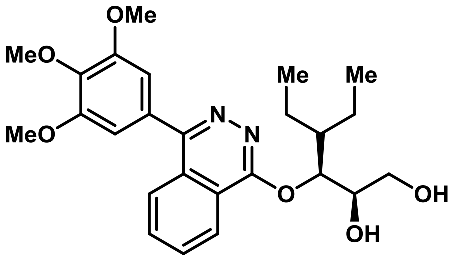

17a

(2*R*,3*S*)-4-ethyl-3-(4-(3,4,5-trimethoxyphenyl)phthalazin-1-yloxy)hexane-1,2-diol

^{1}H NMR (600 MHz, $CDCl_3$): δ 8.29 (d, *J* = 6.0 Hz, 1H), 8.07 (d, *J* = 6.0 Hz, 1H), 7.95 – 7.87 (m, 2H), 6.91 (s, 2H), 5.54 (d, *J* = 12.0 Hz, 1H), 4.65 – 4.40 (m, 1H), 3.97 (d, *J* = 12.0 Hz, 1H), 3.95 (s, 3H), 3.92 (s, 6H), 2.05 – 1.87 (m, 2H), 1.70 – 1.55 (m, 1H), 1.37 – 1.30 (m, 1H), 1.09 (t, *J* = 6.0 Hz, 3H), 0.90 (t, *J* = 6.0 Hz, 3H) ppm.

^{13}C NMR (151 MHz, $CDCl_3$): δ 161.3, 157.0, 153.4, 139.0, 132.9, 132.2, 131.3, 128.2, 126.5, 123.4, 120.4, 107.2, 78.9, 70.5, 62.3, 61.0, 56.3, 42.2, 29.7, 23.2, 21.2, 12.2, 11.8 ppm.

HRMS (ESI) m/z: $[M + H]^+$ Calcd for $C_{25}H_{33}N_2O_6$ 457.2333; Found 457.2327.

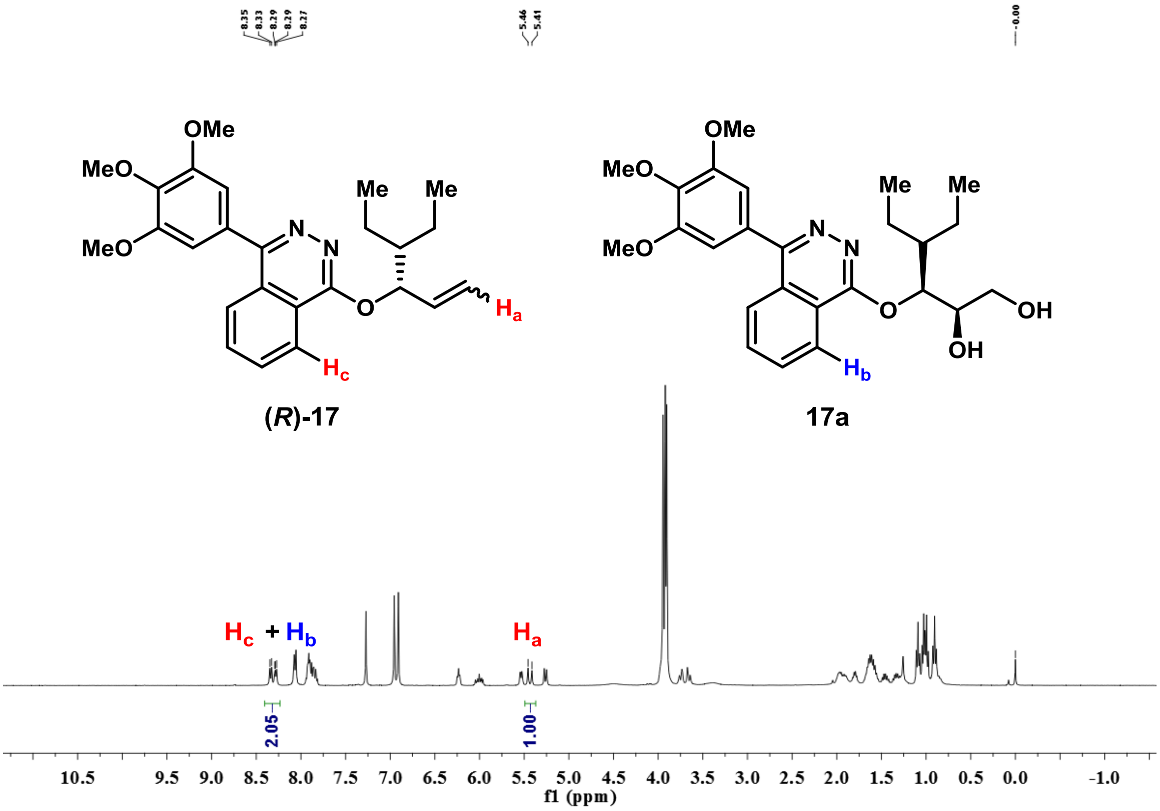

Supplementary Figure 108. ^{1}H NMR spectrum of crude mixture of compound **(*R*)-17** and **17a.**

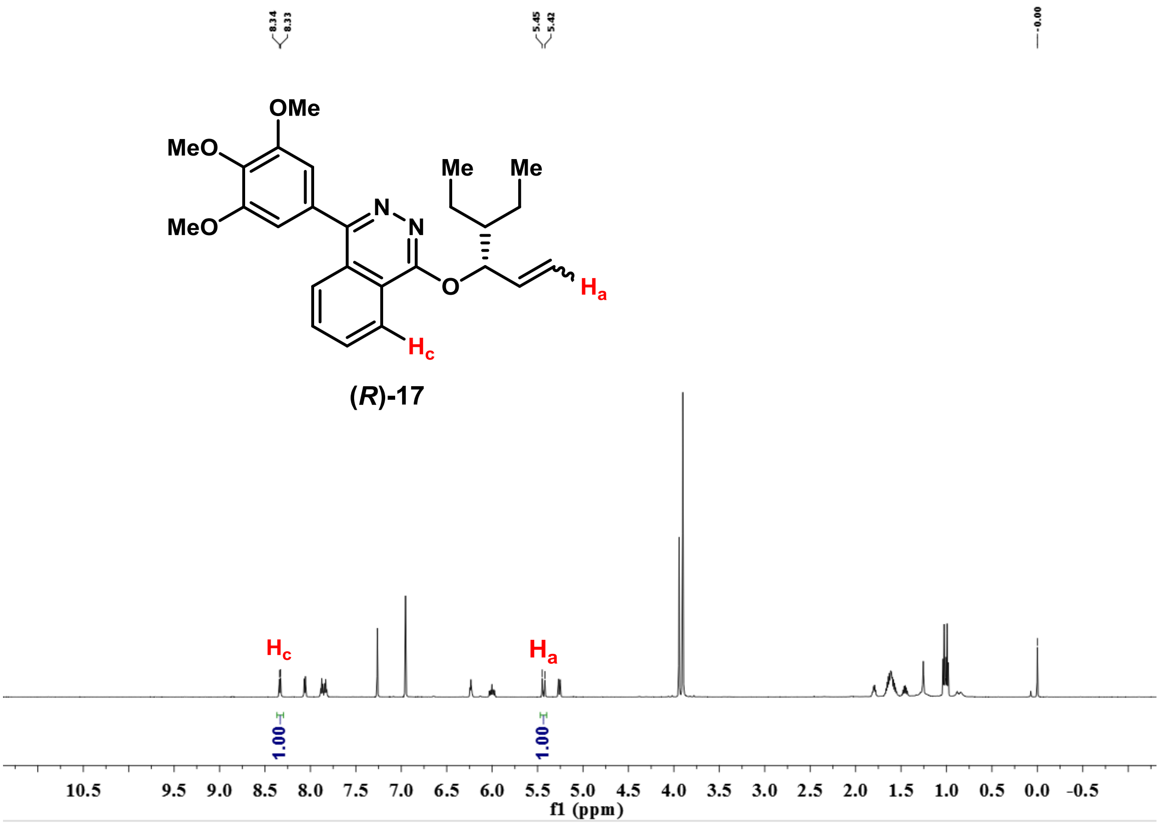

Supplementary Figure 109. ^{1}H NMR spectrum of recovered alkene **(*R*)-17**.

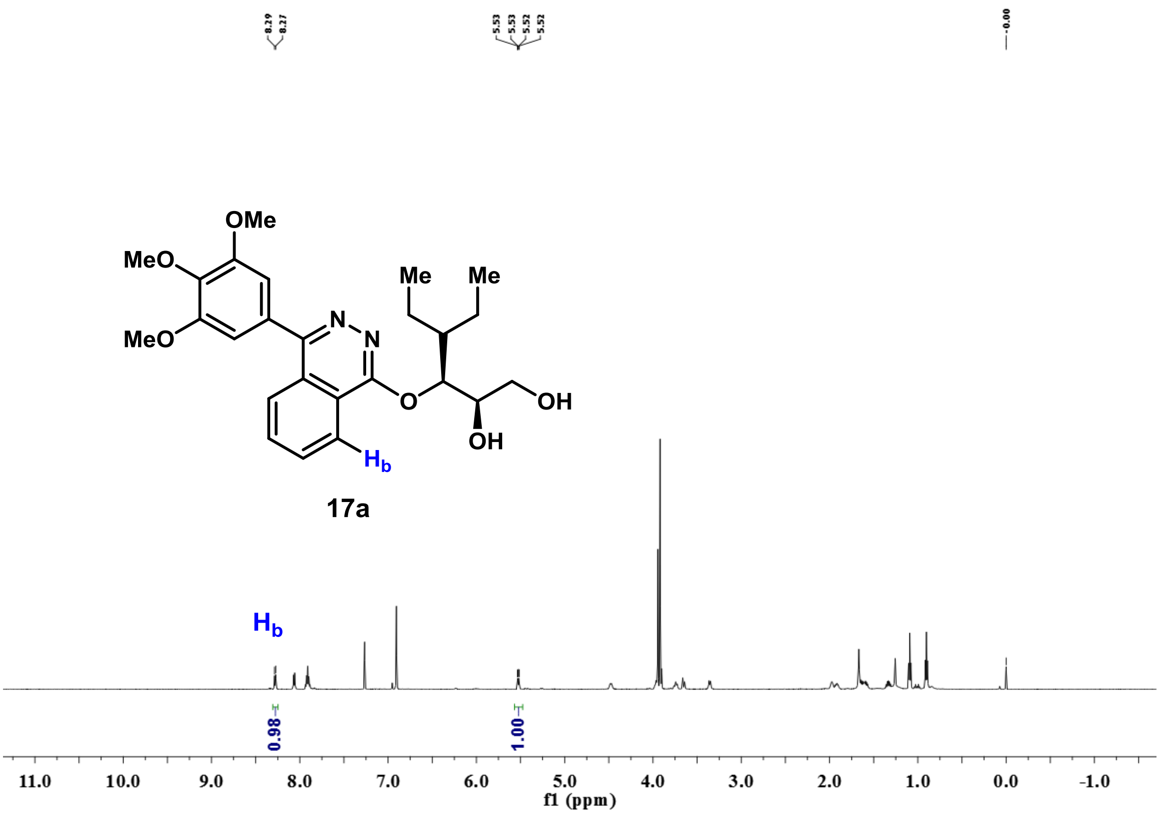

Supplementary Figure 110. ^{1}H NMR spectrum of dihydroxylated product **17a**.

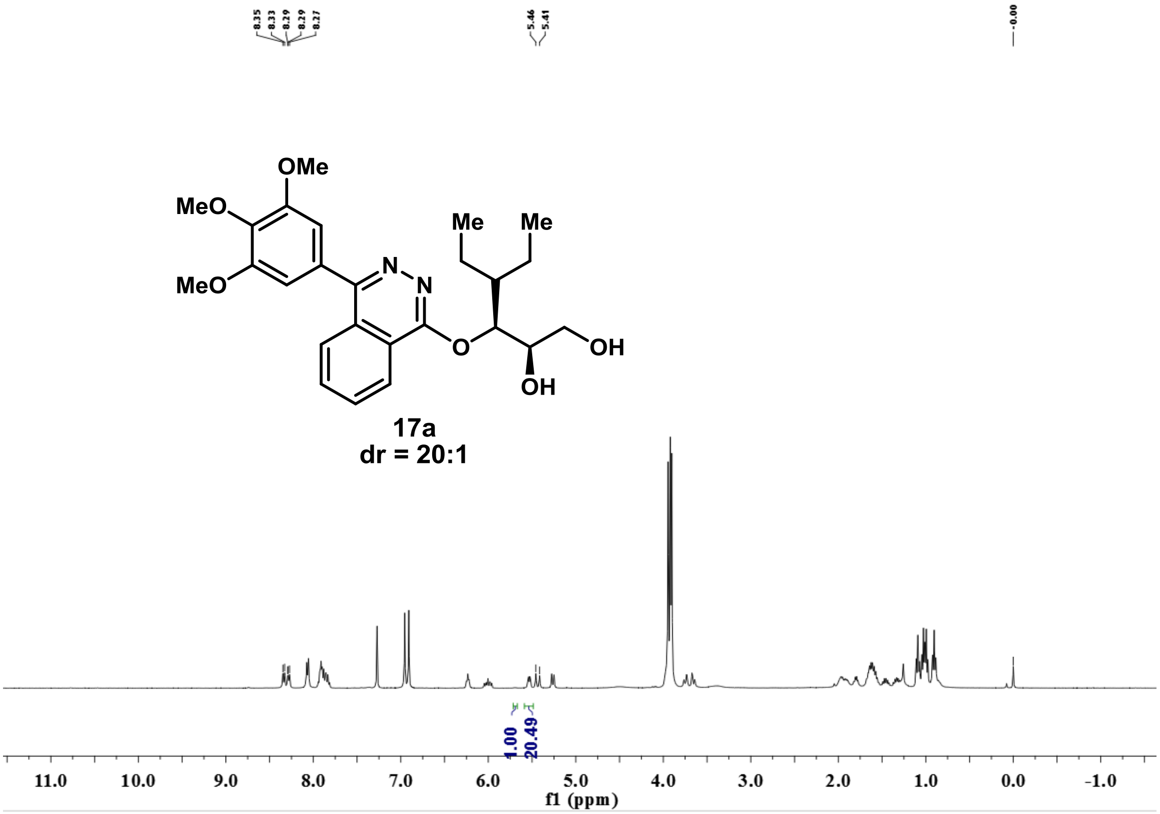

Supplementary Figure 111. ^{1}H NMR spectrum of crude mixture for diastereomeric ratio (dr).

HPLC (AD-H, 0.46*25 cm, 5μm, hexane/isopropanol = 90/10, flow = 1.0 mL/min, detection at 254, nm) retention time = 14.096 min (minor) and 15.258 min (major).

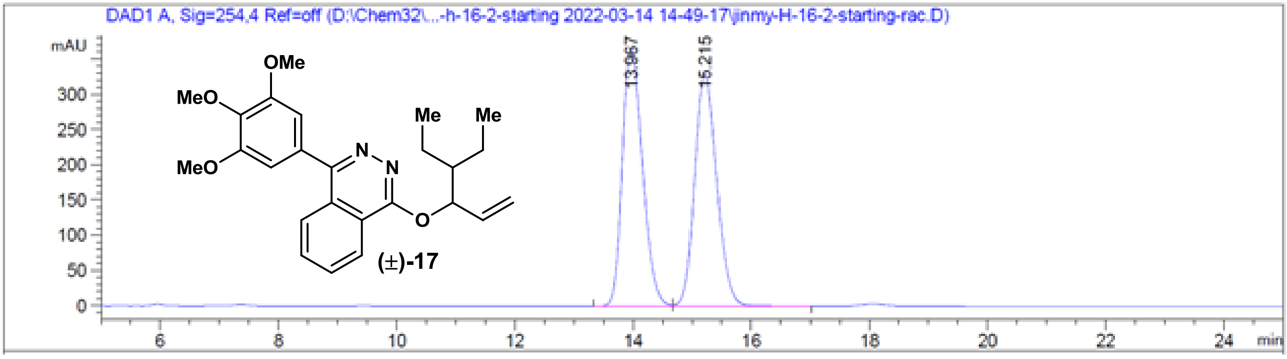

Signal 1: DAD1 A, Sig=254,4 Ref=off

Peak #	RetTime [min]	Type	Width [min]	Area [mAU*s]	Height [mAU]	Area %
1	13.967	BV	0.3677	8646.91602	365.92319	50.1092
2	15.215	VB	0.4071	8609.23730	329.15839	49.8908

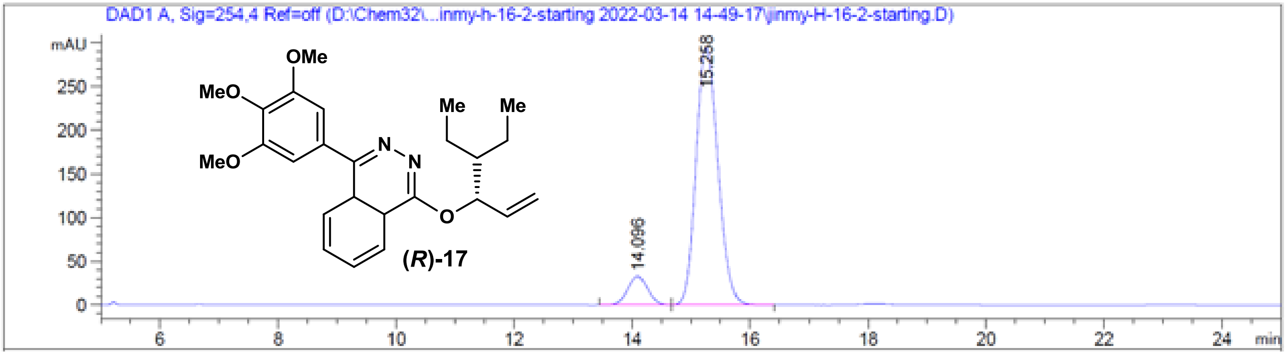

Signal 1: DAD1 A, Sig=254,4 Ref=off

Peak #	RetTime [min]	Type	Width [min]	Area [mAU*s]	Height [mAU]	Area %
1	14.096	BV	0.3670	778.08771	32.76554	9.3173
2	15.258	VB	0.4003	7572.93408	294.16119	90.6827

Supplementary Figure 112. HPLC chromatogram for **(*R*)-17**.

HPLC (AD-H, 0.46*25 cm, 5μm, hexane/isopropanol = 90/10, flow = 1.0 mL/min, detection at 210, nm) retention time = 14.109 min (major) and 17.498 min (minor).

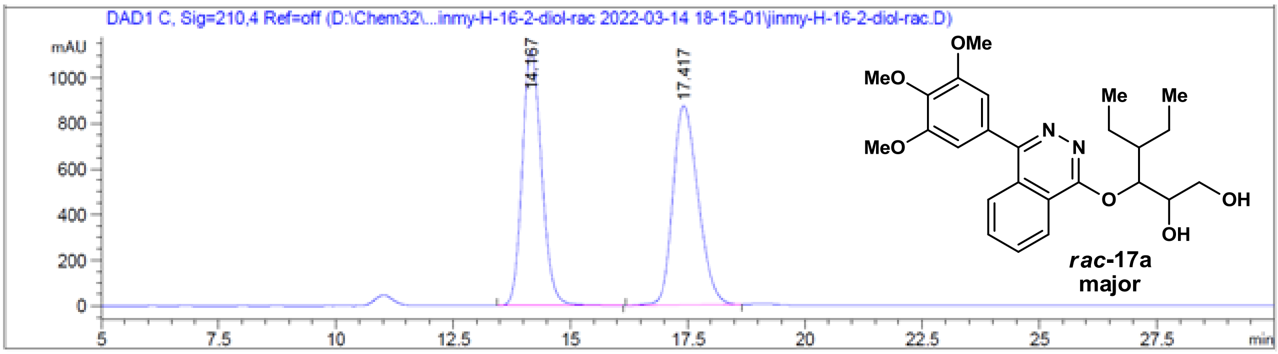

Signal 2: DAD1 C, Sig=210,4 Ref=off

Peak #	RetTime [min]	Type	Width [min]	Area [mAU*s]	Height [mAU]	Area %
1	14.167	BB	0.4506	3.23365e4	1120.84375	49.8312
2	17.417	BB	0.5782	3.25556e4	874.80017	50.1688

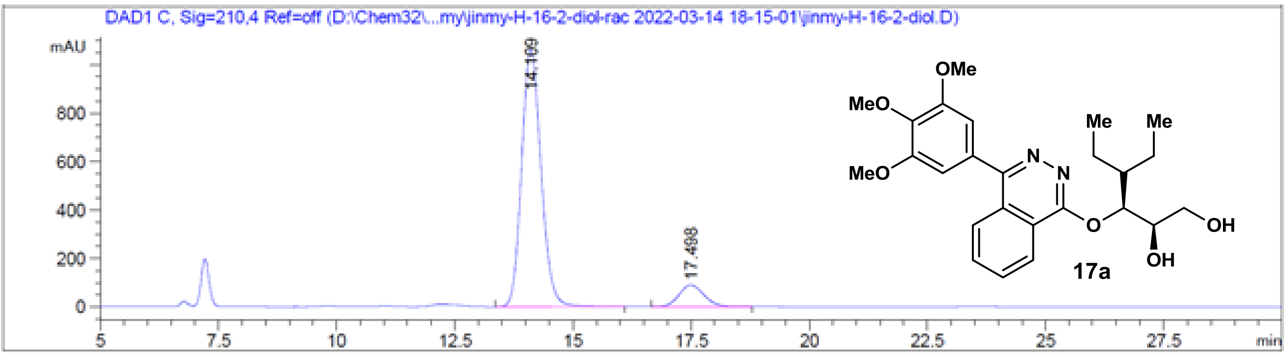

Signal 2: DAD1 C, Sig=210,4 Ref=off

Peak #	RetTime [min]	Type	Width [min]	Area [mAU*s]	Height [mAU]	Area %
1	14.109	BB	0.4457	3.05539e4	1061.86536	90.2322
2	17.498	BB	0.5664	3307.50293	90.06838	9.7678

Supplementary Figure 113. HPLC chromatogram for **17a**.

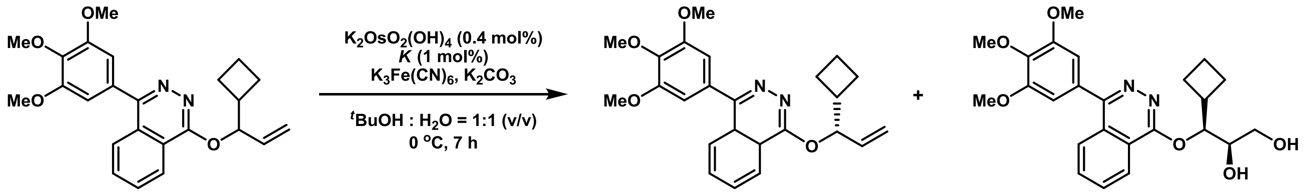

(±)-18

(*R*)-18
s = 11, conv = 59%
ee = 86%, 40% yield

18a
dr = 7:1, 75% ee
56% yield

The general procedure **IV** was followed. The conversion of **(±)-18** was determined by crude ^{1}H NMR. **Conversion** (%) = [(2.46 – 1.00) / 1.46] % = 59%.

S = In [(1 – conv)(1 – ee)] / In [(1 – conv)(1 + ee)] = 11.

The recovered alkene **(*R*)-18** (16.3 mg, 40% yield, 86% ee) was purified by chromatography on silica gel (eluted with petroleum ether : ethyl acetate = 3:1). $[\alpha]_D^{25}$ = –11.3 (*c* 0.81, $CHCl_3$).

The dihydroxylated product **18a** (dr = 7:1, 22.9 mg, 56% yield, 75% ee) was purified by chromatography on silica gel (eluted with petroleum ether : ethyl acetate = 1:2). $[\alpha]_D^{25}$ = – 20.91(*c* 0.55, $CHCl_3$)

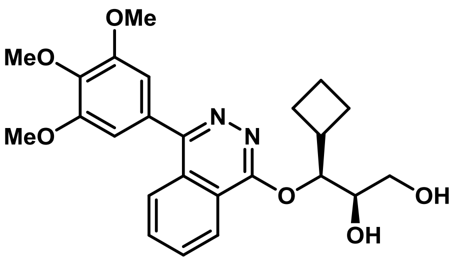

18a

(2*R*,3*S*)-3-cyclobutyl-3-(4-(3,4,5-trimethoxyphenyl)phthalazin-1-yloxy)propane-1,2-diol

^{1}H NMR (600 MHz, $CDCl_3$): δ 8.37 (d, *J* = 6.0 Hz, 1H), 8.07 (d, *J* = 6.0 Hz, 1H), 7.97 – 7.89 (m, 2H), 6.90 (s, 2H), 5.32 (t, *J* = 6.0 Hz, 1H), 4.36 – 4.27 (m, 1H), 3.95 (s, 3H), 3.92 (s, 6H), 3.82 – 3.76 (m, 1H), 3.75 – 3.68 (m, 1H), 3.67 – 3.60 (m, 1H), 3.14 – 3.04 (m, 1H), 2.24 – 2.15 (m, 2H), 2.14 – 2.05 (m, 1H), 2.04 – 1.96 (m, 2H) ppm.

^{13}C NMR (151 MHz, $CDCl_3$): δ 161.2, 157.3, 153.4, 139.0, 132.9, 132.3, 131.3, 128.3, 126.5, 123.4, 120.5, 107.2, 107.15, 82.5, 72.0, 62.5, 61.0, 56.3, 37.0, 29.7, 24.8, 24.0, 18.3 ppm.

HRMS (ESI) m/z: $[M + H]^+$ Calcd for $C_{24}H_{29}N_2O_6$ 441.2020; Found 441.2014.

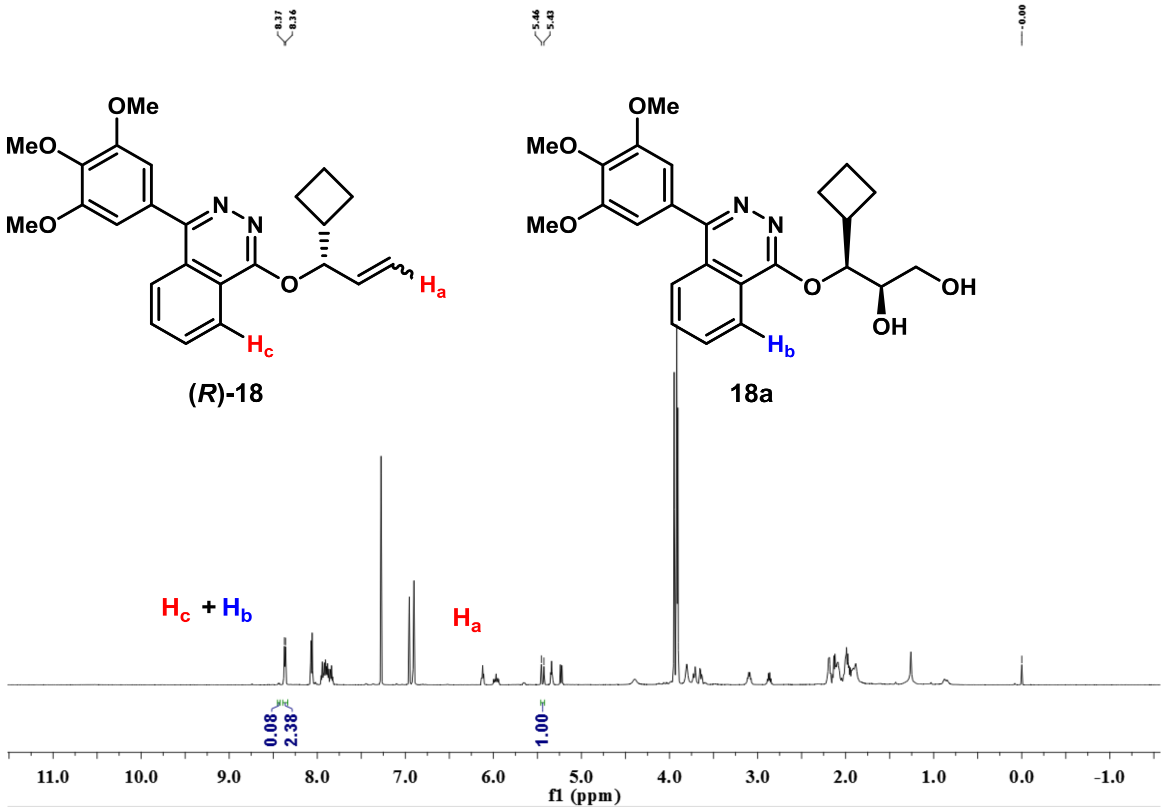

Supplementary Figure 114. ^{1}H NMR spectrum of crude mixture of compound **(*R*)-18** and **18a.**

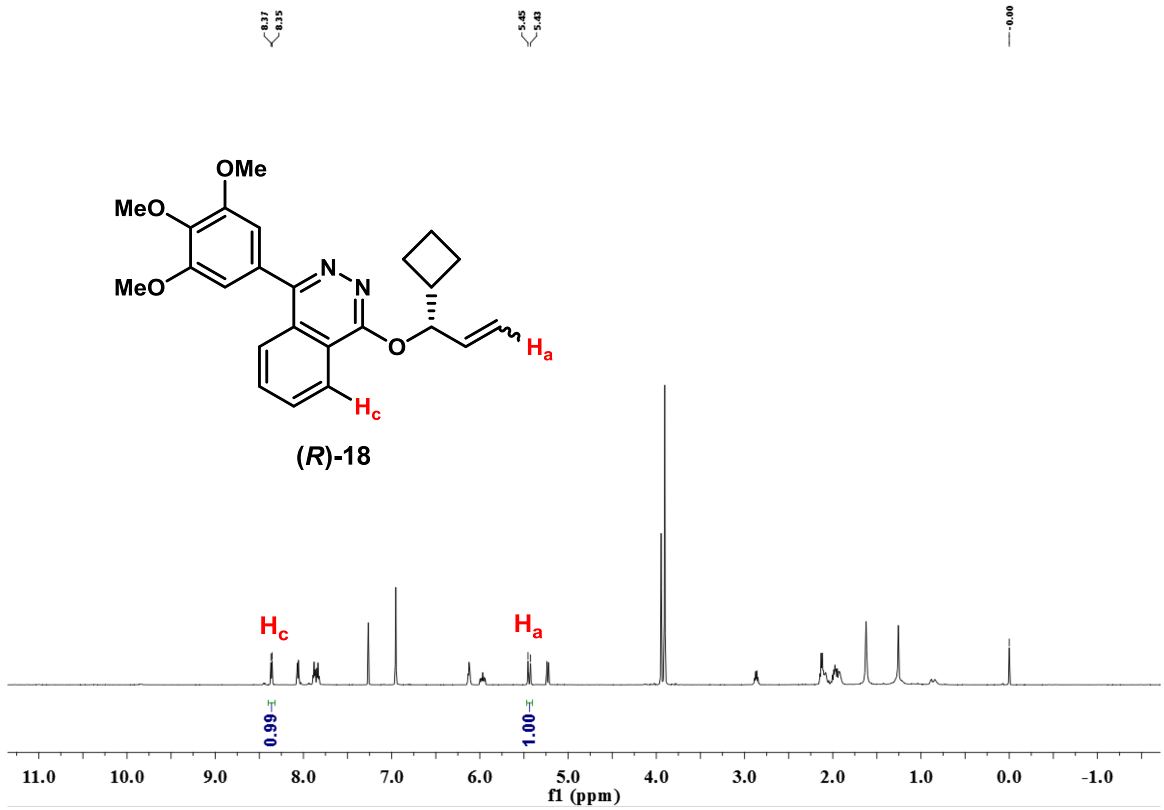

Supplementary Figure 115. ^{1}H NMR spectrum of recovered alkene **(*R*)-18**.

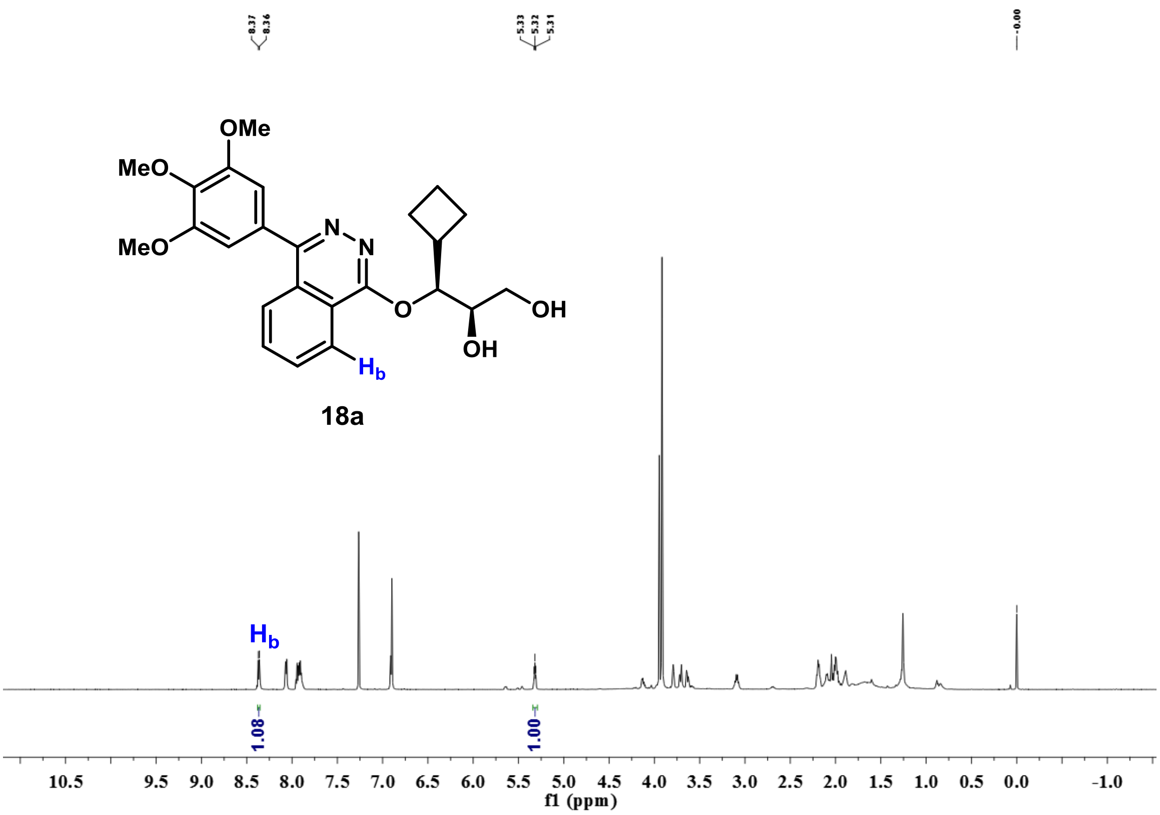

Supplementary Figure 116. ^{1}H NMR spectrum of dihydroxylated product **18a**.

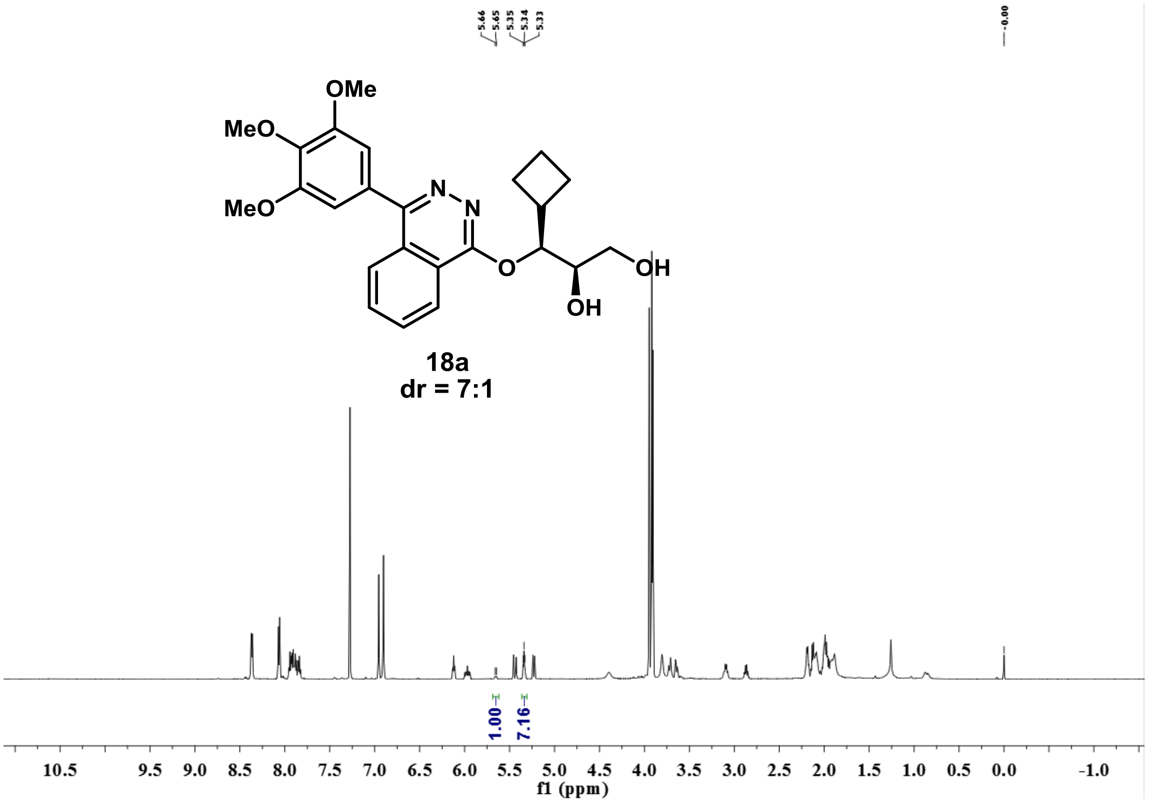

Supplementary Figure 117. ^{1}H NMR spectrum of crude mixture for diastereomeric ratio (dr).

HPLC (AD-H, 0.46*25 cm, 5μm, hexane/isopropanol = 90/10, flow = 1.0 mL/min, detection at 210 nm), retention time = 18.073 min (minor) and 20.406 min (major).

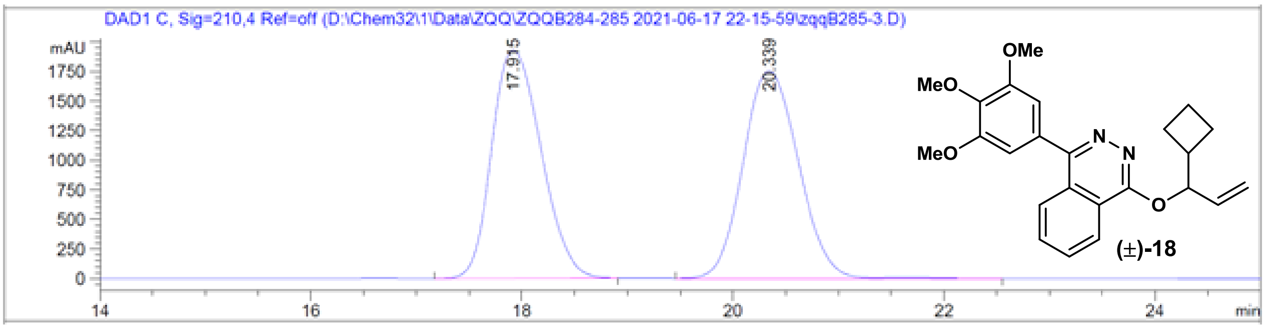

Signal 2: DAD1 C, Sig=210,4 Ref=off

Peak #	RetTime [min]	Type	Width [min]	Area [mAU*s]	Height [mAU]	Area %
1	17.915	BB	0.5062	6.26052e4	1931.04211	49.4076
2	20.339	BV R	0.5729	6.41066e4	1752.29663	50.5924

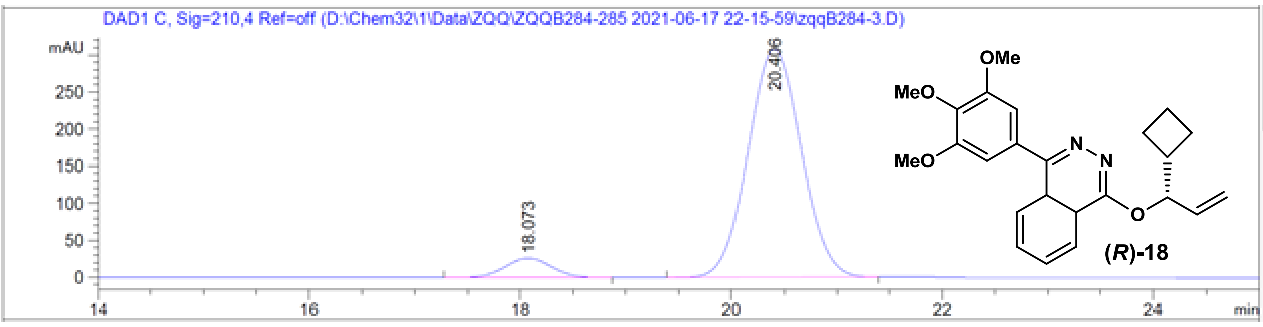

Signal 2: DAD1 C, Sig=210,4 Ref=off

Peak #	RetTime [min]	Type	Width [min]	Area [mAU*s]	Height [mAU]	Area %
1	18.073	BB	0.4715	805.74225	26.44509	6.9405
2	20.406	BB	0.5468	1.08035e4	308.33533	93.0595

Supplementary Figure 118. HPLC chromatogram for ***(R)*-18**.

HPLC (AD-H, 0.46*25 cm, 5μm, hexane/isopropanol = 80/20, flow = 1.0 mL/min, detection at 210 nm), retention time = 25.136 min (major) and 28.211 min (minor).

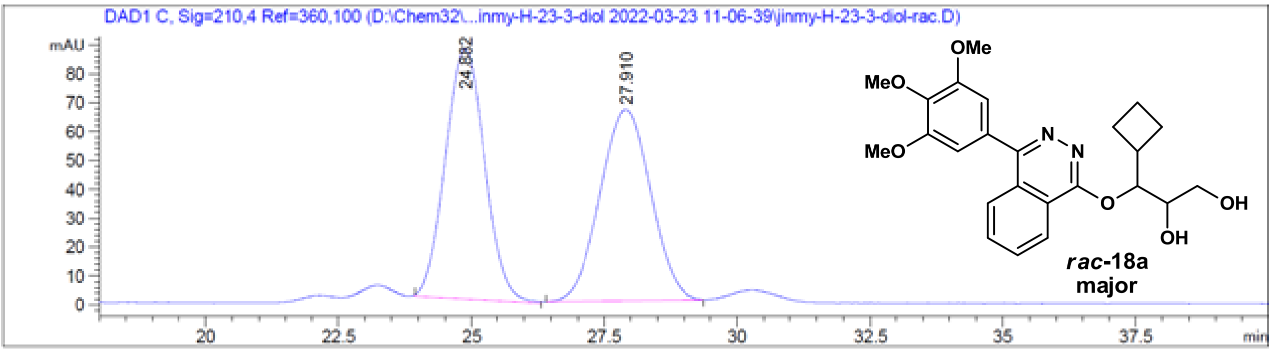

Signal 2: DAD1 C, Sig=210,4 Ref=360,100

Peak #	RetTime [min]	Type	Width [min]	Area [mAU*s]	Height [mAU]	Area %
1	24.882	MM R	0.8603	4473.77588	86.67079	50.5257
2	27.910	BB	0.8536	4380.68799	66.23751	49.4743

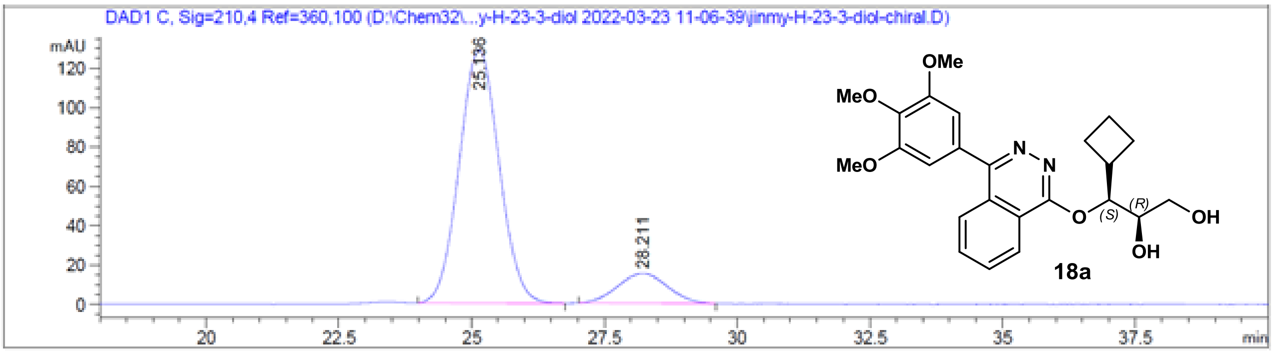

Signal 2: DAD1 C, Sig=210,4 Ref=360,100

Peak #	RetTime [min]	Type	Width [min]	Area [mAU*s]	Height [mAU]	Area %
1	25.136	BB	0.7905	6736.81543	128.73050	87.2001
2	28.211	BB	0.7803	988.88452	15.05566	12.7999

Supplementary Figure 119. HPLC chromatogram for **18a**.

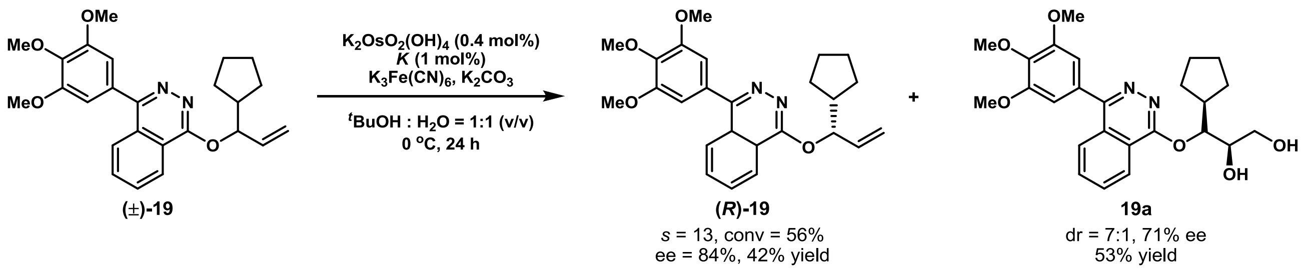

The general procedure **IV** was followed. The conversion of **(±)-19** was determined by crude ^{1}H NMR. **Conversion** (%) = [(2.27 – 1.00) / 2.27] % = 56%.

S = In [(1 – conv)(1 – ee)] / In [(1 – conv)(1 + ee)] = 13.

The recovered alkene **(*R*)-19** (17.6 mg, 42% yield, 84% ee) was purified by chromatography on silica gel (eluted with petroleum ether : ethyl acetate = 3:1). $[\alpha]_D^{25}$ = –11.3 (*c* 0.81, $CHCl_3$).

The dihydroxylated product **19a** (dr = 7:1, 24.1 mg, 53% yield, 71% ee) was purified by chromatography on silica gel (eluted with petroleum ether : ethyl acetate = 1:2). $[\alpha]_D^{25}$ = – 18.00 (*c* 0.80, $CHCl_3$)

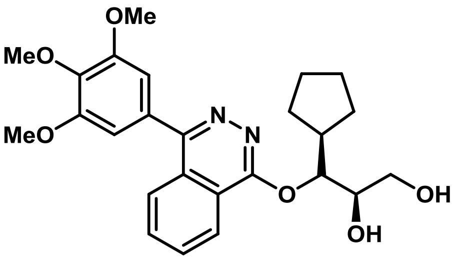

19a

(2*R*,3*R*)-3-cyclopentyl-3-(4-(3,4,5-trimethoxyphenyl)phthalazin-1-yloxy)propane-1,2-diol

^{1}H NMR (600 MHz, $CDCl_3$): δ 8.31 (d, *J* = 6.0 Hz, 1H), 8.06 (d, *J* = 6.0 Hz, 1H), 7.96 – 7.87 (m, 2H), 6.89 (s, 2H), 5.36 (t, *J* = 6.0 Hz, 1H), 4.40 – 4.15 (m, 1H), 3.94 (s, 3H), 3.91 (s, 6H), 3.85 – 3.75 (m, 2H), 3.70 – 3.60 (m, 2H), 2.70 – 2.60 (m, 1H), 2.10 – 1.98 (m, 1H), 1.89 – 1.83 (m, 2H), 1.76 – 1.60 (m, 1H), 1.44 – 1.36 (m, 1H), 0.94 – 0.78 (m, 1H) ppm.

^{13}C NMR (151 MHz, $CDCl_3$): δ 161.3, 157.2, 153.4, 139.0, 132.9, 132.3, 131.3, 128.3, 126.5, 123.4, 120.4, 107.1, 82.6, 72.9, 62.4, 61.0, 56.3, 41.4, 29.7, 29.6, 27.8, 25.8, 25.7 ppm.

HRMS (ESI) m/z: $[M + H]^+$ Calcd for $C_{25}H_{31}N_2O_6$ 455.2177; Found 455.2170.

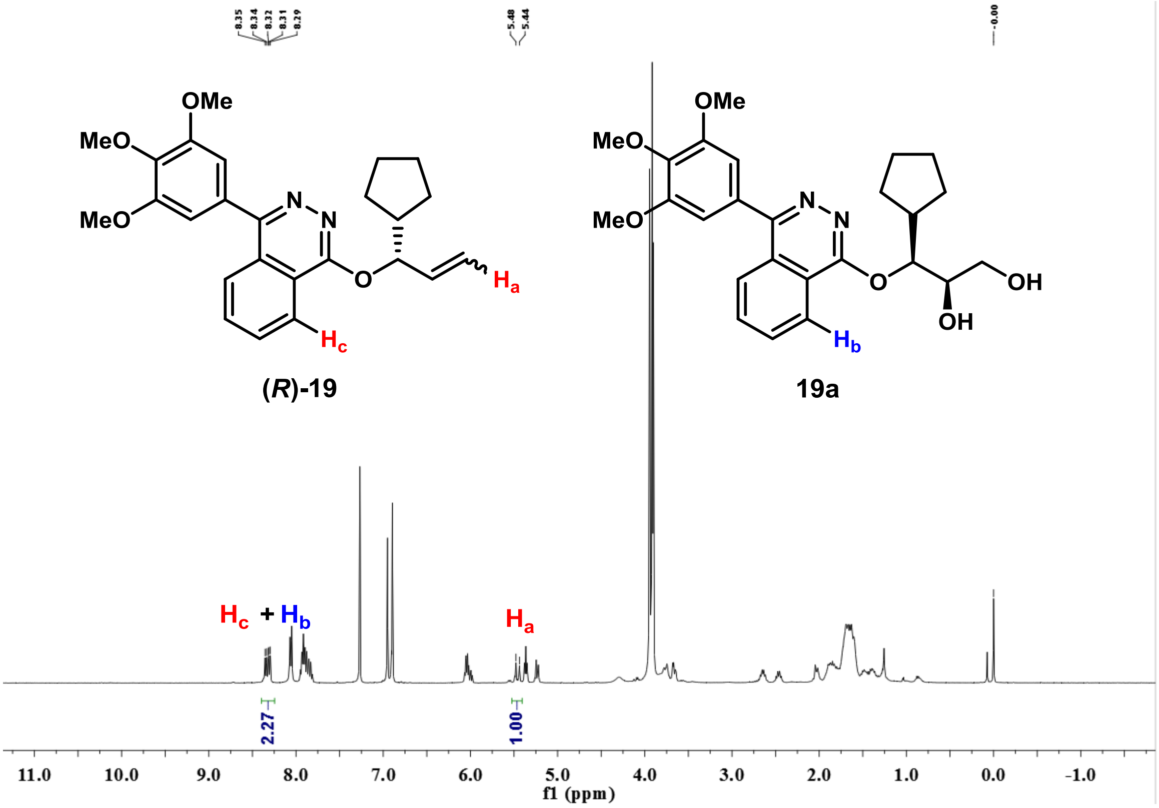

Supplementary Figure 120. ^{1}H NMR spectrum of crude mixture of compound **(*R*)-19** and **19a.**

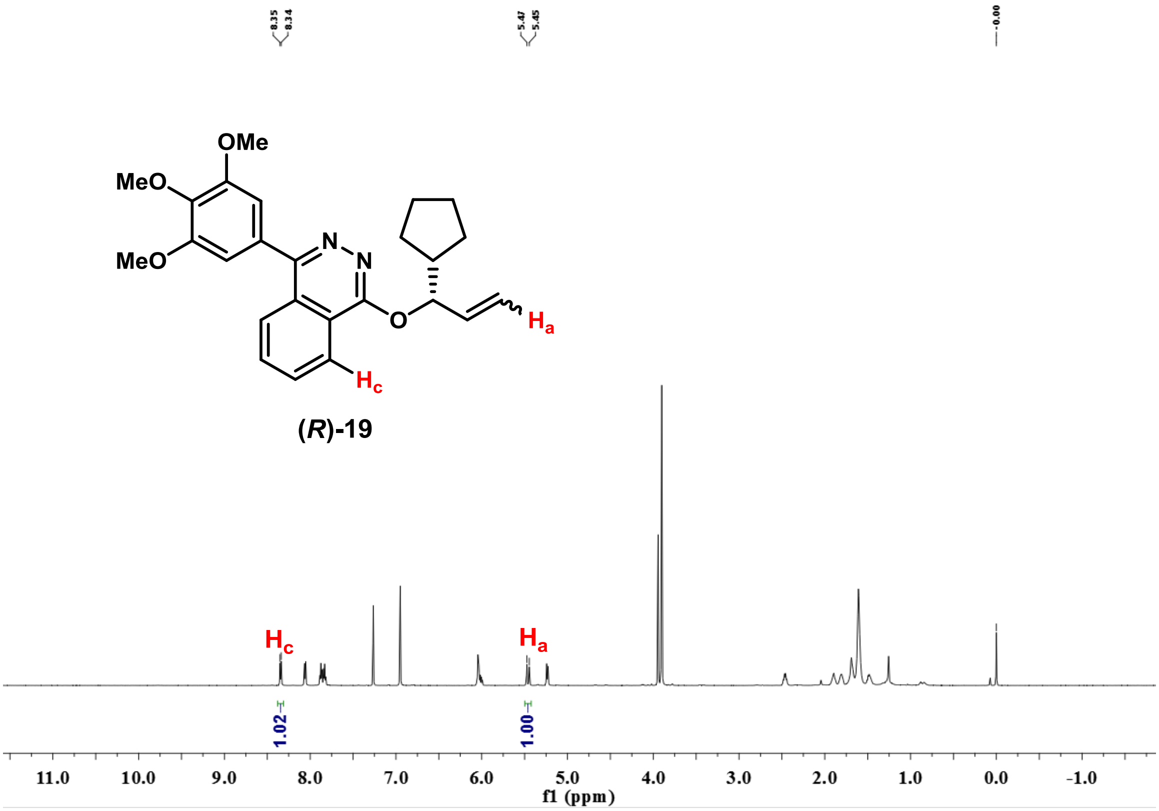

Supplementary Figure 121. ^{1}H NMR spectrum of recovered alkene **(*R*)-19**.

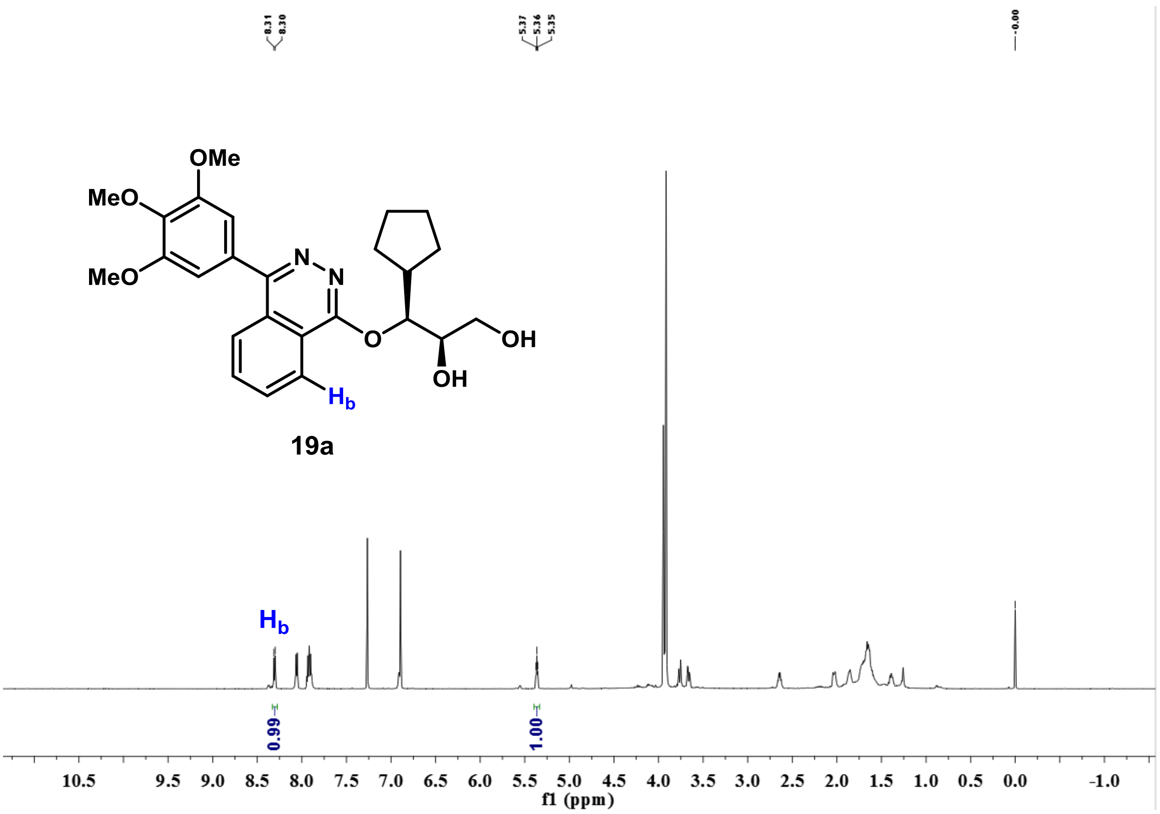

Supplementary Figure 122. ^{1}H NMR spectrum of dihydroxylated product **19a**.

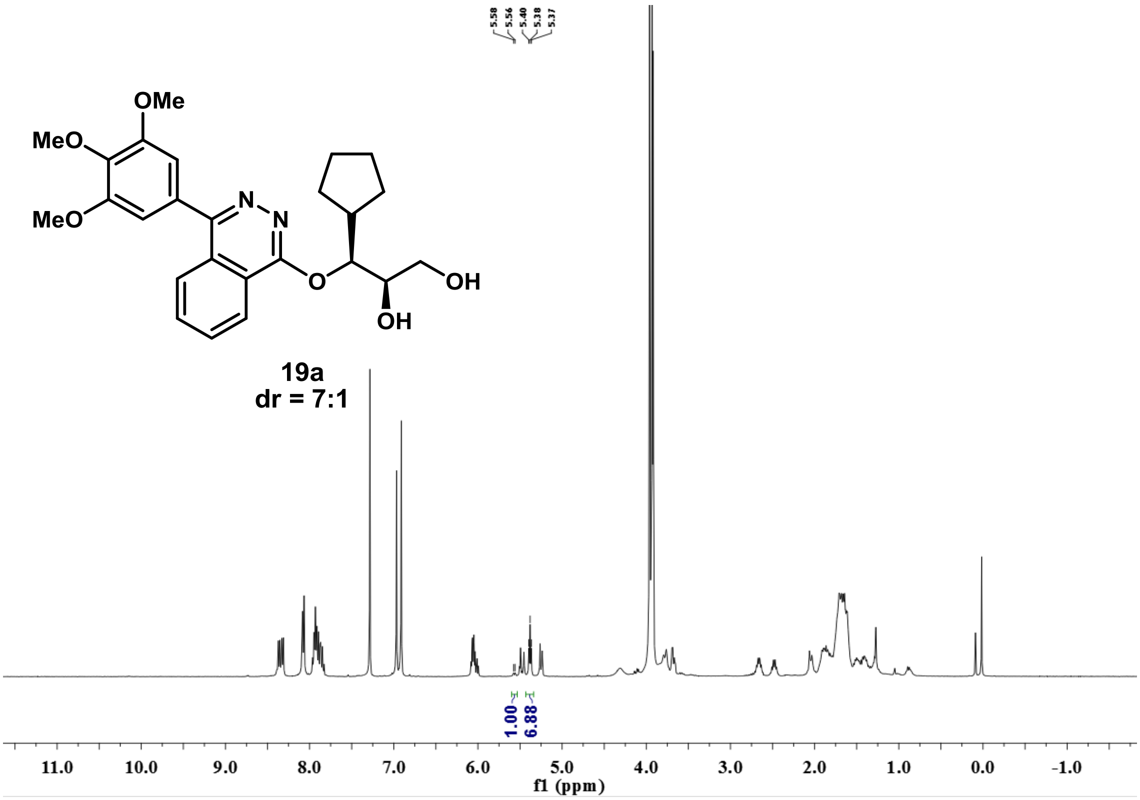

Supplementary Figure 123. ^{1}H NMR spectrum of crude mixture for diastereomeric ratio (dr).

HPLC (AD-H, 0.46*25 cm, 5μm, hexane/isopropanol = 90/10, flow = 1.0 mL/min, detection at 210 nm), retention time = 17.301 min (minor) and 25.421 min (major).

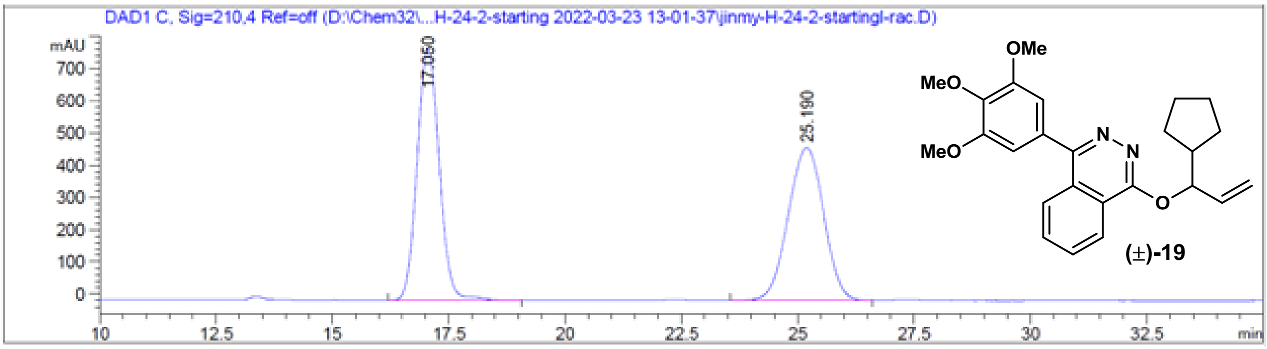

Signal 2: DAD1 C, Sig=210,4 Ref=off

Peak #	RetTime [min]	Type	Width [min]	Area [mAU*s]	Height [mAU]	Area %
1	17.050	BB	0.5095	2.56785e4	781.27002	50.5108
2	25.190	BB	0.8327	2.51591e4	474.44846	49.4892

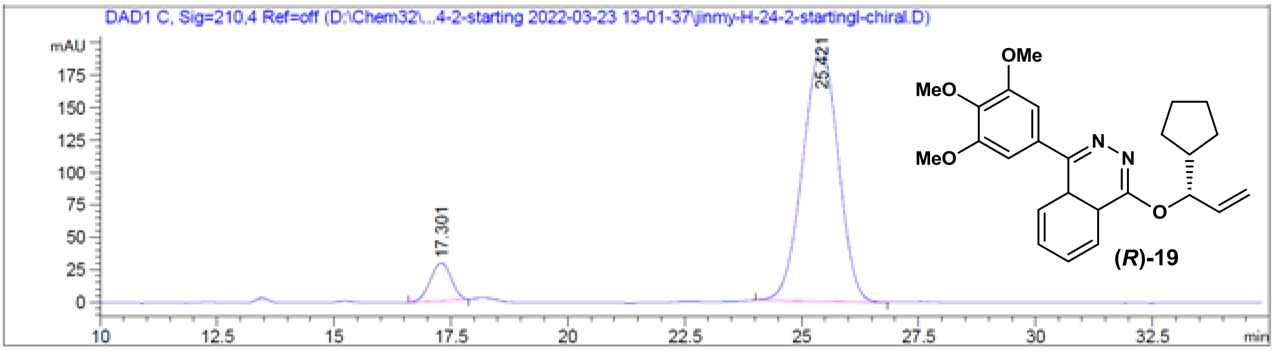

Signal 2: DAD1 C, Sig=210,4 Ref=off

Peak #	RetTime [min]	Type	Width [min]	Area [mAU*s]	Height [mAU]	Area %
1	17.301	BB	0.4788	906.47314	29.15195	8.1018
2	25.421	BB	0.7947	1.02821e4	194.52328	91.8982

Supplementary Figure 124. HPLC chromatogram for ***(R)*-19**.

HPLC (AD-H, 0.46*25 cm, 5μm, hexane/isopropanol = 80/20, flow = 1.0 mL/min, detection at 210 nm), retention time = 30.207 min (minor) and 32.528 min (major).

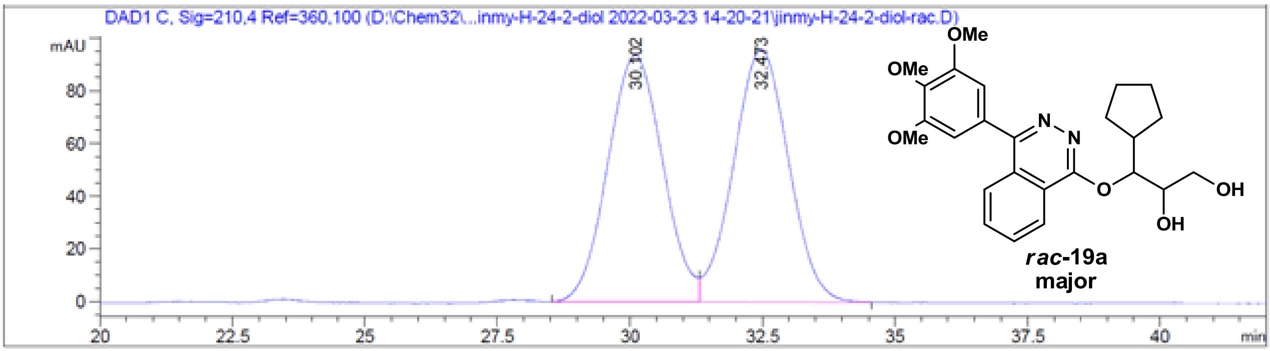

Signal 1: DAD1 B, Sig=254,4 Ref=360,100

Peak #	RetTime [min]	Type	Width [min]	Area [mAU*s]	Height [mAU]	Area %
1	30.098	BB	0.8509	701.29181	10.37099	49.5202
2	32.475	BB	0.8599	714.88208	10.58582	50.4798

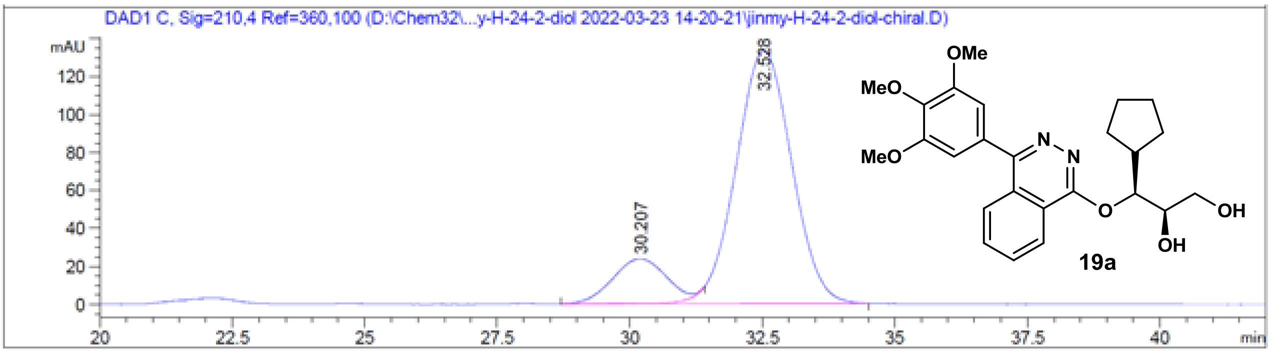

Signal 2: DAD1 C, Sig=210,4 Ref=360,100

Peak #	RetTime [min]	Type	Width [min]	Area [mAU*s]	Height [mAU]	Area %
1	30.207	BV E	0.8370	1644.14539	23.47830	14.4922
2	32.528	VB R	1.0388	9700.92676	133.33502	85.5078

Supplementary Figure 125. HPLC chromatogram for **19a**.

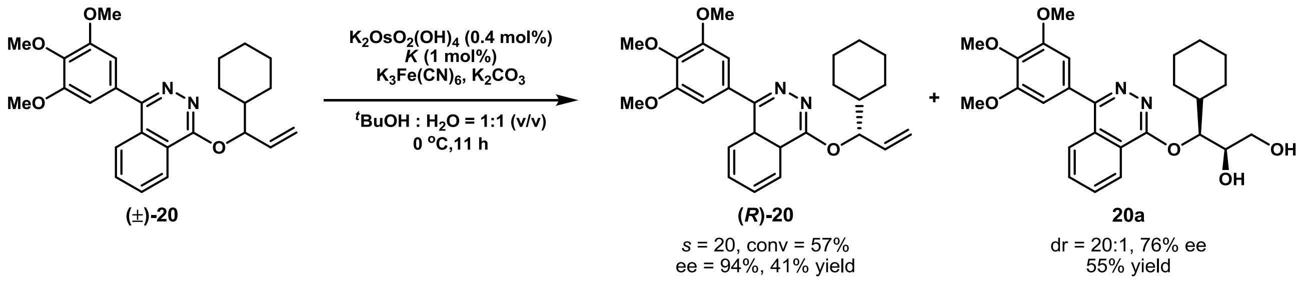

The general procedure **IV** was followed. The conversion of **(±)-20** was determined by crude ^{1}H NMR. **Conversion** (%) = [(2.34 – 1.00) / 2.34] % = 57%.

S = In [(1 – conv)(1 – ee)] / In [(1 – conv)(1 + ee)] = 20.

The recovered alkene **(*R*)-20** (17.8 mg, 41% yield, 94% ee) was purified by chromatography on silica gel (eluted with petroleum ether : ethyl acetate = 3:1). $[\alpha]_D^{25}$ = –6.42 (*c* 0.83, $CHCl_3$).

The dihydroxylated product **20a** (dr = 20:1, 25.7 mg, 55% yield, 76% ee) was purified by chromatography on silica gel (eluted with petroleum ether : ethyl acetate = 1:2). $[\alpha]_D^{25}$ = – 13.48 (*c* 1.38, $CHCl_3$)

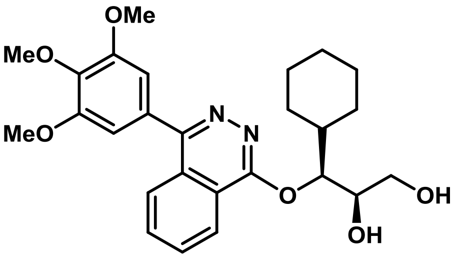

20a

(2*R*,3*S*)-3-cyclohexyl-3-(4-(3,4,5-trimethoxyphenyl)phthalazin-1-yloxy)propane-1,2-diol

^{1}H NMR (600 MHz, $CDCl_3$): δ 8.34 (d, *J* = 6.0 Hz, 1H), 8.06 (d, *J* = 12.0 Hz, 1H), 7.98 – 7.87 (m, 2H), 6.90 (s, 2H), 5.36 – 5.30 (m, 1H), 4.50 – 4.20 (br, 1H), 4.01 – 3.96 (m, 1H), 3.95 (s, 3H), 3.92 (s, 6H), 3.78 – 3.72 (m, 1H), 3.70 – 3.64 (m, 1H), 2.17 – 2.06 (m, 2H), 1.87 (d, *J* = 12.0 Hz, 1H), 1.71 (t, *J* = 12.0 Hz, 3H), 1.54 – 1.44 (m, 1H), 1.40 – 1.16 (m, 5H) ppm.

^{13}C NMR (151 MHz, $CDCl_3$): δ 161.3, 157.0, 153.4, 139.0, 132.9, 132.3, 131.3, 128.3, 126.5, 123.4, 120.4, 107.2, 82.1, 70.4, 62.5, 61.0, 56.3, 38.9, 30.7, 29.7, 26.6, 26.5, 26.3, 26.2 ppm.

HRMS (ESI) m/z: $[M + H]^+$ Calcd for $C_{26}H_{33}N_2O_6$ 469.2333; Found 469.2326.

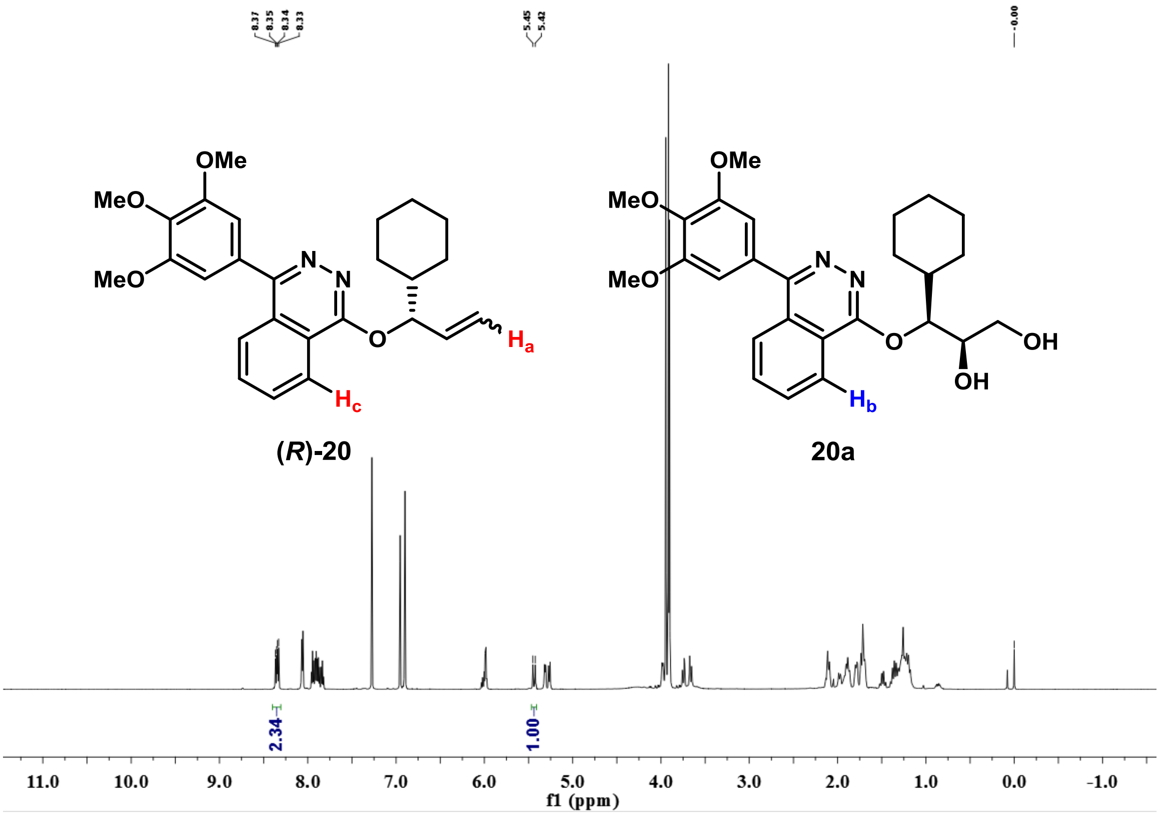

Supplementary Figure 126. ^{1}H NMR spectrum of crude mixture of compound **(*R*)-20** and **20a.**

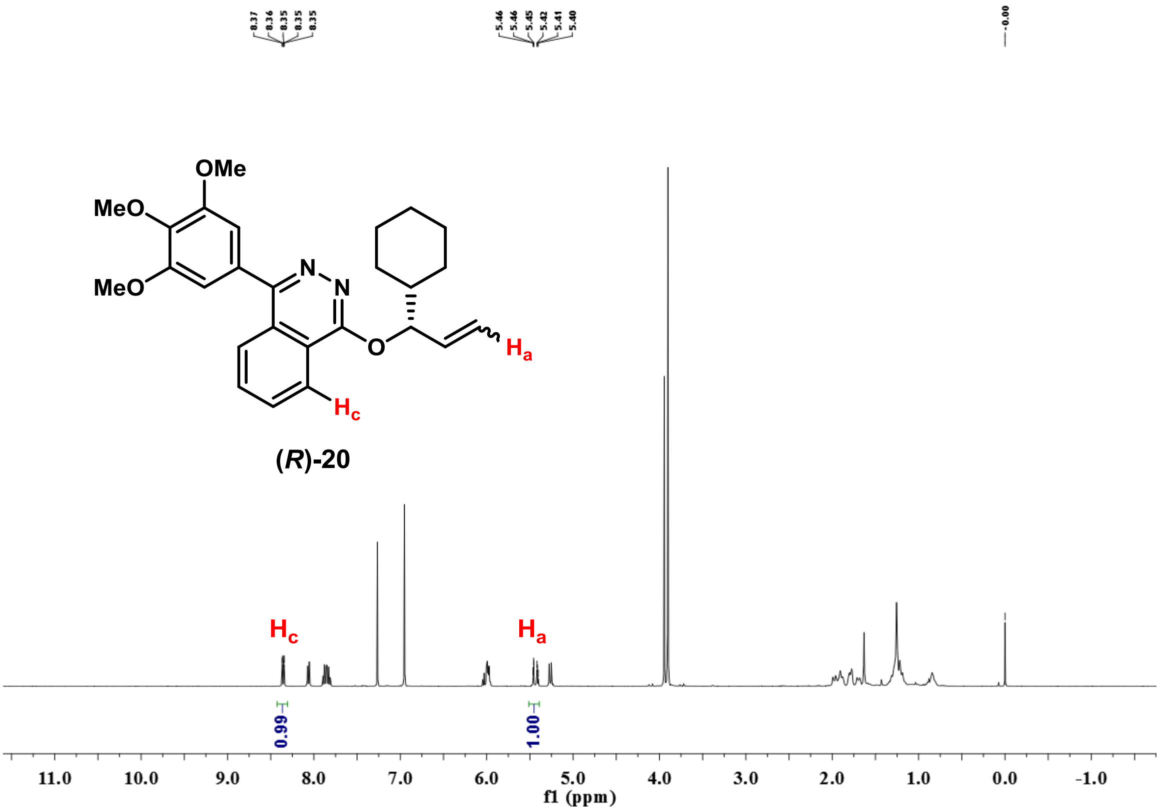

Supplementary Figure 127. ^{1}H NMR spectrum of recovered alkene **(*R*)-20**.

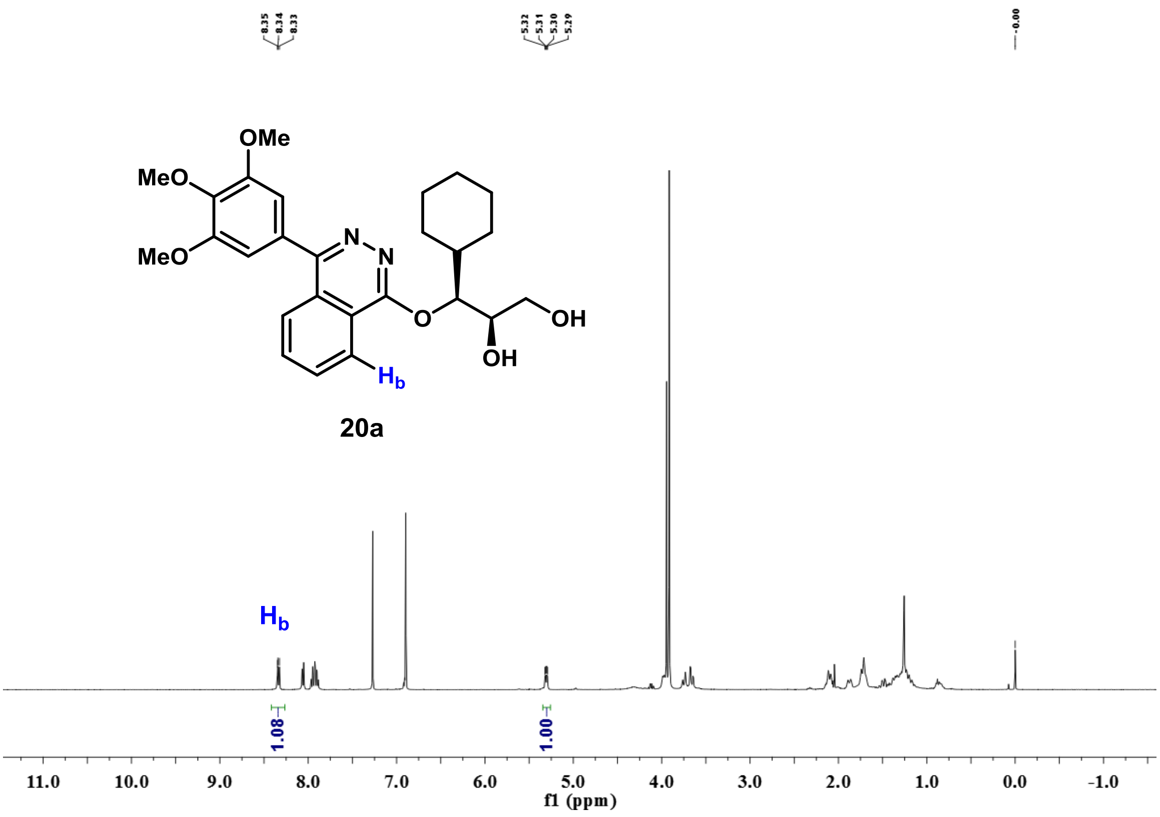

Supplementary Figure 128. ^{1}H NMR spectrum of dihydroxylated product **20a**.

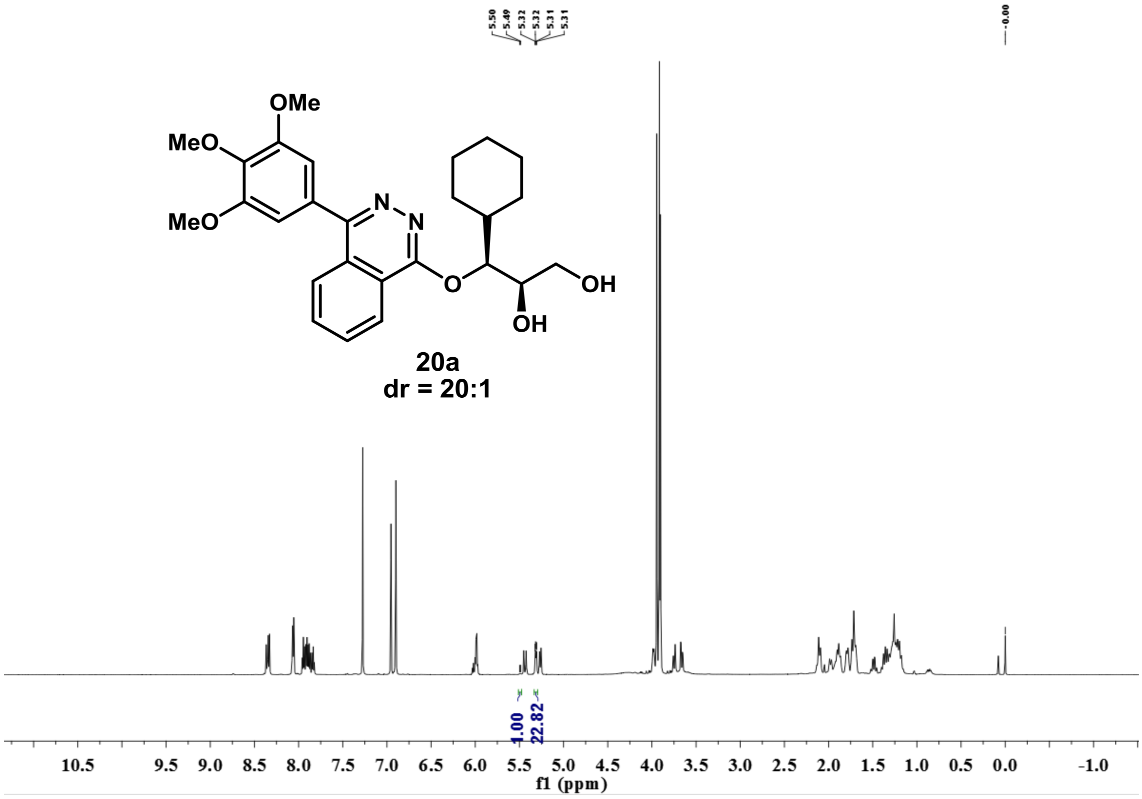

Supplementary Figure 129. ^{1}H NMR spectrum of crude mixture for diastereomeric ratio (dr).

HPLC (AD-H, 0.46*25 cm, 5μm, hexane/isopropanol = 80/20, flow = 1.0 mL/min, detection at 254 nm), retention time = 8.081 min (minor) and 11.537 min (major).

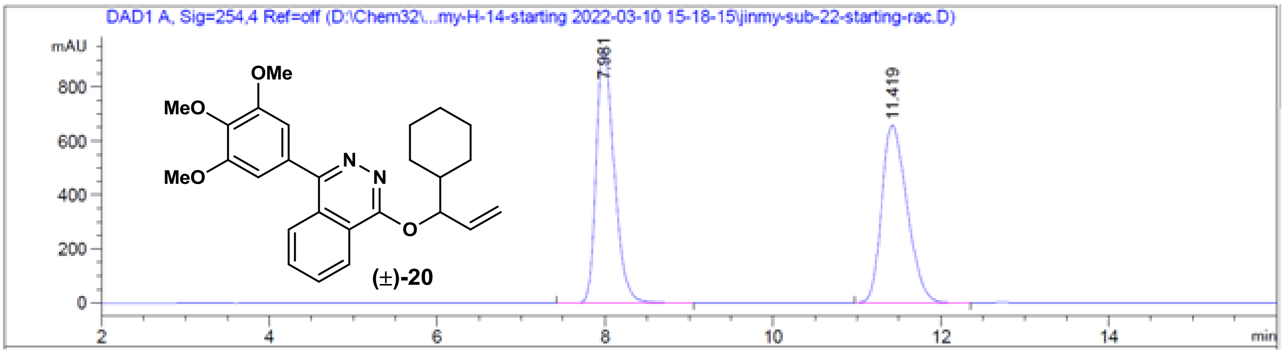

Signal 1: DAD1 A, Sig=254,4 Ref=off

Peak #	RetTime [min]	Type	Width [min]	Area [mAU*s]	Height [mAU]	Area %
1	7.981	BB	0.2290	1.39468e4	940.35510	50.0801
2	11.419	BB	0.3288	1.39022e4	657.08032	49.9199

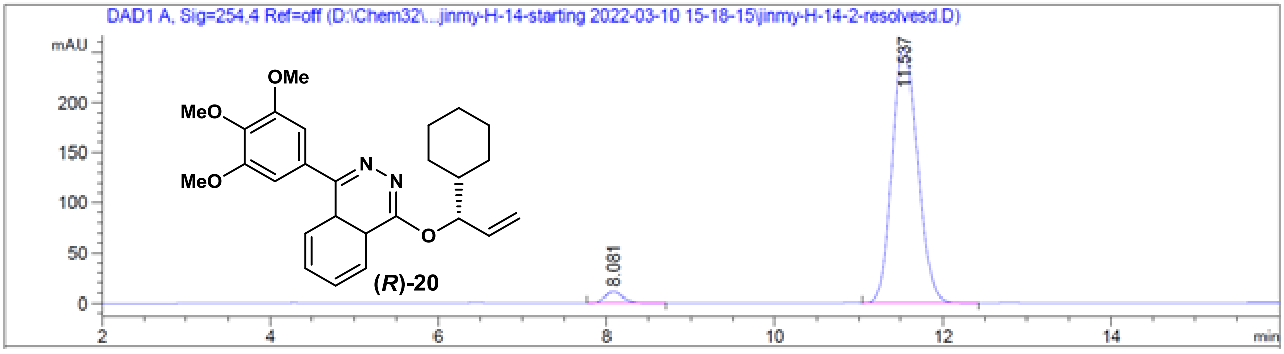

Signal 1: DAD1 A, Sig=254,4 Ref=off

Peak #	RetTime [min]	Type	Width [min]	Area [mAU*s]	Height [mAU]	Area %
1	8.081	BB	0.2246	159.67751	10.79533	2.9453
2	11.537	BB	0.3239	5261.83984	253.75502	97.0547

Supplementary Figure 130. HPLC chromatogram for **(*R*)-20**.

HPLC (AD-H, 0.46*25 cm, 5μm, hexane/isopropanol = 80/20, flow = 1.0 mL/min, detection at 210 nm), retention time = 27.643 min (major) and 33.716 min (minor).

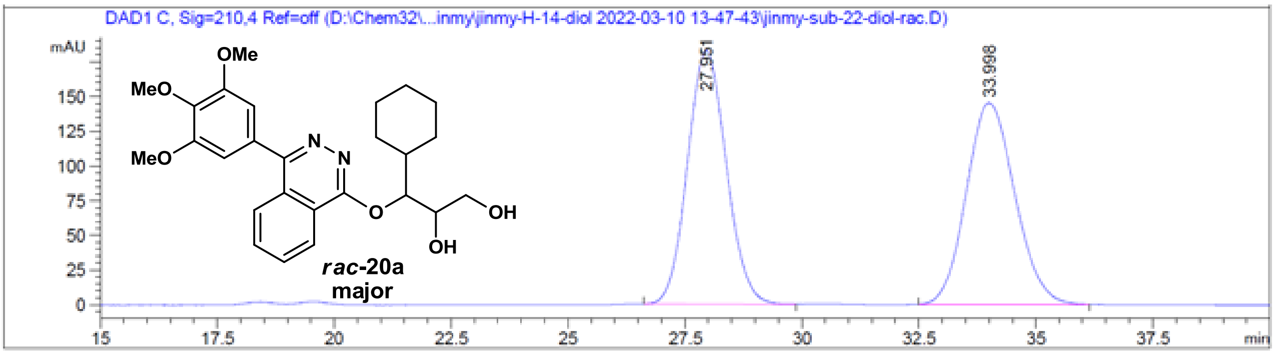

Signal 2: DAD1 C, Sig=210,4 Ref=off

Peak #	RetTime [min]	Type	Width [min]	Area [mAU*s]	Height [mAU]	Area %
1	27.951	BB	0.8733	1.05076e4	182.61343	50.1254
2	33.998	BB	1.0631	1.04551e4	145.01570	49.8746

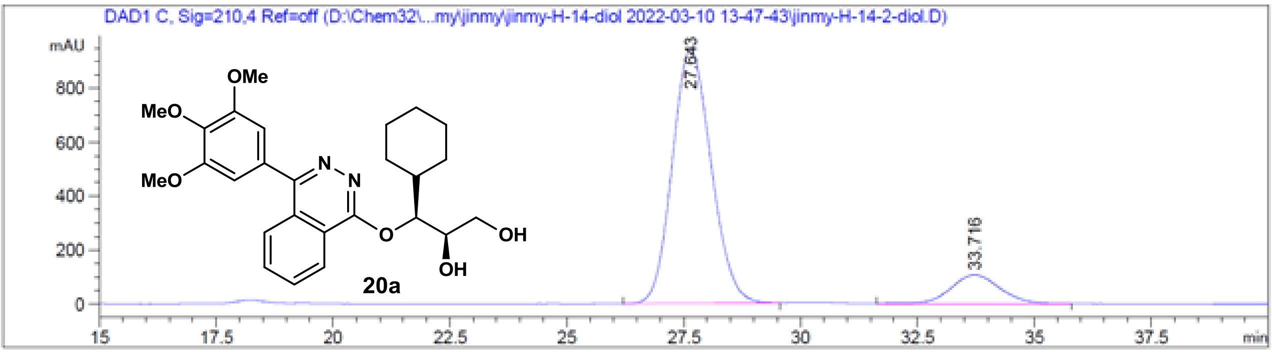

Signal 2: DAD1 C, Sig=210,4 Ref=off

Peak #	RetTime [min]	Type	Width [min]	Area [mAU*s]	Height [mAU]	Area %
1	27.643	BB	0.9001	5.45681e4	944.43274	87.4764
2	33.716	BB	1.0171	7812.28125	107.34276	12.5236

Supplementary Figure 131. HPLC chromatogram for **20a**.

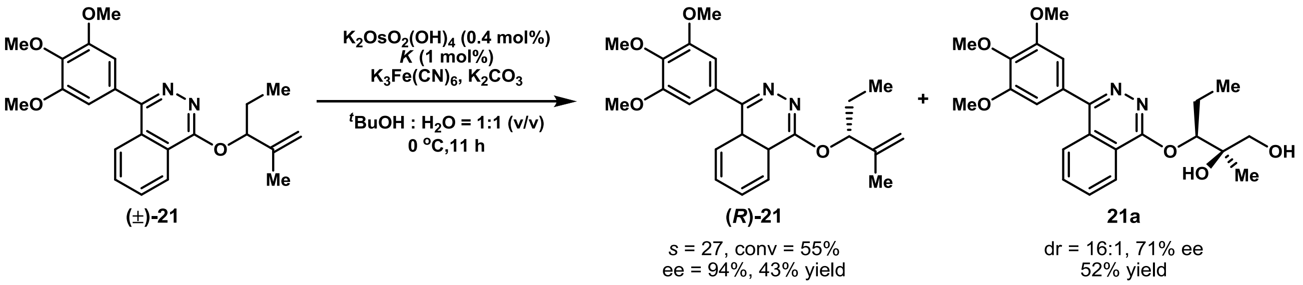

The general procedure **IV** was followed. The conversion of **(±)-21** was determined by crude ^{1}H NMR. **Conversion** (%) = [(2.20 – 1.00) / 2.20] % = 55%.

S = In [(1 – conv)(1 – ee)] / In [(1 – conv)(1 + ee)] = 27.

The recovered alkene **(*R*)-21** (16.9 mg, 43% yield, 94% ee) was purified by chromatography on silica gel (eluted with petroleum ether : ethyl acetate = 3:1). $[\alpha]_D^{25}$ = –11.63 (*c* 0.48, $CHCl_3$).

The dihydroxylated product **21a** (dr = 16:1, 22.3 mg, 52% yield, 71% ee) was purified by chromatography on silica gel (eluted with petroleum ether : ethyl acetate = 1:2). $[\alpha]_D^{25}$ = – 35.33 (*c* 1.05, $CHCl_3$)

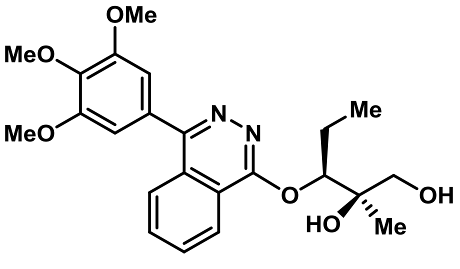

21a

(2*R*,3*S*)-2-methyl-3-(4-(3,4,5-trimethoxyphenyl)phthalazin-1-yloxy)pentane-1,2-diol

^{1}H NMR (600 MHz, $CDCl_3$): δ 8.36 (d, *J* = 6.0 Hz, 1H), 8.06 (d, *J* = 12.0 Hz, 1H), 7.96 – 7.87 (m, 2H), 6.90 (s, 2H), 5.36 (d, *J* = 12.0 Hz, 2H), 3.95 (s, 3H), 3.92 (s, 6H), 3.59 (d, *J* = 18.0 Hz, 1H), 3.35 – 3.27 (m, 1H), 3.18 (m, 1H), 2.20 – 2.10 (m, 1H), 1.98 – 1.88 (m, 1H), 1.24 (s, 3H), 0.98 (t, *J* = 6.0 Hz, 3H) ppm.

^{13}C NMR (151 MHz, $CDCl_3$): δ 161.6, 127.1, 153.4, 139.0, 132.9, 132.2, 131.3, 128.3,126.5, 123.4, 120.4, 107.2, 81.5, 73.6, 66.9, 61.0, 56.3, 22.4, 17.4, 11.3 ppm.

HRMS (ESI) m/z: $[M + H]^+$ Calcd for $C_{23}H_{29}N_2O_6$ 429.2020; Found 429.2015.

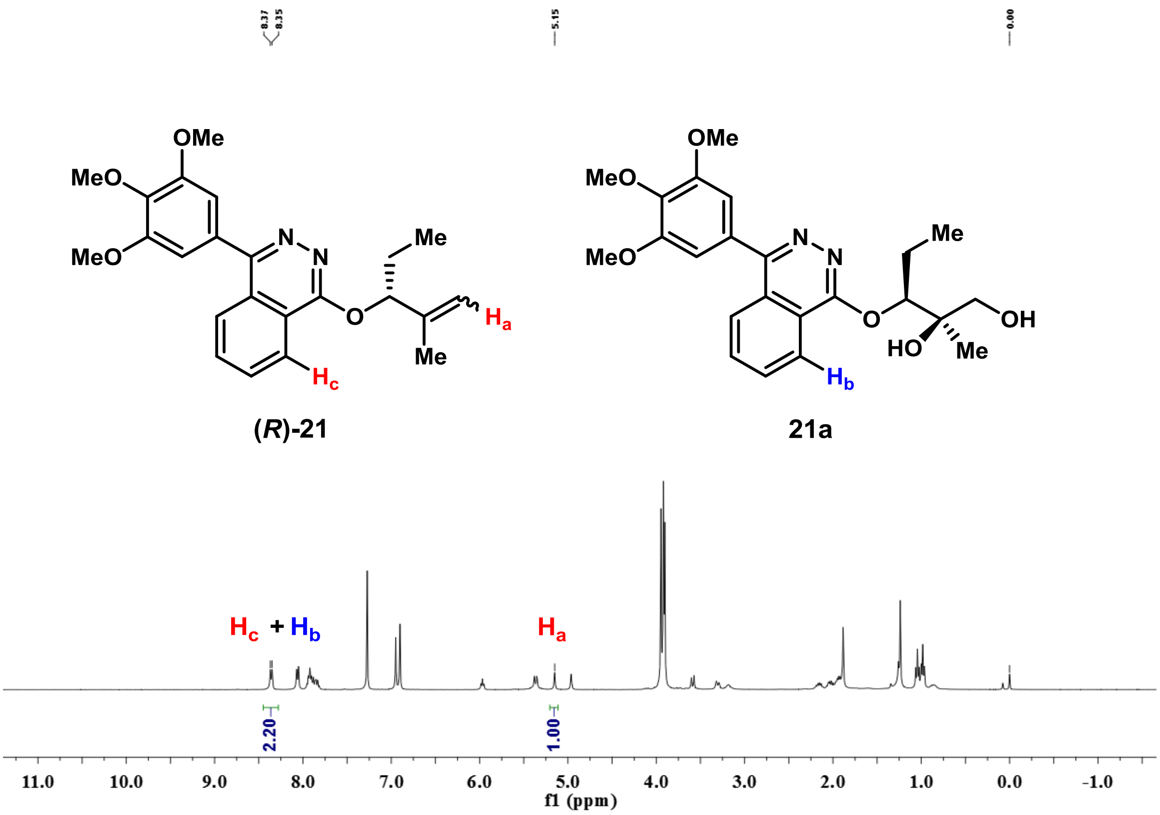

Supplementary Figure 132. ^{1}H NMR spectrum of crude mixture of compound **(*R*)-21** and **21a.**

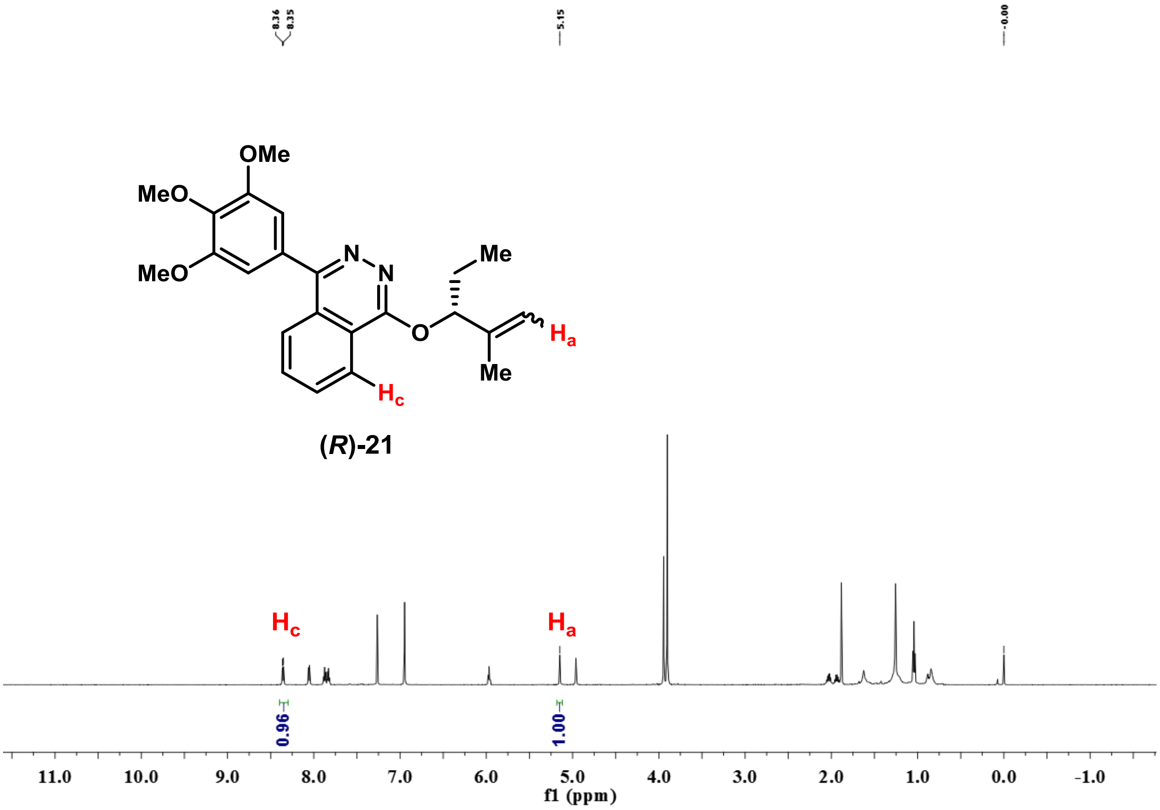

Supplementary Figure 133. ^{1}H NMR spectrum of recovered alkene **(*R*)-21**.

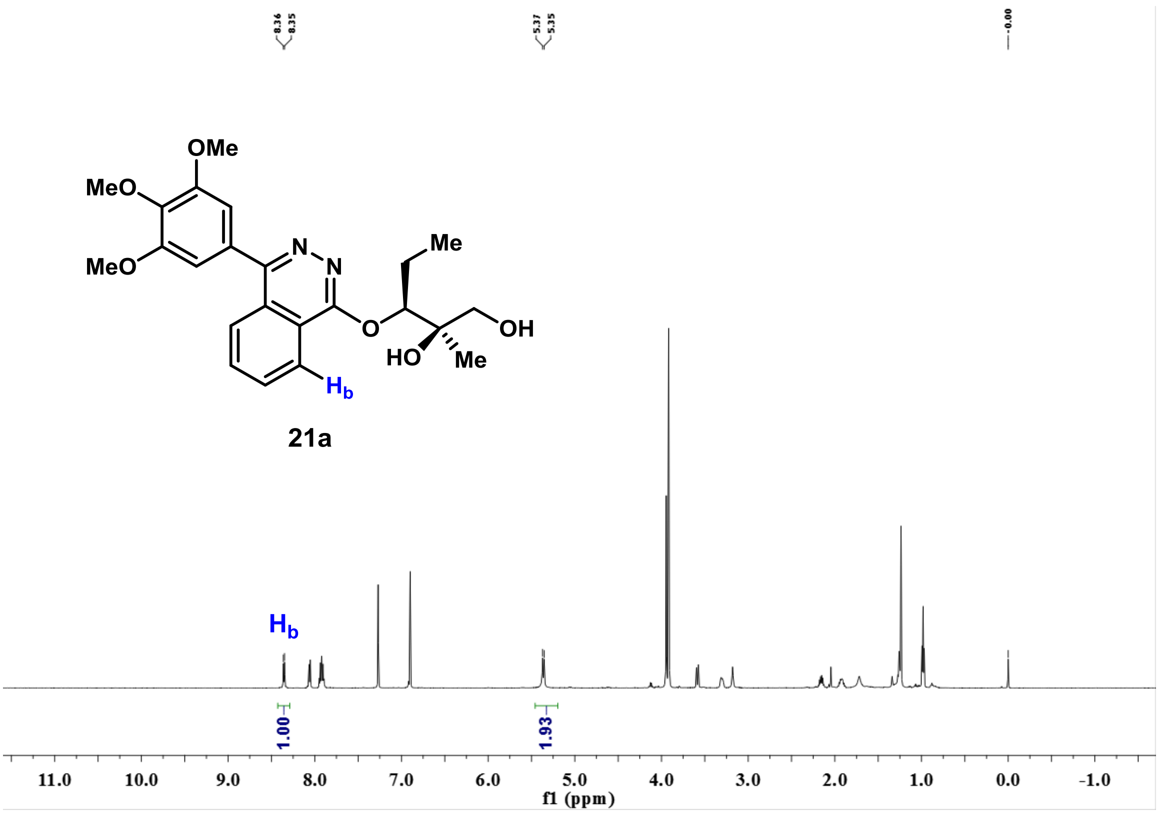

Supplementary Figure 134. ^{1}H NMR spectrum of dihydroxylated product **21a**.

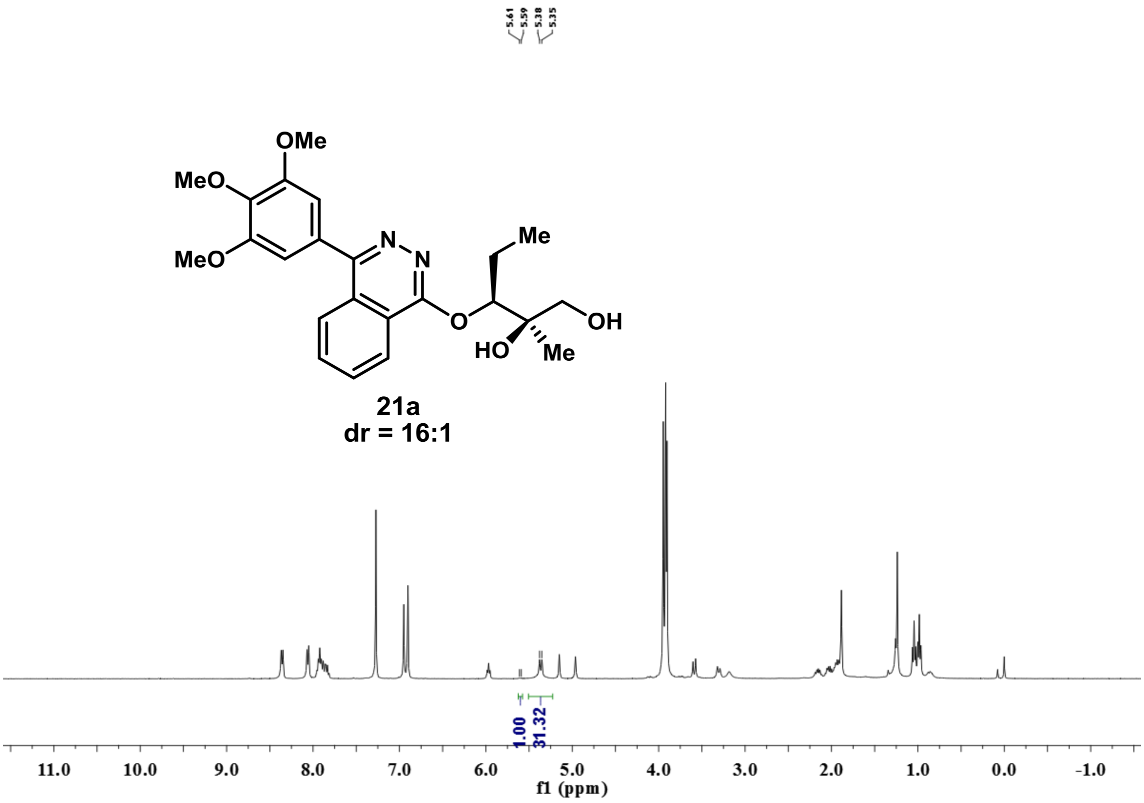

Supplementary Figure 135. ^{1}H NMR spectrum of crude mixture for diastereomeric ratio (dr).

HPLC (OD-H, 0.46*25 cm, 5μm, hexane/isopropanol = 90/10, flow = 1.0 mL/min, detection at 254 nm) retention time = 10.884 min (major) and 14.411 min (minor).

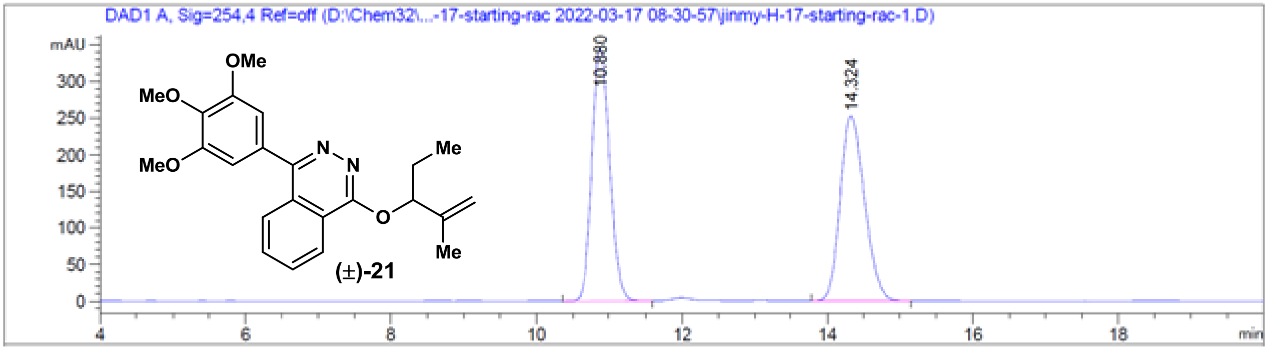

Signal 1: DAD1 A, Sig=254,4 Ref=off

Peak #	RetTime [min]	Type	Width [min]	Area [mAU*s]	Height [mAU]	Area %
1	10.880	BB	0.2633	5858.51270	346.47034	50.0574
2	14.324	BB	0.3598	5845.06982	252.73717	49.9426

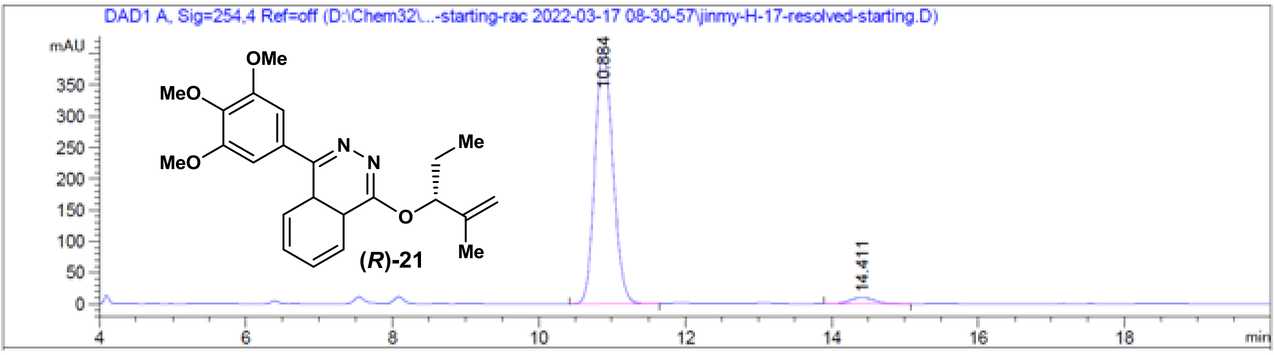

Signal 1: DAD1 A, Sig=254,4 Ref=off

Peak #	RetTime [min]	Type	Width [min]	Area [mAU*s]	Height [mAU]	Area %
1	10.884	BB	0.2643	6940.27002	408.38849	96.8150
2	14.411	BB	0.3472	228.31908	10.04286	3.1850

Supplementary Figure 136. HPLC chromatogram for ***(R)*-21**.

HPLC (OD-H, 0.46*25 cm, 5μm, hexane/isopropanol = 90/10, flow = 1.0 mL/min, detection at 254 nm) retention time = 19.563 min (minor) and 21.494 min (major).

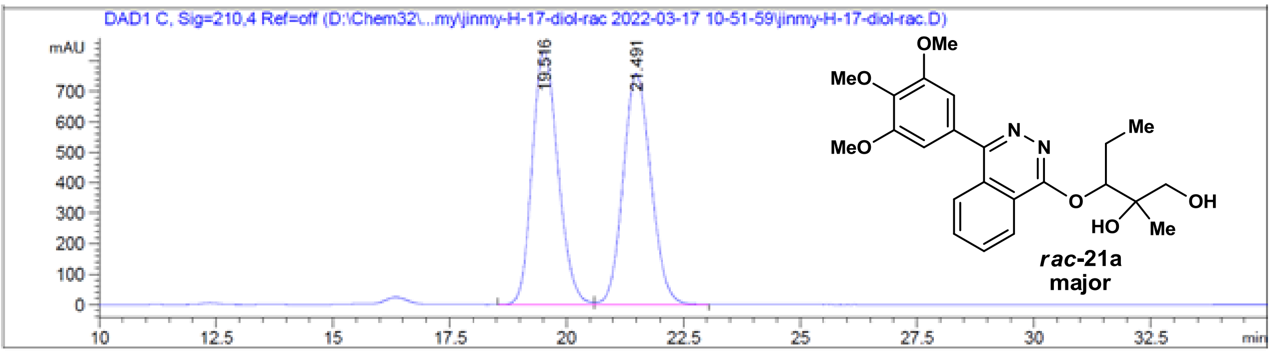

Signal 2: DAD1 C, Sig=210,4 Ref=off

Peak #	RetTime [min]	Type	Width [min]	Area [mAU*s]	Height [mAU]	Area %
1	19.516	BV	0.5972	3.18985e4	832.18353	50.5335
2	21.491	VB	0.6434	3.12250e4	756.66364	49.4665

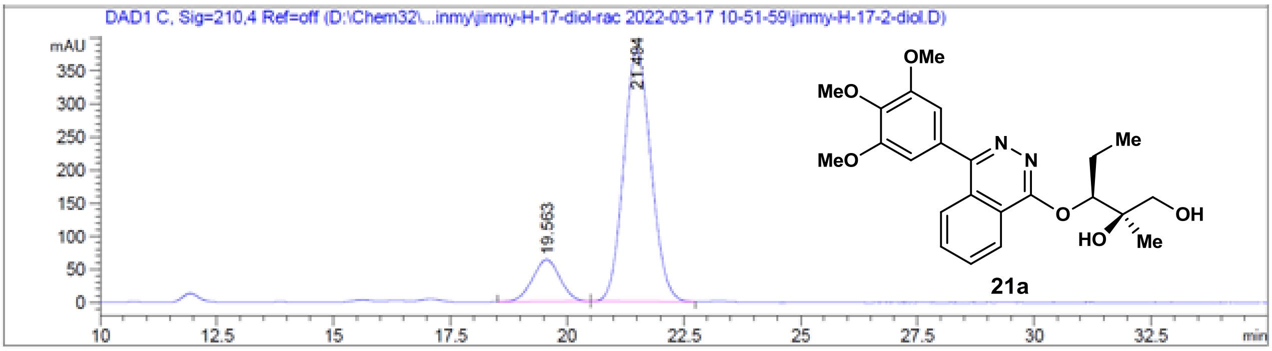

Signal 2: DAD1 C, Sig=210,4 Ref=off

Peak #	RetTime [min]	Type	Width [min]	Area [mAU*s]	Height [mAU]	Area %
1	19.563	BB	0.6269	2620.97217	63.58509	14.4259
2	21.494	BB	0.6414	1.55475e4	381.58606	85.5741

Supplementary Figure 137. HPLC chromatogram for **21a**.

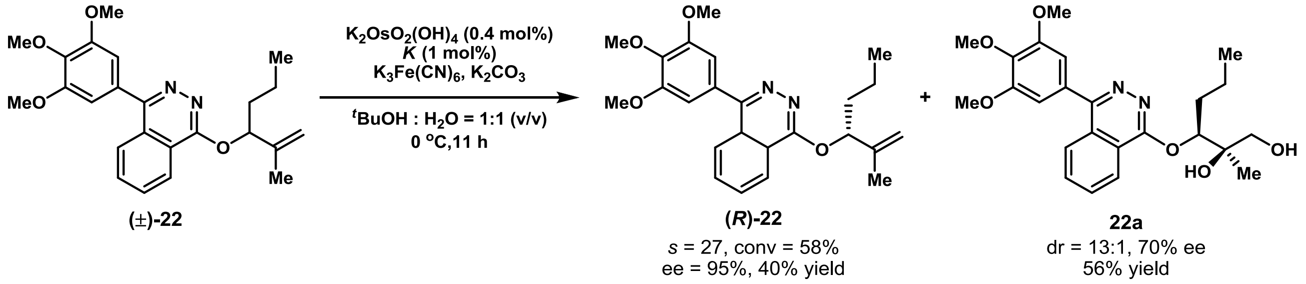

The general procedure **IV** was followed. The conversion of **(±)-22** was determined by crude ^{1}H NMR. **Conversion** (%) = [(2.37 – 1.00) / 2.37] % = 58%.

S = In [(1 – conv)(1 – ee)] / In [(1 – conv)(1 + ee)] = 27.

The recovered alkene **(*R*)-22** (16.3 mg, 40% yield, 95% ee) was purified by chromatography on silica gel (eluted with petroleum ether : ethyl acetate = 3:1). $[\alpha]_D^{25}$ = –10.62 (*c* 0.58, $CHCl_3$).

The dihydroxylated product **22a** (dr = 13:1, 24.7 mg, 56% yield, 70% ee) was purified by chromatography on silica gel (eluted with petroleum ether : ethyl acetate = 1:2). $[\alpha]_D^{25}$ = – 51.73 (*c* 0.75, $CHCl_3$)

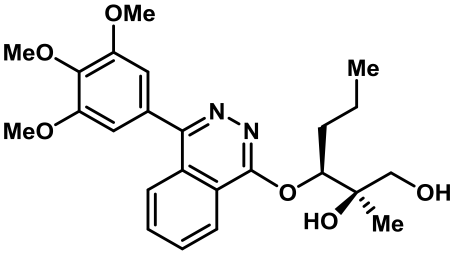

22a

(2*R*,3*S*)-2-methyl-3-(4-(3,4,5-trimethoxyphenyl)phthalazin-1-yloxy)hexane-1,2-diol

^{1}H NMR (600 MHz, $CDCl_3$): δ 8.35 (d, *J* = 6.0 Hz, 1H), 8.07 (d, *J* = 6.0 Hz, 1H), 7.98 – 7.88 (m, 2H), 6.91 (s, 2H), 5.48 (d, *J* = 12.0 Hz, 1H), 5.39 (br, 1H), 3.95 (s, 3H), 3.92 (s, 6H), 3.58 (d, *J* = 12.0 Hz, 1H), 3.31 (d, *J* = 12.0 Hz, 1H), 3.24 (br, 1H), 2.11 – 2.02 (m, 1H), 1.98 – 1.87 (m, 1H), 1.52 – 1.42 (m, 1H), 1.39 – 1.30 (m, 1H), 1.24 (s, 3H), 0.93 (t, *J* = 6.0 Hz, 3H) ppm.

^{13}C NMR (151 MHz, $CDCl_3$): δ 161.4, 157.0, 153.4, 139.0, 132.9, 132.2, 131.3, 128.2, 126.5, 123.4, 120.4, 107.2, 79.4, 73.5, 66.9, 61.0, 56.3, 31.2, 20.0, 17.4, 14.0 ppm.

HRMS (ESI) m/z: $[M + H]^+$ Calcd for $C_{24}H_{31}N_2O_2$ 443.2177; Found 443.2171.

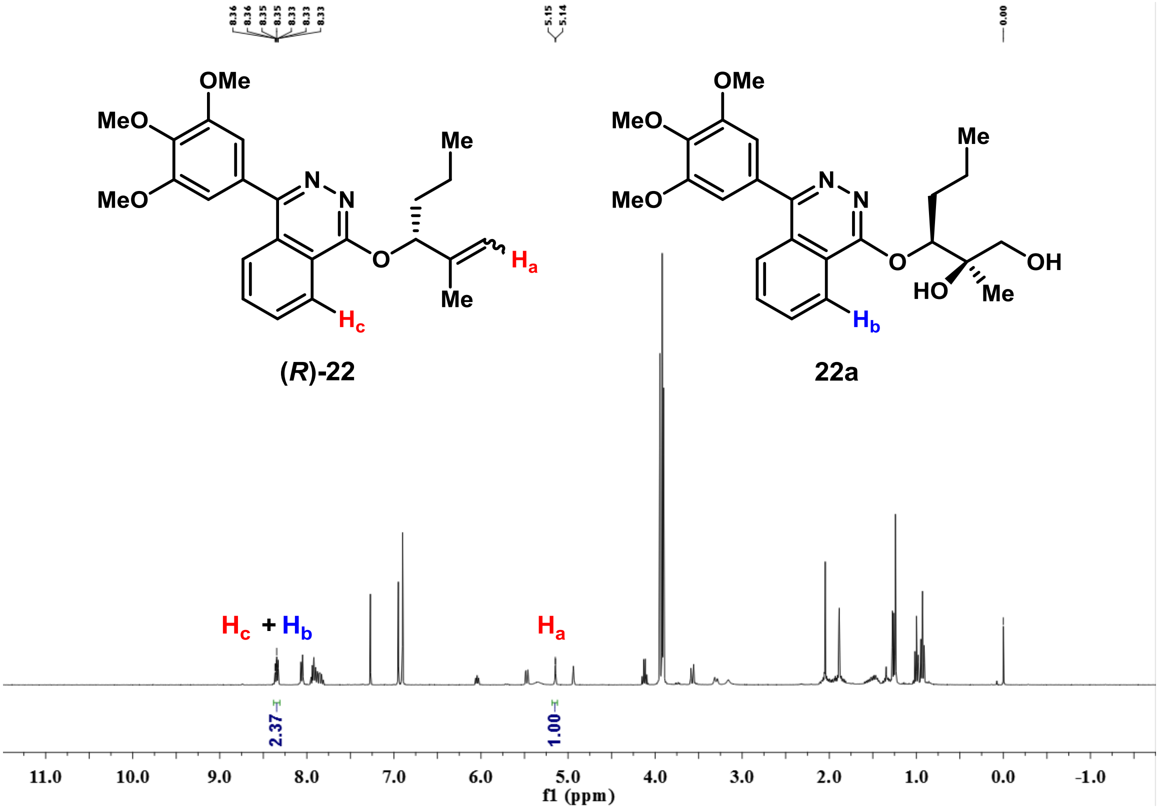

Supplementary Figure 138. ^{1}H NMR spectrum of crude mixture of compound **(*R*)-22** and **22a.**

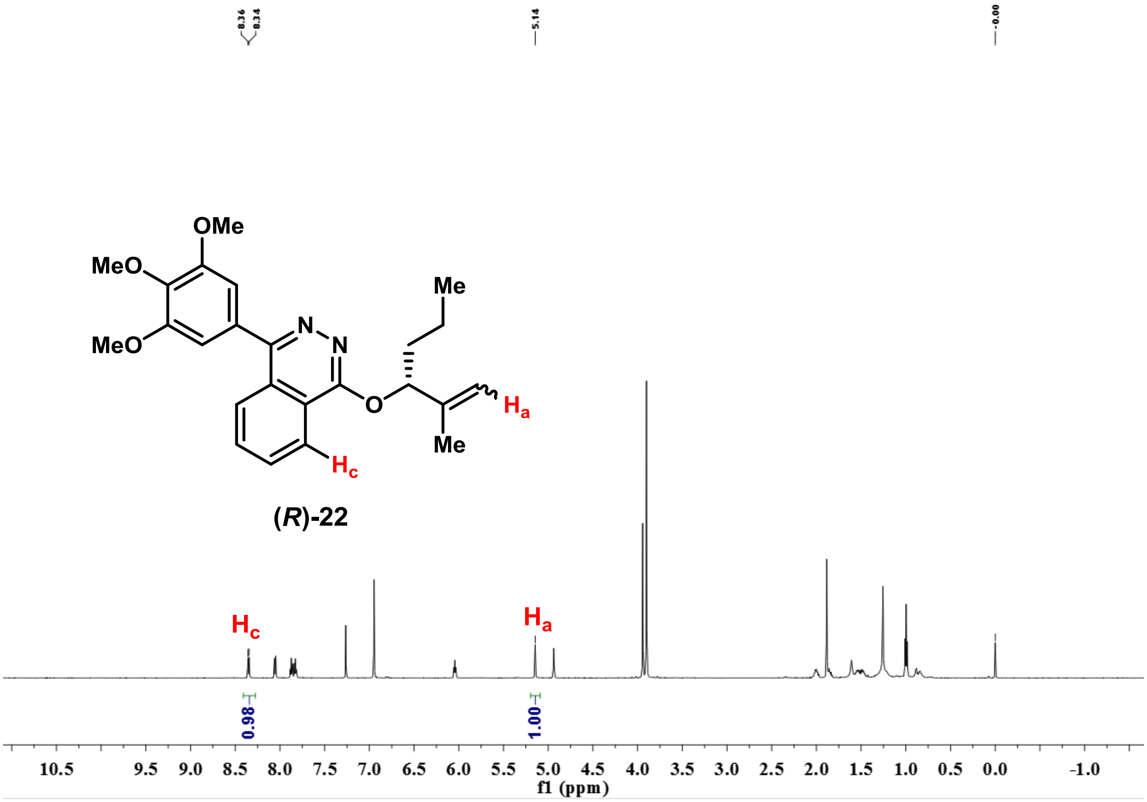

Supplementary Figure 139. ^{1}H NMR spectrum of recovered alkene **(*R*)-22**.

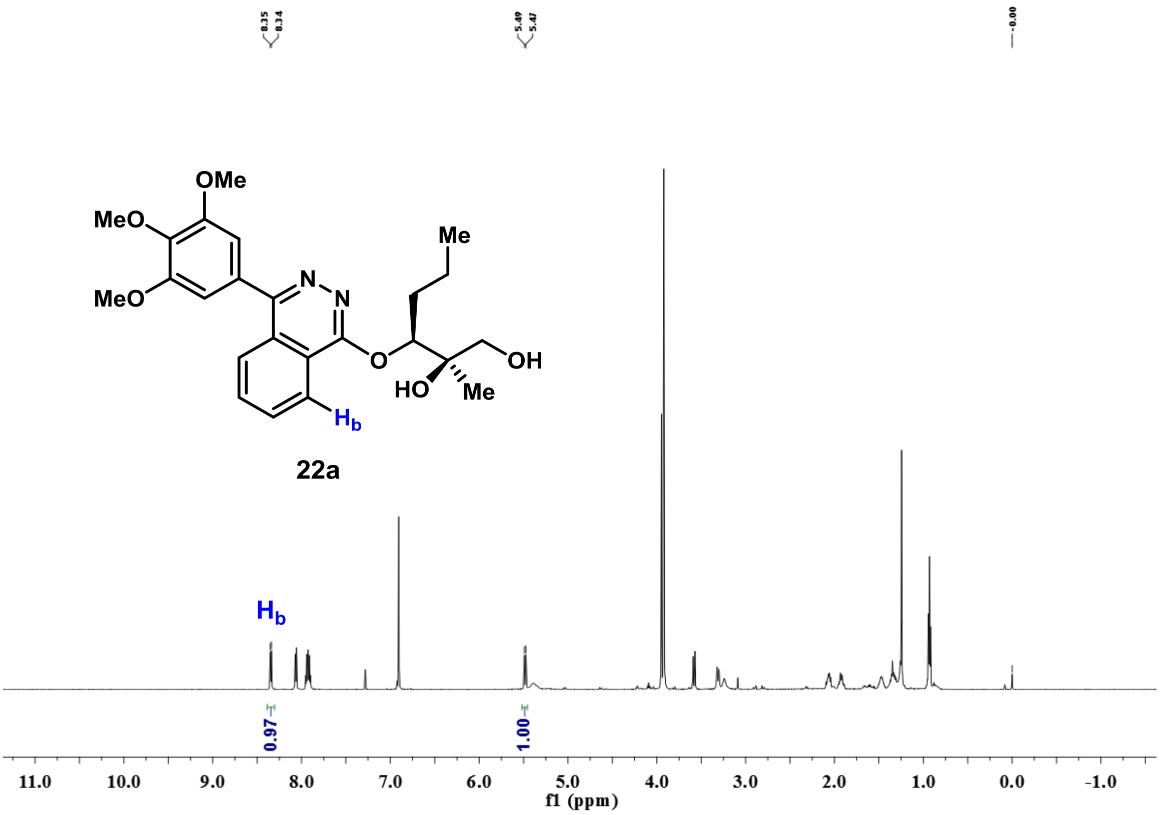

Supplementary Figure 140. ^{1}H NMR spectrum of dihydroxylated product **22a**.

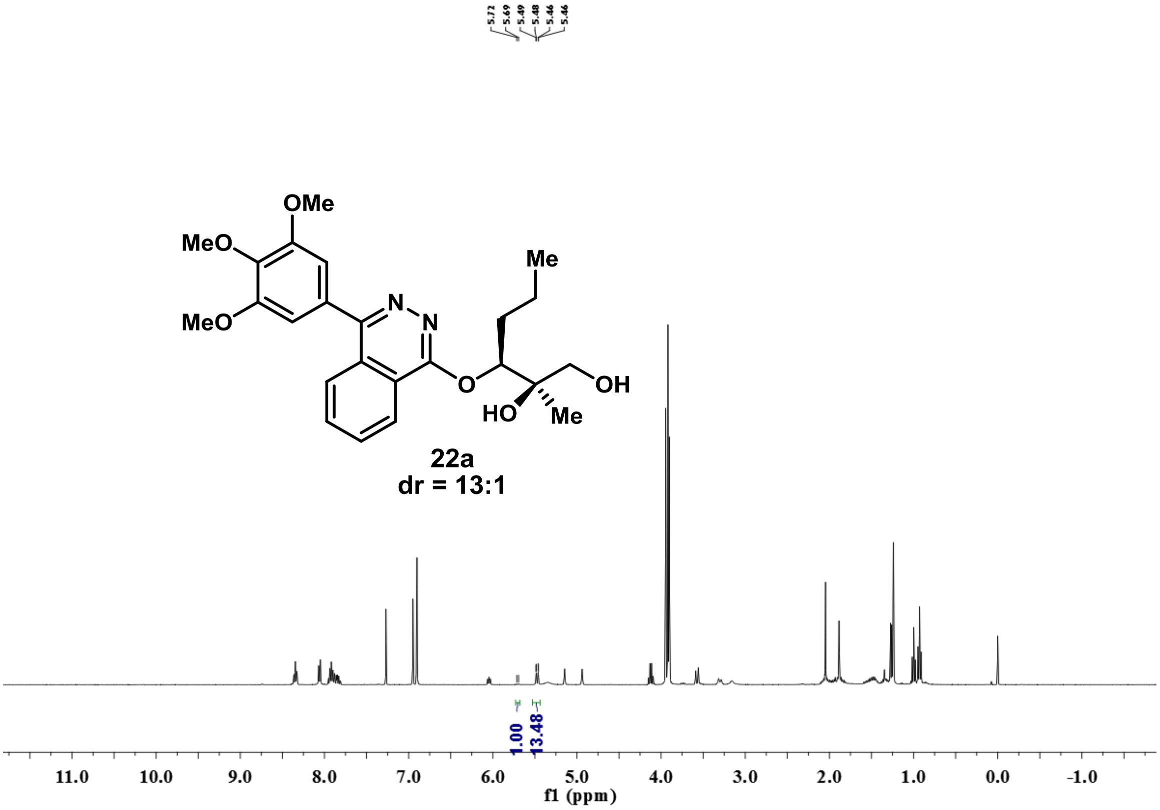

Supplementary Figure 141. ^{1}H NMR spectrum of crude mixture for diastereomeric ratio (dr).

HPLC (OD-H, 0.46*25 cm, 5μm, hexane/isopropanol = 90/10, flow = 1.0 mL/min, detection at 254 nm), retention time = 8.525 min (major) and 9.925 min (minor).

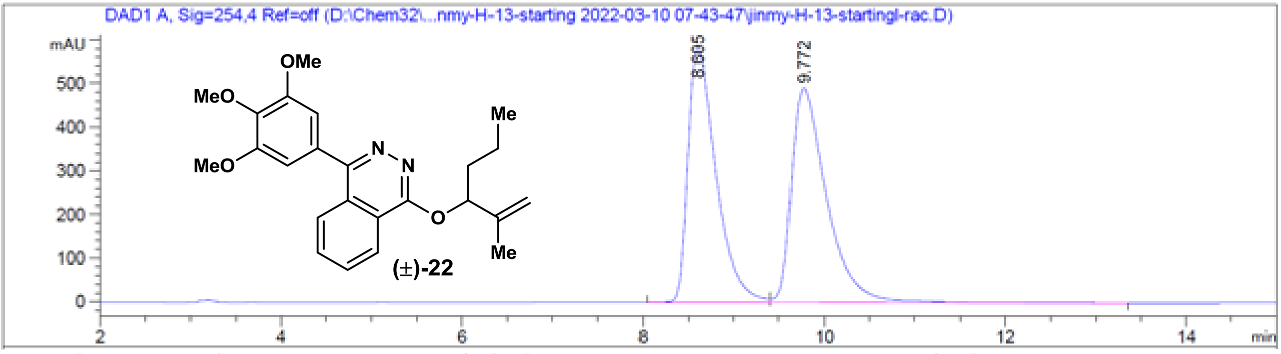

Peak #	RetTime [min]	Type	Width [min]	Area [mAU*s]	Height [mAU]	Area %
1	8.605	BV	0.3267	1.26408e4	583.61121	48.9164
2	9.772	VB	0.4043	1.32008e4	489.99841	51.0836

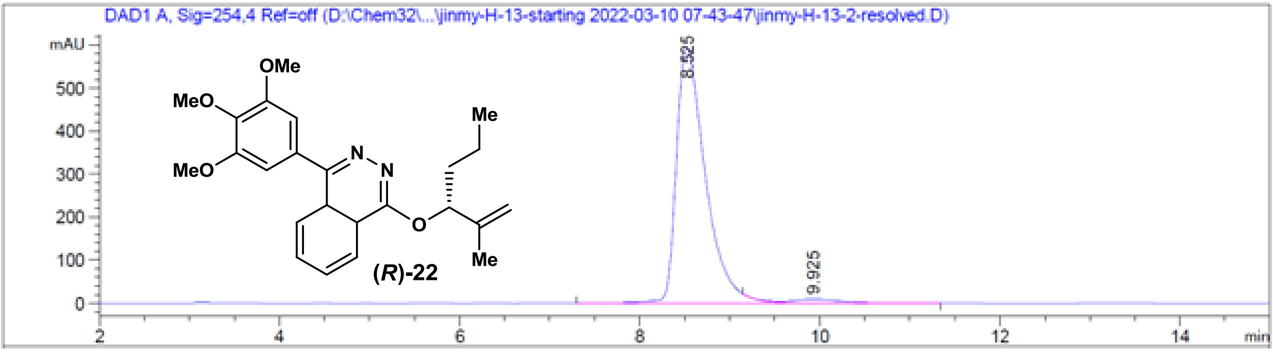

Signal 1: DAD1 A, Sig=254,4 Ref=off

Peak #	RetTime [min]	Type	Width [min]	Area [mAU*s]	Height [mAU]	Area %
1	8.525	BV R	0.3361	1.32446e4	594.02173	97.3219
2	9.925	VB E	0.5572	364.46234	9.34120	2.6781

Supplementary Figure 142. HPLC chromatogram for **(*R*)-22**.

HPLC (OD-H, 0.46*25 cm, 5μm, hexane/isopropanol = 90/10, flow = 1.0 mL/min, detection at 254 nm), retention time = 49.832min (major) and 72.481 min (minor).

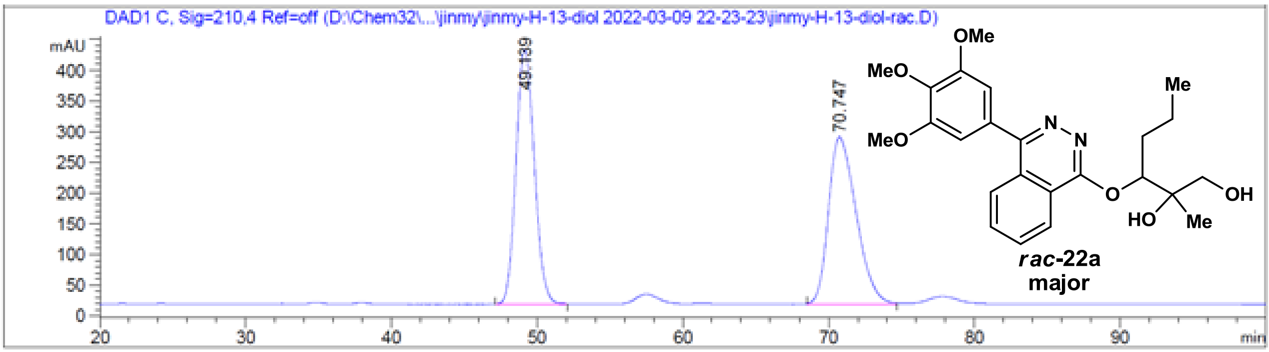

Signal 2: DAD1 C, Sig=210,4 Ref=off

Peak #	RetTime [min]	Type	Width [min]	Area [mAU*s]	Height [mAU]	Area %
1	49.139	BB	1.3350	3.59627e4	414.16129	50.1377
2	70.747	MM R	2.1957	3.57651e4	271.47400	49.8623

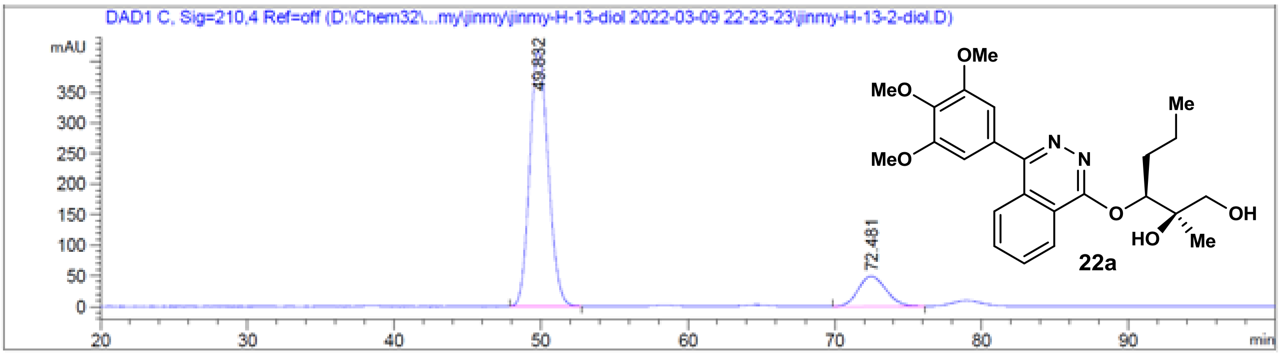

Signal 2: DAD1 C, Sig=210,4 Ref=off

Peak #	RetTime [min]	Type	Width [min]	Area [mAU*s]	Height [mAU]	Area %
1	49.832	BB	1.3200	3.66434e4	418.29129	84.9311
2	72.481	MM R	2.2046	6501.47119	49.14983	15.0689

Supplementary Figure 143. HPLC chromatogram for **22a**.

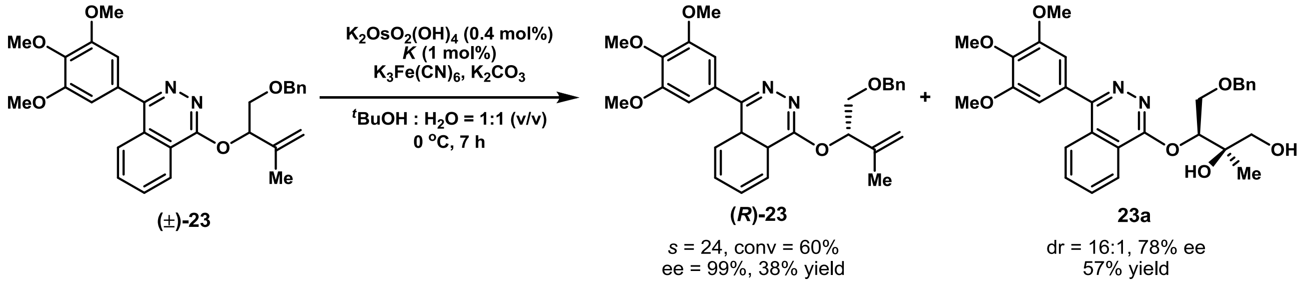

The general procedure **IV** was followed. The conversion of **(±)-23** was determined by crude ^{1}H NMR. **Conversion** (%) = [(2.51 – 1.00) / 2.51] % = 60%.

S = In [(1 – conv)(1 – ee)] / In [(1 – conv)(1 + ee)] = 24.

The recovered alkene **(*R*)-23** (18.4 mg, 38% yield, 99% ee) was purified by chromatography on silica gel (eluted with petroleum ether : ethyl acetate = 3:1). $[\alpha]_D^{25}$ = –8.92 (*c* 1.11, $CHCl_3$).

The dihydroxylated product **23a** (dr = 16:1, 29.6 mg, 57% yield, 78% ee) was purified by chromatography on silica gel (eluted with petroleum ether : ethyl acetate = 1:2). $[\alpha]_D^{25}$ = – 12.32 (*c* 1.38, $CHCl_3$)

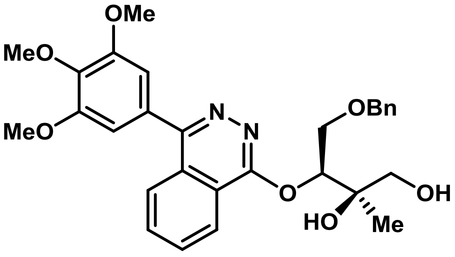

23a

(2*R*,3*S*)-4-(benzyloxy)-2-methyl-3-(4-(3,4,5-trimethoxyphenyl)phthalazin-1-yloxy)butane-1,2-diol

^{1}H NMR (600 MHz, $CDCl_3$): δ 8.33 (d, *J* = 12.0 Hz, 1H), 8.06 (d, *J* = 6.0 Hz, 1H), 7.95 – 7.88 (m, 2H), 7.26 – 7.20 (m, 5H), 5.71 (d, *J* = 6.0 Hz, 1H), 5.20 – 5.10 (m, 1H), 4.60 (d, *J* = 12.0 Hz, 1H), 4.53 (d, *J* = 12.0 Hz, 1H), 4.18 (d, *J* = 12.0 Hz, 1H), 4.03 (t, *J* = 12.0 Hz, 1H), 3.95 (s, 3H), 3.92 (s, 6H), 3.63 (d, *J* = 12.0 Hz, 1H), 3.44 (s, 1H), 3.33 (t, *J* = 6.0 Hz, 1H), 1.23 (s, 3H) ppm.

^{13}C NMR (151 MHz, $CDCl_3$): δ 161.1, 157.2, 153.4, 139.0, 138.0, 132.9, 132.1, 131.4, 128.5, 128.3, 128.6, 127.9, 127.6, 126.4, 123.6, 120.5, 107.2, 78.7, 73.2, 73.7, 69.6, 66.9, 61.0, 56.3, 18.2 ppm.

HRMS (ESI) m/z: $[M + H]^+$ Calcd for $C_{29}H_{33}N_2O_7$ 521.2282; Found 521.2276.

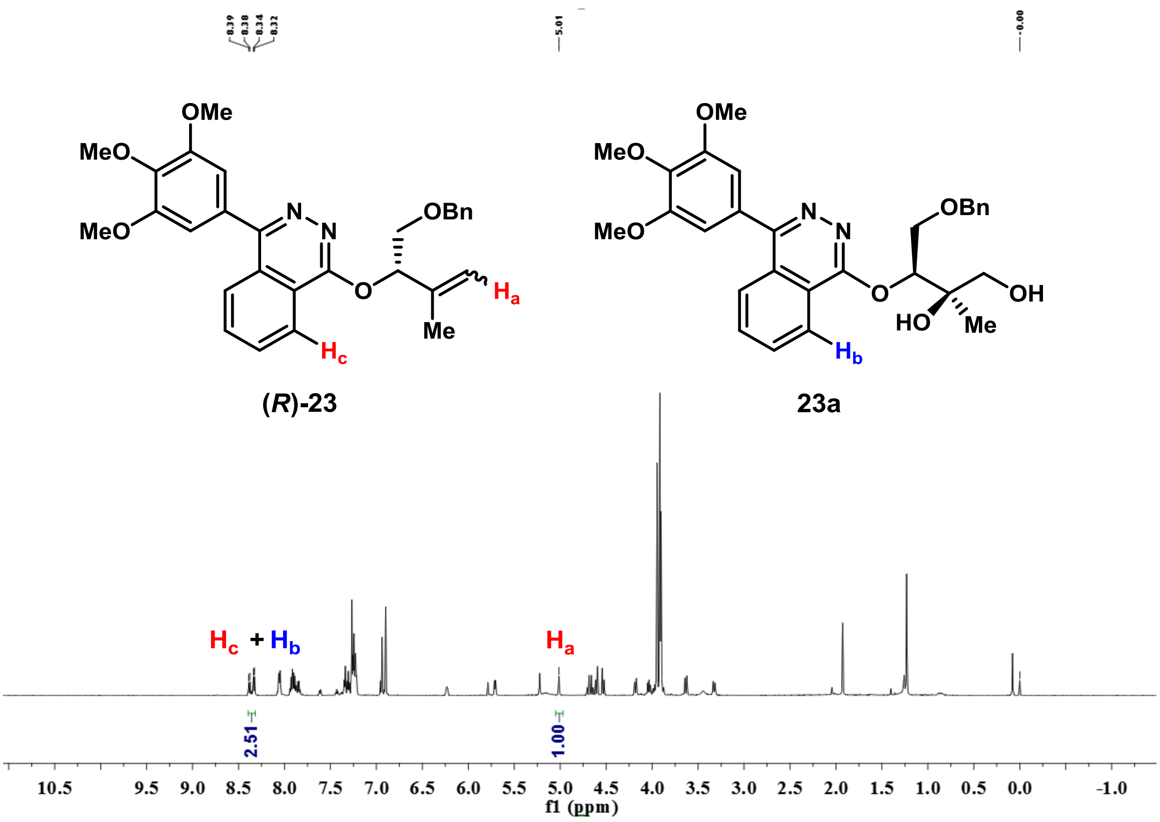

Supplementary Figure 144. ^{1}H NMR spectrum of crude mixture of compound ***(R)*-23** and **23a.**

:

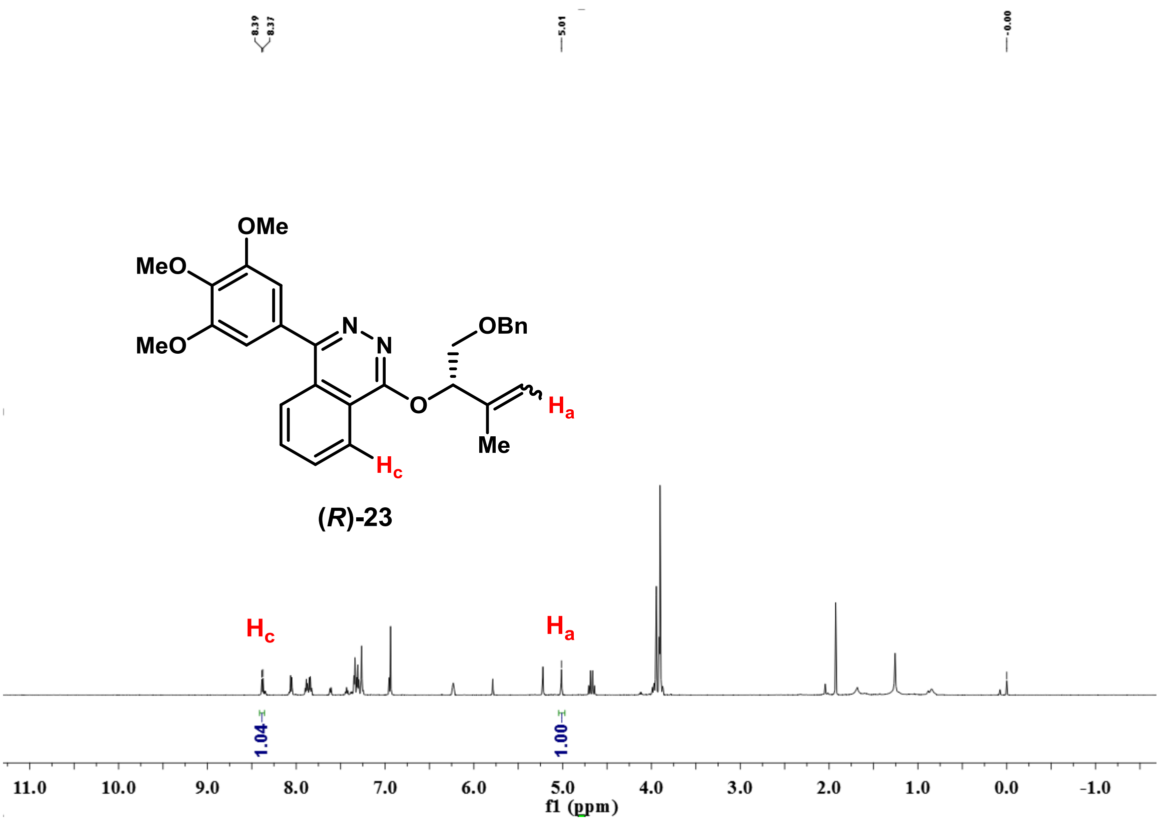

Supplementary Figure 145. ^{1}H NMR spectrum of recovered alkene ***(R)*-23**.

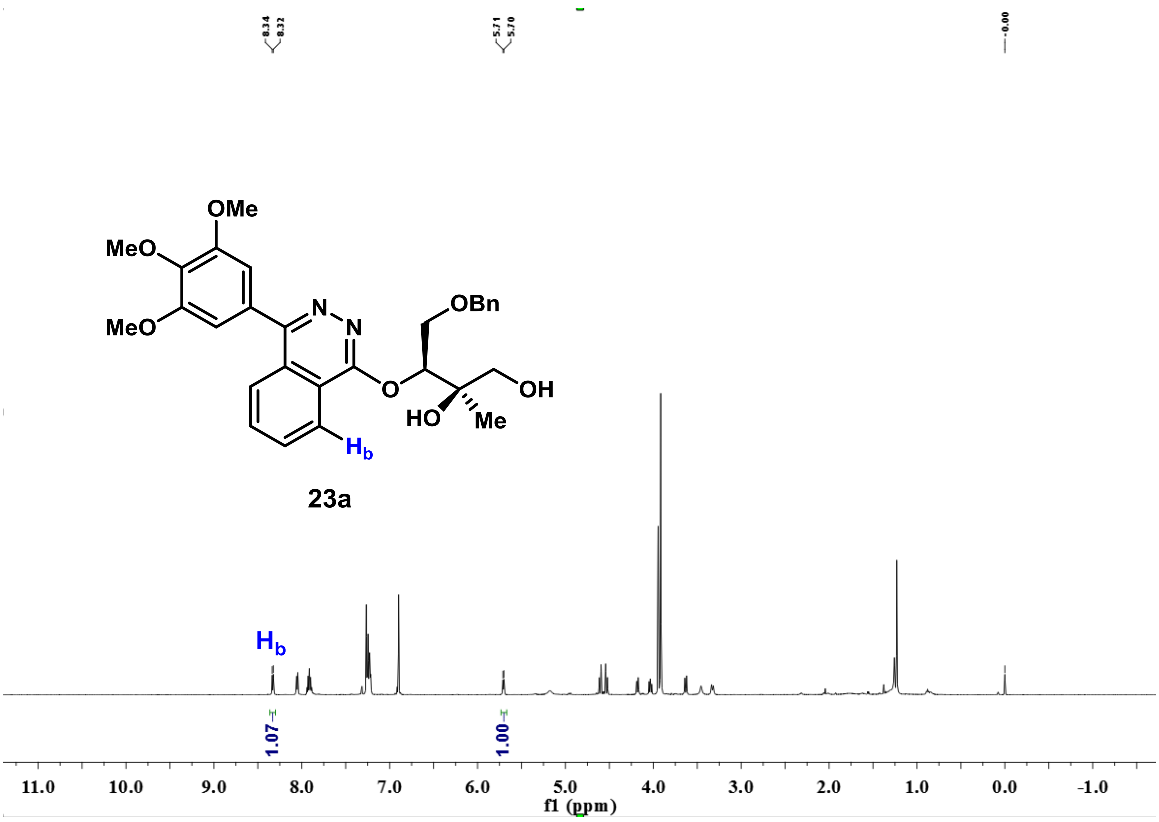

Supplementary Figure 146. ^{1}H NMR spectrum of dihydroxylated product **23a**.

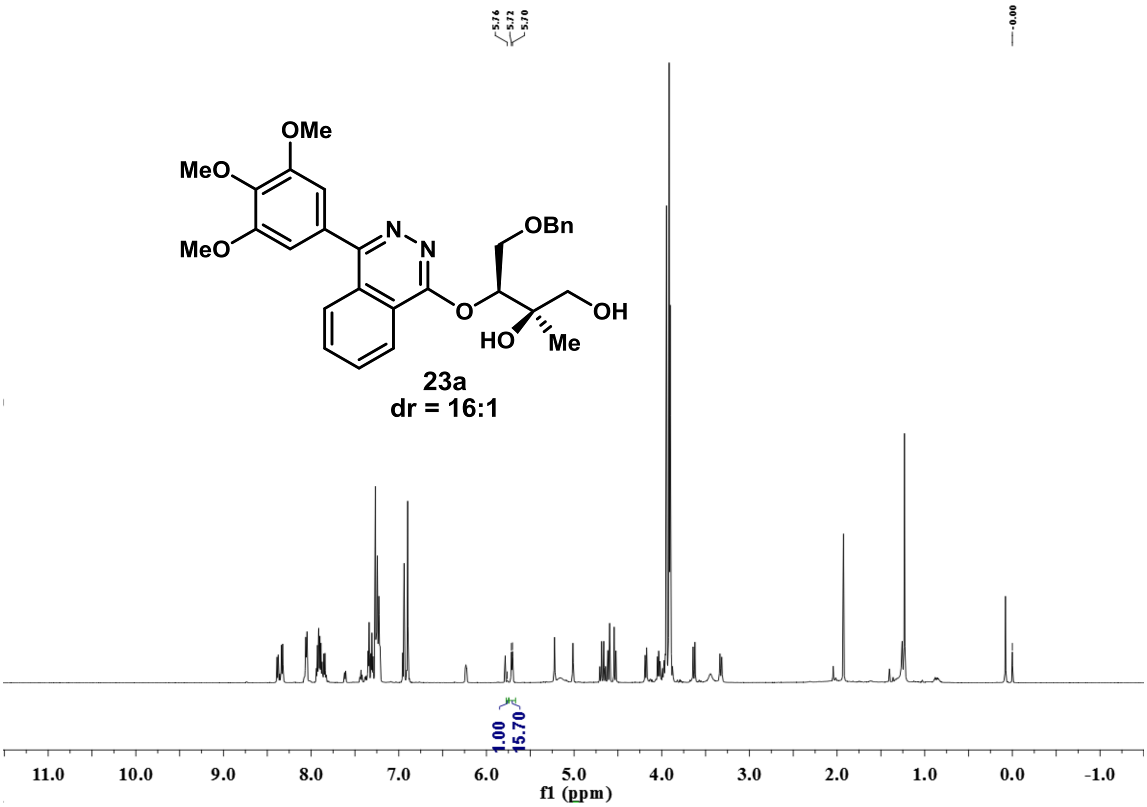

Supplementary Figure 147. ^{1}H NMR spectrum of crude mixture for diastereomeric ratio (dr).

HPLC (AD-H, 0.46*25 cm, 5μm, hexane/isopropanol = 80/20, flow = 1.0 mL/min, detection at 210 nm), retention time = 10.799 min (minor) and 13.921 min (major).

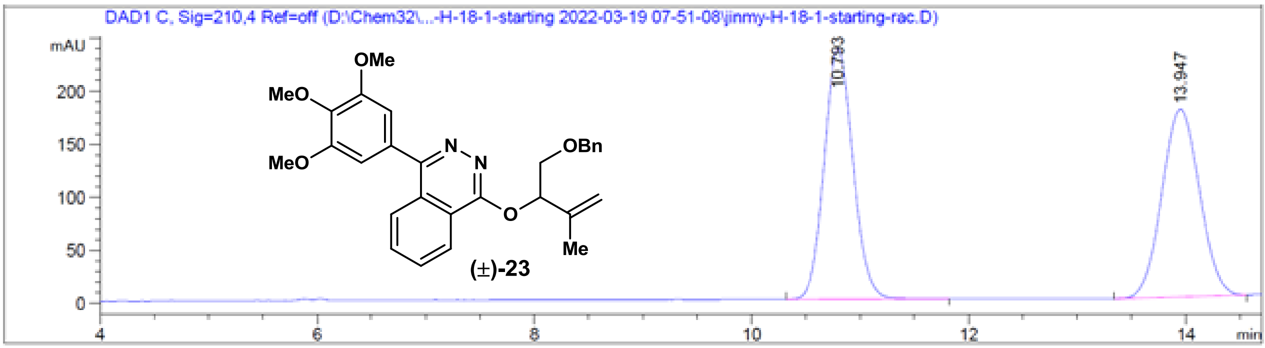

Signal 2: DAD1 C, Sig=210,4 Ref=off

Peak #	RetTime [min]	Type	Width [min]	Area [mAU*s]	Height [mAU]	Area %
1	10.793	BB	0.2874	4355.33301	235.90344	50.5989
2	13.947	BB	0.3763	4252.23047	176.98738	49.4011

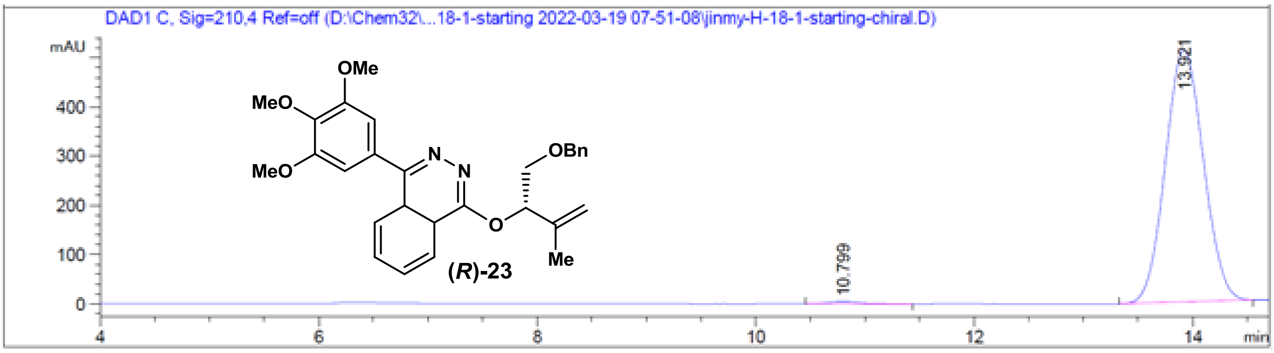

Signal 2: DAD1 C, Sig=210,4 Ref=off

Peak #	RetTime [min]	Type	Width [min]	Area [mAU*s]	Height [mAU]	Area %
1	10.799	BB	0.2746	93.11371	5.01573	0.7506
2	13.921	BB	0.3790	1.23123e4	511.26190	99.2494

Supplementary Figure 148. HPLC chromatogram for ***(R)*-23**.

HPLC (AD-H, 0.46*25 cm, 5μm, hexane/isopropanol = 80/20, flow = 1.0 mL/min, detection at 210 nm), retention time = 22.830 min (major) and 32.923 min (minor).

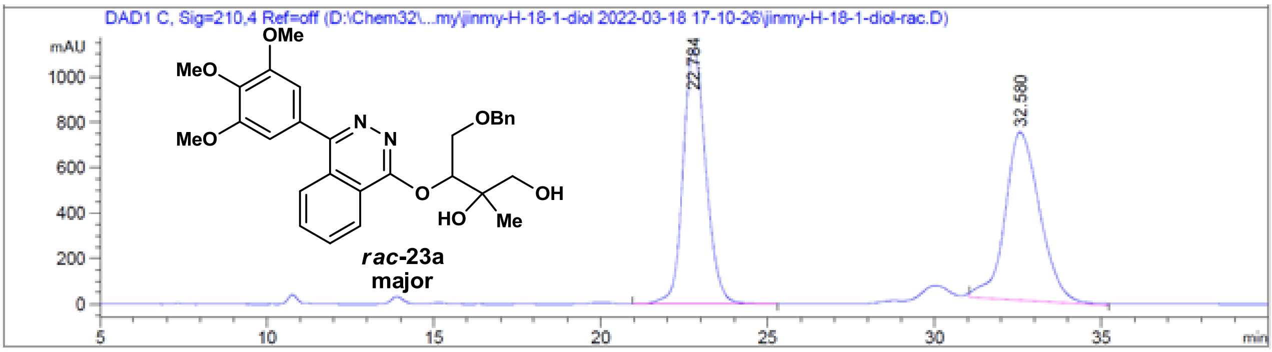

Signal 2: DAD1 C, Sig=210,4 Ref=off

Peak #	RetTime [min]	Type	Width [min]	Area [mAU*s]	Height [mAU]	Area %
1	22.784	BB	0.7347	5.31645e4	1118.11707	49.4806
2	32.580	MM R	1.2208	5.42807e4	741.02924	50.5194

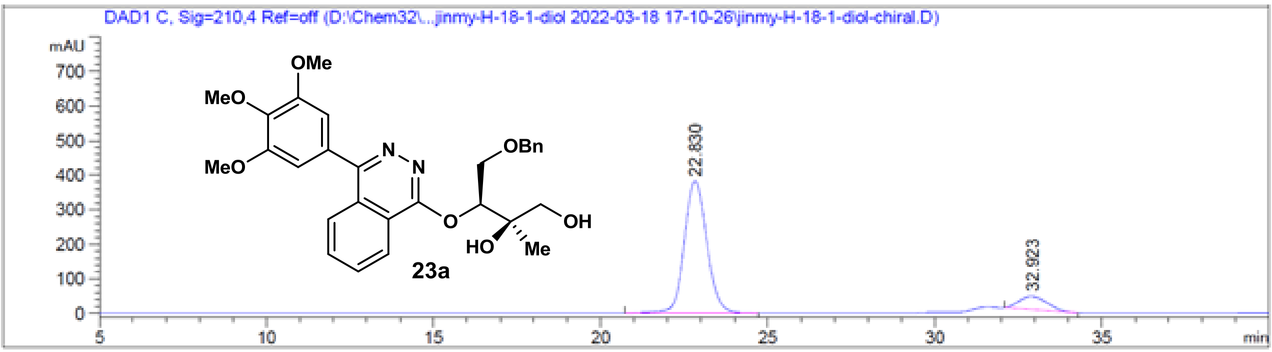

Signal 2: DAD1 C, Sig=210,4 Ref=off

Peak #	RetTime [min]	Type	Width [min]	Area [mAU*s]	Height [mAU]	Area %
1	22.830	BB	0.7283	1.80398e4	382.43927	88.8234
2	32.923	MF R	0.9728	2269.93872	38.89003	11.1766

Supplementary Figure 149. HPLC chromatogram for **23a**.

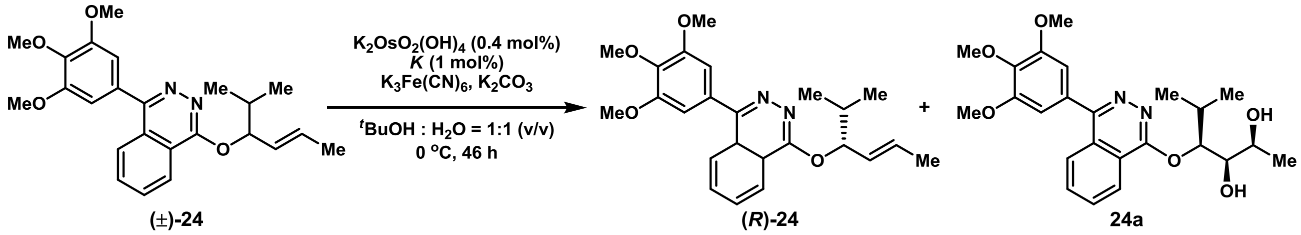

The general procedure **IV** was followed. The conversion of **(±)-24** was determined by crude ^{1}H NMR. **Conversion** (%) = [(2.55 – 1.00) / 2.55] % = 61%.

S = In [(1 – conv)(1 – ee)] / In [(1 – conv)(1 + ee)] = 13.

The recovered alkene **(*R*)-24** (14.3 mg, 35% yield, 94% ee) was purified by chromatography on silica gel (eluted with petroleum ether : ethyl acetate = 3:1). $[\alpha]_D^{25}$ = +15.28 (*c* 0.89, $CHCl_3$).

The dihydroxylated product **24a** (dr = 20:1, 29.6 mg, 55% yield, 57% ee) was purified by chromatography on silica gel (eluted with petroleum ether : ethyl acetate = 1:2). $[\alpha]_D^{25}$ = –10.56 (*c* 0.90, $CHCl_3$)

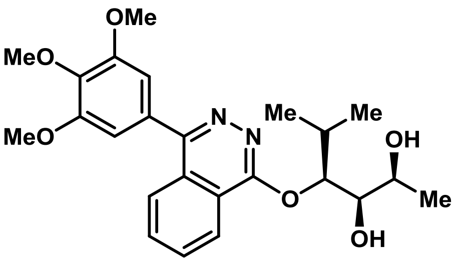

24a

(2*S*,3*R*,4*S*)-5-methyl-4-(4-(3,4,5-trimethoxyphenyl)phthalazin-1-yloxy)hexane-2,3-diol

^{1}H NMR (600 MHz, $CDCl_3$): δ 8.34 (d, *J* = 6.0 Hz, 1H), 8.06 (d, *J* = 12.0 Hz, 1H), 7.97 – 7.88 (m, 2H), 6.90 (s, 2H), 5.29 (d, *J* = 12.0 Hz, 1H), 4.97 (br, 1H), 3.95 (s, 3H), 3.92 (s, 6H), 3.89 – 3.83 (m, 1H), 3.64 (t, *J* = 6.0 Hz, 1H), 2.69 (d, *J* = 12.0 Hz, 1H), 2.56 – 2.46 (m, 1H), 1.29 (d, *J* = 6.0 Hz, 3H), 1.25 (d, *J* = 6.0 Hz, 3H), 1.02 (d, *J* = 6.0 Hz, 3H) ppm.

^{13}C NMR (151 MHz, $CDCl_3$): δ 161.6, 157.1, 153.4, 139.0, 132.9, 132.2, 131.4, 128.3, 126.5, 123.3, 120.4, 107.2, 82.2, 73.4, 64.8, 61.0, 56.3, 28.9, 20.4, 18.4, 15.5 ppm.

HRMS (ESI) m/z: $[M + H]^+$ Calcd for $C_{24}H_{31}N_2O_2$ 443.2177; Found 443.2182.

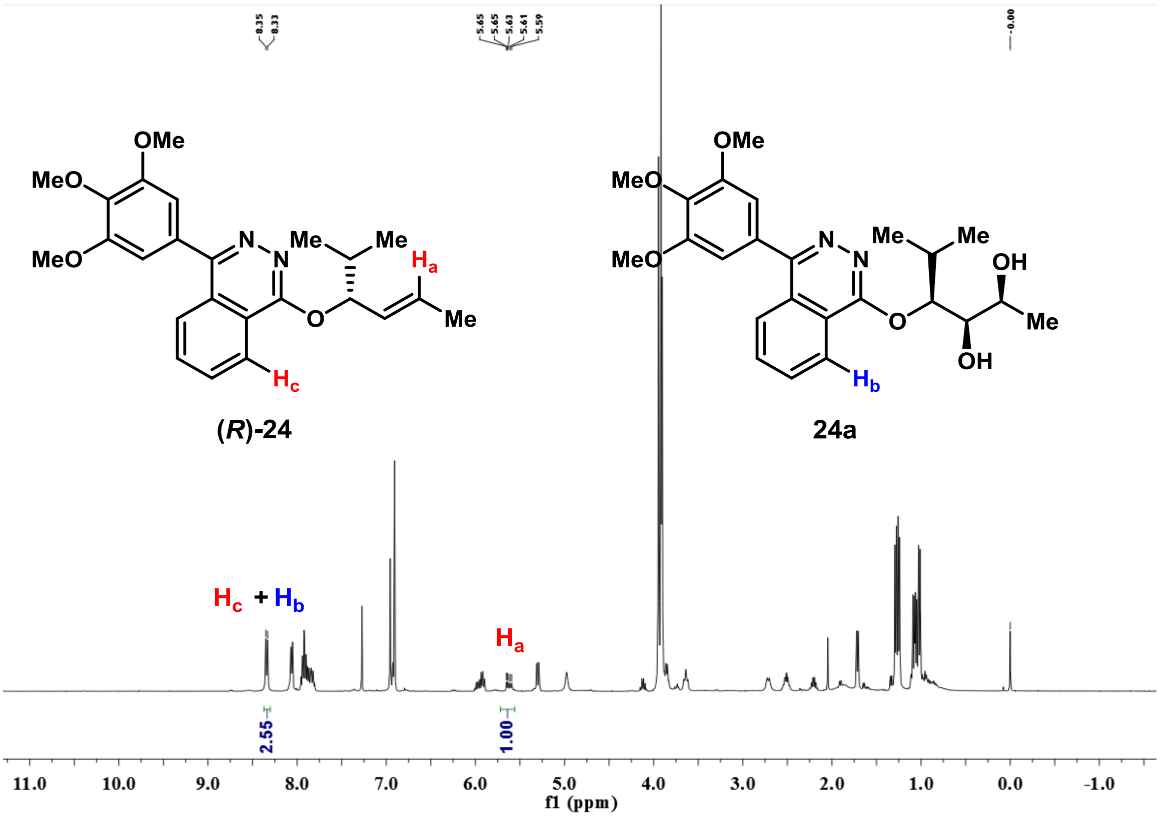

Supplementary Figure 150. ^{1}H NMR spectrum of crude mixture of compound **(*R*)-24** and **24a.**

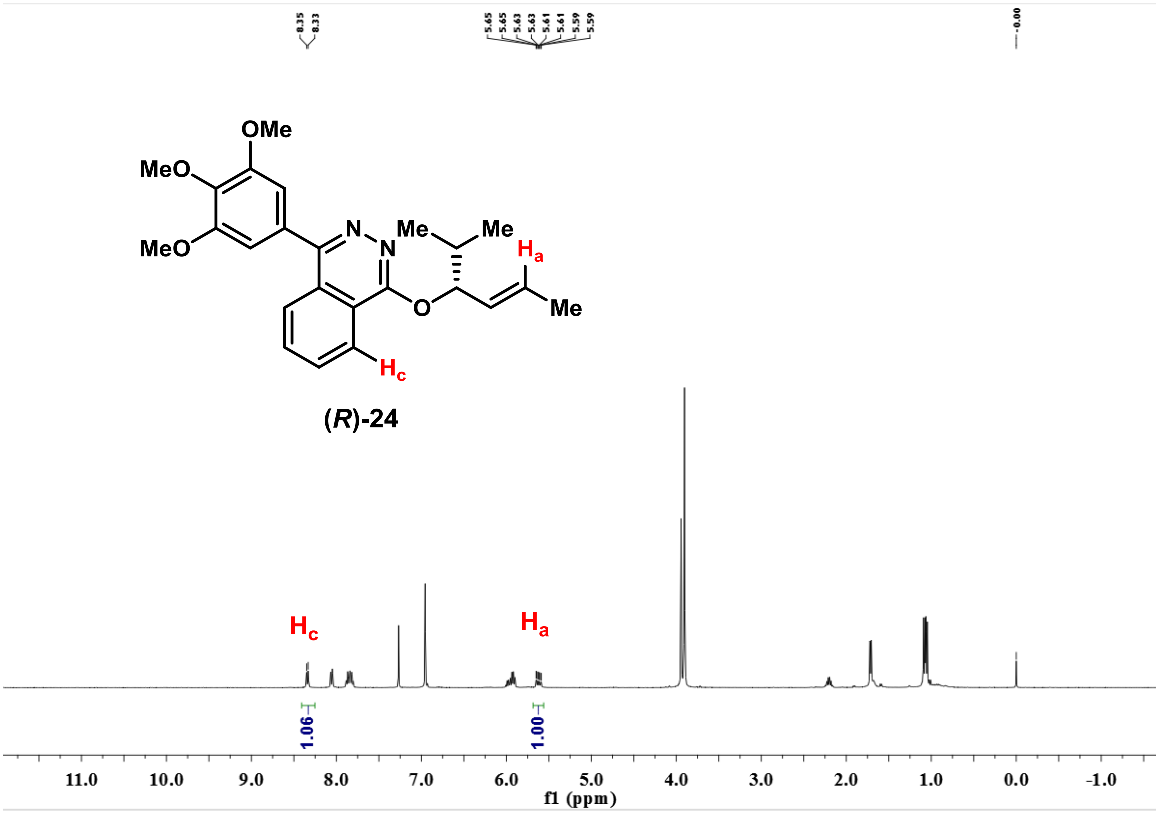

Supplementary Figure 151. ^{1}H NMR spectrum of recovered alkene **(*R*)-24**.

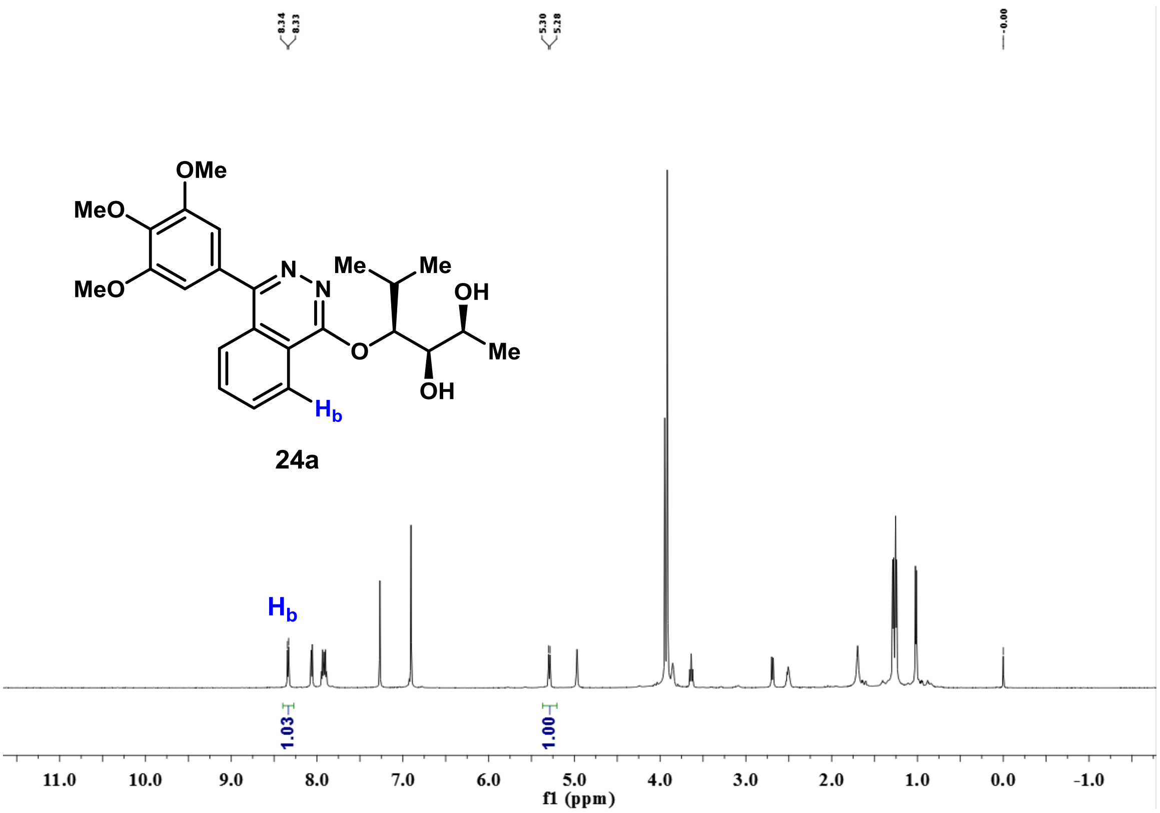

Supplementary figure 152. ^{1}H NMR spectrum of dihydroxylated product **24a**.

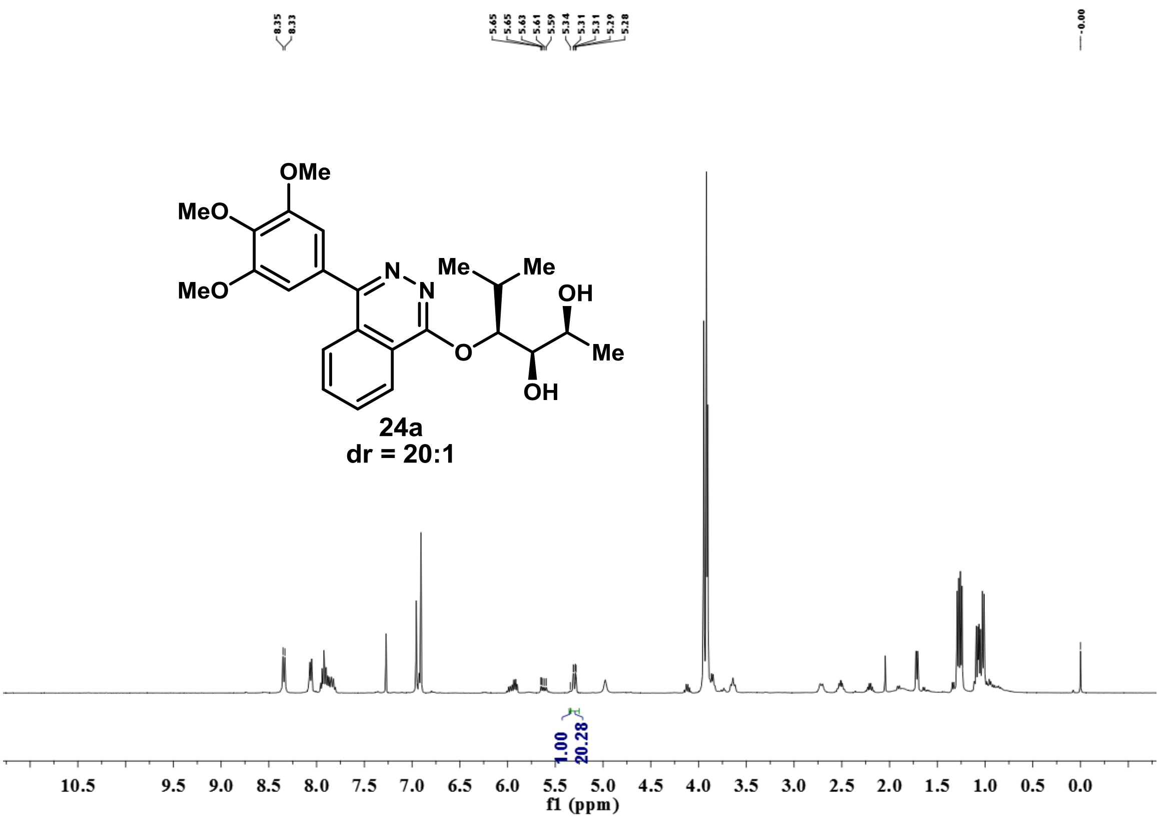

Supplementary Figure 153. ^{1}H NMR spectrum of crude mixture for diastereomeric ratio (dr).

HPLC (OD-H, 0.46*25 cm, 5μm, hexane/isopropanol = 98/2, flow = 1.0 mL/min, detection at 210 nm) retention time =34.607 min (minor) and 37.737 min (major).

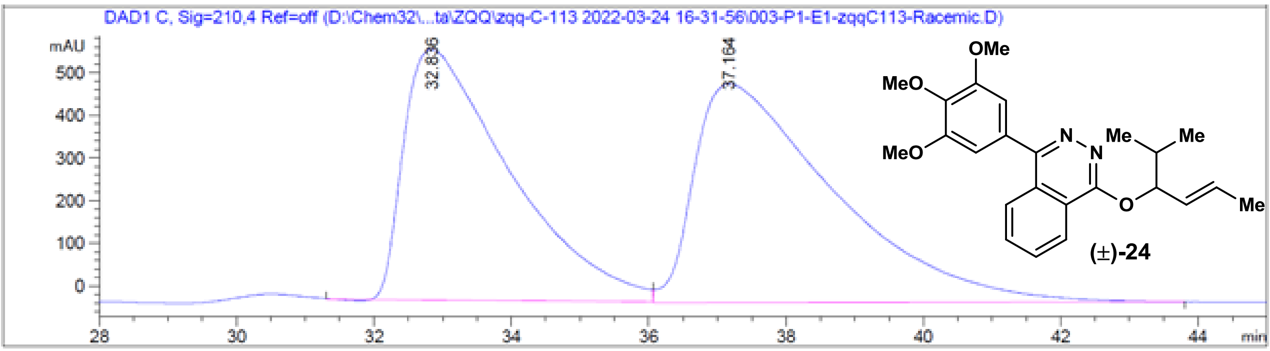

Signal 2: DAD1 C, Sig=210,4 Ref=off

Peak #	RetTime [min]	Type	Width [min]	Area [mAU*s]	Height [mAU]	Area %
1	32.836	MM R	1.7952	6.34176e4	588.78241	46.4914
2	37.164	VB	1.8336	7.29896e4	513.11353	53.5086

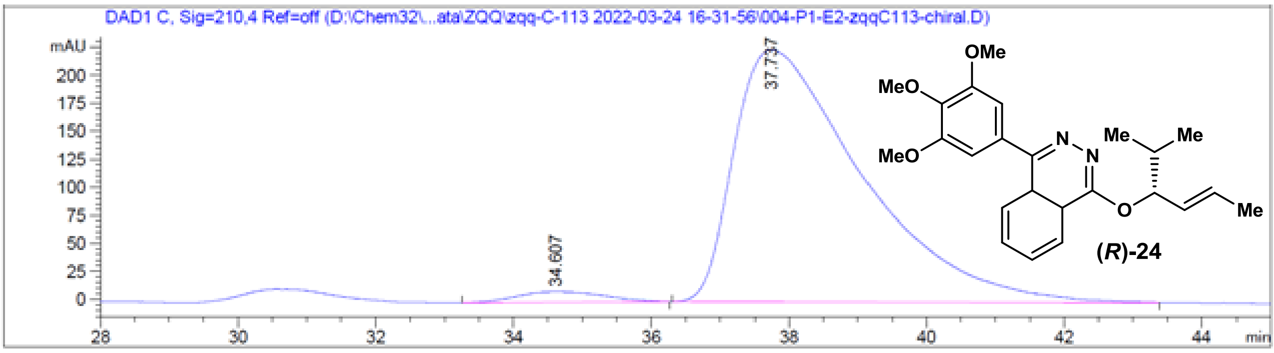

Signal 2: DAD1 C, Sig=210,4 Ref=off

Peak #	RetTime [min]	Type	Width [min]	Area [mAU*s]	Height [mAU]	Area %
1	34.607	BB	1.0413	853.42828	9.65414	2.8441
2	37.737	BB	1.7075	2.91540e4	225.32011	97.1559

Supplementary Figure 154. HPLC chromatogram for ***(R)*-24**.

HPLC (OJ-H, 0.46*25 cm, 5μm, hexane/isopropanol = 85/15, flow = 1.0 mL/min, detection at 210 nm) retention time = 14.117 min (minor) and 20.482 min (major).

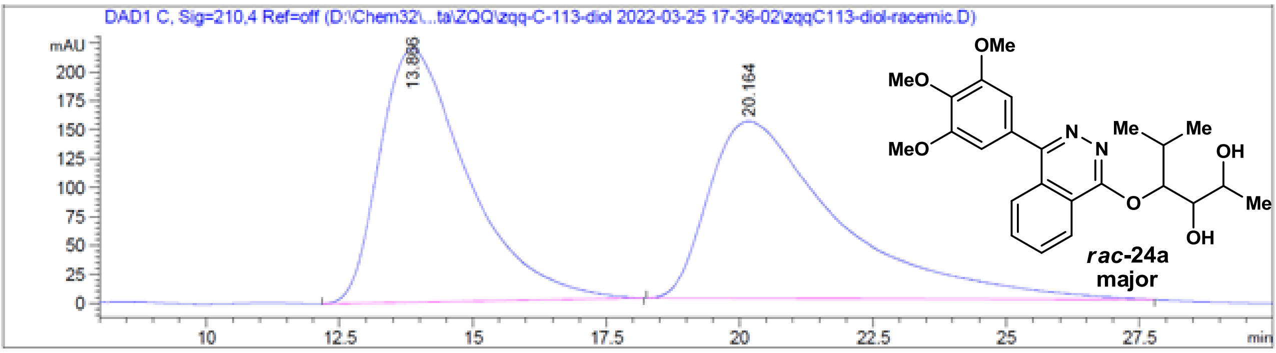

Signal 2: DAD1 C, Sig=210,4 Ref=off

Peak #	RetTime [min]	Type	Width [min]	Area [mAU*s]	Height [mAU]	Area %
1	13.866	BB	1.4057	2.52113e4	218.90302	49.3250
2	20.164	BB	1.9991	2.59013e4	153.30450	50.6750

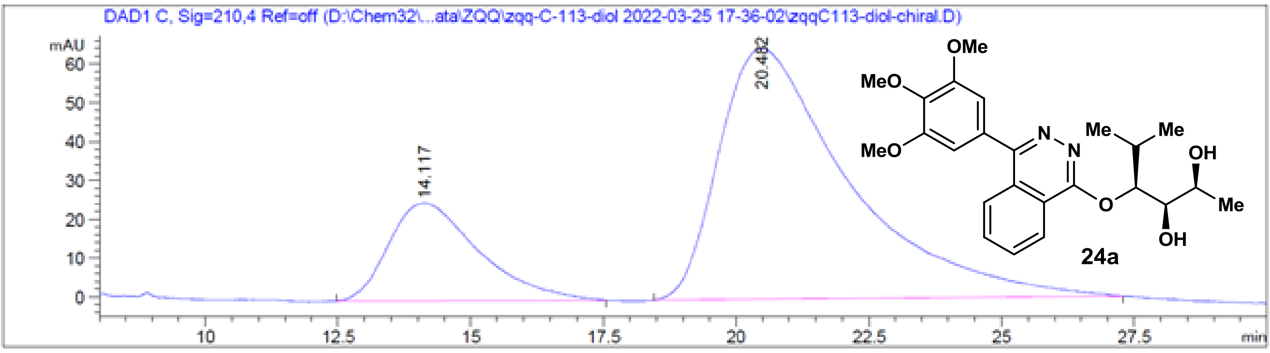

Peak #	RetTime [min]	Type	Width [min]	Area [mAU*s]	Height [mAU]	Area %
1	14.117	BB	1.3790	2959.37866	25.18233	21.1858
2	20.482	BB	2.0111	1.10093e4	64.50692	78.8142

Supplementary Figure 155. HPLC chromatogram for **24a**.

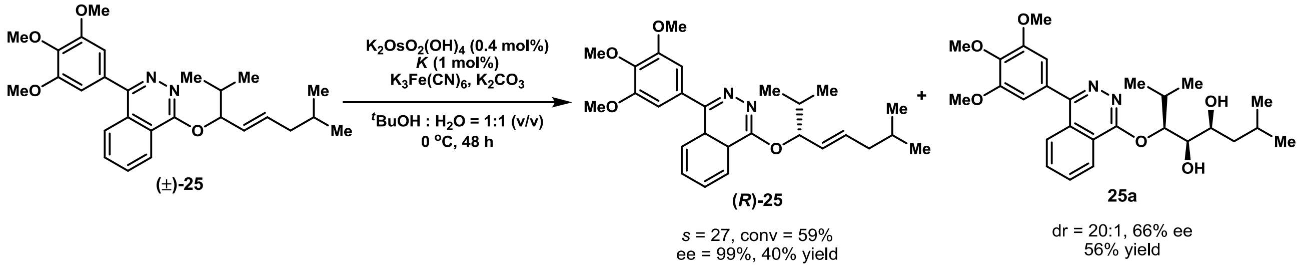

The general procedure **IV** was followed. The conversion of **(±)-25** was determined by crude ^{1}H NMR. **Conversion** (%) = [(2.44 – 1.00) / 2.44] % = 59%.

S = In [(1 – conv)(1 – ee)] / In [(1 – conv)(1 + ee)] = 27.

The recovered alkene **(*R*)-25** (18.1 mg, 40% yield, 99% ee) was purified by chromatography on silica gel (eluted with petroleum ether : ethyl acetate = 3:1). $[\alpha]_D^{25}$ = –8.62 (*c* 0.70, $CHCl_3$).

The dihydroxylated product **25a** (dr = 20:1, 27.1 mg, 56% yield, 66% ee) was purified by chromatography on silica gel (eluted with petroleum ether : ethyl acetate = 1:2). $[\alpha]_D^{25}$ = – 32.73 (*c* 0.55, $CHCl_3$)

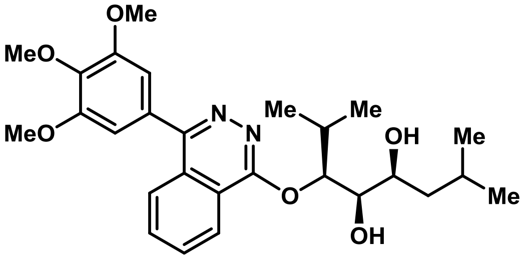

25a

(3*S*,4*R*,5*S*)-2,7-dimethyl-3-(4-(3,4,5-trimethoxyphenyl)phthalazin-1-yloxy)octane-4,5-diol

^{1}H NMR (600 MHz, $CDCl_3$): δ 8.36 (d, *J* = 6.0 Hz, 1H), 8.09 (d, *J* = 6.0 Hz, 1H), 7.97 – 7.89 (m, 2H), 6.92 (s, 2H), 5.30 (d, *J* = 12.0 Hz, 1H), 4.88 (s, 1H), 3.95 (s, 3H), 3.92 (s, 6H), 3.76 – 3.70 (m, 1H), 3.68 (t, *J* = 6.0 Hz, 1H), 2.75 (d, *J* = 6.0 Hz, 1H), 2.56 – 2.45 (m 1H), `1.82 –1.74 (m, 2H), 1.72 – 1.66 (m, 1H), 1.38 – 1.31 (m, 1H), 1.25 (d, *J* = 6.0Hz, 3H), 1.01 (d, *J* = 6.0 Hz, 3H), 0.87 (d, *J* = 6.0 Hz, 3H), 0.80 (d, *J* = 6.0 Hz, 3H) ppm.

^{13}C NMR (151 MHz, $CDCl_3$): δ 161.8, 157.0, 153.4, 139.0, 132.9, 132.2, 131.4, 128.3, 126.6, 123.4, 120.4, 107.2, 82.5, 72.5, 66.9, 61.0, 56.4, 41.4, 29.7, 29.0, 24.6, 23.2, 22.4, 20.4, 15.5 ppm.

HRMS (ESI) m/z: $[M + H]^+$ Calcd for $C_{27}H_{37}N_2O_2$ 485.2646; Found 485.2639.

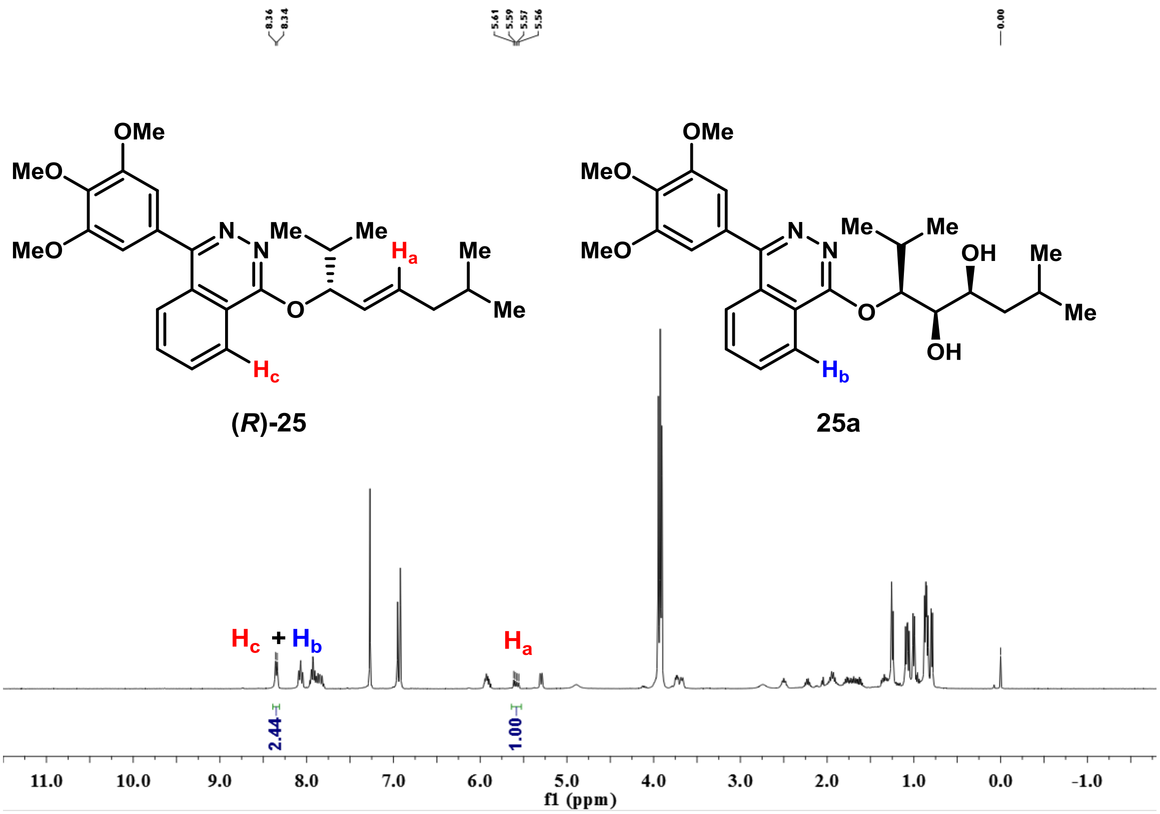

Supplementary Figure 156. ^{1}H NMR spectrum of crude mixture of compound **(*R*)-25** and **25a.**

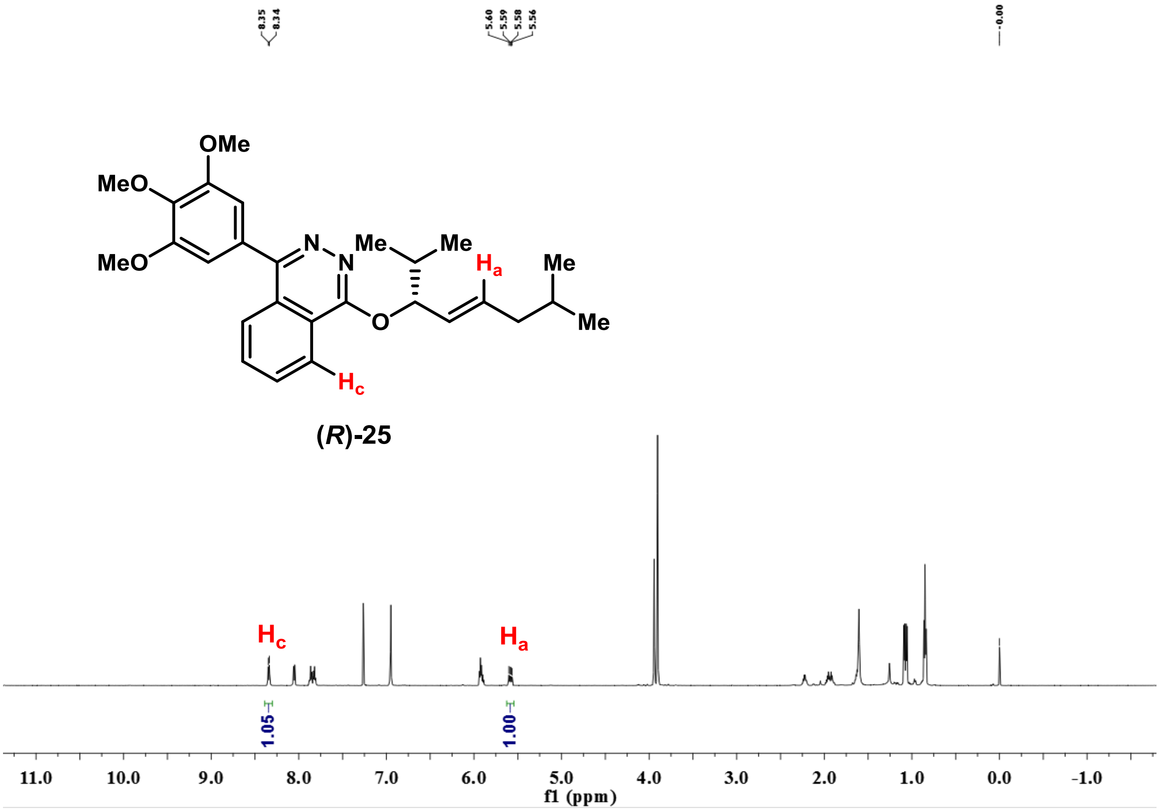

Supplementary Figure 157. ^{1}H NMR spectrum of recovered alkene **(*R*)-25**.

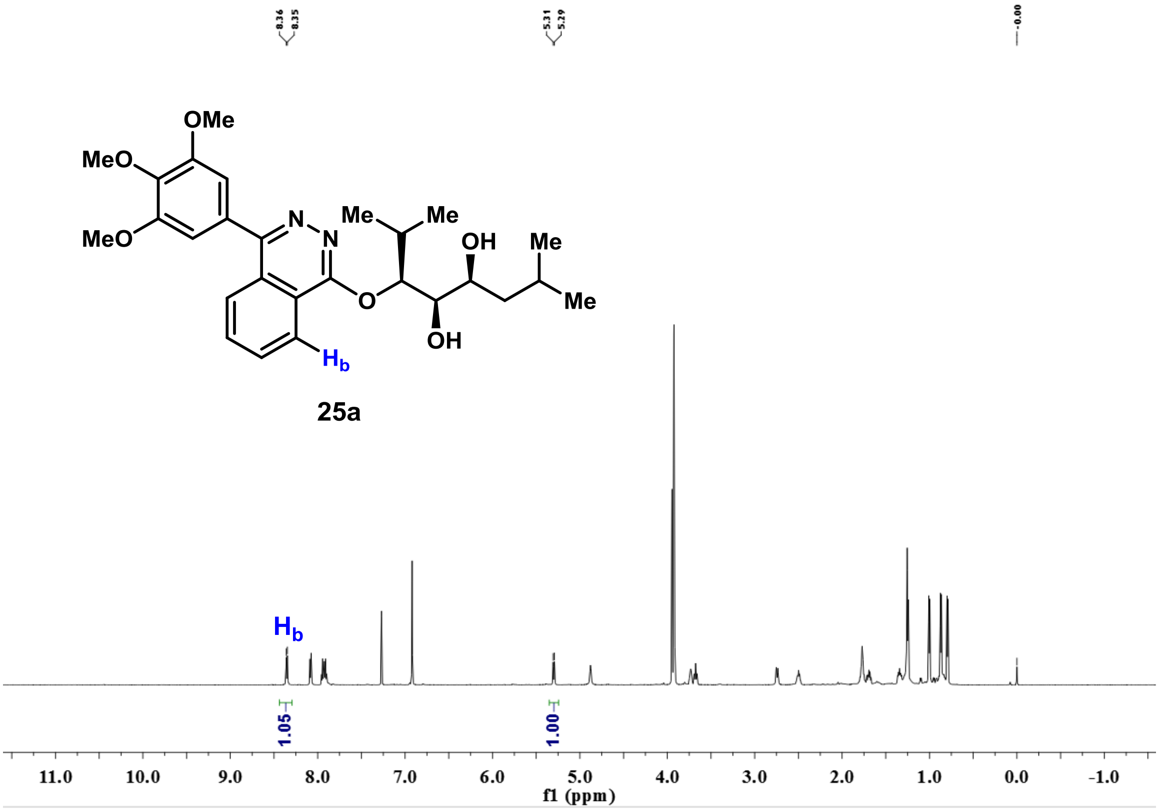

Supplementary Figure 158. ^{1}H NMR spectrum of dihydroxylated product **25a**.

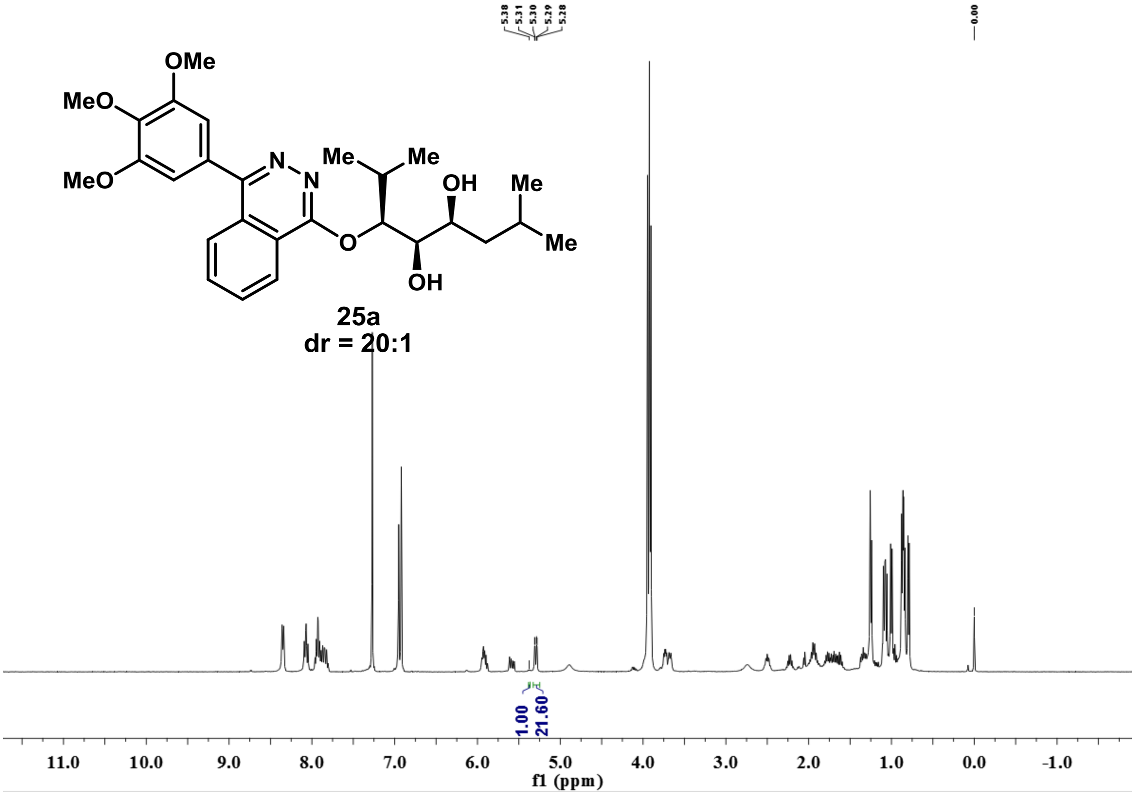

Supplementary Figure 159. ^{1}H NMR spectrum of crude mixture for diastereomeric ratio (dr).

HPLC (AD-H, 0.46*25 cm, 5μm, hexane/isopropanol = 90/10, flow = 1.0 mL/min, detection at 254 nm), retention time = 8.542 min (major) and 15.620 min (minor).

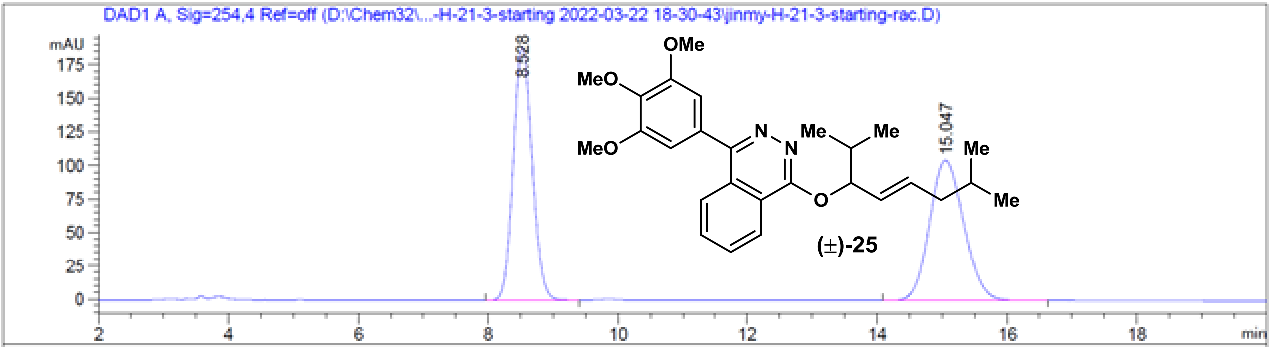

Signal 1: DAD1 A, Sig=254,4 Ref=off

Peak #	RetTime [min]	Type	Width [min]	Area [mAU*s]	Height [mAU]	Area %
1	8.528	BB	0.3142	3829.61548	189.15508	49.9348
2	15.047	BB	0.5667	3839.61426	104.97179	50.0652

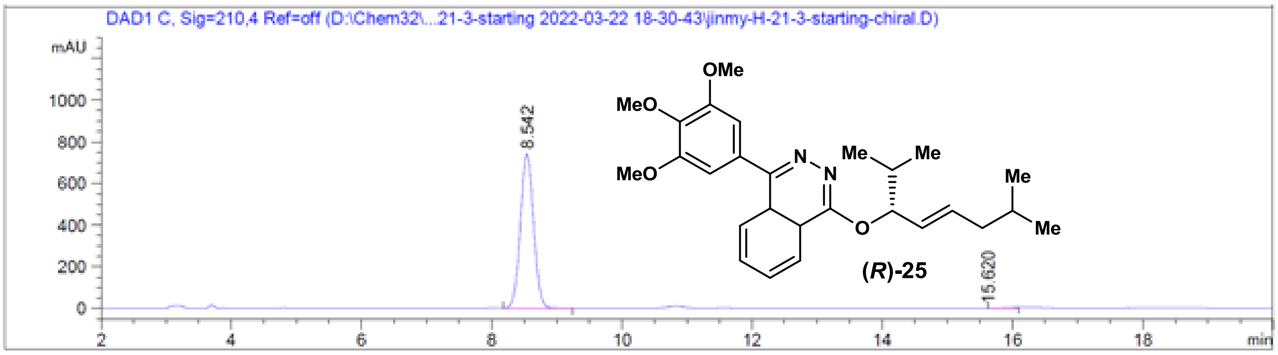

Signal 2: DAD1 C, Sig=210,4 Ref=off

Peak #	RetTime [min]	Type	Width [min]	Area [mAU*s]	Height [mAU]	Area %
1	8.542	MM R	0.2380	1.05758e4	740.55096	99.3976
2	15.620	MM R	0.2061	64.09294	3.02731e-1	0.6024

Supplementary Figure 160. HPLC chromatogram for ***(R)*-25**.

HPLC (OJ-H, 0.46*25 cm, 5μm, hexane/isopropanol = 90/10, flow = 1.0 mL/min, detection at 254 nm), retention time = 12.086 min (minor) and 19.063 min (major).

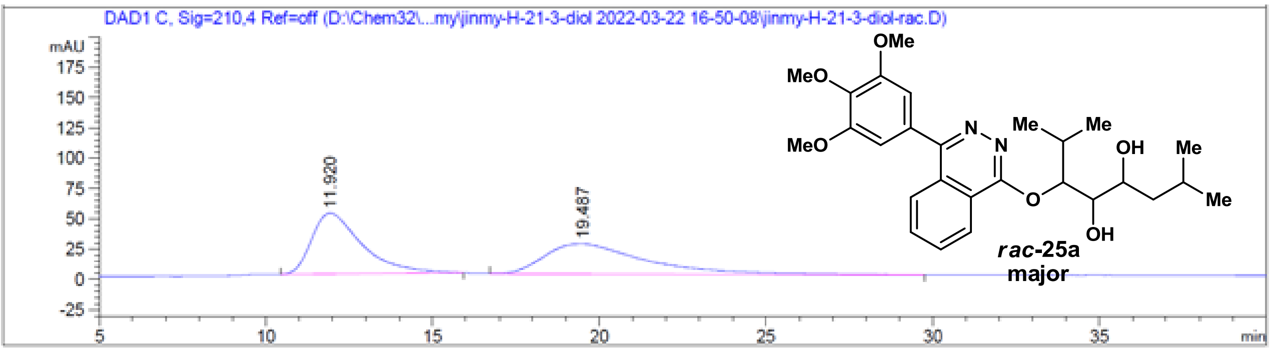

Signal 2: DAD1 C, Sig=210,4 Ref=off

Peak #	RetTime [min]	Type	Width [min]	Area [mAU*s]	Height [mAU]	Area %
1	11.920	BB	1.2407	5278.64307	50.20443	49.8968
2	19.487	MM R	3.5136	5300.48242	25.14295	50.1032

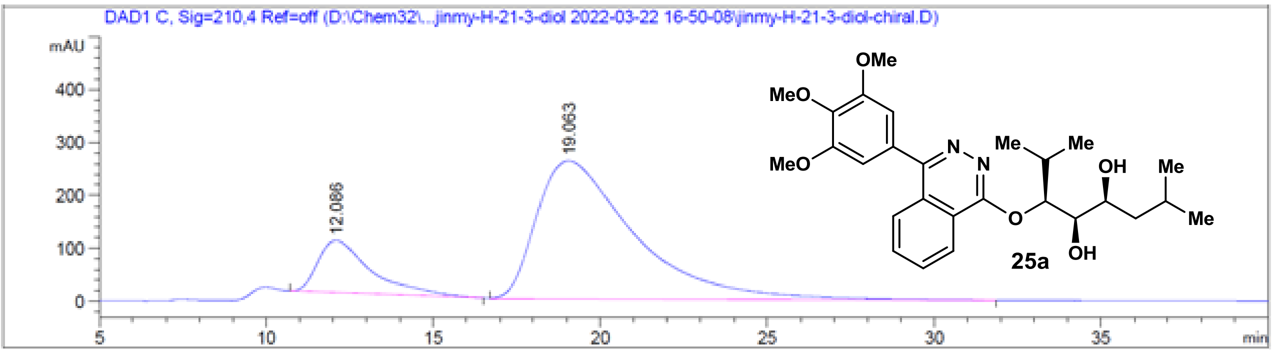

Signal 2: DAD1 C, Sig=210,4 Ref=off

Peak #	RetTime [min]	Type	Width [min]	Area [mAU*s]	Height [mAU]	Area %
1	12.086	BB	1.3883	1.08469e4	98.37897	16.9616
2	19.063	MM R	3.3885	5.31029e4	261.19171	83.0384

Supplementary Figure 161. HPLC chromatogram for **25a**.

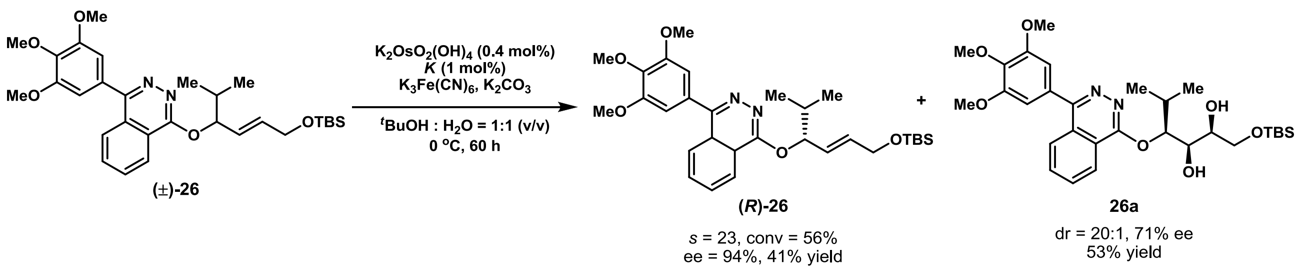

The general procedure **IV** was followed. The conversion of **(±)-26** was determined by crude ^{1}H NMR. **Conversion** (%) = [(2.29 – 1.00) / 2.29] % = 56%.

S = In [(1 – conv)(1 – ee)] / In [(1 – conv)(1 + ee)] = 23.

The recovered alkene **(*R*)-26** (22.1 mg, 41% yield, 94% ee) was purified by chromatography on silica gel (eluted with petroleum ether : ethyl acetate = 3:1). $[\alpha]_D^{25}$ = –20.68 (*c* 0.56, $CHCl_3$).

The dihydroxylated product **26a** (dr = 20:1, 30.3 mg, 53% yield, 71% ee) was purified by chromatography on silica gel (eluted with petroleum ether : ethyl acetate = 1:2). $[\alpha]_D^{25}$ = – 17.16 (*c* 0.95, $CHCl_3$)

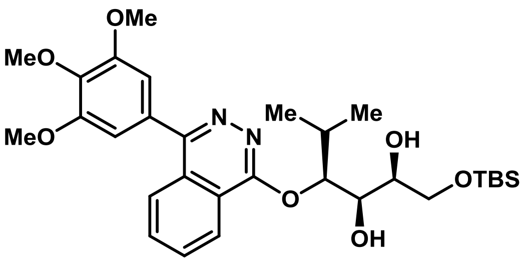

26a

(2*S*,3*R*,4*S*)-1-(tert-butyldimethylsilyloxy)-5-methyl-4-(4-(3,4,5-trimethoxyphenyl)phthalazin-1-yloxy)hexane-2,3-diol

^{1}H NMR (600 MHz, $CDCl_3$): δ 8.35 (d, *J* = 6.0 Hz, 1H), 8.06 (d, *J* = 6.0 Hz, 1H), 7.96 – 7.86 (m, 2H), 6.90 (s, 2H), 5.38 (d, *J* = 12.0 Hz, 1H), 4.81 (br, 1H), 4.04 (*J* = 12.0 Hz, 1H), 3.94 (s, 3H), 3.92 (s, 6H), 3.84 – 3.79 (m, 1H), 3.78 – 3.73 (m, 1H), 3.71 – 3.66 (m, 1H), 3.17 (br, 1H), 2.56 – 2.45 (m, 1H), 1.26 (*J* = 6.0 Hz, 3H), 1.03 (d, *J* = 6.0 Hz, 3H), 0.88 (s, 9H), 0.08 (s, 3H), 0.04 (s, 3H) ppm.

^{13}C NMR (151 MHz, $CDCl_3$): δ 161.6, 157.0, 153.4, 139.0, 132.8, 132.1, 131.4, 128.2, 126.5, 123.4, 120.4, 107.2, 81.8, 69.8, 69.0, 64.0, 61.0, 56.3, 29.7, 28.9, 25.9, 20.3, 18.3, 15.6, -5.4 ppm.

HRMS (ESI) m/z: $[M + H]^+$ Calcd for $C_{30}H_{45}N_2O_7Si$ 573.2991; Found 573.2983.

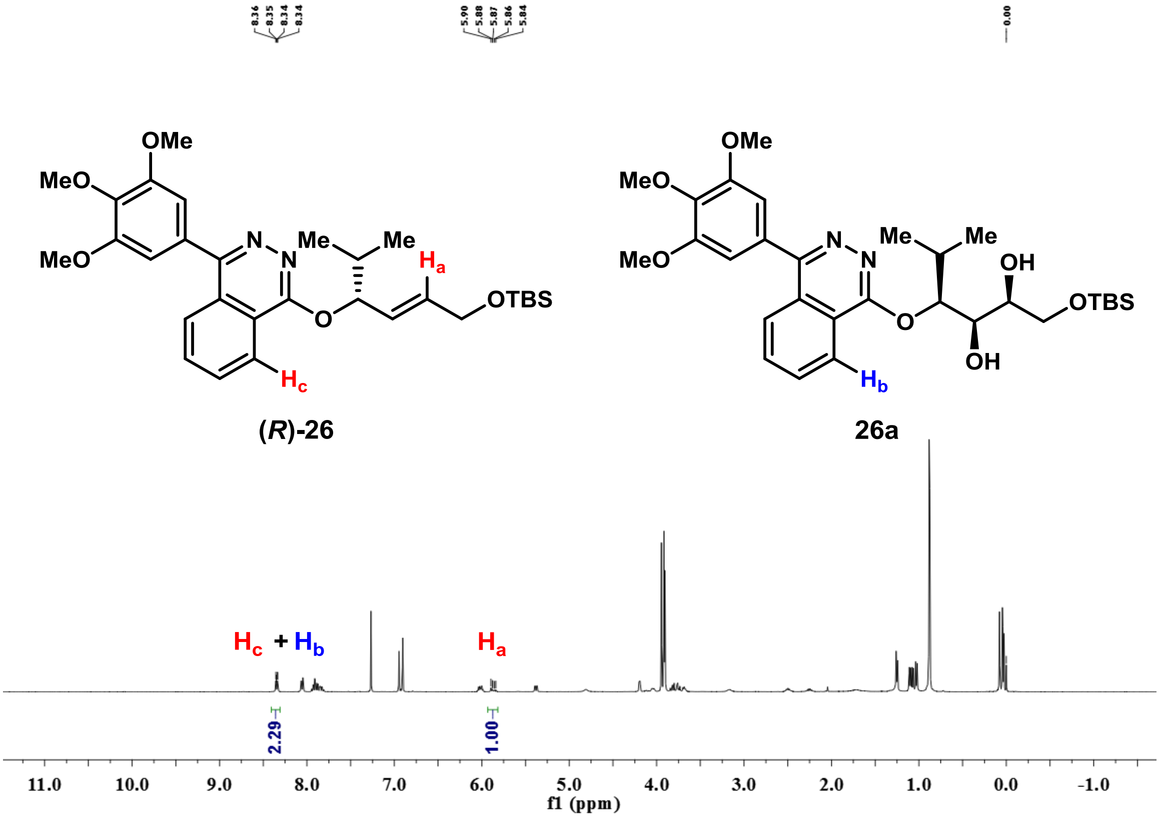

Supplementary Figure 162. ^{1}H NMR spectrum of crude mixture of compound **(*R*)-26** and **26a.**

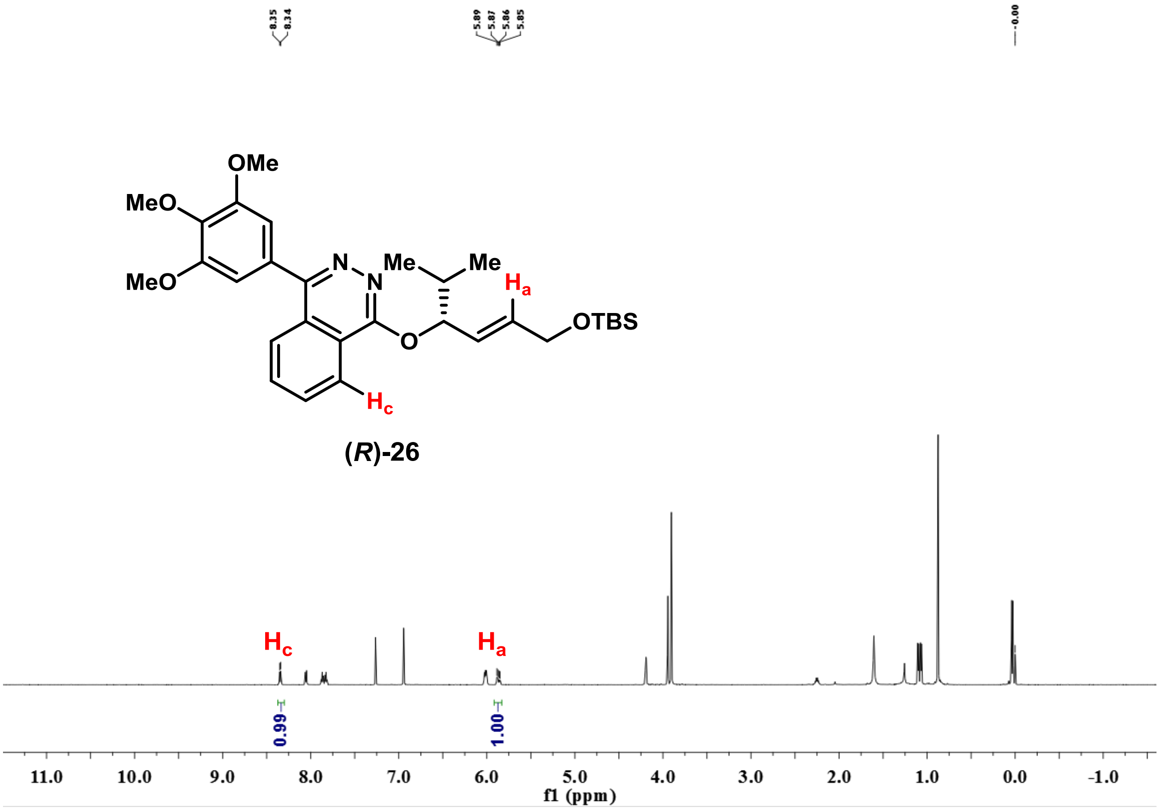

Supplementary Figure 163. ^{1}H NMR spectrum of recovered alkene **(*R*)-26**.

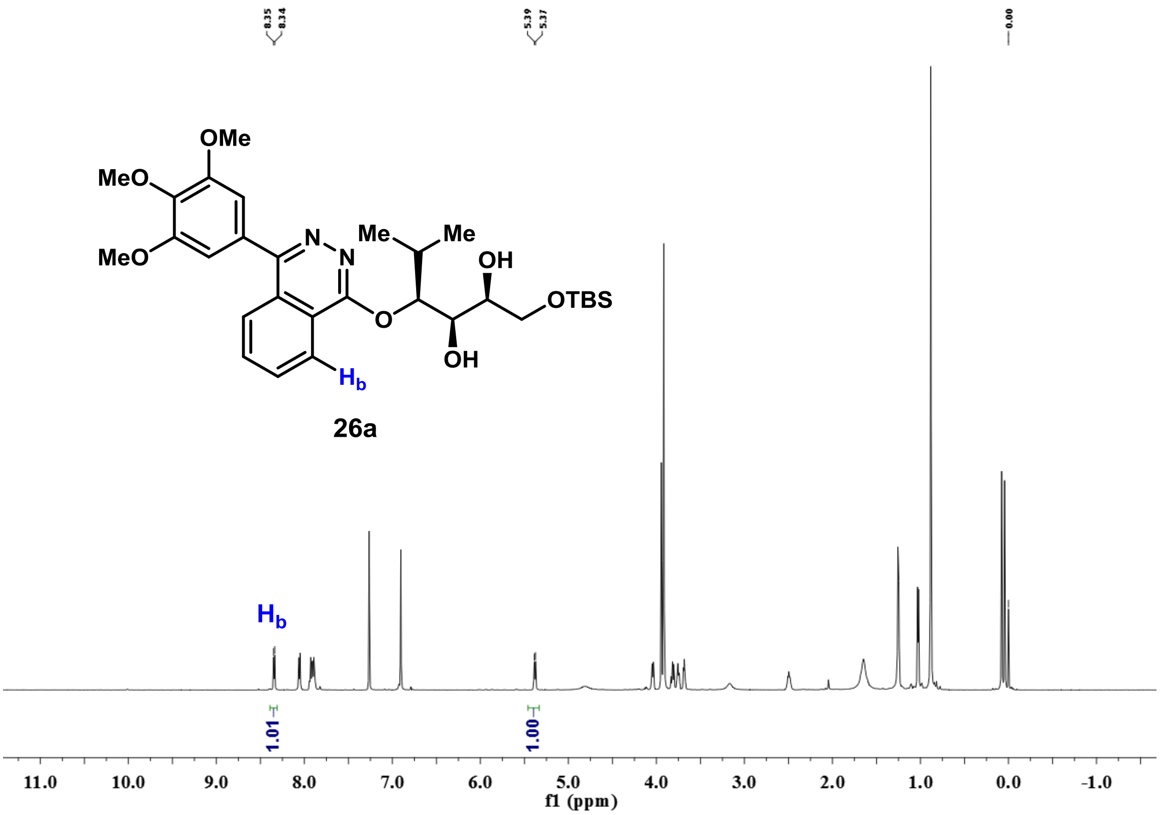

Supplementary Figure 164. ^{1}H NMR spectrum of dihydroxylated product **26a**.

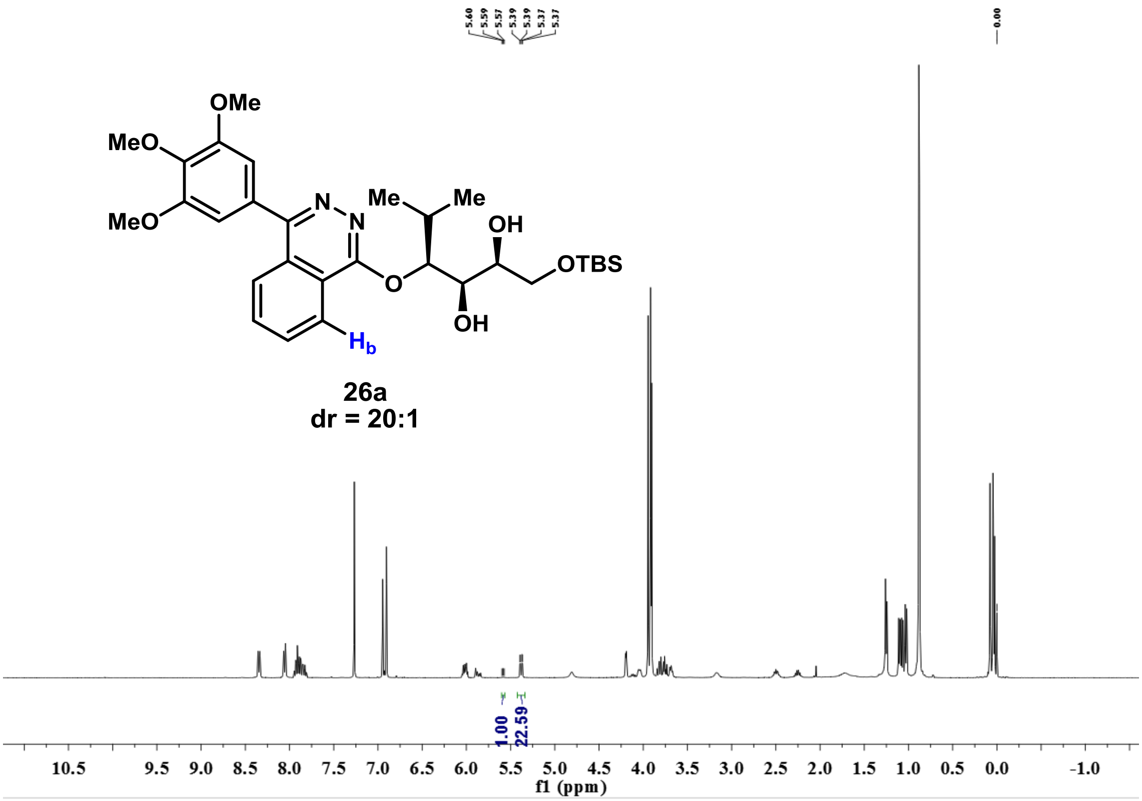

Supplementary Figure 165. ^{1}H NMR spectrum of crude mixture for diastereomeric ratio (dr).

HPLC (AD-H, 0.46*25 cm, 5μm, hexane/isopropanol = 90/10, flow = 1.0 mL/min, detection at 210 nm), retention time = 9.226 min (major) and 15.958 min (minor).

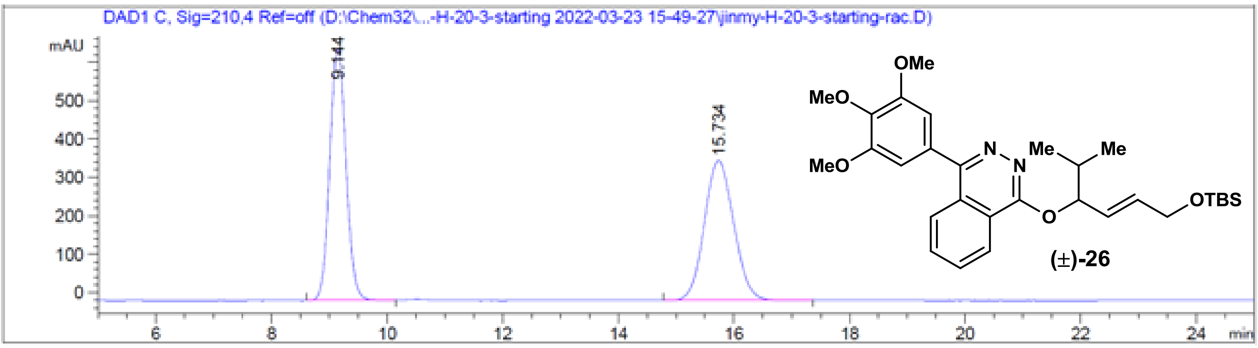

Signal 2: DAD1 C, Sig=210,4 Ref=off

Peak #	RetTime [min]	Type	Width [min]	Area [mAU*s]	Height [mAU]	Area %
1	9.144	BB	0.3040	1.28099e4	655.51349	49.9218
2	15.734	BB	0.5517	1.28500e4	364.12115	50.0782

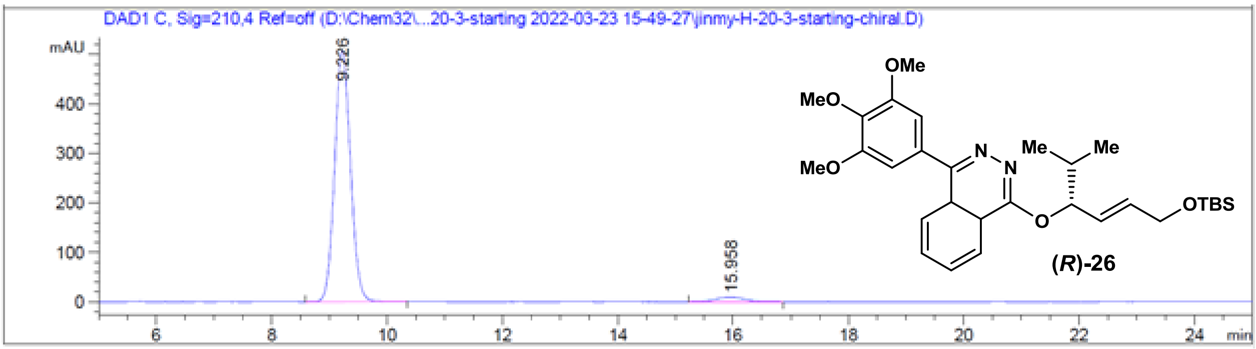

Signal 2: DAD1 C, Sig=210,4 Ref=off

Peak #	RetTime [min]	Type	Width [min]	Area [mAU*s]	Height [mAU]	Area %
1	9.226	BB	0.3031	9888.01270	508.07224	96.7767
2	15.958	BB	0.4284	329.33408	9.36244	3.2233

Supplementary Figure 166. HPLC chromatogram for **(*R*)-26**.

HPLC (AD-H, 0.46*25 cm, 5μm, hexane/isopropanol = 80/20, flow = 1.0 mL/min, detection at 210 nm), retention time = 12.720 min (minor) and 21.212 min (major).

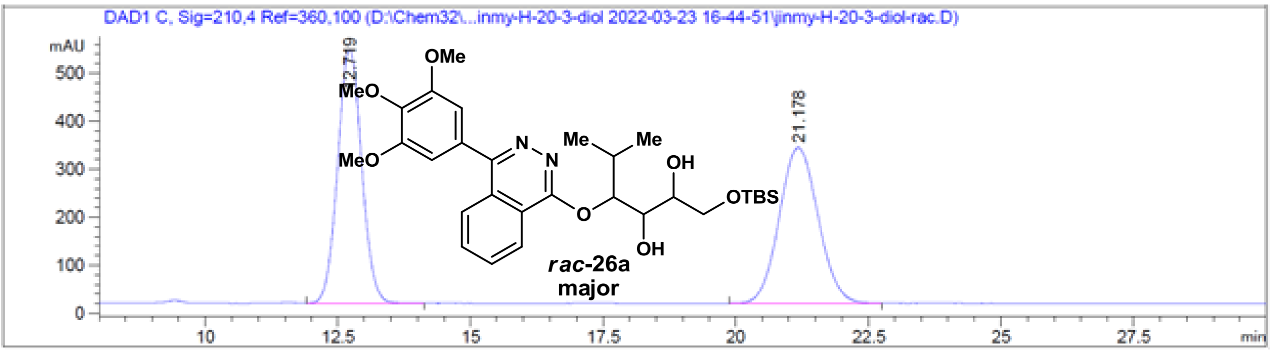

Signal 2: DAD1 C, Sig=210,4 Ref=360,100

Peak #	RetTime [min]	Type	Width [min]	Area [mAU*s]	Height [mAU]	Area %
1	12.719	BB	0.4913	1.66680e4	529.49963	50.1023
2	21.178	BB	0.7862	1.65999e4	325.92383	49.8977

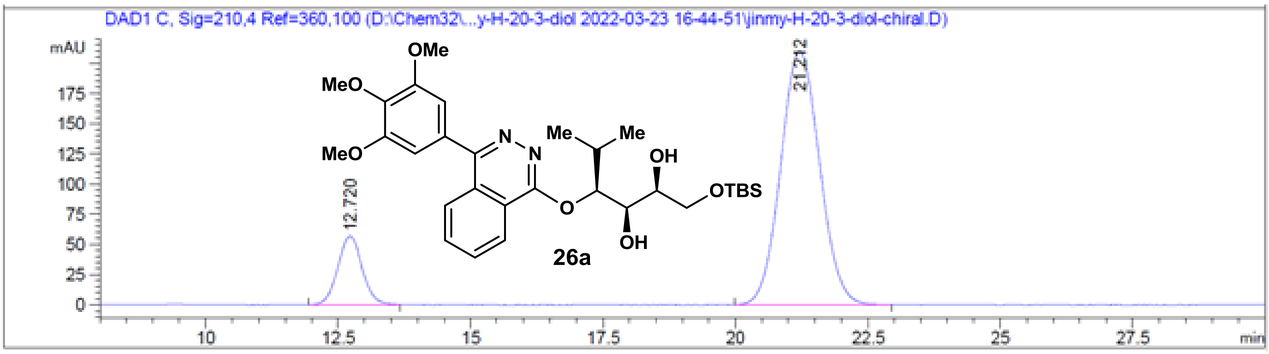

Signal 2: DAD1 C, Sig=210,4 Ref=360,100

Peak #	RetTime [min]	Type	Width [min]	Area [mAU*s]	Height [mAU]	Area %
1	12.720	BB	0.4872	1805.14417	56.43466	14.4015
2	21.212	BB	0.8026	1.07293e4	209.80414	85.5985

Supplementary Figure 167. HPLC chromatogram for **26a**.

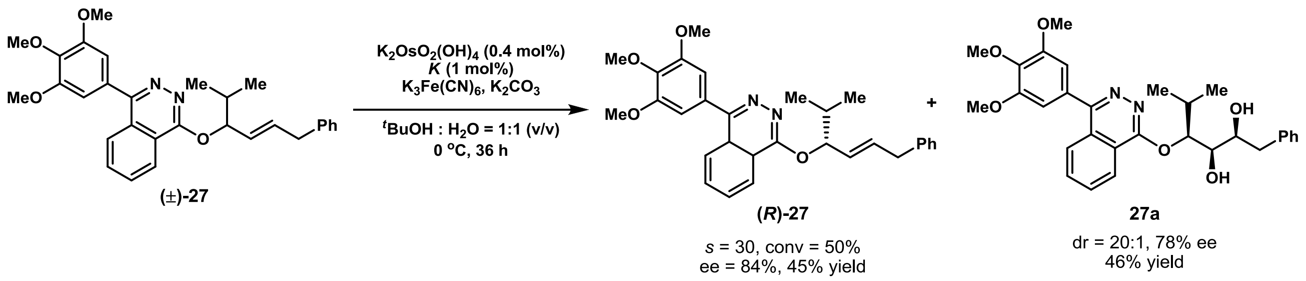

The general procedure **IV** was followed. The conversion of **(±)-27** was determined by crude ^{1}H NMR. **Conversion** (%) = [(2.03 – 1.00) / 2.03] % = 50%.

S = In [(1 – conv)(1 – ee)] / In [(1 – conv)(1 + ee)] = 30.

The recovered alkene **(*R*)-27** (24.2 mg, 45% yield, 84% ee) was purified by chromatography on silica gel (eluted with petroleum ether : ethyl acetate = 3:1). $[\alpha]_D^{25}$ = –2.35 (*c* 1.15, $CHCl_3$).

The dihydroxylated product **27** (dr = 20:1, 25.9 mg, 46% yield, 78% ee) was purified by chromatography on silica gel (eluted with petroleum ether : ethyl acetate = 1:2). $[\alpha]_D^{25}$ = – 19.85 (*c* 1.30, $CHCl_3$)

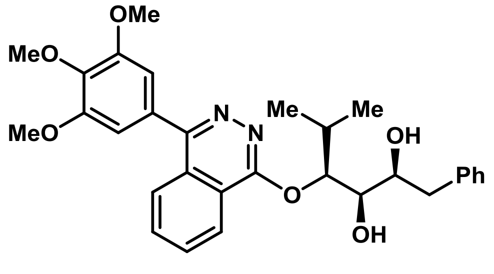

27a

(2*S*,3*R*,4*S*)-5-methyl-1-phenyl-4-(4-(3,4,5-trimethoxyphenyl)phthalazin-1-yloxy)hexane-2,3-diol

^{1}H NMR (600 MHz, $CDCl_3$): δ 8.29 (d, *J* = 6.0 Hz, 1H), 8.05 (d, *J* = 12.0 Hz, 1H), 7.94 – 7.87 (m, 2H), 7.25 – 7.20 (m, 4H), 7.19 – 7.15 (m, 1H), 5.32 (d, *J* = 6.0 Hz, 1H), 5.10 (br, 1H), 3.94 (s, 3H), 3.91 (s, 6H), 3.89 – 3.84 (m, 1H), 3.74 (d, *J* = 6.0 Hz, 1H), 3.07 – 2.99 (m, 1H), 2.95 – 2.87 (m, 1H), 2.82 (br, 1H), 2.52 – 2.43 (m, 1H), 1.17 (*J* = 6.0 Hz, 3H), 0.97 (d, *J* = 6.0 Hz, 3H) ppm.

^{13}C NMR (151 MHz, $CDCl_3$): δ 161.8, 157.1, 153.4, 139.0, 132.9, 132.2, 129.6, 128.3, 128.2, 126.5, 126.1, 123.4, 120.4, 107.2, 82.4, 71.2, 70.2, 61.0, 56.4, 39.0, 29.0, 20.3, 15.5 ppm.

HRMS (ESI) m/z: $[M + H]^+$ Calcd for $C_{30}H_{35}N_2O_6$ 519.2490; Found 519.2492.

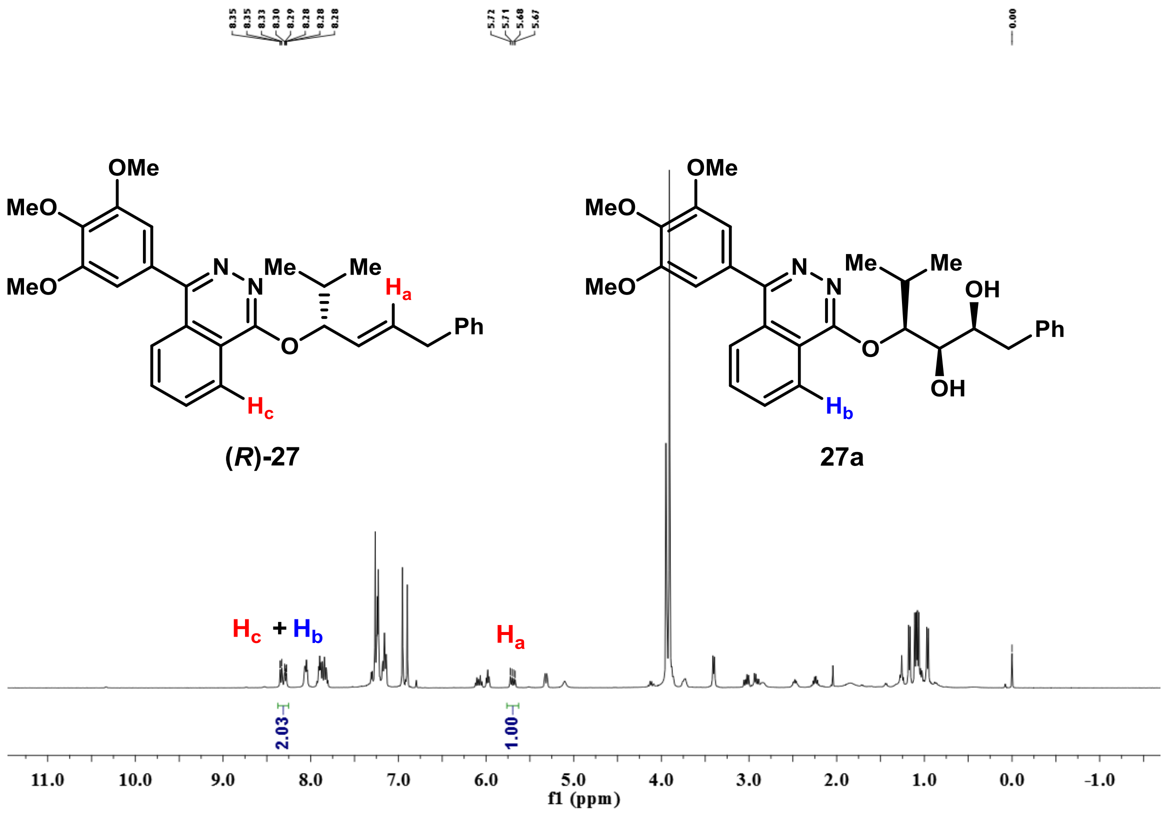

Supplementary Figure 168. ^{1}H NMR spectrum of crude mixture of compound **(*R*)-27** and **27a.**

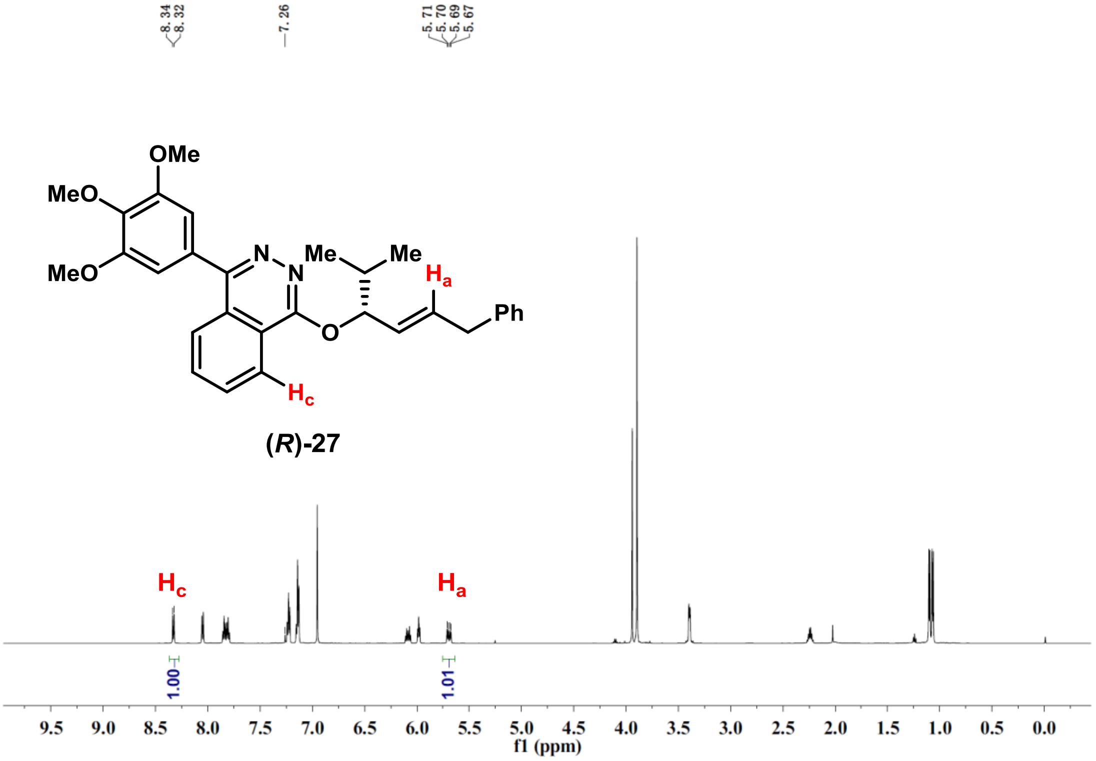

Supplementary Figure 169. ^{1}H NMR spectrum of recovered alkene **(*R*)-27**.

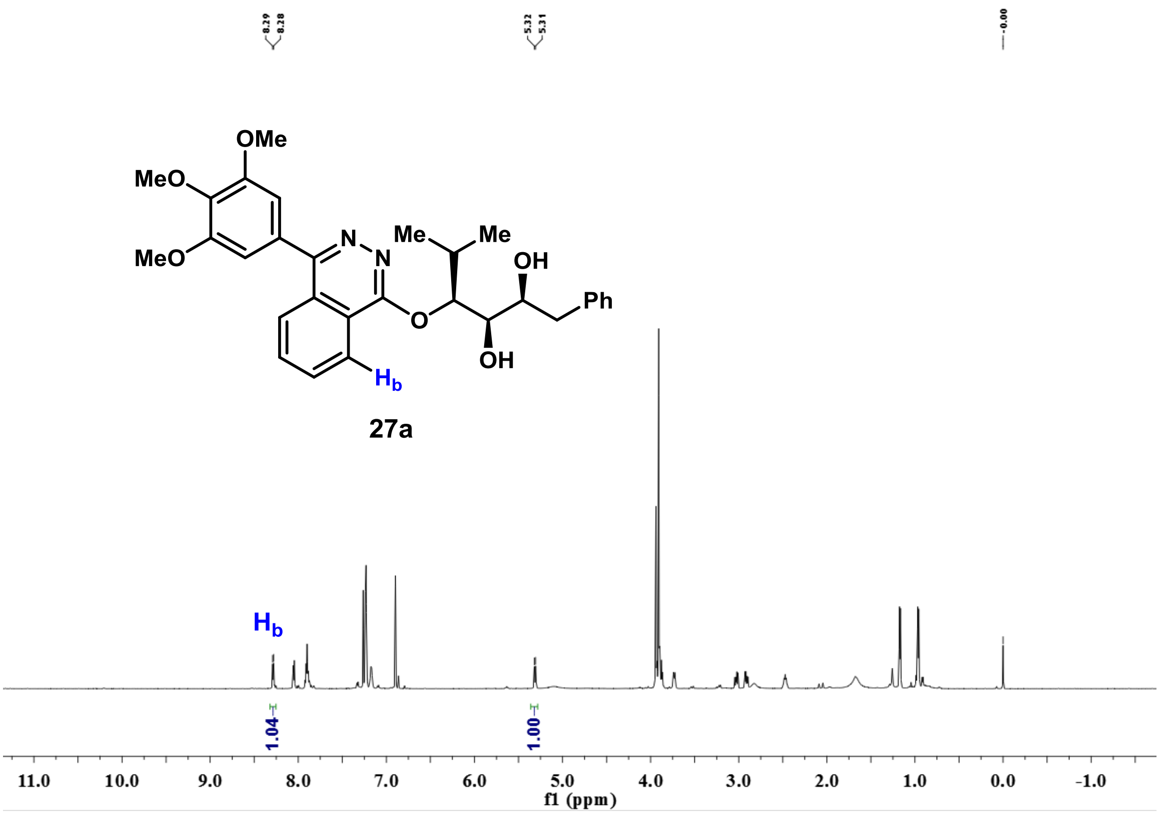

Supplementary Figure 170. ^{1}H NMR spectrum of dihydroxylated product **27a**.

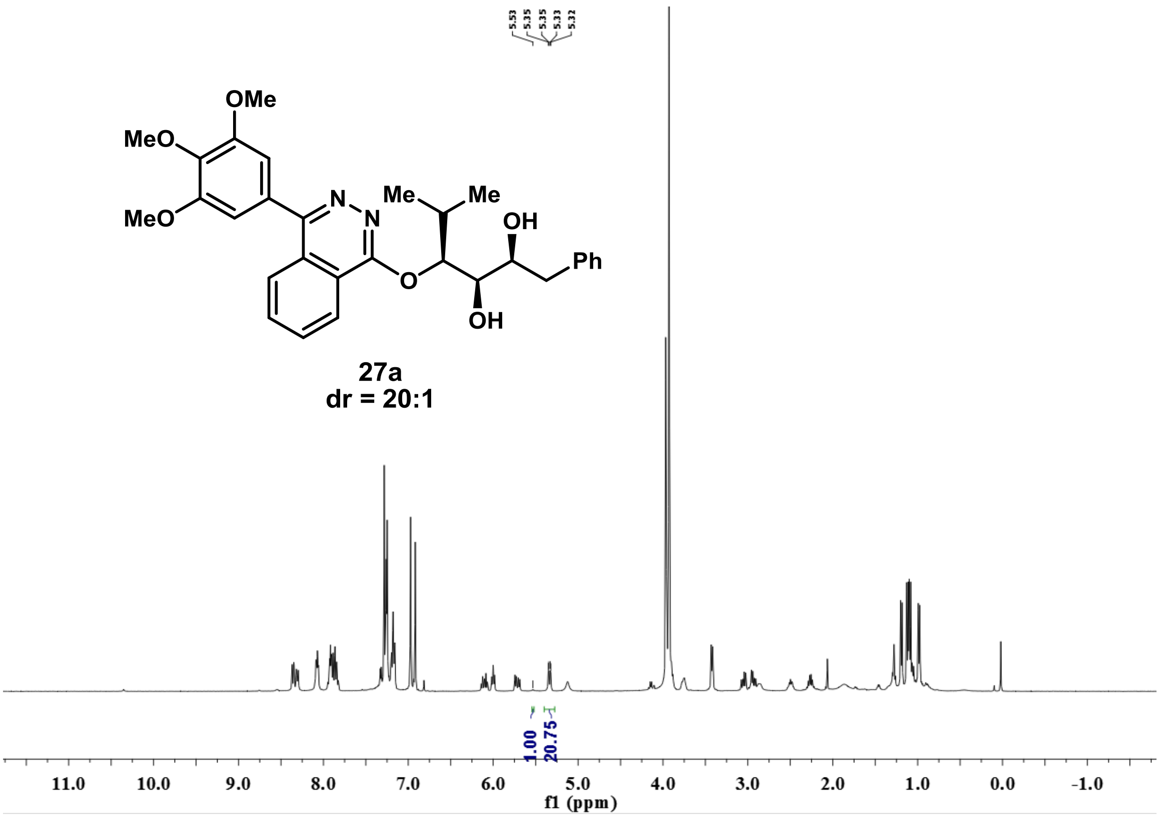

Supplementary Figure 171. ^{1}H NMR spectrum of crude mixture for diastereomeric ratio (dr).

HPLC (AD-H, 0.46*25 cm, 5μm, hexane/isopropanol = 75/25, flow = 1.0 mL/min, detection at 210 nm), retention time = 7.372 min (major) and 16.133 min (minor).

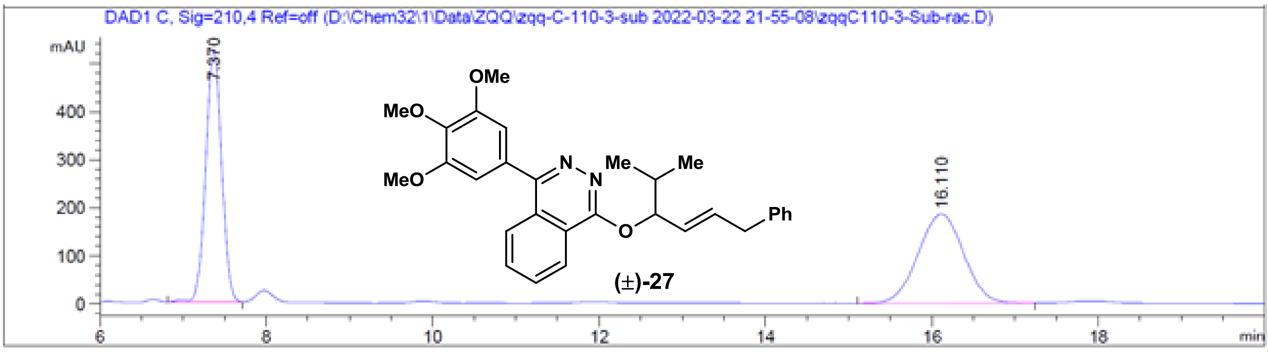

Signal 2: DAD1 C, Sig=210,4 Ref=off

Peak #	RetTime [min]	Type	Width [min]	Area [mAU*s]	Height [mAU]	Area %
1	7.370	VV R	0.2072	7046.64697	525.80658	50.1202
2	16.110	BB	0.5941	7012.85254	185.05539	49.8798

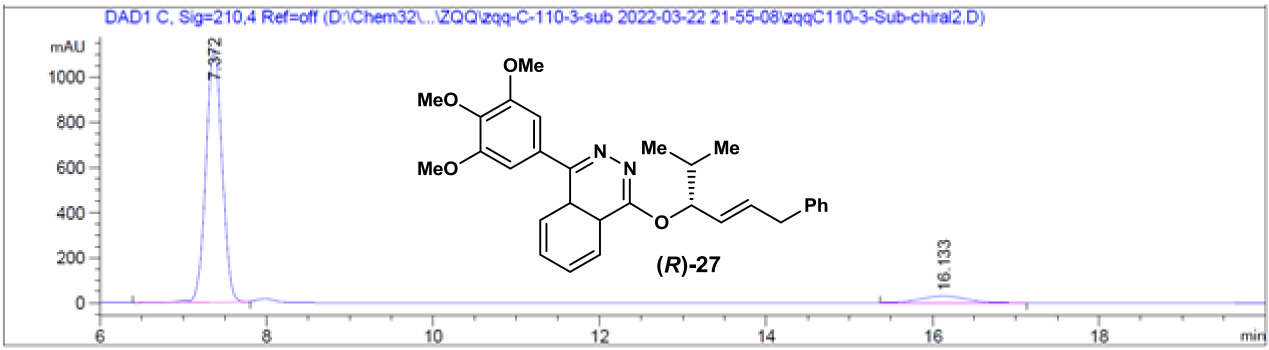

Signal 2: DAD1 C, Sig=210,4 Ref=off

Peak #	RetTime [min]	Type	Width [min]	Area [mAU*s]	Height [mAU]	Area %
1	7.372	MM R	0.2255	1.51415e4	1119.01318	92.1135
2	16.133	MM R	0.6950	1296.38049	31.08863	7.8865

Supplementary Figure 172. HPLC chromatogram for ***(R)*-27**.

HPLC (AD-H, 0.46*25 cm, 5µm, hexane/isopropanol = 75/25, flow = 1.0 mL/min, detection at 210 nm), retention time = 11.589 min (minor) and 31.670 min (major).

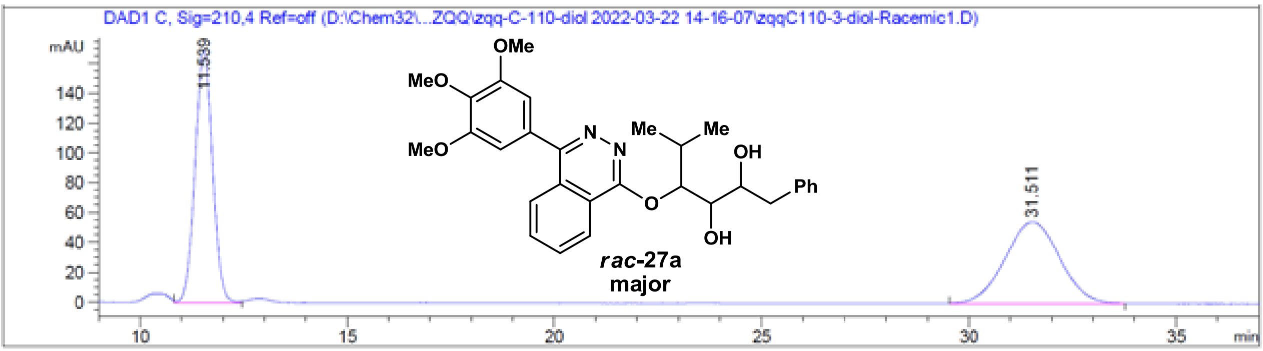

Signal 2: DAD1 C, Sig=210,4 Ref=off

Peak #	RetTime [min]	Type	Width [min]	Area [mAU*s]	Height [mAU]	Area %
1	11.539	MM R	0.4932	5008.00098	169.22406	50.1675
2	31.511	BB	1.0844	4974.56445	54.32043	49.8325

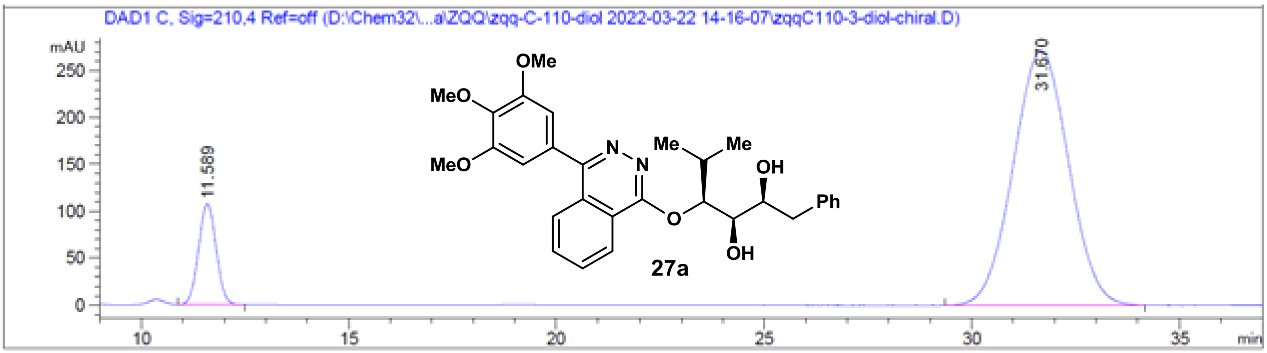

Signal 2: DAD1 C, Sig=210,4 Ref=off

Peak #	RetTime [min]	Type	Width [min]	Area [mAU*s]	Height [mAU]	Area %
1	11.589	BB	0.4547	3182.49463	107.70312	11.2493
2	31.670	BB	1.3192	2.51080e4	271.41843	88.7507

Supplementary Figure 173. HPLC chromatogram for **27a**.

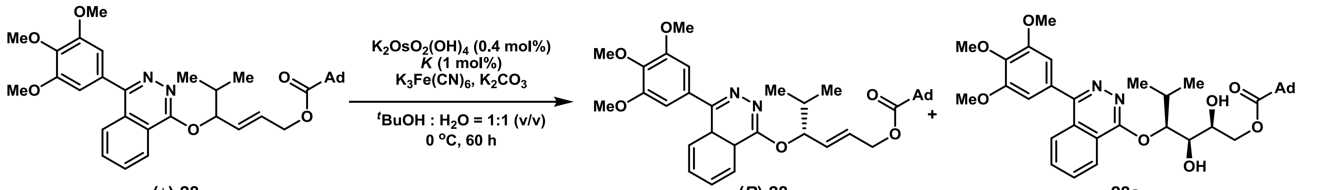

(±)-28 — (*R*)-28: *s* = 31, conv = 58%, ee = 99%, 40% yield — 28a: dr = 20:1, 75% ee, 53% yield

The general procedure **IV** was followed. The conversion of **(±)-28** was determined by crude ^{1}H NMR. **Conversion** (%) = [(2.40 – 1.00) / 2.40] % = 58%.

S = In [(1 – conv)(1 – ee)] / In [(1 – conv)(1 + ee)] = 31.

The recovered alkene **(*R*)-28** (23.4 mg, 40% yield, 99% ee) was purified by chromatography on silica gel (eluted with petroleum ether : ethyl acetate = 3:1). $[\alpha]_D^{25}$ = + 14.20(*c* 0.70, $CHCl_3$).

The dihydroxylated product **28a** (dr = 20:1, 32.8 mg, 53% yield, 75% ee) was purified by chromatography on silica gel (eluted with petroleum ether : ethyl acetate = 1:2). $[\alpha]_D^{25}$ = – 20.73 (*c* 0.55, $CHCl_3$)

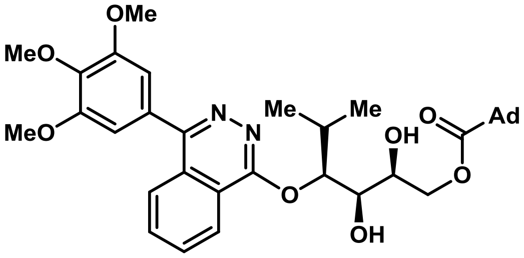

28a

(2*S*,3*R*,4*S*)-2,3-dihydroxy-5-methyl-4-(4-(3,4,5-trimethoxyphenyl)phthalazin-1-yloxy)hexyl adamantanecarboxylate

^{1}H NMR (600 MHz, $CDCl_3$): δ 8.38 – 8.31 (m, 1H), 8.10 – 8.04 (m, 1H), 8.01 – 7.87 (m, 2H), 6.91 (s, 2H), 5.34 – 5.28 (m, 1H), 5.11 (br, 1H), 4.31 – 4.19 (m, 2H), 3.95 (s, 3H), 3.92 (s, 6H), 2.99 (br, 1H), 2.56 – 2.45 (m, 1H), 2.02 – 1.96 (m, 3H), 1.90 – 1.82 (m, 6H), 1.75 – 1.60 (m, 9H), 1.25 (d, *J* = 6.0 Hz, 3H), 1.03 (d, *J* = 6.0 Hz, 3H) ppm.

^{13}C NMR (151 MHz, $CDCl_3$): δ 177.6, 161.8, 157.3, 153.4, 139.0, 133.0, 132.3, 131.3, 128.4, 126.6, 123.4, 120.5, 107.2, 82.1, 69.8, 67.0, 63.8, 61.0, 56.3, 40.7, 38.8, 36.5, 29.0, 27.9, 20.3, 15.5 ppm.

HRMS (ESI) m/z: $[M + H]^+$ Calcd for $C_{35}H_{45}N_2O_8$ 621.3170; Found 621.3176.

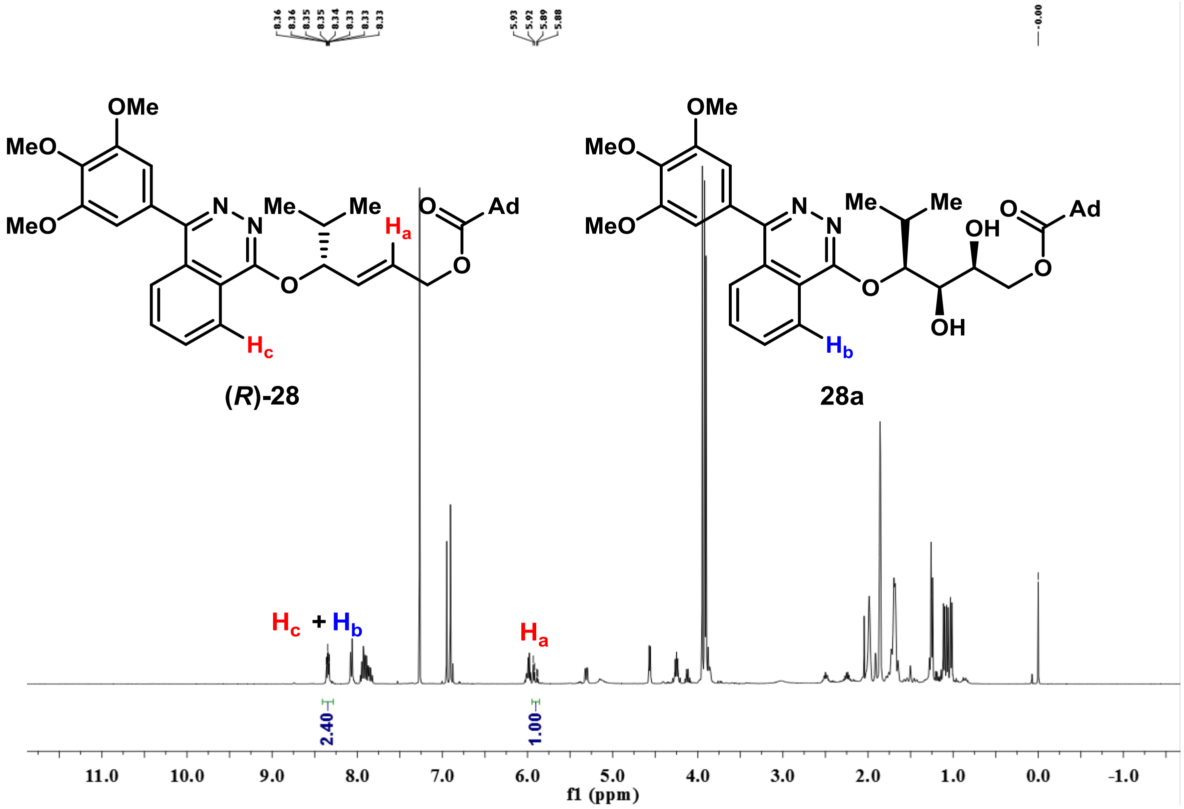

Supplementary Figure 174. ^{1}H NMR spectrum of crude mixture of compound **(*R*)-28** and **28a.**

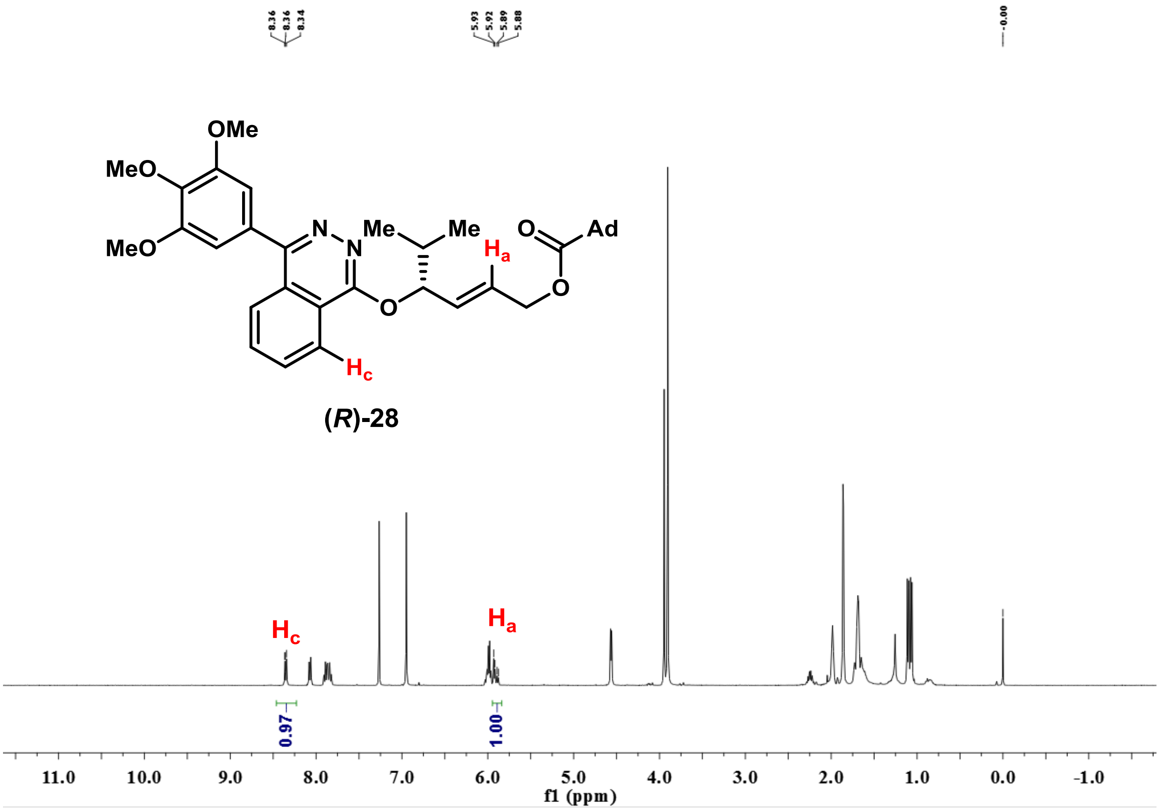

Supplementary Figure 175. ^{1}H NMR spectrum of recovered alkene **(*R*)-28**.

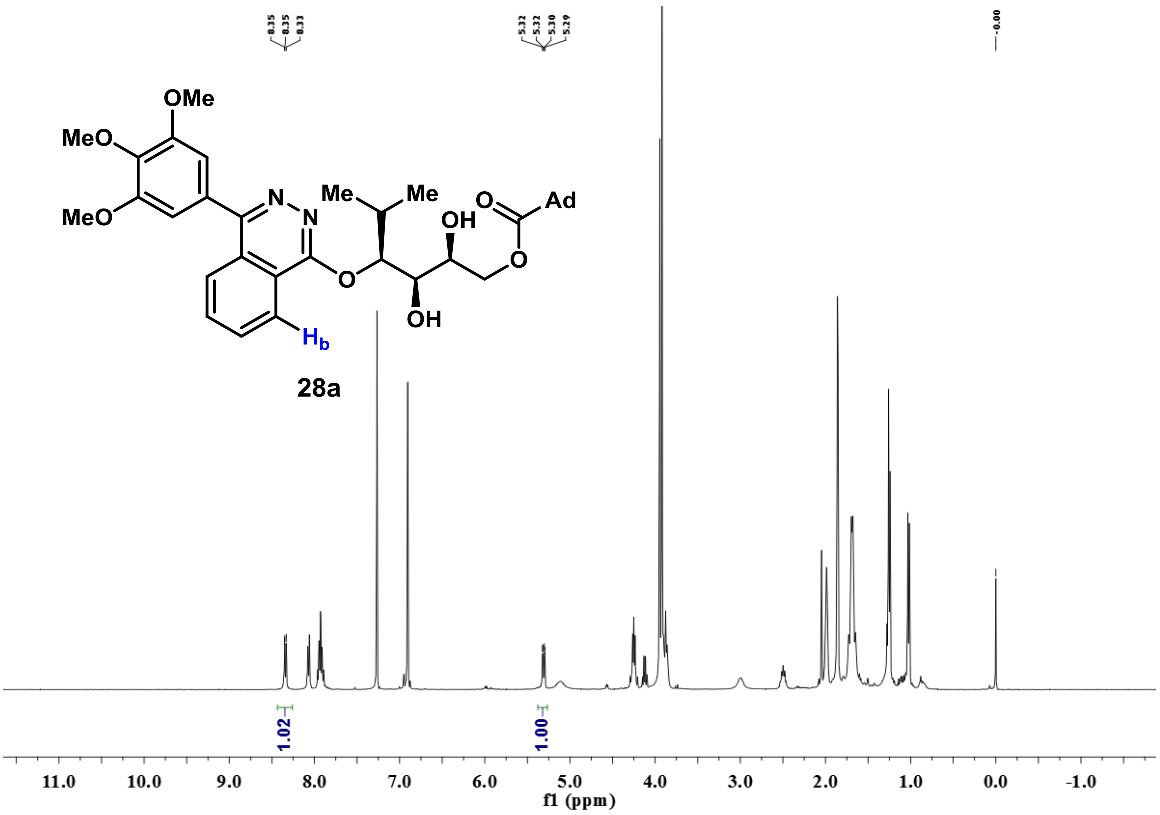

Supplementary Figure 176. ^{1}H NMR spectrum of dihydroxylated product **28a**.

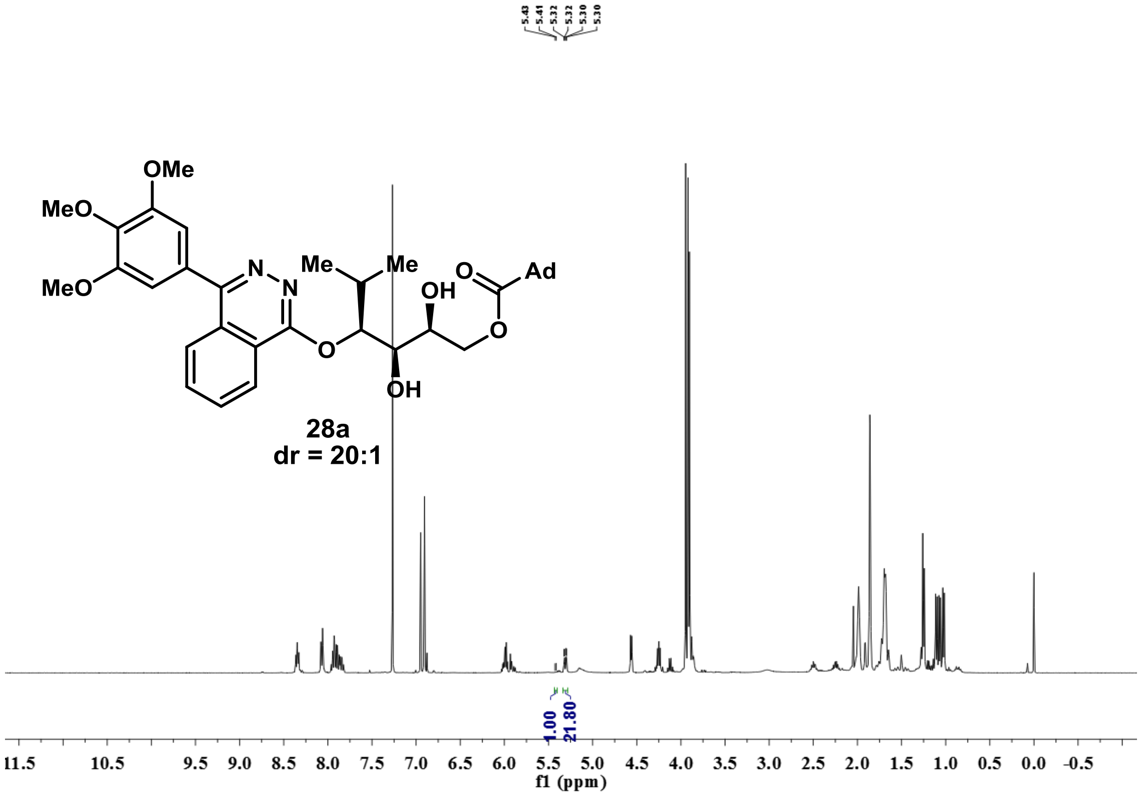

Supplementary Figure 177. ^{1}H NMR spectrum of crude mixture for diastereomeric ratio (dr).

HPLC (OD-H, 0.46*25 cm, 5μm, hexane/isopropanol = 95/5, flow = 1.0 mL/min, detection at 210 nm), retention time = 26.636 min (minor) and 28.861 min (major).

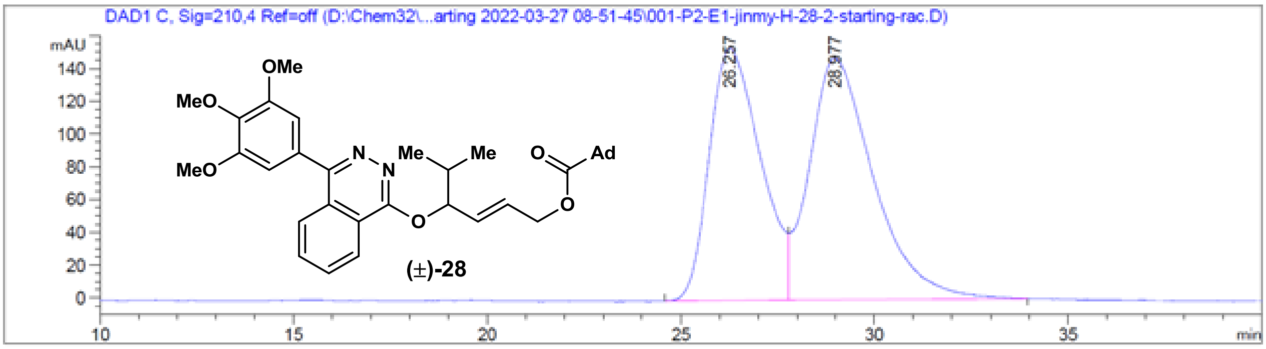

Signal 2: DAD1 C, Sig=210,4 Ref=off

Peak #	RetTime [min]	Type	Width [min]	Area [mAU*s]	Height [mAU]	Area %
1	26.257	BV	1.2398	1.41629e4	153.87209	45.5161
2	28.977	VB	1.5329	1.69533e4	147.28668	54.4839

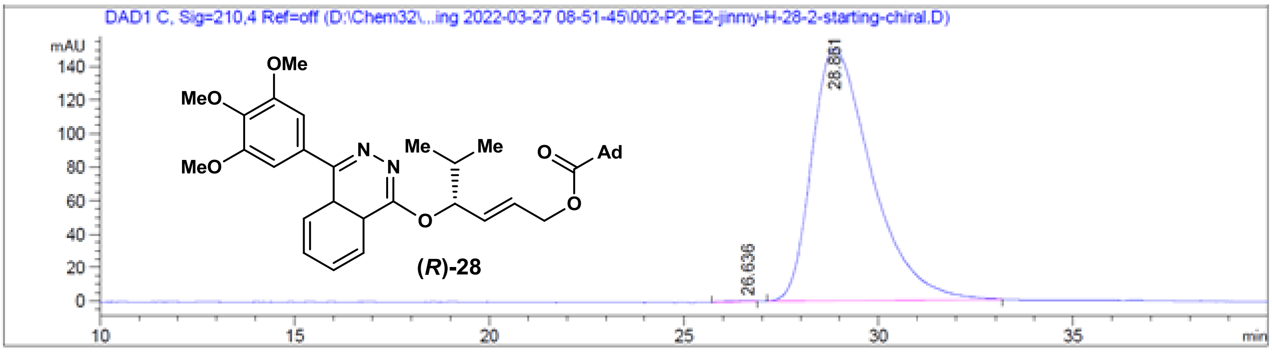

Signal 2: DAD1 C, Sig=210,4 Ref=off

Peak #	RetTime [min]	Type	Width [min]	Area [mAU*s]	Height [mAU]	Area %
1	26.636	MM R	0.6086	16.71570	4.57740e-1	0.1043
2	28.861	BB R	1.3512	1.60041e4	150.19429	99.8957

Supplementary Figure 178. HPLC chromatogram for ***(R)*-28**.

HPLC (AD-H, 0.46*25 cm, 5μm, hexane/isopropanol = 90/10, flow = 1.0 mL/min, detection at 210 nm), retention time = 13.980 min (major) and 21.100 min (minor).

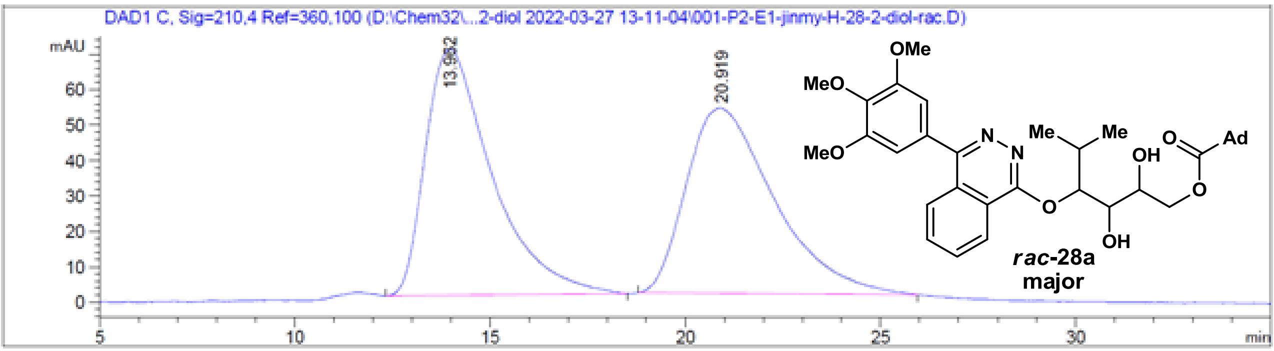

Signal 2: DAD1 C, Sig=210,4 Ref=360,100

Peak #	RetTime [min]	Type	Width [min]	Area [mAU*s]	Height [mAU]	Area %
1	13.962	BB	1.4090	8275.05957	69.50427	50.1921
2	20.919	BB	1.8518	8211.71191	52.41449	49.8079

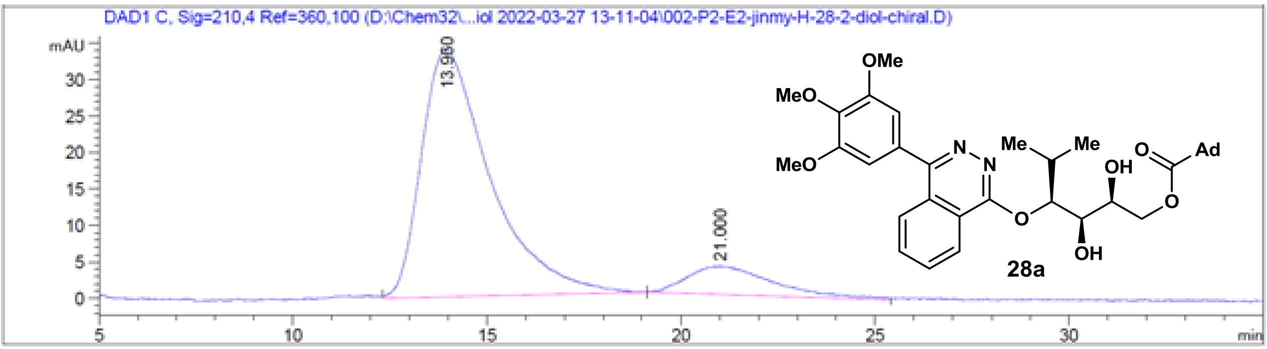

Signal 2: DAD1 C, Sig=210,4 Ref=360,100

Peak #	RetTime [min]	Type	Width [min]	Area [mAU*s]	Height [mAU]	Area %
1	13.960	MM R	2.0494	4181.19922	34.00411	87.3509
2	21.000	MM R	2.5931	605.47107	3.89150	12.6491

Supplementary Figure 179. HPLC chromatogram for **28a**.

2.7 Unsuccessful substrates

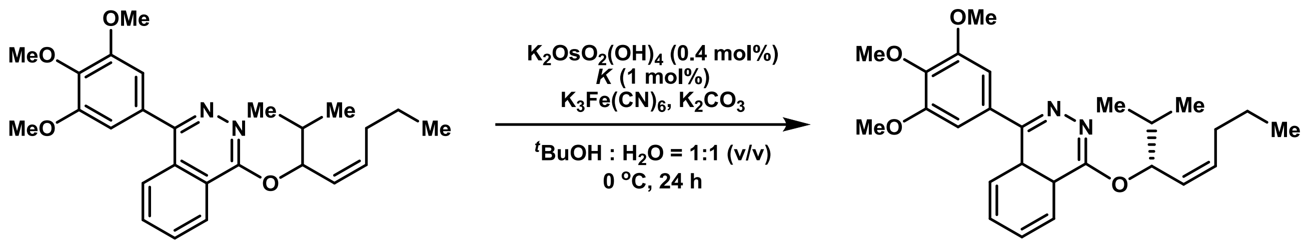

(±)-29

(*R*)-29
no conversion

The general procedure **IV** was followed. The conversion of **(±)-29** was determined by crude ^{1}H NMR (no conversion).

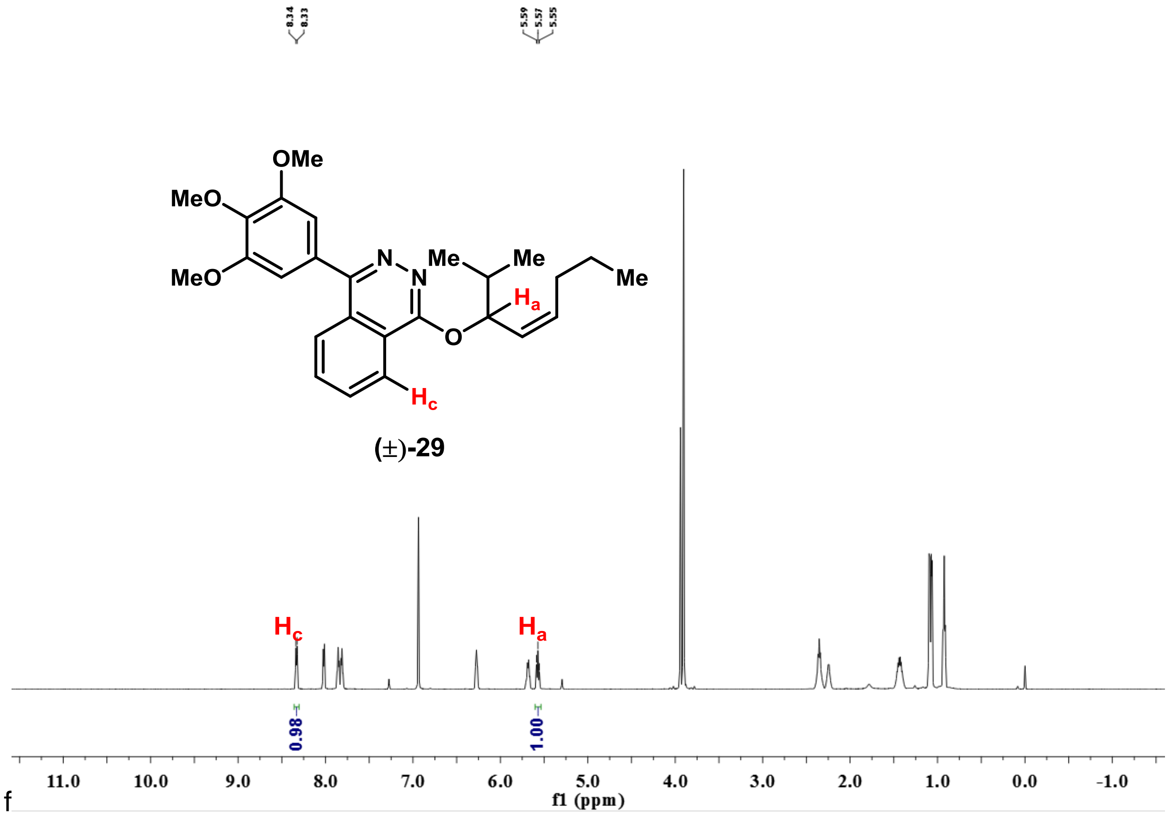

Supplementary Figure 180. ^{1}H NMR spectrum of recovered alkene **(±)-29**.

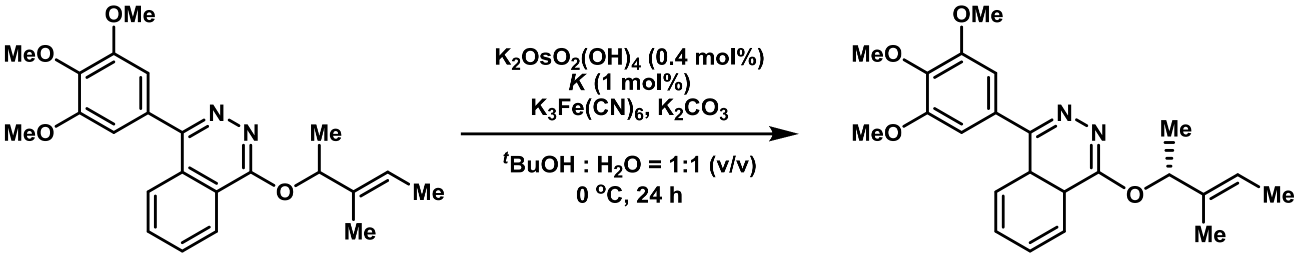

The general procedure **IV** was followed. The conversion of **(±)-30** was determined by crude ^{1}H NMR.

Conversion (%) = [(2.14 – 1.00) / 2.14] % = 53%

S = In [(1 – conv)(1 – ee)] / In [(1 – conv)(1 + ee)] = 3 .

The recovered alkene **(*R*)-30** (17.8 mg, 45% yield, 20% ee) was purified by chromatography on silica gel (eluted with petroleum ether : ethyl acetate = 3:1). $[\alpha]_D^{25}$ = – 0.77 (*c* 0.65, $CHCl_3$).

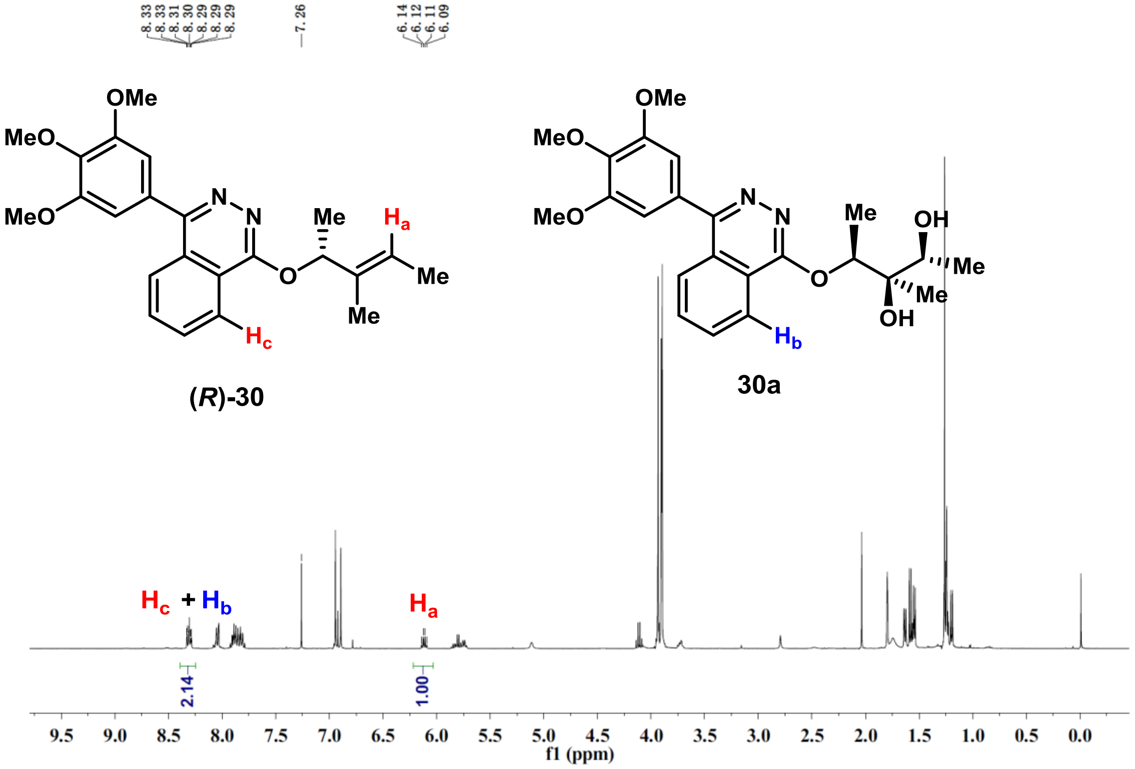

Supplementary Figure 181. ^{1}H NMR spectrum of crude mixture of compound **(*R*)-30** and **30a.**

HPLC (OD-H, 0.46*25 cm, 5μm, hexane/isopropanol = 80/20, flow = 1.0 mL/min, detection at 210, nm) retention time = 6.604 min (major) and 8.043 min (minor).

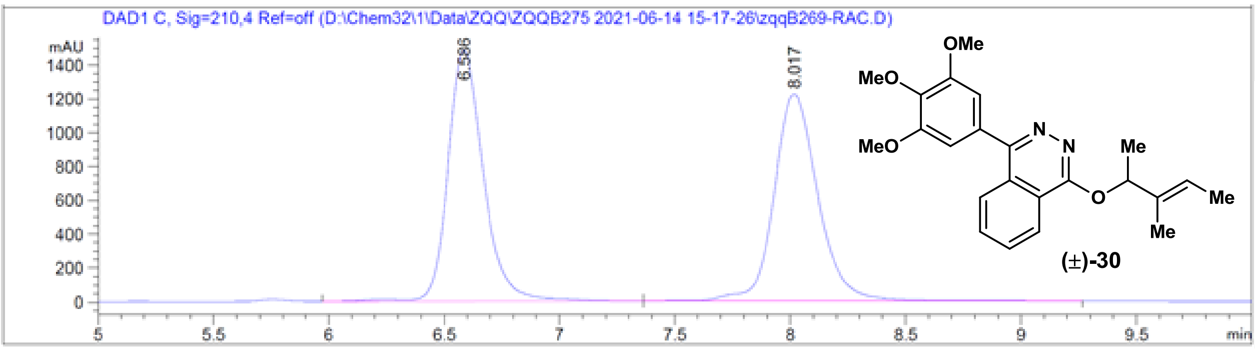

Signal 2: DAD1 C, Sig=210,4 Ref=off

Peak #	RetTime [min]	Type	Width [min]	Area [mAU*s]	Height [mAU]	Area %
1	6.586	VB R	0.1619	1.59260e4	1483.60034	49.4701
2	8.017	VB R	0.2030	1.62672e4	1222.48291	50.5299

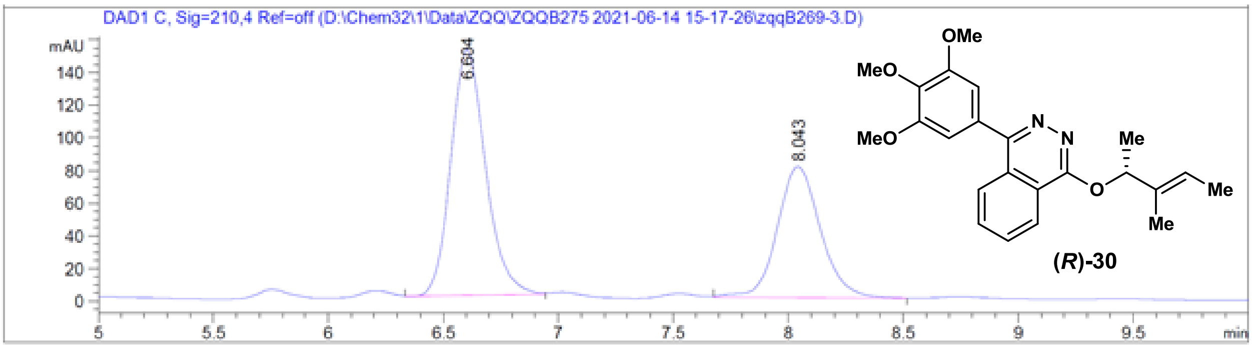

Signal 2: DAD1 C, Sig=210,4 Ref=off

Peak #	RetTime [min]	Type	Width [min]	Area [mAU*s]	Height [mAU]	Area %
1	6.604	MM R	0.1743	1577.05530	150.76903	60.0135
2	8.043	MM R	0.2180	1050.77966	80.32583	39.9865

Supplementary Figure 182. HPLC chromatogram for ***(R)*-30**.

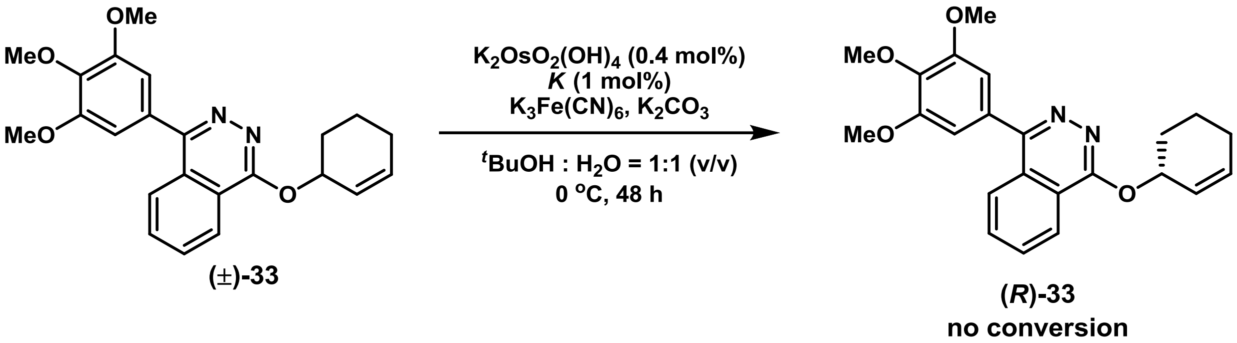

The general procedure **IV** was followed. The conversion of **(±)-33** was determined by crude ^{1}H NMR (no conversion).

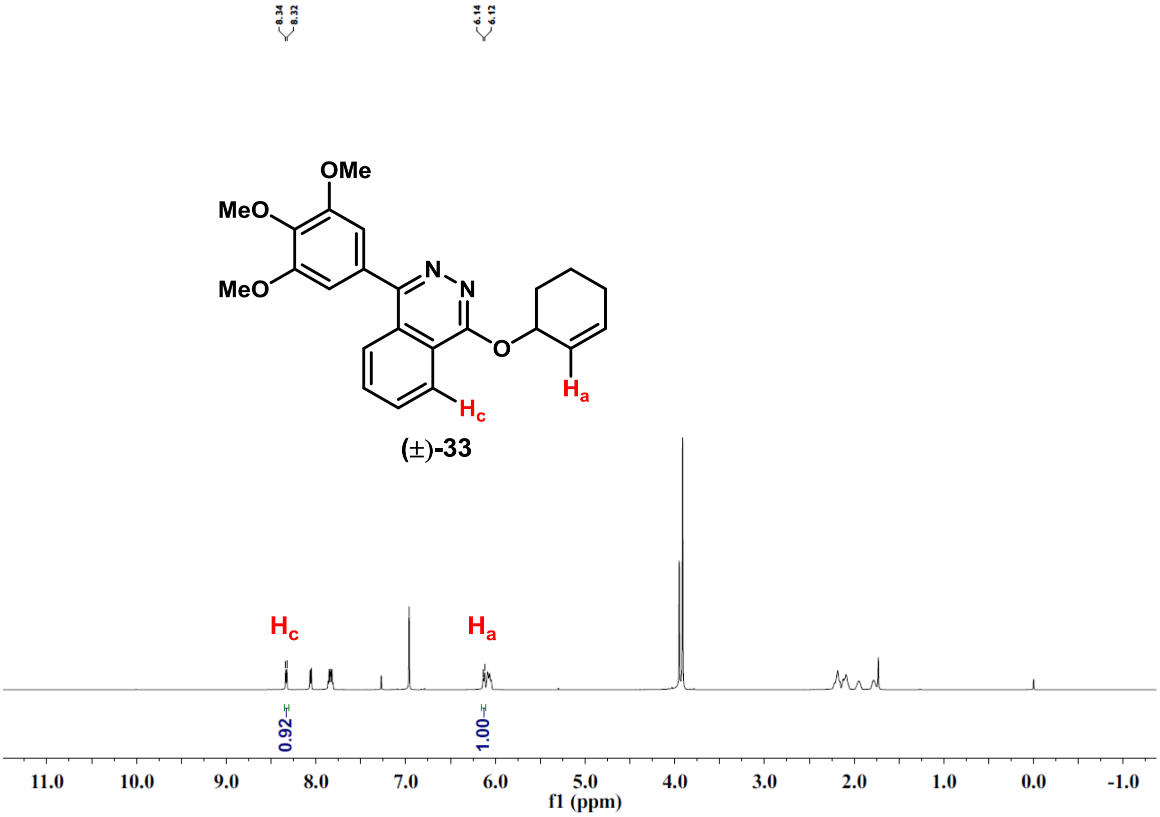

Supplementary Figure 183. ^{1}H NMR spectrum of recovered alkene **(*R*)-33**.

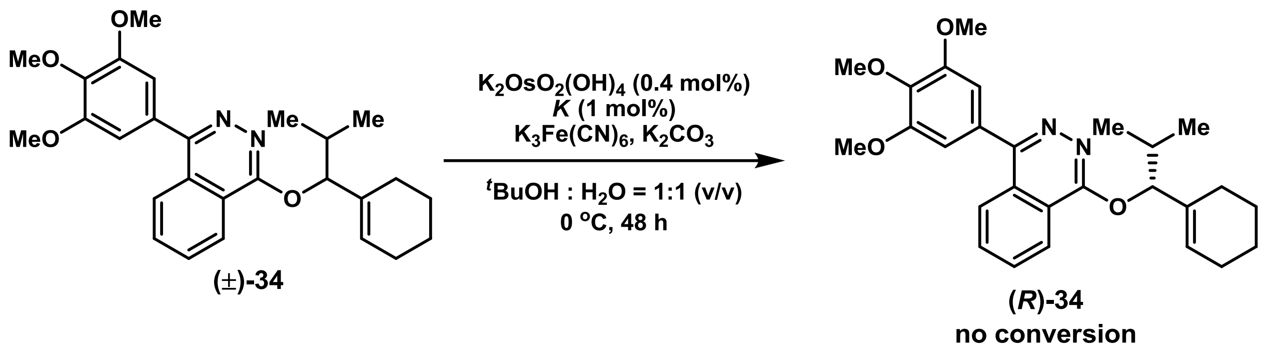

The general procedure **IV** was followed. The conversion of **(±)-34** was determined by crude ^{1}H NMR (no conversion).

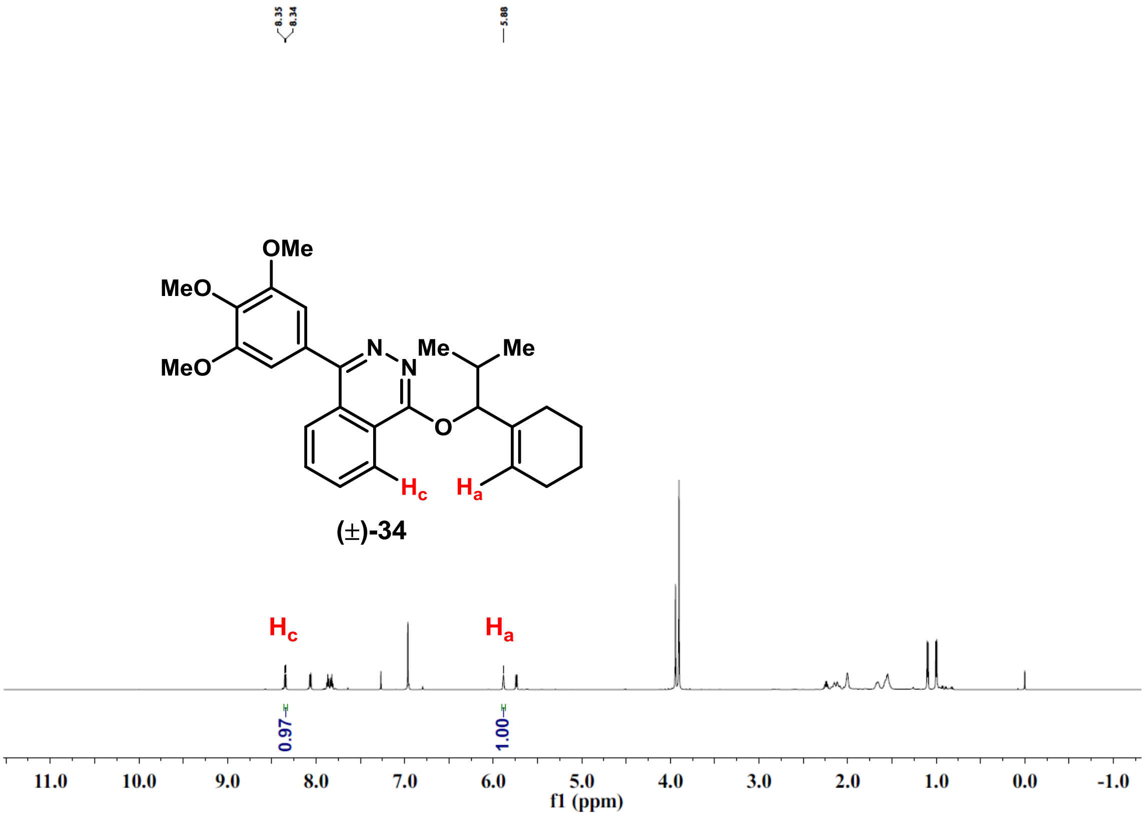

Supplementary Figure 184. ^{1}H NMR spectrum of recovered alkene **(*R*)-34**.

2.8 Synthetic Application

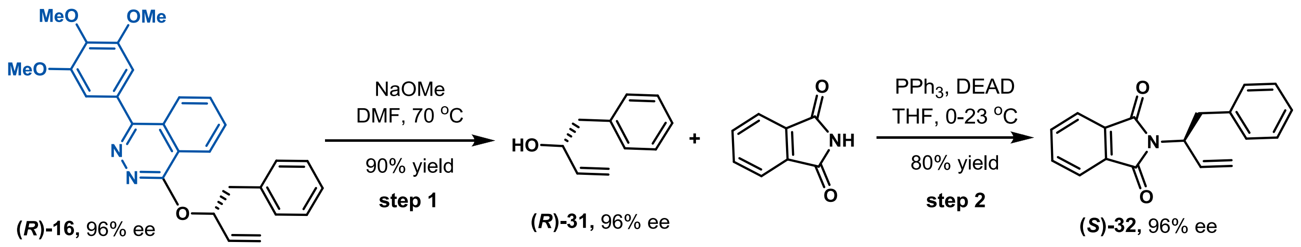

Step 1: To a round bottle flask, **(*R*)-16** (22.0 mg, 0.05 mmol) in DMF (1.0 mL) was added NaOMe (0.15 mmol, 3.0 equiv.) under air atmosphere. The mixture was stirred at 70 °C for 12 h. After the reaction was completed, the mixture was diluted with water and extracted with EtOAc. The organic phase was washed with brine, dried over Na_2SO_4, filtered, and concentrated under vacuum. The crude residue was purified by flash column chromatography on silica gel to afford product **(*R*)-31** (6.7 mg, 90% yield, 96% ee). (**Ref:** *Angew. Chem. Int. Ed.* **2009**, *48*, 3155 –3157.)

Step 2: To a two-necked 10 mL flask were added **(*R*)-31** (6.7 mg, 0.05 mmol), phthalimide (11.0 mg, 0.075 mmol, 1.5 equiv), PPh_3 (19.7 mg, 0.075, 1.5 equiv) and 2.0 mL anhydrous THF under argon atmosphere, then DEAD (13.1 mg, 0.075 mmol, 1.5 equiv) in 1.0 mL THF was added dropwise to the solution mixture at 0 °C. Then the reaction was stirred at room temperature for 5 h. The reaction mixture was concentrated under vacuum and further purified by flash column chromatography (Petroleum ether: ethyl acetate=20:1) to afford the product **(*S*)-32** (11.0 mg, 80% yield, 96% ee).

Analytic Data of Compounds:

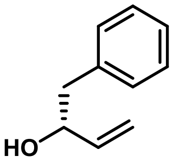

(*R*)-31

^{1}H NMR (400 MHz, $CDCl_3$): δ 7.34 – 7.31 (m, 2H), 7.26 – 7.23 (m, 3H), 5.96 -5.91 (m, 1H), 5.26 – 5.23 (m, 1H), 5.14 – 5.12 (m, 1H), 4.36 – 4.35 (m, 1H), 2.89 – 2.86 (m, 1H), 2.82 – 2.79 (m, 1H) ppm.

^{13}C NMR (101 MHz, $CDCl_3$): δ 140.1, 137.8, 129.5, 128.4, 126.5, 114.9, 73.6, 43.8 ppm.

$[\alpha]_D^{25}$ = + 12.25 (c 0.35, $CHCl_3$).

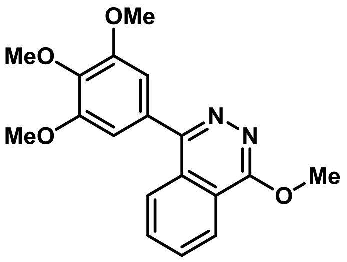

31'

^{1}H NMR (400 MHz, CDCl$_3$): δ 8.30 – 8.29 (m, 1H), 8.07 – 8.05 (m, 1H), 7.88 – 7.83 (m, 2H), 6.94 (s, 2H), 4.32 (s, 3H), 3.94 (s, 3H), 3.90 (s, 6H) ppm.

^{13}C NMR (101 MHz, CDCl$_3$): δ 160.1, 156.6, 153.3, 138.8, 132.1, 132.0, 131.7, 127.7, 126.1, 123.4, 120.3, 107.3, 61.0, 56.3, 55.0 ppm.

HRMS (ESI) m/z: $[M + H]^+$ Calcd for $C_{18}H_{19}N_2O_4$ 327.1339; Found 327.1348.

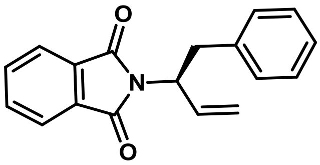

(*S*)-32

^{1}H NMR (400 MHz, CDCl$_3$): δ 7.77 – 7.74 (m, 1H), 7.69 – 7.63 (m, 1H), 7.24 – 7.08 (m, 5H), 6.28 (ddd, *J* = 17.4, 10.3, 7.2 Hz, 1H), 5.35 – 5.11 (m, 2H), 5.05 (dd, *J* = 16.7, 6.9 Hz, 1H), 3.43 (dd, *J* = 13.8, 9.9 Hz, 1H), 3.22 (dd, *J* = 13.8, 6.5 Hz, 1H) ppm.

^{13}C NMR (101 MHz, CDCl$_3$): δ168.0, 137.6, 135.2, 133.9, 131.8, 129.1, 128.5, 126.7, 123.2, 117.7, 55.1, 38.3 ppm.

HRMS (ESI) m/z: $[M + H]^+$ Calcd for $C_{18}H_{16}NO_2$ 278.1176; Found 278.1171.

HPLC (OD-H, 0.46*25 cm, 5μm, hexane/isopropanol = 90/10, flow = 1.0 mL/min, detection at 210 nm), retention time = 5.245 min (major) and 5.955 min (minor).

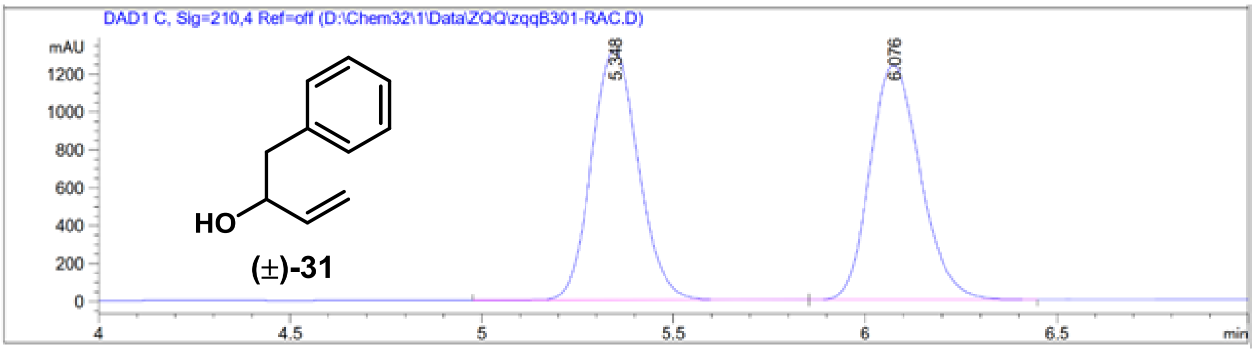

Signal 2: DAD1 C, Sig=210,4 Ref=off

Peak #	RetTime [min]	Type	Width [min]	Area [mAU*s]	Height [mAU]	Area %
1	5.348	BV R	0.1332	1.12495e4	1321.59631	49.8254
2	6.076	VB	0.1447	1.13283e4	1238.77002	50.1746

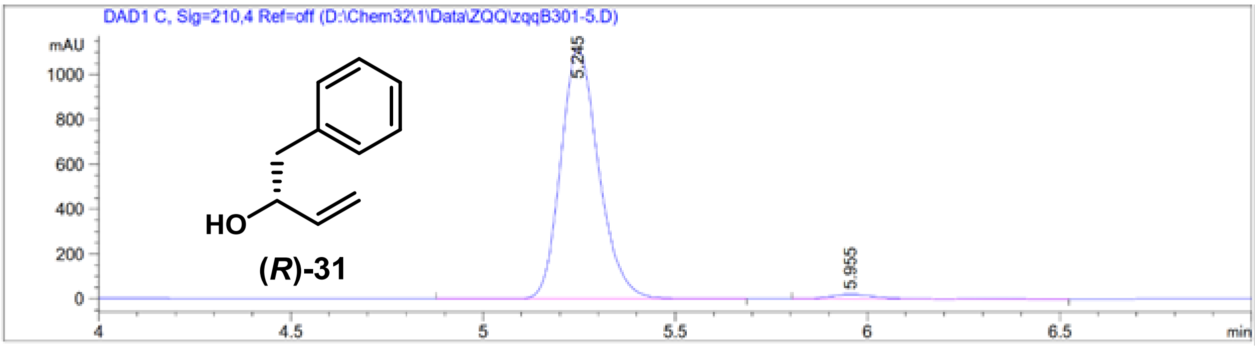

Signal 2: DAD1 C, Sig=210,4 Ref=off

Peak #	RetTime [min]	Type	Width [min]	Area [mAU*s]	Height [mAU]	Area %
1	5.245	BB	0.1061	7675.17139	1117.66333	97.8840
2	5.955	BV R	0.1174	165.91653	20.31760	2.1160

Supplementary Figure 185. HPLC chromatogram for **(*R*)-31**.

HPLC (OD-H, 0.46*25 cm, 5µm, hexane/ethanol = 90/10, flow = 1.0 mL/min, detection at 254 nm), retention time = 11.426 min (major) and 12.987 min (minor).

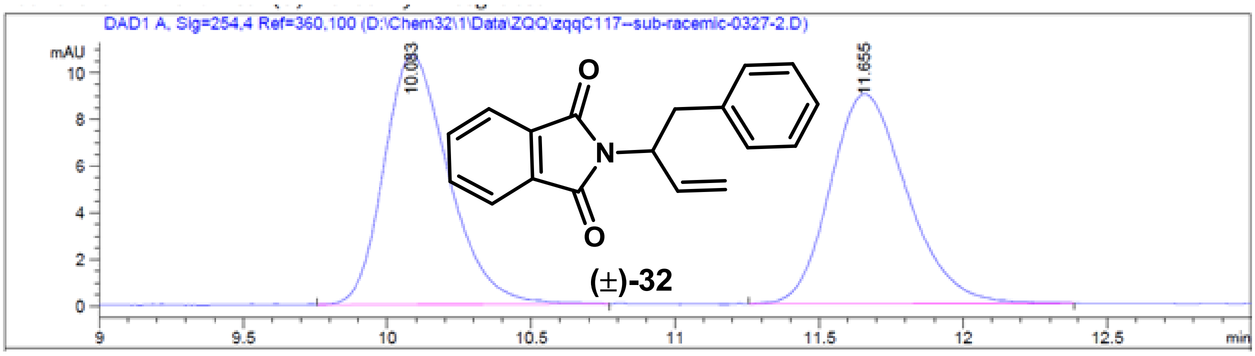

Signal 1: DAD1 A, Sig=254,4 Ref=360,100

Peak #	RetTime [min]	Type	Width [min]	Area [mAU*s]	Height [mAU]	Area %
1	10.083	BB	0.2399	166.32690	10.66947	49.9062
2	11.655	BB	0.2866	166.95184	8.99287	50.0938

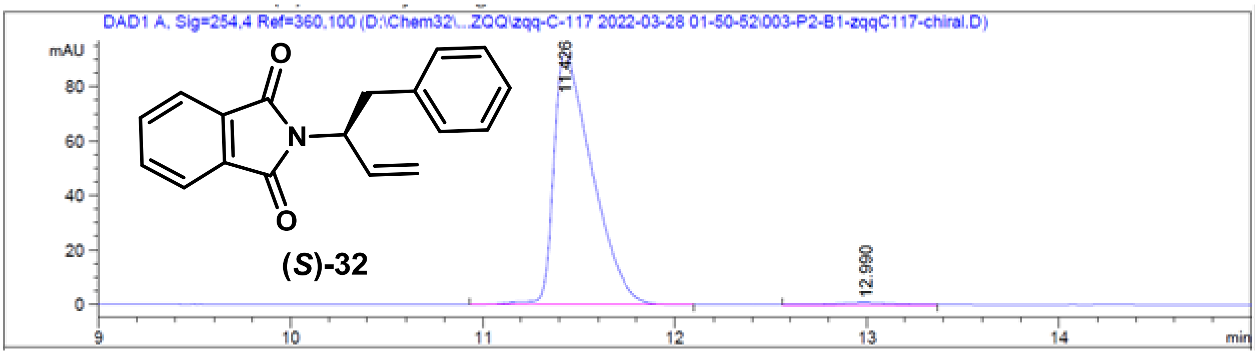

Signal 1: DAD1 A, Sig=254,4 Ref=360,100

Peak #	RetTime [min]	Type	Width [min]	Area [mAU*s]	Height [mAU]	Area %
1	11.426	BB	0.2052	1274.72742	92.26303	98.2406
2	12.990	MM R	0.4041	22.82932	9.41652e-1	1.7594

Supplementary Figure 186. HPLC chromatogram for **(*S*)-32**.

3 Supplementary Discussion

3.1 Determination of absolute configuration of (±)-12

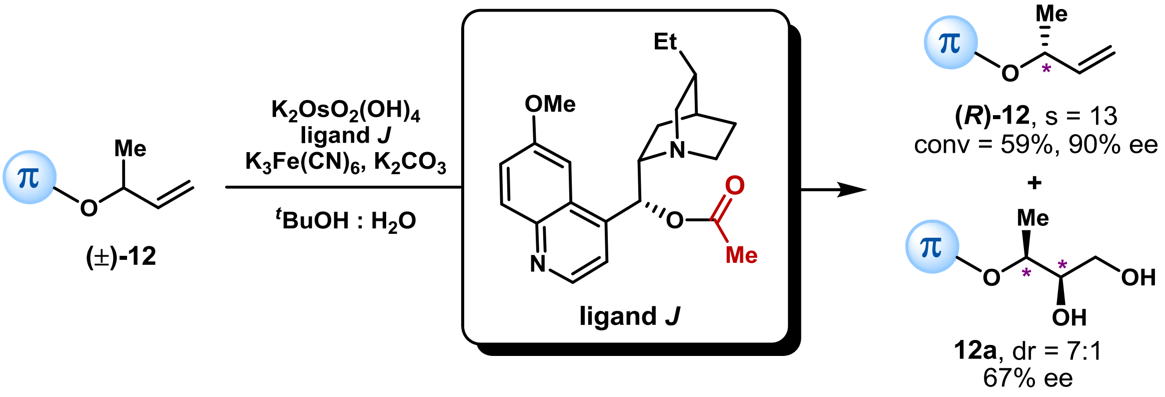

The conversion of **(±)-12** was determined by crude ^{1}H NMR. **Conversion** (%) = [(2.43 – 1.00)/ 2.43] = 59%.

S = In [(1–conv)(1–ee)] / In [(1–conv)(1+ee)] = 13

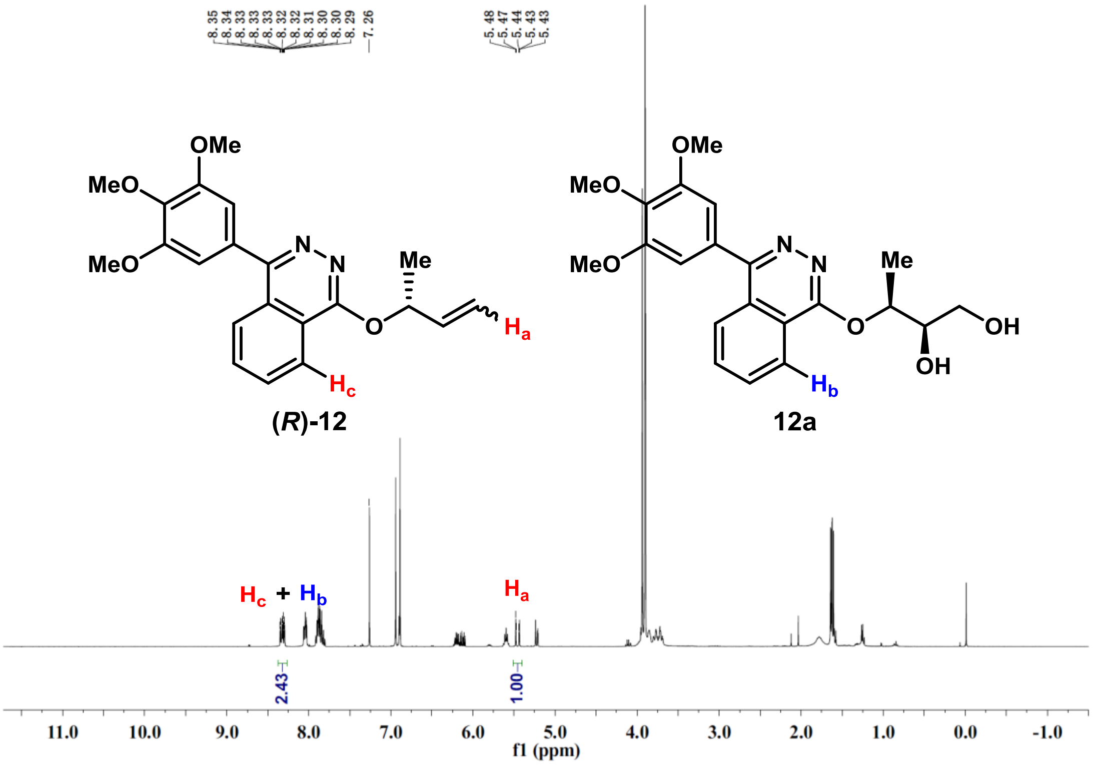

Supplementary Figure 187. ^{1}H NMR spectrum of crude mixture of compound **(*R*)-12** and **12a.**

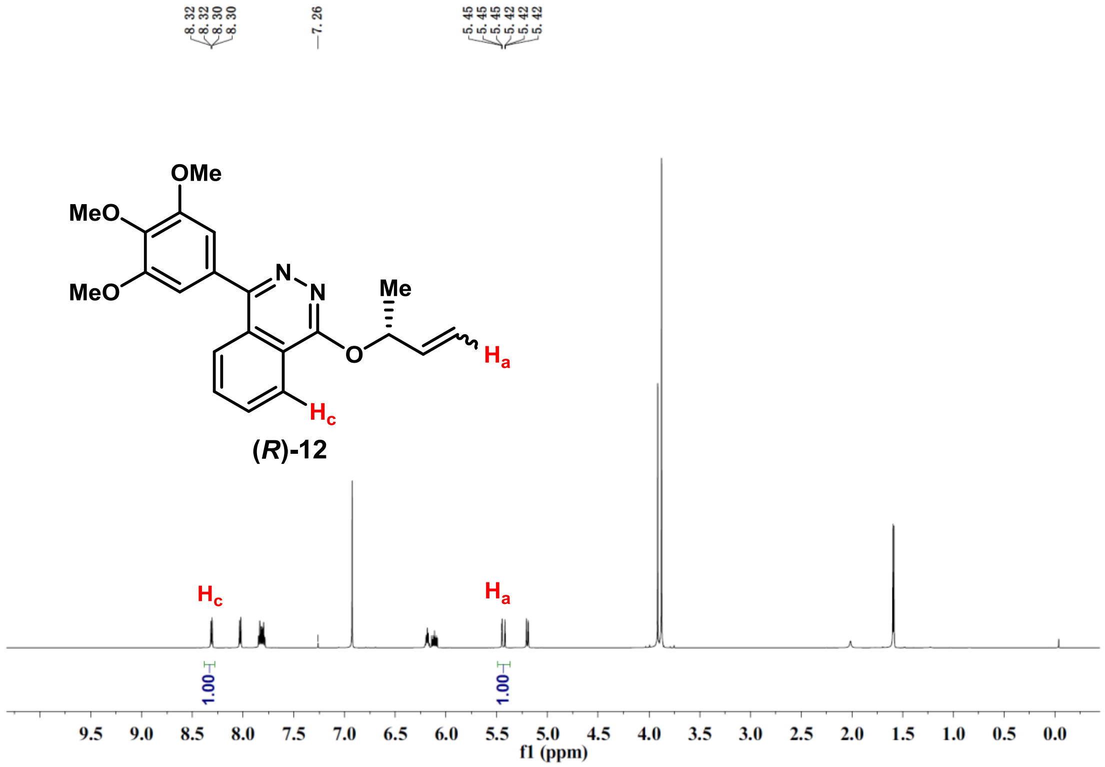

Supplementary Figure 188. ^{1}H NMR spectrum of recovered alkene **(*R*)-12**.

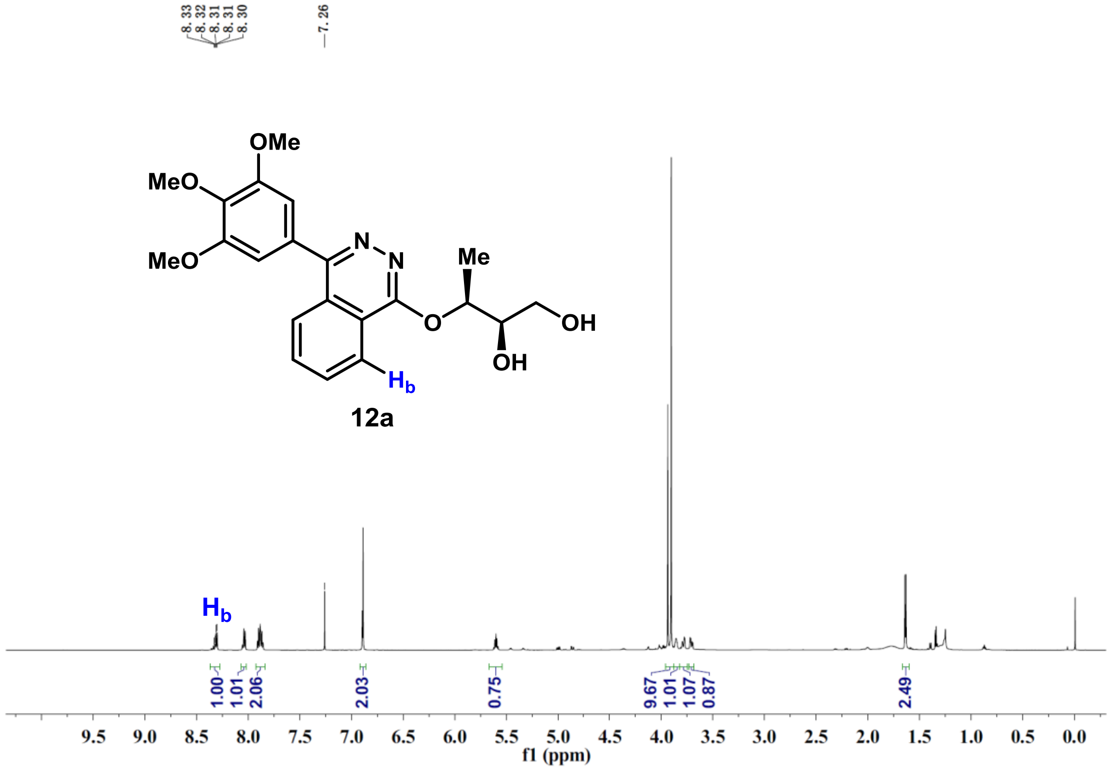

Supplementary Figure 189. ^{1}H NMR spectrum of dihydroxylated product **12a**.

HPLC (AD-H, 0.46*25 cm, 5µm, hexane/isopropanol = 80/20, flow = 1.0 mL/min, detection at 210 nm) retention time = 9.181 min (major) and 11.231 min (minor).

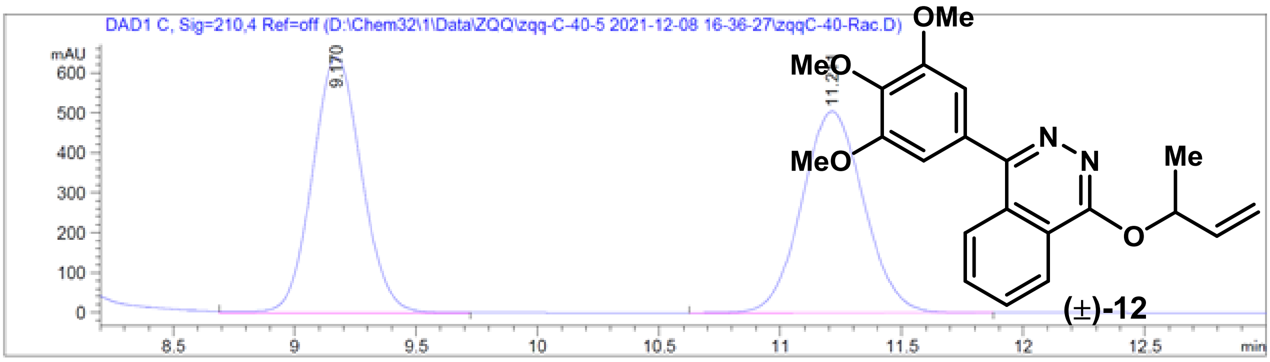

Signal 2: DAD1 C, Sig=210,4 Ref=off

Peak #	RetTime [min]	Type	Width [min]	Area [mAU*s]	Height [mAU]	Area %
1	9.170	MM R	0.2345	8993.25488	639.14502	50.1444
2	11.211	BB	0.2772	8941.47363	503.48059	49.8556

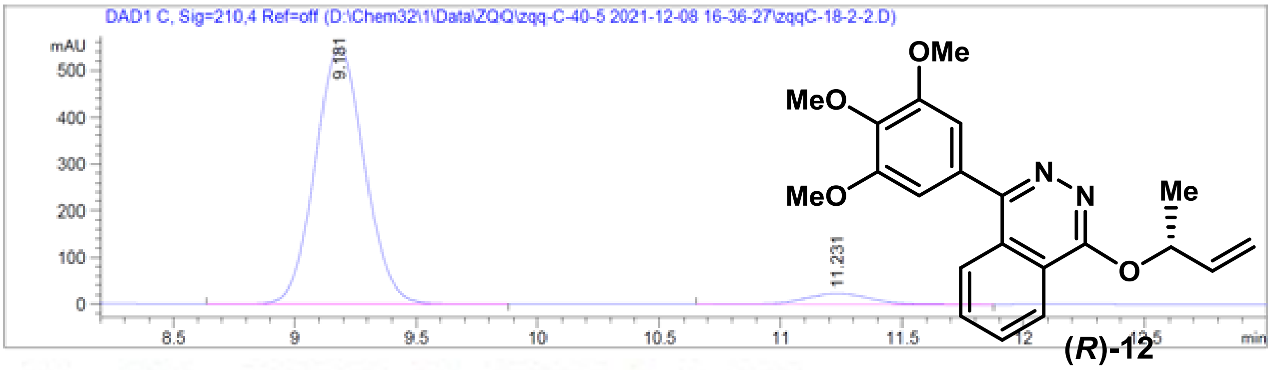

Signal 2: DAD1 C, Sig=210,4 Ref=off

Peak #	RetTime [min]	Type	Width [min]	Area [mAU*s]	Height [mAU]	Area %
1	9.181	BB	0.2212	7739.37793	546.41711	94.7081
2	11.231	BB	0.2834	432.44656	23.64190	5.2919

Supplementary Figure 190. HPLC chromatogram for **(*R*)-12**.

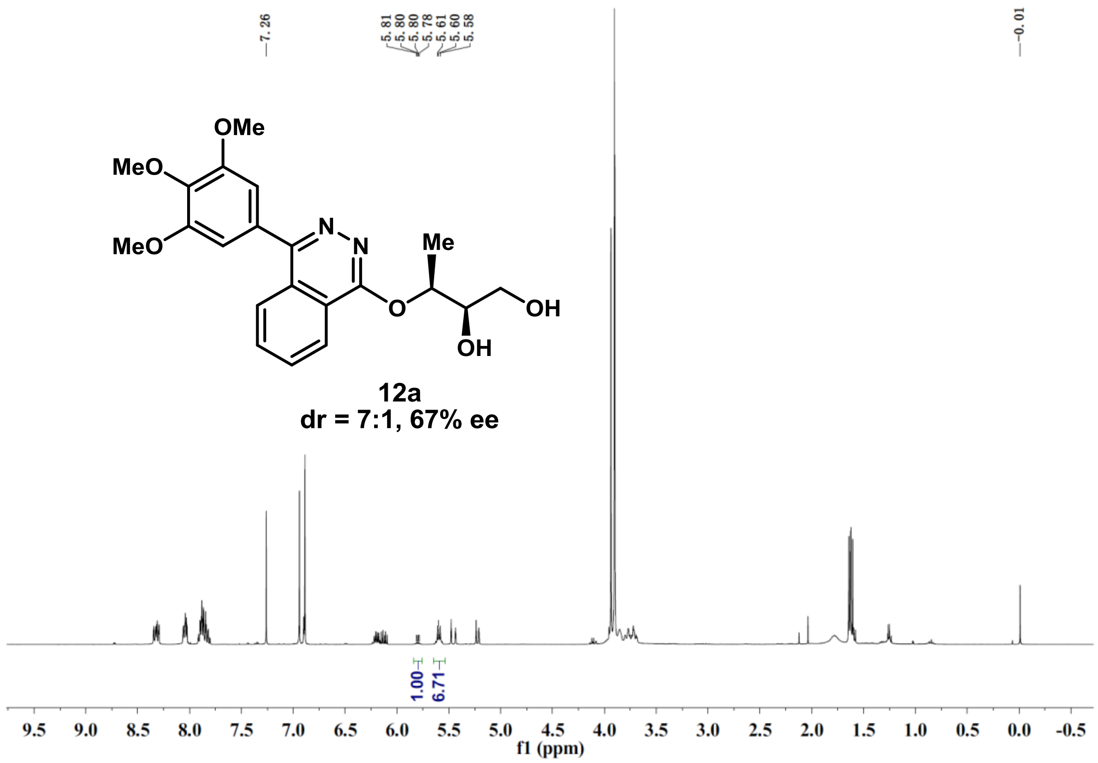

Supplementary Figure 191. ^{1}H NMR spectrum of crude mixture for diastereomeric ratio (dr).

HPLC (OJ-H, 0.46*25 cm, 5μm, hexane/isopropanol = 60/40, flow = 1.0 mL/min, detection at 210 nm), retention time = 7.717 min (major) and 13.615 min (minor).

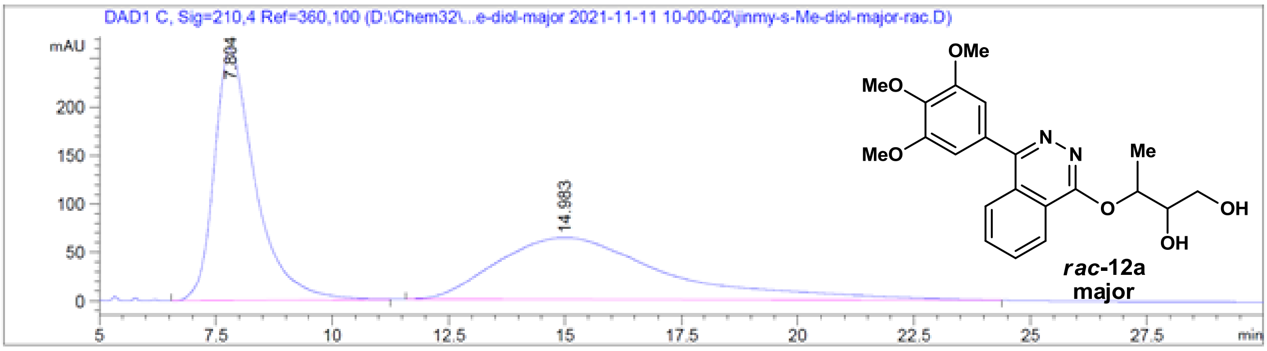

Signal 2: DAD1 C, Sig=210,4 Ref=360,100

Peak #	RetTime [min]	Type	Width [min]	Area [mAU*s]	Height [mAU]	Area %
1	7.804	BB	0.9030	1.57972e4	259.19754	49.7585
2	14.983	BB	2.9307	1.59506e4	63.62598	50.2415

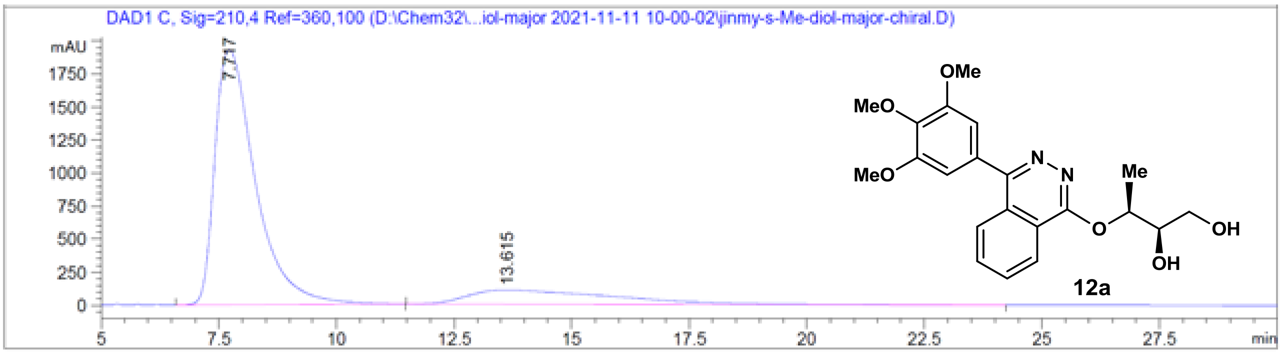

Signal 2: DAD1 C, Sig=210,4 Ref=360,100

Peak #	RetTime [min]	Type	Width [min]	Area [mAU*s]	Height [mAU]	Area %
1	7.717	BB	0.8083	1.16058e5	1928.06104	83.2399
2	13.615	BB	2.5727	2.33679e4	108.24039	16.7601

Supplementary Figure 192. HPLC chromatogram for **12a**.

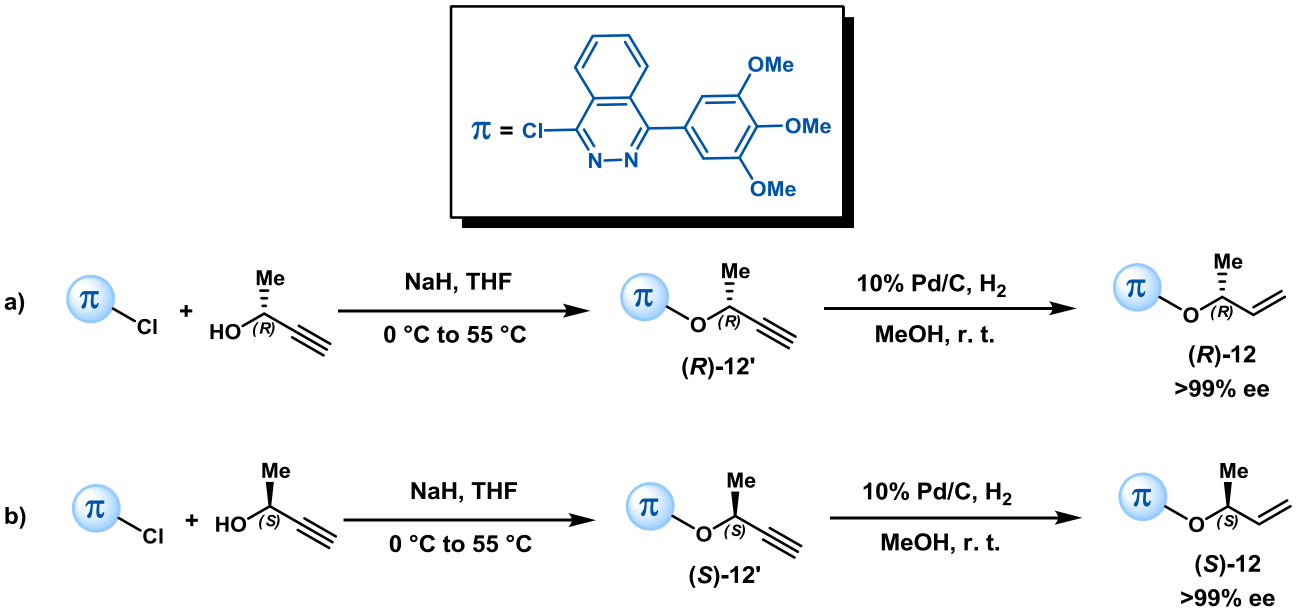

Supplementary Figure 193. Preparation of enantiomerically pure **(*R*)-12** and **(*S*)-12** from commercially available **(*R*)-3-Butyn-2-ol** and **(*S*)-3-butyn-2-ol**, respectively.

Analytic Data of (±)12'

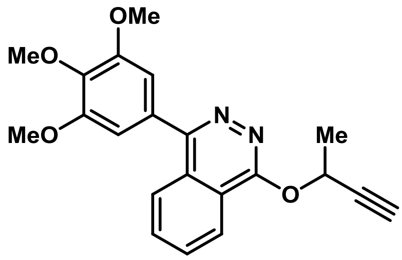

(±)12'

1-(but-3-yn-2-yloxy)-4-(3,4,5-trimethoxyphenyl)phthalazine

^{1}H NMR (400 MHz, CDCl$_3$): δ 8.40 – 8.29 (m, 1H), 8.07 (d, *J* = 7.6 Hz, 1H), 7.96 – 7.78 (m, 2H), 6.95 (s, 2H), 6.30 (m, 1H), 3.94 (s, 3H), 3.90 (s, 6H), 2.50 (d, *J* = 2.0 Hz, 1H), 1.82 (d, *J* = 6.6 Hz, 3H) ppm.

^{13}C NMR (101 MHz, CDCl$_3$): δ 158.4, 153.3, 138.9, 132.3, 131.8, 127.8, 126.1, 123.4, 120.3, 107.4, 83.0, 73.0, 62.6, 61.0, 56.3, 21.6 ppm.

HPLC (AD-H, 0.46*25 cm, 5µm, hexane/isopropanol = 80/20, flow = 1.0 mL/min, detection at 210 nm) retention time = 9.170 min and 11.211 min.

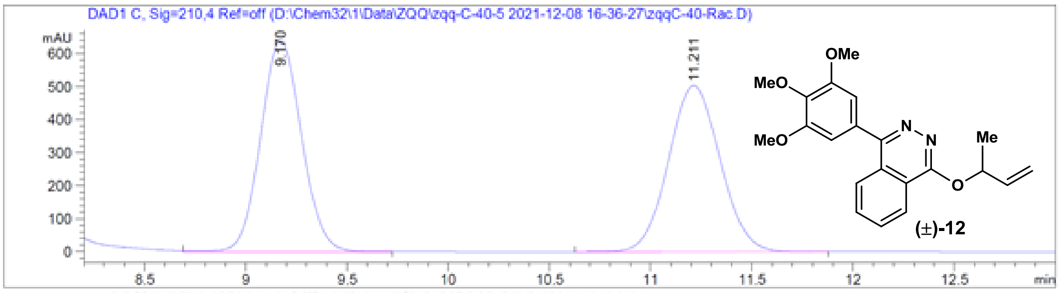

Signal 2: DAD1 C, Sig=210,4 Ref=off

Peak #	RetTime [min]	Type	Width [min]	Area [mAU*s]	Height [mAU]	Area %
1	9.170	MM R	0.2345	8993.25488	639.14502	50.1444
2	11.211	BB	0.2772	8941.47363	503.48059	49.8556

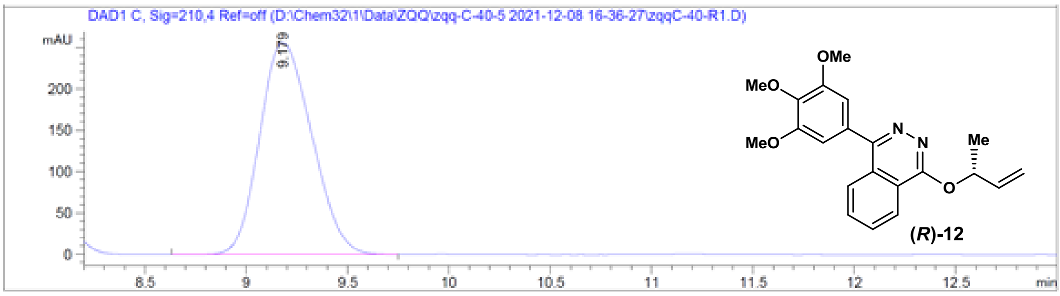

Signal 2: DAD1 C, Sig=210,4 Ref=off

Peak #	RetTime [min]	Type	Width [min]	Area [mAU*s]	Height [mAU]	Area %
1	9.179	BB	0.2823	4567.76367	255.86749	100.0000

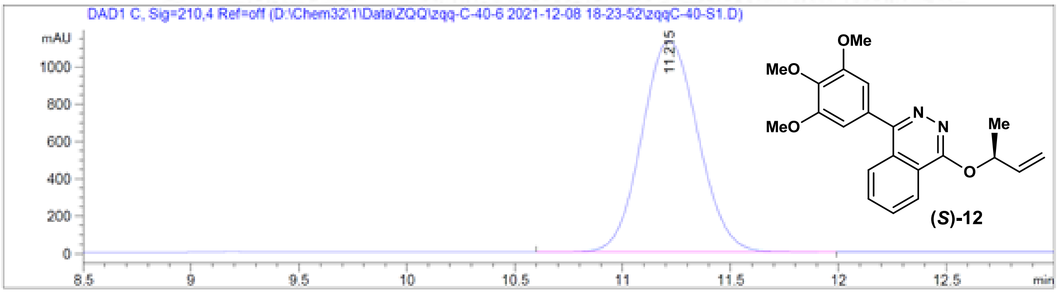

Signal 2: DAD1 C, Sig=210,4 Ref=off

Peak #	RetTime [min]	Type	Width [min]	Area [mAU*s]	Height [mAU]	Area %
1	11.215	BB	0.2832	2.04978e4	1132.20508	100.0000

Supplementary Figure 194. HPLC chromatogram for ***(R)*-12** and ***(S)*-12**.

3.2 Computational studies.

3.2.1. Computational methods

Molecular structures for computations were built using *GaussView*[1]. Initial conformational searches of transition states were performed with *xTB*[2] using the GFN-FF force field[3]. Then the semi-empirical method GFN2-xTB[4] was used to optimize the resulting conformers. Conformers within 5 kcal/mol were subject to DFT calculations. DFT geometry optimizations and frequency calculations were performed with *Gaussian 16*[5]. All structures were optimized at the wB97XD[6]/def2-SVP[7] level of theory. Normal mode vibrational frequency calculations at the same level confirmed that the optimized structures are minima (no imaginary frequency) or transition states (one imaginary frequency). To obtain electronic energies of higher accuracy, single-point energies were calculated on the optimized structures at the PBE0[8]+D3(BJ)[9]/def2-TZVPP[7] level. Natural population analysis (NPA) and second order perturbation theory analysis (E(2)) were calculated using *NBO 7.0* program[10]. E2 is the energy of the interaction between "filled" natural bonding orbitals (NBOs) and "empty" NBOs. For each donor NBO (i) and acceptor NBO (j), the donor-acceptor stabilization energy E(2) associated with i → j delocalization is estimated as

$$E(2) = \Delta E_{ij}^{(2)} = \frac{q_i F(i,j)^2}{\varepsilon_j - \varepsilon_i} \qquad (1)$$

where q_i is the donor orbital occupancy, and $F(i,j)$ is the off-diagonal NBO Fock matrix element[10].

To better illustrate and visualize our proposed π-π interactions, a Non-Covalent-Interaction (NCI) analysis[11] was performed on the key transition states using VMD[12] as the visualization tool.

Calculations of stabilizing non-covalent interaction between the interacting fragments were performed at PBE0-D3(BJ)/def2-TZVPP level of theory in gas-phase. The stabilization energy of non-covalent interaction (ΔE) was calculated by:

$$\Delta E_{int} = E_{complex} - E_{separate} \qquad (2)$$

*E*complex refers to the gas-phase single point energy of the interacting ester group and aryl group. The geometry of the interacting fragments was taken from the optimized geometry of **TS-(*R*)** and **TS-(*S*)**. E_{separate} ($E_{\text{Frag-ester}}$ and $E_{\text{Frag-Ar}}$) refers to the gas phase energies of each fragment.

Energy decomposition analysis (EDA)[14-16] was performed at the PBE0/TZ2P[17] level using the ADF software[18]. In the EDA scheme, the interaction energy (ΔE_{int}) has been decomposed into four terms as shown below,

$$\Delta E_{int} = \Delta E_{elstat} + \Delta E_{pauli} + \Delta E_{orb} + \Delta E_{disp} \tag{3}$$

The quasiclassical Coulomb interaction ΔE_{elstat} was contributed by frozen charge density of two fragments (A and B) at equilibrium geometry of to the complex.

$$\Delta E_{elstat} = \sum_{\alpha\epsilon A}\sum_{\beta\in B}\frac{Z_\alpha Z_\beta}{R_{\alpha\beta}} + \int dr V_B(r)\,\rho_A(r) + \int dr V_A(r)\,\rho_B(r) + \iint dr_1 dr_2\frac{\rho_A(r_1)\rho_B(r_2)}{r_{12}} \tag{4}$$

The second term is Pauli repulsion ΔE_{pauli} which is energy difference between ${E_{AB}}^0$ and E^0. For the fragment wavefunction $\psi_A\psi_B$, which is normalized against Pauli principle.

$$\Psi^0 = N\hat{A}\{\Psi_A\Psi_B\} \tag{5}$$

$$\Delta E_{pauli} = E_{AB}^0 - E^0 \tag{6}$$

Fort the ΔE_{orb} term, the Ψ^0 is relaxed to the final state Ψ_{AB} of the molecule A-B with the energy E_{AB}. The associated energy lowering comes from the orbital mixing, and thus, it can be identified as covalent contribution to the chemical bond. It is termed orbital interaction

$$E_{orb} = E_{AB} - E_{AB}^0 \tag{7}$$

3.2.2. Conformational analysis

Due to the structural flexibility of the cinchona alkaloid ligand and the substrate, our computational investigation involves a large number of conformers as a result of bond rotation in the molecular structures. We performed a systematic analysis and extensive search of potential configurations and conformations for the transition state structures (*Supplementary Fig. 195*). The OsO_4 catalyst coordinated with the cinchona alkaloid ligand adopts a trigonal bipyramid (D3h) configuration and subsequently react with the alkene double bond using its two adjacent oxygens in OsO_4 during the dihydroxylation process. In theory, there are 6 different models to construct (3 types of axial–axial and 3 types of axial–equatorial arrangement). After geometry optimization, all conformations in the axial–axial arrangement were converted to axial –equatorial. In addition, because of steric hindrance we excluded one types of axial–equatorial arrangement. To account for all possible stereoisomeric transition state structures, we considered two the two faces of the alkene moiety and two different orientations of the substrate for each of the *S*- and *R*-substrates, respectively, which added to a total number of 16 starting geometries for subsequent conformational searches.

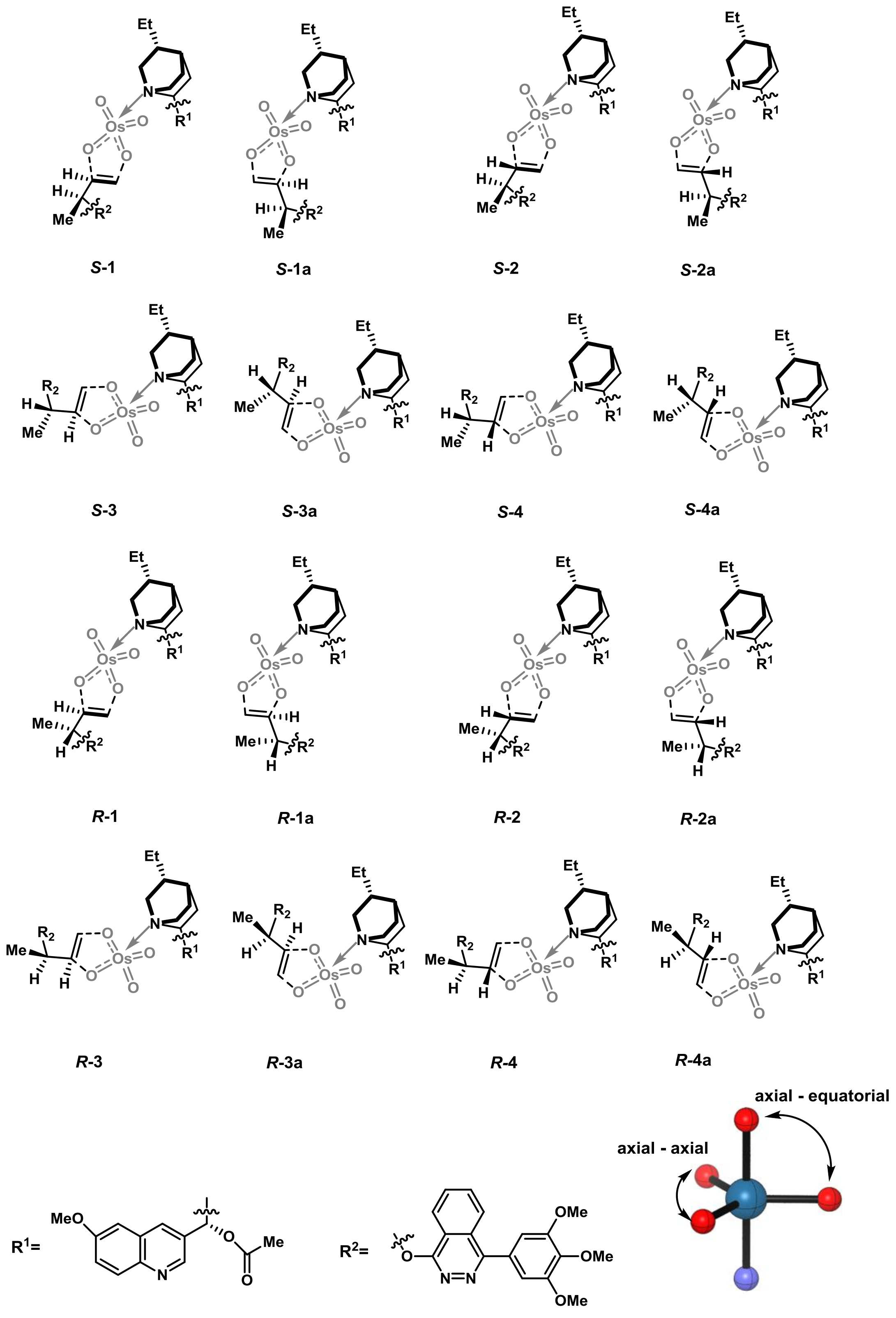

Supplementary Figure 195. Starting geometries of transition state structures for subsequent conformational searches.

All 16 starting geometries of transition state structures with three fixed bond lengths (two reacting C-O bond at 2.1 Å, and N-Os bond at 2.5 Å) were then subjected to conformational searches by restrained molecular dynamics (MD) simulations with *xTB*[2] using the GFN-FF force field[3]. Temperature for the MD simulations was set to 400K, with a time gap of 50 fs and simulation time of 100 ps. A total number of 695 and 743 conformations were generate the *S*- and *R*-substrates, respectively. Then the semi-empirical method GFN2-xTB[4] was used to optimize the resulting structures. Conformers within 5 kcal/mol were subject to DFT calculations. There might be other bonds being formed during the geometry optimization process, not the dihydroxylation reaction, in some structures, which were manually deleted. All resulting structures were re-optimized at the wB97XD[6]/def2-SVP[7] level of theory. To obtain electronic energies of higher accuracy, single-point energies were calculated on the optimized structures at the PBE0[8]+D3(BJ)[9]/def2-TZVPP[7] level. Conformations within 3 kcal/mol of relative Gibbs free energies are shown in *Supplementary Fig. 196* and *197*.

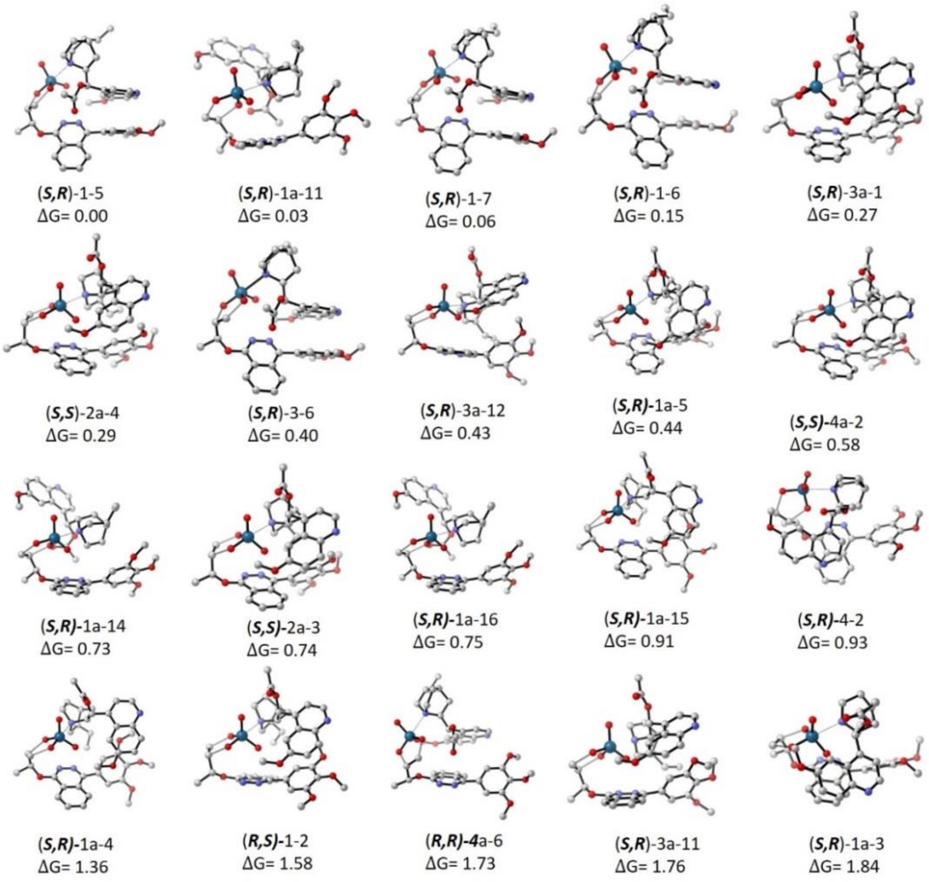

Supplementary Figure 196. Lowest-energy transition state structures computed at the PBE0+D3(BJ)/def2-TZVPP//wB97XD/def2-SVP level, arranged according to their relative Gibbs free energies (kcal/mol).

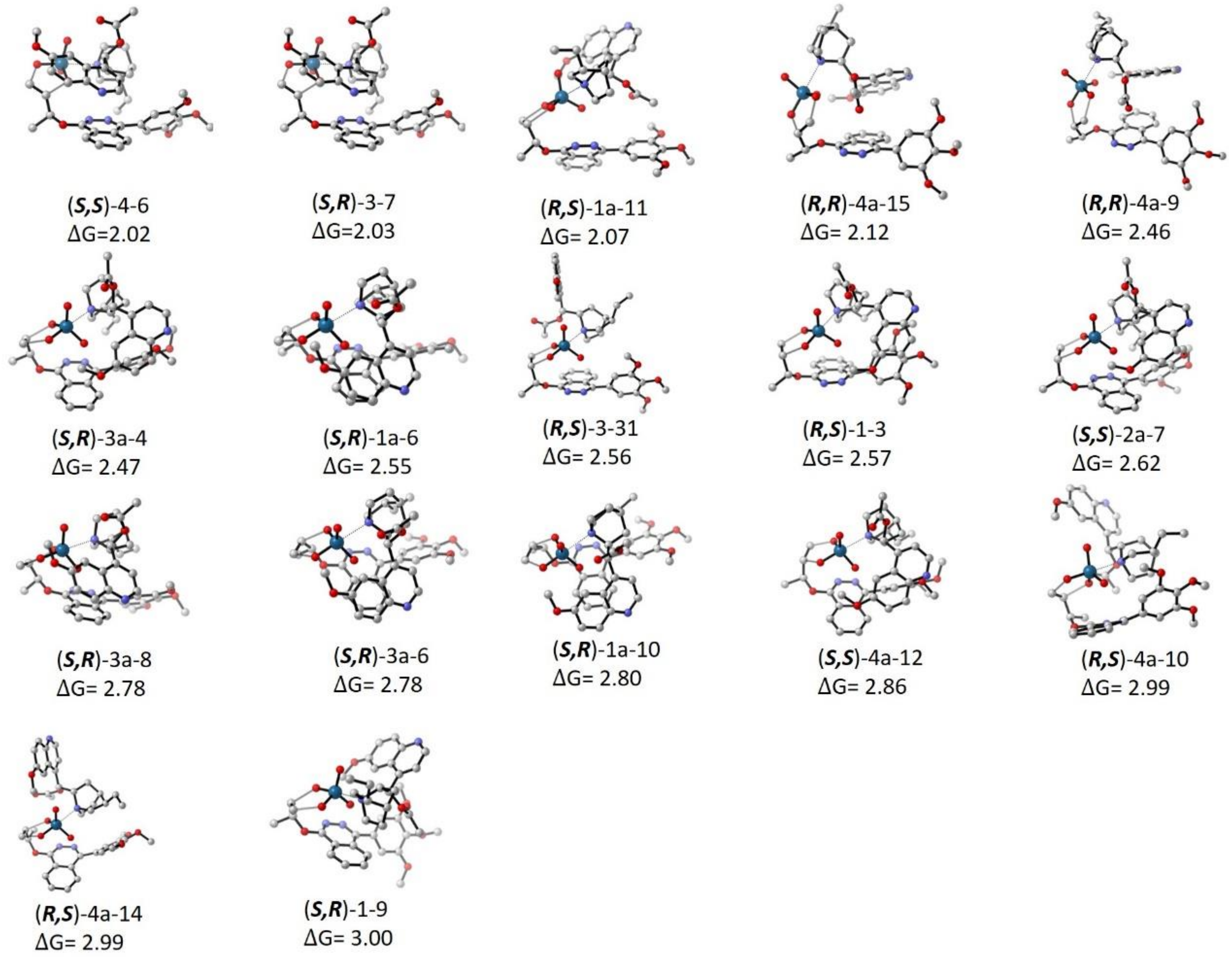

Supplementary Figure 197. Lowest-energy transition state structures computed at the PBE0+D3(BJ)/def2-TZVPP//wB97XD/def2-SVP level, arranged according to their relative Gibbs free energies (kcal/mol) (continued).

The electronic energies (*E*), enthalpies (*H*), Gibbs free energies (*G*) and corresponding percentages (Boltzmann distribution) for these 37 structures are shown in *Supplementary Table 2*.

Supplementary Table 2. The electronic energies (*E*), enthalpies (*H*), Gibbs free energies (*G*) (in Hartree) and corresponding percentages (Boltzmann distribution) for the transition states structures.

Entry	*E*	*H*	*G*	Percentage
(***S***,***R***)-1-5	-2803.874817	-2802.923456	-2803.066753	12.38%
(***S***,***R***)-1a-11	-2803.868468	-2802.917856	-2803.066706	11.73%
(***S***,***R***)-1-7	-2803.874715	-2802.923348	-2803.066656	11.07%
(***S***,***R***)-1-6	-2803.874233	-2802.922656	-2803.066509	9.34%
(***S***,***R***)-3a-1	-2803.874	-2802.922721	-2803.066319	7.50%
(***S***,***S***)-2a-4	-2803.871843	-2802.92029	-2803.066298	7.32%
(***S***,***R***)-3-6	-2803.874686	-2802.923118	-2803.066113	5.91%
(***S***,***R***)-3a-12	-2803.871587	-2802.920339	-2803.066068	5.61%
(***S***,***R***)-1a-5	-2803.871254	-2802.92033	-2803.06605	5.50%
(***S***,***S***)-4a-2	-2803.873109	-2802.921327	-2803.065832	4.27%
(***S***,***R***)-1a-14	-2803.868084	-2802.917278	-2803.065594	3.24%
(***S***,***S***)-2a-3	-2803.873386	-2802.921675	-2803.06557	3.15%
(***S***,***R***)-1a-16	-2803.868084	-2802.917274	-2803.065559	3.11%
(***S***,***R***)-1a-15	-2803.871735	-2802.9203	-2803.065305	2.32%
(***S***,***R***)-4-2	-2803.86965	-2802.918171	-2803.065267	2.22%
(***S***,***R***)-1a-4	-2803.871163	-2802.919826	-2803.064582	1.01%
(***R***,***S***)-1-2	-2803.869872	-2802.918932	-2803.064235	0.67%
(***R***,***R***)-4a-6	-2803.86677	-2802.916244	-2803.063988	0.51%
(***S***,***R***)-3a-11	-2803.870188	-2802.918745	-2803.063944	0.48%
(***S***,***R***)-1a-3	-2803.868177	-2802.91689	-2803.063818	0.42%
(***S***,***R***)-4-6	-2803.865371	-2802.914327	-2803.063533	0.30%
(***S***,***R***)-3-7	-2803.872654	-2802.921073	-2803.063517	0.29%
(***R***,***S***)-1a-11	-2803.866321	-2802.915134	-2803.063452	0.27%
(***R***,***R***)-4a-15	-2803.865814	-2802.91483	-2803.063375	0.25%
(***R***,***R***)-4a-9	-2803.866056	-2802.915433	-2803.062833	0.13%
(***S***,***R***)-3a-4	-2803.869829	-2802.918911	-2803.062821	0.13%
(***S***,***R***)-1a-6	-2803.865486	-2802.914351	-2803.062688	0.11%
(***R***,***S***)-3-31	-2803.864574	-2802.913636	-2803.06268	0.11%
(***R***,***S***)-1-3	-2803.869655	-2802.918555	-2803.062663	0.11%
(***S***,***S***)-2a-7	-2803.869515	-2802.91802	-2803.062581	0.10%
(***S***,***R***)-3a-8	-2803.864485	-2802.913276	-2803.062325	0.07%
(***S***,***R***)-3a-6	-2803.864481	-2802.913277	-2803.062317	0.07%
(***S***,***R***)-1a-10	-2803.864484	-2802.913272	-2803.062298	0.07%
(***S***,***S***)-4a-12	-2803.86562	-2802.914883	-2803.0622	0.06%
(***R***,***S***)-4a-10	-2803.865686	-2802.914637	-2803.061984	0.05%
(***R***,***S***)-4a-14	-2803.865688	-2802.914638	-2803.06198	0.05%
(***S***,***R***)-1-9	-2803.86421	-2802.913566	-2803.061954	0.05%

3.2.3. Stereoselectivity-determining transition structures

For each of the four stereoisomeric products from racemic substrate **12**, the corresponding lowest energy transition structures were located and shown below in *Supplementary Fig. 198*. The computed selectivities are in good agreement with experimentally measured dr and ee values.

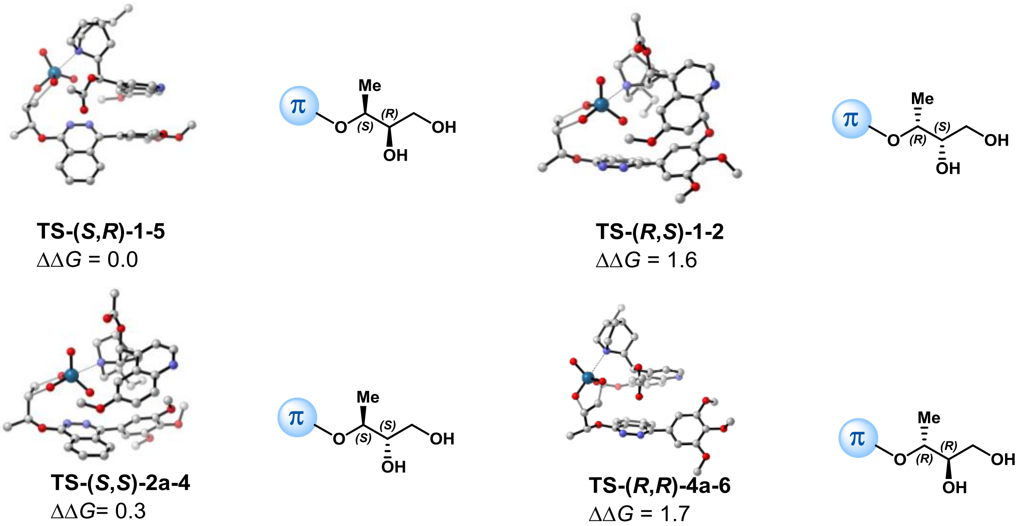

Supplementary Figure 198. Lowest-energy dihydroxylation TSs that lead to the four corresponding stereoisomeric products, respectively (relative free energies are in kcal/mol).

3.2.4. DFT-computed free energy profile

Free energy profile for the lowest-energy dihydroxylation TSs of the *S*- and *R*-substrates are shown in *Supplementary Fig. 199*. Using (±)-**12** as a model substrate, the computed free energy of activation for the dihydroxylation of the **(*R*)-12** via lowest transition-state **TS-(*R*)** [**(*R*, *S*)-1-2**] is 12.0 kcal/mol, as compared to that of **(*S*)-12** via lowest transition-state **TS-(*S*)** [**(*S*, *R*)-1-5**] being 13.6 kcal/mol, in good agreement of the observed selectivity factor for the kinetic resolution process. This process is irreversible, as indicated by the large exergonicity to generate **IM2-(*R*)** and **IM2-(*S*)** of -27.5 and -33.5 kcal/mol, respectively.

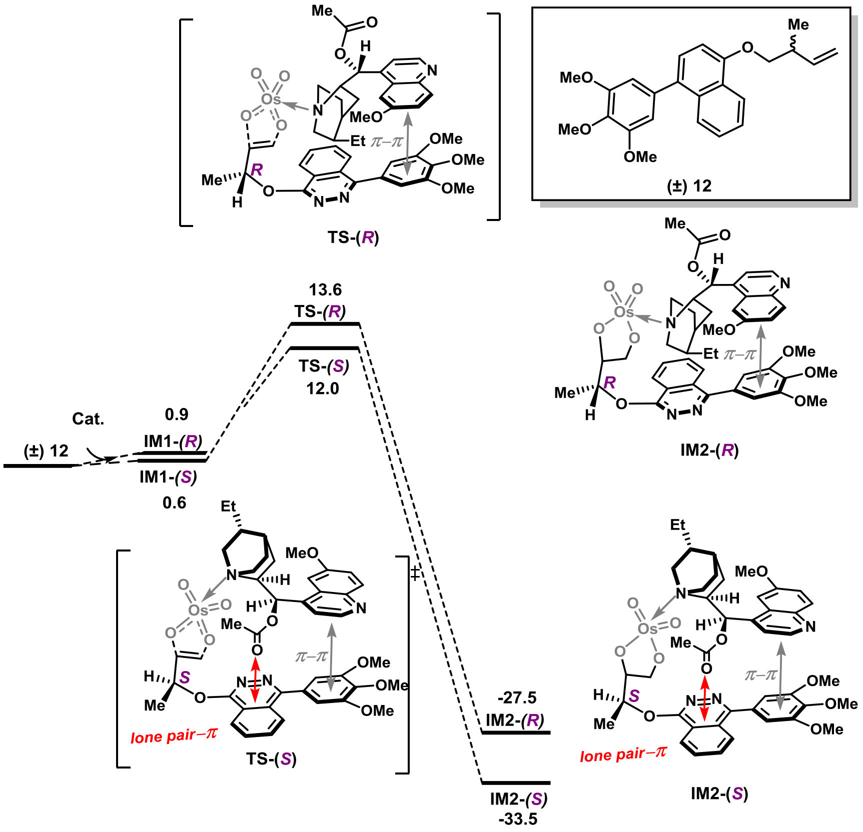

Supplementary Figure 199. DFT-computed free energy profiles for the lowest-energy dihydroxylation TSs of the *S*- and *R*-substrates, respectively (free energies are in kcal/mol).

3.2.5. Calculations of stabilizing non-covalent interaction

To better illustrate the non-covalent interactions involved in determining the stereoselectivity, Non-Covalent-Interaction[11] (NCI) analysis was performed on selected transition state structures, with VMD[12] as the visualization tool. Results shown in *Supplementary Fig. 200* reveal attractive Non-Covalent-Interactions present as pi-pi and lone pair-pi in **TS-(*S*)**, while lone pair-pi interaction is absent in **TS-(*R*)**. This result supports that dispersion is a significant component in the EDA calculations.

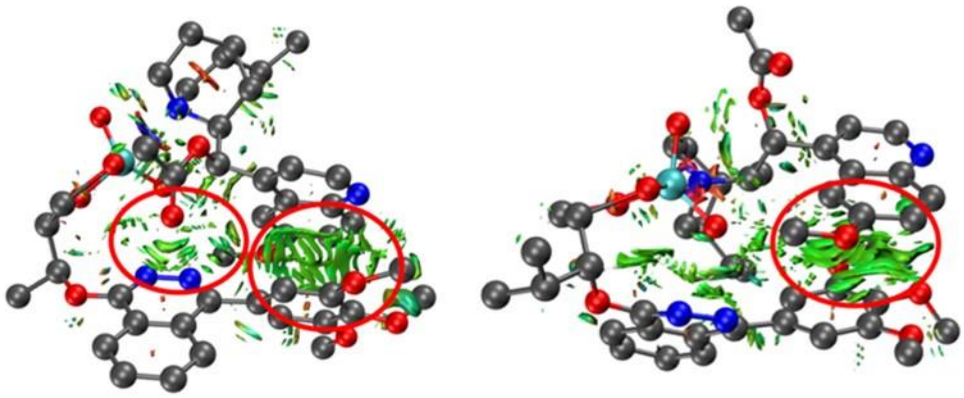

TS-(*S*) TS-(*R*)

Supplementary Figure 200. Noncovalent-Interaction (NCI) analysis on **TS-(*S*)** and **TS-(*R*).**

The quasiclassical Coulomb interaction (E_{elstat}) is another main component of the lone pair-pi interaction involved in TS-(*S*). As shown in *Supplementary Fig. 201A* one major difference for the two aromatic rings can be observed: phthalazine moiety (highlighted in blue color) is much more electron-poor than trimethoxyphenyl (highlighted in Red color). The O atom in the carbonyl group is electron-rich, which enables it to attract the phthalazine moiety through favorable Coulombic interactions.

For the orbital interaction term (E_{orb}), E2 analysis was performed to further investigate such orbital interactions. *Supplementary Fig. 201B* shows that the interaction between the p-orbital of O and the pi* orbital located at the C-N moiety in the phthalazine accounts for 0.14 kcal/mol stabilization energy. The overlap between the two orbitals is quite small.

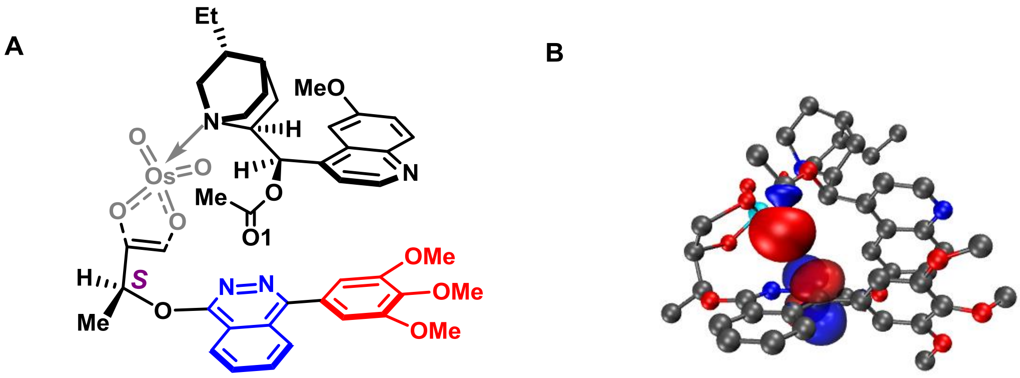

TS-(*S*) TS-(*S*)

NPA	
Red	+0.02
Blue	+0.15
O1	-0.59
E2 analysis	
p(O)→p(C-N)*	0.14

Supplementary Figure 201. Natural bonding orbitals analysis on TS-(*S*). A) Computed charges from natural population analysis (NPA) for key fragments. B) Second order perturbation theory (E2) analysis for key orbital interactions.

4 Supplementary Figures

4.1 NMR spectra

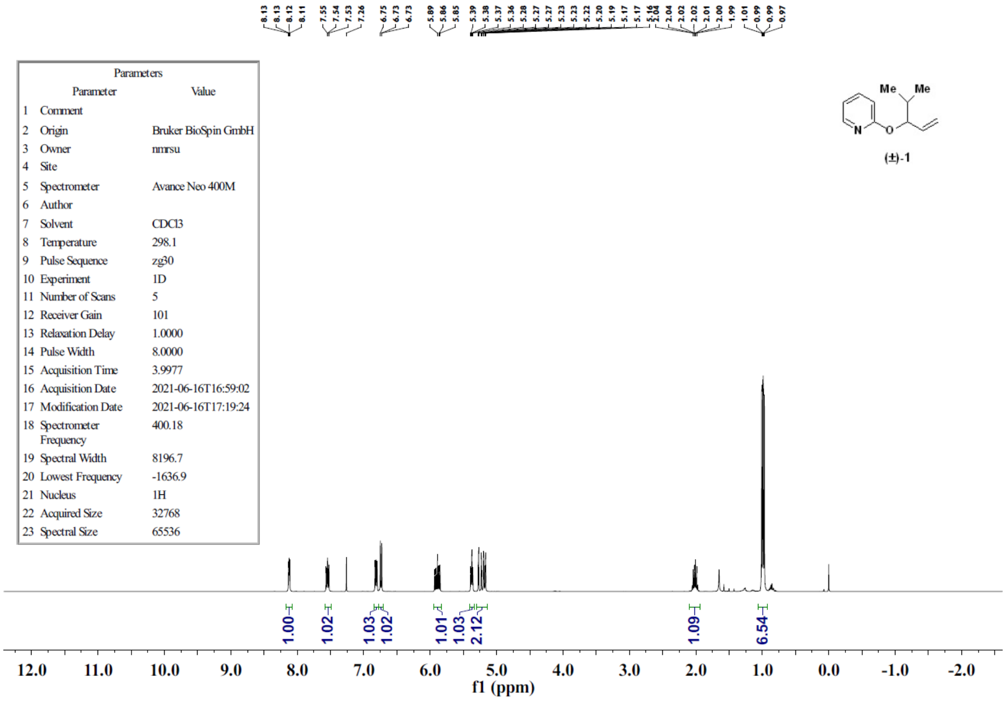

Supplementary Figure 202. ^{1}H NMR (400 MHz, $CDCl_3$) spectrum of compound **(±)-1**.

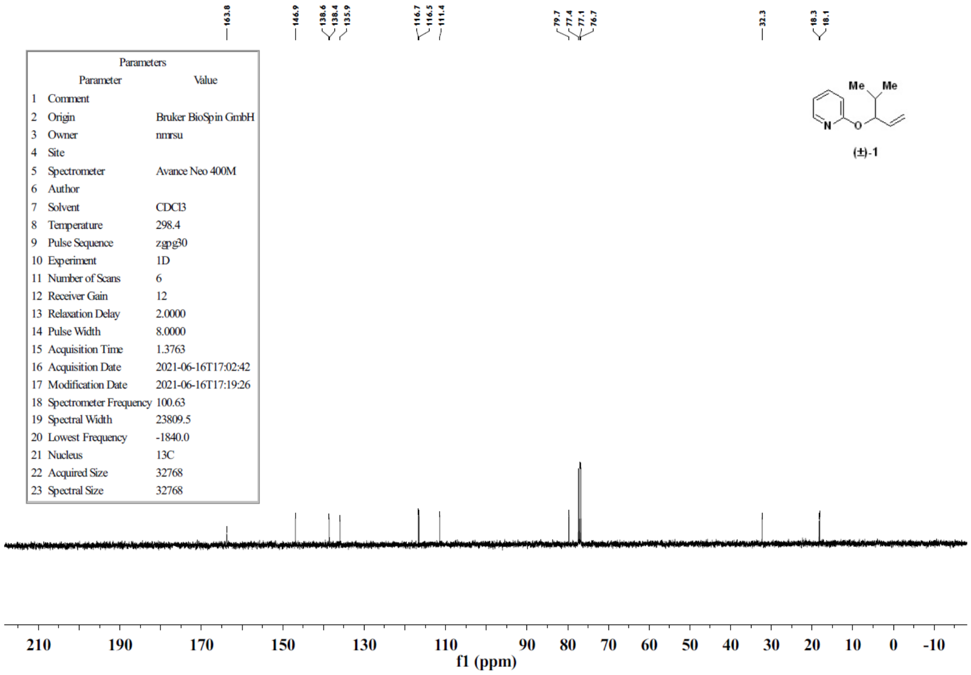

Supplementary Figure 203. ^{13}C NMR (101 MHz, $CDCl_3$) spectrum of compound **(±)-1**.

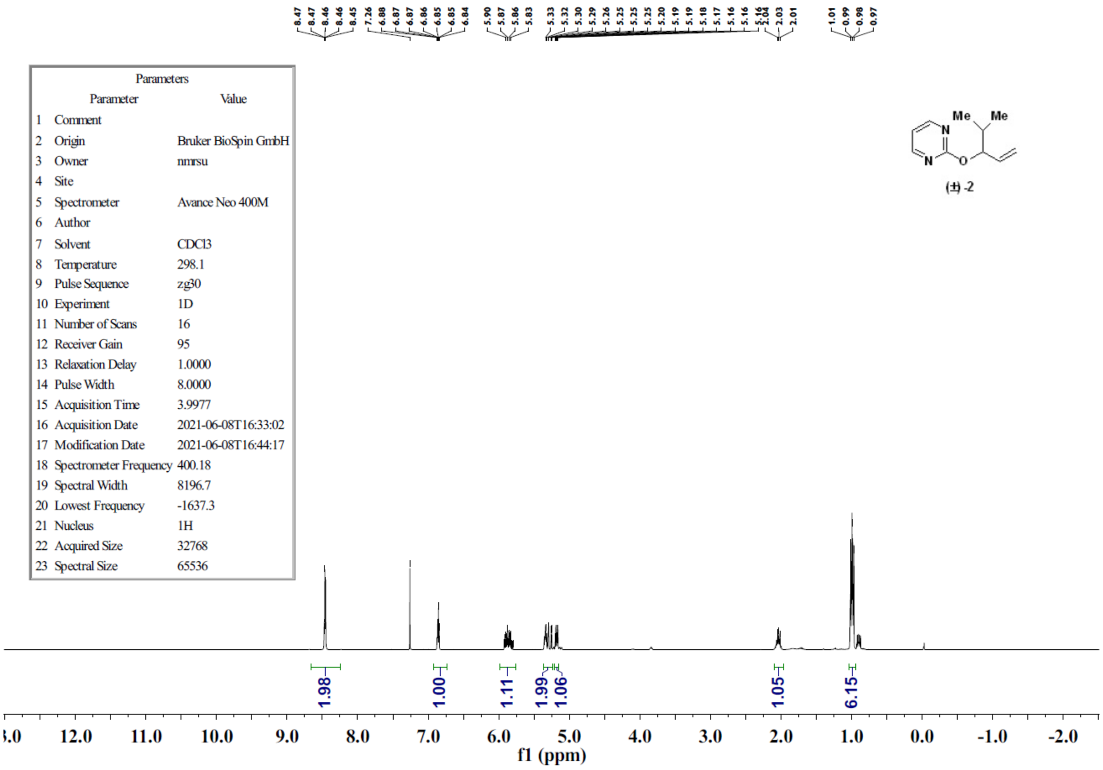

Supplementary Figure 204. ^{1}H NMR (400 MHz, $CDCl_3$) spectrum of compound **(±)-2**.

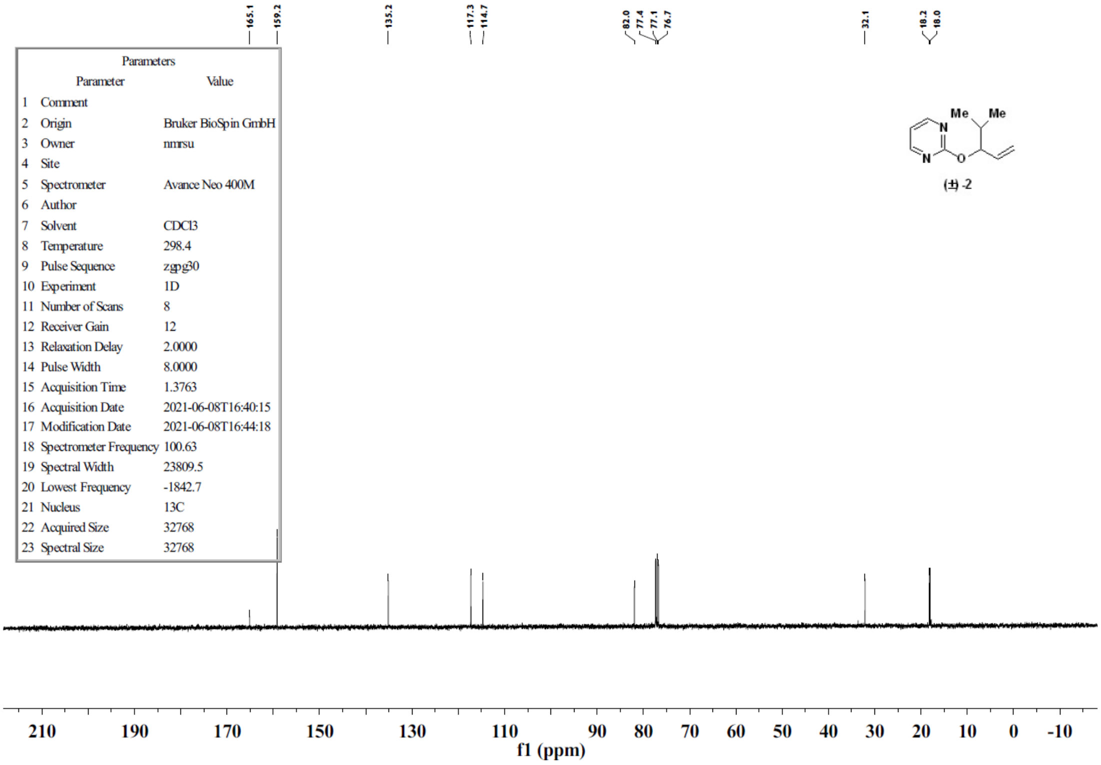

Supplementary Figure 205. ^{13}C NMR (101 MHz, $CDCl_3$) spectrum of compound **(±)-2**.

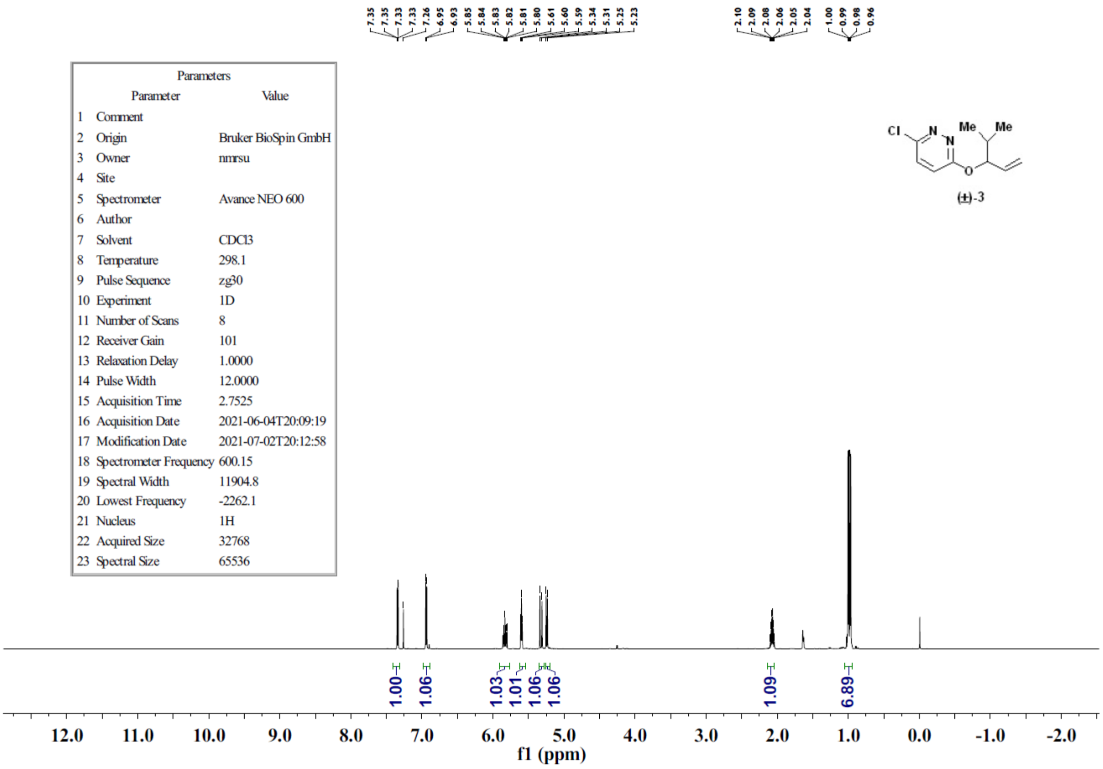

Supplementary Figure 206. ^{1}H NMR (600 MHz, $CDCl_3$) spectrum of compound **(±)-3**.

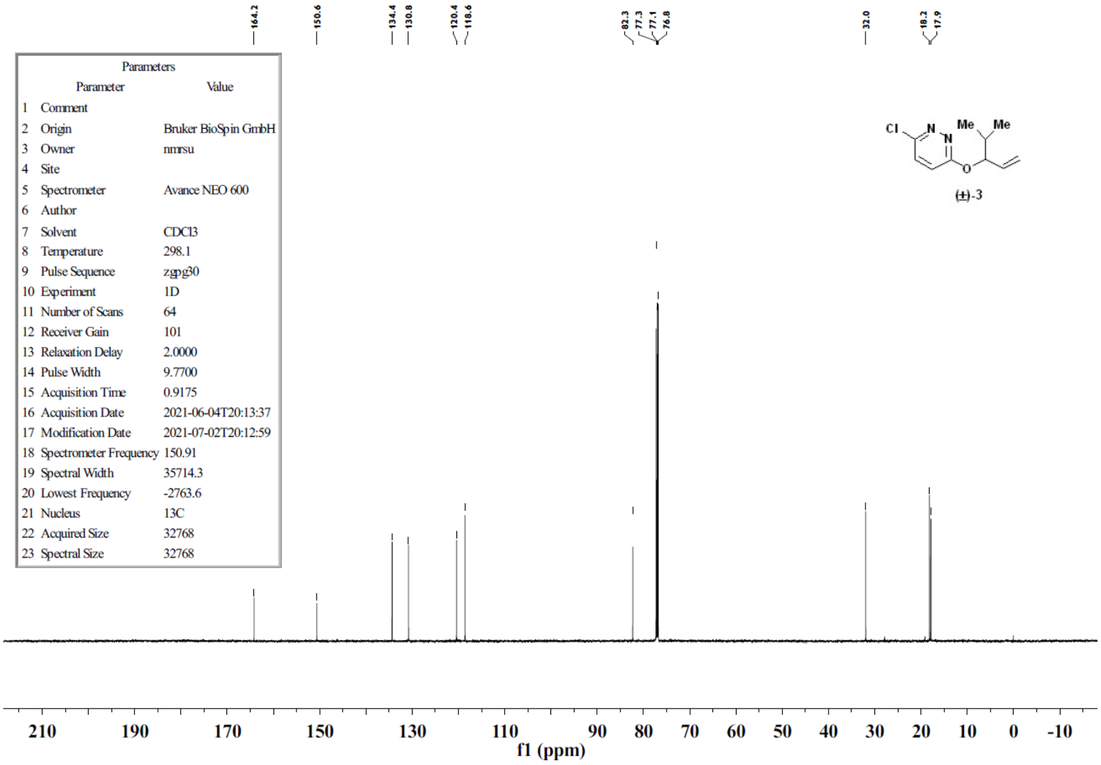

Supplementary Figure 207. ^{13}C NMR (151 MHz, $CDCl_3$) spectrum of compound **(±)-3**.

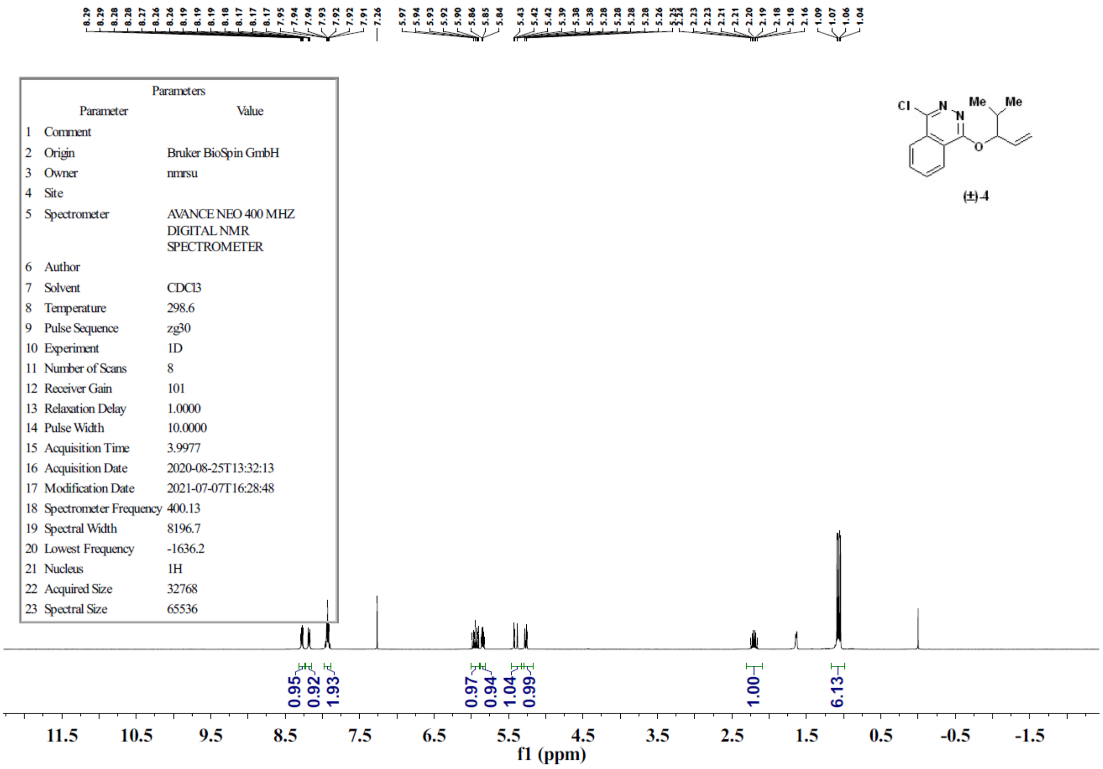

Supplementary Figure 208. ^{1}H NMR (600 MHz, $CDCl_3$) spectrum of compound **(±)-4**.

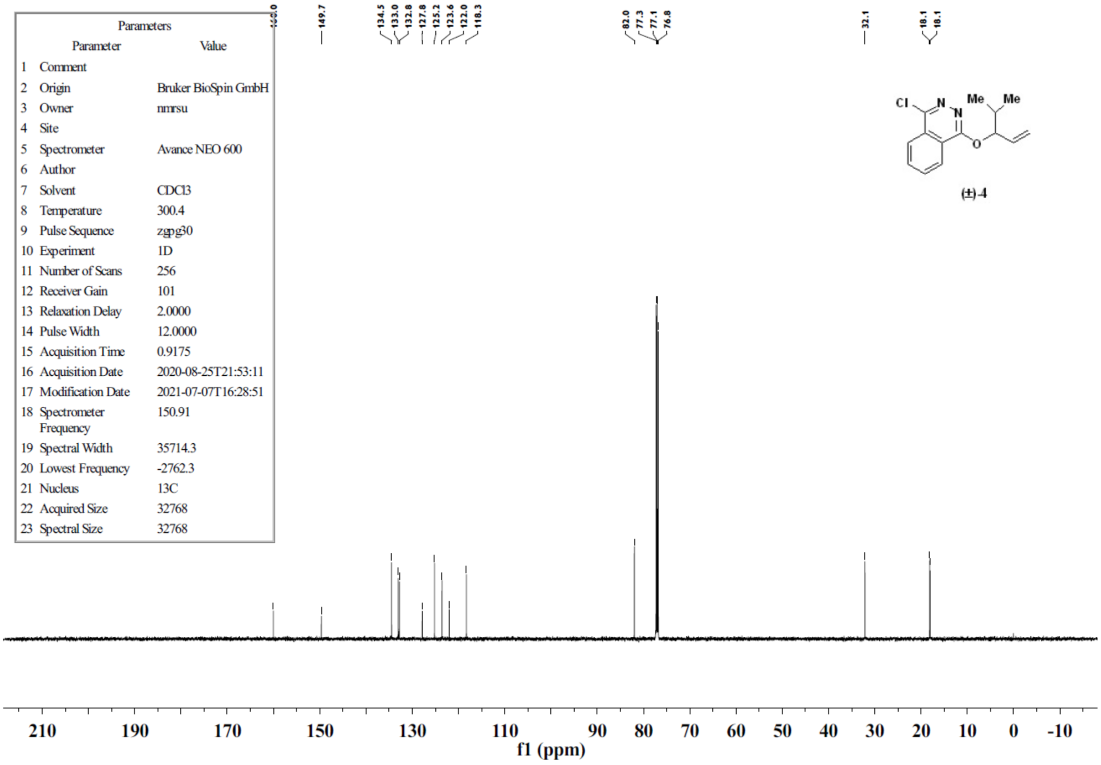

Supplementary Figure 209. ^{13}C NMR (151 MHz, $CDCl_3$) spectrum of compound **(±)-4**.

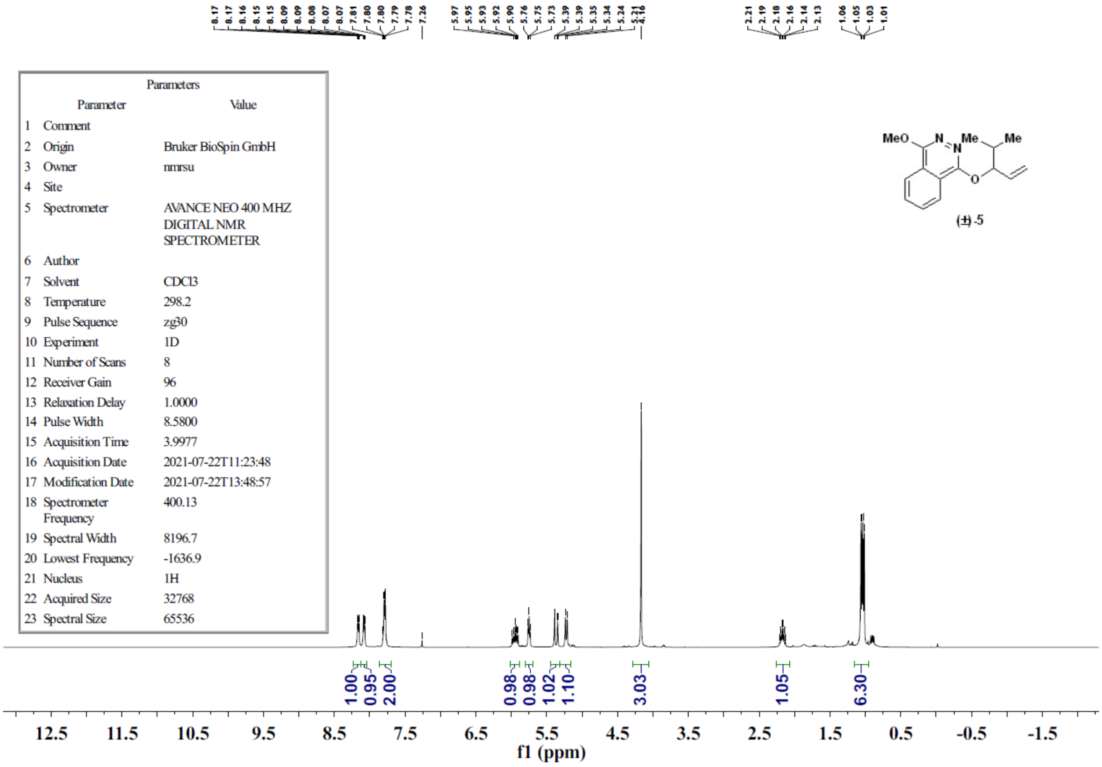

Supplementary Figure 210. ^{1}H NMR (600 MHz, $CDCl_3$) spectrum of compound **(±)-5**.

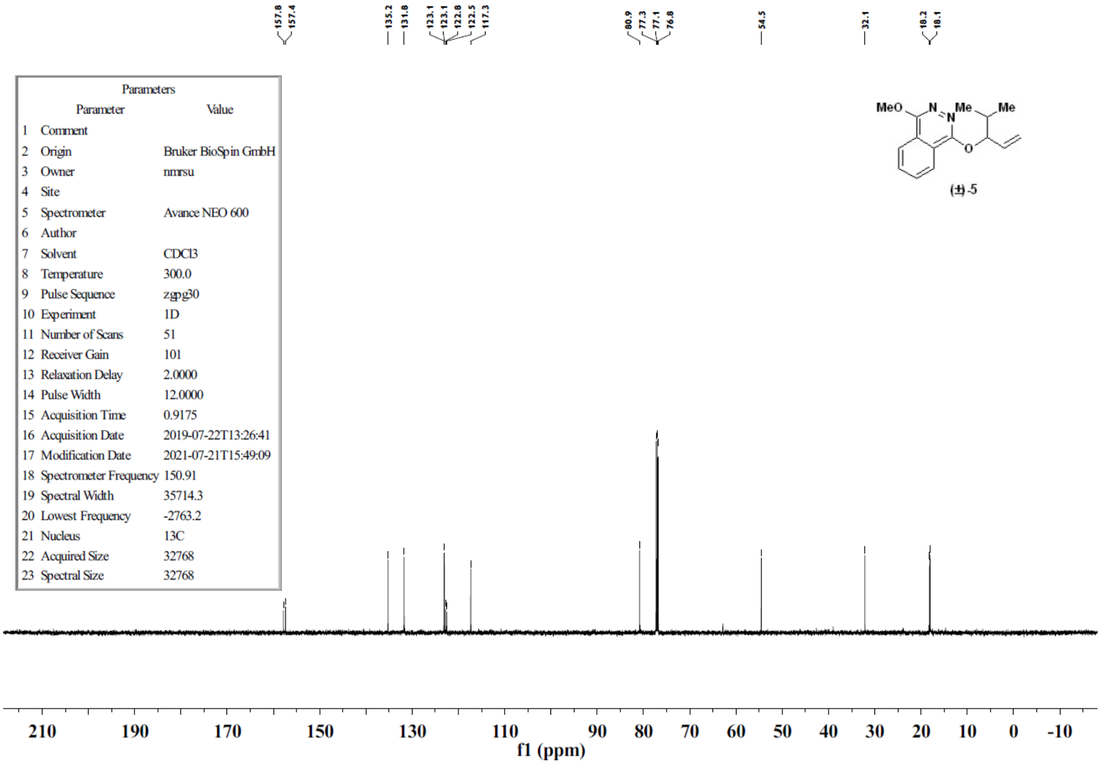

Supplementary Figure 211. ^{13}C NMR (151 MHz, $CDCl_3$) spectrum of compound **(±)-5**.

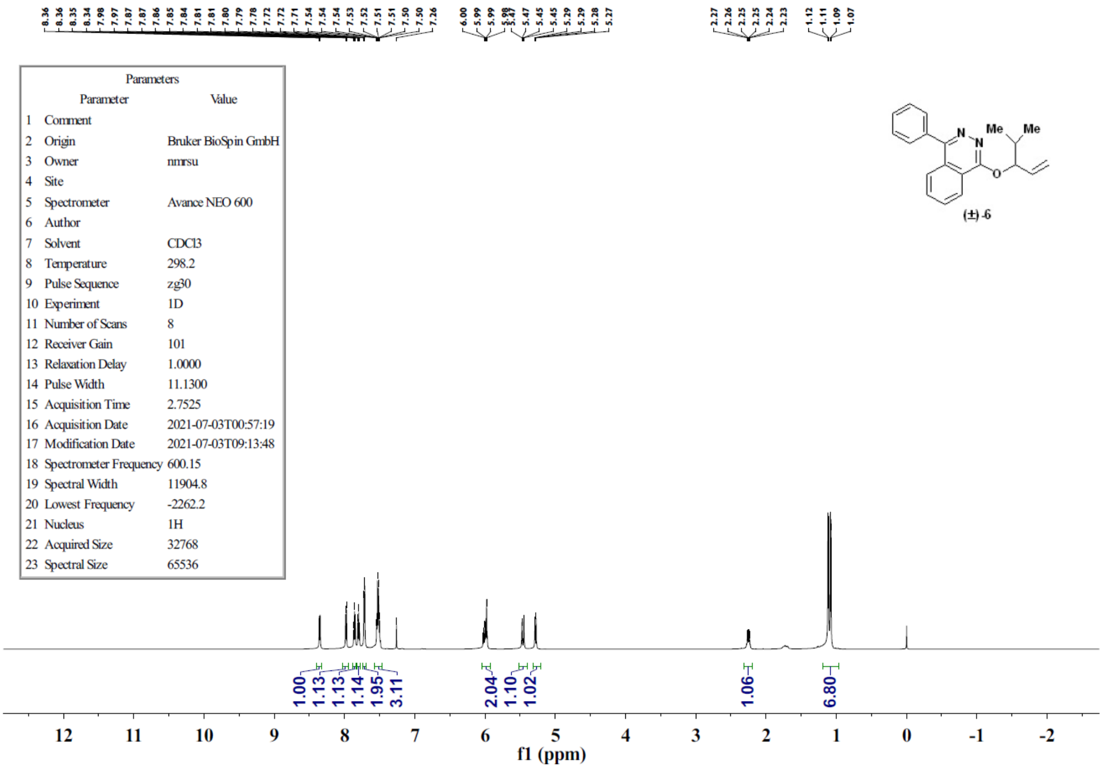

Supplementary Figure 212. ^{1}H NMR (600 MHz, $CDCl_3$) spectrum of compound **(±)-6**.

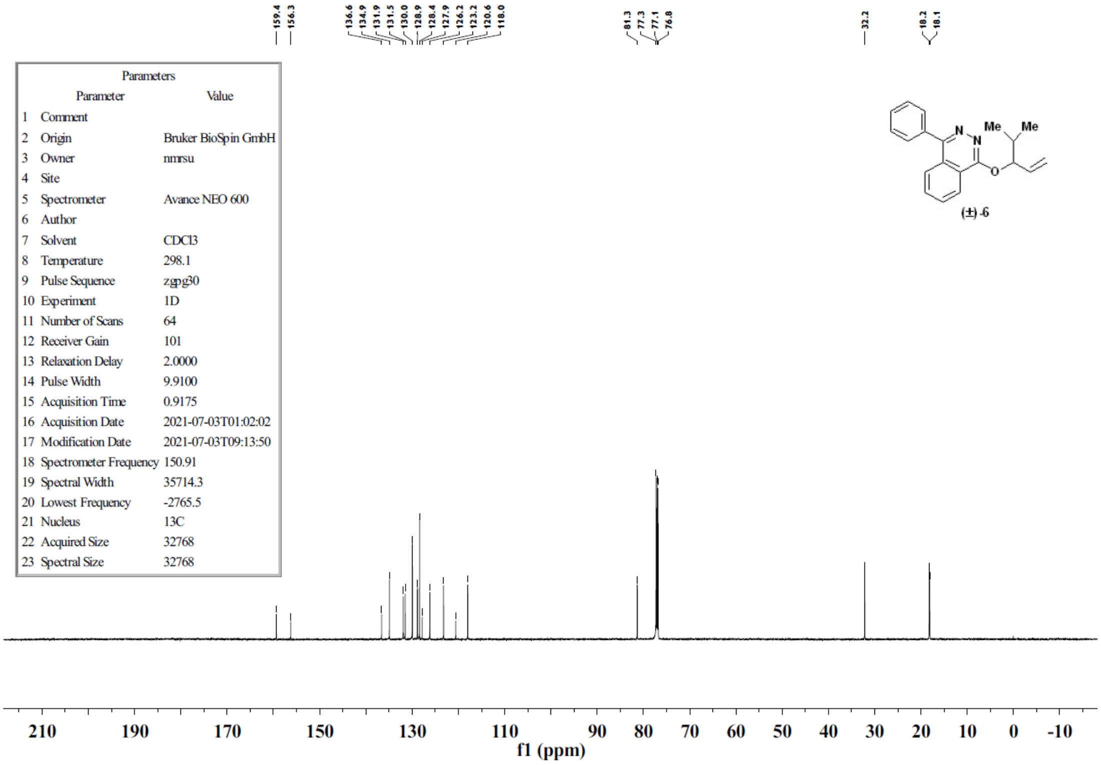

Supplementary Figure 213. ^{13}C NMR (151 MHz, $CDCl_3$) spectrum of compound **(±)-6**.

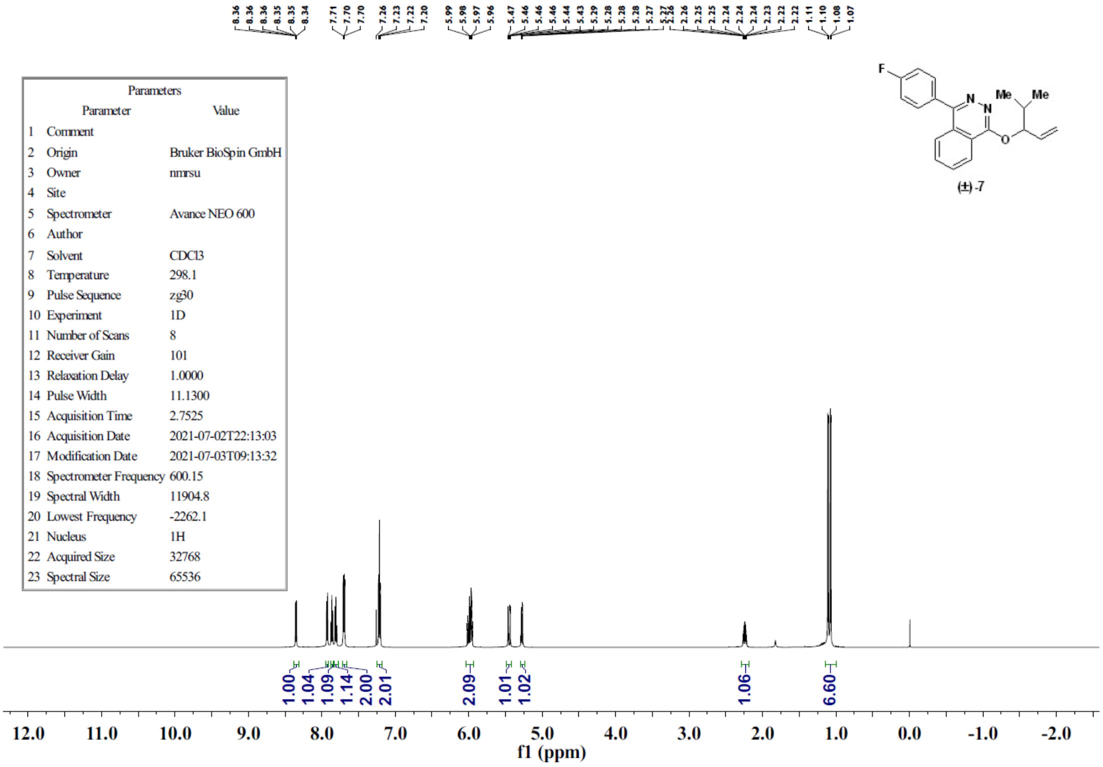

Supplementary Figure 214. ^{1}H NMR (600 MHz, $CDCl_3$) spectrum of compound **(±)-7**.

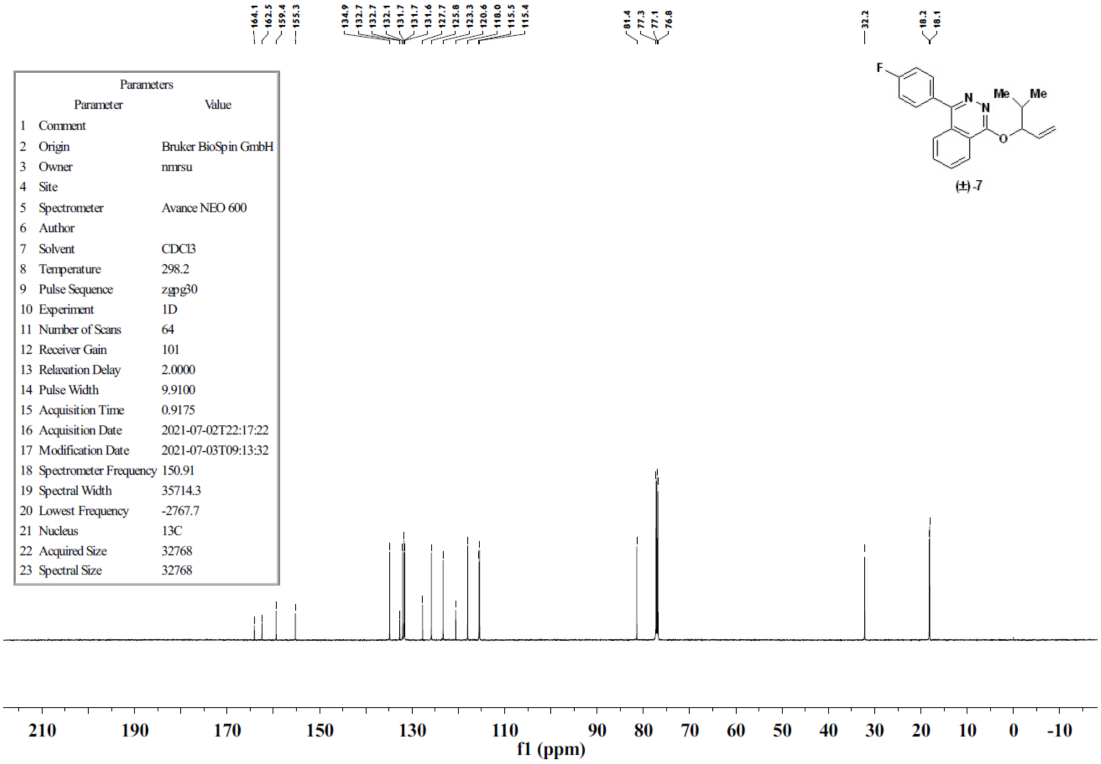

Supplementary Figure 215. ^{13}C NMR (151 MHz, $CDCl_3$) spectrum of compound **(±)-7**.

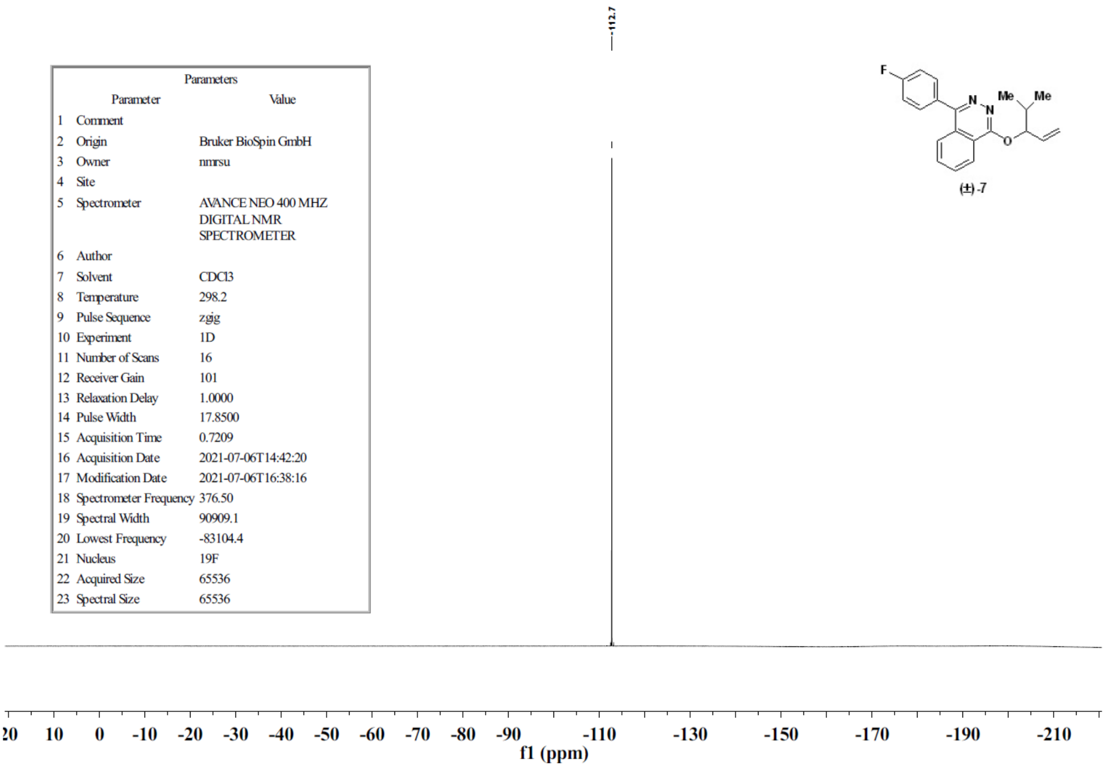

Supplementary Figure 216. ^{19}F NMR (376 MHz, $CDCl_3$) spectrum of compound **(±)-7**.

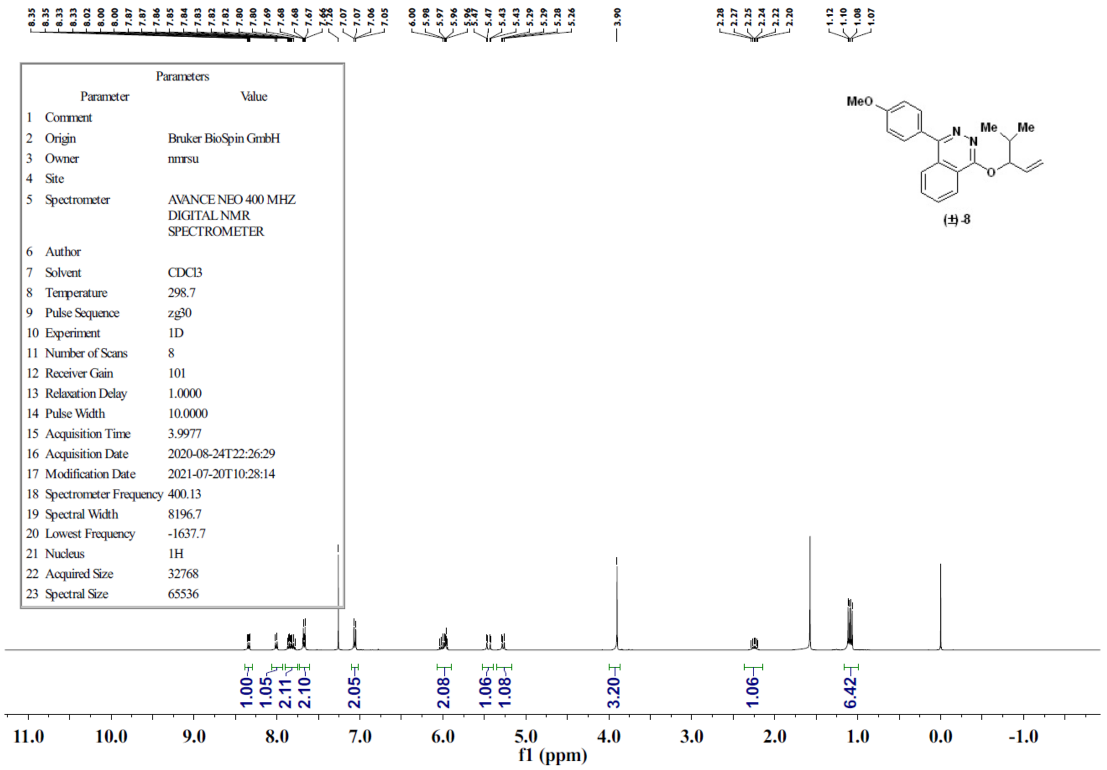

Supplementary Figure 217. ^{1}H NMR (400 MHz, $CDCl_3$) spectrum of compound **(±)-8**.

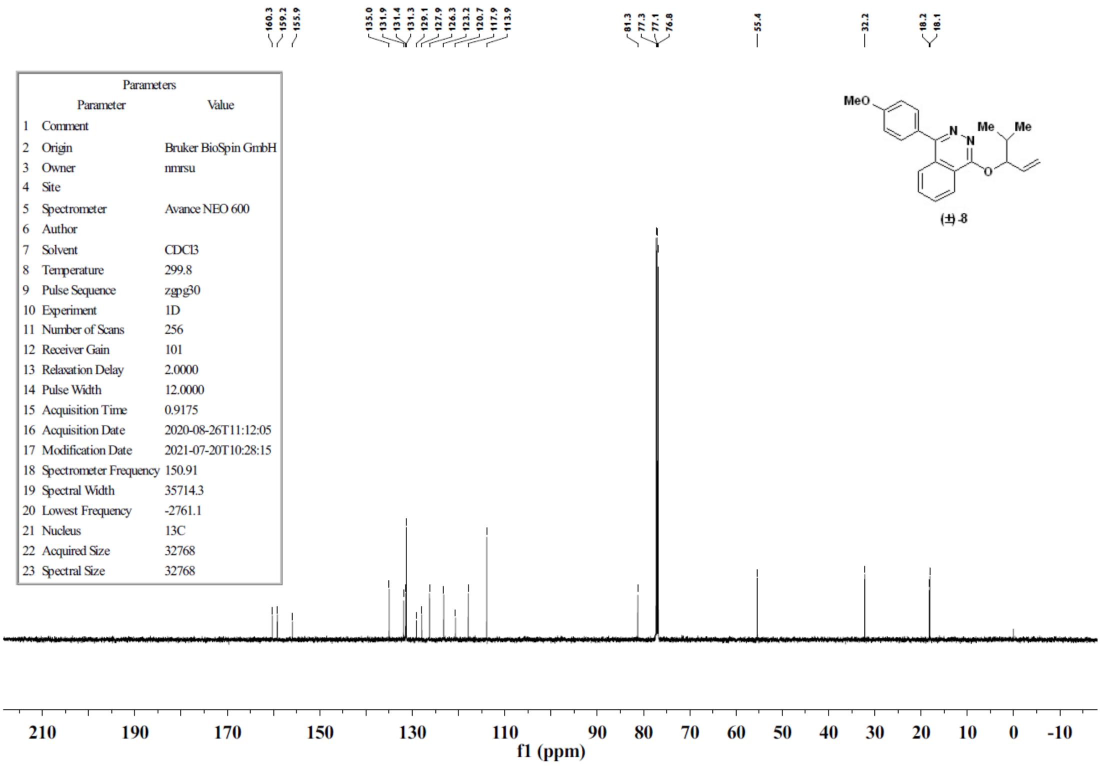

Supplementary Figure 218. ^{13}C NMR (101 MHz, $CDCl_3$) spectrum of compound **(±)-8**.

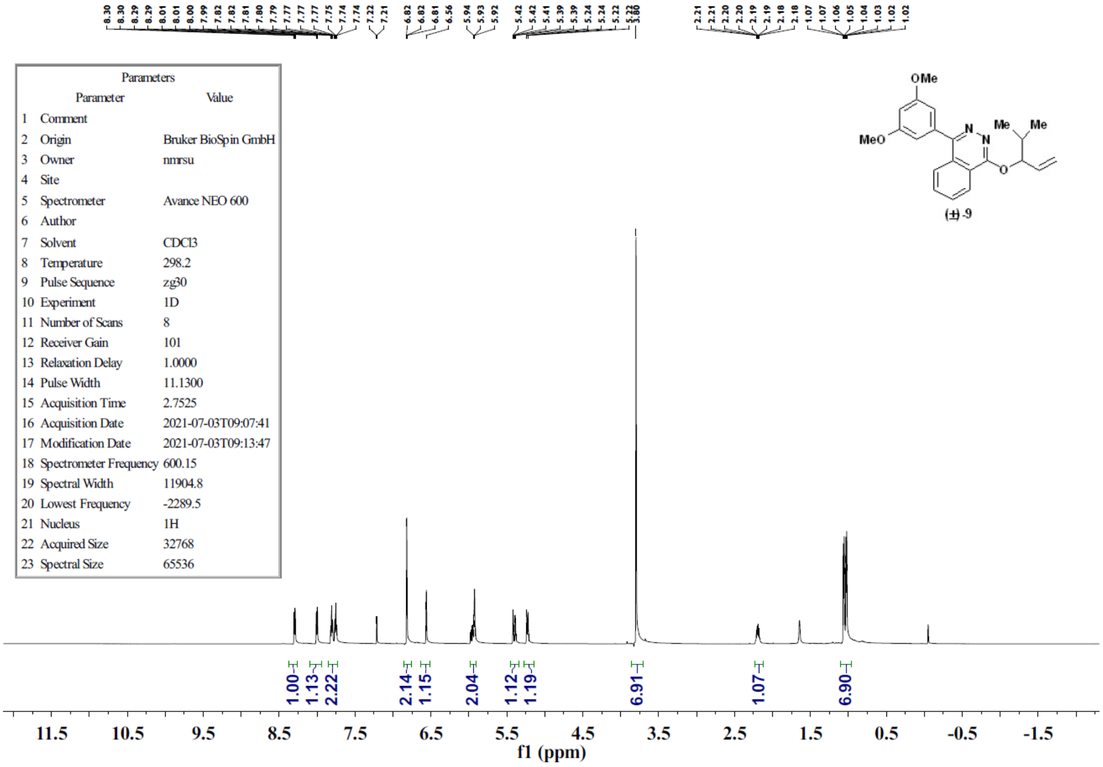

Supplementary Figure 219. ^{1}H NMR (600 MHz, $CDCl_3$) spectrum of compound **(±)-9**.

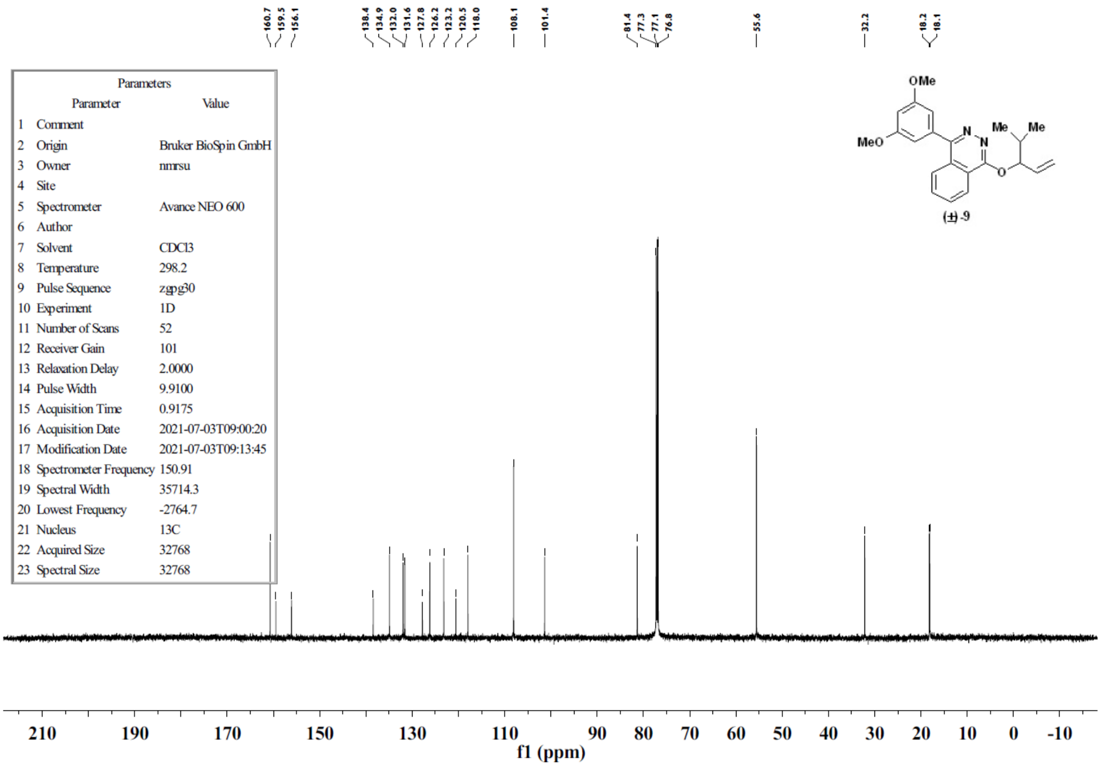

Supplementary Figure 220. ^{13}C NMR (151 MHz, $CDCl_3$) spectrum of compound **(±)-9**.

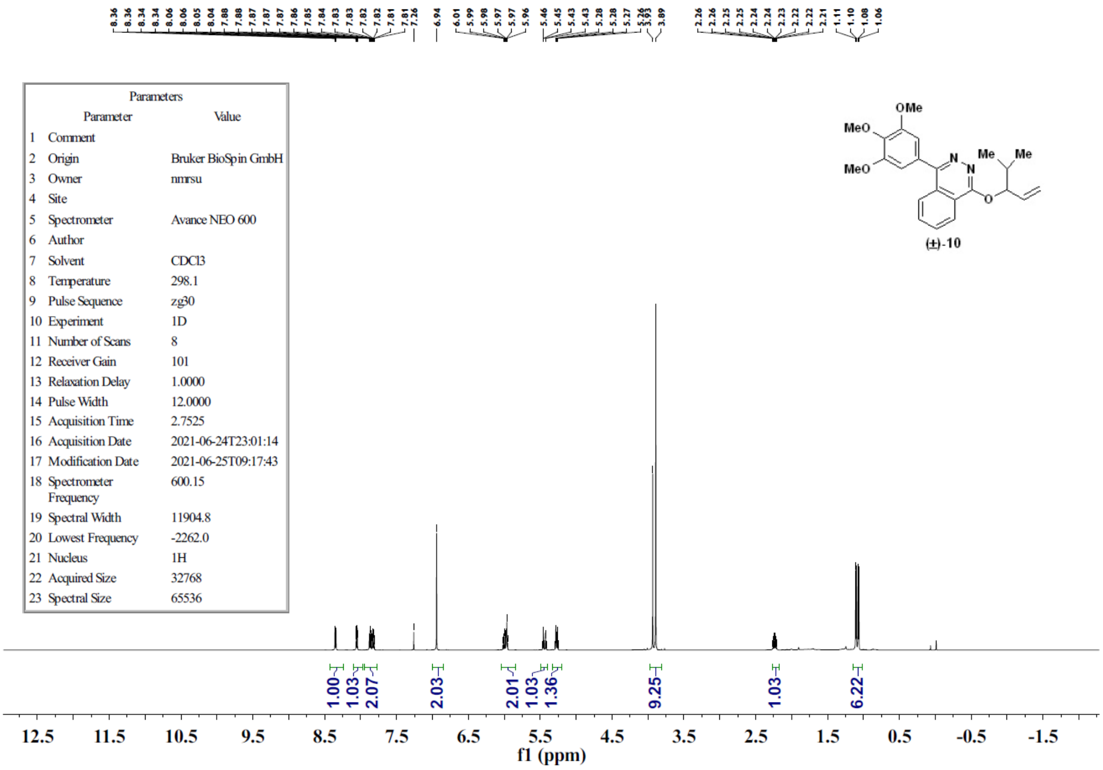

Supplementary Figure 221. ^{1}H NMR (600 MHz, $CDCl_3$) spectrum of compound **(±)-10**.

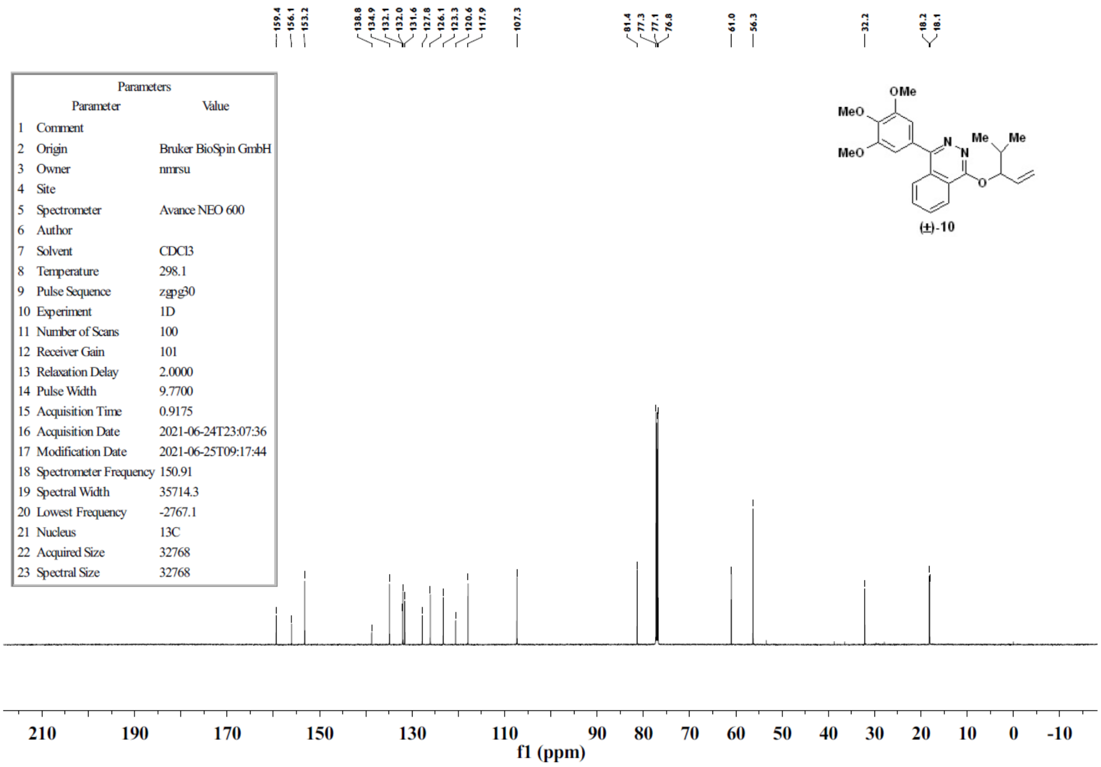

Supplementary Figure 222. ^{13}C NMR (151 MHz, $CDCl_3$) spectrum of compound **(±)-10**.

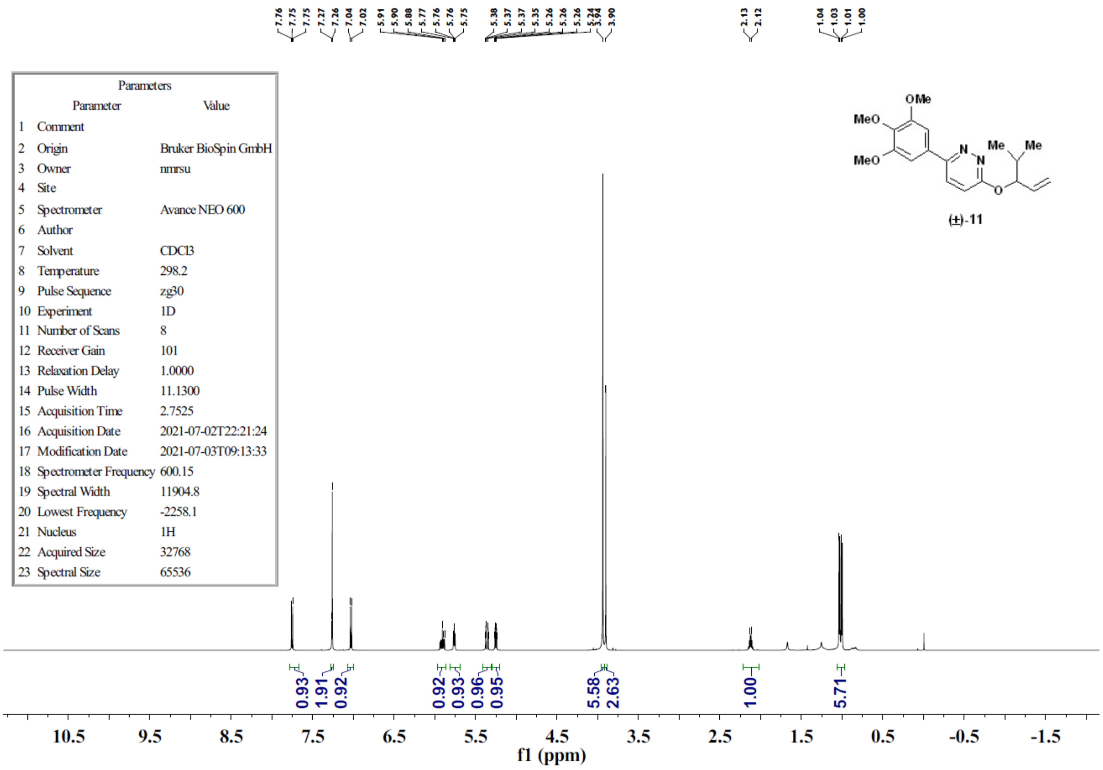

Supplementary Figure 223. ^{1}H NMR (600 MHz, $CDCl_3$) spectrum of compound **(±)-11**.

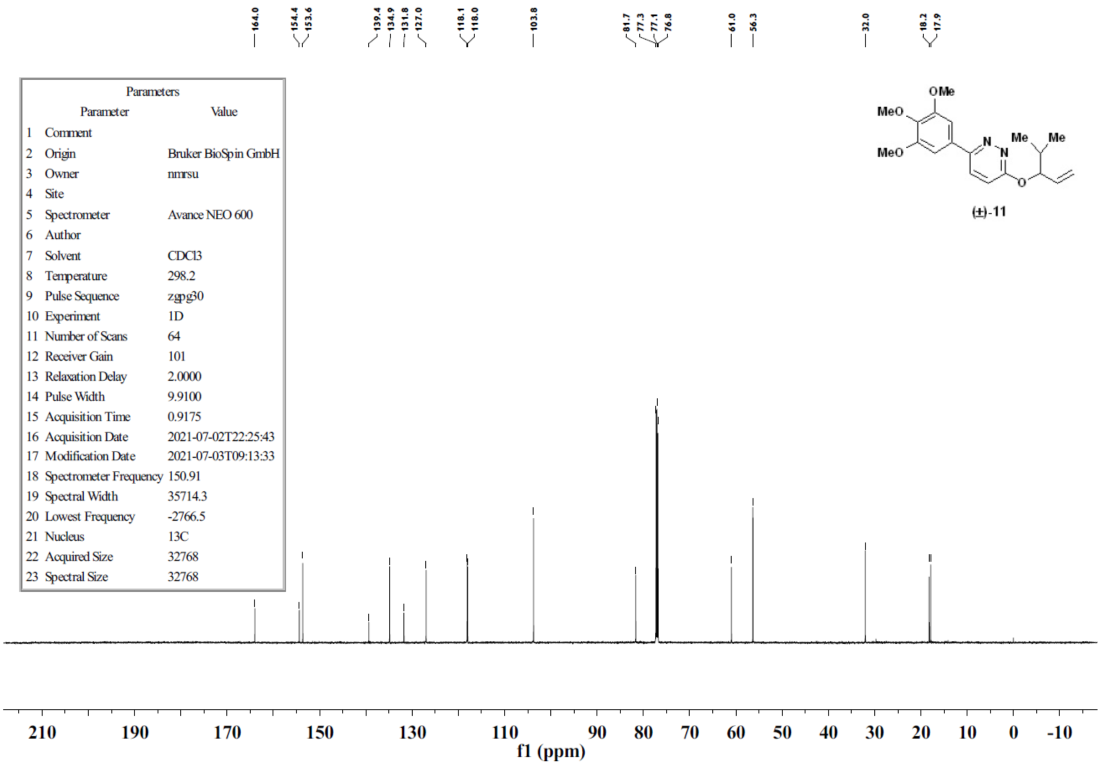

Supplementary Figure 224. ^{13}C NMR (151 MHz, $CDCl_3$) spectrum of compound **(±)-11**.

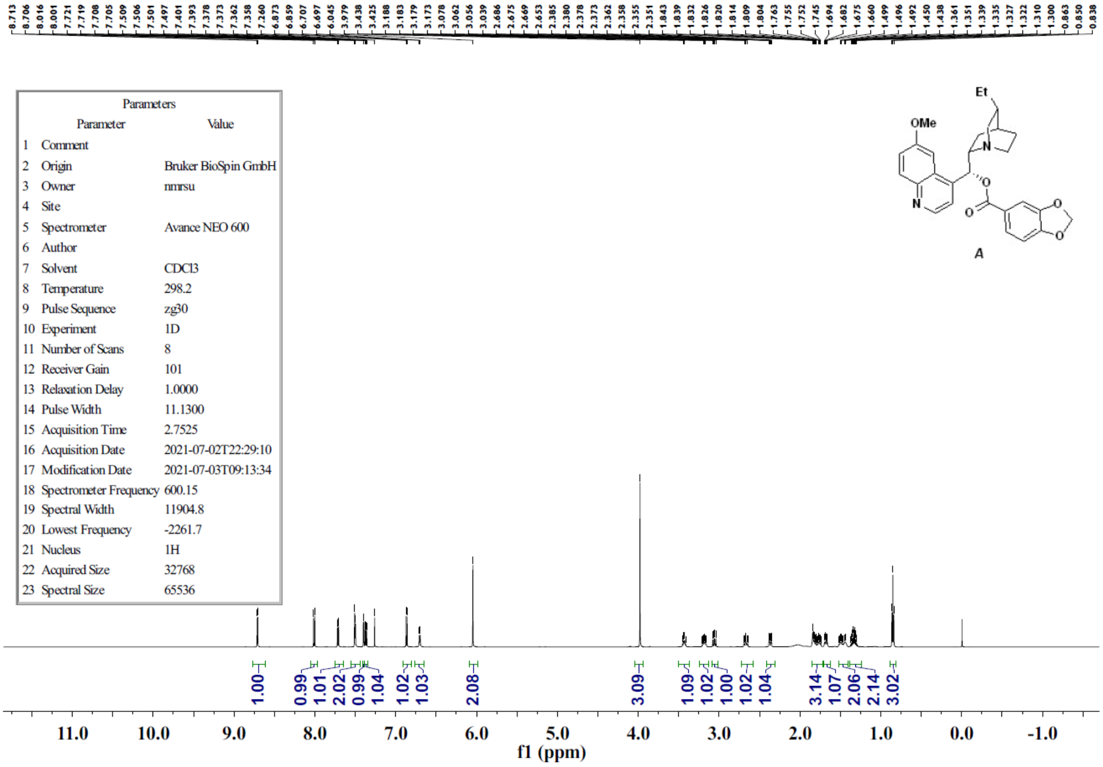

Supplementary Figure 225. ^{1}H NMR (600 MHz, $CDCl_3$) spectrum of ligand **A**.

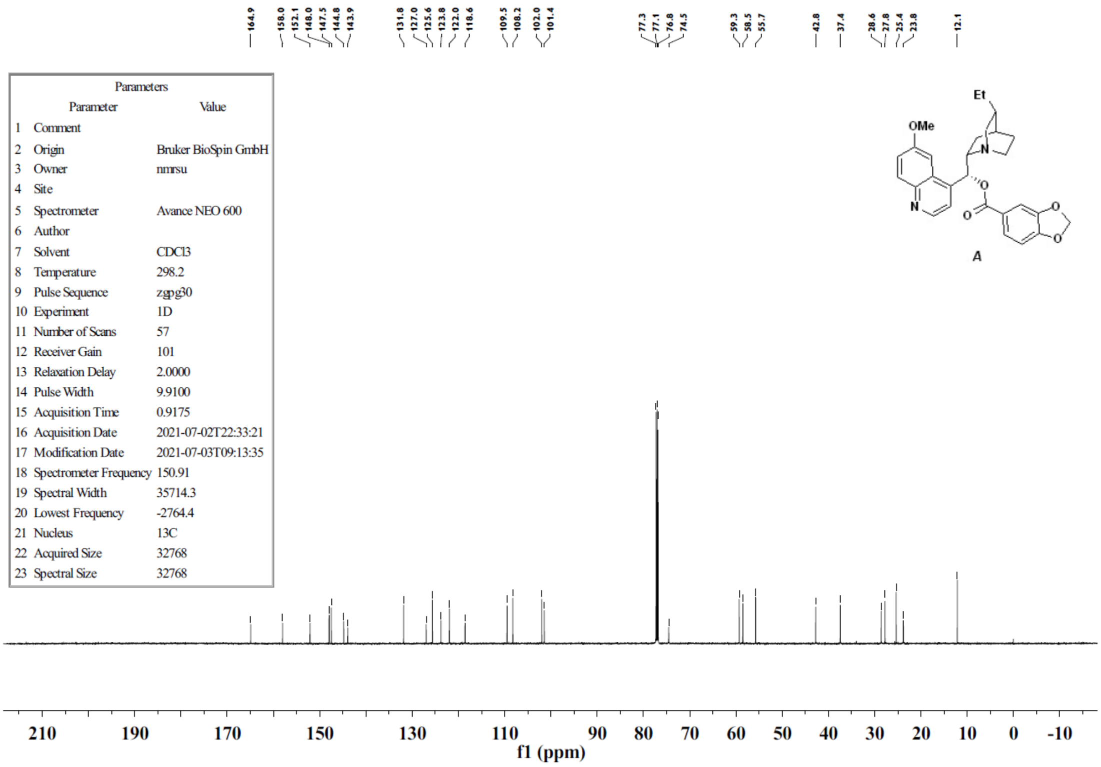

Supplementary Figure 226. ^{13}C NMR (151 MHz, $CDCl_3$) spectrum of ligand **A**.

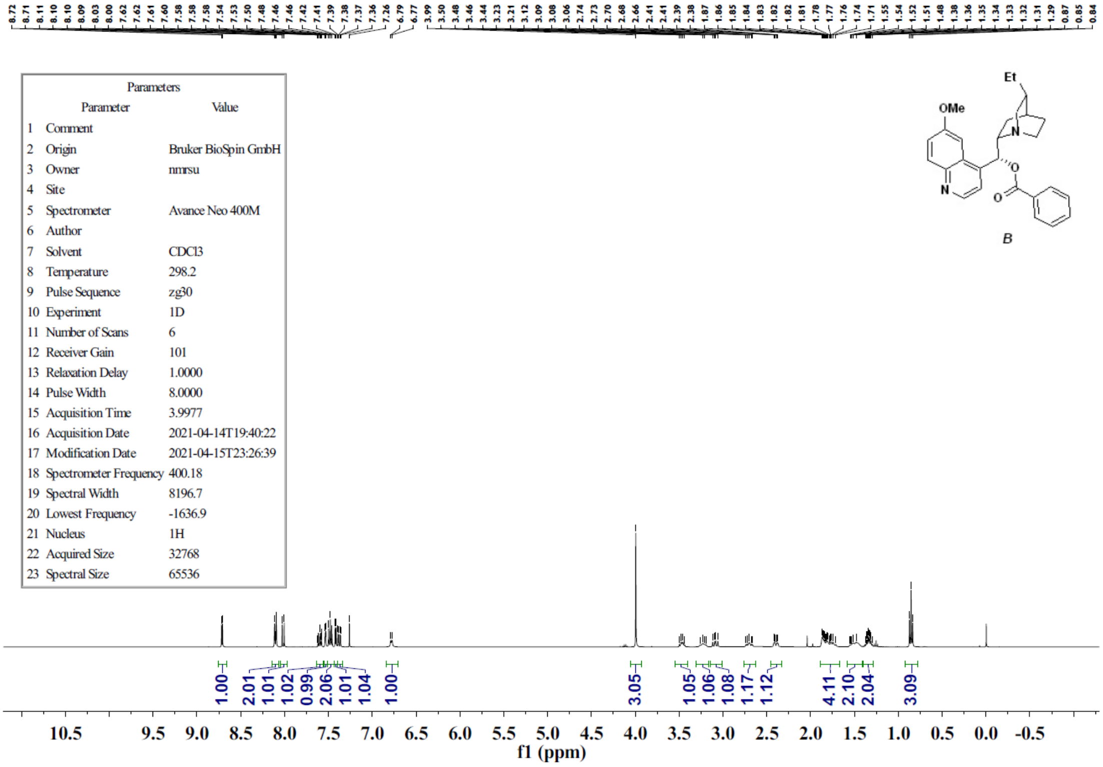

Supplementary Figure 225. ^{1}H NMR (100 MHz, $CDCl_3$) spectrum of ligand **B**.

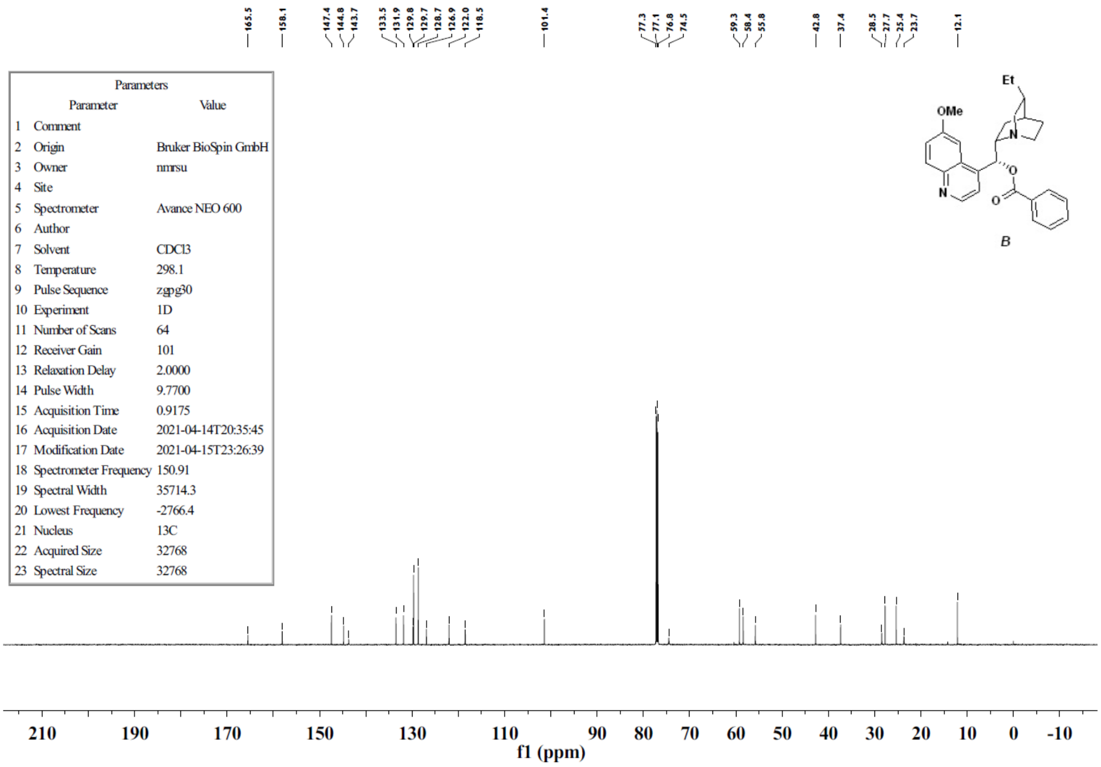

Supplementary Figure 226. ^{13}C NMR (151 MHz, $CDCl_3$) spectrum of ligand **B**.

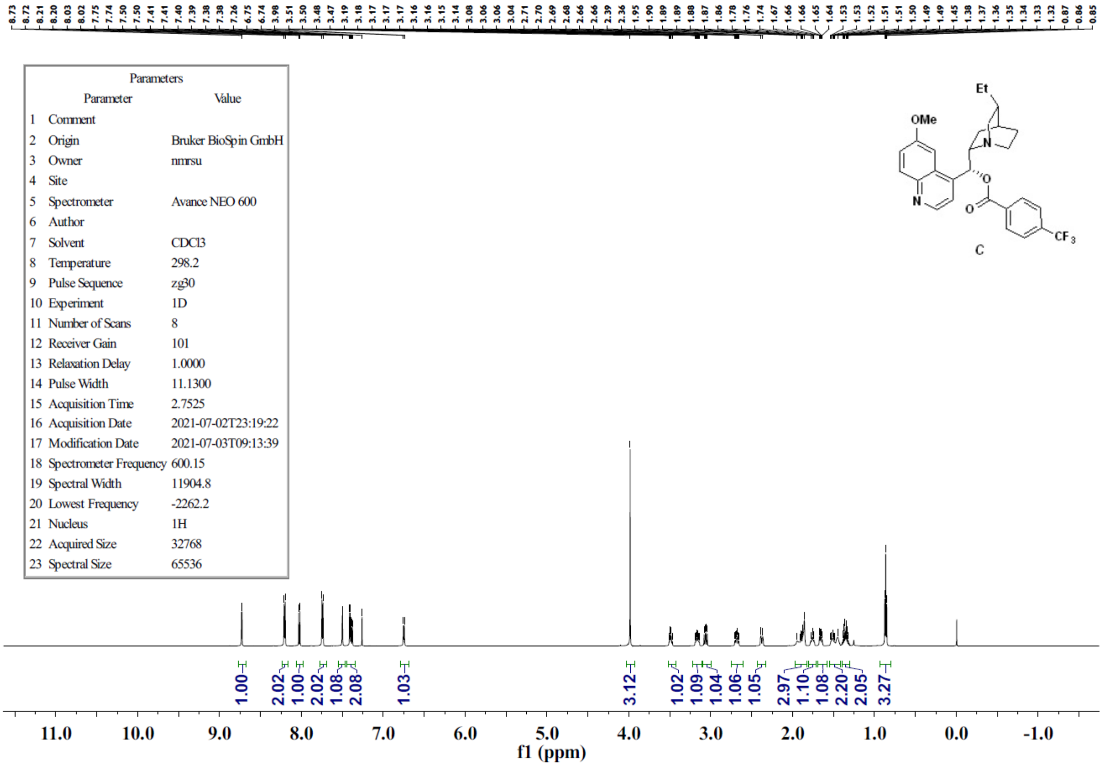

Supplementary Figure 227. ^{1}H NMR (600 MHz, $CDCl_3$) spectrum of ligand **C**.

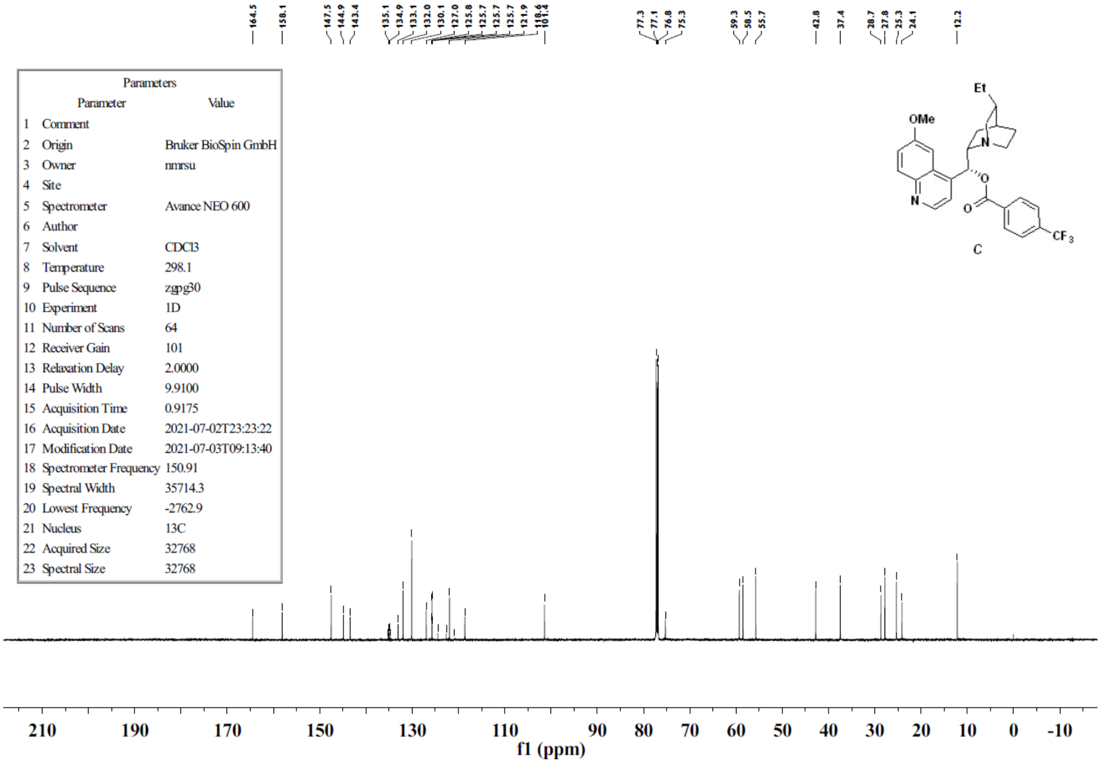

Supplementary Figure 228. ^{13}C NMR (151 MHz, $CDCl_3$) spectrum of ligand **C**.

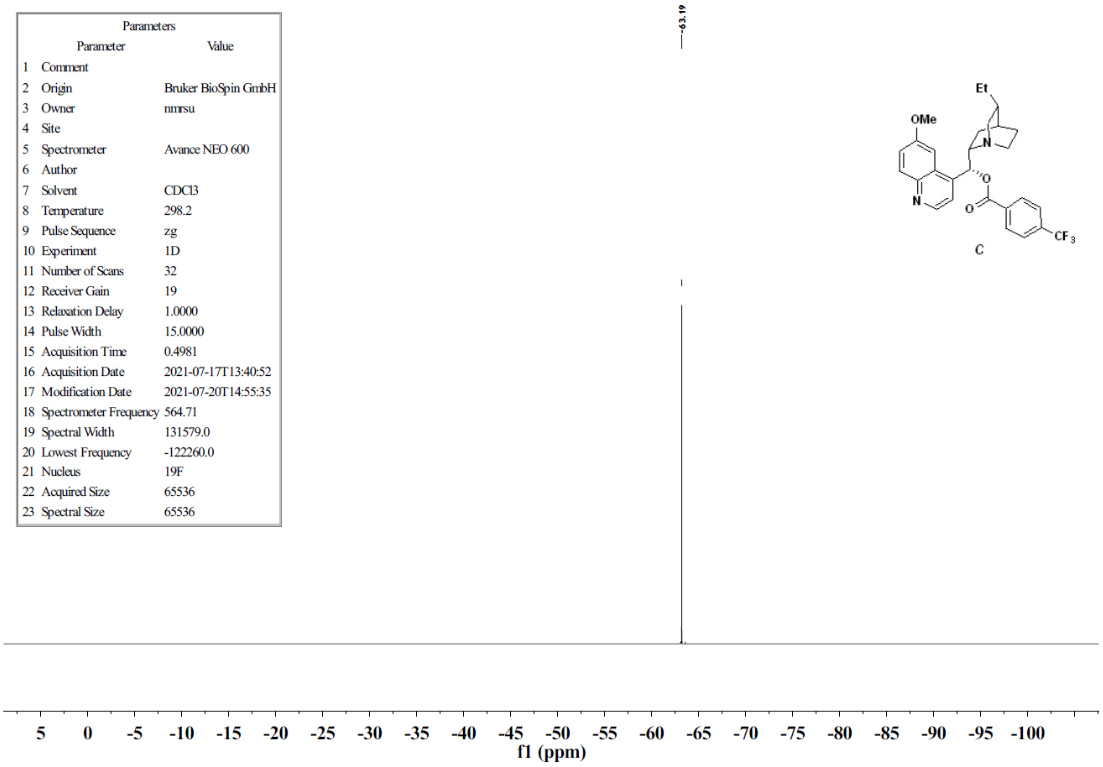

Supplementary Figure 229. ^{19}F NMR (376 MHz, $CDCl_3$) spectrum of ligand **C**.

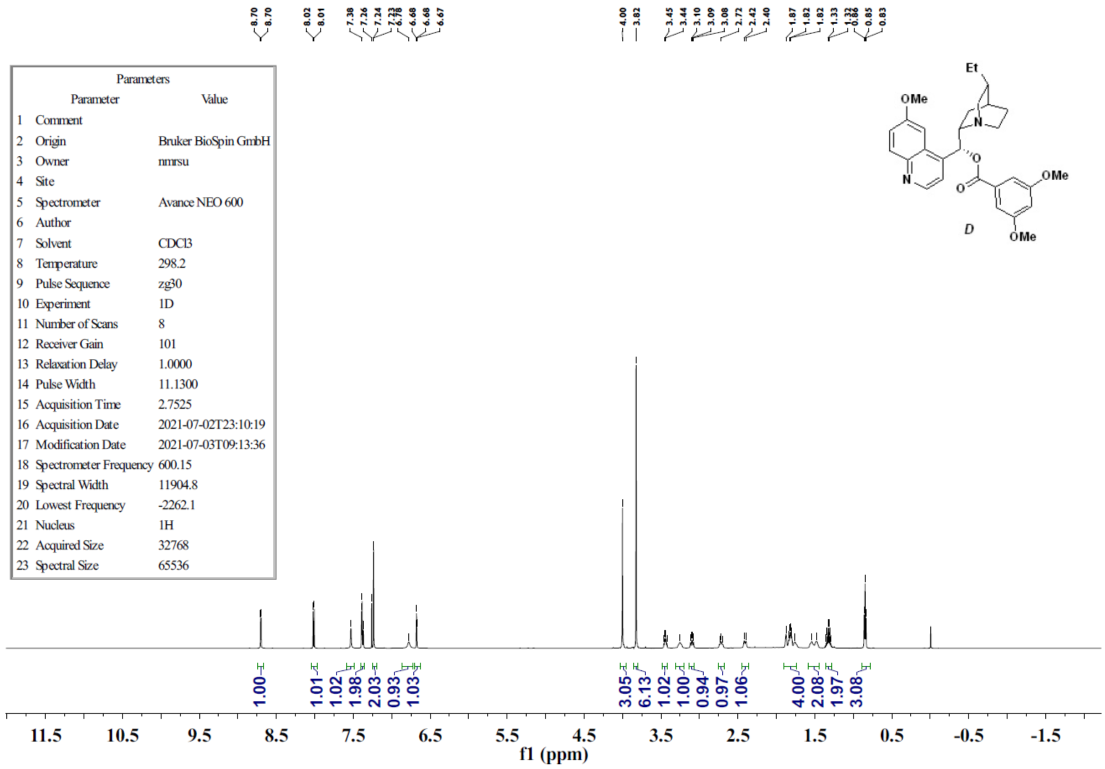

Supplementary Figure 230. ^{1}H NMR (600 MHz, $CDCl_3$) spectrum of ligand **D**.

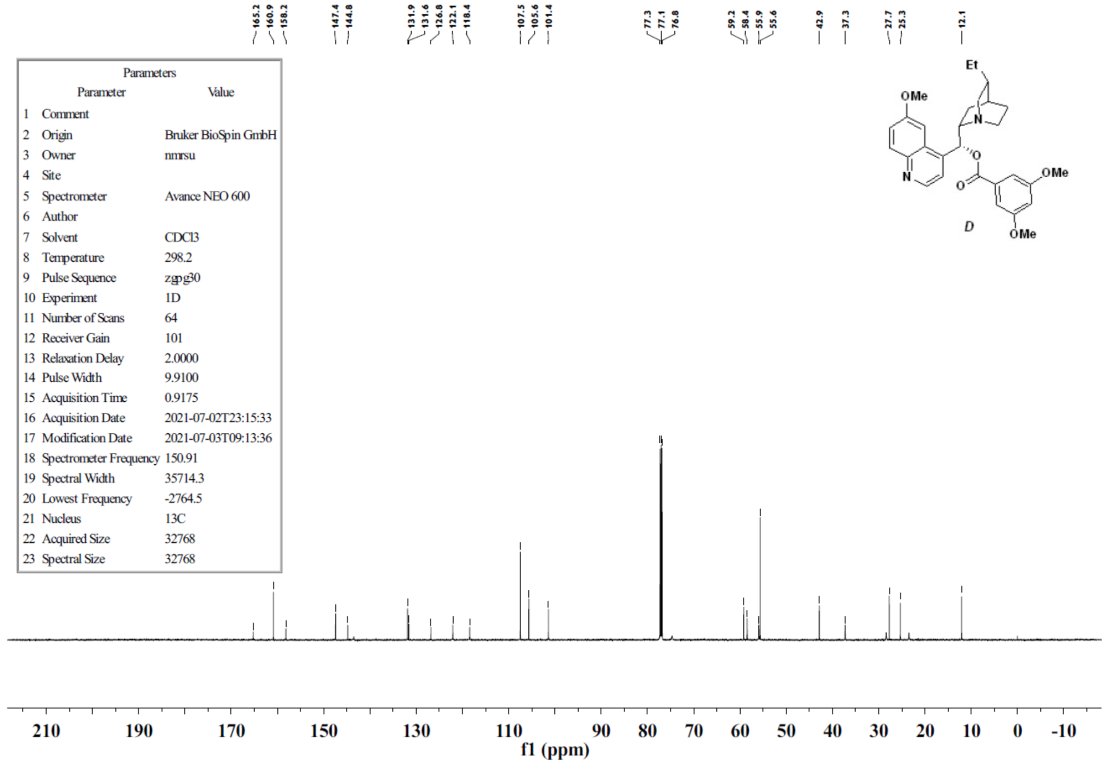

Supplementary Figure 231. ^{13}C NMR (151 MHz, $CDCl_3$) spectrum of ligand **D**.

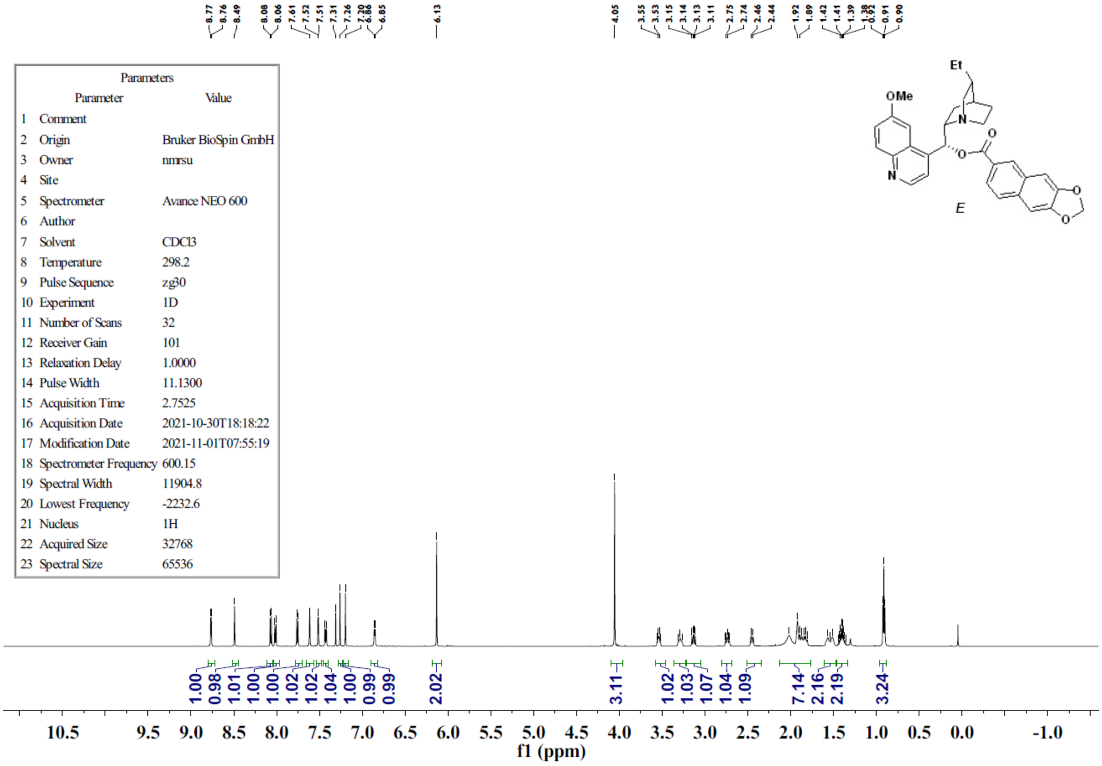

Supplementary Figure 232. ^{1}H NMR (600 MHz, $CDCl_3$) spectrum of ligand **E**.

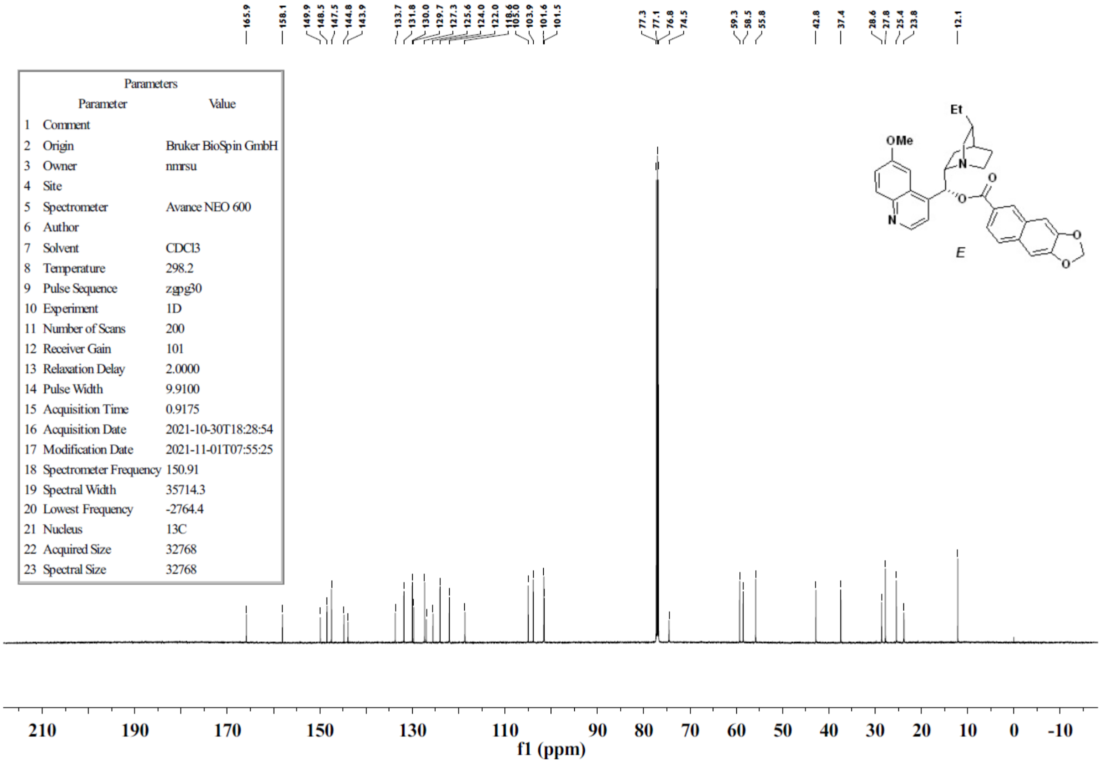

Supplementary Figure 233. ^{13}C NMR (151 MHz, $CDCl_3$) spectrum of ligand **E**.

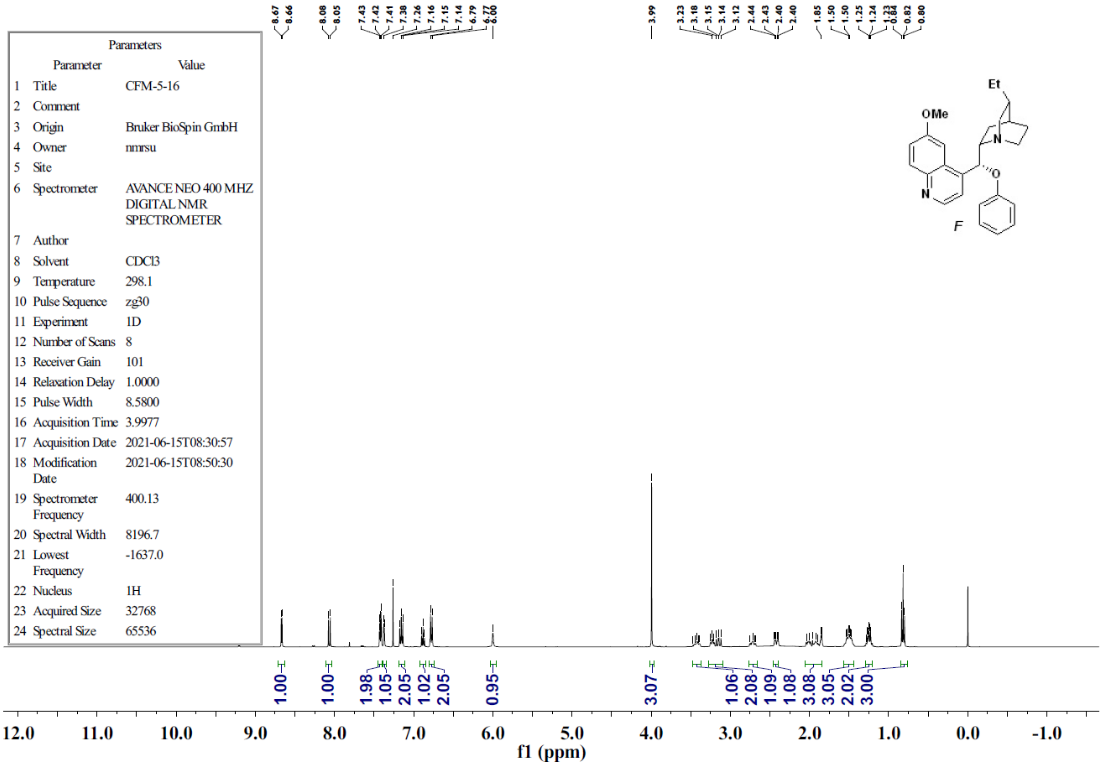

Supplementary Figure 234. ^{1}H NMR (400 MHz, $CDCl_3$) spectrum of ligand **F**.

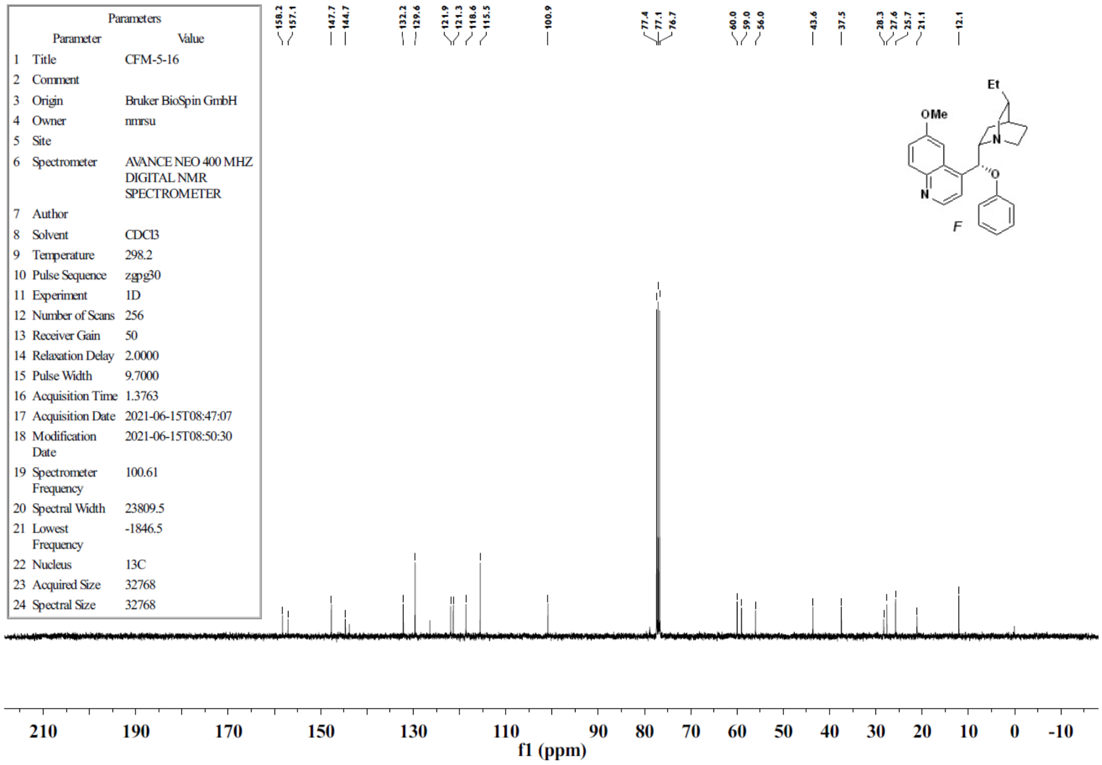

Supplementary Figure 235. ^{13}C NMR (101 MHz, $CDCl_3$) spectrum of ligand **F**.

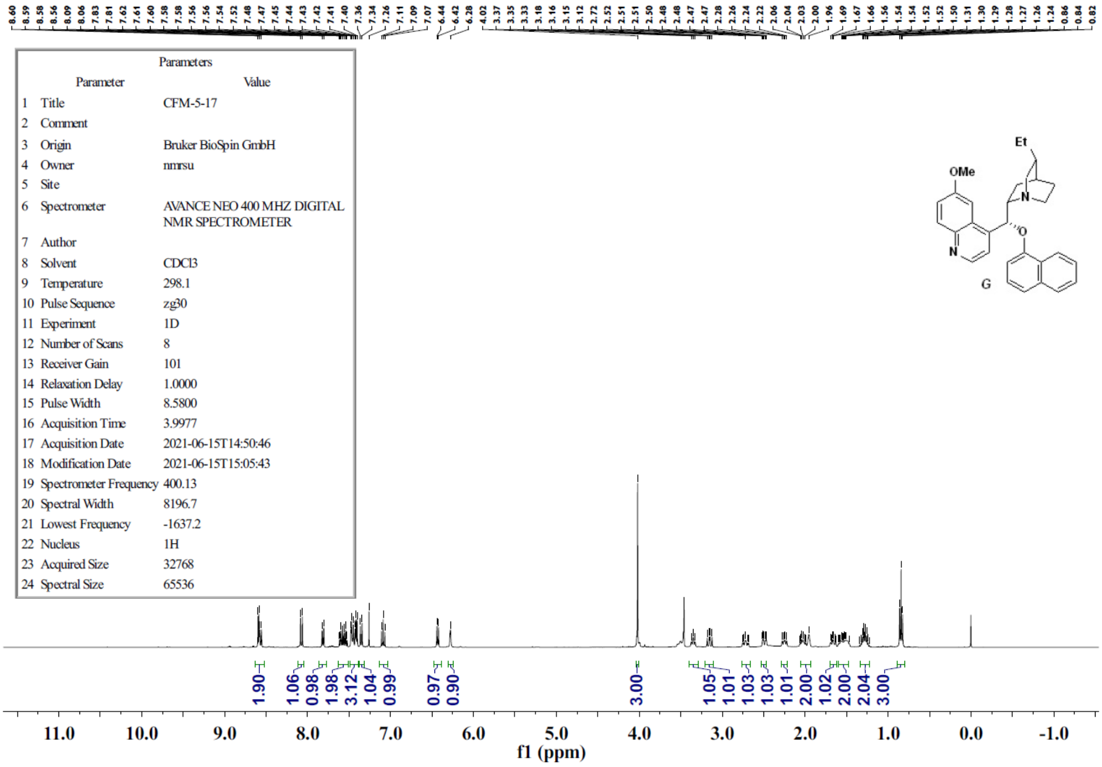

Supplementary Figure 236. ^{1}H NMR (400 MHz, $CDCl_3$) spectrum of ligand **G**.

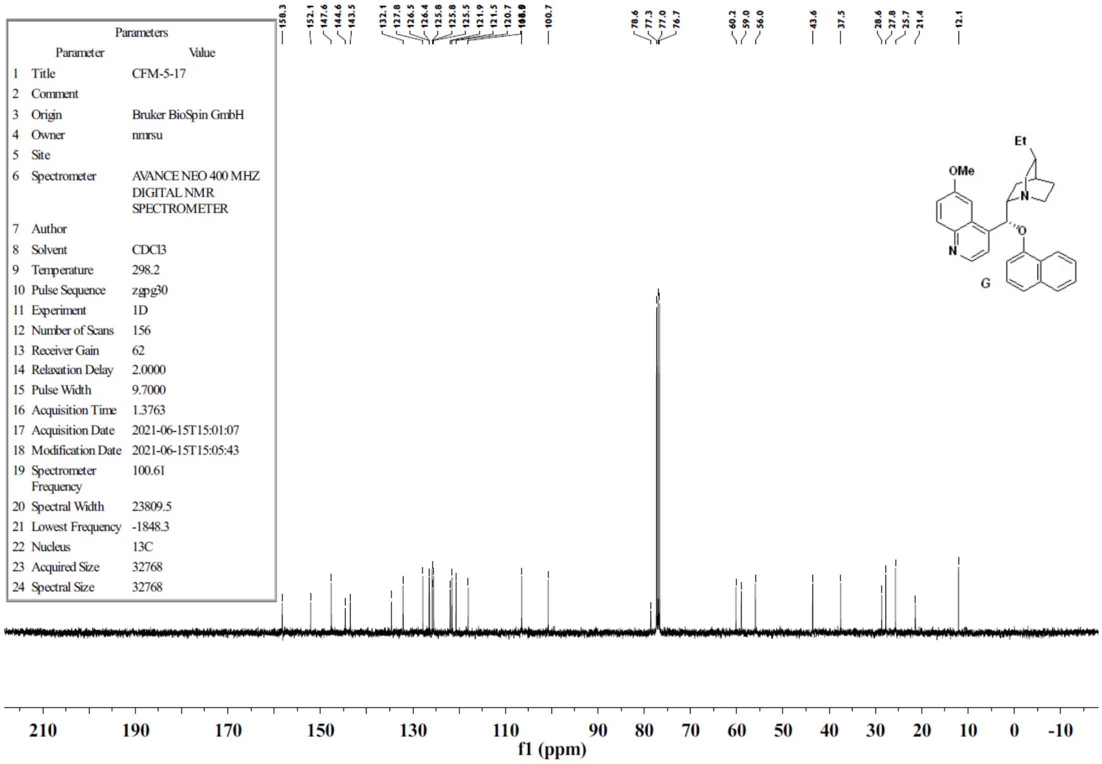

Supplementary Figure 237. ^{13}C NMR (101 MHz, $CDCl_3$) spectrum of ligand **G**.

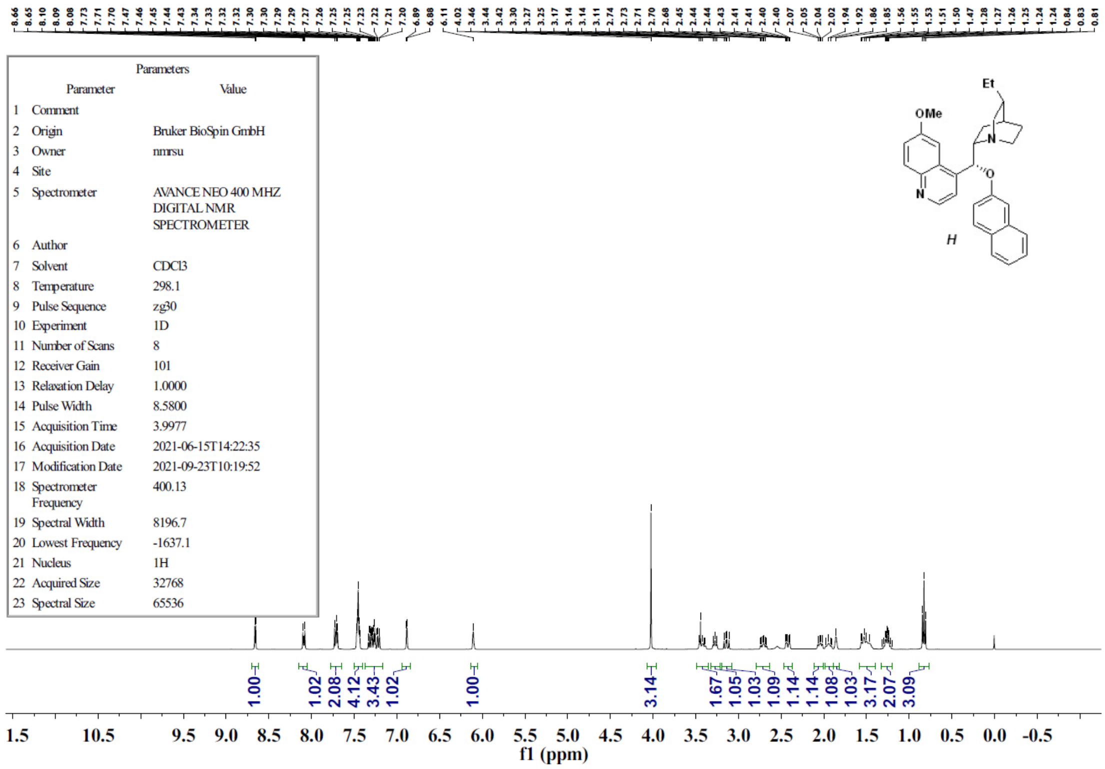

Supplementary Figure 238. ^{1}H NMR (400 MHz, $CDCl_3$) spectrum of ligand **H**.

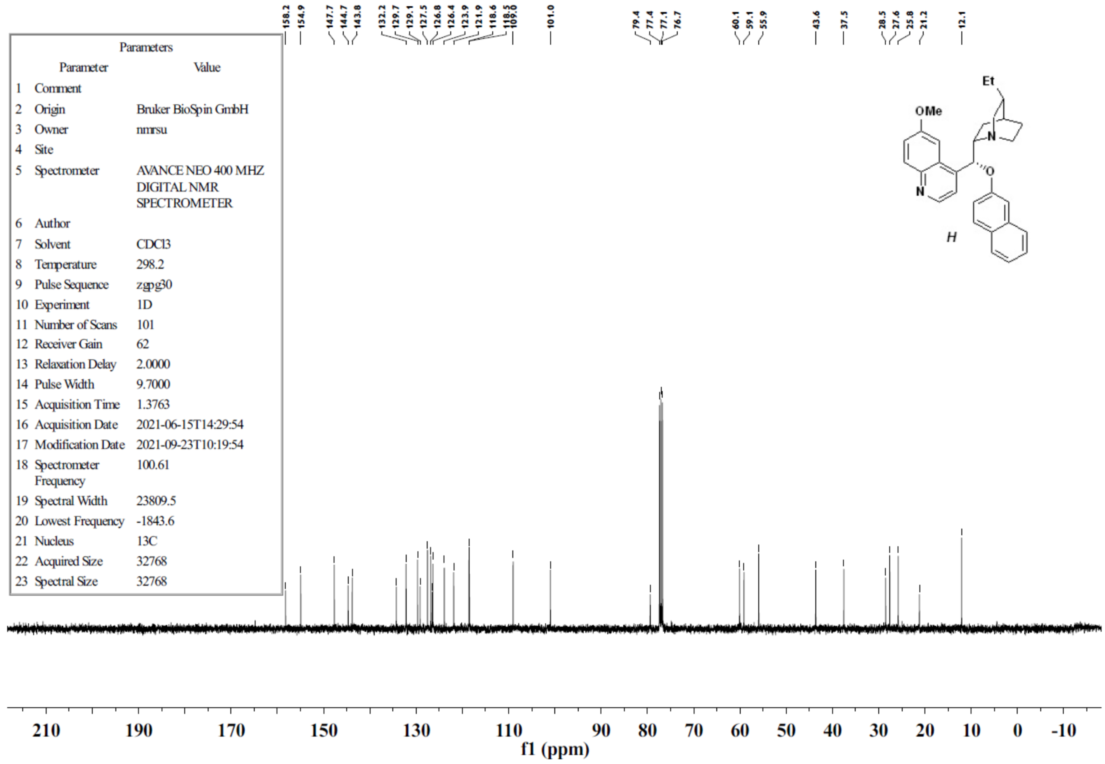

Supplementary Figure 239. ^{13}C NMR (101 MHz, $CDCl_3$) spectrum of ligand **H**.

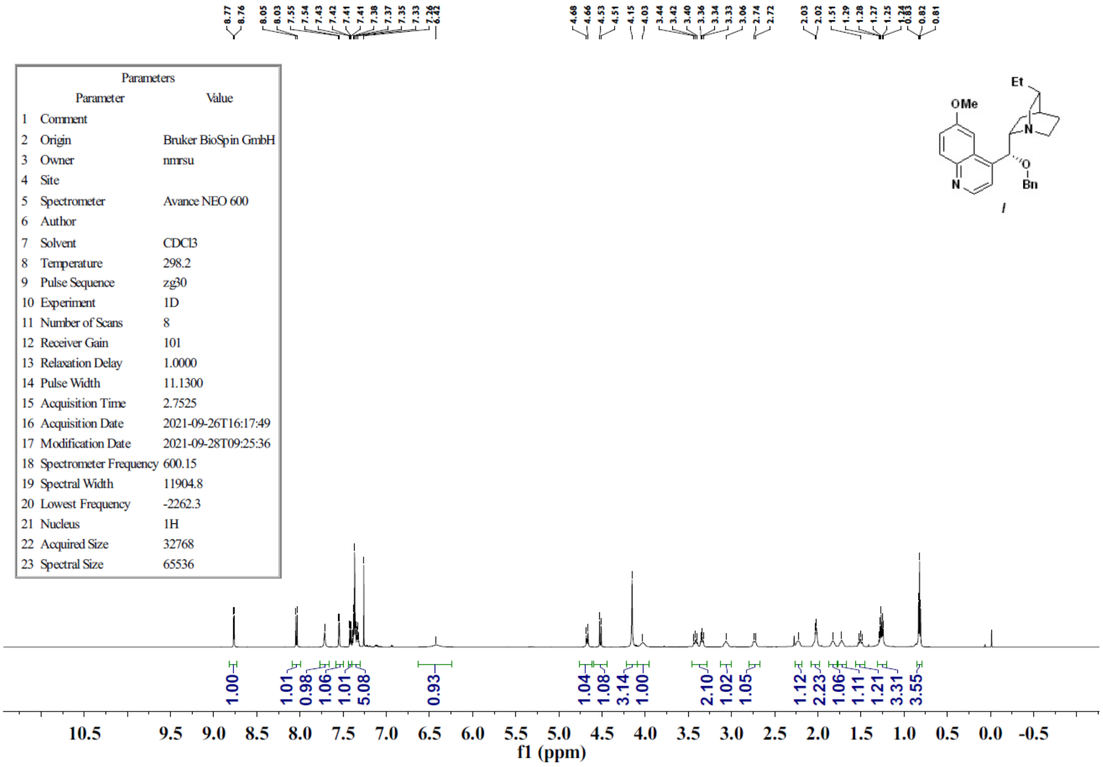

Supplementary Figure 240. ^{1}H NMR (600 MHz, $CDCl_3$) spectrum of ligand **I**.

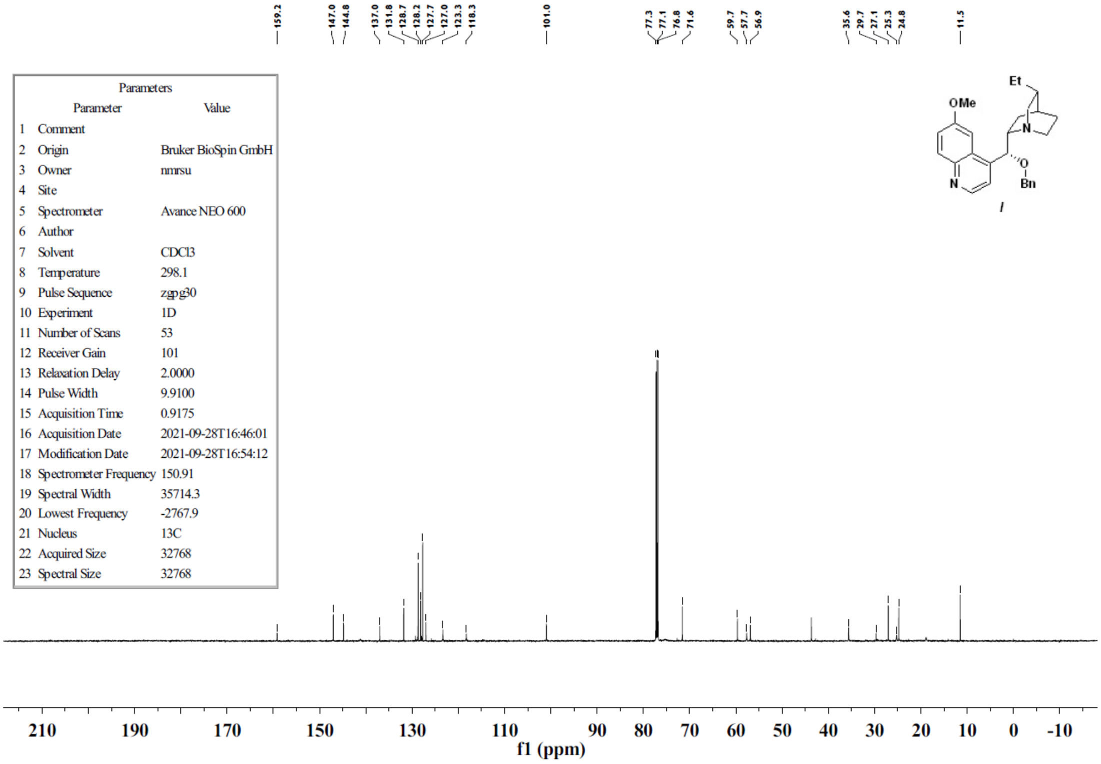

Supplementary Figure 241. ^{13}C NMR (151 MHz, $CDCl_3$) spectrum of ligand **I**.

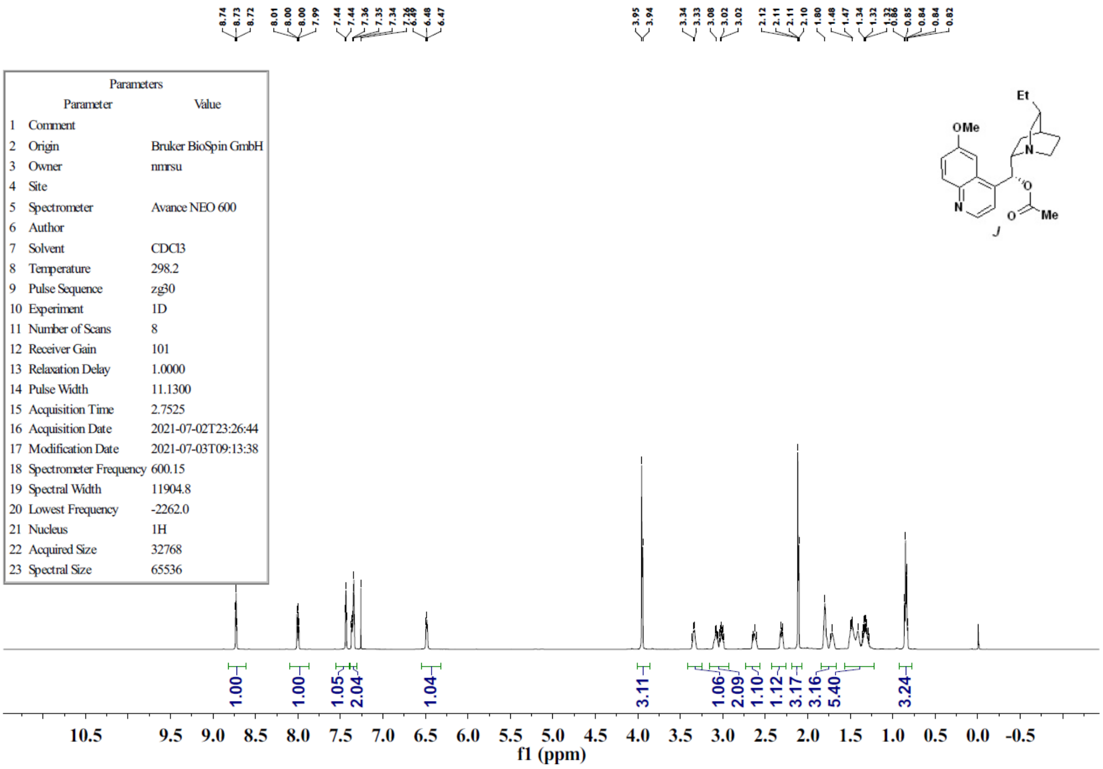

Supplementary Figure 242. ^{1}H NMR (600 MHz, $CDCl_3$) spectrum of ligand **J**.

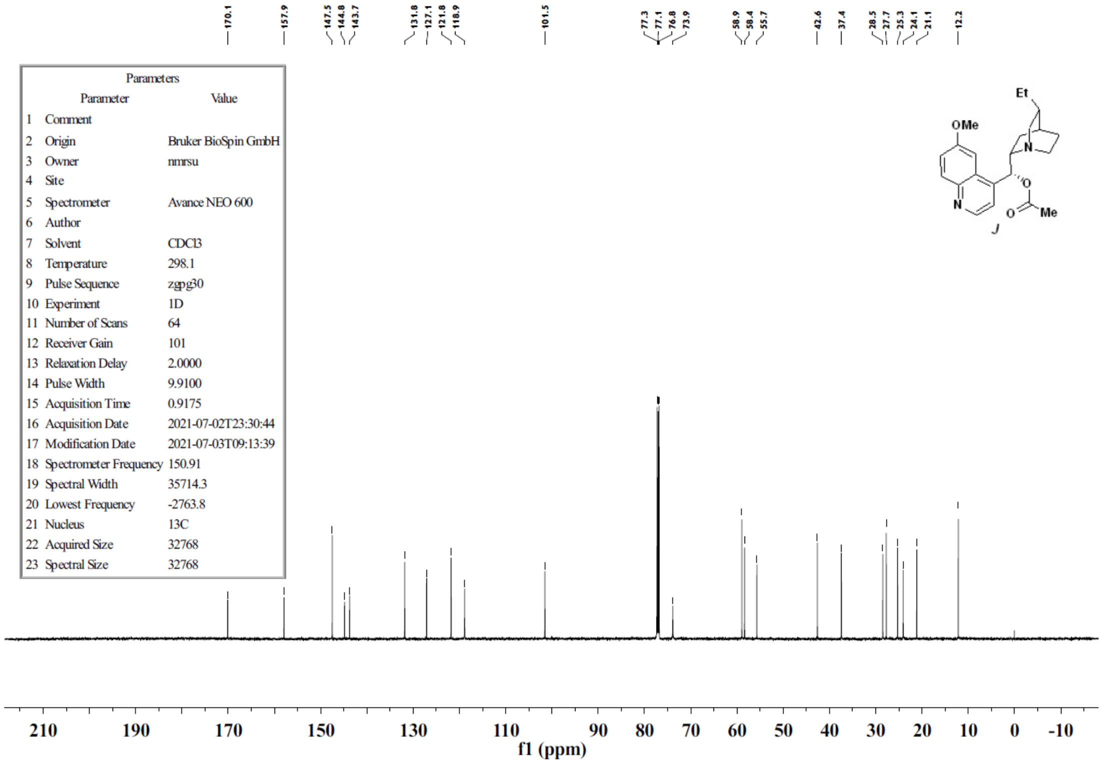

Supplementary Figure 243. ^{13}C NMR (151 MHz, $CDCl_3$) spectrum of ligand **J**.

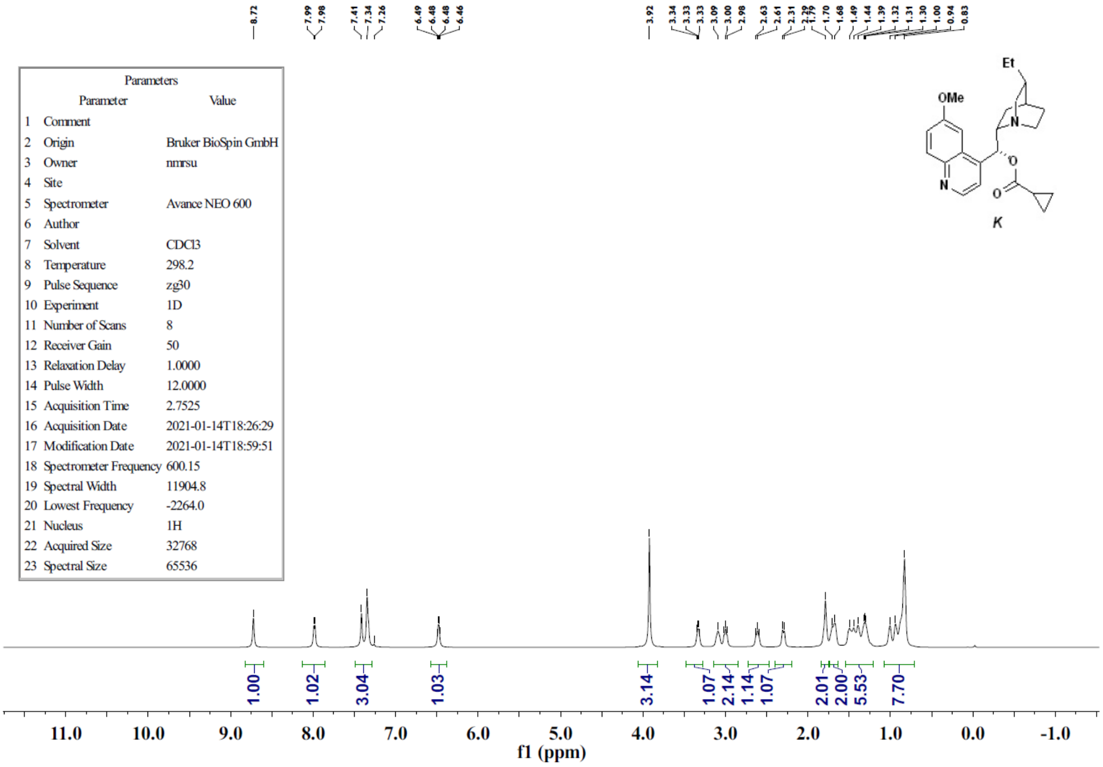

Supplementary Figure 244. ^{1}H NMR (600 MHz, $CDCl_3$) spectrum of ligand **K**.

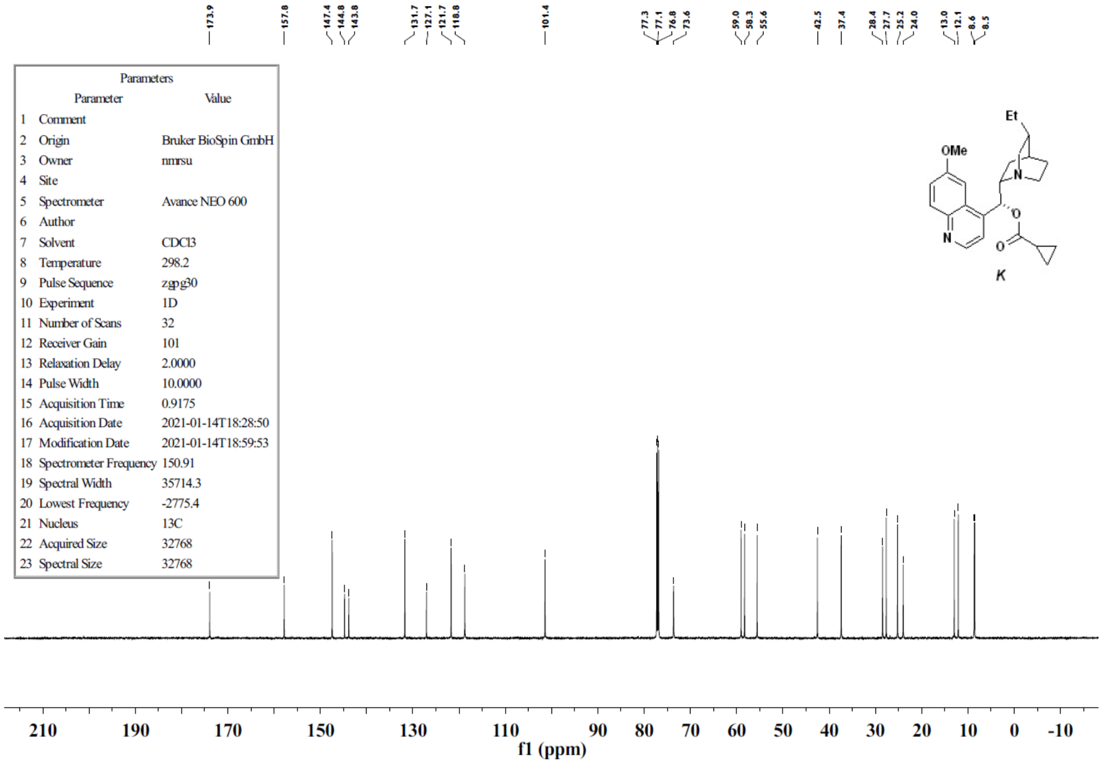

Supplementary Figure 245. ^{13}C NMR (151 MHz, $CDCl_3$) spectrum of ligand **K**.

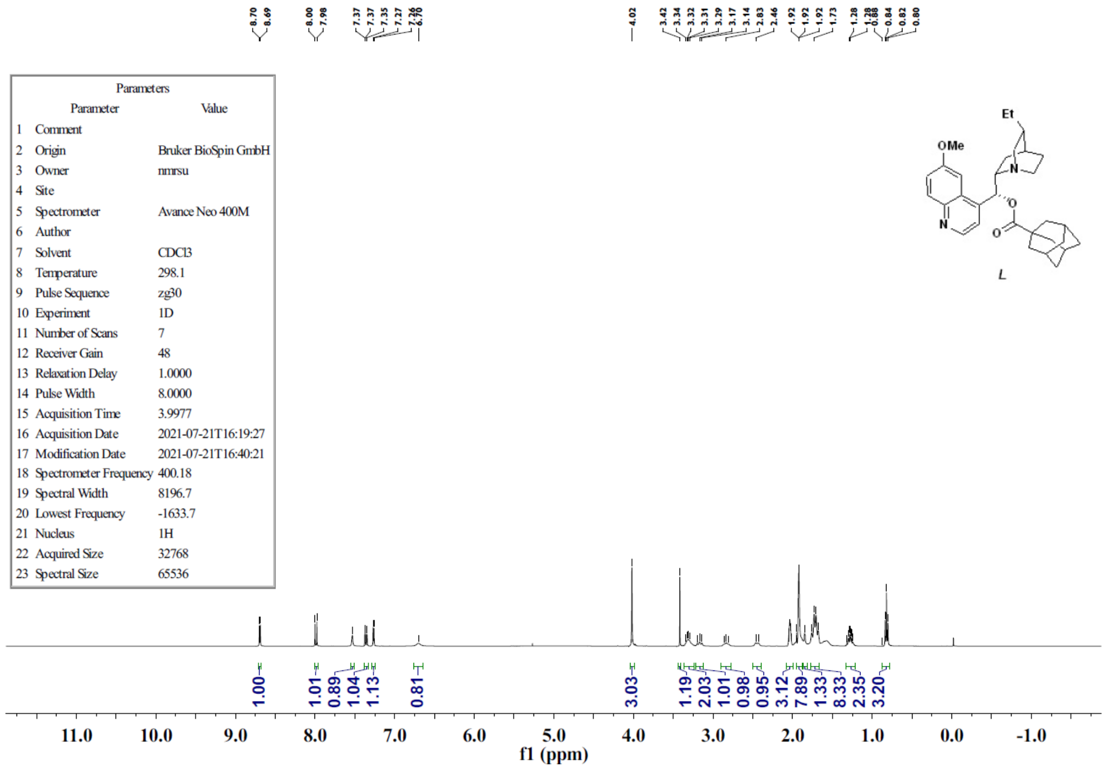

Supplementary Figure 246. ^{1}H NMR (400 MHz, $CDCl_3$) spectrum of ligand **L**.

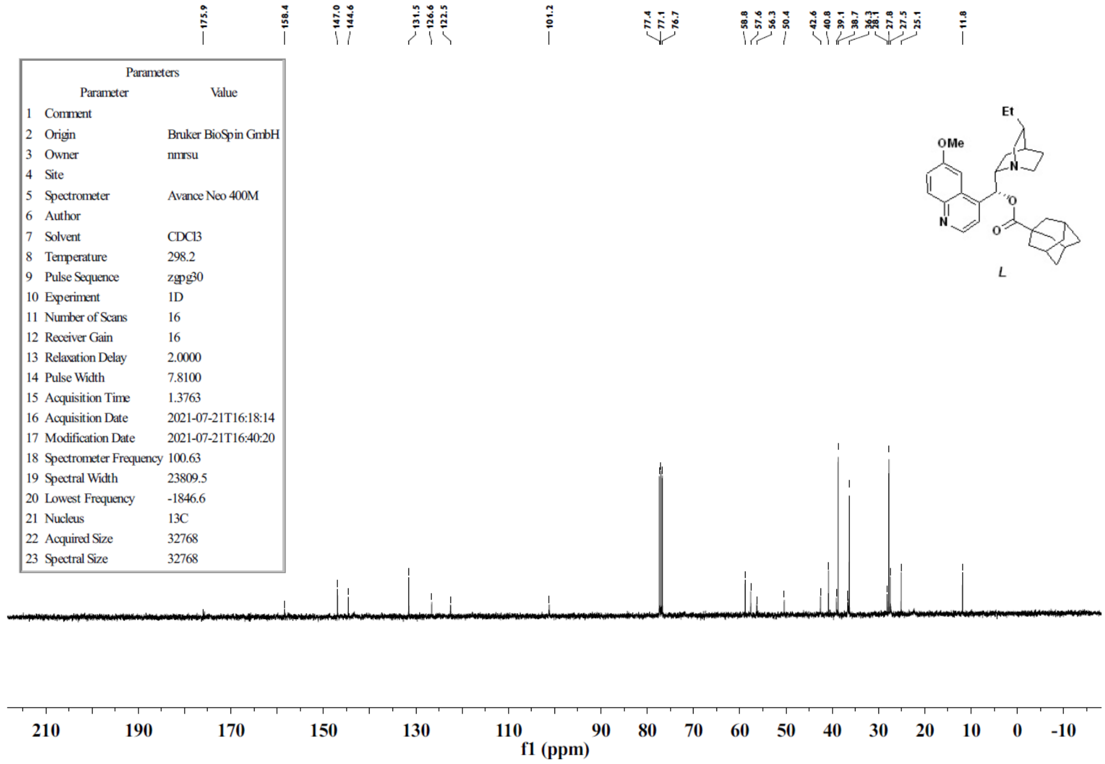

Supplementary Figure 247. ^{13}C NMR (101 MHz, $CDCl_3$) spectrum of ligand **L**.

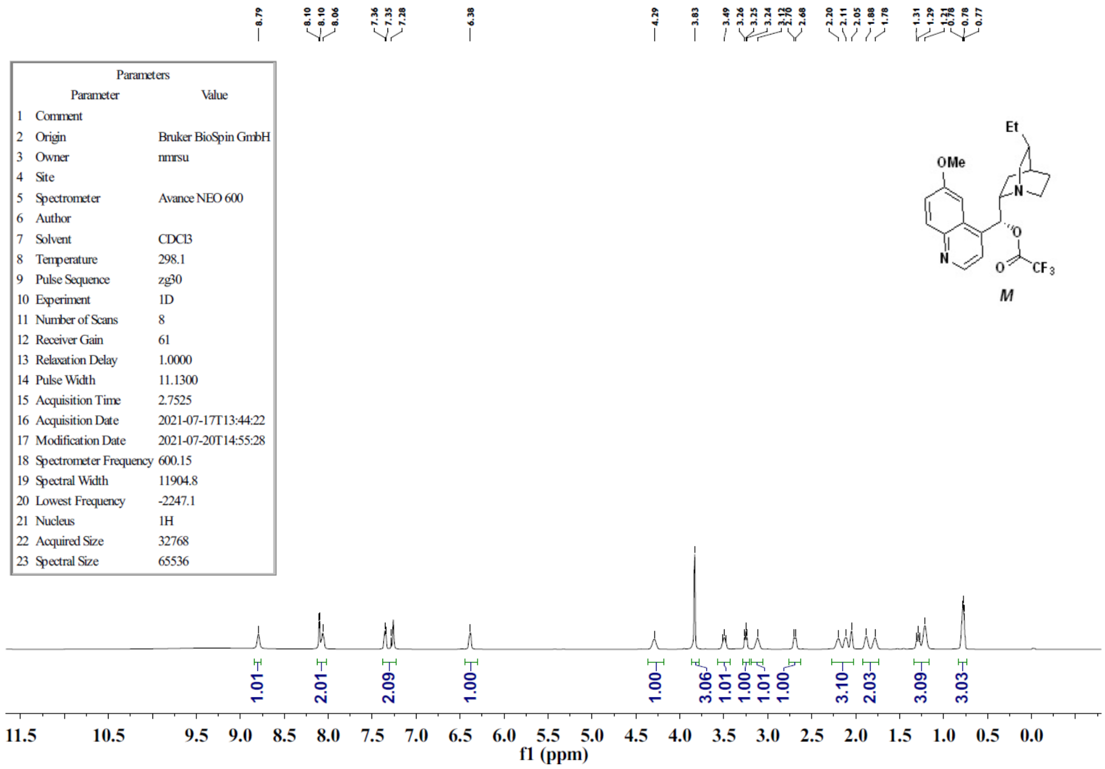

Supplementary Figure 248. ^{1}H NMR (600 MHz, $CDCl_3$) spectrum of ligand **M**.

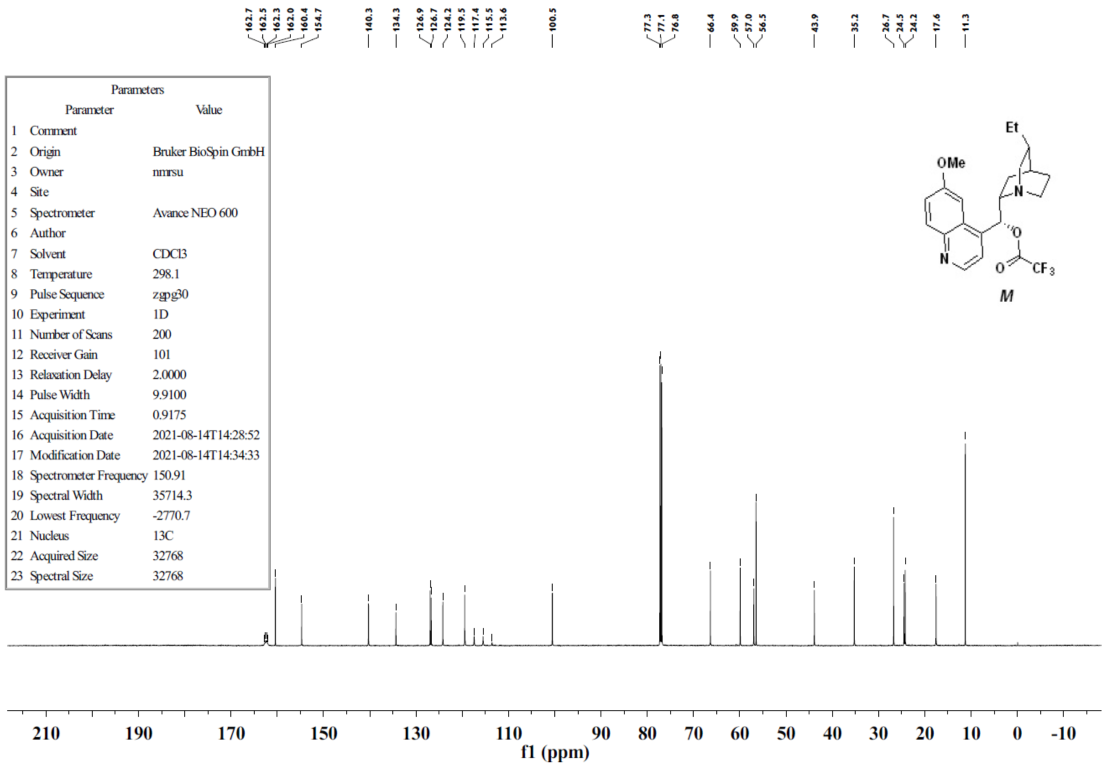

Supplementary Figure 249. ^{13}C NMR (151 MHz, $CDCl_3$) spectrum of ligand **M**.

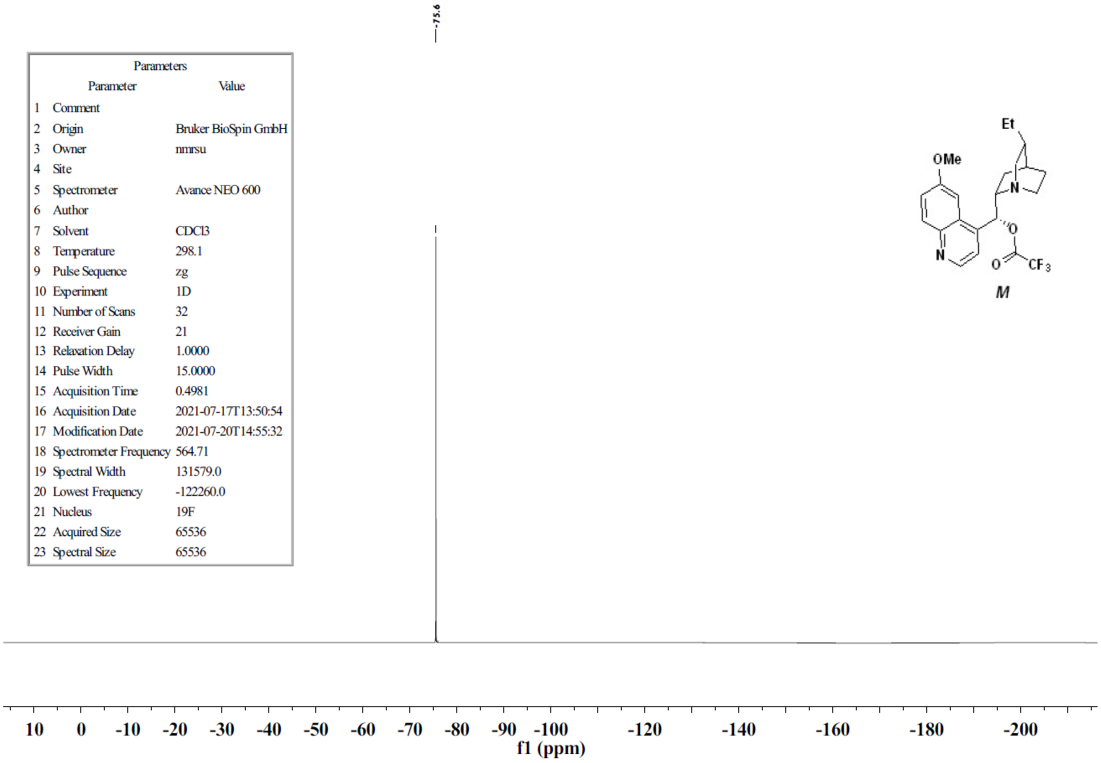

Supplementary Figure 250. ^{19}F NMR (376 MHz, $CDCl_3$) spectrum of ligand **M**.

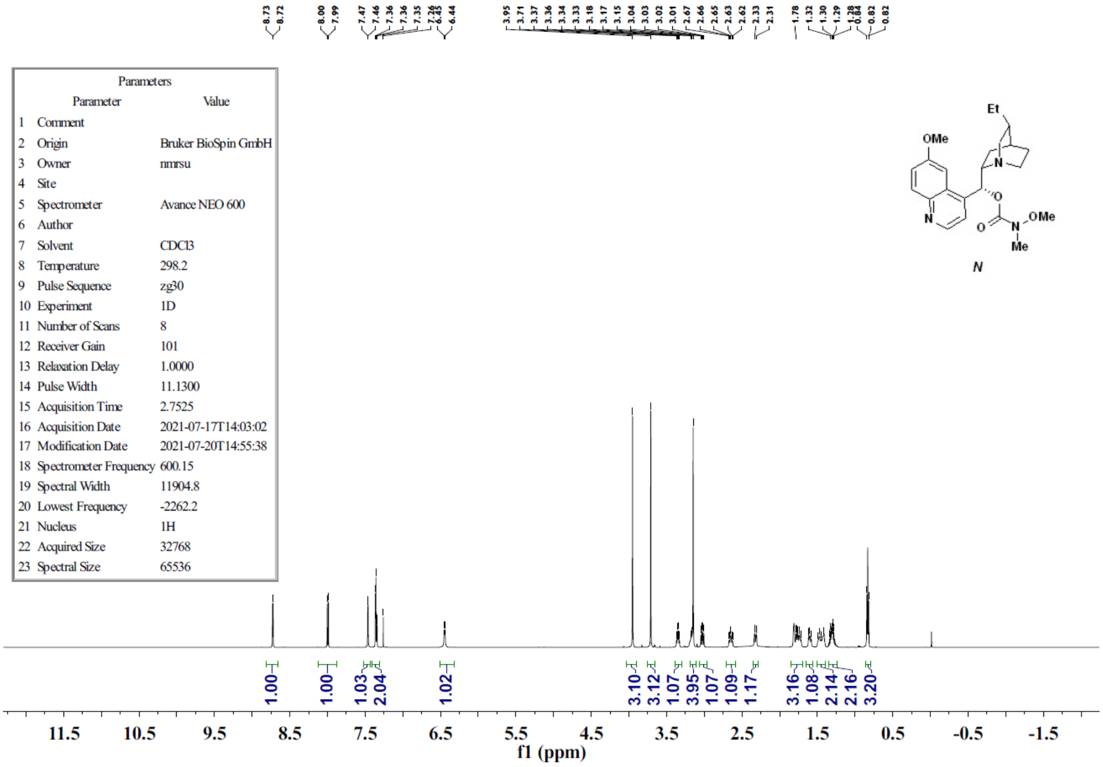

Supplementary Figure 251. ^{1}H NMR (600 MHz, $CDCl_3$) spectrum of ligand **N**.

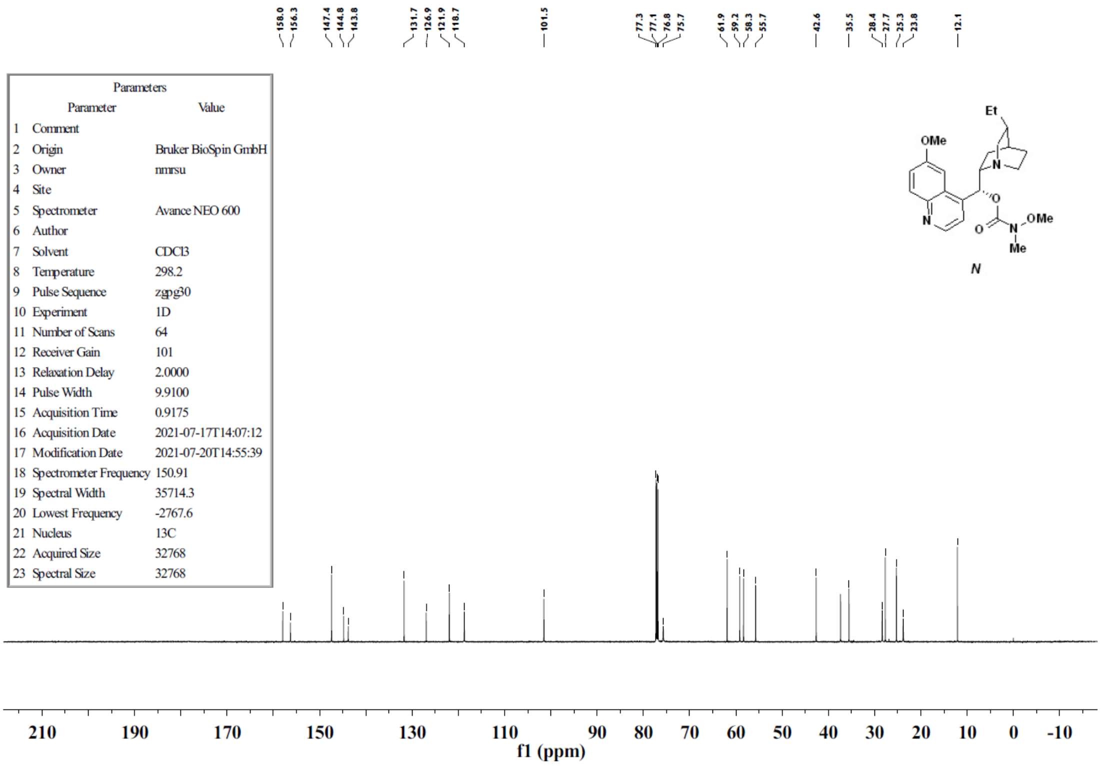

Supplementary Figure 252. ^{13}C NMR (151 MHz, $CDCl_3$) spectrum of ligand **N**.

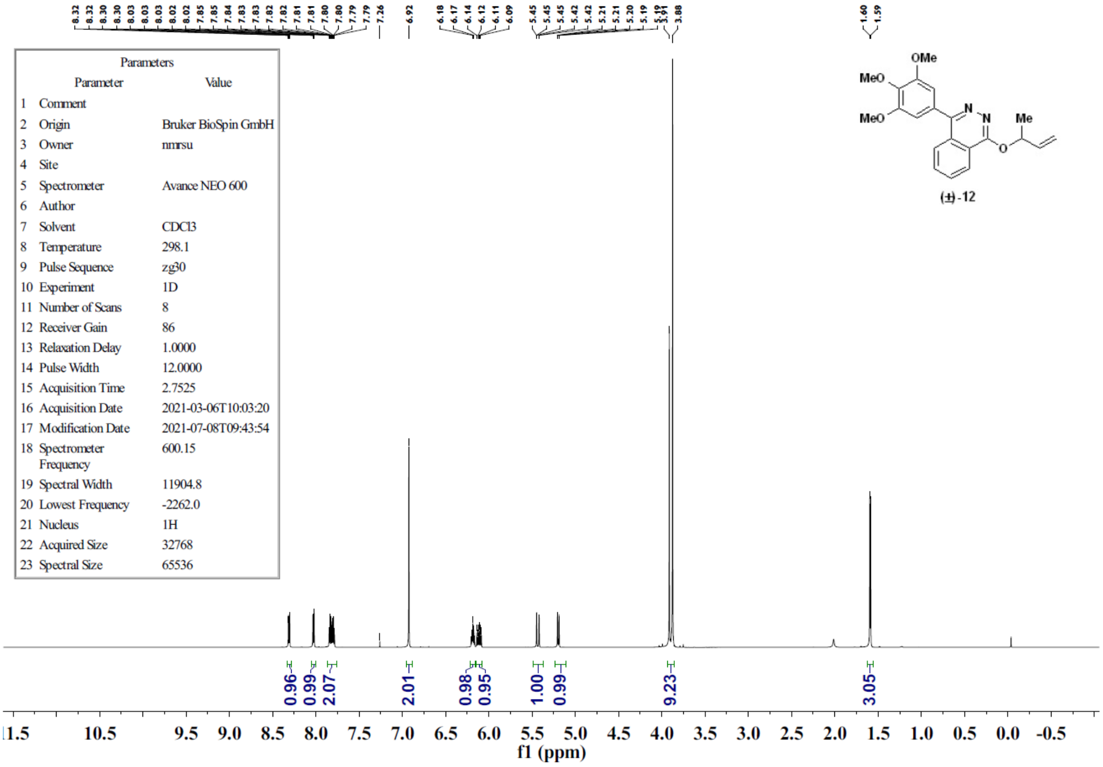

Supplementary Figure 253. ^{1}H NMR (600 MHz, $CDCl_3$) spectrum of compound **(±)-12**.

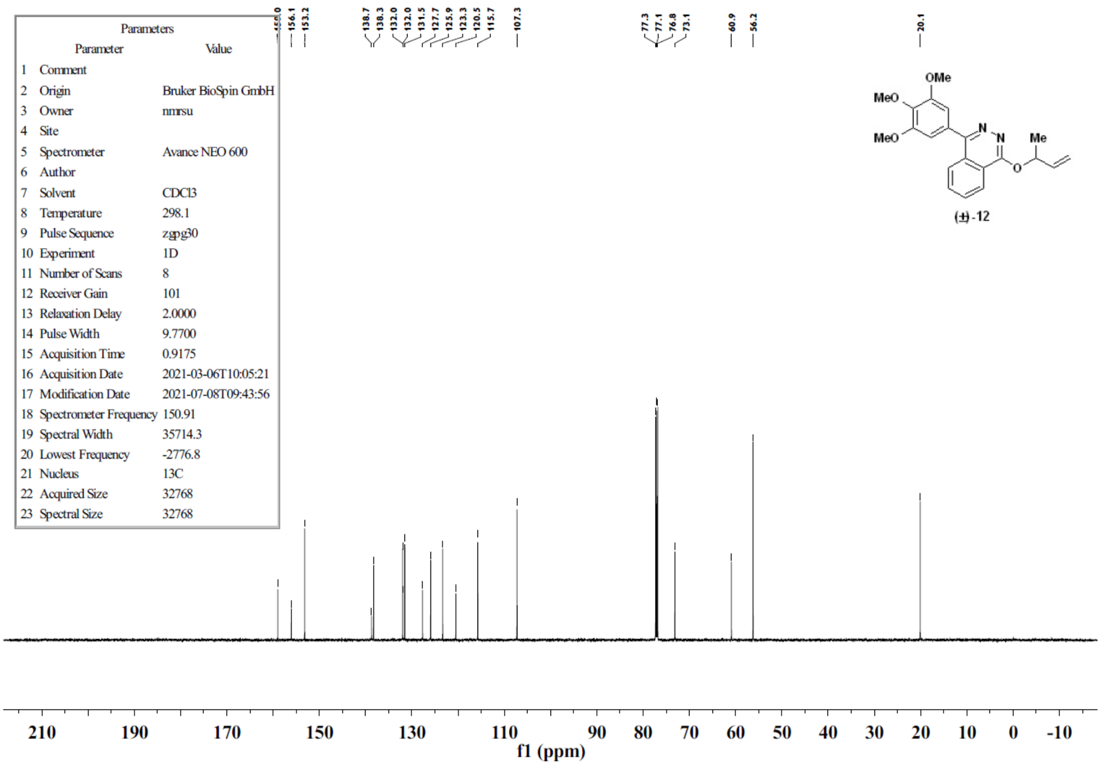

Supplementary Figure 254. ^{13}C NMR (151 MHz, $CDCl_3$) spectrum of compound **(±)-12**.

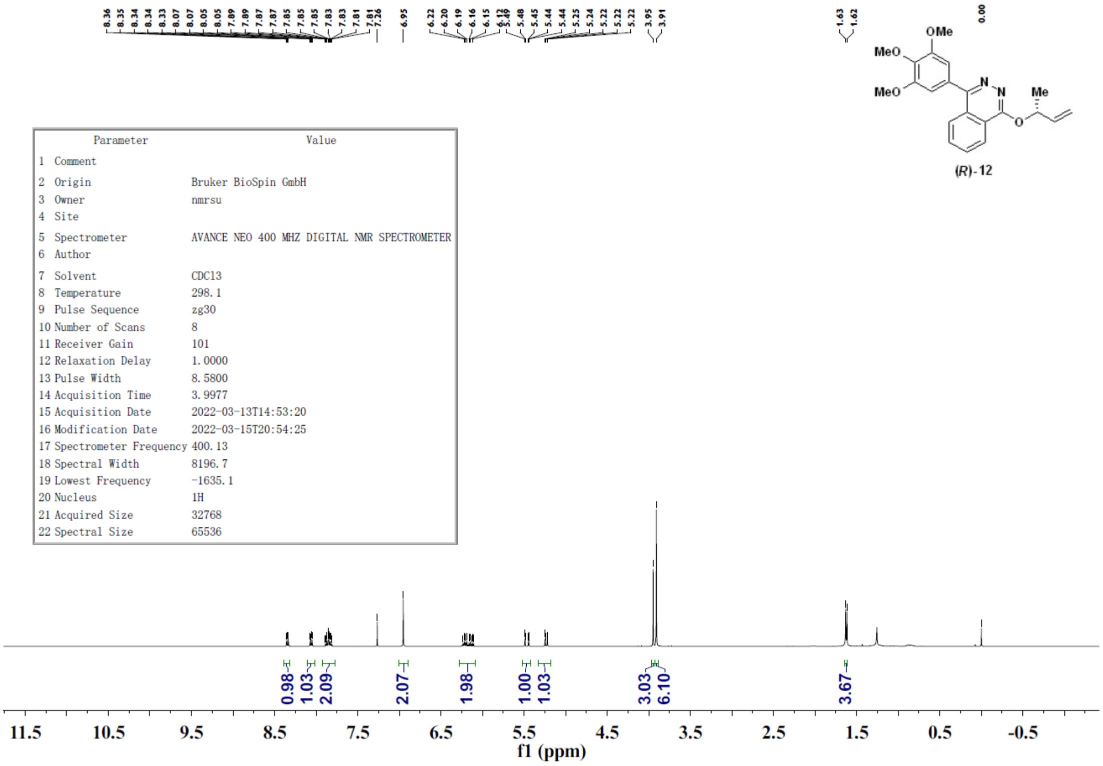

Supplementary Figure 255. ^{1}H NMR (600 MHz, $CDCl_3$) spectrum of compound **(*R*)-12**.

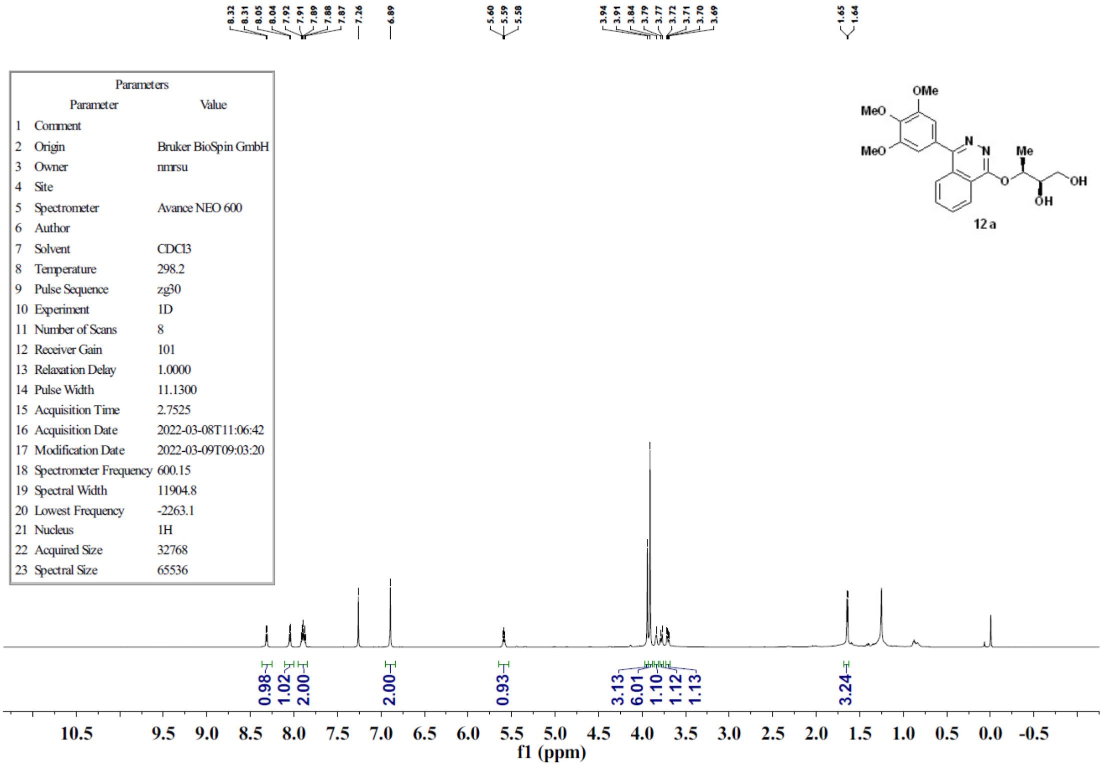

Supplementary Figure 256. ^{1}H NMR (600 MHz, $CDCl_3$) spectrum of compound **12a**.

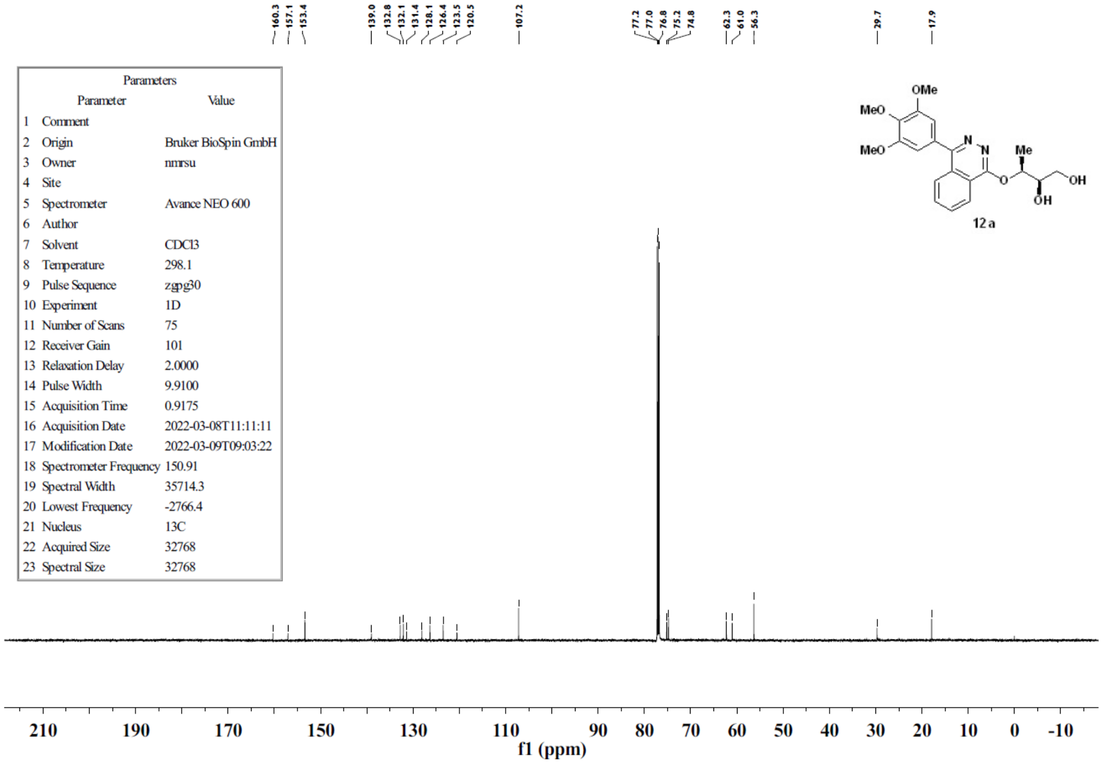

Supplementary Figure 257. ^{13}C NMR (151 MHz, $CDCl_3$) spectrum of compound **12a**.

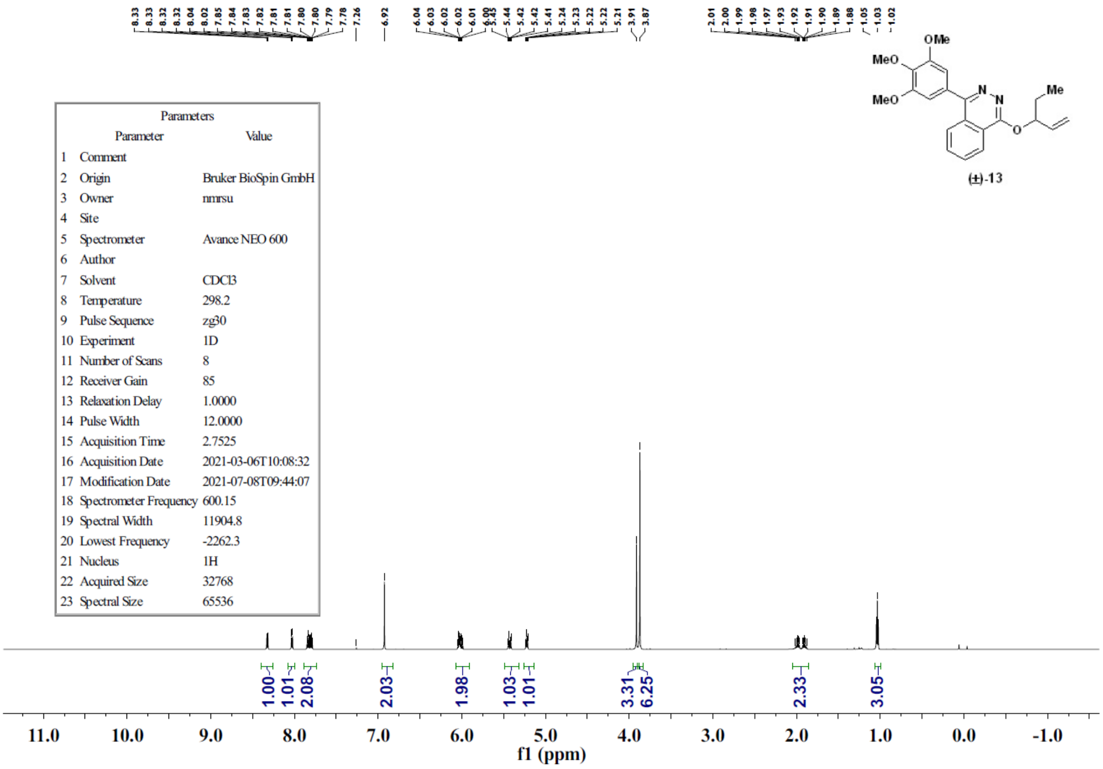

Supplementary Figure 258. ^{1}H NMR (600 MHz, $CDCl_3$) spectrum of compound **(±)-13**.

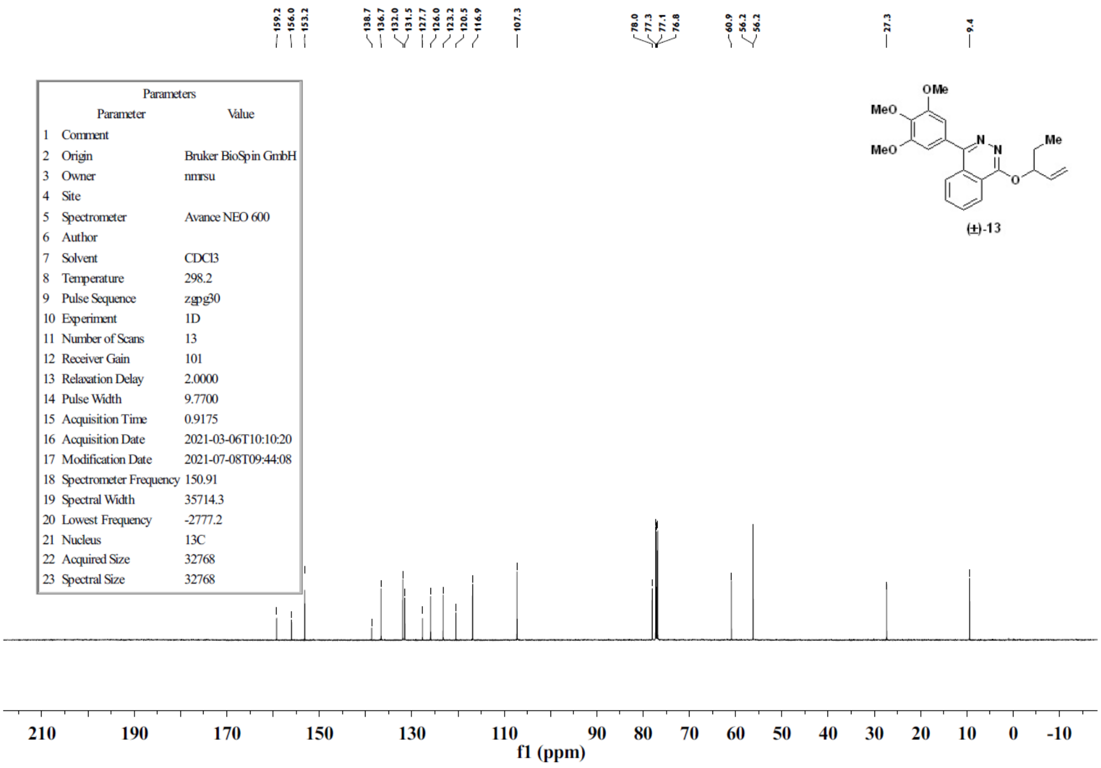

Supplementary Figure 259. ^{13}C NMR (151 MHz, $CDCl_3$) spectrum of compound **(±)-13**.

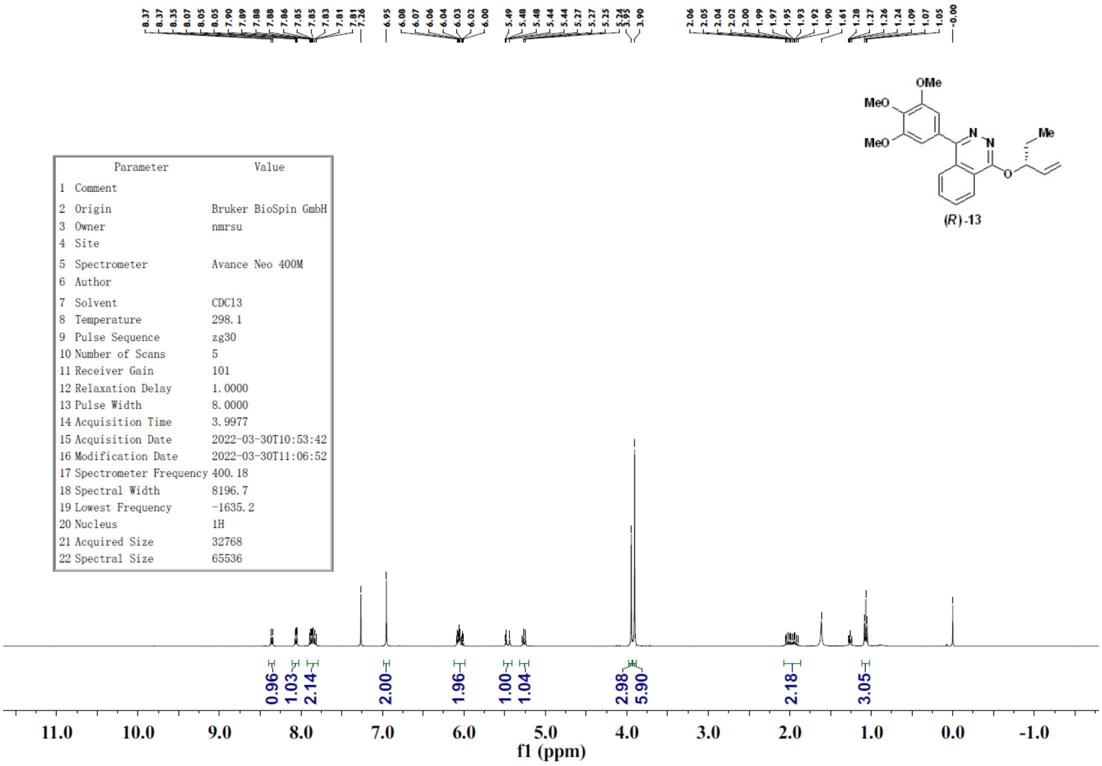

Supplementary Figure 260. ^{1}H NMR (600 MHz, $CDCl_3$) spectrum of compound ***(R)*-13**.

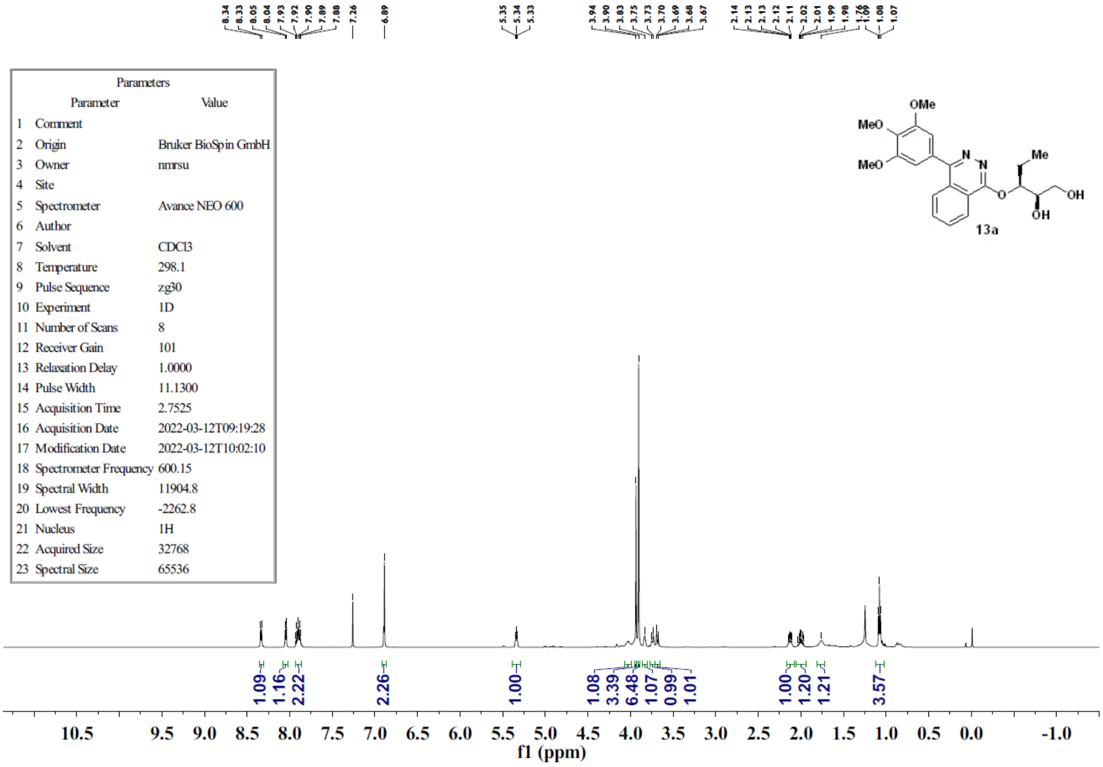

Supplementary Figure 261. ^{1}H NMR (600 MHz, $CDCl_3$) spectrum of compound **13a**.

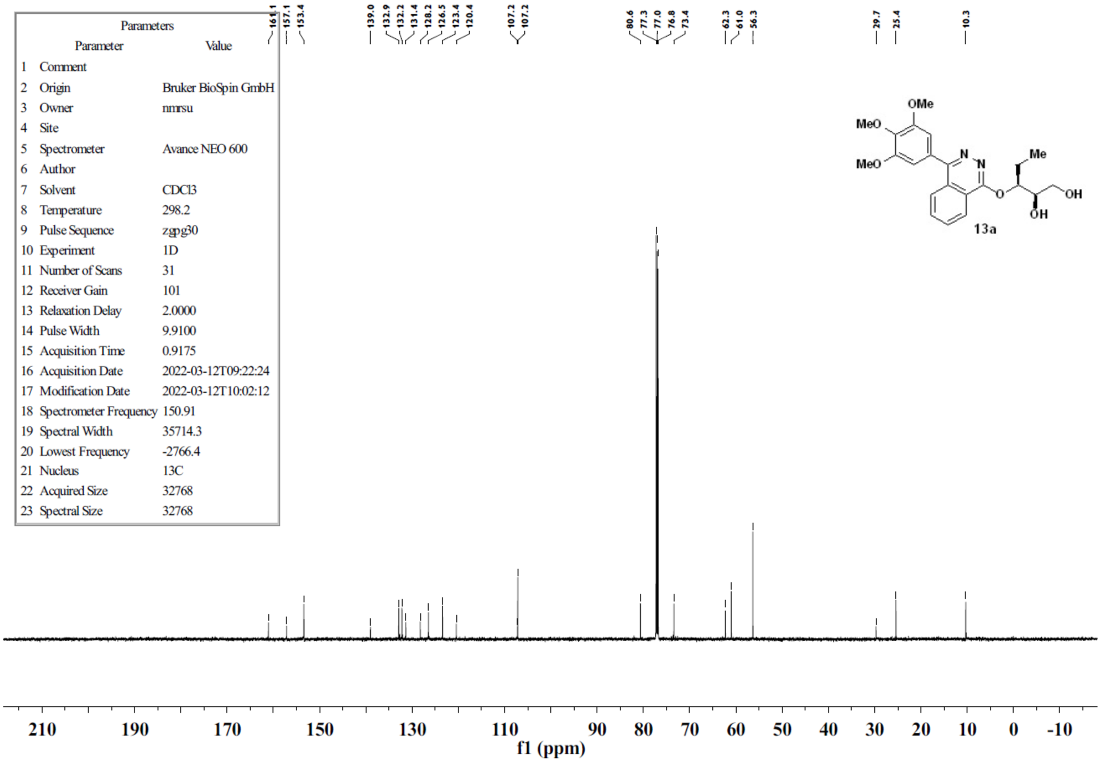

Supplementary Figure 262. ^{13}C NMR (151 MHz, $CDCl_3$) spectrum of compound **13a**.

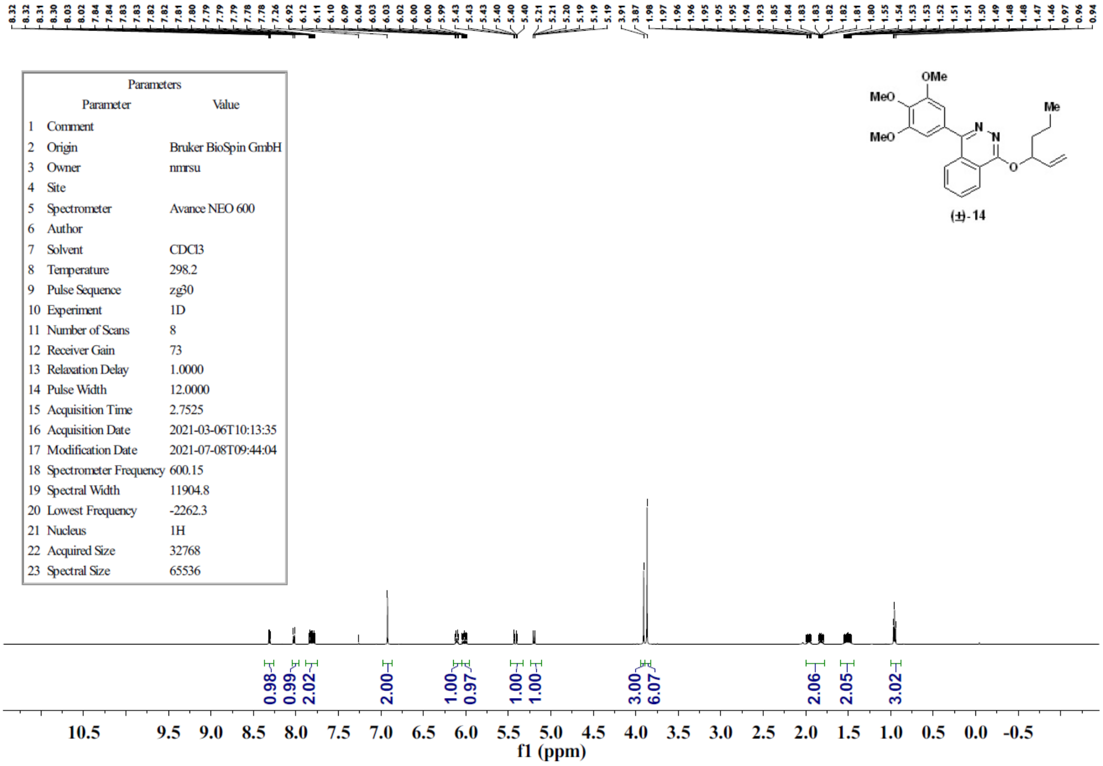

Supplementary Figure 263. ^{1}H NMR (600 MHz, $CDCl_3$) spectrum of compound **(±)-14**.

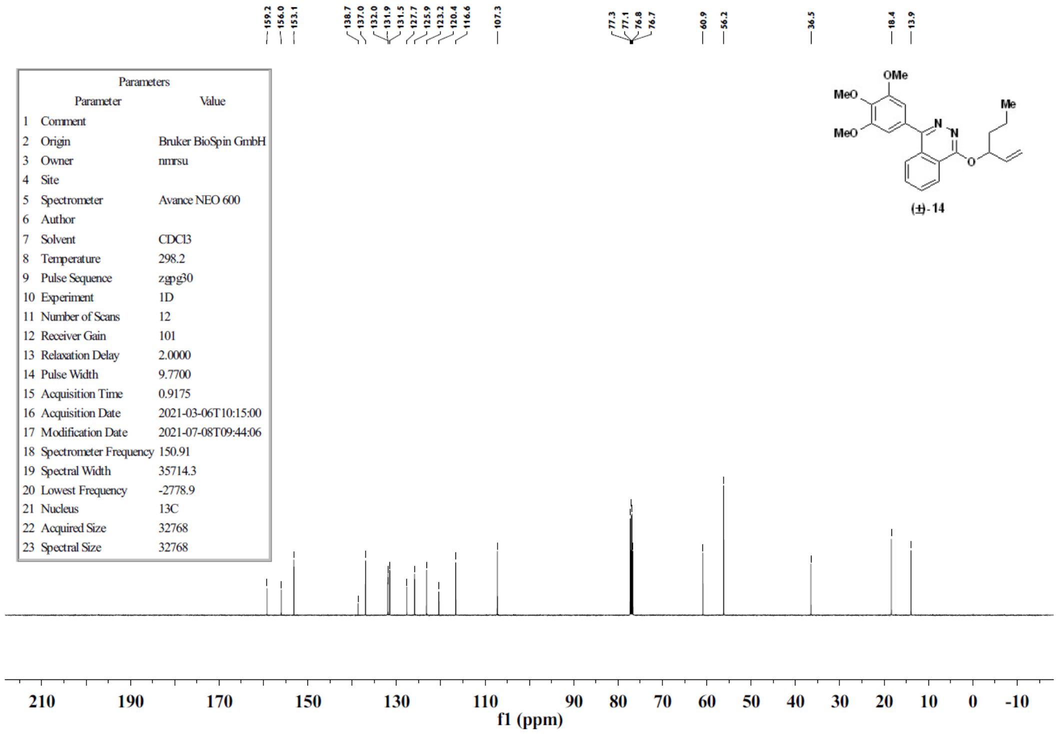

Supplementary Figure 264. ^{13}C NMR (151 MHz, $CDCl_3$) spectrum of compound **(±)-14**.

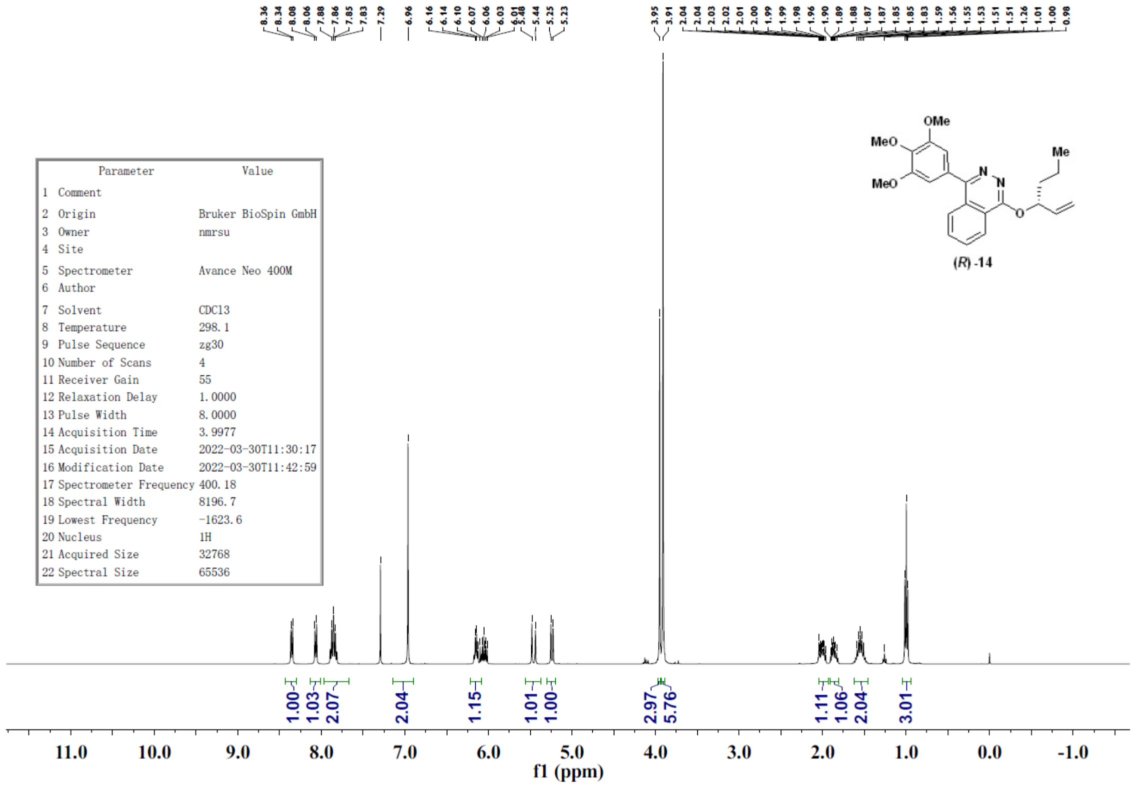

Supplementary Figure 265. ^{1}H NMR (400 MHz, $CDCl_3$) spectrum of compound ***(R)*-14**.

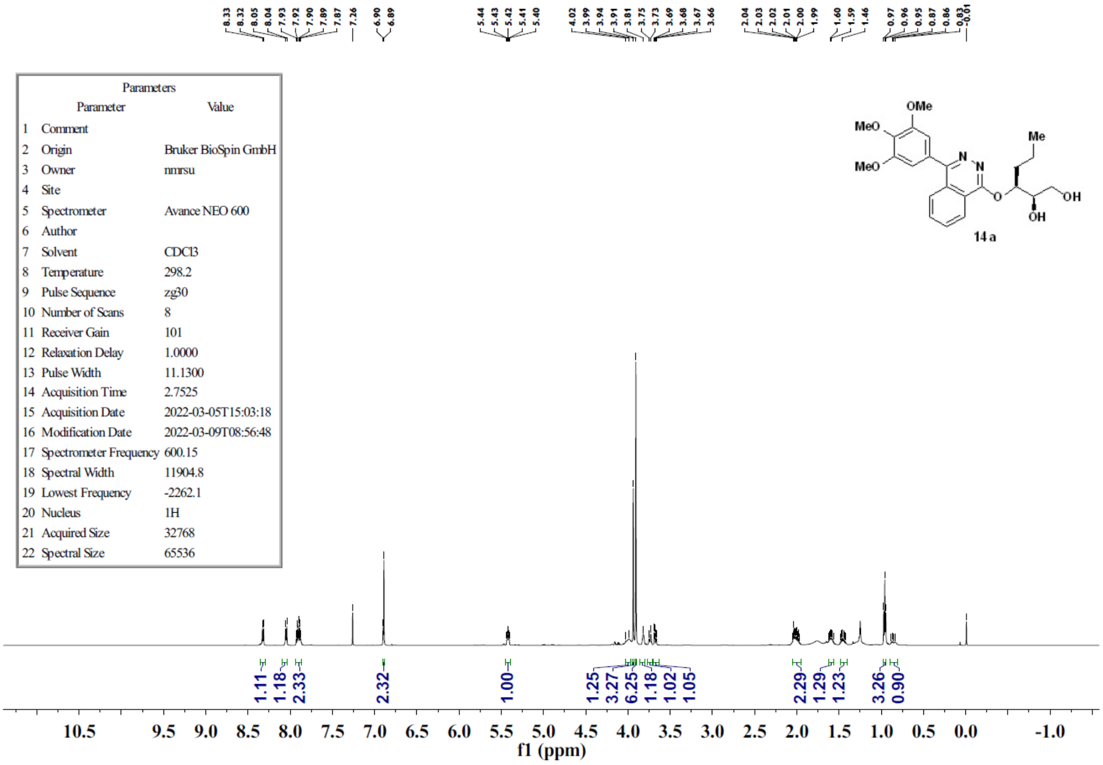

Supplementary Figure 266. ^{1}H NMR (600 MHz, $CDCl_3$) spectrum of compound **14a**.

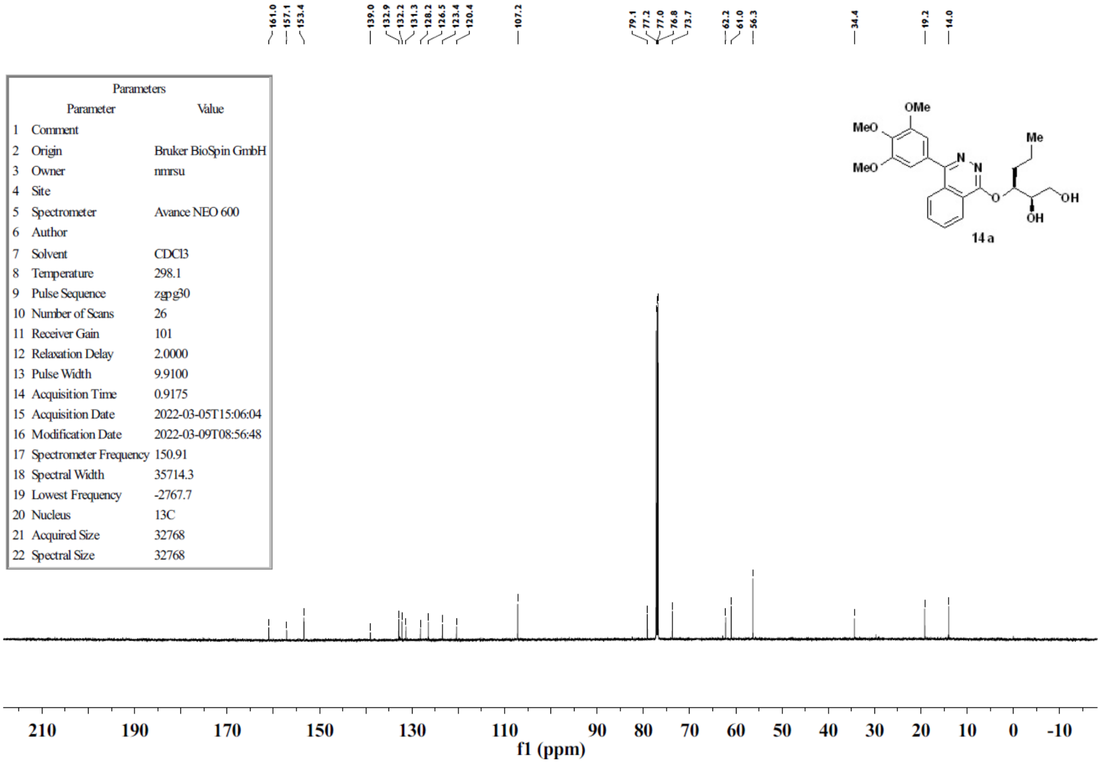

Supplementary Figure 267. ^{13}C NMR (151 MHz, $CDCl_3$) spectrum of compound **14a**.

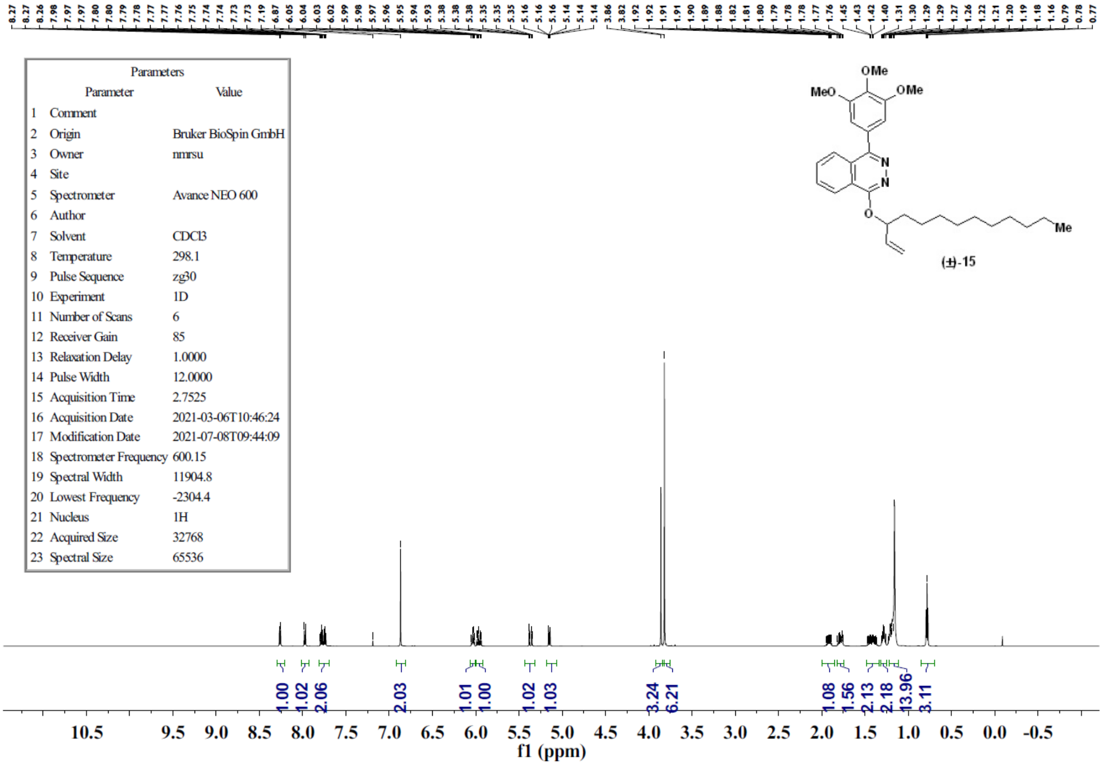

Supplementary Figure 268. ^{1}H NMR (600 MHz, $CDCl_3$) spectrum of compound **(±)-15**.

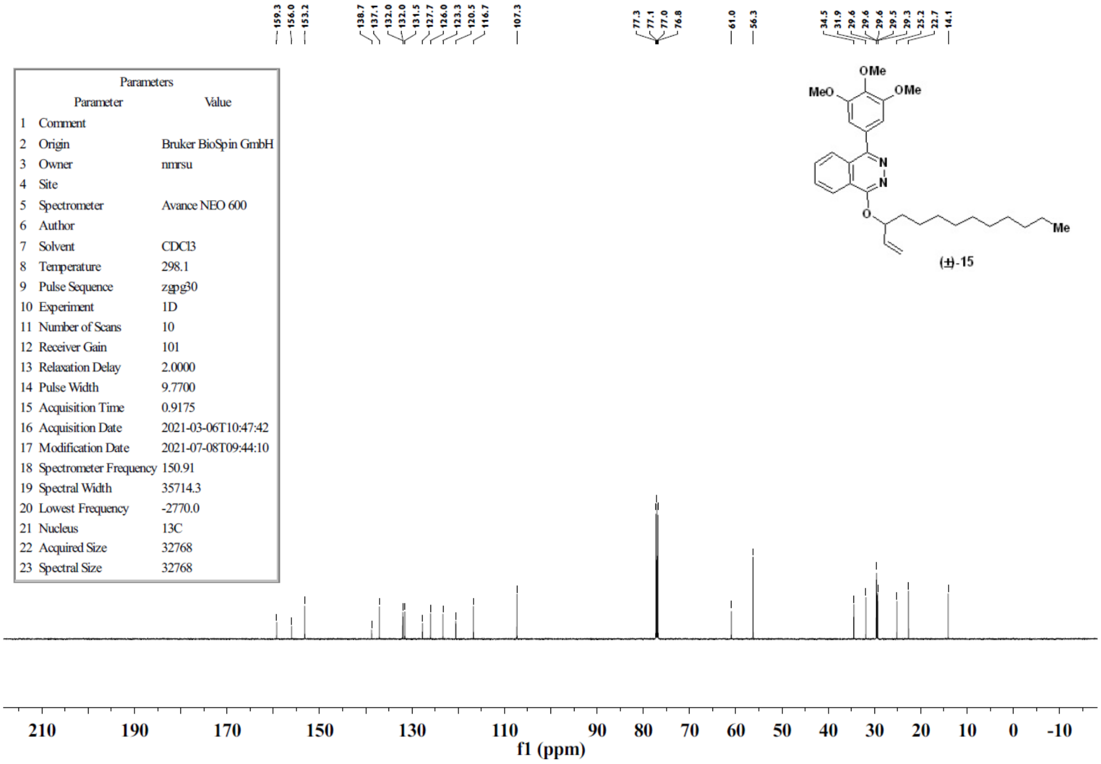

Supplementary Figure 269. ^{13}C NMR (151 MHz, $CDCl_3$) spectrum of compound **(±)-15**.

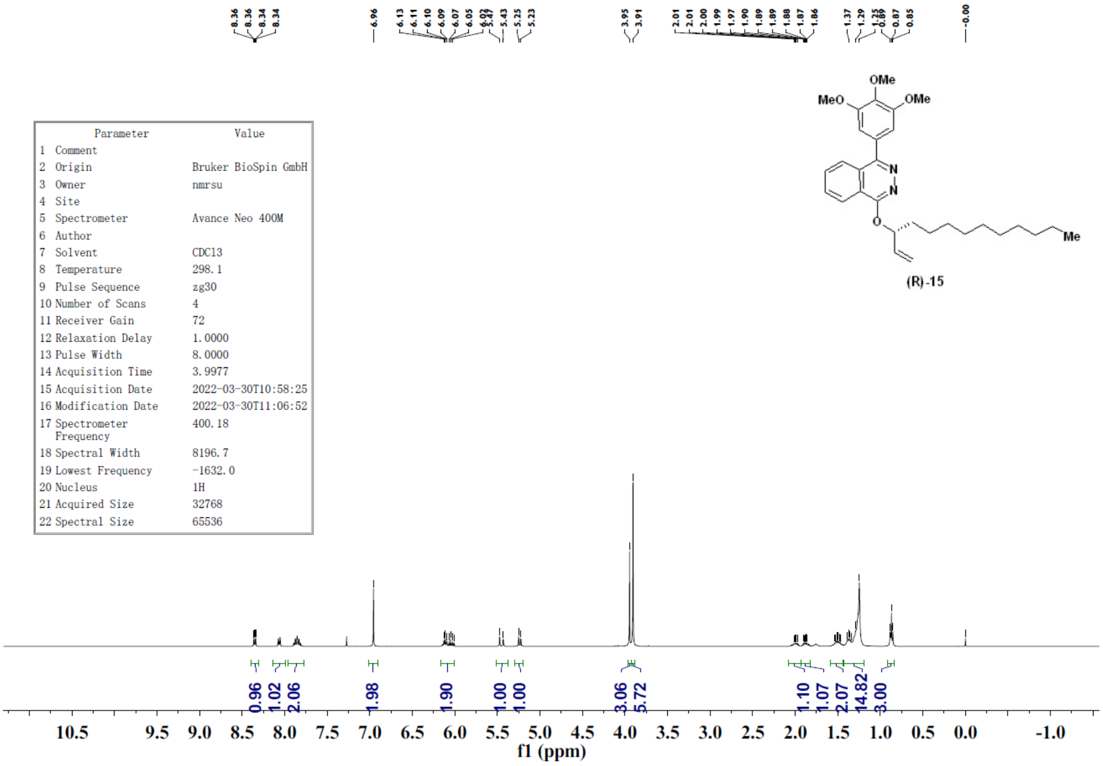

Supplementary Figure 270. ^{1}H NMR (400 MHz, $CDCl_3$) spectrum of compound ***(R)*-15**.

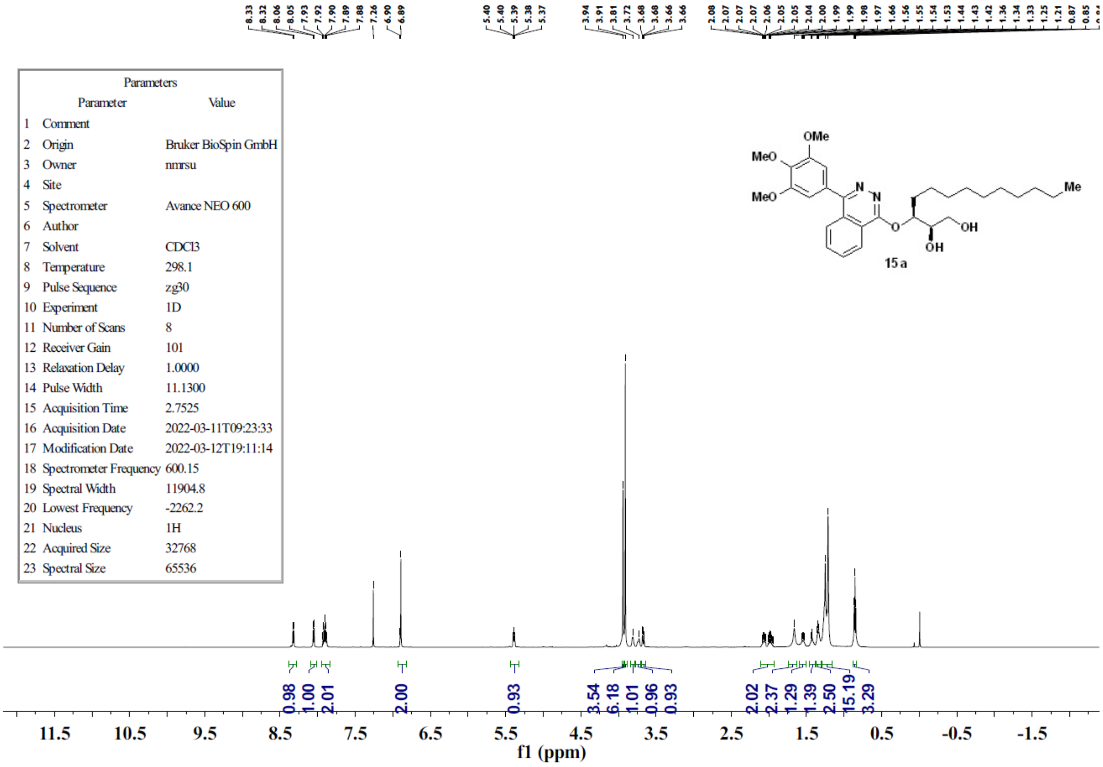

Supplementary Figure 271. ^{1}H NMR (600 MHz, $CDCl_3$) spectrum of compound **15a**.

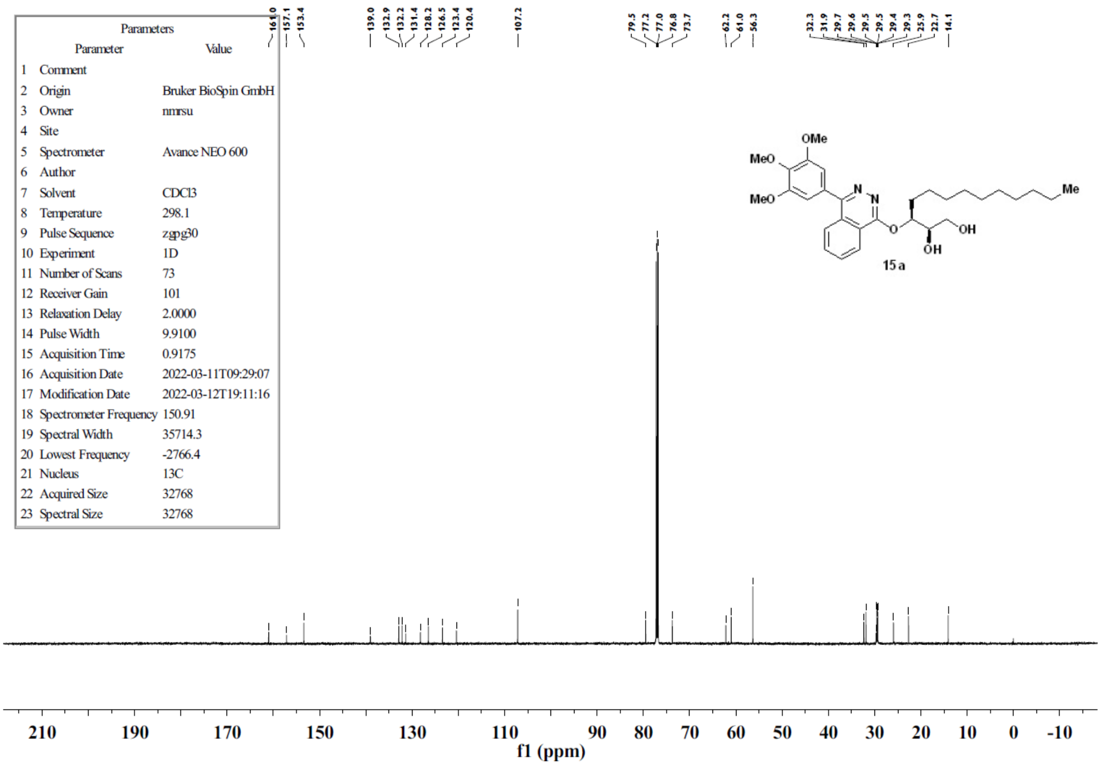

Supplementary Figure 272. ^{13}C NMR (151 MHz, $CDCl_3$) spectrum of compound **15a**.

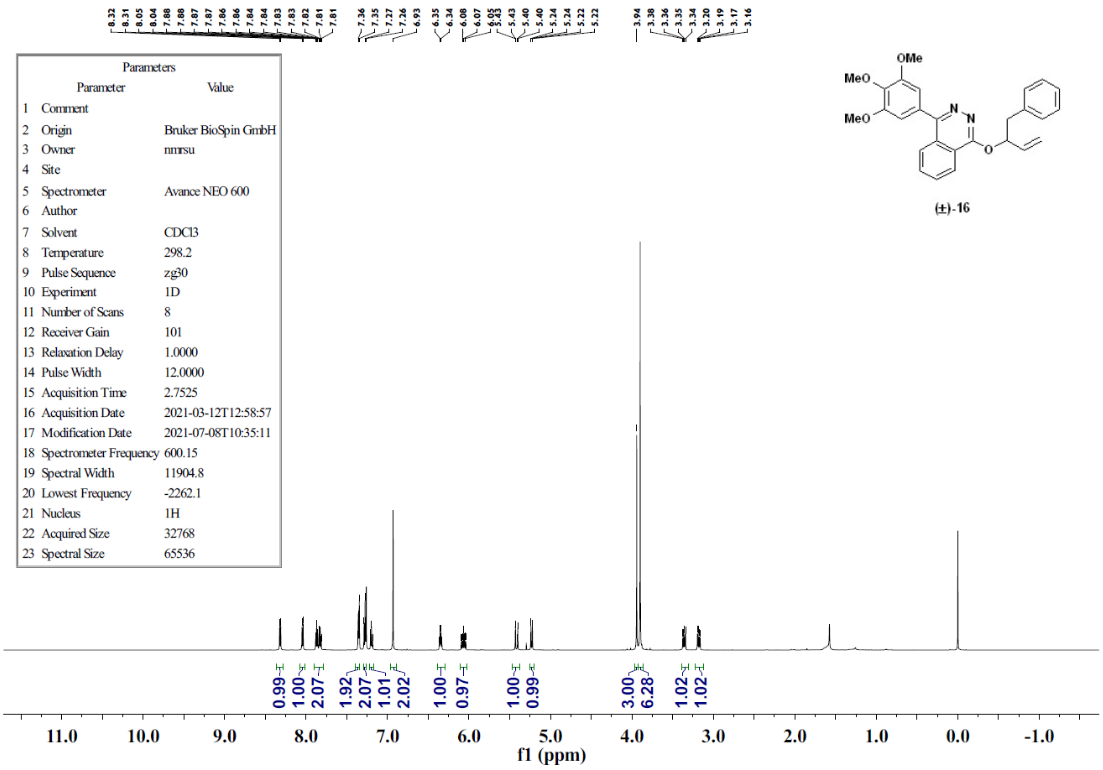

Supplementary Figure 273. ^{1}H NMR (600 MHz, $CDCl_3$) spectrum of compound **(±)-16**.

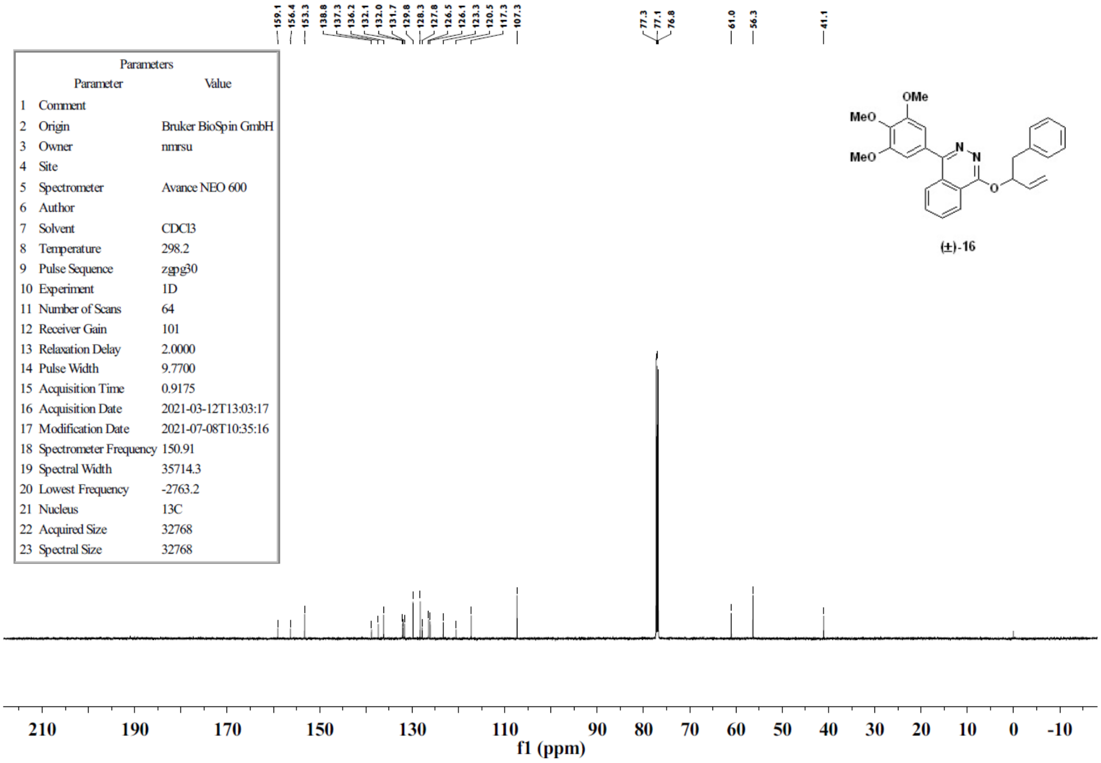

Supplementary Figure 274. ^{13}C NMR (151 MHz, $CDCl_3$) spectrum of compound **(±)-16**.

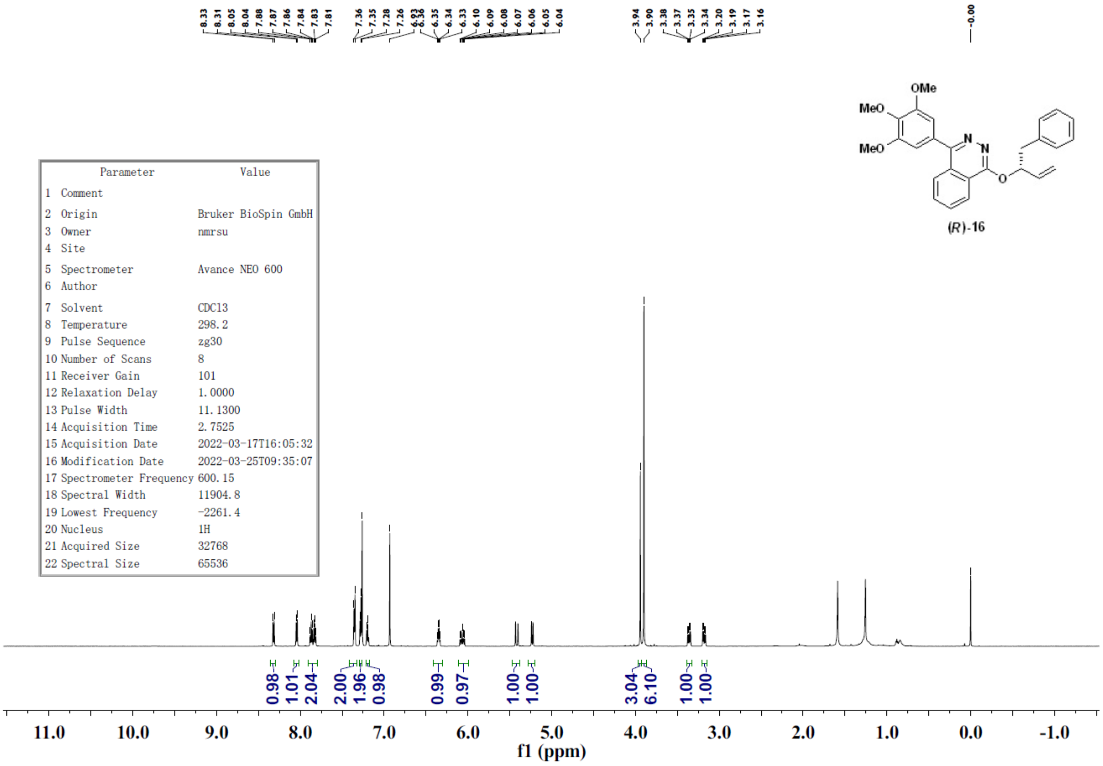

Supplementary Figure 275. ^{1}H NMR (400 MHz, $CDCl_3$) spectrum of compound **(*R*)-16**.

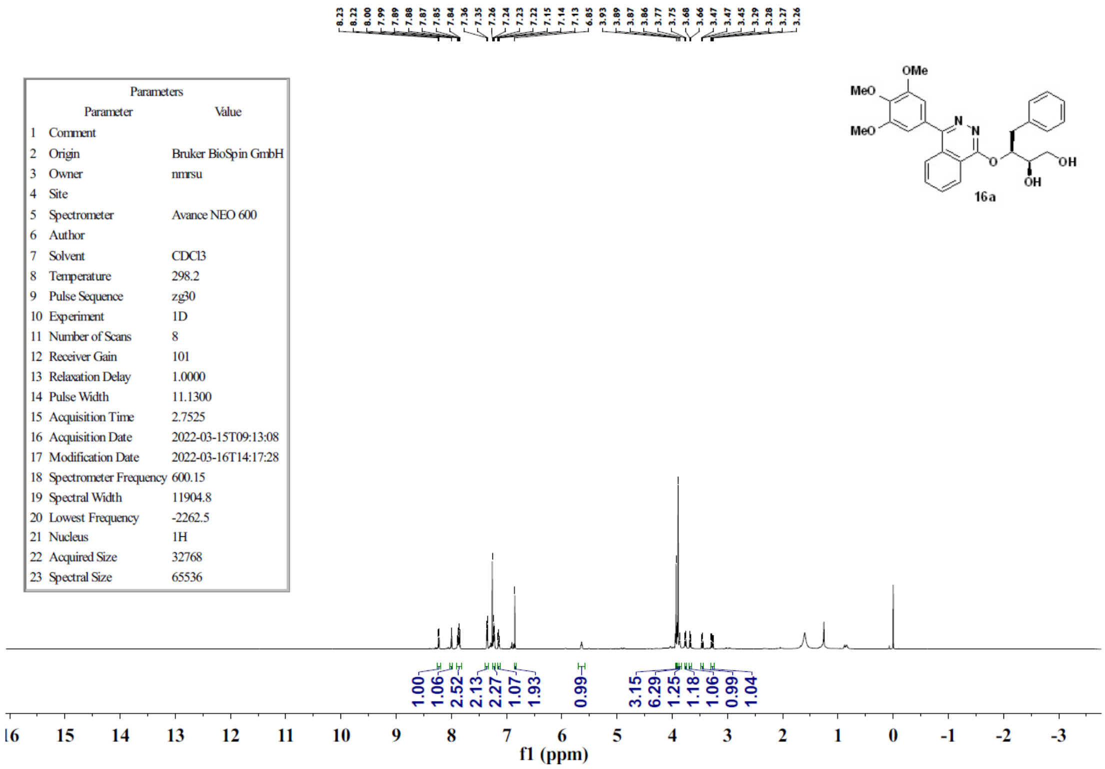

Supplementary Figure 276. ^{1}H NMR (600 MHz, $CDCl_3$) spectrum of compound **16a**.

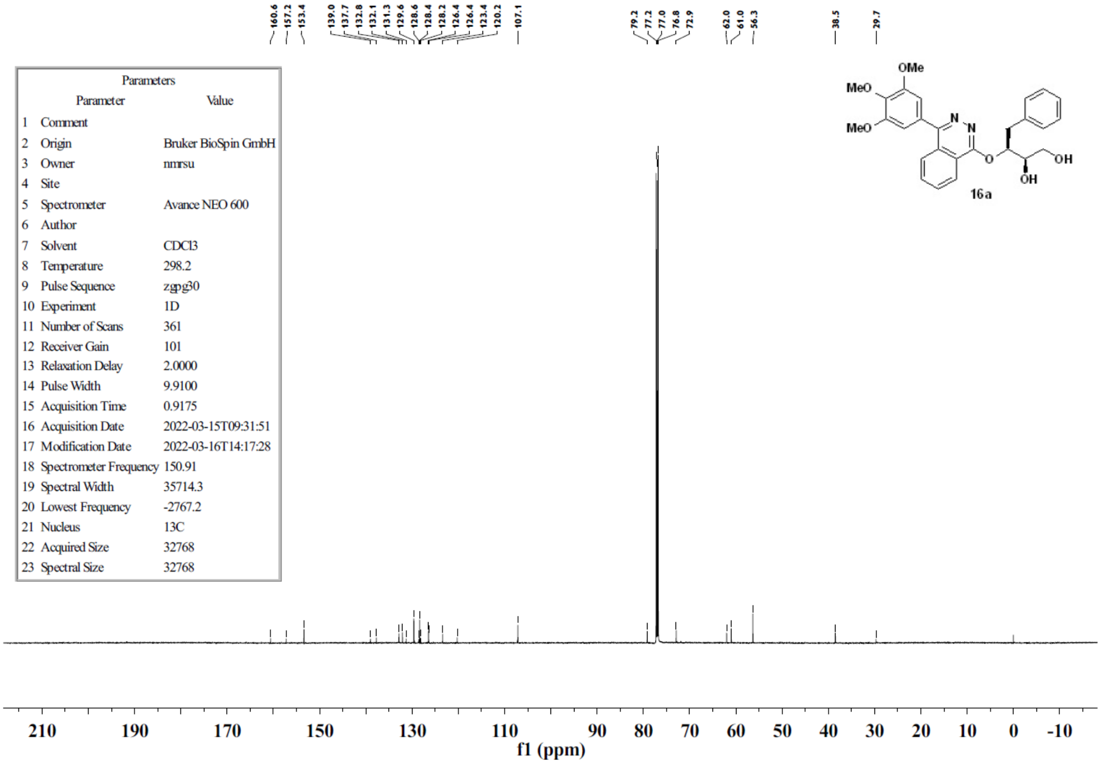

Supplementary Figure 277. ^{13}C NMR (151 MHz, $CDCl_3$) spectrum of compound **16a**.

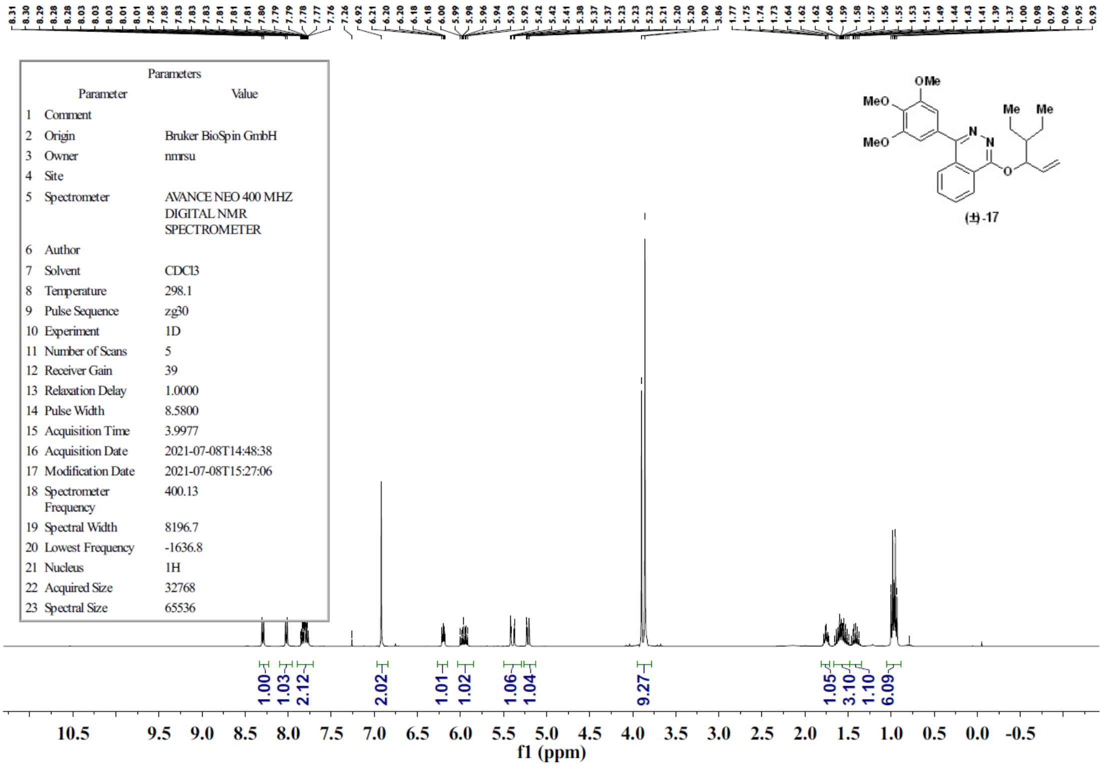

Supplementary Figure 278. ^{1}H NMR (600 MHz, $CDCl_3$) spectrum of compound **(±)-17**.

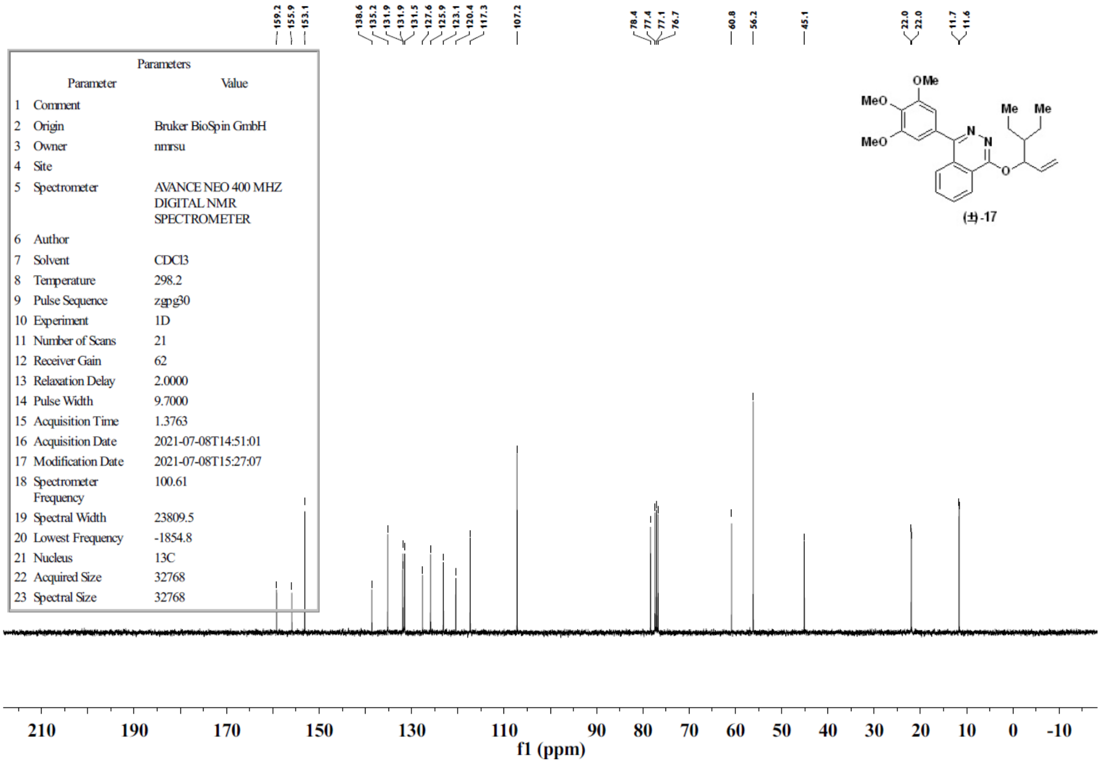

Supplementary Figure 279. ^{13}C NMR (151 MHz, $CDCl_3$) spectrum of compound **(±)-17**.

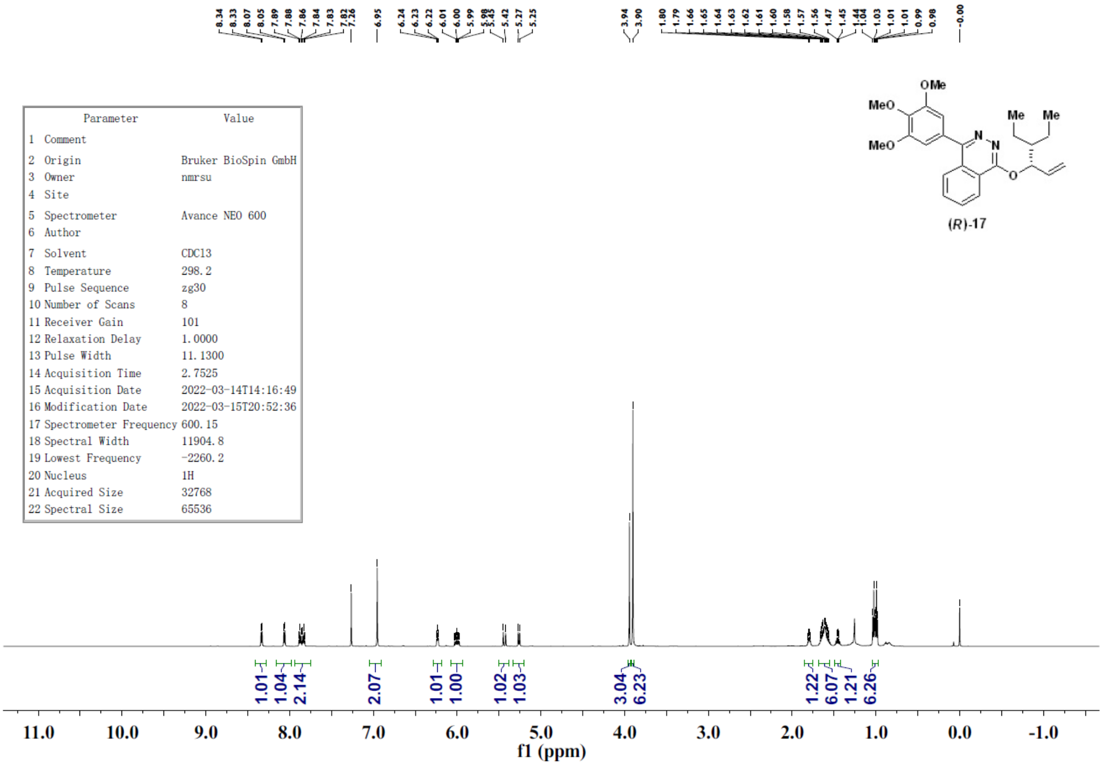

Supplementary Figure 280. ^{1}H NMR (600 MHz, $CDCl_3$) spectrum of compound ***(R)*-17**.

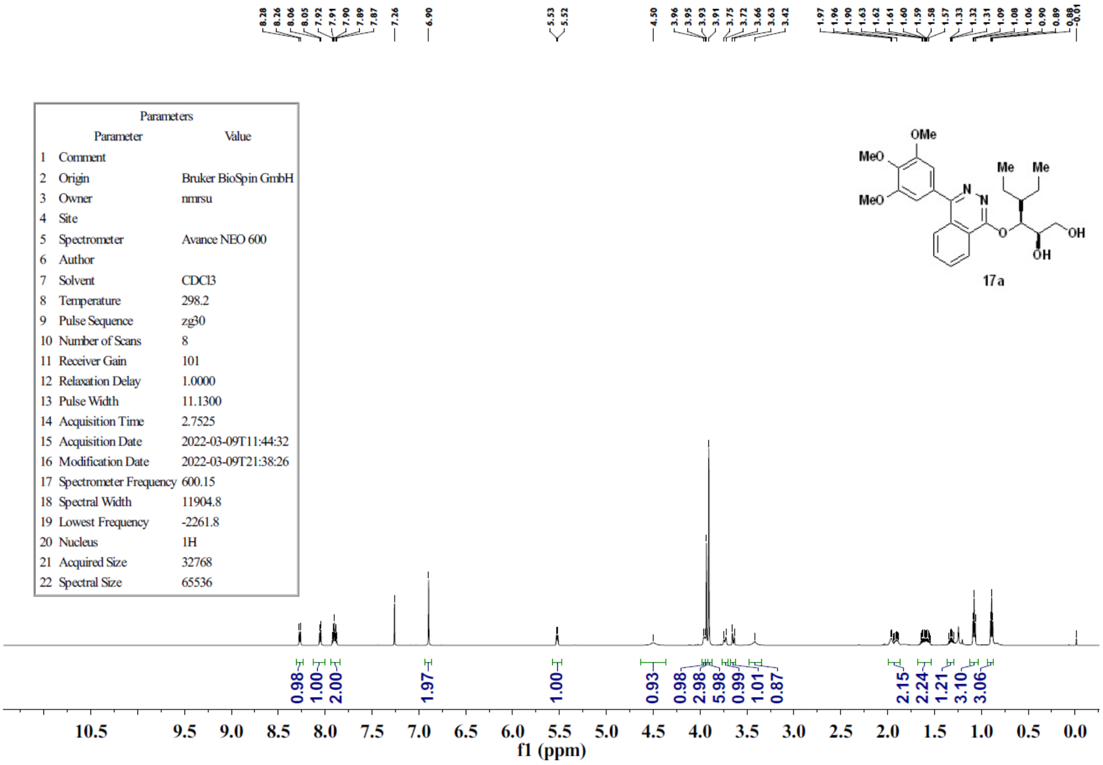

Supplementary Figure 281. ^{1}H NMR (600 MHz, $CDCl_3$) spectrum of compound **17a**.

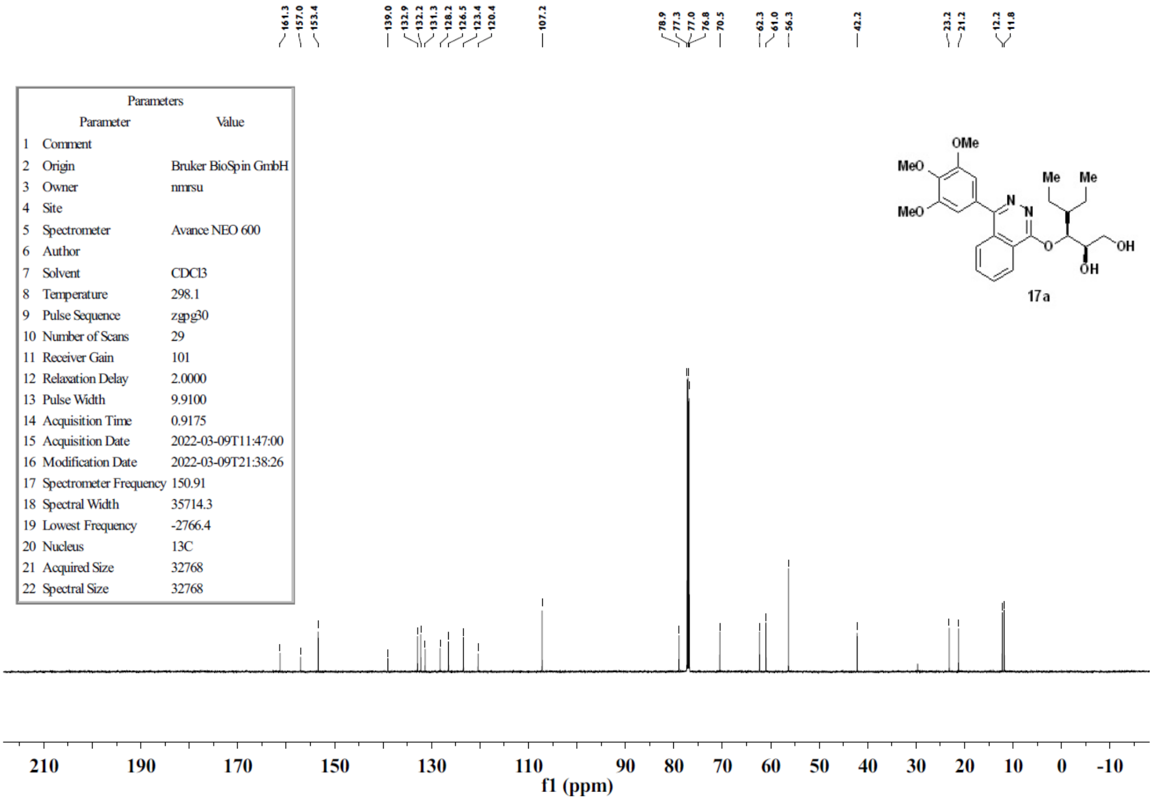

Supplementary Figure 282. ^{13}C NMR (151 MHz, $CDCl_3$) spectrum of compound **17a**.

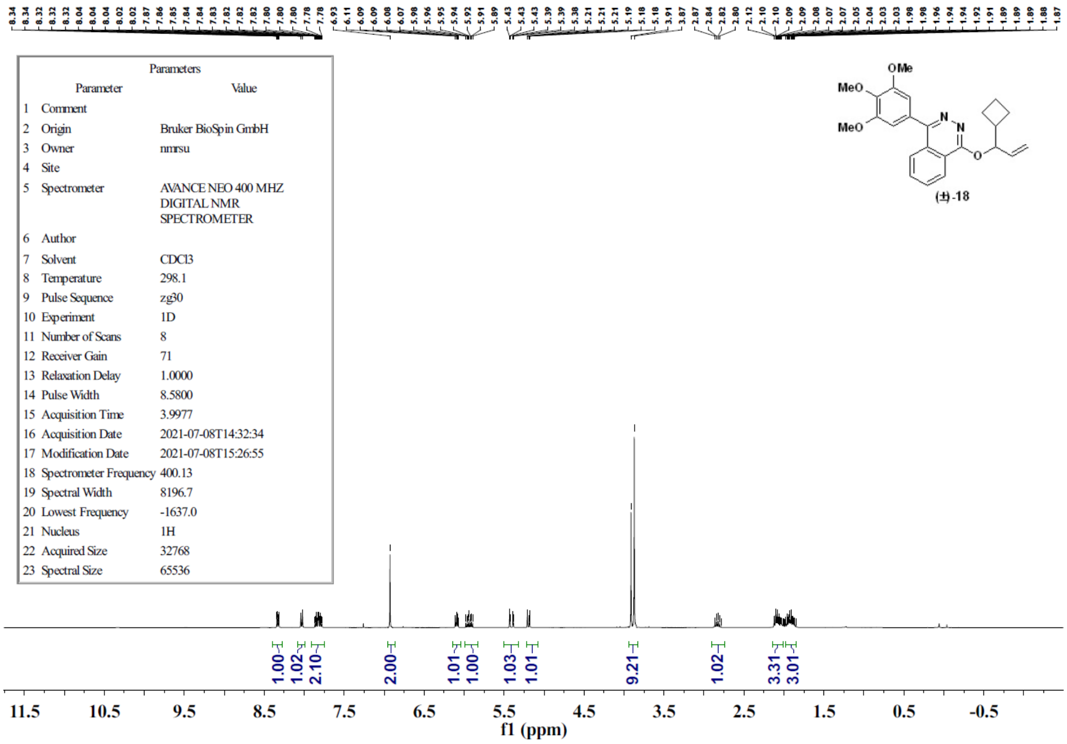

Supplementary Figure 283. ^{1}H NMR (400 MHz, $CDCl_3$) spectrum of compound **(±)-18**.

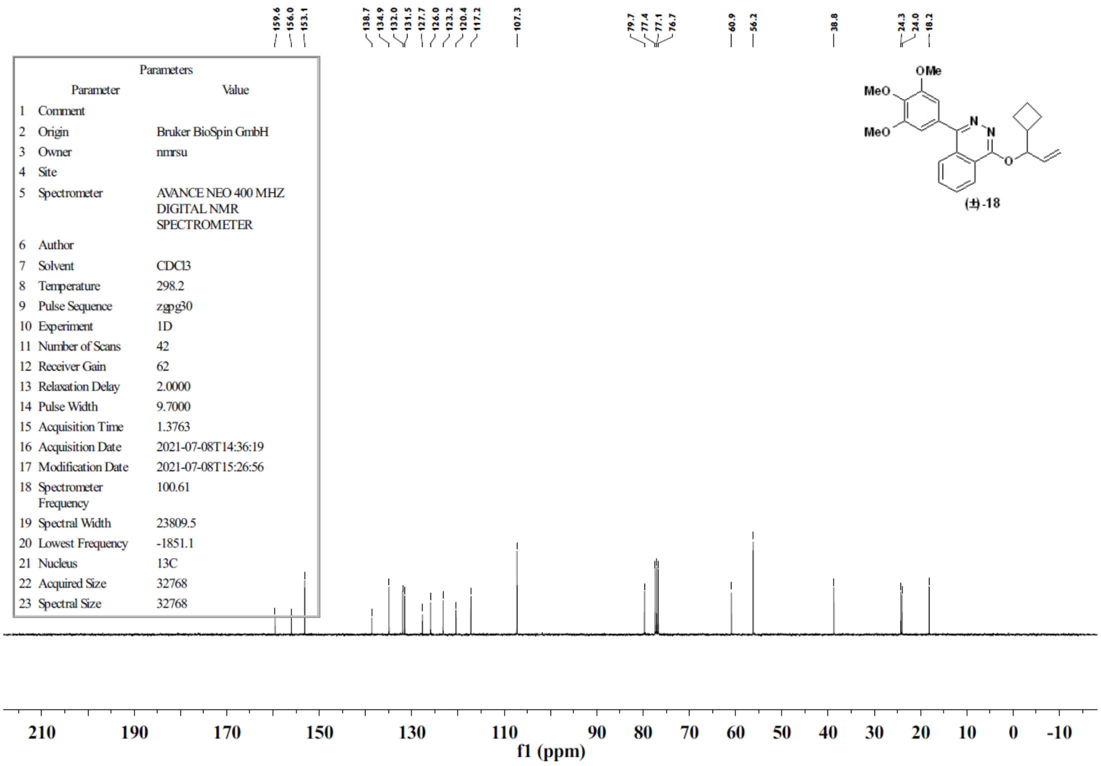

Supplementary Figure 284. ^{13}C NMR (101 MHz, $CDCl_3$) spectrum of compound **(±)-18**.

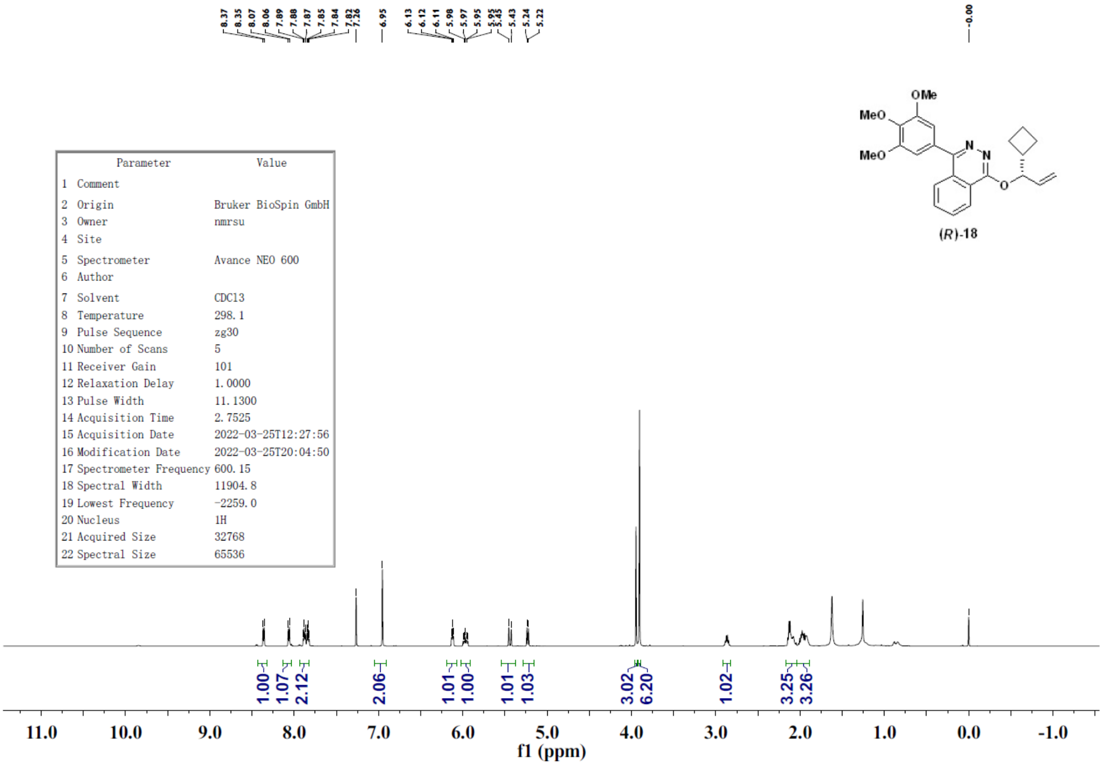

Supplementary Figure 285. ^{1}H NMR (600 MHz, $CDCl_3$) spectrum of compound **(*R*)-18**.

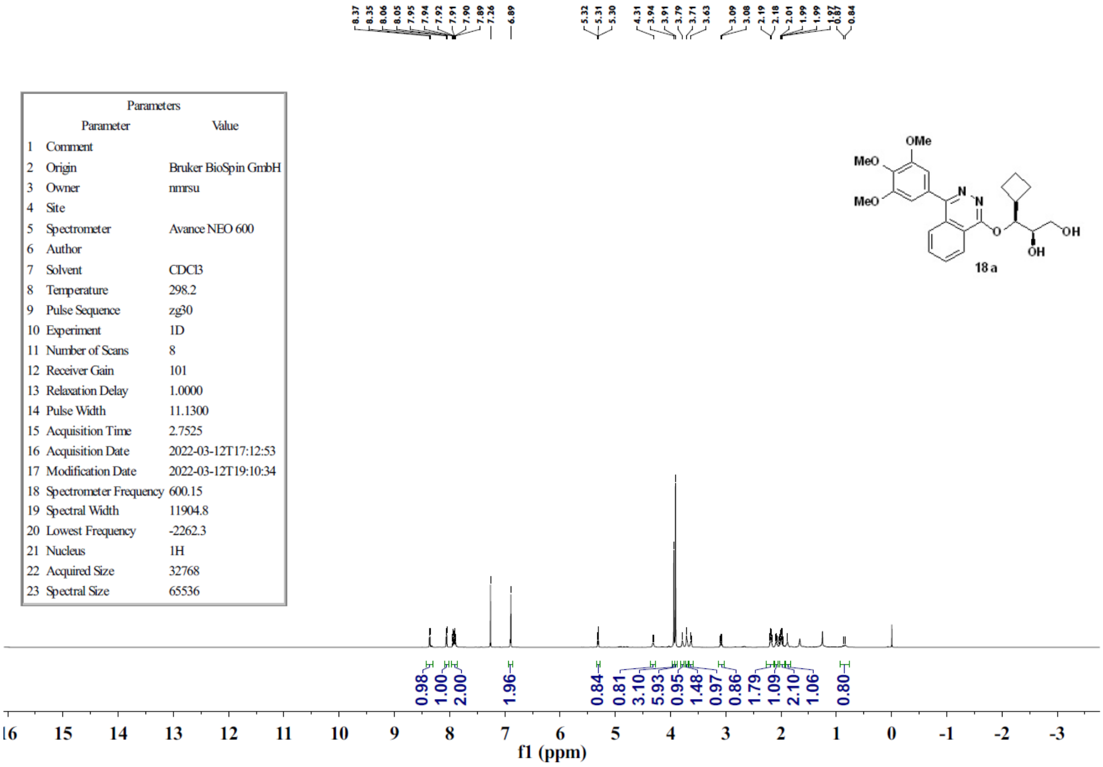

Supplementary Figure 286. ^{1}H NMR (600 MHz, $CDCl_3$) spectrum of compound **18a**.

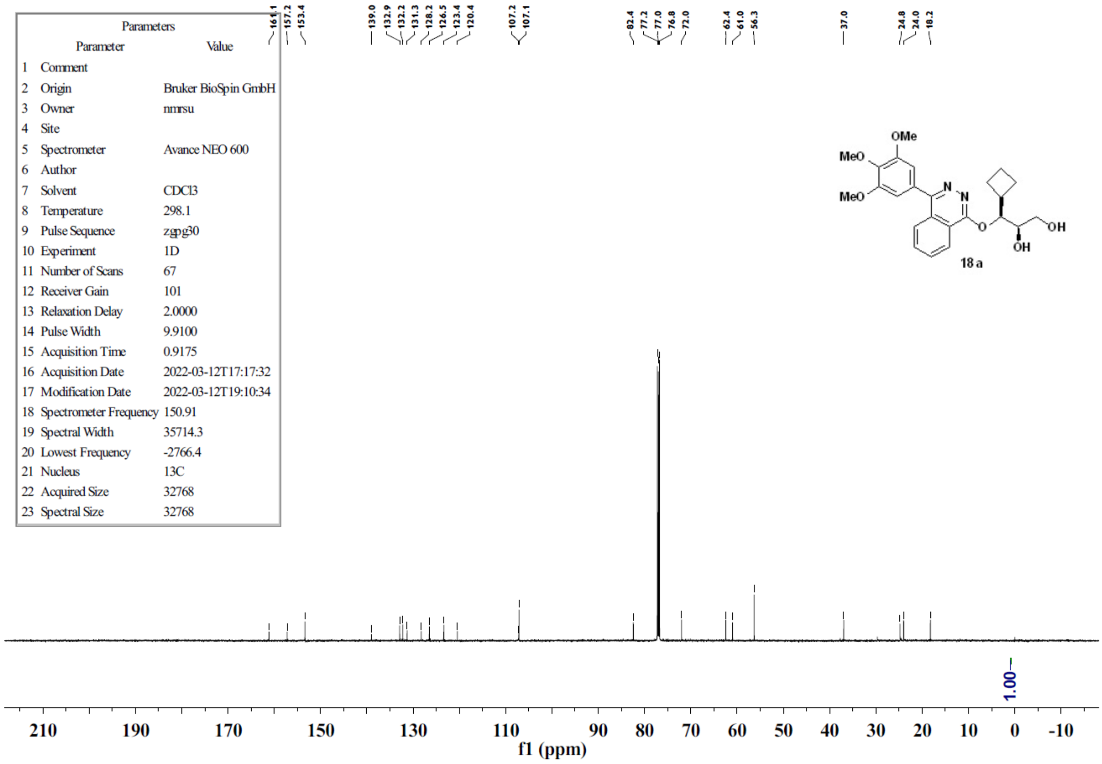

Supplementary Figure 287. ^{13}C NMR (151 MHz, $CDCl_3$) spectrum of compound **18a**.

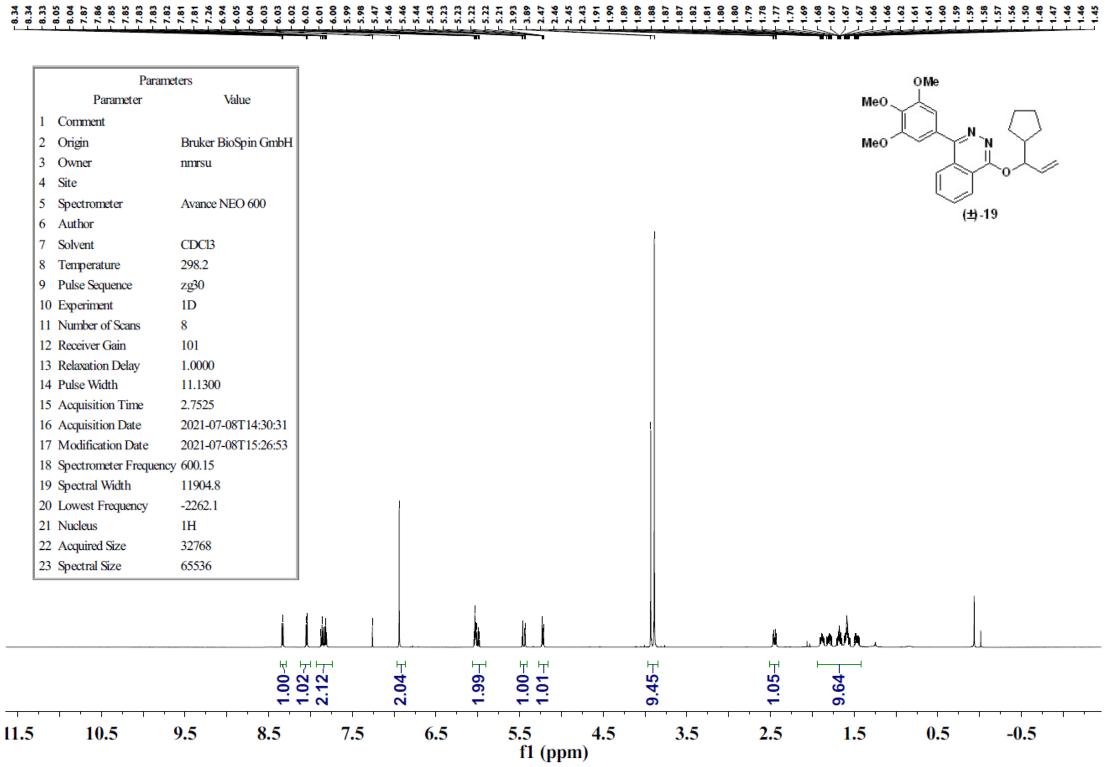

Supplementary Figure 288. ^{1}H NMR (600 MHz, $CDCl_3$) spectrum of compound **(±)-19**.

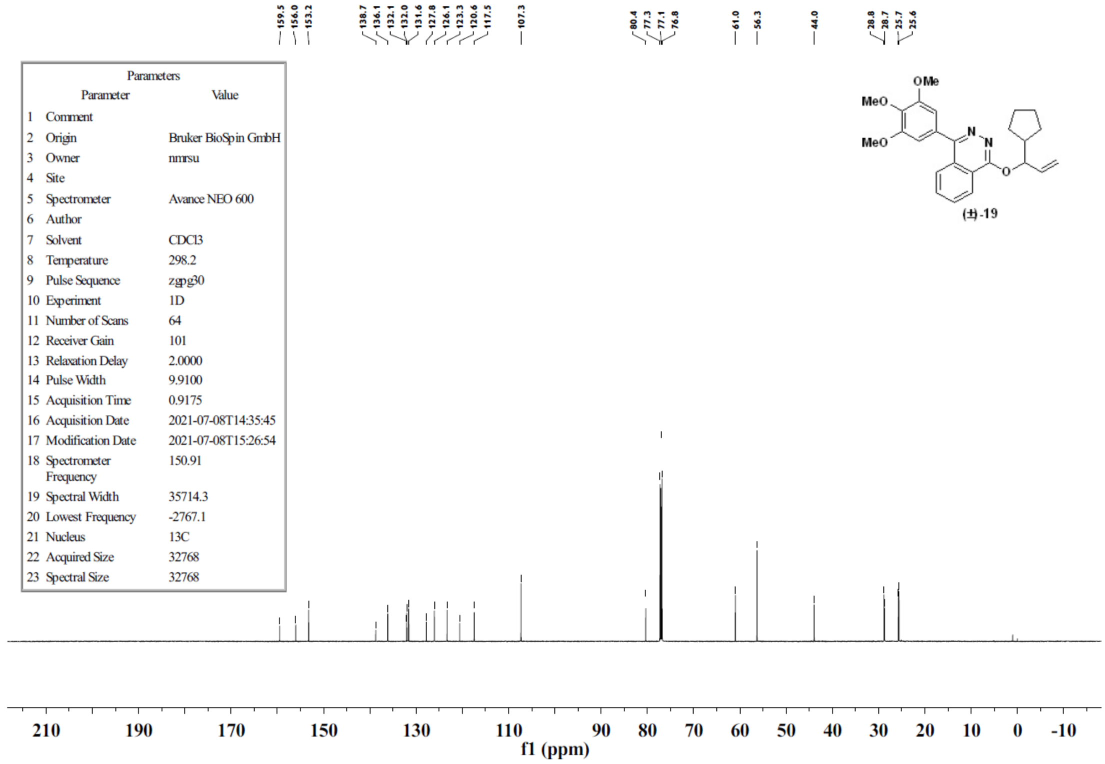

Supplementary Figure 289. ^{13}C NMR (151 MHz, $CDCl_3$) spectrum of compound **(±)-19**.

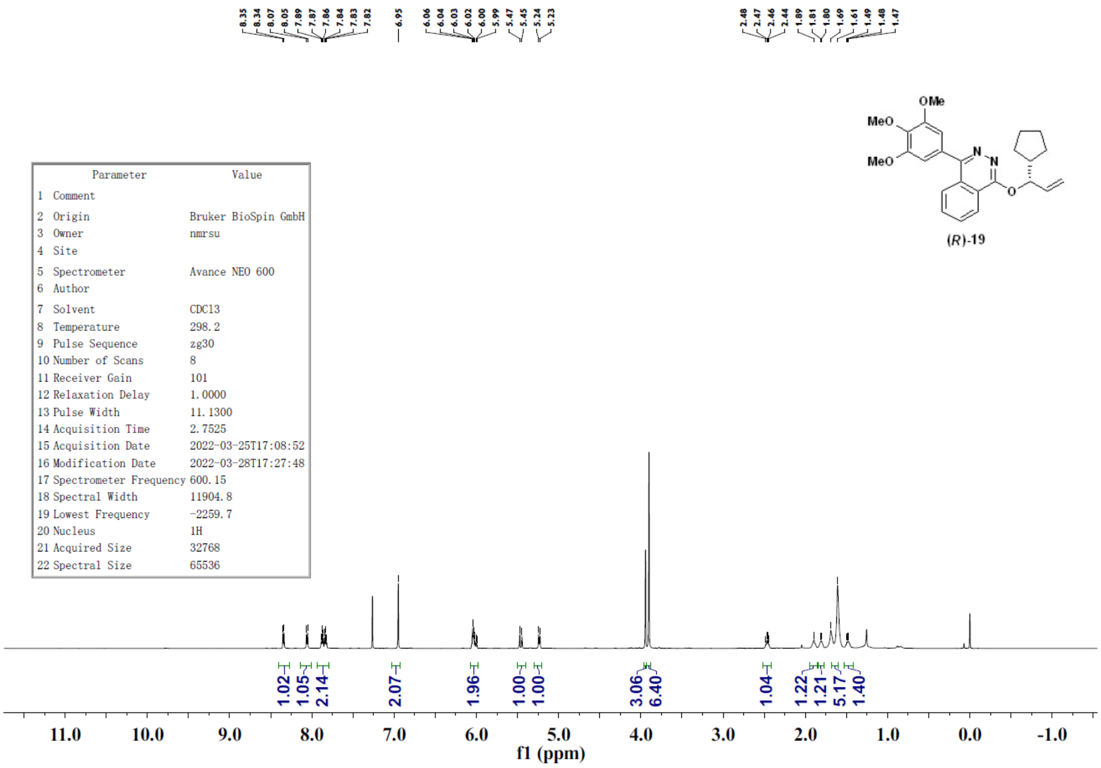

Supplementary Figure 290. ^{1}H NMR (600 MHz, $CDCl_3$) spectrum of compound ***(R)*-19**.

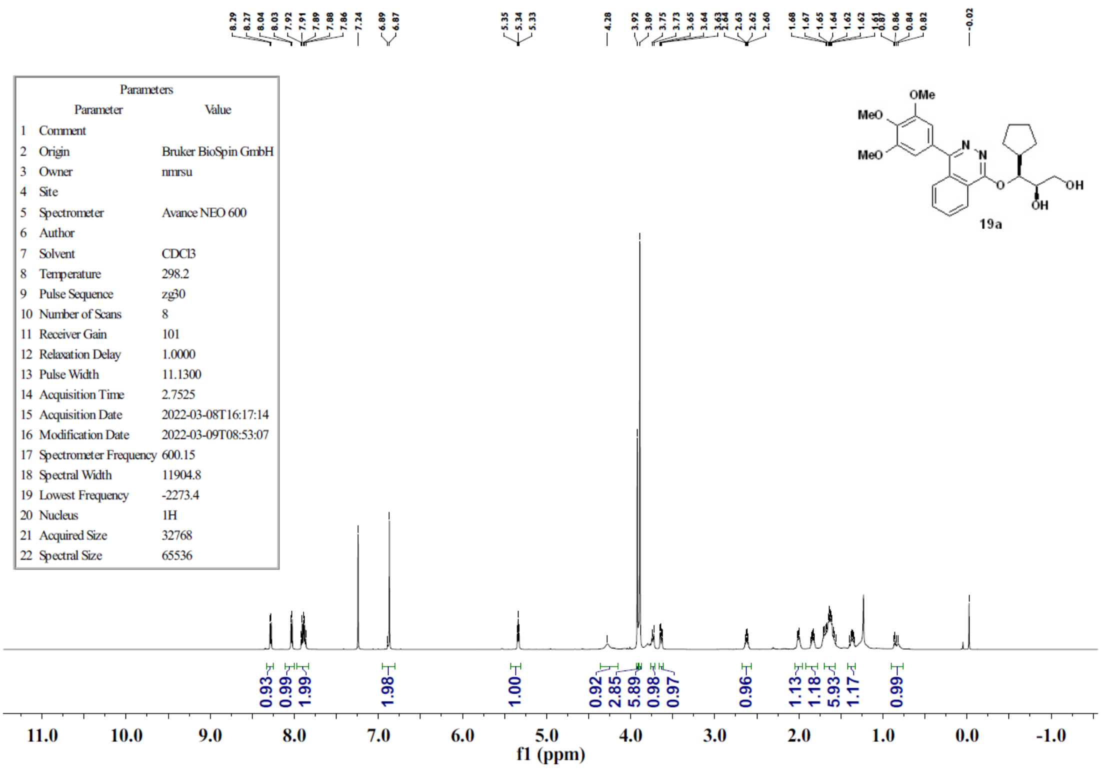

Supplementary Figure 291. ^{1}H NMR (600 MHz, $CDCl_3$) spectrum of compound **19a**.

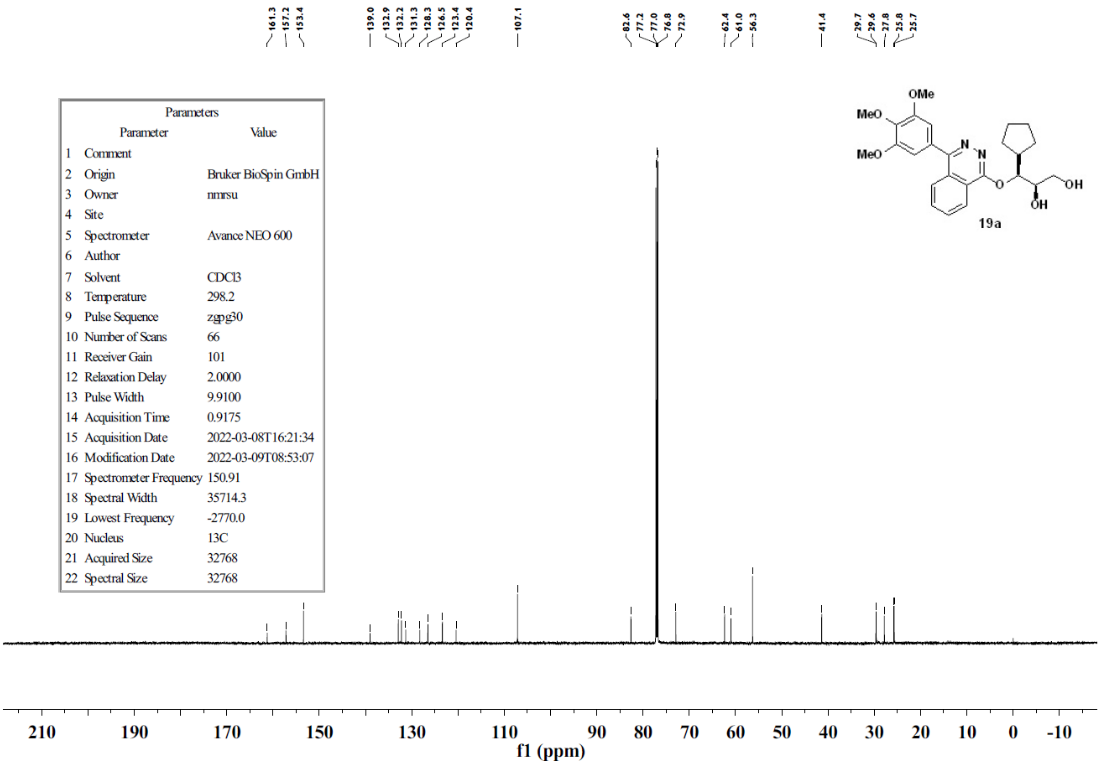

Supplementary Figure 292. ^{13}C NMR (151 MHz, $CDCl_3$) spectrum of compound **19a**.

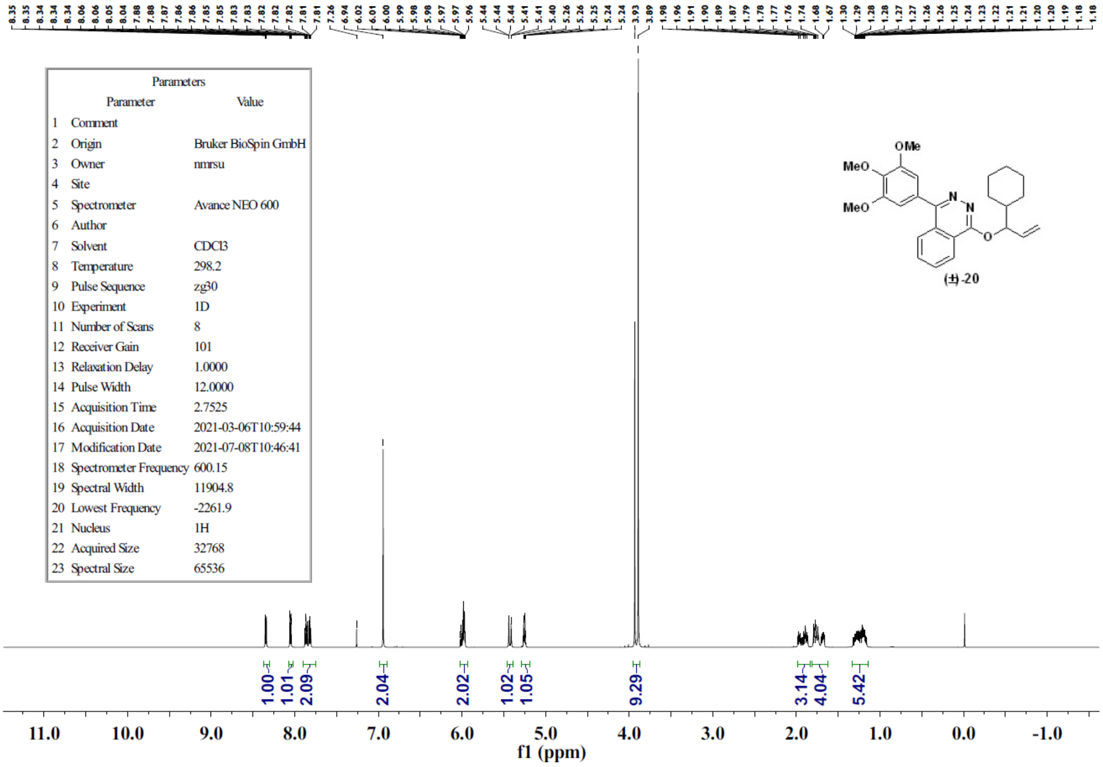

Supplementary Figure 293. ^{1}H NMR (600 MHz, $CDCl_3$) spectrum of compound **(±)-20**.

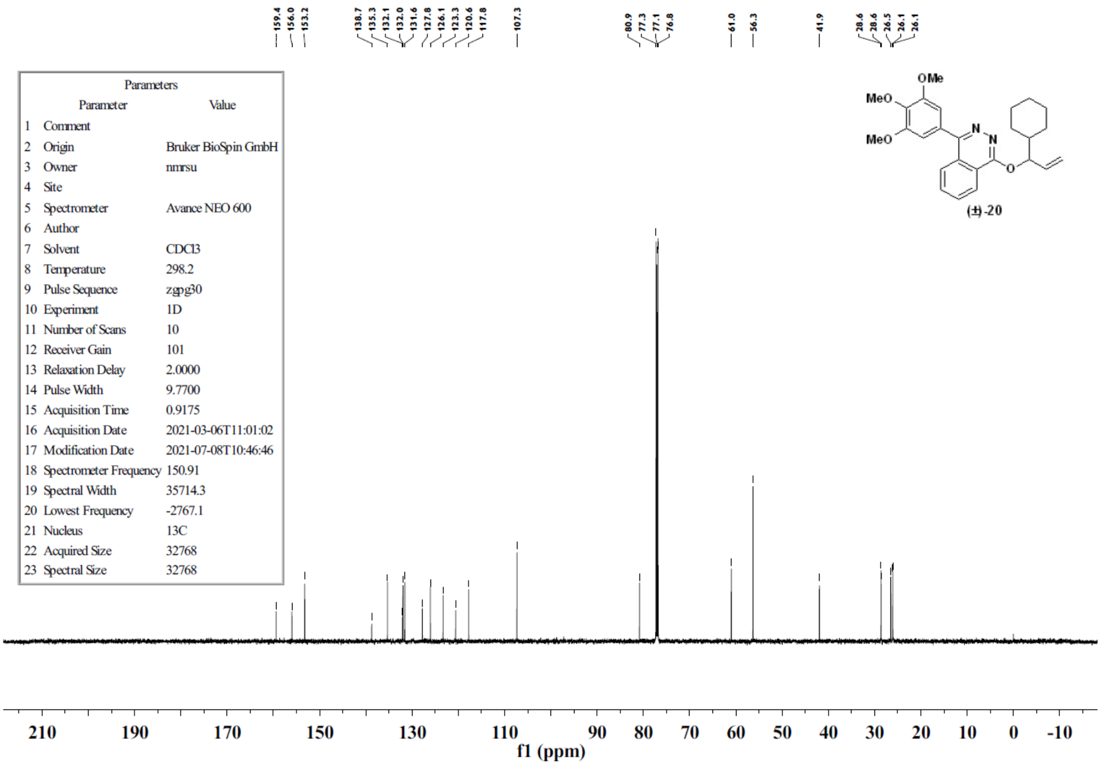

Supplementary Figure 294. ^{13}C NMR (151 MHz, $CDCl_3$) spectrum of compound **(±)-20**.

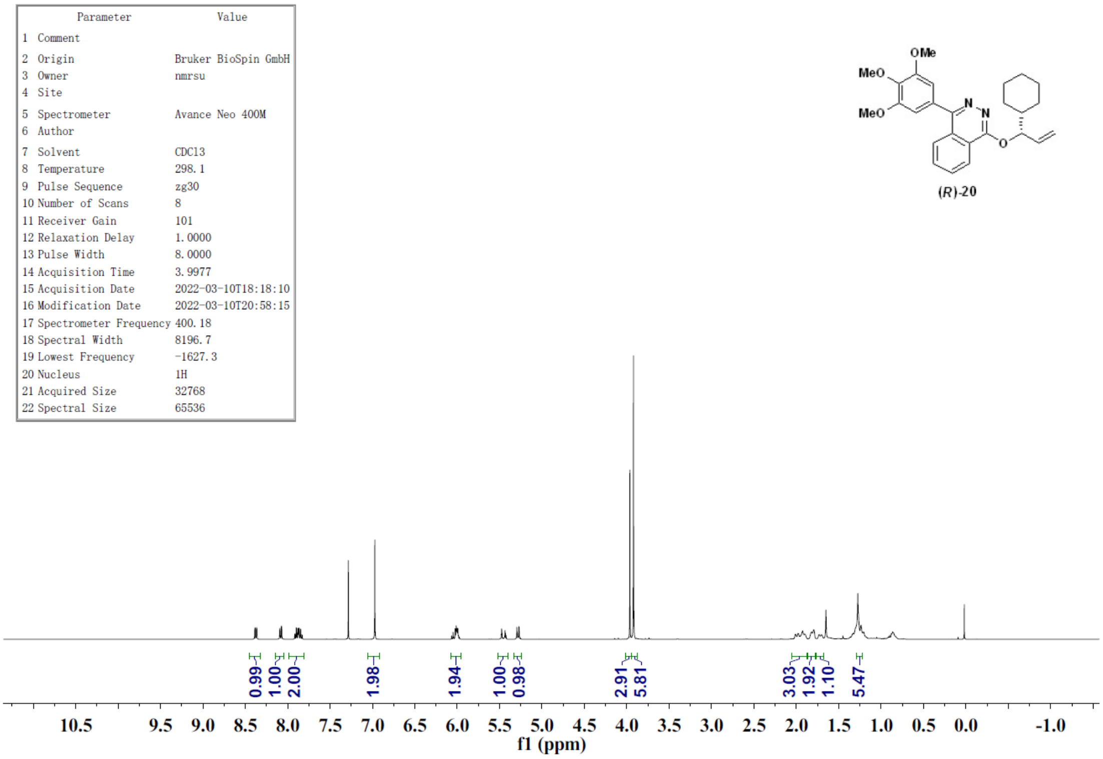

Supplementary Figure 295. ^{1}H NMR (400 MHz, $CDCl_3$) spectrum of compound ***(R)*-20**.

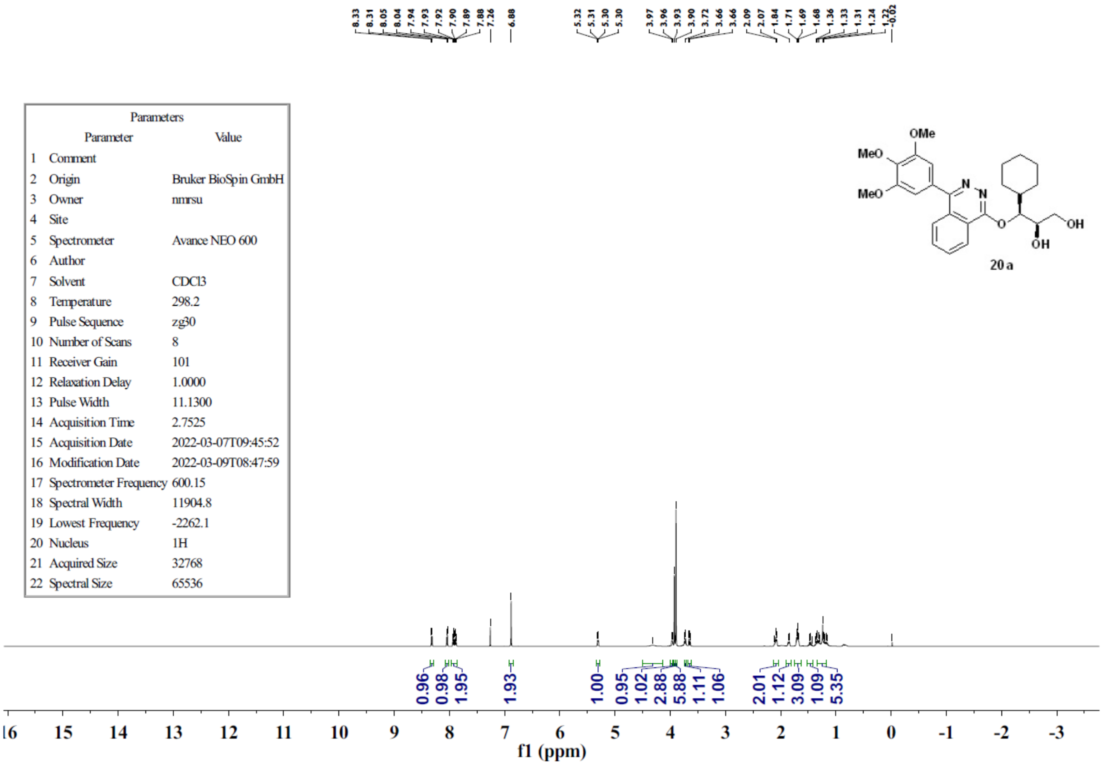

Parameters

	Parameter	Value
1	Comment	
2	Origin	Bruker BioSpin GmbH
3	Owner	nmrsu
4	Site	
5	Spectrometer	Avance NEO 600
6	Author	
7	Solvent	CDCl3
8	Temperature	298.2
9	Pulse Sequence	zg30
10	Number of Scans	8
11	Receiver Gain	101
12	Relaxation Delay	1.0000
13	Pulse Width	11.1300
14	Acquisition Time	2.7525
15	Acquisition Date	2022-03-07T09:45:52
16	Modification Date	2022-03-09T08:47:59
17	Spectrometer Frequency	600.15
18	Spectral Width	11904.8
19	Lowest Frequency	-2262.1
20	Nucleus	1H
21	Acquired Size	32768
22	Spectral Size	65536

Supplementary Figure 296. ^{1}H NMR (600 MHz, $CDCl_3$) spectrum of compound **20a**.

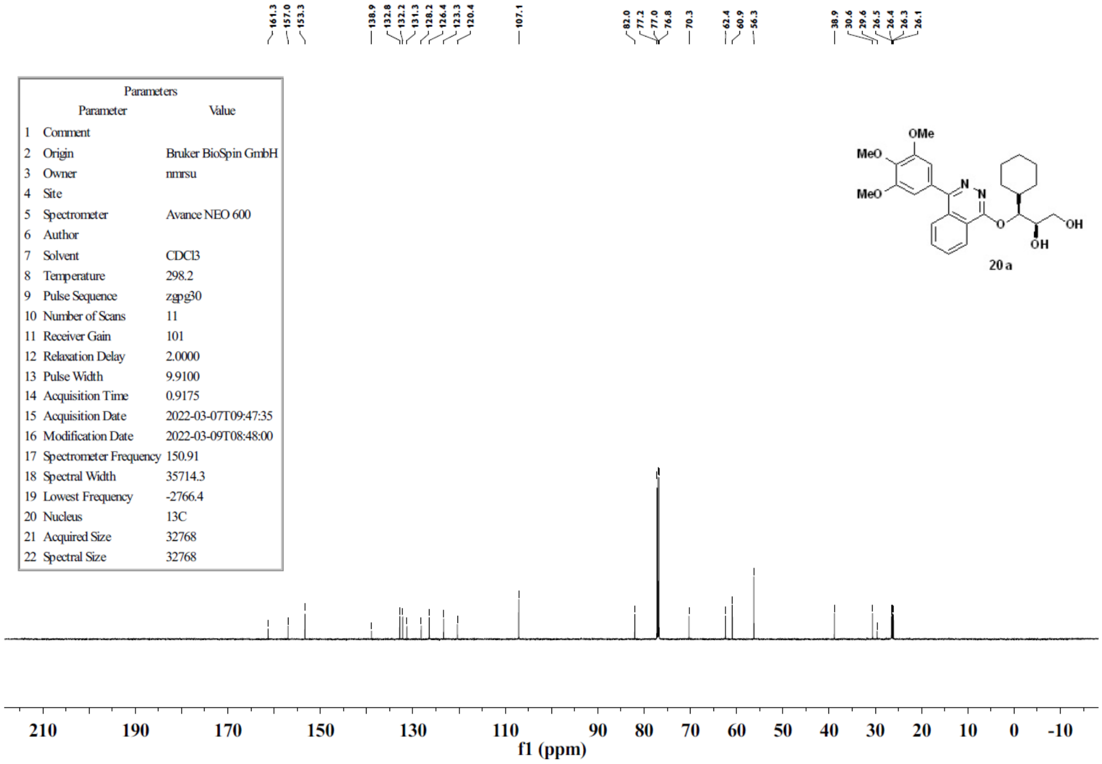

Parameters

	Parameter	Value
1	Comment	
2	Origin	Bruker BioSpin GmbH
3	Owner	nmrsu
4	Site	
5	Spectrometer	Avance NEO 600
6	Author	
7	Solvent	CDCl3
8	Temperature	298.2
9	Pulse Sequence	zgpg30
10	Number of Scans	11
11	Receiver Gain	101
12	Relaxation Delay	2.0000
13	Pulse Width	9.9100
14	Acquisition Time	0.9175
15	Acquisition Date	2022-03-07T09:47:35
16	Modification Date	2022-03-09T08:48:00
17	Spectrometer Frequency	150.91
18	Spectral Width	35714.3
19	Lowest Frequency	-2766.4
20	Nucleus	13C
21	Acquired Size	32768
22	Spectral Size	32768

Supplementary Figure 297. ^{13}C NMR (151 MHz, $CDCl_3$) spectrum of compound **20a**.

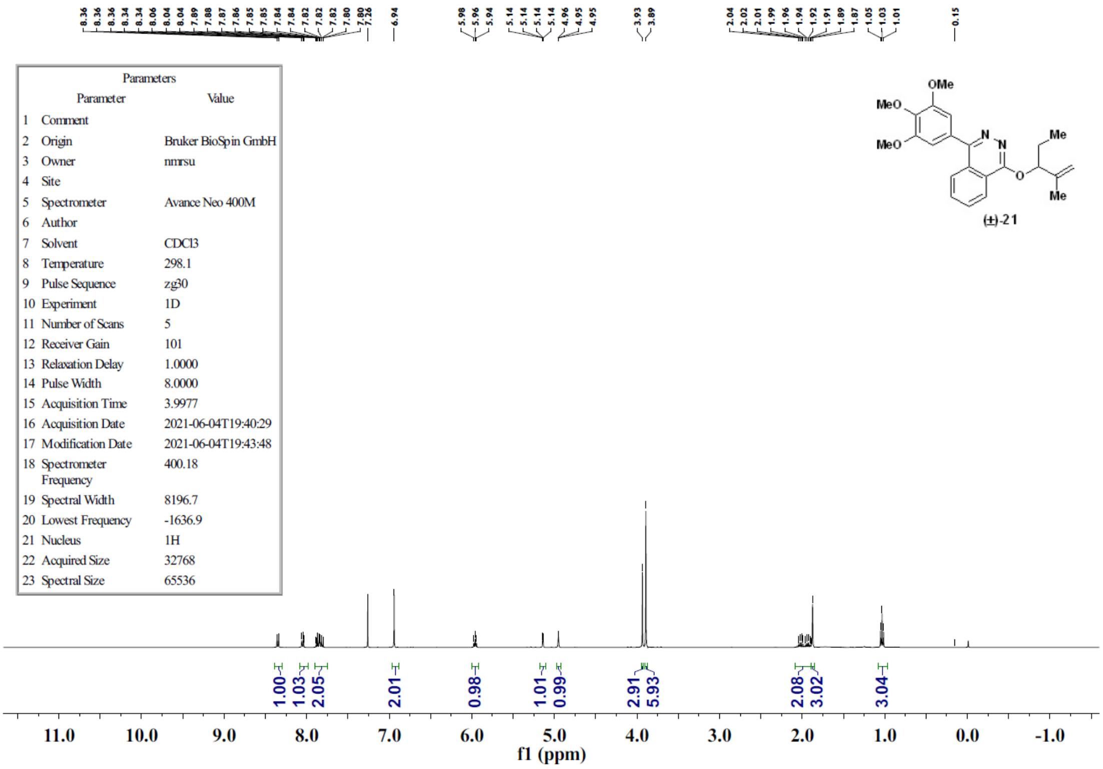

Supplementary Figure 298. ^{1}H NMR (400 MHz, $CDCl_3$) spectrum of compound **(±)-21**.

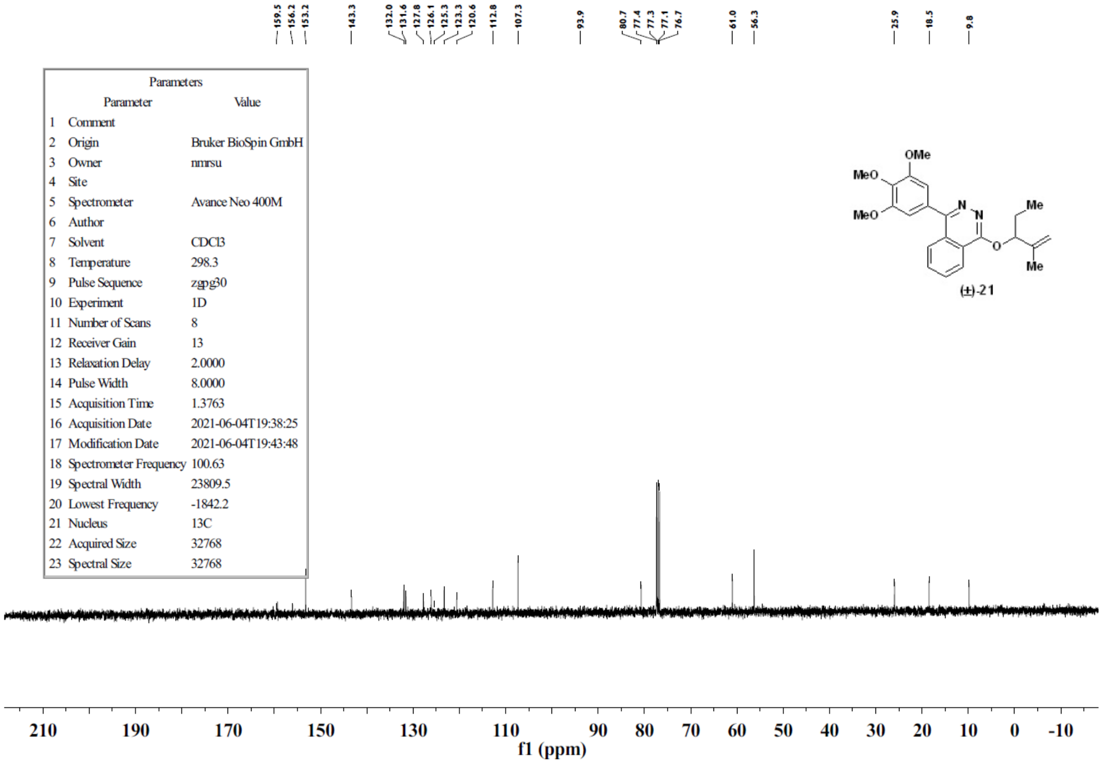

Supplementary Figure 299. ^{13}C NMR (101 MHz, $CDCl_3$) spectrum of compound **(±)-21**.

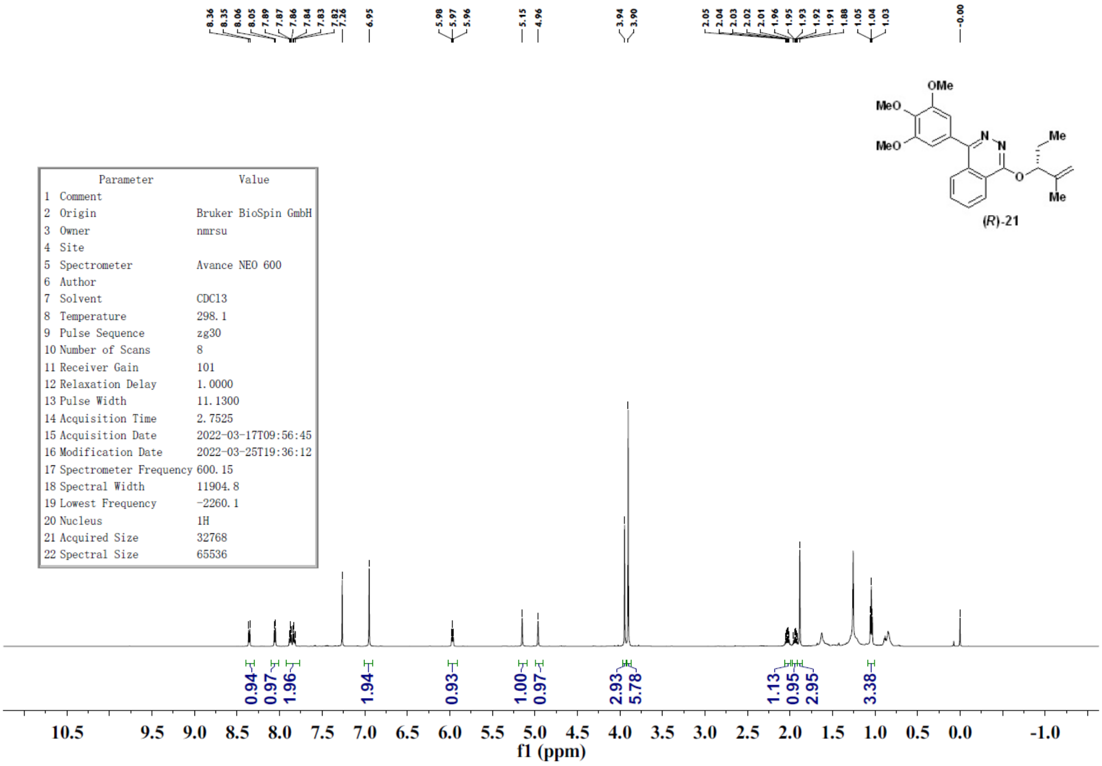

Supplementary Figure 300. ^{1}H NMR (600 MHz, $CDCl_3$) spectrum of compound ***(R)*-21**.

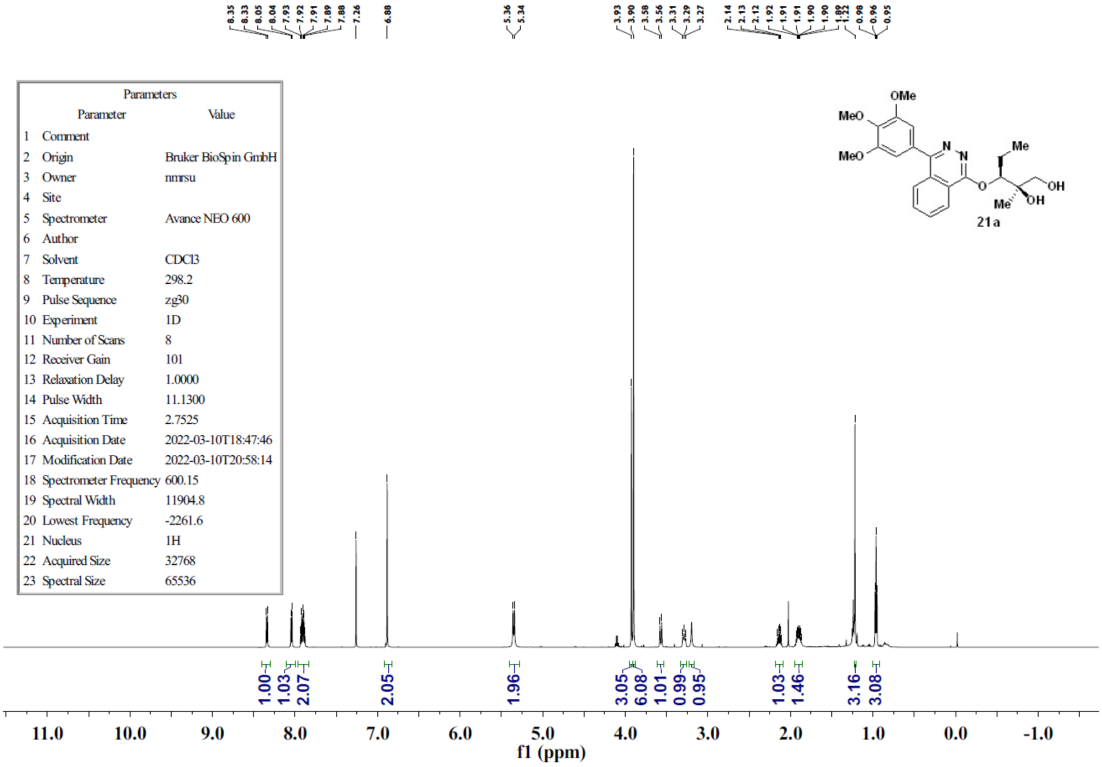

Supplementary Figure 301. ^{1}H NMR (600 MHz, $CDCl_3$) spectrum of compound **21a**.

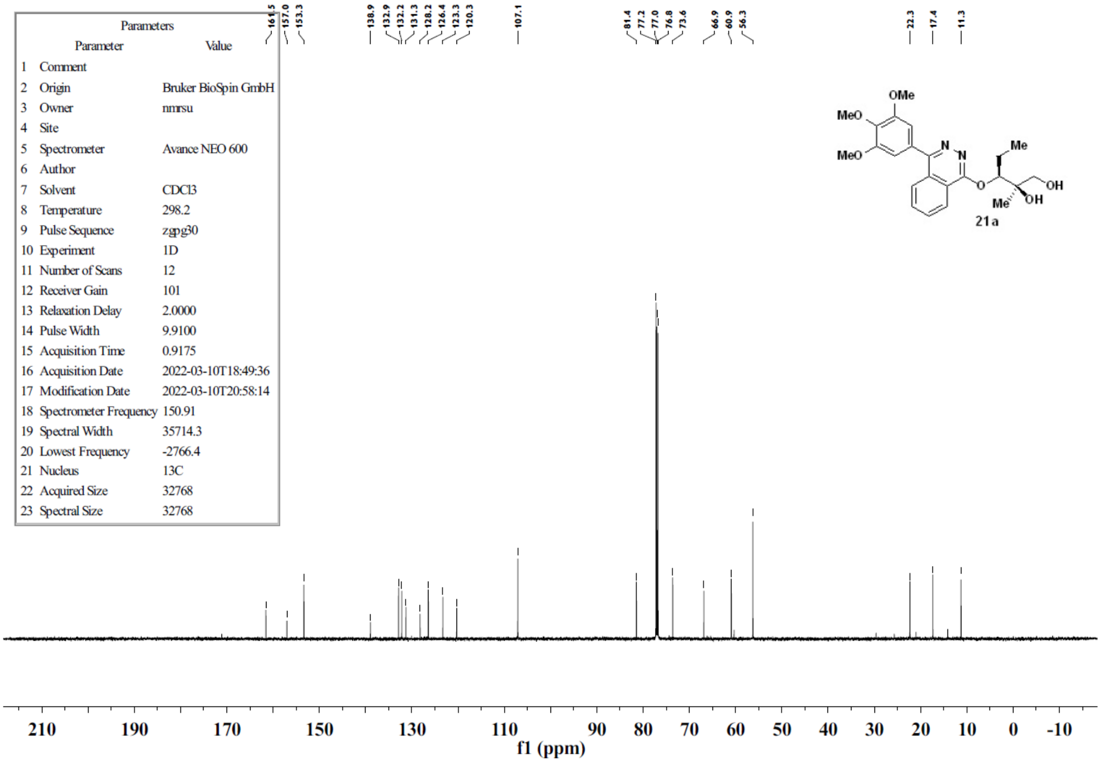

Supplementary Figure 302. ^{13}C NMR (151 MHz, $CDCl_3$) spectrum of compound **21a**.

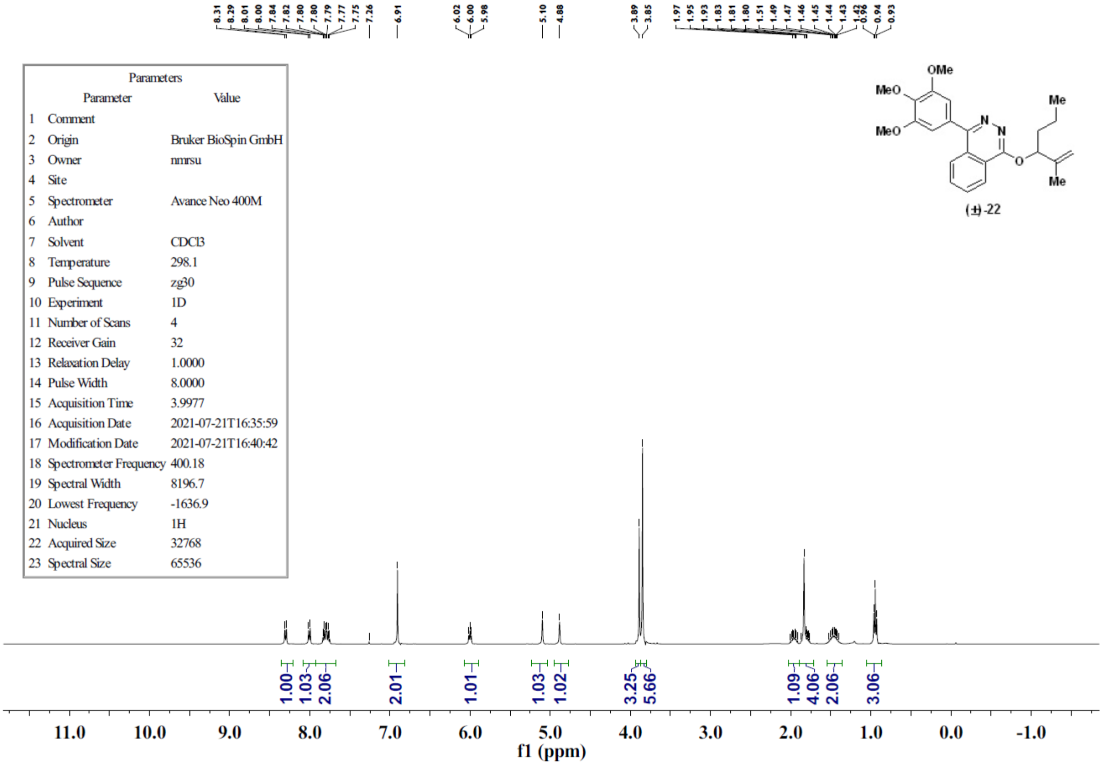

Supplementary Figure 303. ^{1}H NMR (600 MHz, $CDCl_3$) spectrum of compound **(±)-22**.

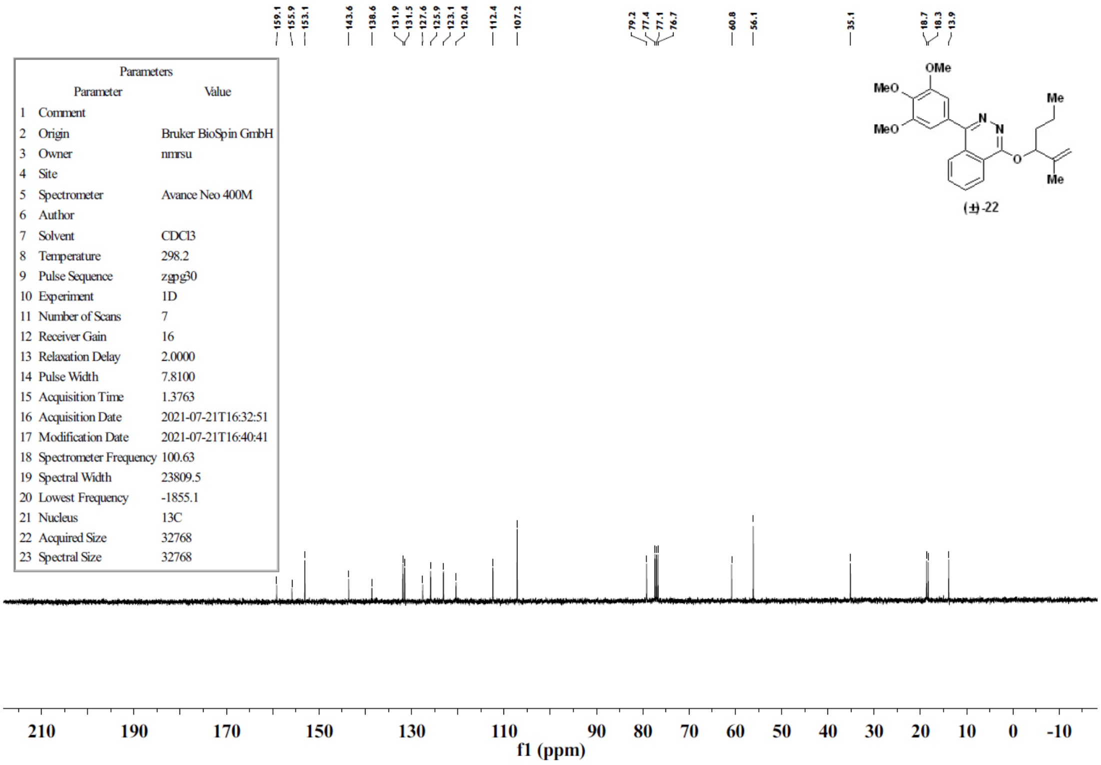

Supplementary Figure 304. ^{13}C NMR (101 MHz, $CDCl_3$) spectrum of compound **(±)-22**.

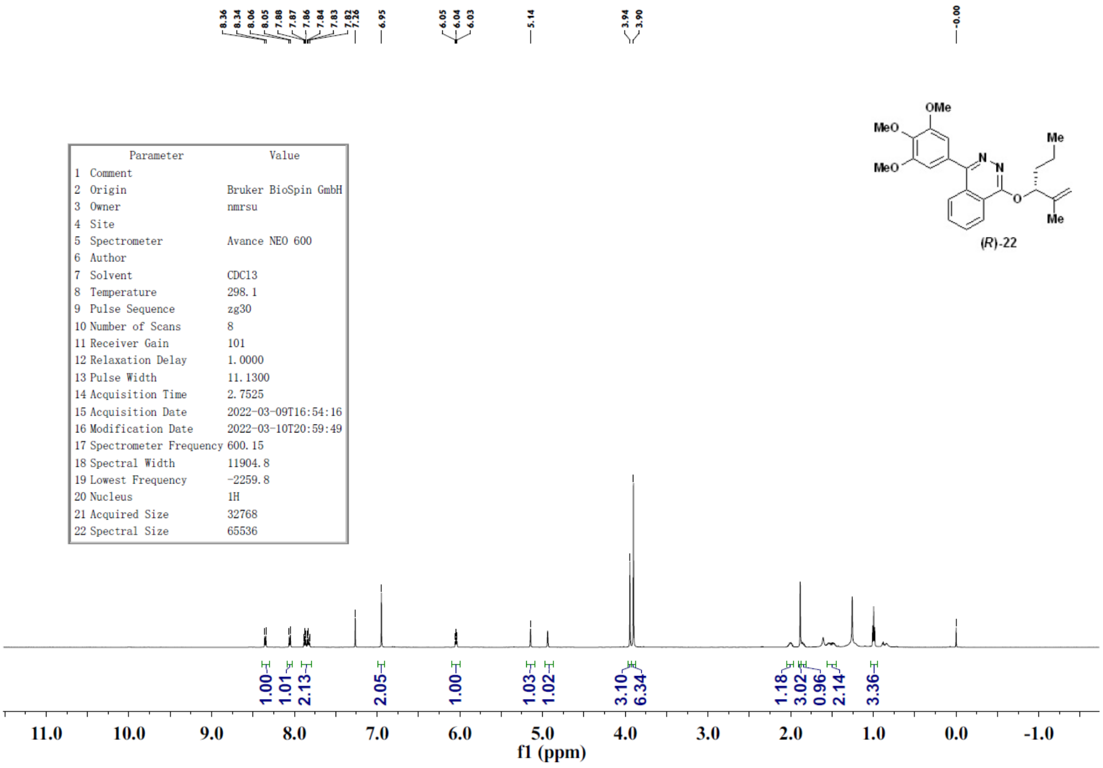

Supplementary Figure 305. ^{1}H NMR (600 MHz, $CDCl_3$) spectrum of compound **(*R*)-22**.

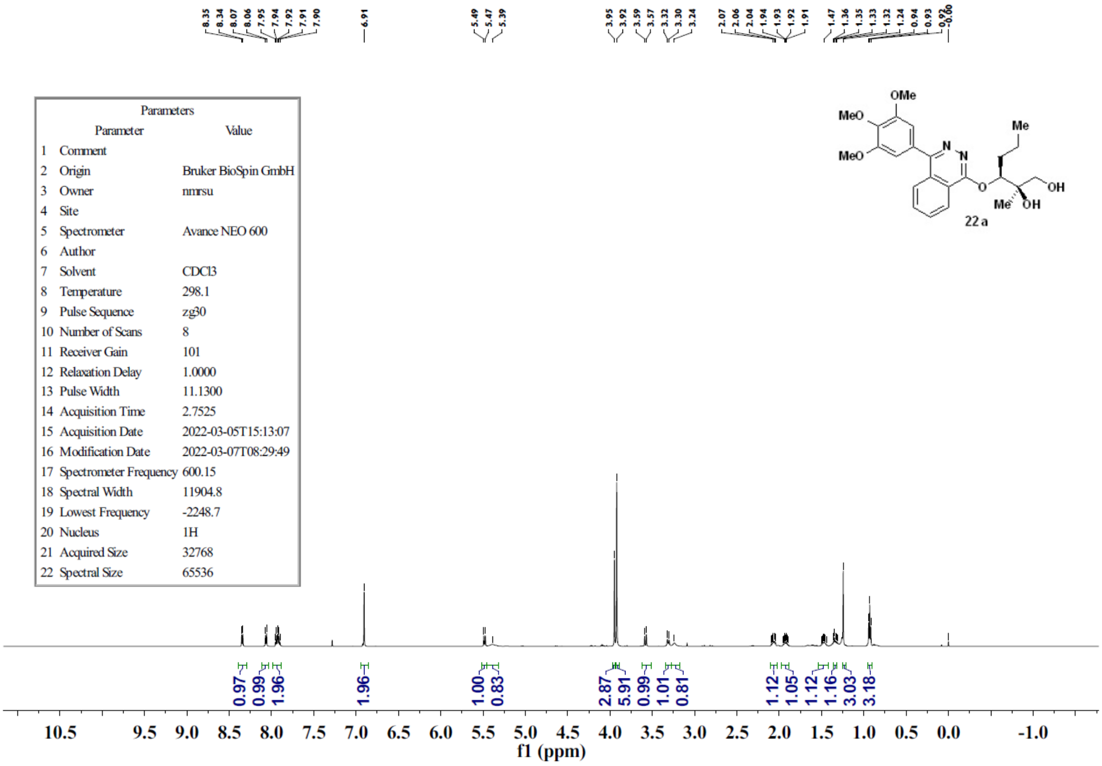

Supplementary Figure 306. ^{1}H NMR (600 MHz, $CDCl_3$) spectrum of compound **22a**.

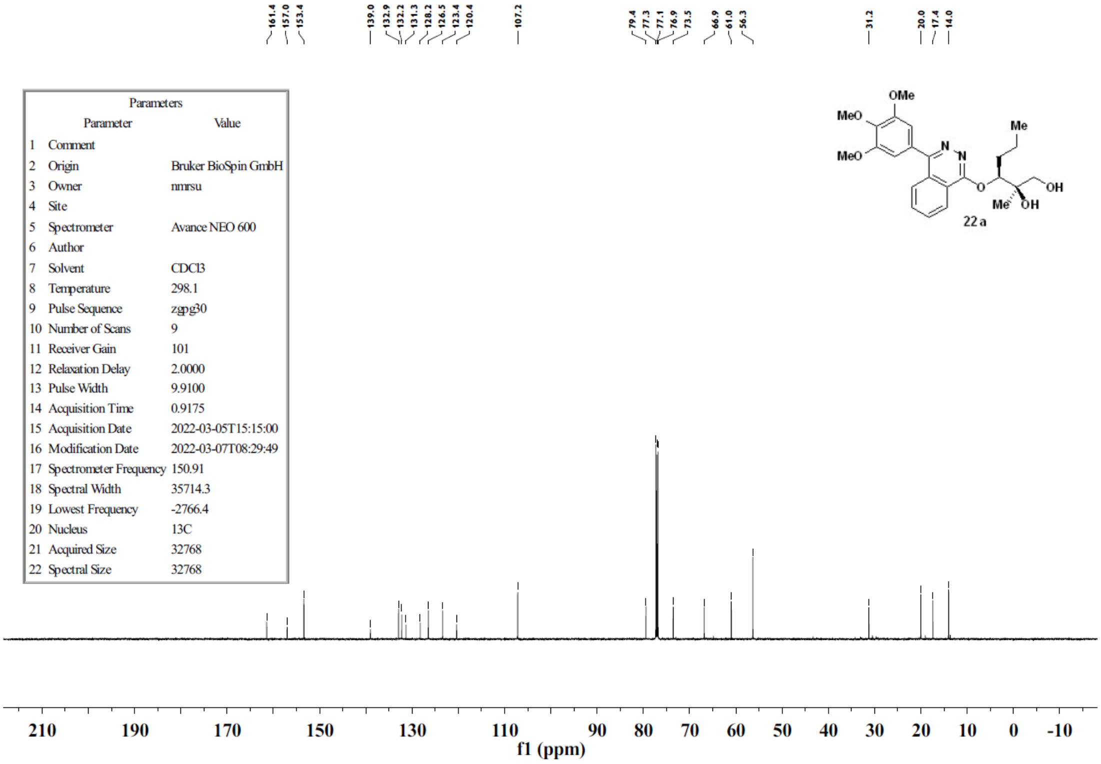

Supplementary Figure 307. ^{13}C NMR (151 MHz, $CDCl_3$) spectrum of compound **22a**.

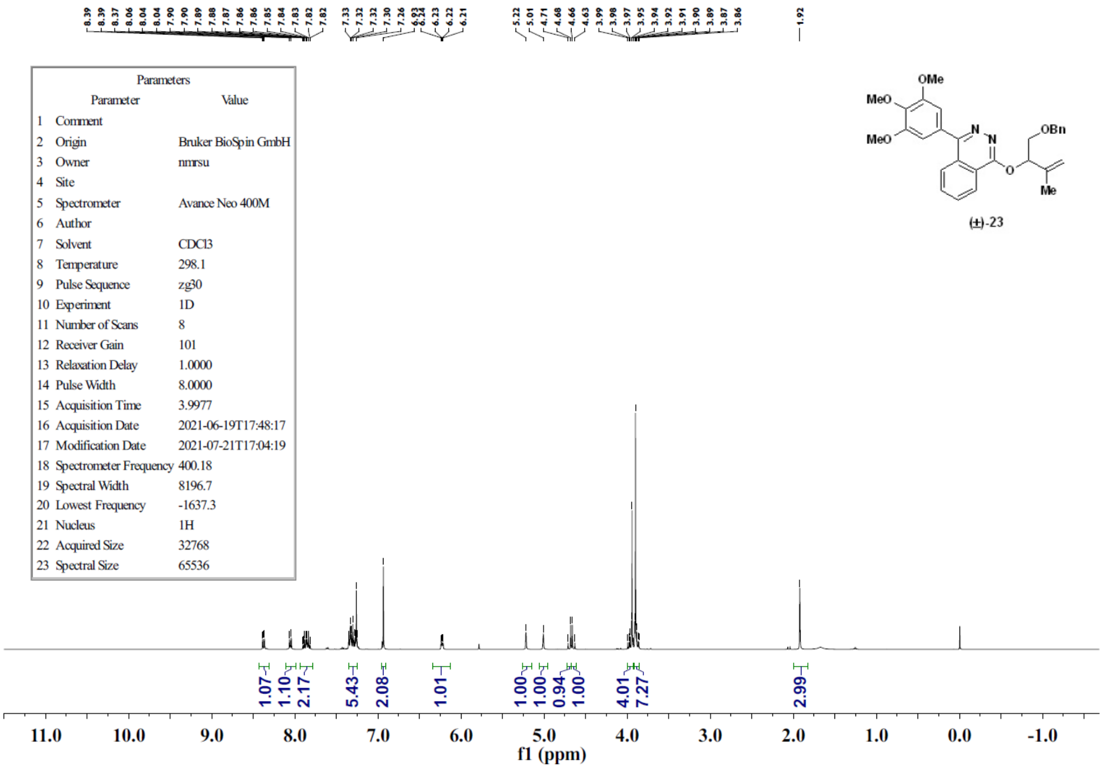

Supplementary Figure 308. ^{1}H NMR (600 MHz, $CDCl_3$) spectrum of compound **(±)-23**.

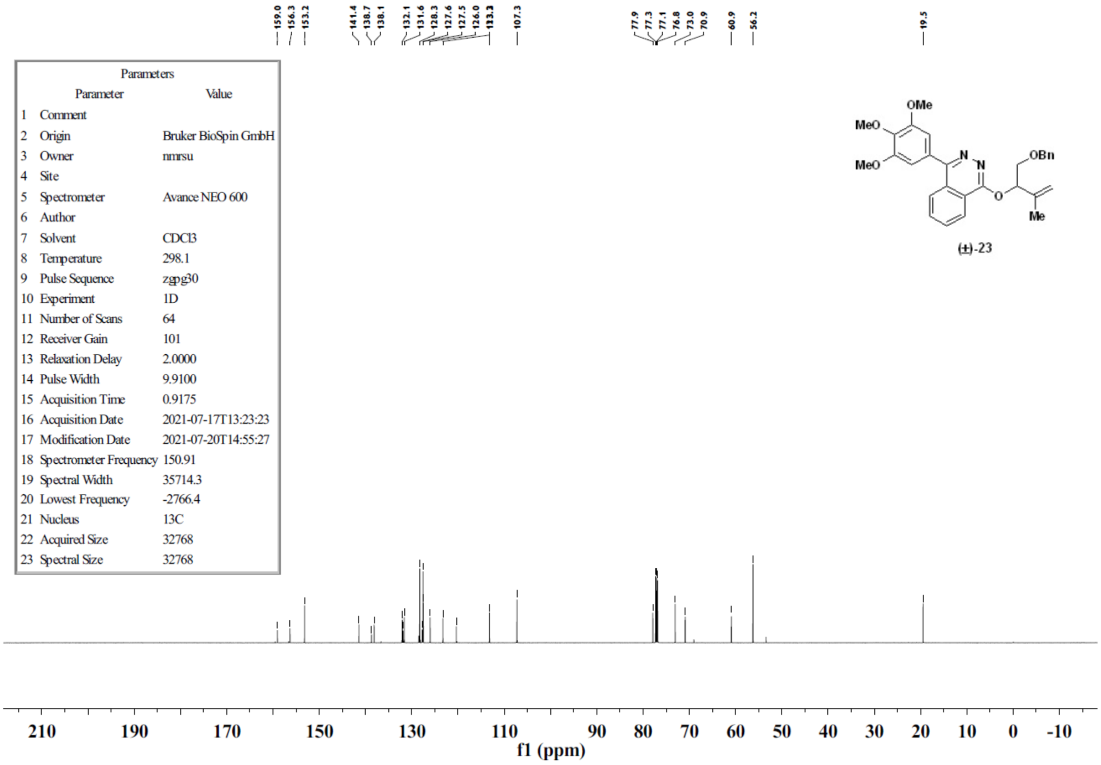

Supplementary Figure 309. ^{13}C NMR (101 MHz, $CDCl_3$) spectrum of compound **(±)-23**.

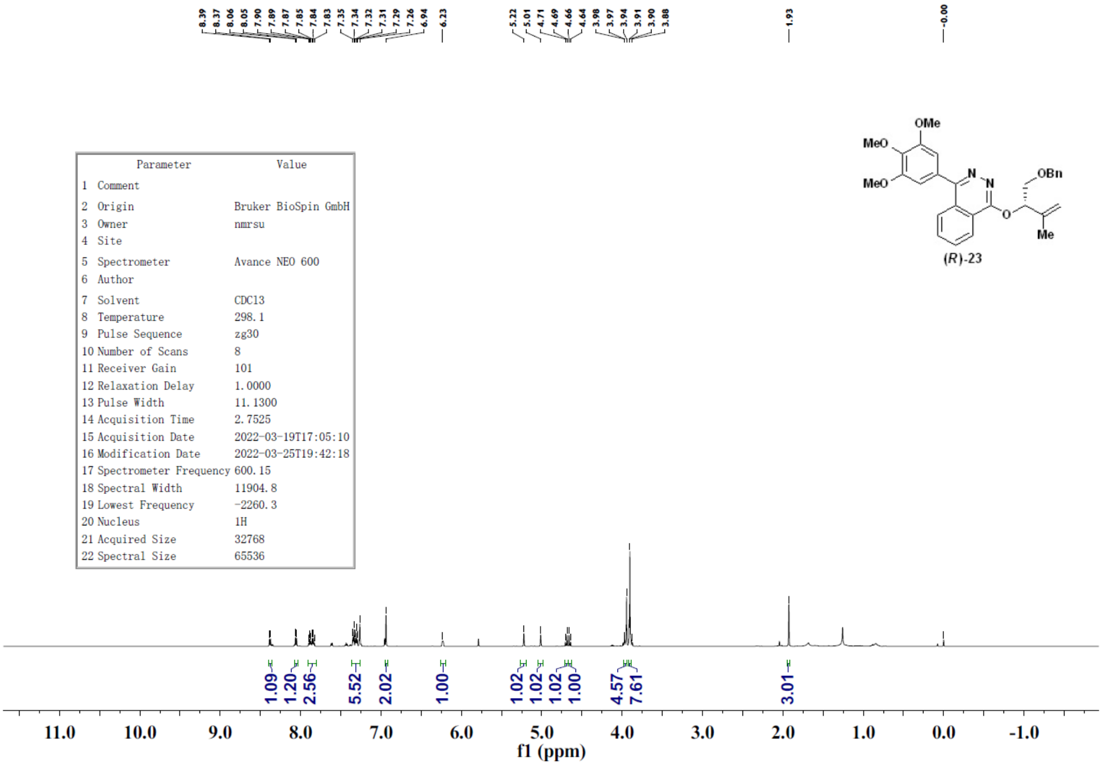

Supplementary Figure 310. ^{1}H NMR (600 MHz, $CDCl_3$) spectrum of compound ***(R)*-23**.

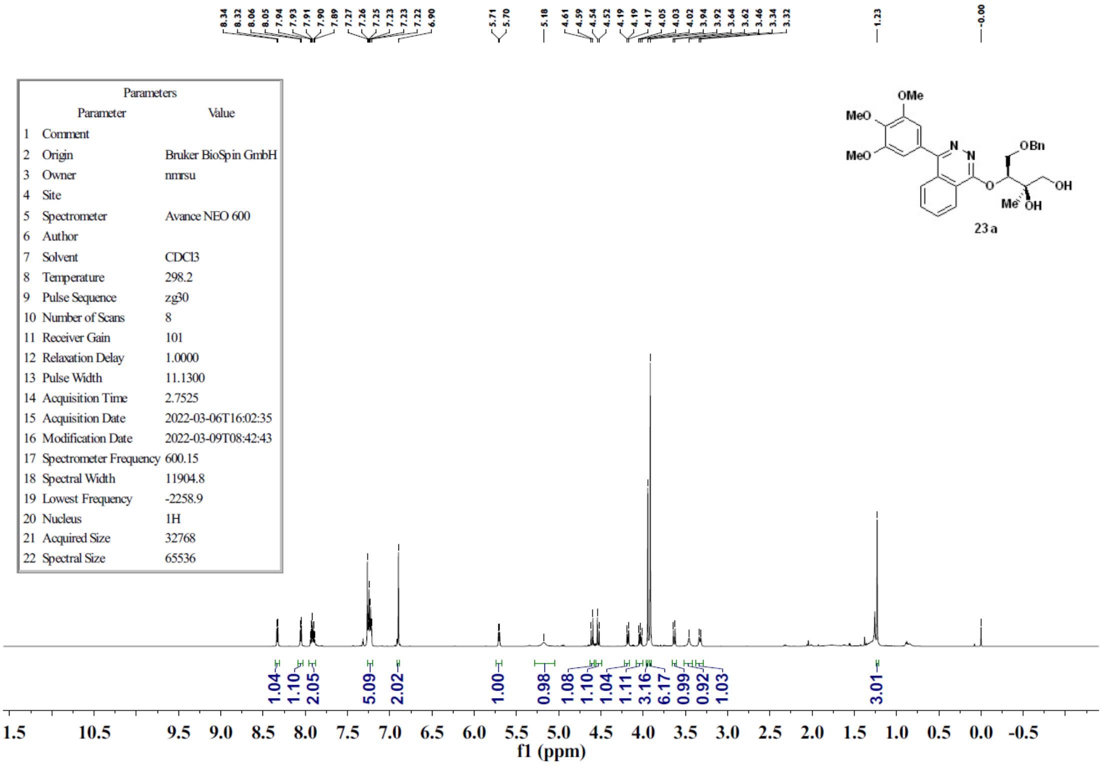

Supplementary Figure 311. ^{1}H NMR (600 MHz, $CDCl_3$) spectrum of compound **23a**.

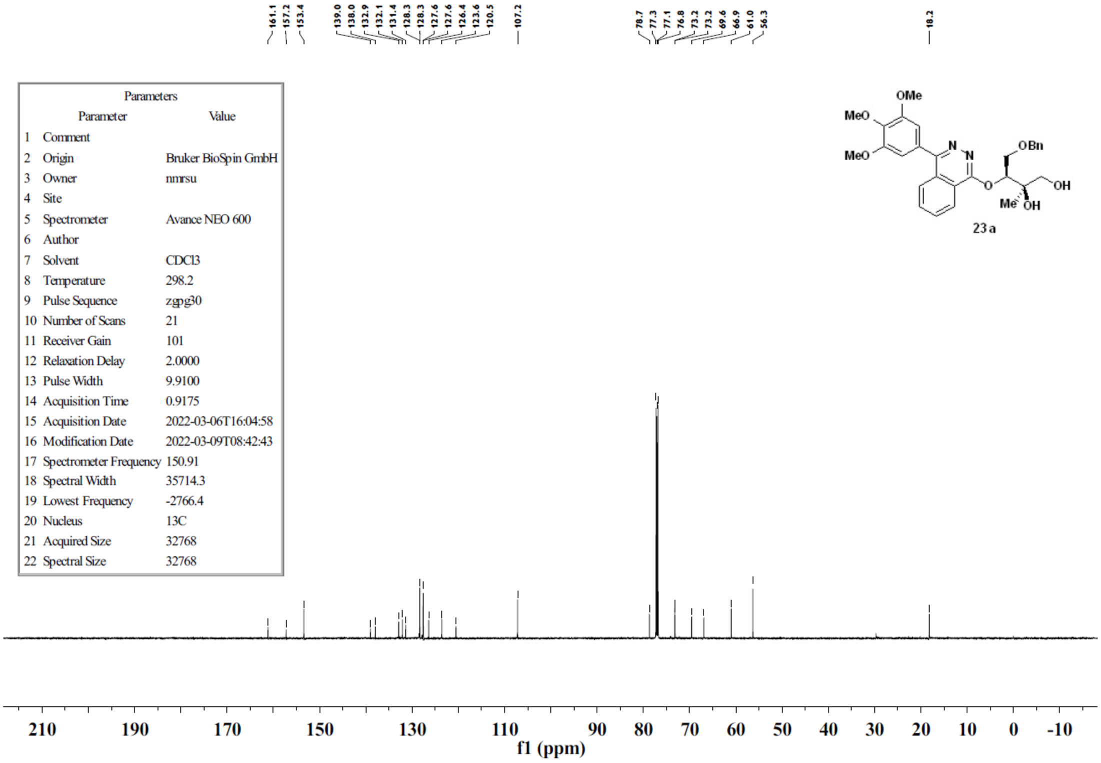

Supplementary Figure 312. ^{13}C NMR (151 MHz, $CDCl_3$) spectrum of compound **23a**.

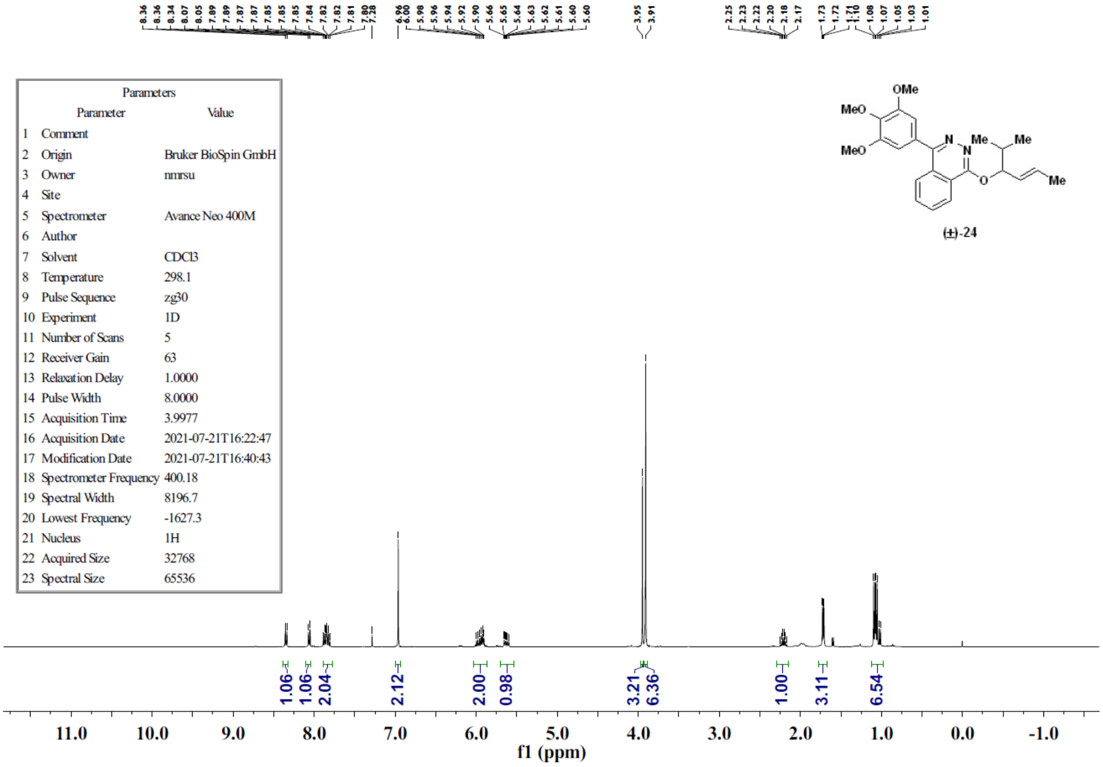

Supplementary Figure 313. ^{1}H NMR (400 MHz, $CDCl_3$) spectrum of compound **(±)-24**.

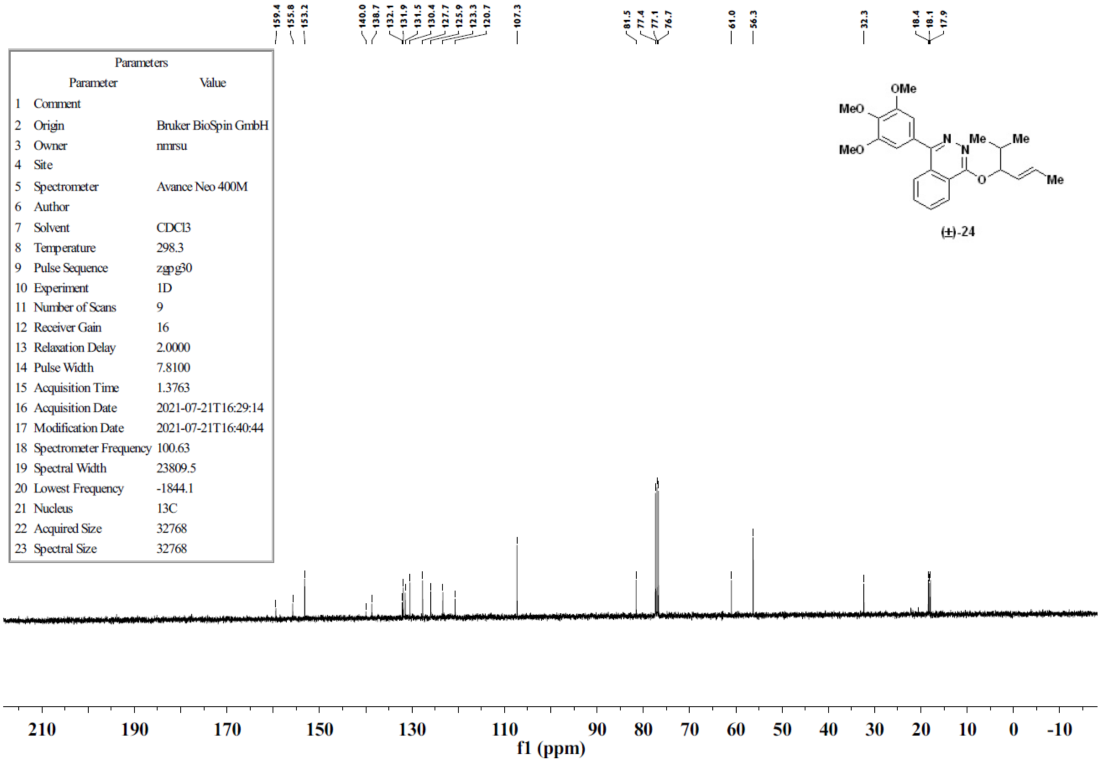

Supplementary Figure 314. ^{13}C NMR (101 MHz, $CDCl_3$) spectrum of compound **(±)-24**.

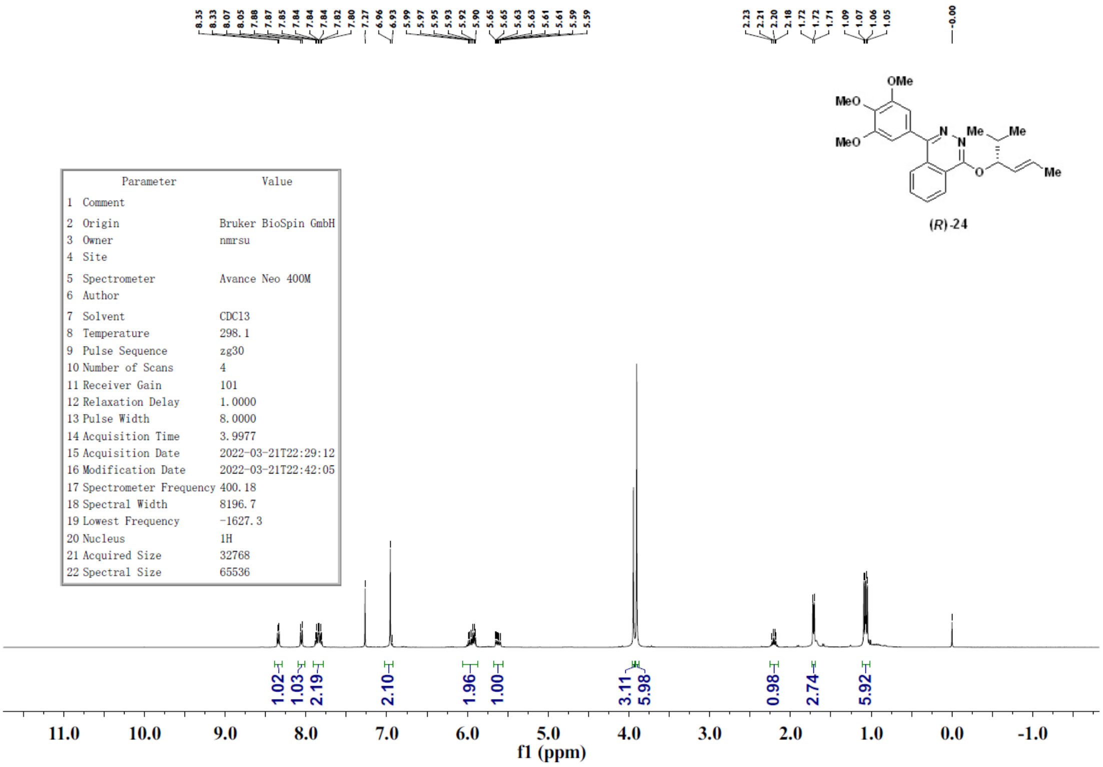

Supplementary Figure 315. ^{1}H NMR (400 MHz, $CDCl_3$) spectrum of compound ***(R)*-24**.

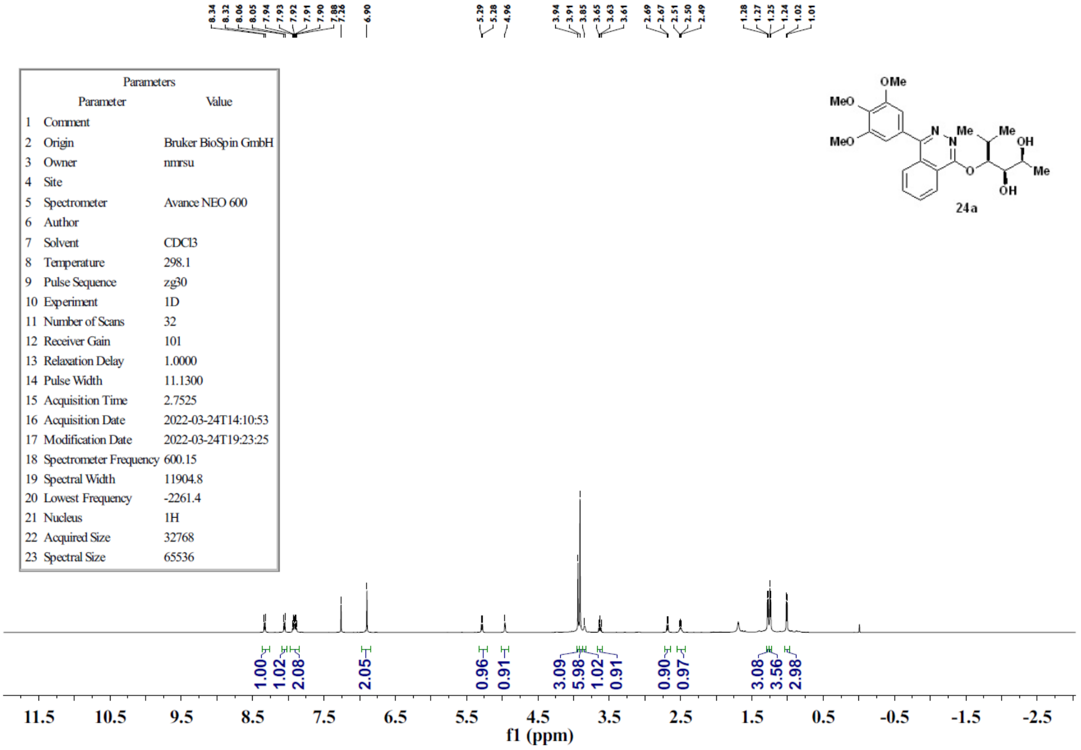

Supplementary Figure 316. ^{1}H NMR (600 MHz, $CDCl_3$) spectrum of compound **24a**.

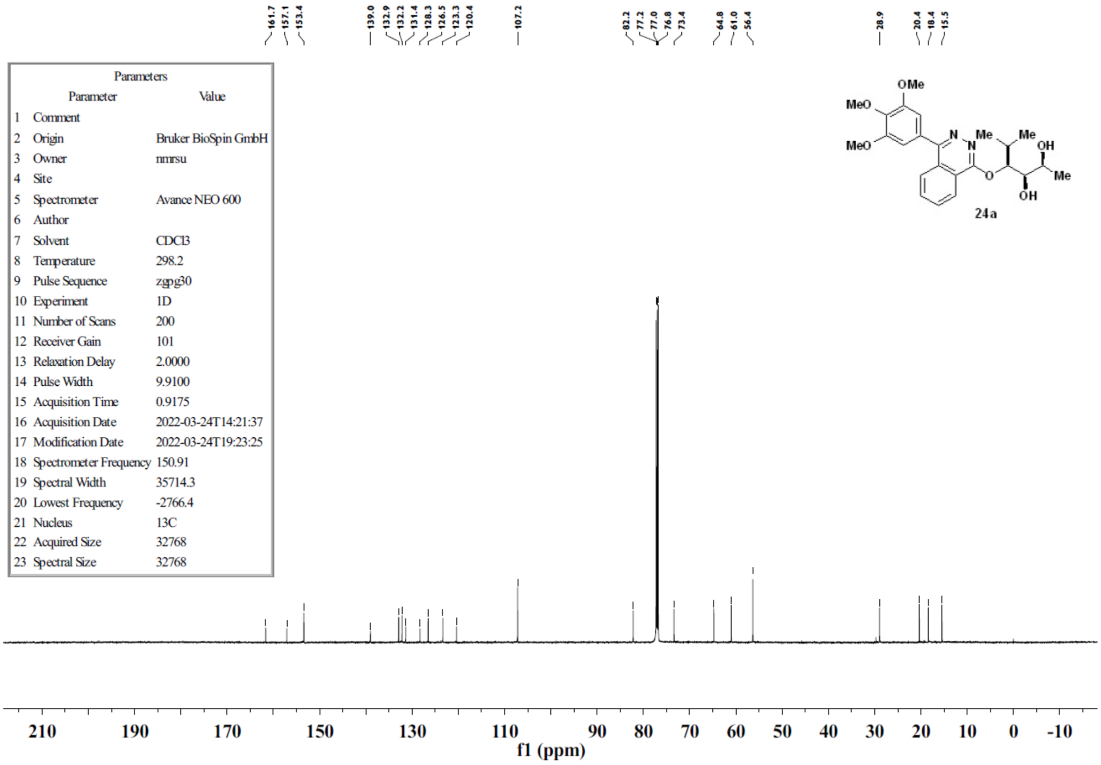

Supplementary Figure 317. ^{13}C NMR (151 MHz, $CDCl_3$) spectrum of compound **24a**.

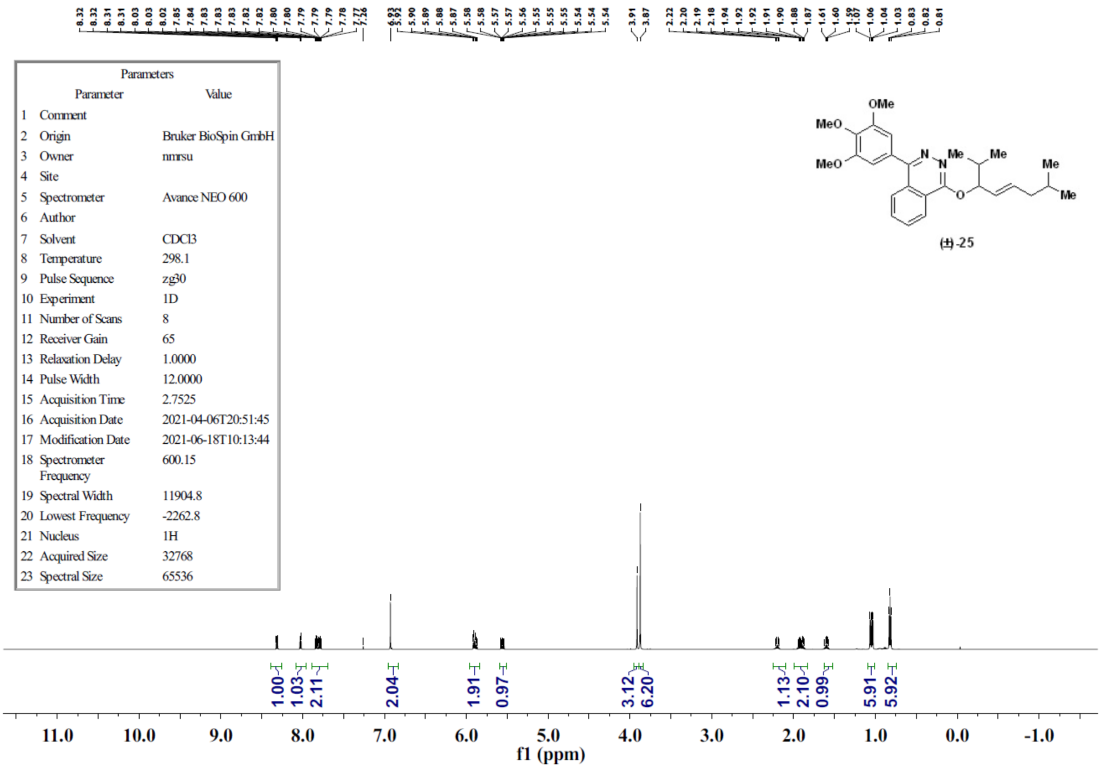

Supplementary Figure 318. ^{1}H NMR (600 MHz, $CDCl_3$) spectrum of compound **(±)-25**.

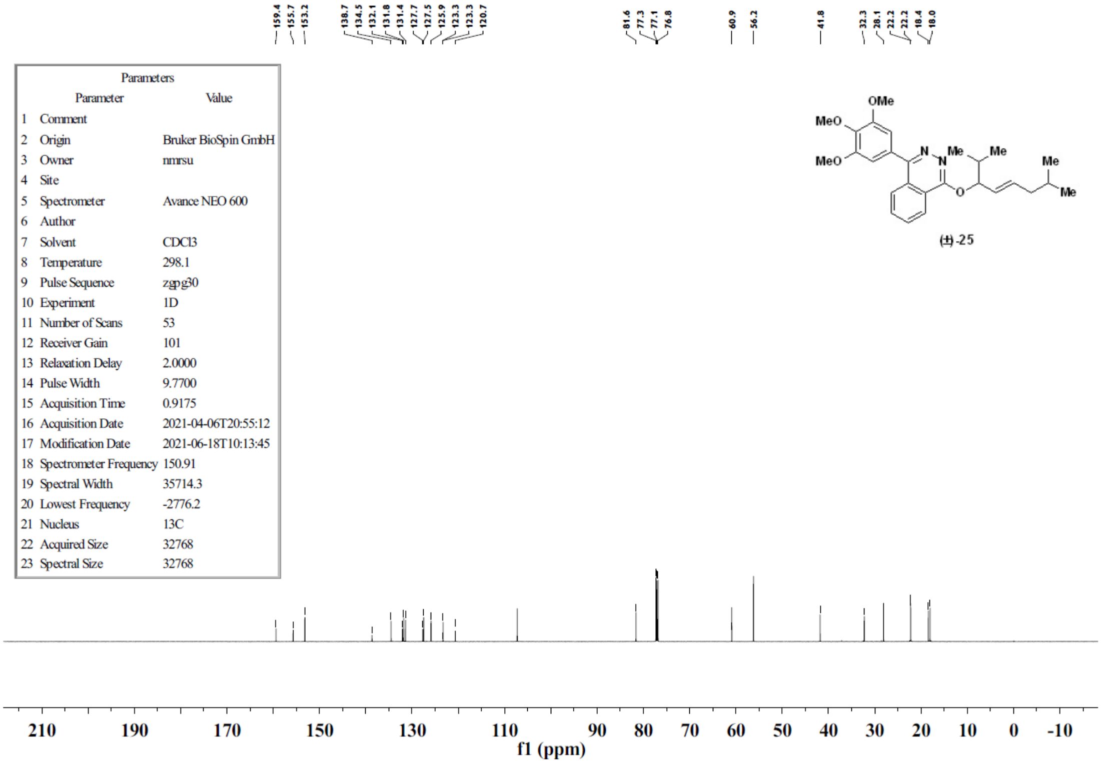

Supplementary Figure 319. ^{13}C NMR (151 MHz, $CDCl_3$) spectrum of compound **(±)-25**.

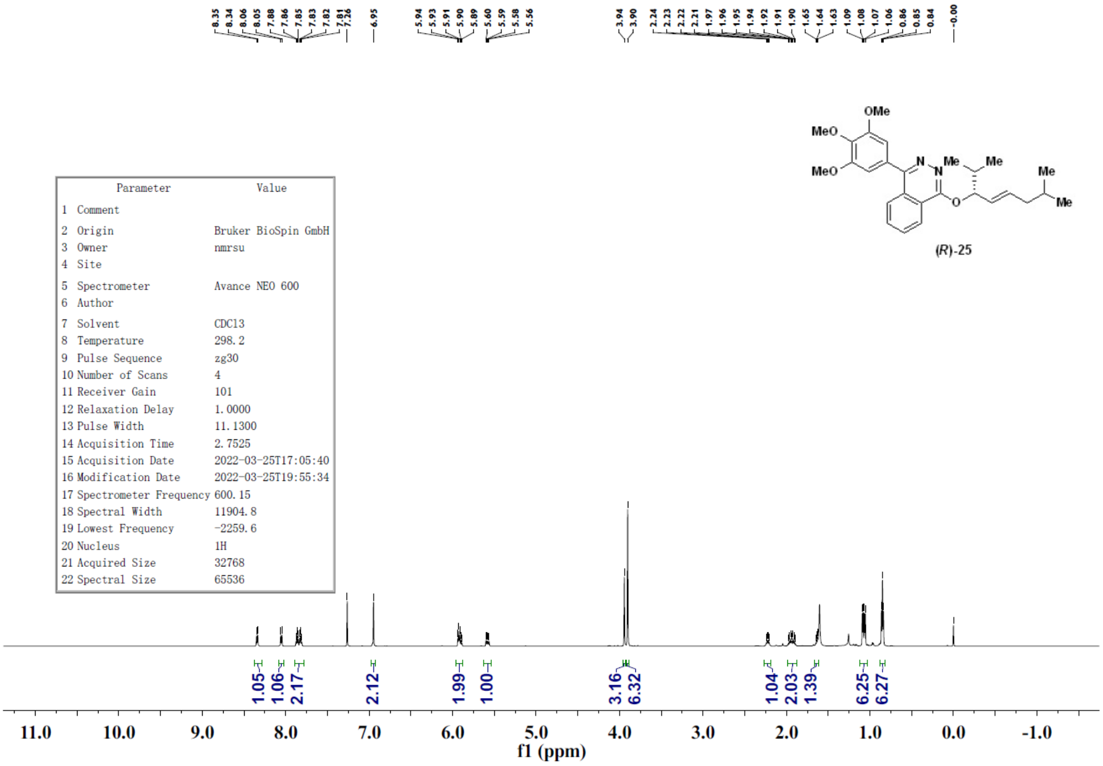

Supplementary Figure 320. ^{1}H NMR (600 MHz, $CDCl_3$) spectrum of compound **(*R*)-25**.

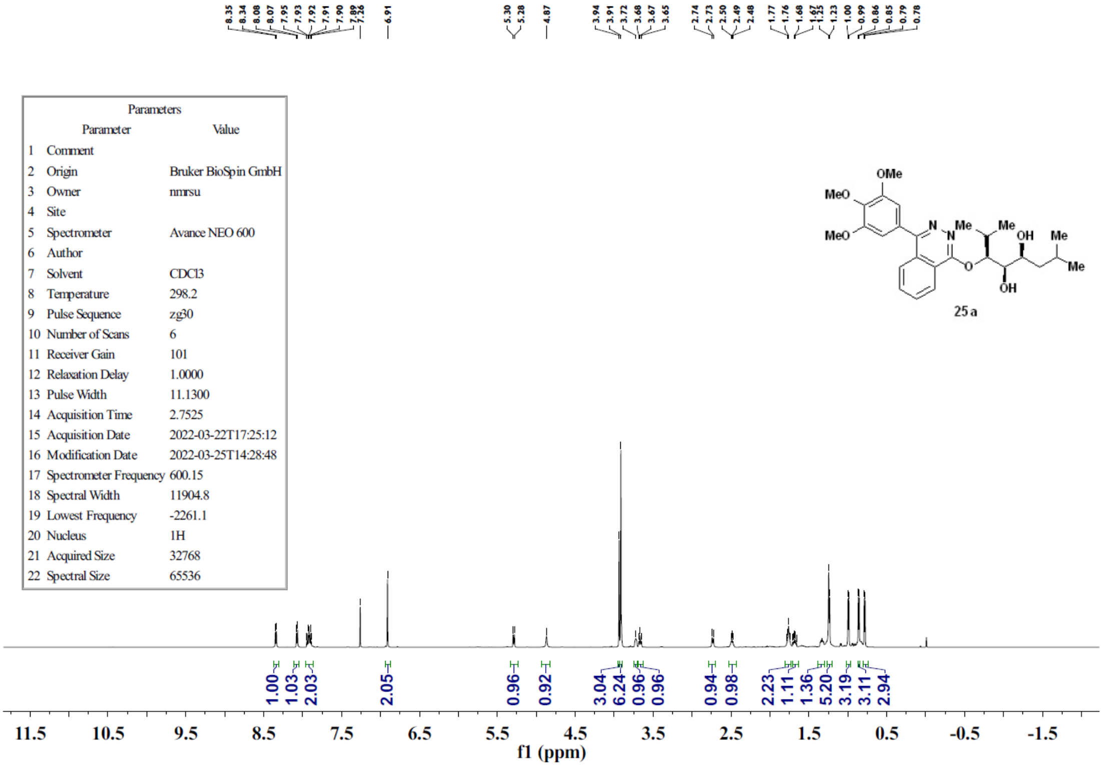

Supplementary Figure 321. ^{1}H NMR (600 MHz, $CDCl_3$) spectrum of compound **25a**.

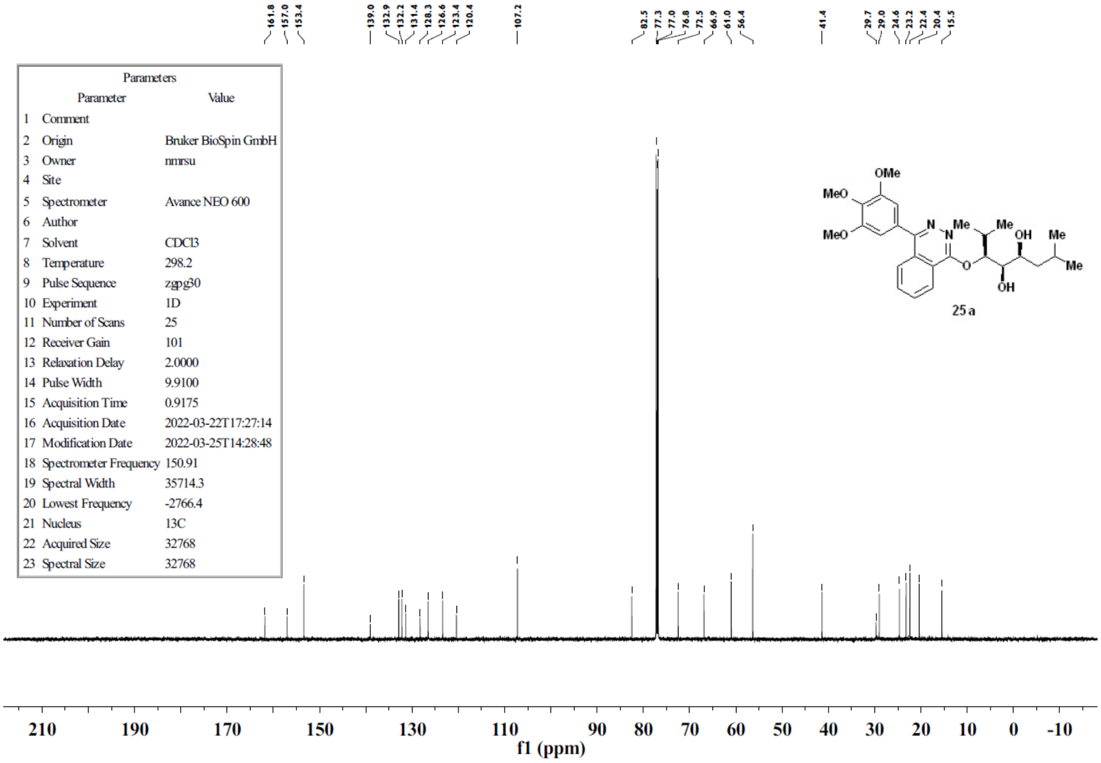

Supplementary Figure 322. ^{13}C NMR (151 MHz, $CDCl_3$) spectrum of compound **25a**.

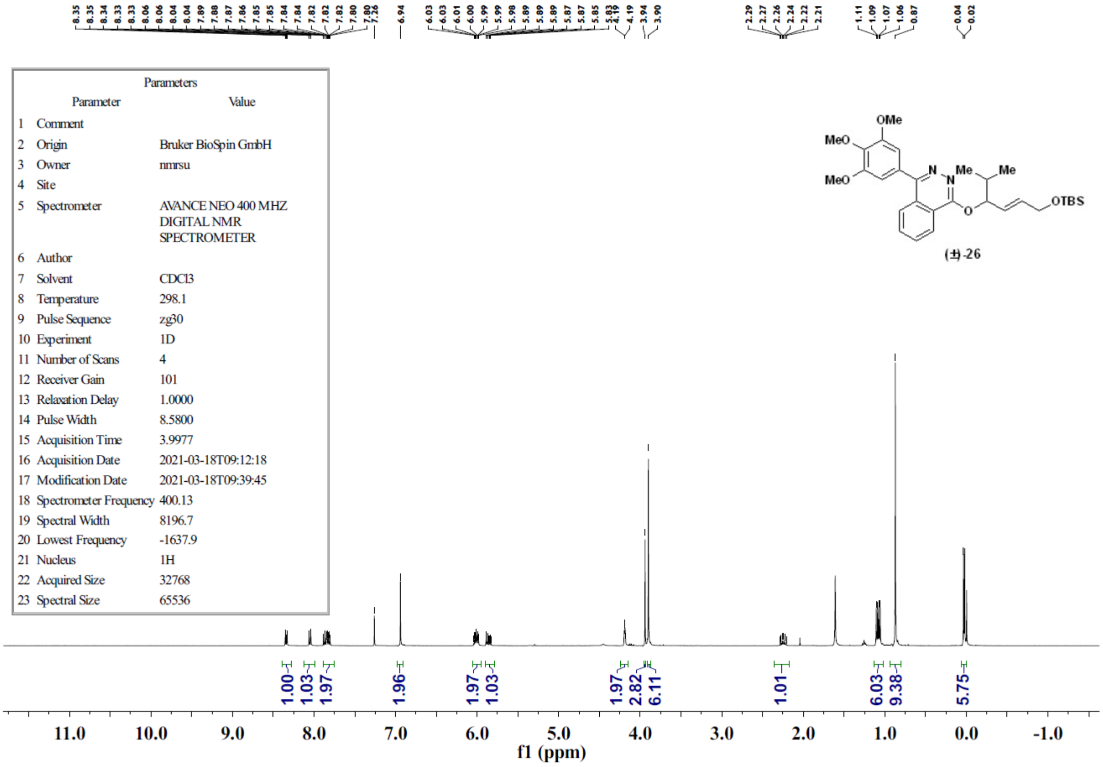

Supplementary Figure 323. ^{1}H NMR (400 MHz, $CDCl_3$) spectrum of compound **(±)-26**.

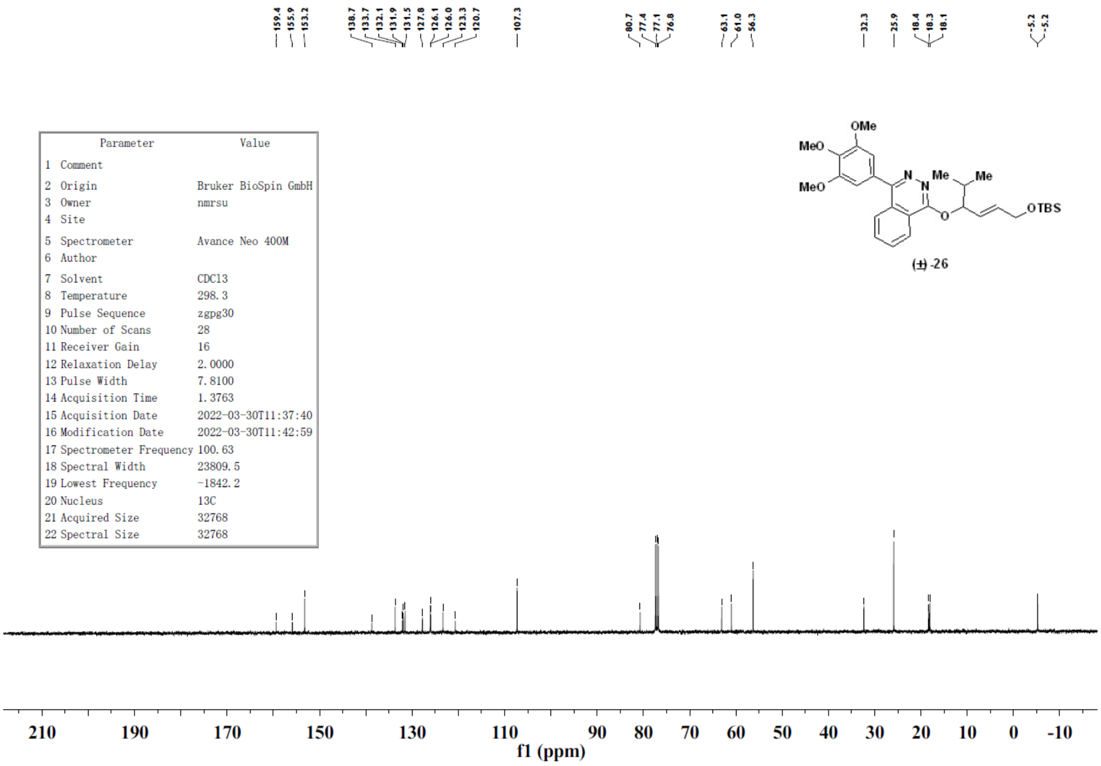

Supplementary Figure 324. ^{13}C NMR (101 MHz, $CDCl_3$) spectrum of compound **(±)-26**.

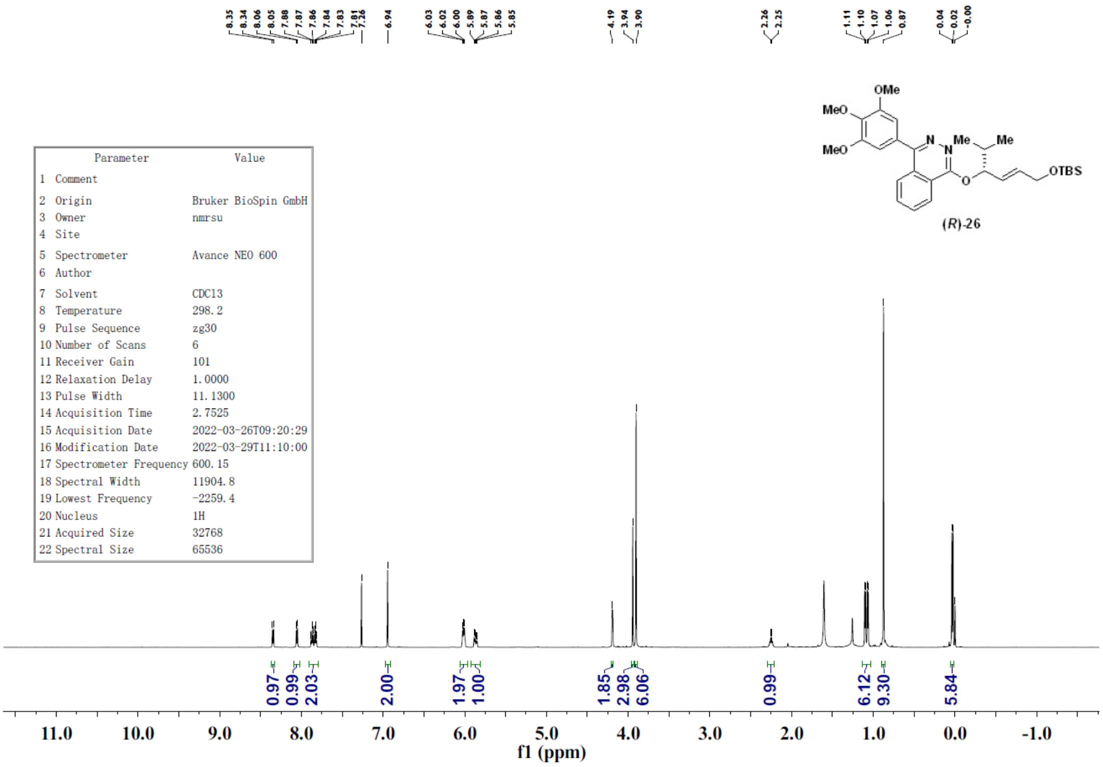

Supplementary Figure 325. ^{1}H NMR (600 MHz, $CDCl_3$) spectrum of compound **(*R*)-26**.

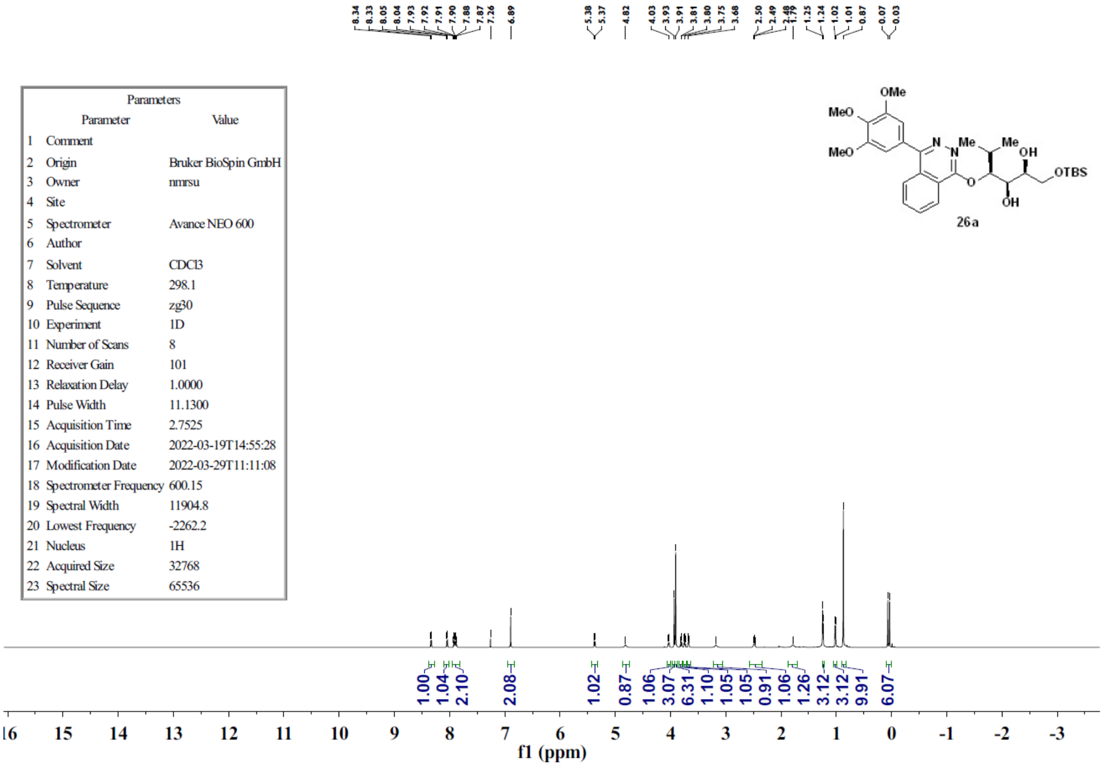

Supplementary Figure 326. ^{1}H NMR (600 MHz, $CDCl_3$) spectrum of compound **26a**.

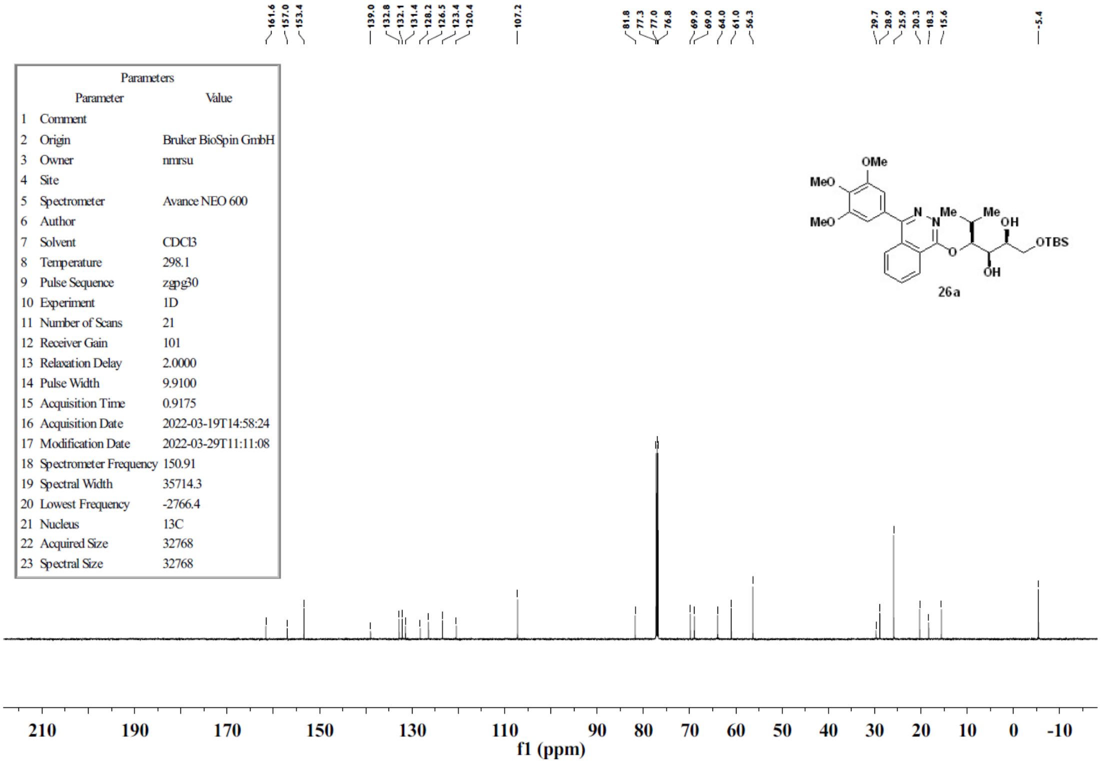

Supplementary Figure 327. ^{13}C NMR (151 MHz, $CDCl_3$) spectrum of compound **26a**.

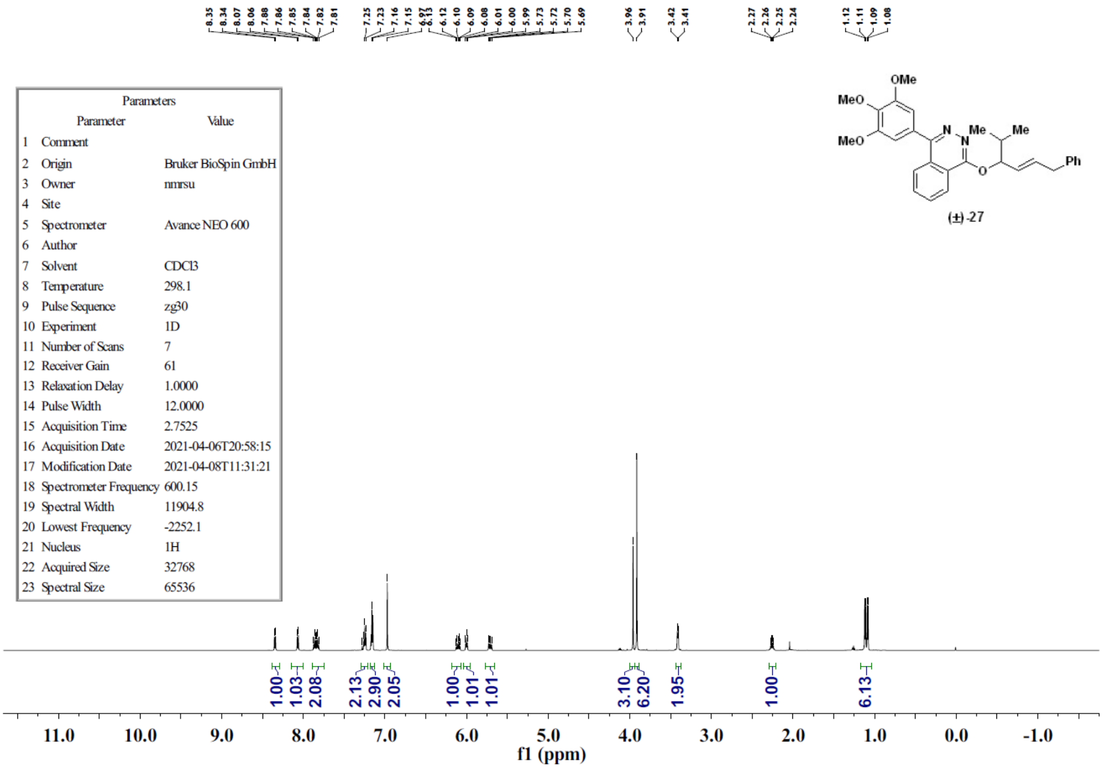

Supplementary Figure 328. ^{1}H NMR (600 MHz, $CDCl_3$) spectrum of compound **(±)-27**.

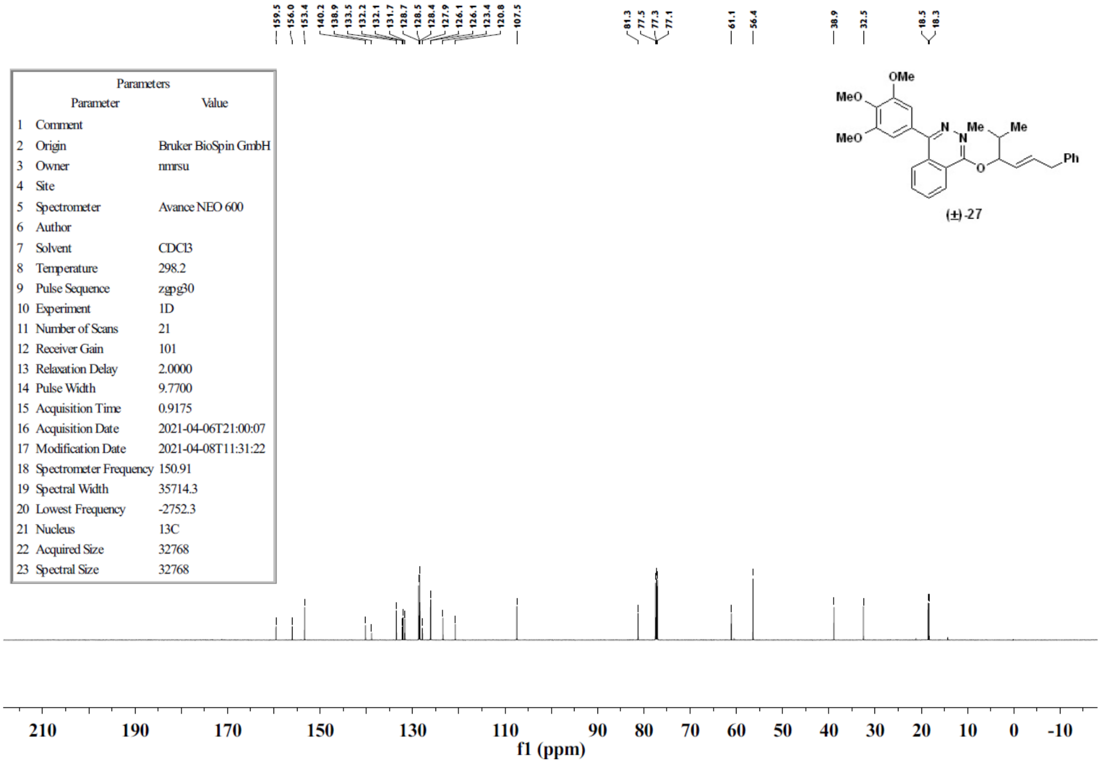

Supplementary Figure 329. ^{13}C NMR (151 MHz, $CDCl_3$) spectrum of compound **(±)-27**.

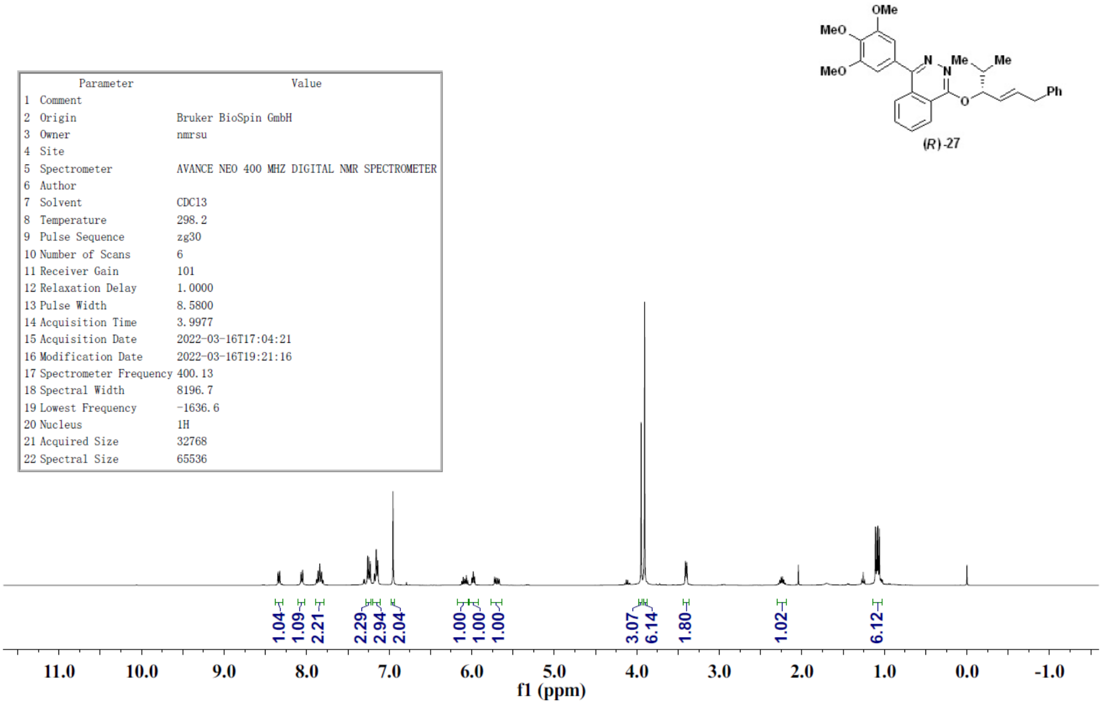

Supplementary Figure 330. ^{1}H NMR (400 MHz, $CDCl_3$) spectrum of compound ***(R)*-27**.

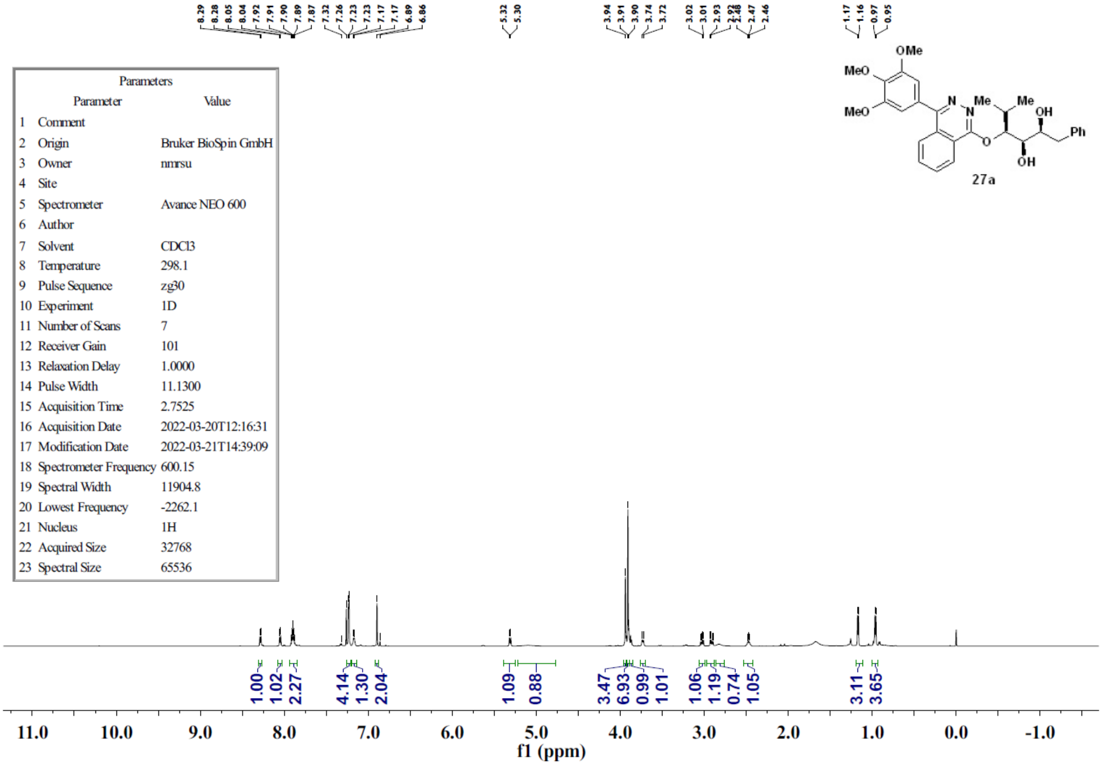

Supplementary Figure 331. ^{1}H NMR (600 MHz, $CDCl_3$) spectrum of compound **27a**.

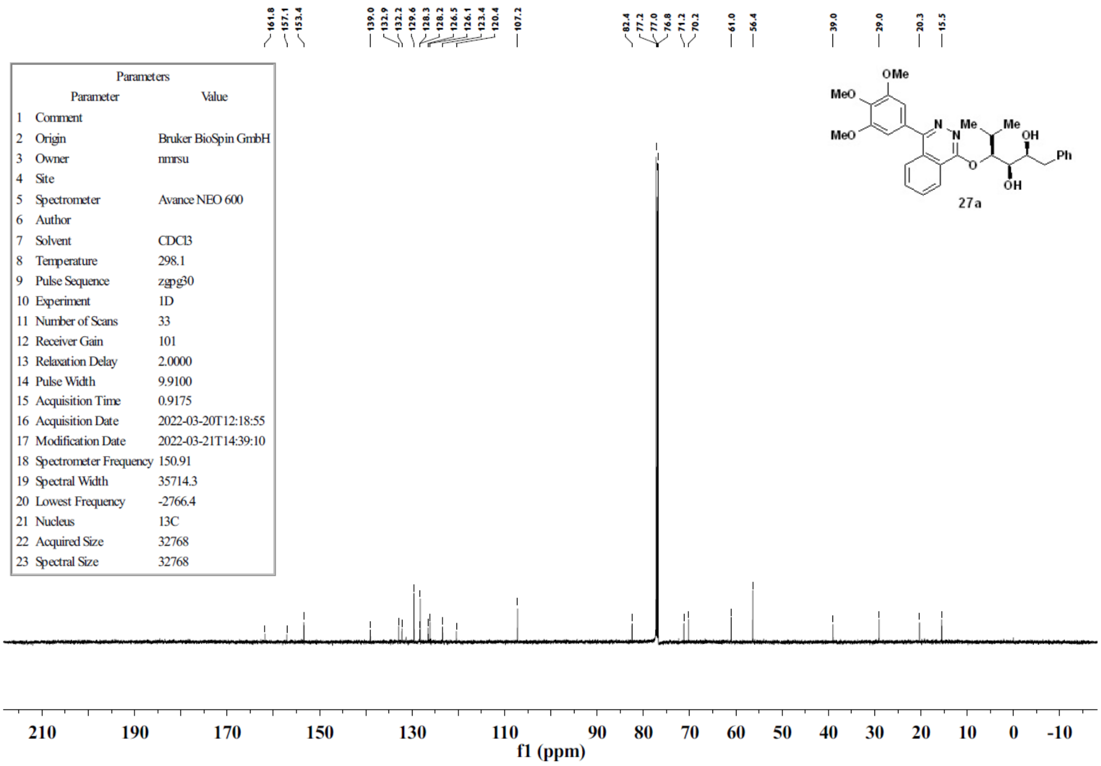

Supplementary Figure 332. ^{13}C NMR (151 MHz, $CDCl_3$) spectrum of compound **27a**.

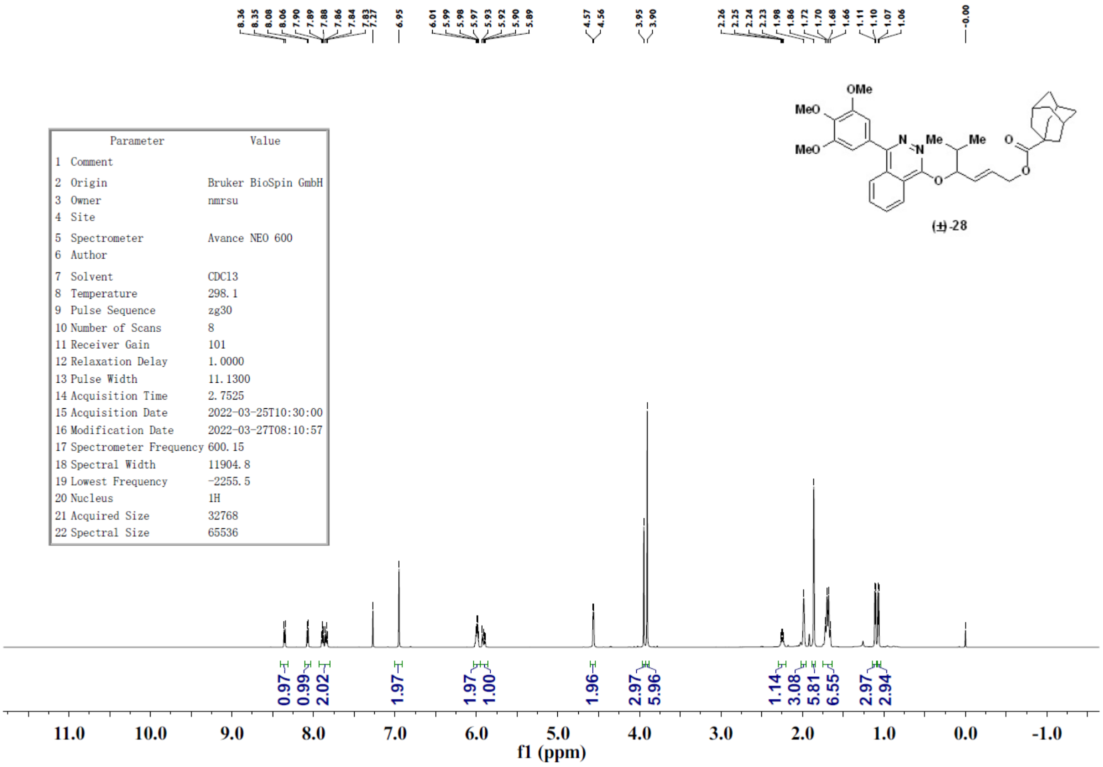

Supplementary Figure 333. ^{1}H NMR (600 MHz, $CDCl_3$) spectrum of compound **(±)-28**.

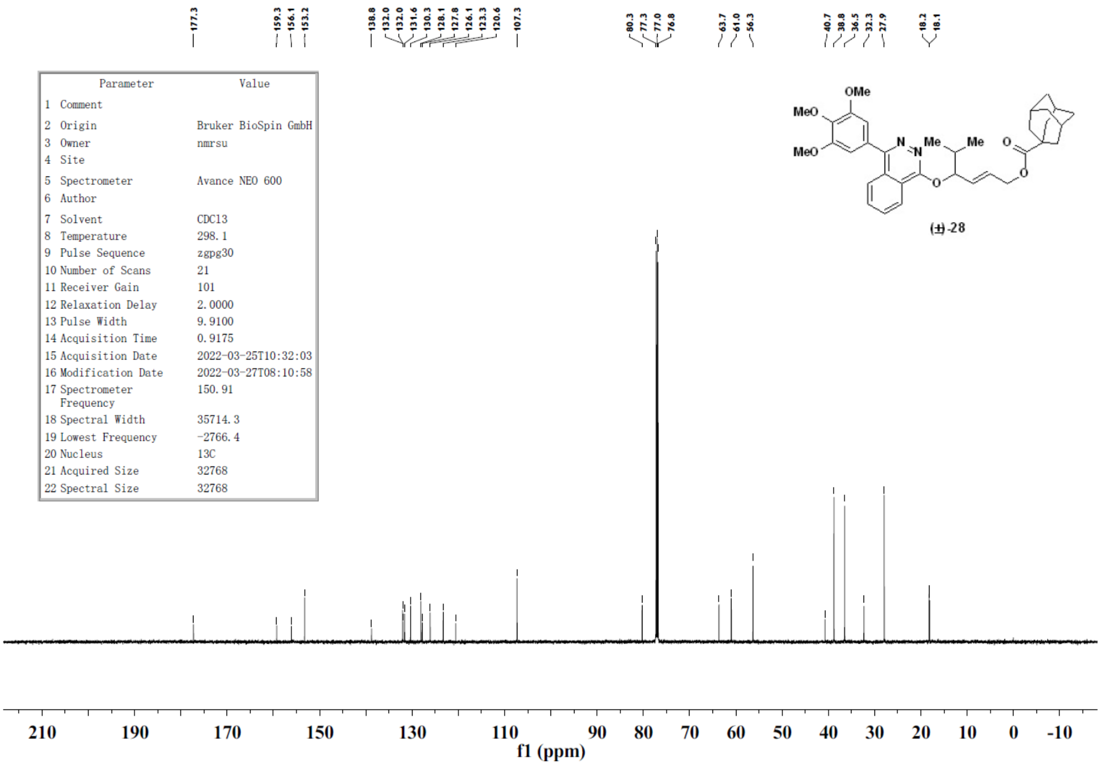

Supplementary Figure 334. ^{13}C NMR (151 MHz, $CDCl_3$) spectrum of compound **(±)-28**.

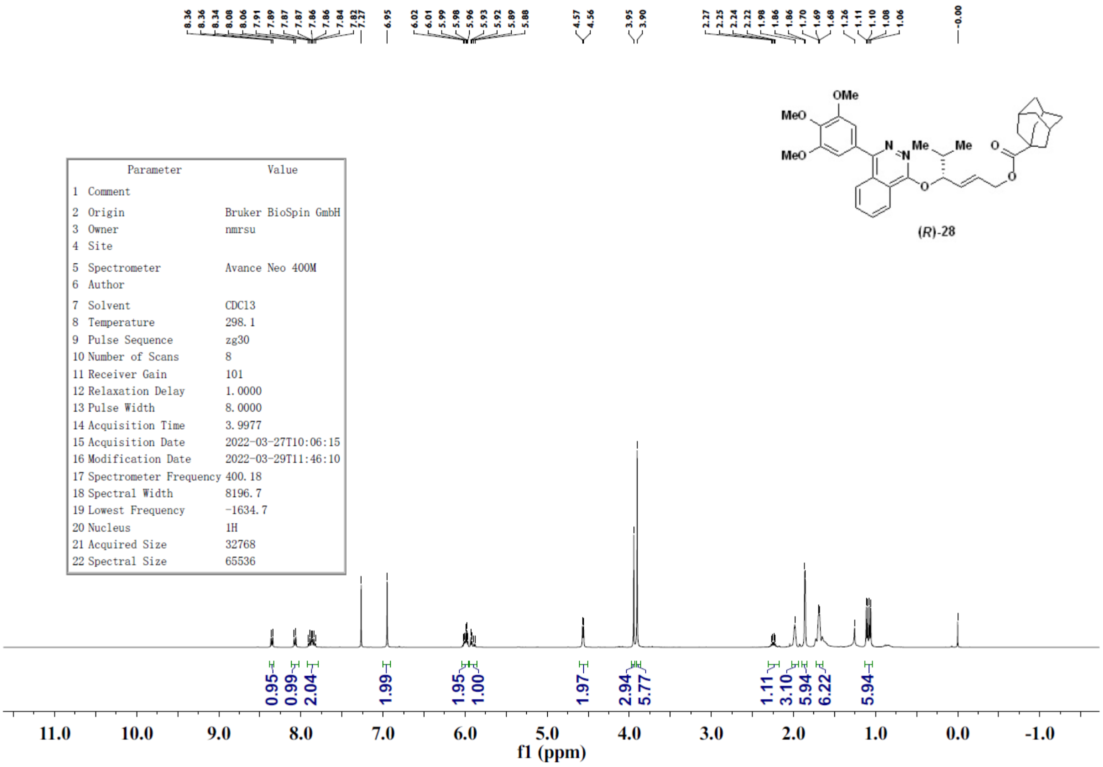

Supplementary Figure 335. ^{1}H NMR (400 MHz, $CDCl_3$) spectrum of compound **(*R*)-28**.

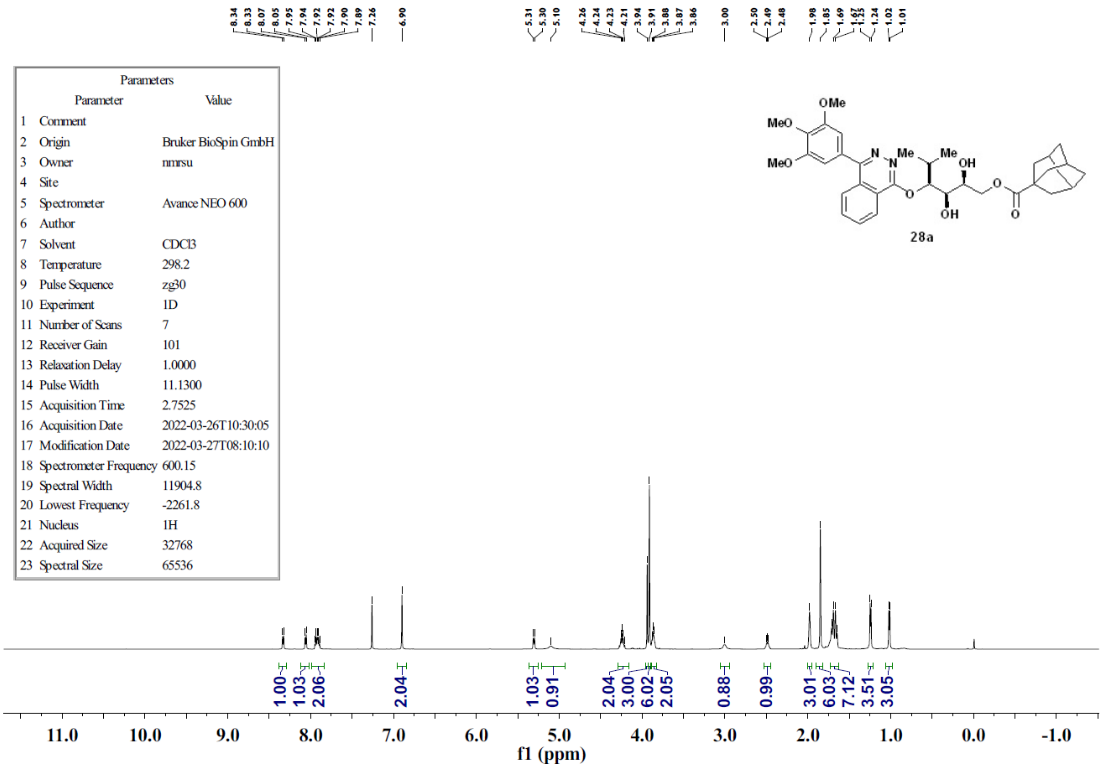

Supplementary Figure 336. ^{1}H NMR (600 MHz, $CDCl_3$) spectrum of compound **28a**.

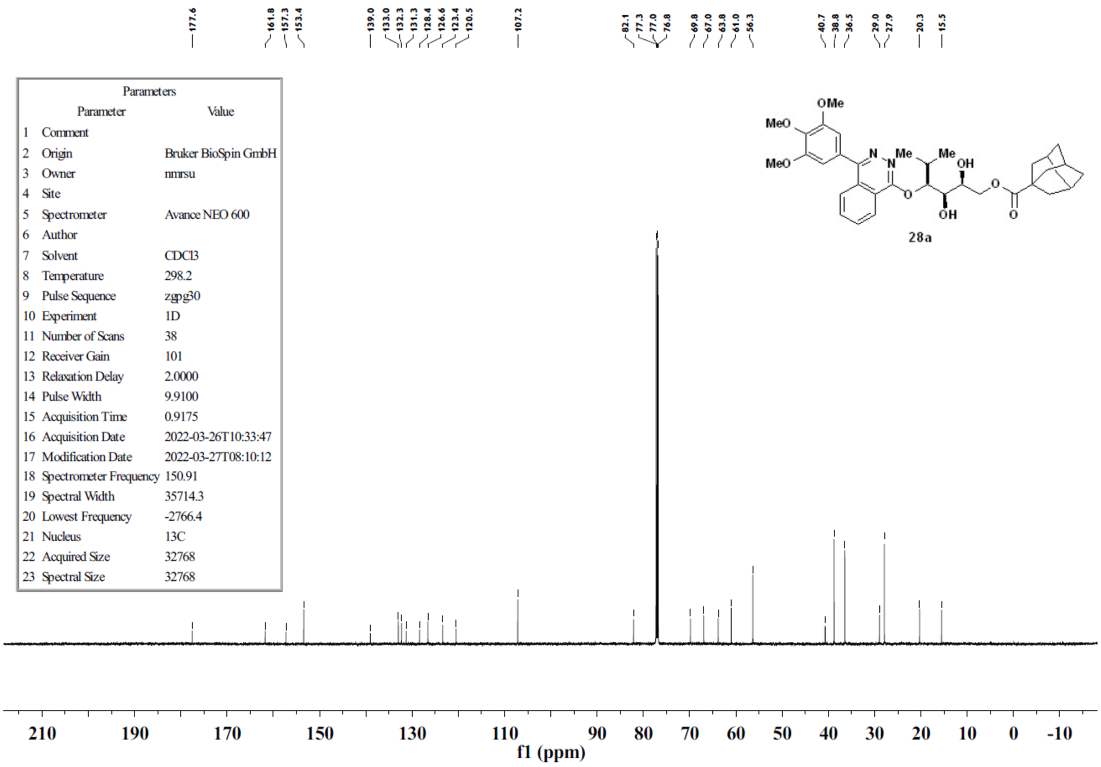

Supplementary Figure 337. ^{13}C NMR (151 MHz, $CDCl_3$) spectrum of compound **28a**.

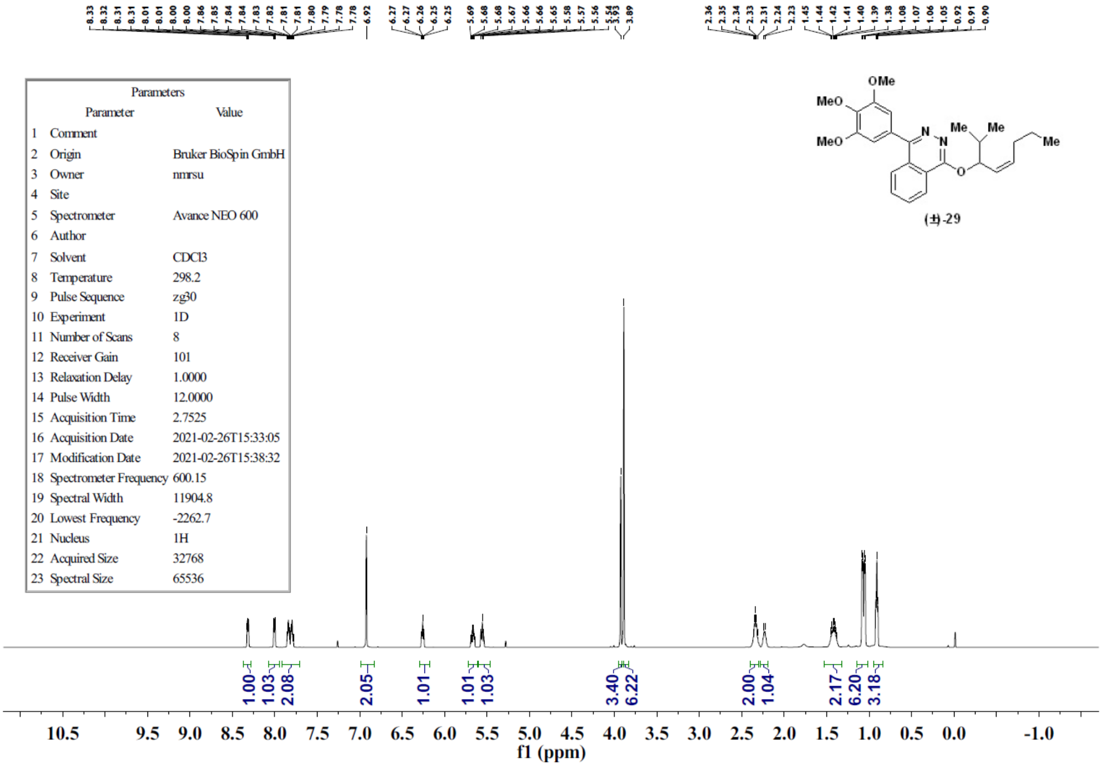

Supplementary Figure 338. ^{1}H NMR (600 MHz, $CDCl_3$) spectrum of compound **(±)-29**.

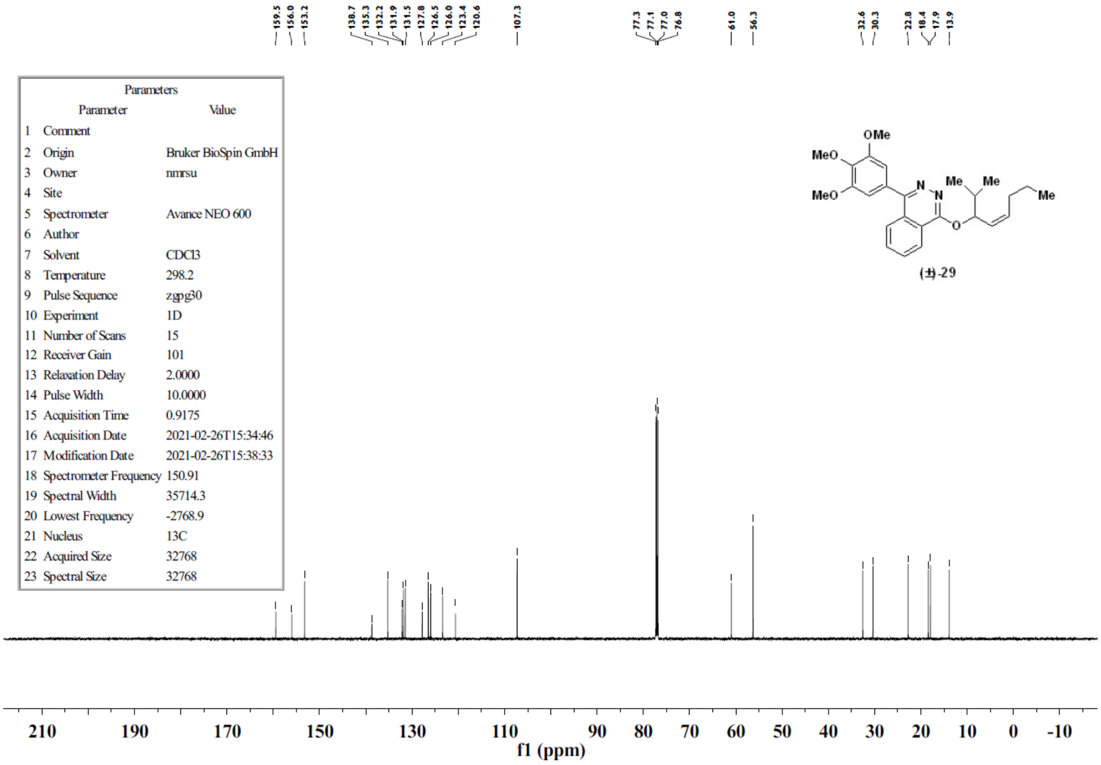

Supplementary Figure 339. ^{13}C NMR (151 MHz, $CDCl_3$) spectrum of compound **(±)-29**.

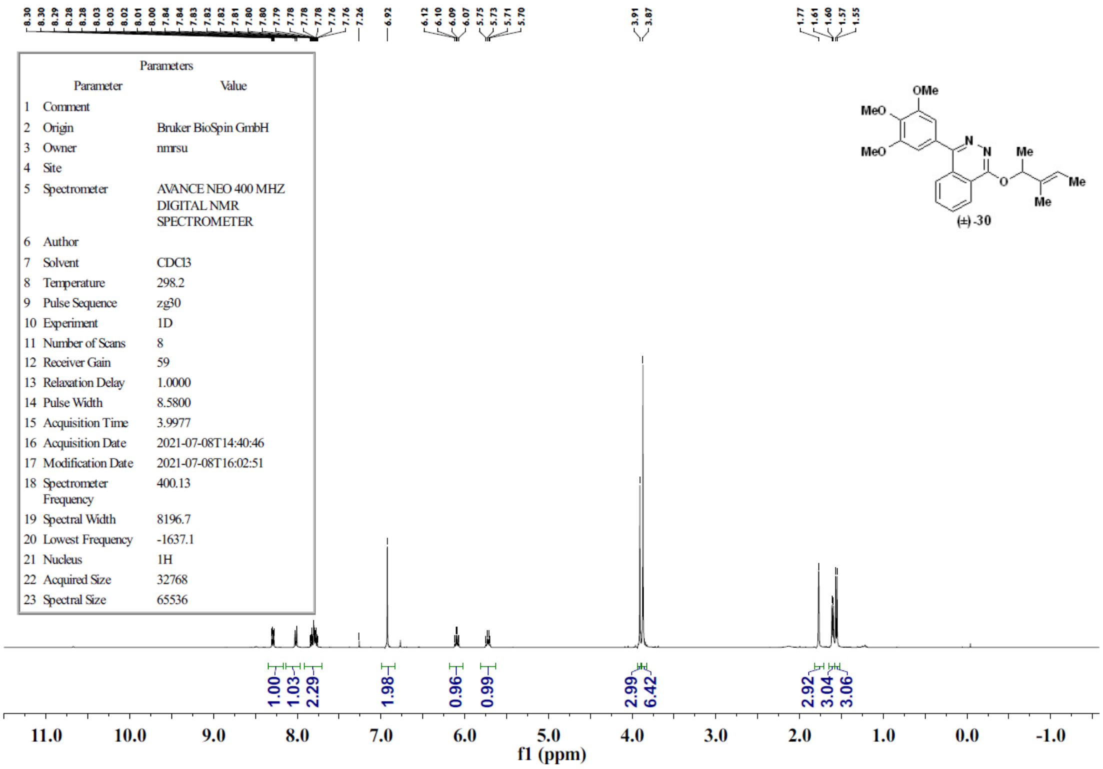

Supplementary Figure 340. ^{1}H NMR (400 MHz, $CDCl_3$) spectrum of compound **(±)-30**.

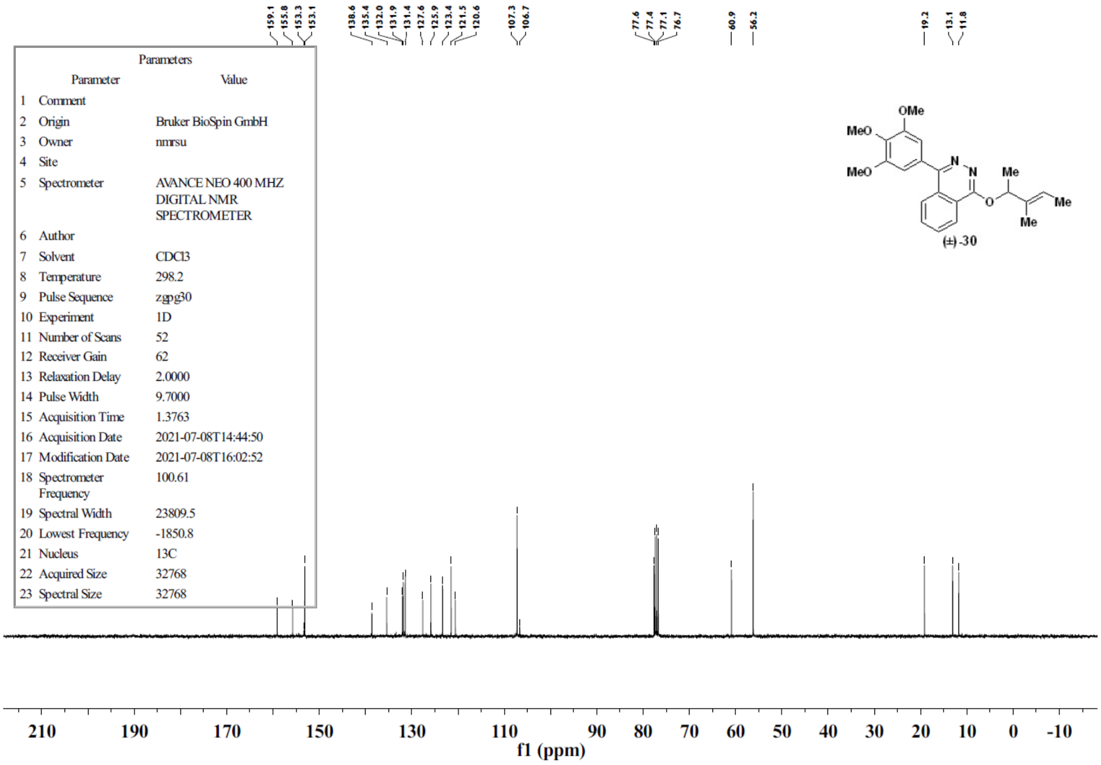

Supplementary Figure 341. ^{13}C NMR (101 MHz, $CDCl_3$) spectrum of compound **(±)-30**.

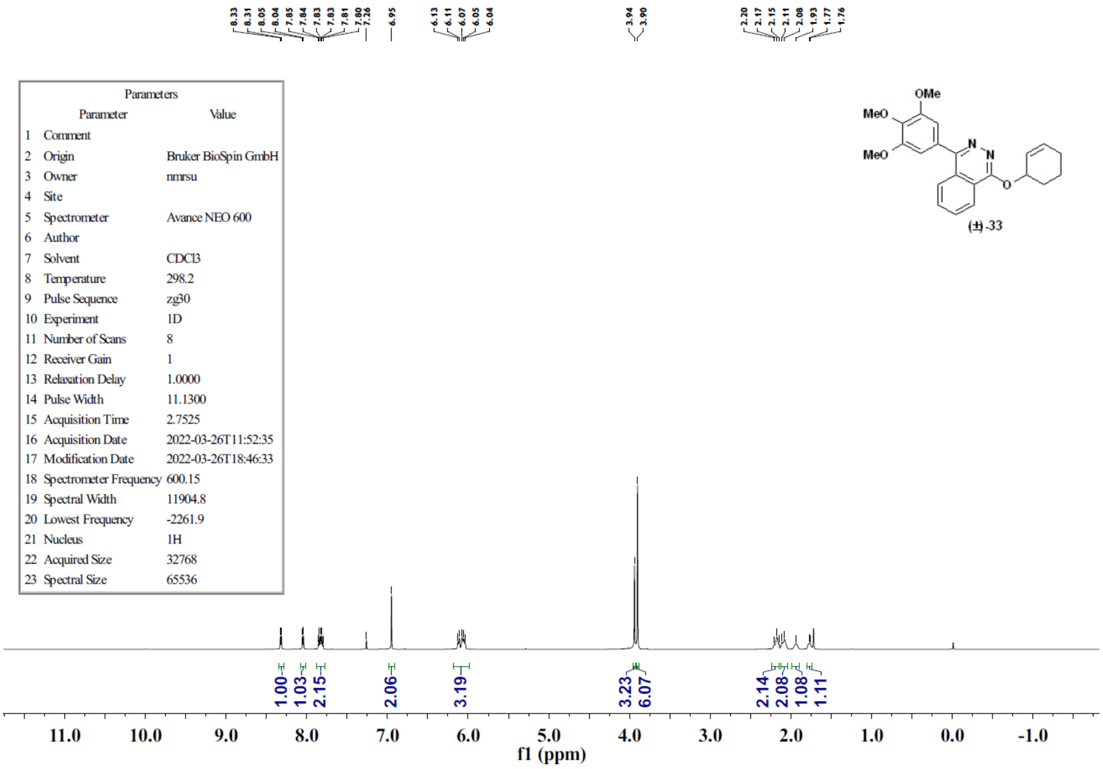

Supplementary Figure 342. ^{1}H NMR (600 MHz, $CDCl_3$) spectrum of compound **(±)-33**.

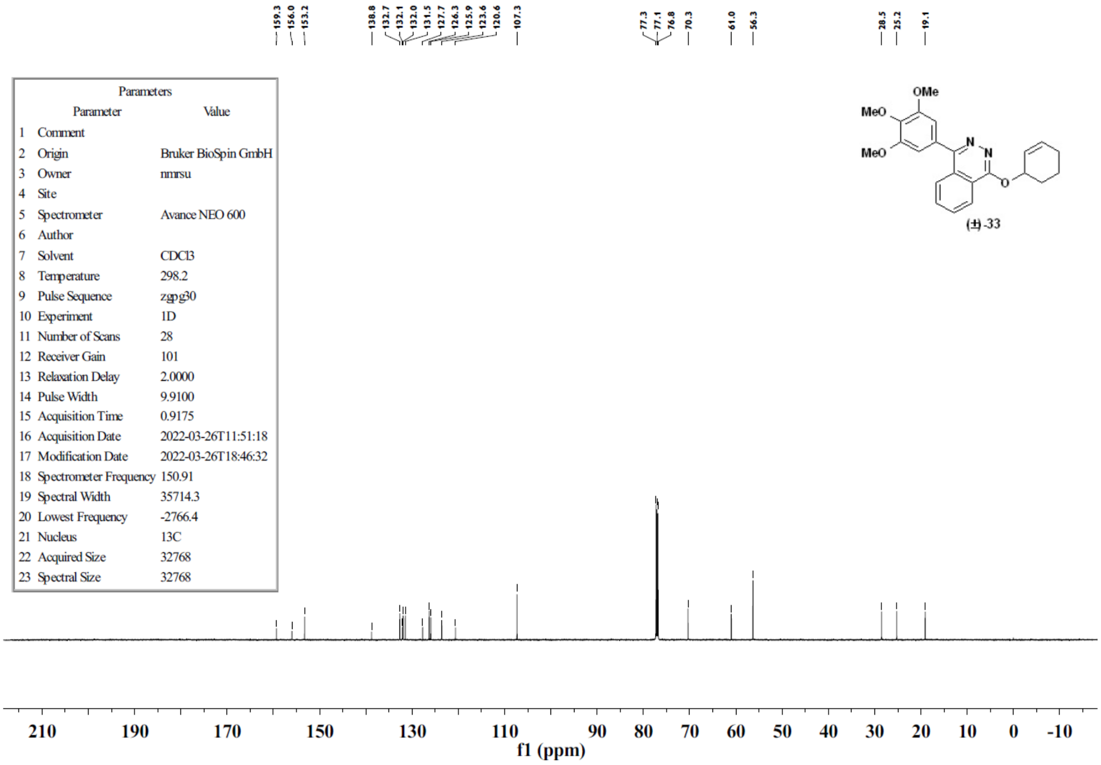

Supplementary Figure 343. ^{13}C NMR (151 MHz, $CDCl_3$) spectrum of compound **(±)-33**.

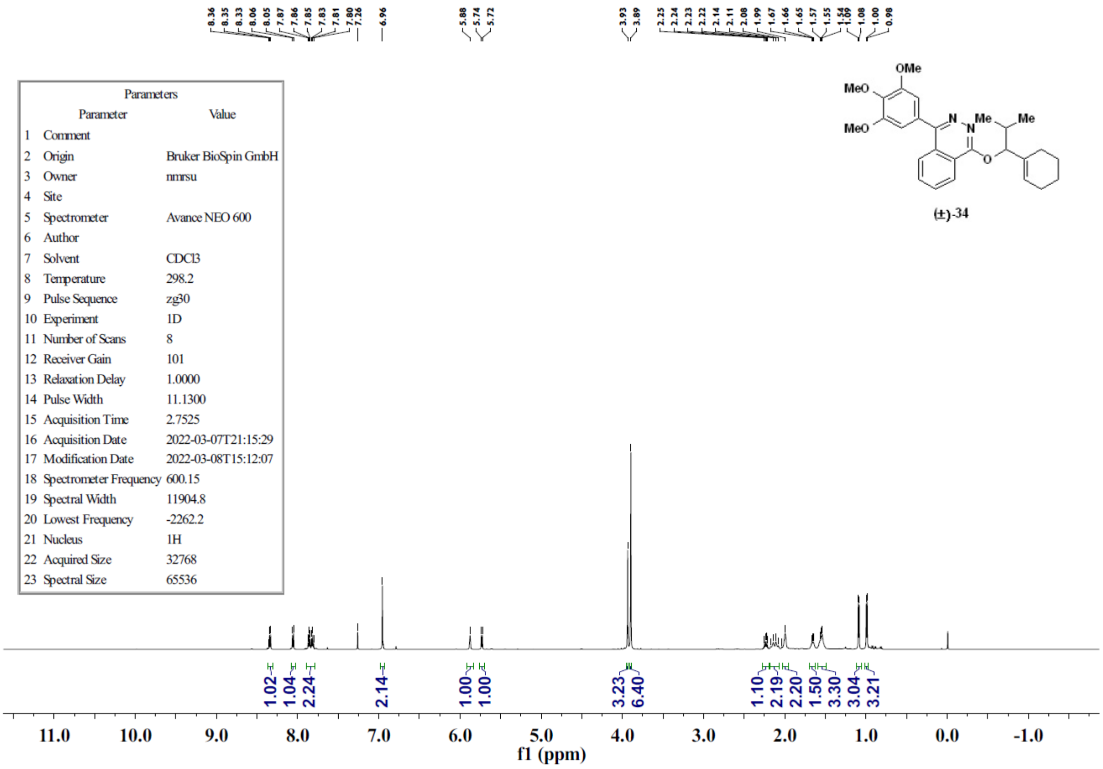

Supplementary Figure 344. ^{1}H NMR (600 MHz, $CDCl_3$) spectrum of compound **(±)-34**.

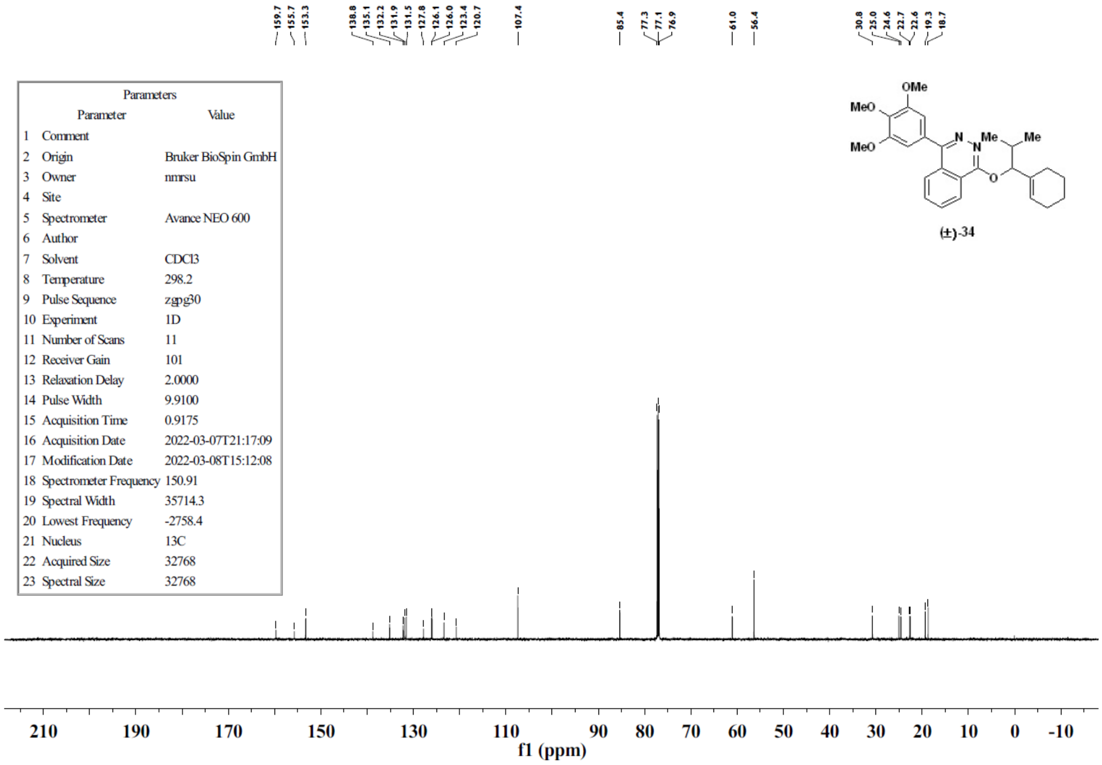

Supplementary Figure 345. ^{13}C NMR (151 MHz, $CDCl_3$) spectrum of compound **(±)-34**.

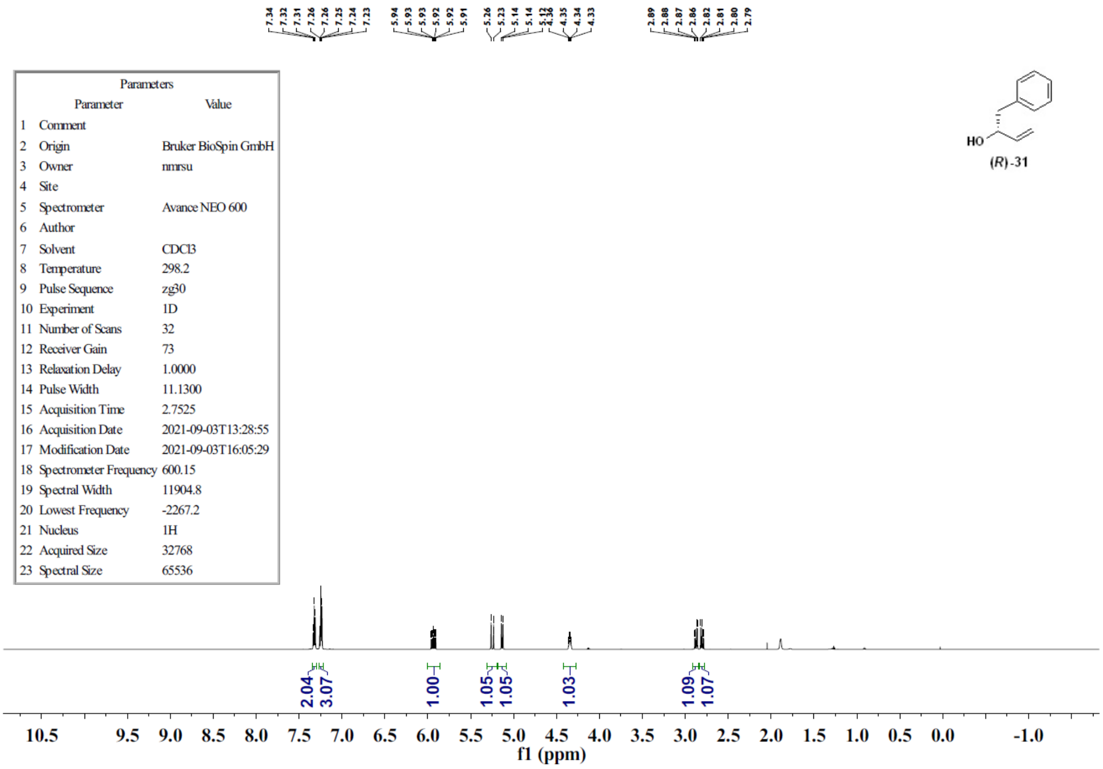

Supplementary Figure 346. ^{1}H NMR (600 MHz, $CDCl_3$) spectrum of compound ***(R)*-31**.

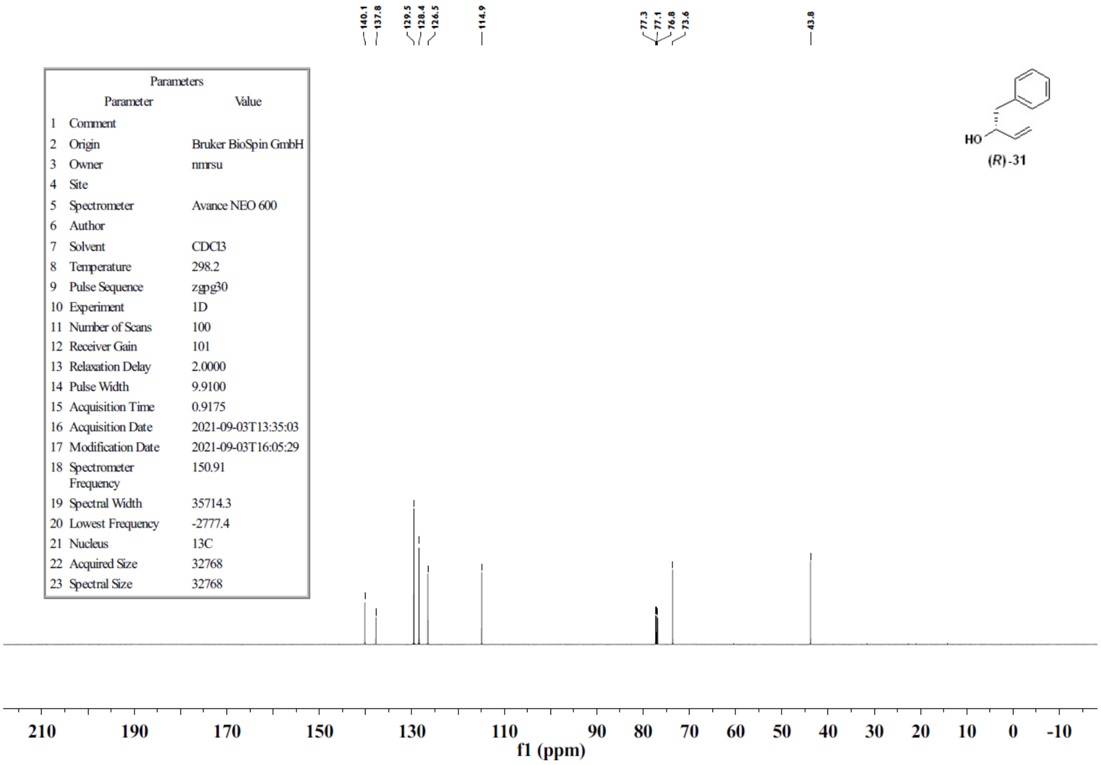

Supplementary Figure 347. ^{13}C NMR (151 MHz, $CDCl_3$) spectrum of compound ***(R)*-31**.

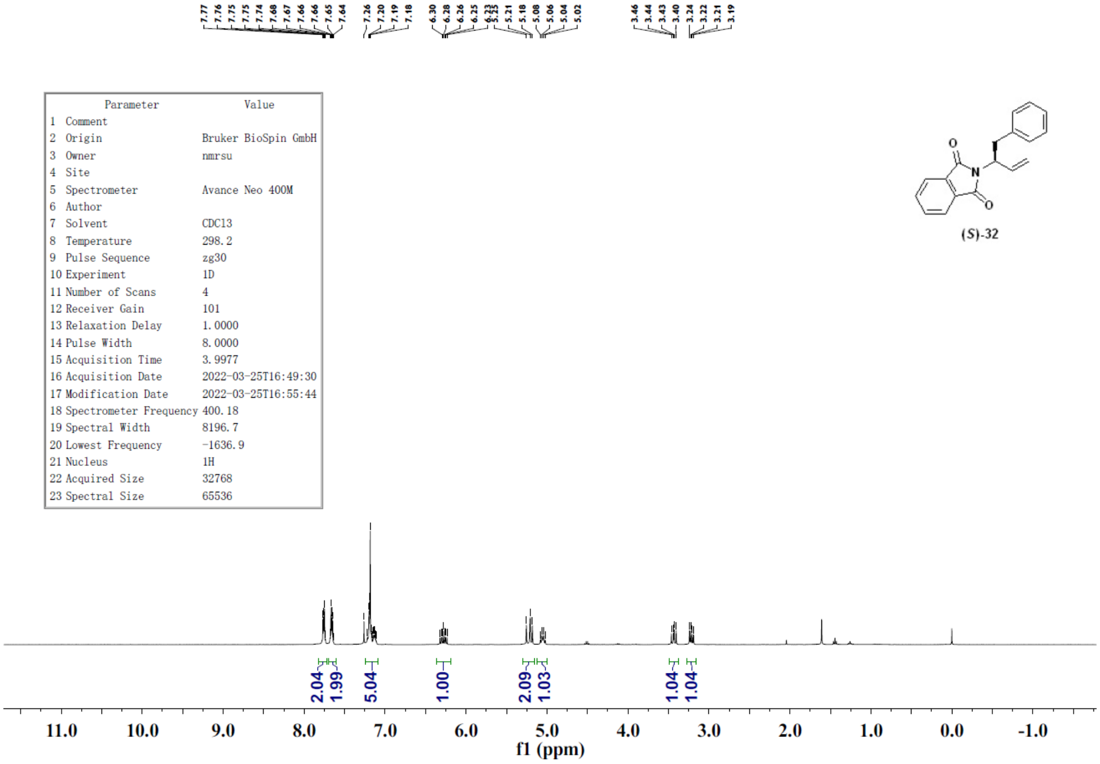

Supplementary Figure 348. ^{1}H NMR (400 MHz, $CDCl_3$) spectrum of compound ***(S)*-32**.

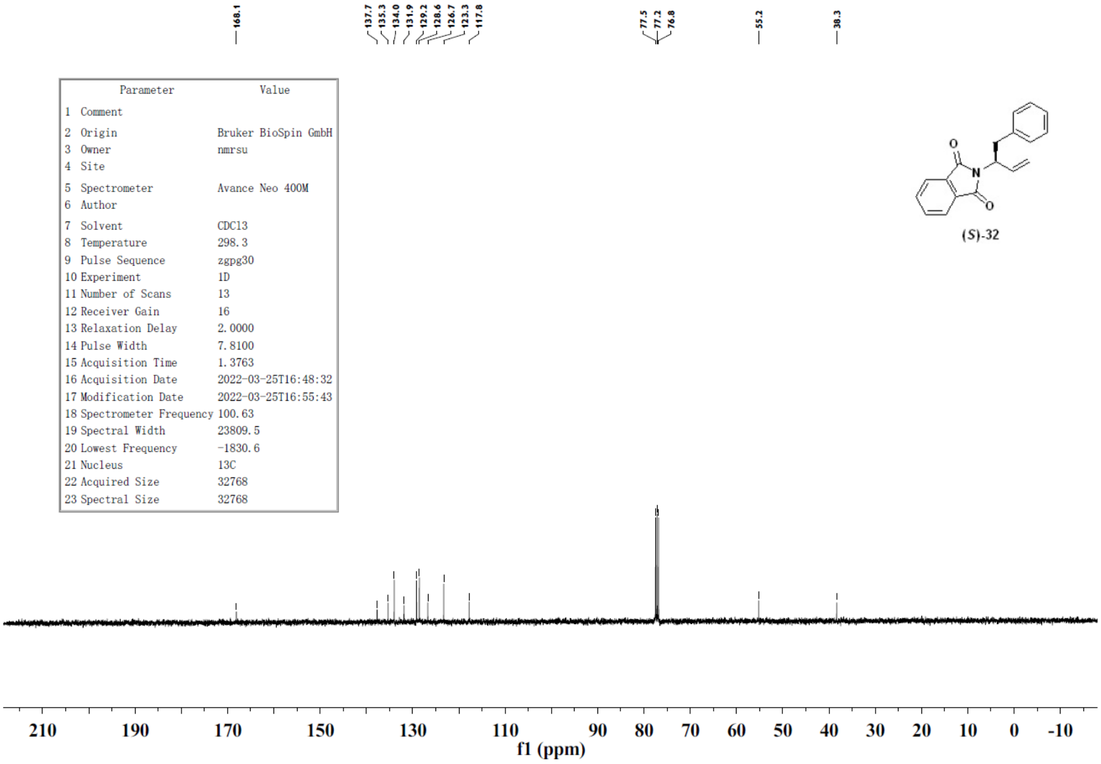

Supplementary Figure 349. ^{13}C NMR (101 MHz, $CDCl_3$) spectrum of compound ***(S)*-32**.

5 Supplementary References

1. GaussView, Version 6.1, R. Dennington, T. A. Keith, and J. M. Millam, Semichem Inc., Shawnee Mission, KS, (2016).
2. C. Bannwarth, E. Caldeweyher, S. Ehlert, A. Hansen, P. Pracht, J. Seibert, S. Spicher, S. Grimme, *WIREs Comput. Mol. Sci.*, e01493 (2020).
3. S. Spicher, S. Grimme, *Angew. Chem. Int. Ed.* **59**, 15665-15673 (2020).
4. C. Bannwarth, S. Ehlert, S. Grimme, *J. Chem. Theory Comput.* **15**, 1652-1671 (2019).
5. Gaussian 16, Revision A.03, M. J. Frisch, G. W. Trucks, H. B. Schlegel, G. E. Scuseria, M. A. Robb, J. R. Cheeseman, G. Scalmani, V. Barone, G. A. Petersson, H. Nakatsuji, X. Li, M. Caricato, A. V. Marenich, J. Bloino, B. G. Janesko, R. Gomperts, B. Mennucci, H. P. Hratchian, J. V. Ortiz, A. F. Izmaylov, J. L. Sonnenberg, D. Williams-Young, F. Ding, F. Lipparini, F. Egidi, J. Goings, B. Peng, A. Petrone, T. Henderson, D. Ranasinghe, V. G. Zakrzewski, J. Gao, N. Rega, G. Zheng, W. Liang, M. Hada, M. Ehara, K. Toyota, R. Fukuda, J. Hasegawa, M. Ishida, T. Nakajima, Y. Honda, O. Kitao, H. Nakai, T. Vreven, K. Throssell, J. A. Montgomery, Jr., J. E. Peralta, F. Ogliaro, M. J. Bearpark, J. J. Heyd, E. N. Brothers, K. N. Kudin, V. N. Staroverov, T. A. Keith, R. Kobayashi, J. Normand, K. Raghavachari, A. P. Rendell, J. C. Burant, S. S. Iyengar, J. Tomasi, M. Cossi, J. M. Millam, M. Klene, C. Adamo, R. Cammi, J. W. Ochterski, R. L. Martin, K. Morokuma, O. Farkas, J. B. Foresman, and D. J. Fox, Gaussian, Inc., Wallingford CT, (2016).
6. J.-D. Chai, M. Head-Gorden, *J. Chem. Phys.* **128**, 084106 (2008).
7. F. Weigend, R. Ahlrichs, *Phys. Chem. Chem. Phys.* **7**, 3297-3305 (2005).
8. C. Adamo, V. Barone, *J. Chem. Phys.* **110**, 6158-6170 (1999).
9. S. Grimme, S. Ehrlich, L. Goerigk, *J. Comput. Chem.* **32**, 1456-1465 (2011).
10. NBO 7.0. E. D. Glendening, J, K. Badenhoop, A. E. Reed, J. E. Carpenter, J. A. Bohmann, C. M. Morales, P. Karafiloglou, C. R. Landis, and F. Weinhold, Theoretical Chemistry Institute, University of Wisconsin, Madison (2018).
11. E. R. Johnson; S. Keinan; P. Mori-Sánchez; J. Contreras-García; A. J. Cohen; W.-T. Yang. *J. Am. Chem. Soc.* **132**, 6498-6506 (2010).
12. W. Humphrey; A. Dalke; K. Schulten. *J. Mol. Graphics.* **14**, 33-38 (1996).

13. A. V. Marenich, C. J. Cramer, D. G. Truhlar, *J. Phys. Chem. B.* **113**, 6378-6396 (2009).

14. L. Zhao, M. V. Hopffgarten, D. M. Andrada, G. Frenking, WIREs Comput. *Mol. Sci*. **8**, e1345 (2018).

15. K. Morokuma, *J. Chem. Phys.* **55**, 1236-1244 (1971).

16. T. Ziegler, A. Rauk, *Theeoretica. Chimica. Acta.* **46**, 1-10 (1977).

17. J. G. Snijders, P.Vernooijs, E. J. Baerends, *At. Data Nucl. Data Tables.* **26**, 483-509 (1981).

18. E. J. Baerends, T. Ziegler, J. Autschbach, D. Bashford, A. Be'rces, F. M. Bickelhaupt, C. Bo, P. M. Boerrigter, L. Cavallo, D. P. Chong, L. Deng, R. M. Dickson, D. E. Ellis, M. v. Faassen, L. Fan, T. H. Fischer, C. F. Guerra, A. Ghysels, A. Giammona, S. J. A. v. Gisbergen, A. W. Gotz, J.A. Groeneveld, O. V. Gritsenko, M. Gr ning, S. Gusarov, F. E. Harris, P. v. d. Hoek, C. R. Jacob, H. Jacobsen, L. Jensen, J. W. Kaminski, G. v. Kessel, F. Kootstra, A. Kovalenko, M. V. Krykunov, E. v. Lenthe, D. A. McCormack, A. Michalak, M. Mitoraj, J. Neugebauer, V. P. Nicu, L. Noodleman, V. P. Osinga, S. Patchkovskii, P. H. T. Philipsen, D. Post, C. C. Pye, W. Ravenek, J. I. Rodrlguez, P. Ros, P. R. T. Schipper, G. Schreckenbach, J. S. Seldenthuis, M. Seth, J. G. Snijders, M. Sola, M. Swart, D. Swerhone, G. t. Velde, P. Vernooijs, L. Versluis, L. Visscher, O. Visser, F. Wang, T. A. Wesolowski, E. M. v. Wezenbeek, G. Wiesenekker, S. K.Wolff, T. K. Woo, A. L. Yakovlev, ADF, SCM, Theoretical Chemistry, Vrije Universiteit, Amsterdam, The Netherlands.

19. E. R. Johnson; S. Keinan; P. Mori-Sánchez; J. Contreras-García; A.J. Cohen; W.-T. Yang. *J. Am. Chem. Soc*. **132**, 6498-6506 (2010),.

20. W. Humphrey; A. Dalke; K. Schulten. *J. Mol. Graphics.* **14**, 33-38 (1996).
