# Supplementary material for: Engineered non-covalent π interactions as key elements for chiral recognition
Source: Nat Commun. 2022 Jun 7;13:3276. doi: 10.1038/s41467-022-31026-8 (PMC9174283; doi:10.1038/s41467-022-31026-8)
Supplement: Supplementary file 2 — Description of Additional Supplementary Files [file 41467_2022_31026_MOESM2_ESM.docx]

**Description of Additional Supplementary Files**

File Name: Supplementary Data 1

Description: Cartesian coordinates of computed structures.
