# Supplementary material for: Engineered non-covalent π interactions as key elements for chiral recognition
Source: Nat Commun. 2022 Jun 7;13:3276. doi: 10.1038/s41467-022-31026-8 (PMC9174283; doi:10.1038/s41467-022-31026-8)
Supplement: Supplementary file 3 — Supplementary Data 1 [file 41467_2022_31026_MOESM3_ESM.pdf]

Cartesian coordinates of computed structures.

12-*R*

Cartesian coordinates

ATOM	X	Y	Z
C	2.51311	3.17806	-1.60976
C	3.15954	2.05615	-1.13984
C	2.40096	0.96722	-0.65838
C	0.99147	1.02103	-0.63267
C	0.35256	2.17397	-1.15179
C	1.10319	3.22806	-1.62698
H	3.09004	4.02562	-1.98537
H	4.24745	1.98296	-1.13864
C	2.99185	-0.26254	-0.21074
C	0.31001	-0.14538	-0.11502
H	-0.73613	2.21515	-1.18844
H	0.60053	4.11010	-2.02961
C	-1.17112	-0.20399	0.03284
C	-1.86999	0.83196	0.66113
C	-1.84689	-1.33745	-0.42160
C	-3.25748	0.74052	0.81878
H	-1.32424	1.69113	1.04900
C	-3.23275	-1.43155	-0.26497
H	-1.26817	-2.13785	-0.87923
C	-3.94388	-0.39876	0.36916
O	-3.97698	-2.47271	-0.68716
O	-4.02132	1.68905	1.39811
C	-3.40336	2.83586	1.91021
H	-2.88172	3.40991	1.12354
H	-4.20012	3.45969	2.33331
H	-2.68107	2.59151	2.70852
C	-3.33180	-3.57095	-1.27303
H	-2.80637	-3.29064	-2.20266
H	-2.60778	-4.03506	-0.58180
H	-4.11388	-4.30136	-1.51326
N	0.95733	-1.22734	0.23296
N	2.29924	-1.28965	0.17866
O	4.32661	-0.31462	-0.23930
C	4.99188	-1.46612	0.28113
H	4.36099	-2.34712	0.08675
C	5.19392	-1.34973	1.76897
C	4.95315	-0.26443	2.49955
H	5.58722	-2.25488	2.24529
H	5.13355	-0.25665	3.57654
H	4.55809	0.64961	2.04808

O	-5.28055	-0.50567	0.56500
C	-6.07375	-0.07050	-0.51403
H	-7.12286	-0.20763	-0.22135
H	-5.90079	0.99769	-0.73235
H	-5.87555	-0.66426	-1.42265
C	6.30934	-1.58663	-0.47000
H	6.87554	-2.45565	-0.10549
H	6.12720	-1.71533	-1.54620
H	6.92034	-0.68517	-0.31623

PBE0+D3(BJ)/Def2-SVP Gibbs free energy:-1222.171209 a.u.

PBE0+D3(BJ)/Def2-SVP enthalpy:-1222.087307 a.u.

PBE0+D3(BJ)/Def2-SVP SCF energy:-1222.513108 a.u.

PBE0+D3(BJ)/Def2-TZVPP Gibbs free energy:-1222.554548 a.u.

PBE0+D3(BJ)/Def2-TZVPP enthalpy:-1222.470646 a.u.

PBE0+D3(BJ)/Def2-TZVPP SCF energy:-1222.896447 a.u.

12-*S*

Cartesian coordinates

ATOM	X	Y	Z
C	-2.51337	3.17830	-1.60930
C	-3.15972	2.05631	-1.13948
C	-2.40105	0.96732	-0.65828
C	-0.99155	1.02117	-0.63270
C	-0.35274	2.17421	-1.15172
C	-1.10346	3.22835	-1.62667
H	-3.09037	4.02591	-1.98472
H	-4.24762	1.98307	-1.13819
C	-2.99184	-0.26256	-0.21088
C	-0.31000	-0.14531	-0.11536
H	0.73594	2.21542	-1.18852
H	-0.60086	4.11046	-2.02925
C	1.17112	-0.20385	0.03261
C	1.86992	0.83206	0.66101
C	1.84698	-1.33731	-0.42178
C	3.25741	0.74065	0.81880
H	1.32415	1.69118	1.04895
C	3.23279	-1.43139	-0.26499
H	1.26828	-2.13770	-0.87945
C	3.94388	-0.39859	0.36922
O	3.97710	-2.47259	-0.68702
O	4.02115	1.68917	1.39828
C	3.40317	2.83618	1.90992
H	2.68078	2.59214	2.70824
H	4.19990	3.46013	2.33289

H	2.88163	3.40997	1.12299
C	3.33201	-3.57088	-1.27293
H	2.60787	-4.03492	-0.58181
H	2.80675	-3.29062	-2.20268
H	4.11414	-4.30129	-1.51297
N	-0.95726	-1.22739	0.23237
N	-2.29915	-1.28977	0.17815
O	-4.32662	-0.31463	-0.23916
C	-4.99178	-1.46628	0.28103
H	-4.36089	-2.34719	0.08627
C	-5.19366	-1.35033	1.76895
C	-4.95279	-0.26521	2.49978
H	-5.58685	-2.25563	2.24504
H	-5.13306	-0.25774	3.57679
H	-4.55779	0.64892	2.04845
O	5.28051	-0.50546	0.56525
C	6.07390	-0.07129	-0.51404
H	7.12295	-0.20812	-0.22101
H	5.87591	-0.66591	-1.42214
H	5.90094	0.99668	-0.73339
C	-6.30936	-1.58662	-0.46992
H	-6.87561	-2.45558	-0.10533
H	-6.92022	-0.68510	-0.31601
H	-6.12747	-1.71532	-1.54617

PBE0+D3(BJ)/Def2-SVP Gibbs free energy:-1222.171233 a.u.

PBE0+D3(BJ)/Def2-SVP enthalpy:-1222.087309 a.u.

PBE0+D3(BJ)/Def2-SVP SCF energy:-1222.513108 a.u.

PBE0+D3(BJ)/Def2-TZVPP Gibbs free energy:-1222.554572 a.u.

PBE0+D3(BJ)/Def2-TZVPP enthalpy:-1222.470648 a.u.

PBE0+D3(BJ)/Def2-TZVPP SCF energy:-1222.896447 a.u.

Cat

Cartesian coordinates

ATOM	X	Y	Z
C	-0.13769	-0.16723	3.35510
O	0.16086	-1.32474	3.27609
O	0.15385	0.73798	2.39288
C	0.72420	0.23397	1.19473
H	0.32265	-0.77283	1.02294
C	0.35361	1.15753	0.02649
C	0.94743	2.57661	0.16103
H	0.80439	0.66999	-0.84847
C	-1.79765	2.21938	0.55979
C	-1.18133	1.72220	-1.70385

C	0.00005	3.56211	-0.52703
H	1.05377	2.85082	1.22213
H	1.95669	2.60229	-0.27382
H	-2.87131	2.14132	0.33518
H	-1.65051	1.90418	1.59714
C	-1.27567	3.65249	0.31735
C	-0.38134	3.03479	-1.92239
H	-2.24505	1.85794	-1.93678
H	-0.80521	0.91868	-2.35687
H	0.46786	4.55384	-0.60569
H	-2.02779	4.26600	-0.20282
H	-1.06265	4.14907	1.27582
H	-1.05427	3.77926	-2.38141
N	-1.09594	1.24552	-0.30530
C	0.80082	2.84034	-2.87961
H	0.39923	2.47468	-3.83994
H	1.45799	2.03362	-2.51140
C	1.62612	4.09795	-3.12426
H	2.15332	4.42472	-2.21503
H	2.38718	3.92624	-3.89915
H	0.98964	4.93283	-3.45868
C	2.23362	0.12894	1.30670
C	2.98336	-0.65513	0.37780
C	2.92194	0.82717	2.27111
C	2.38530	-1.44843	-0.64455
C	4.40203	-0.62727	0.50214
C	4.33350	0.76738	2.30881
H	2.38533	1.43082	3.00414
C	3.17620	-2.16872	-1.51599
H	1.30100	-1.49877	-0.72517
C	5.18456	-1.38241	-0.41939
H	4.87525	1.32682	3.08031
C	4.59516	-2.12631	-1.40047
H	6.26885	-1.34249	-0.30483
H	5.18268	-2.71078	-2.11048
N	5.05299	0.08123	1.45824
O	2.70990	-2.94655	-2.50827
Os	-2.43838	-1.00125	-0.21695
O	-1.00002	-1.41219	-1.03258
O	-3.47094	0.04298	-1.06779
O	-3.29701	-2.45463	-0.18933
O	-2.26887	-0.60532	1.42374
C	-0.90034	0.47472	4.47699
H	-0.87796	-0.17594	5.35788

H	-0.49611	1.46733	4.71397
H	-1.94358	0.59959	4.14935
C	1.32116	-3.05607	-2.68582
H	0.82809	-3.47670	-1.79336
H	0.86025	-2.07854	-2.90711
H	1.16557	-3.73050	-3.53607

PBE0+D3(BJ)/Def2-SVP Gibbs free energy:-1580.047603 a.u.

PBE0+D3(BJ)/Def2-SVP enthalpy:-1579.955110 a.u.

PBE0+D3(BJ)/Def2-SVP SCF energy:-1580.479100 a.u.

PBE0+D3(BJ)/Def2-TZVPP Gibbs free energy:-1580.531343 a.u.

PBE0+D3(BJ)/Def2-TZVPP enthalpy:-1580.438850 a.u.

PBE0+D3(BJ)/Def2-TZVPP SCF energy:-1580.962840 a.u.

IM1-*R*

Cartesian coordinates

ATOM	X	Y	Z
C	-0.67323	2.16187	0.62520
C	-0.34709	3.53145	2.69718
C	-1.35855	3.12681	1.62255
H	-0.95858	1.12973	0.86981
H	-1.69764	4.03602	1.10639
H	-2.24757	2.64516	2.05234
C	1.31850	3.59616	0.84193
H	2.41481	3.53229	0.90422
H	1.06842	4.05999	-0.11661
C	0.73313	4.38893	2.03096
H	1.51653	4.64107	2.76259
H	0.29936	5.33846	1.68270
H	-0.84577	4.11219	3.48657
C	0.31578	2.27815	3.29024
H	1.04679	2.61581	4.04545
C	1.09201	1.59154	2.13264
H	2.17646	1.64803	2.29177
H	0.84186	0.52355	2.06253
N	0.80643	2.21068	0.82038
C	-0.66227	1.32909	3.98991
H	-1.39722	0.93756	3.26864
H	-0.09057	0.45432	4.33836
C	-1.15131	2.34155	-0.82112
H	-0.56969	1.69971	-1.48807
O	-0.92504	3.69211	-1.22025
C	-0.79145	3.91105	-2.54529
O	-0.93281	3.05278	-3.36995
C	-2.61886	1.97717	-0.97290

C	-3.03255	0.69791	-1.45776
C	-3.59395	2.90000	-0.66439
C	-2.13328	-0.31900	-1.88641
C	-4.43759	0.47136	-1.56305
C	-4.95701	2.55488	-0.78278
H	-3.32273	3.90524	-0.34139
C	-2.61724	-1.48368	-2.44383
H	-1.06220	-0.17724	-1.77688
C	-4.89766	-0.76778	-2.09232
H	-5.72534	3.28886	-0.51245
C	-4.01897	-1.71158	-2.53289
H	-5.97578	-0.92166	-2.14121
H	-4.36034	-2.65736	-2.95537
N	-5.37222	1.38728	-1.20458
O	-1.85208	-2.46989	-2.94399
C	-0.45440	-2.28469	-2.99657
H	-0.04647	-3.15225	-3.52847
H	-0.19965	-1.36608	-3.55259
H	-0.00987	-2.25612	-1.99045
Os	2.22435	0.88817	-0.95306
O	0.91821	-0.13426	-0.58213
O	3.10194	0.00747	-2.09731
O	1.81325	2.31200	-1.78069
O	3.33434	1.12257	0.31253
C	6.31779	-0.24180	-0.89039
C	6.05535	-1.33600	-1.60163
H	7.05991	0.48342	-1.23148
H	5.78822	-0.02999	0.04145
H	6.58508	-1.51820	-2.54328
C	5.04238	-2.38813	-1.25217
H	4.19592	-2.31213	-1.95335
C	5.60079	-3.80328	-1.30221
H	4.80297	-4.52743	-1.08716
O	4.55188	-2.10797	0.06098
C	3.28573	-2.37367	0.36597
C	2.85866	-1.97764	1.67904
C	1.50037	-2.19814	1.98150
C	0.67085	-2.66930	0.89490
C	-0.81297	-2.59300	0.92880
C	-1.59501	-3.53829	0.25477
C	-1.42489	-1.48965	1.50737
C	-2.97535	-3.37273	0.20578
H	-1.09995	-4.36552	-0.24969
C	-2.81189	-1.30004	1.45256

H	-0.83911	-0.71214	1.98931
C	-3.60831	-2.26710	0.82709
N	2.50830	-2.98212	-0.48213
N	1.19312	-3.08840	-0.22729
O	-3.80926	-4.21244	-0.44032
O	-4.95664	-2.12174	0.72900
O	-3.22804	-0.13695	2.00046
C	-3.25683	-5.17942	-1.29927
H	-4.10220	-5.67378	-1.79335
H	-2.61334	-4.70495	-2.05801
H	-2.67823	-5.93741	-0.74382
C	-5.73855	-3.02935	1.47116
H	-5.57611	-4.06658	1.14128
H	-5.51874	-2.95275	2.55173
H	-6.78806	-2.75660	1.30054
C	-4.58579	0.23443	2.10858
H	-5.14517	-0.46388	2.75136
H	-4.57784	1.22487	2.58258
H	-5.07716	0.29979	1.12997
C	1.05984	-1.97068	3.30675
C	3.73883	-1.44376	2.64357
H	4.77750	-1.26260	2.36679
C	1.93548	-1.47831	4.25129
H	1.58842	-1.31065	5.27321
C	3.27380	-1.18666	3.91456
H	0.02678	-2.19123	3.57630
H	3.94662	-0.77887	4.67158
C	-1.40531	1.94811	5.16806
H	-2.00860	1.19259	5.69260
H	-0.70723	2.38739	5.89918
H	-2.09258	2.74533	4.84544
C	-0.41197	5.33921	-2.81616
H	-0.98484	6.02565	-2.17933
H	0.65535	5.46539	-2.57885
H	-0.57143	5.56713	-3.87563
H	5.99916	-4.01836	-2.30458
H	6.41205	-3.92401	-0.56927

PBE0+D3(BJ)/Def2-SVP Gibbs free energy:-2802.240061 a.u.

PBE0+D3(BJ)/Def2-SVP enthalpy:-2802.089899 a.u.

PBE0+D3(BJ)/Def2-SVP SCF energy:-2803.042463 a.u.

PBE0+D3(BJ)/Def2-TZVPP Gibbs free energy:-2803.084483 a.u.

PBE0+D3(BJ)/Def2-TZVPP enthalpy:-2802.934321 a.u.

PBE0+D3(BJ)/Def2-TZVPP SCF energy:-2803.886885 a.u.

IM1-*S*

Cartesian coordinates

ATOM	X	Y	Z
C	-0.46746	0.49825	-2.79641
O	0.25280	1.24641	-2.19490
O	-0.67518	-0.78007	-2.43929
C	-0.24339	-1.20324	-1.14966
H	-0.38370	-0.36815	-0.44861
C	-1.08335	-2.42869	-0.74381
C	-0.86519	-3.63084	-1.69322
H	-0.70813	-2.68783	0.25537
C	-3.28388	-2.19965	-1.81792
C	-3.03303	-3.33672	0.27553
C	-2.16532	-4.43138	-1.77269
H	-0.59322	-3.28092	-2.70063
H	-0.02583	-4.24449	-1.33702
H	-4.32369	-1.93141	-1.58266
H	-2.86794	-1.40358	-2.44218
C	-3.20062	-3.57709	-2.51329
C	-2.68824	-4.71215	-0.35369
H	-4.11706	-3.20790	0.38328
H	-2.59045	-3.24196	1.27924
H	-2.00188	-5.37305	-2.31634
H	-4.17849	-4.08356	-2.50754
H	-2.90627	-3.45794	-3.56700
H	-3.62031	-5.29458	-0.45451
N	-2.53854	-2.20626	-0.54246
C	-1.73491	-5.52510	0.53031
H	-2.22505	-5.67023	1.50797
H	-0.82583	-4.93694	0.74452
C	-1.34294	-6.88109	-0.04410
H	-0.73364	-6.77884	-0.95506
H	-0.75250	-7.46102	0.68004
H	-2.23335	-7.47601	-0.30380
C	1.21029	-1.63080	-1.13584
C	1.87387	-1.85529	0.10814
C	1.87871	-1.94425	-2.29528
C	1.33269	-1.46080	1.36179
C	3.14430	-2.49459	0.06246
C	3.16467	-2.52585	-2.22361
H	1.41043	-1.77975	-3.26707
C	2.03307	-1.70412	2.52247
H	0.40815	-0.88861	1.39398
C	3.80668	-2.78793	1.28858
H	3.69732	-2.77381	-3.14919

C	3.26609	-2.41252	2.48456
H	4.77134	-3.29432	1.23779
H	3.78099	-2.60324	3.42724
N	3.76321	-2.82486	-1.09932
O	1.65539	-1.27224	3.74125
Os	-3.18442	-0.06842	0.81475
O	-1.93856	-0.79150	1.71987
O	-4.62300	-0.96942	0.72599
O	-3.66044	1.24098	1.76846
O	-2.72391	0.59134	-0.67899
C	1.22208	6.04318	-1.88946
C	0.11030	5.52224	-1.26404
C	0.21891	4.30582	-0.55758
C	1.43848	3.61211	-0.49506
C	2.57379	4.18417	-1.11699
C	2.46078	5.37512	-1.80251
H	1.15037	6.98382	-2.43959
H	-0.85256	6.03216	-1.29489
C	-0.87153	3.72865	0.17922
C	1.44299	2.36766	0.24467
H	3.53783	3.68150	-1.04288
H	3.34297	5.81038	-2.27701
C	2.69006	1.56031	0.29953
C	3.30304	1.16820	-0.89033
C	3.28790	1.25991	1.52261
C	4.53951	0.51829	-0.86943
H	2.79944	1.38014	-1.83161
C	4.50538	0.57625	1.55307
H	2.78794	1.55685	2.44338
C	5.15812	0.21106	0.35538
O	5.15007	0.23458	2.68515
O	5.21324	0.17252	-1.99491
C	4.68033	0.53035	-3.23689
H	4.56892	1.62485	-3.33522
H	5.38793	0.17424	-3.99612
H	3.69770	0.05992	-3.41511
C	4.59073	0.59896	3.91758
H	4.49390	1.69517	4.01355
H	3.60345	0.13257	4.07463
H	5.28119	0.23850	4.69036
N	0.38895	1.93477	0.88295
N	-0.77651	2.62520	0.85818
O	-1.99742	4.44367	0.14397
C	-3.11545	4.12845	0.96938

H	-2.80364	3.37979	1.71100
C	-4.22630	3.54680	0.13883
C	-4.16185	3.29954	-1.16735
H	-5.14191	3.32521	0.69694
H	-5.01142	2.86968	-1.70270
H	-3.25221	3.50556	-1.73661
O	6.36750	-0.36484	0.48700
C	6.79307	-1.40271	-0.37375
H	5.95786	-2.04702	-0.68605
H	7.28770	-1.00144	-1.27046
H	7.51736	-1.99644	0.20181
C	-3.53683	5.41194	1.67192
H	-4.40546	5.22243	2.31909
H	-2.71653	5.79351	2.29610
H	-3.81134	6.18202	0.93584
C	-1.27796	0.85689	-4.00833
H	-0.87271	1.76341	-4.47121
H	-1.30857	0.02807	-4.72715
H	-2.30709	1.05563	-3.67144
C	0.49222	-0.47852	3.83834
H	0.53892	0.39423	3.16475
H	-0.40885	-1.05992	3.58735
H	0.43128	-0.14486	4.88117

PBE0+D3(BJ)/Def2-SVP Gibbs free energy:-2802.237277 a.u.

PBE0+D3(BJ)/Def2-SVP enthalpy:-2802.087790 a.u.

PBE0+D3(BJ)/Def2-SVP SCF energy:-2803.040096 a.u.

PBE0+D3(BJ)/Def2-TZVPP Gibbs free energy:-2803.084972 a.u.

PBE0+D3(BJ)/Def2-TZVPP enthalpy:-2802.935485 a.u.

PBE0+D3(BJ)/Def2-TZVPP SCF energy:-2803.887791 a.u.

IM2-*R*

Cartesian coordinates

ATOM	X	Y	Z
C	-0.21967	2.00138	0.78702
C	0.34790	3.02021	3.01004
C	-0.73759	2.88728	1.94202
H	-0.64203	0.99135	0.87768
H	-0.98394	3.89161	1.57166
H	-1.66257	2.45278	2.34451
C	1.99272	3.08784	1.13628
H	3.06023	2.83314	1.19051
H	1.84254	3.69405	0.24048
C	1.51176	3.80807	2.40911
H	2.32783	3.89331	3.14281

H	1.18728	4.83055	2.16509
H	-0.04815	3.55635	3.88424
C	0.85468	1.63111	3.42075
H	1.66289	1.77778	4.15799
C	1.46834	0.97080	2.15760
H	2.55024	0.81282	2.26115
H	1.02202	-0.01340	1.96331
N	1.26511	1.80261	0.93344
C	-0.21586	0.75660	4.08012
H	-1.04405	0.57424	3.37692
H	0.23029	-0.22901	4.28079
C	-0.67436	2.48271	-0.59491
H	-0.22970	1.86090	-1.37976
O	-0.20925	3.81323	-0.78918
C	-0.04633	4.20628	-2.07335
O	-0.39785	3.54575	-3.00815
C	-2.18366	2.39954	-0.73660
C	-2.81347	1.26010	-1.32437
C	-2.97984	3.44445	-0.32367
C	-2.10141	0.14924	-1.85754
C	-4.23624	1.29281	-1.42769
C	-4.38284	3.35195	-0.44664
H	-2.53521	4.35415	0.08119
C	-2.77935	-0.85080	-2.52364
H	-1.02226	0.08445	-1.74178
C	-4.90358	0.20977	-2.06680
H	-5.01136	4.17865	-0.09502
C	-4.19970	-0.82145	-2.61323
H	-5.99195	0.24915	-2.11425
H	-4.69936	-1.64456	-3.12541
N	-4.99547	2.31954	-0.96880
O	-2.19407	-1.89407	-3.13359
C	-0.78334	-1.92135	-3.21612
H	-0.52559	-2.79774	-3.82183
H	-0.40227	-1.01049	-3.70808
H	-0.32153	-2.03712	-2.22549
Os	2.25585	0.68183	-0.79996
O	0.98582	-0.42200	-0.51745
O	3.12785	-0.33687	-2.16998
O	2.30214	2.20437	-1.55664
O	3.86566	0.34714	0.16379
C	4.95170	-0.10293	-0.64251
C	4.41373	-0.84490	-1.86866
H	5.53653	0.77866	-0.95405

H	5.59255	-0.74326	-0.01817
H	5.05268	-0.63258	-2.74386
C	4.38380	-2.37524	-1.70710
H	3.60706	-2.76766	-2.37794
C	5.72511	-3.02168	-1.99309
H	5.66114	-4.10588	-1.83020
O	4.04206	-2.70870	-0.35789
C	2.76390	-2.91098	-0.02096
C	2.43535	-2.69472	1.35712
C	1.06896	-2.79180	1.68396
C	0.16226	-2.98369	0.57753
C	-1.28366	-2.65968	0.67021
C	-2.23368	-3.34986	-0.09016
C	-1.66706	-1.53526	1.38821
C	-3.55500	-2.91535	-0.08593
H	-1.90743	-4.18701	-0.70373
C	-2.98939	-1.07385	1.38773
H	-0.93531	-0.94887	1.93732
C	-3.95906	-1.78808	0.67313
N	1.90477	-3.32230	-0.90280
N	0.59599	-3.32532	-0.60744
O	-4.53744	-3.49622	-0.80434
O	-5.25451	-1.37587	0.62306
O	-3.17030	0.07422	2.07930
C	-4.18195	-4.44011	-1.78453
H	-5.10580	-4.69629	-2.31758
H	-3.45352	-4.01279	-2.49322
H	-3.76509	-5.35827	-1.33597
C	-6.19133	-2.18828	1.29186
H	-6.22847	-3.20093	0.86230
H	-5.95846	-2.25958	2.37012
H	-7.17145	-1.70916	1.16964
C	-4.41970	0.71986	2.20314
H	-5.13551	0.10884	2.77576
H	-4.21509	1.64342	2.76089
H	-4.85664	0.96907	1.22835
C	0.69173	-2.71604	3.04529
C	3.39918	-2.42433	2.35107
H	4.44388	-2.32001	2.05881
C	1.64699	-2.49369	4.01456
H	1.35024	-2.44625	5.06463
C	3.00317	-2.31854	3.66627
H	-0.35641	-2.83893	3.32037
H	3.74078	-2.11834	4.44611

C	-0.78206	1.32783	5.37477
H	-1.46221	0.60904	5.85469
H	0.01823	1.56504	6.09453
H	-1.35680	2.25041	5.20068
C	0.65618	5.53133	-2.14185
H	0.31607	6.20358	-1.34373
H	1.73293	5.34870	-2.00413
H	0.49644	5.98271	-3.12716
H	6.02407	-2.84255	-3.03548
H	6.50983	-2.62196	-1.33174

PBE0+D3(BJ)/Def2-SVP Gibbs free energy:-2802.300464 a.u.

PBE0+D3(BJ)/Def2-SVP enthalpy:-2802.157932 a.u.

PBE0+D3(BJ)/Def2-SVP SCF energy:-2803.113438 a.u.

PBE0+D3(BJ)/Def2-TZVPP Gibbs free energy:-2803.129734 a.u.

PBE0+D3(BJ)/Def2-TZVPP enthalpy:-2802.987202 a.u.

PBE0+D3(BJ)/Def2-TZVPP SCF energy:-2803.942708 a.u.

IM2-*S*

Cartesian coordinates

ATOM	X	Y	Z
C	-0.40181	0.76360	-2.53387
O	0.27281	1.42867	-1.79616
O	-0.83341	-0.47408	-2.23008
C	-0.66519	-0.90421	-0.88570
H	-0.78160	-0.02798	-0.23459
C	-1.72924	-1.97064	-0.56306
C	-1.52105	-3.27343	-1.36418
H	-1.58585	-2.17306	0.50682
C	-3.64034	-1.59355	-2.08213
C	-3.96710	-2.54138	0.11685
C	-2.87647	-3.92605	-1.62650
H	-1.03259	-3.05707	-2.32615
H	-0.84212	-3.93799	-0.81234
H	-4.64833	-1.16352	-2.10005
H	-2.98094	-0.93729	-2.65737
C	-3.63380	-3.03861	-2.61741
C	-3.68280	-3.99850	-0.32069
H	-5.02217	-2.27179	-0.02118
H	-3.72239	-2.39204	1.17853
H	-2.74151	-4.93079	-2.05143
H	-4.66107	-3.41194	-2.74947
H	-3.14890	-3.07380	-3.60425
H	-4.64661	-4.48213	-0.55419
N	-3.17134	-1.54936	-0.66569

C	-3.02038	-4.81398	0.79658
H	-3.69723	-4.80254	1.66742
H	-2.09894	-4.31113	1.13688
C	-2.70650	-6.25665	0.41949
H	-1.93123	-6.31722	-0.35949
H	-2.33927	-6.81990	1.28942
H	-3.60253	-6.77320	0.03960
C	0.70852	-1.50618	-0.66172
C	1.21134	-1.68161	0.66026
C	1.46238	-1.97115	-1.71501
C	0.55745	-1.17325	1.81371
C	2.44261	-2.38478	0.79846
C	2.67402	-2.64971	-1.46072
H	1.11948	-1.83991	-2.74261
C	1.12474	-1.33543	3.05819
H	-0.35223	-0.58520	1.70757
C	2.97451	-2.57915	2.10503
H	3.27264	-3.02587	-2.29867
C	2.33456	-2.07408	3.20100
H	3.92105	-3.11519	2.19204
H	2.73797	-2.19803	4.20799
N	3.13796	-2.87166	-0.25744
O	0.62754	-0.81968	4.19236
Os	-3.64813	0.49570	0.25514
O	-2.73613	-0.10775	1.56572
O	-5.22458	0.16972	-0.29476
O	-4.01663	2.24093	0.98931
O	-2.65105	1.47633	-1.03331
C	1.98776	5.60268	-1.74901
C	1.03198	5.11823	-0.88238
C	1.27062	3.91268	-0.19084
C	2.46281	3.18818	-0.39058
C	3.43813	3.71903	-1.26678
C	3.19924	4.90335	-1.93165
H	1.81549	6.53712	-2.28726
H	0.09643	5.65173	-0.71037
C	0.34426	3.34237	0.74189
C	2.60785	1.95901	0.35397
H	4.38211	3.18985	-1.40198
H	3.95953	5.30929	-2.60251
C	3.74492	1.02657	0.14399
C	4.05975	0.59712	-1.14780
C	4.43206	0.51896	1.24502
C	5.08482	-0.33232	-1.33688

H	3.46441	0.95619	-1.98619
C	5.44948	-0.41999	1.05638
H	4.12160	0.83043	2.24074
C	5.80593	-0.83072	-0.24055
O	6.13961	-0.99617	2.06058
O	5.43820	-0.82715	-2.54661
C	4.76389	-0.36870	-3.68253
H	4.88117	0.72091	-3.81898
H	5.20928	-0.88327	-4.54306
H	3.68496	-0.60357	-3.64366
C	5.89814	-0.56923	3.37280
H	6.10666	0.50793	3.49653
H	4.85818	-0.76679	3.68439
H	6.57882	-1.13915	4.01732
N	1.73826	1.59213	1.25537
N	0.60552	2.28566	1.45027
O	-0.83968	3.95345	0.86867
C	-1.90496	3.22454	1.48195
H	-1.57222	2.18602	1.60891
C	-3.11109	3.22918	0.52721
C	-2.75928	2.89270	-0.91379
H	-3.61767	4.20866	0.57358
H	-3.55870	3.24457	-1.58763
H	-1.80213	3.33200	-1.22928
O	6.83169	-1.69696	-0.39527
C	6.50580	-3.00939	-0.81249
H	5.56536	-3.35013	-0.35131
H	6.40268	-3.06217	-1.90863
H	7.33403	-3.65887	-0.49792
C	-2.23015	3.80329	2.84179
H	-3.09523	3.27279	3.26309
H	-1.37375	3.68501	3.52016
H	-2.47455	4.87364	2.76228
C	-0.89687	1.18857	-3.88477
H	-0.33289	2.06241	-4.22895
H	-0.82592	0.36752	-4.61013
H	-1.95859	1.45763	-3.77679
C	-0.46376	0.07057	4.09746
H	-0.22537	0.91779	3.43181
H	-1.36865	-0.43339	3.71973
H	-0.65276	0.43650	5.11390

PBE0+D3(BJ)/Def2-SVP Gibbs free energy:-2802.302559 a.u.

PBE0+D3(BJ)/Def2-SVP enthalpy:-2802.158087 a.u.

PBE0+D3(BJ)/Def2-SVP SCF energy:-2803.113564 a.u.

PBE0+D3(BJ)/Def2-TZVPP Gibbs free energy:-2803.139247 a.u.
PBE0+D3(BJ)/Def2-TZVPP enthalpy:-2802.994775 a.u.
PBE0+D3(BJ)/Def2-TZVPP SCF energy:-2803.950252 a.u.

***R*-pi-O-frag1**

Cartesian coordinates

ATOM	X	Y	Z
C	3.33073	-1.04687	0.21685
H	3.60697	0.01458	0.15822
O	1.94108	-1.17374	-0.09467
C	1.16453	-0.08130	-0.09771
C	-0.24816	-0.30103	0.00078
C	-1.04844	0.85700	0.09120
C	-0.35221	2.12137	0.12030
N	1.69146	1.09655	-0.22890
N	0.93409	2.19518	-0.10730
C	-2.45668	0.70627	0.07793
C	-0.83387	-1.58459	-0.03927
H	-0.18678	-2.45993	-0.09978
C	-3.01769	-0.55014	0.00609
H	-4.10412	-0.65597	-0.01953
C	-2.20549	-1.70368	-0.03827
H	-3.09212	1.59184	0.09293
H	-2.66995	-2.69083	-0.08549
H	3.49402	-1.39721	1.21459
H	3.88352	-1.61011	-0.50571
H	-0.83530	3.05318	0.32826

PBE0+D3(BJ)/Def2-TZVPP SCF energy:-532.063959 a.u.

***R*-pi-O-frag2**

Cartesian coordinates

ATOM	X	Y	Z
C	-1.87386	-0.16875	-0.01446
H	-1.85993	0.68436	-0.70134
O	-0.55364	-0.70436	0.03148
C	0.46444	0.18328	0.01174
O	0.30160	1.36953	0.00434
C	1.78696	-0.53103	-0.01228
H	1.87375	-1.11034	-0.94324
H	2.59979	0.20055	0.04504
H	1.85129	-1.23786	0.82661
H	-2.17739	0.16687	0.95515

H	-2.53637	-0.92590	-0.37877

PBE0+D3(BJ)/Def2-TZVPP SCF energy:-268.199485 a.u.

_R_-pi-O-total

Cartesian coordinates

ATOM	X	Y	Z
C	3.75752	-0.92044	0.47589
H	3.45659	0.11881	0.64683
O	4.91783	-0.90063	-0.35191
C	5.81945	0.07651	-0.11303
O	5.70456	0.87406	0.77267
C	-2.23726	3.42624	-0.97353
H	-1.92088	3.64543	0.05520
O	-2.76201	2.09688	-1.01521
C	-2.52937	1.26470	0.00940
C	-2.70133	-0.13390	-0.25140
C	-2.36361	-1.00521	0.80520
C	-1.84299	-0.39303	2.00454
N	-2.20991	1.73455	1.17533
N	-1.85560	0.90524	2.16634
C	-2.61827	-2.38929	0.64551
C	-3.22297	-0.63414	-1.46376
H	-3.46505	0.06524	-2.26430
C	-3.15781	-2.86264	-0.53061
H	-3.36544	-3.92909	-0.63900
C	-3.44922	-1.98560	-1.59747
H	-2.41410	-3.07199	1.47044
H	-3.86775	-2.38213	-2.52486
C	6.94298	0.01302	-1.10978
H	6.54930	0.20587	-2.11856
H	7.69908	0.76415	-0.85817
H	7.39017	-0.99072	-1.11346
H	3.97465	-1.38171	1.41663
H	2.98207	-1.45029	-0.03678
H	-1.38747	3.48218	-1.62130
H	-3.01629	4.09779	-1.26857
H	-1.42197	-0.95194	2.81402

PBE0+D3(BJ)/Def2-TZVPP SCF energy:-800.263645 a.u.

_R_-pi-pi-frag1

Cartesian coordinates

ATOM	X	Y	Z
C	-1.29805	2.67870	0.09696
H	-0.33266	2.89367	-0.31134

C	-1.56265	1.18536	0.01201
C	-0.50085	0.23029	0.05650
C	-2.85280	0.71374	-0.09715
C	0.87800	0.58007	0.07580
C	-0.86903	-1.14922	0.03490
C	-3.09950	-0.67616	-0.09623
H	-3.69081	1.40520	-0.19294
C	1.84669	-0.40172	0.03285
H	1.17006	1.62487	0.11787
C	0.15855	-2.13427	0.05008
H	-4.13163	-1.04057	-0.16164
C	1.47390	-1.77541	0.04161
H	-0.14640	-3.17975	0.07932
H	2.26598	-2.52528	0.06575
N	-2.15424	-1.57719	-0.01798
O	3.16770	-0.16895	-0.02295
C	3.61268	1.16517	-0.12393
H	4.70545	1.12558	-0.19398
H	3.20355	1.64982	-1.02768
H	3.34793	1.74468	0.77158
H	-2.04560	3.20554	-0.45848
H	-1.32951	2.98884	1.12055

PBE0+D3(BJ)/Def2-TZVPP SCF energy:-555.345163 a.u.

_R_-pi-pi-frag2

Cartesian coordinates

ATOM	X	Y	Z
C	0.07519	-2.31895	0.37813
C	-1.14869	-1.66137	0.25491
C	1.27404	-1.61148	0.22839
C	-1.18044	-0.28914	0.00771
H	-2.06075	-2.25163	0.31209
C	1.24441	-0.23064	0.00548
H	2.22279	-2.14629	0.24380
C	0.01498	0.43928	-0.09148
O	-2.31288	0.41918	-0.18397
O	-0.00549	1.76055	-0.38759
O	2.33779	0.53945	-0.15997
C	-3.50908	-0.28709	-0.39493
H	-3.83681	-0.82251	0.51284
H	-4.26967	0.45819	-0.65807
H	-3.40057	-1.00834	-1.22230
C	-0.28206	2.61812	0.69407
H	-1.28307	2.42541	1.11524

H	0.47500	2.51300	1.49053
H	-0.24911	3.64488	0.30725
C	3.57915	-0.07723	-0.34277
H	3.90979	-0.62626	0.55866
H	3.56010	-0.77027	-1.20101
H	4.29926	0.72188	-0.55591
H	0.07271	-3.38053	0.51206

PBE0+D3(BJ)/Def2-TZVPP SCF energy:-575.387998 a.u.

***R*-pi-pi-total**

Cartesian coordinates

ATOM	X	Y	Z
C	-3.89031	1.63640	1.26457
H	-3.38756	2.56412	1.08713
C	-3.46533	0.62176	0.21709
C	-2.17546	0.66959	-0.39569
C	-4.32303	-0.38911	-0.15883
C	-1.22952	1.70773	-0.16896
C	-1.85783	-0.37241	-1.31896
C	-3.89503	-1.37523	-1.07375
H	-5.33797	-0.43284	0.23809
C	-0.03746	1.72828	-0.86371
H	-1.44905	2.49159	0.54939
C	-0.59370	-0.34819	-1.97307
H	-4.57799	-2.18756	-1.34952
C	0.28635	0.67123	-1.76030
H	-0.35486	-1.17731	-2.63822
H	1.26164	0.68910	-2.24894
N	-2.70810	-1.38324	-1.62387
O	0.88425	2.69989	-0.76989
C	0.59770	3.82486	0.03036
H	1.45450	4.50141	-0.06343
H	-0.31522	4.33661	-0.32141
H	0.49471	3.54779	1.08890
C	1.61899	0.73556	2.31044
C	2.62952	0.78858	1.35049
C	0.63295	-0.25603	2.24229
C	2.67477	-0.16449	0.33327
H	3.34192	1.60961	1.39463
C	0.69106	-1.22778	1.23749
H	-0.19817	-0.24110	2.94613
C	1.72226	-1.19404	0.28609
O	3.57776	-0.15869	-0.66944
O	1.72501	-2.08961	-0.72969

O	-0.21007	-2.21717	1.07947
C	4.35970	0.99450	-0.85097
H	5.07557	1.14111	-0.02382
H	4.92187	0.84752	-1.78131
H	3.72430	1.89121	-0.94411
C	2.65641	-3.13827	-0.60836
H	3.69137	-2.75751	-0.58798
H	2.46948	-3.73493	0.30136
H	2.53067	-3.78362	-1.48737
C	-1.40168	-2.17084	1.80928
H	-1.23101	-2.28715	2.89586
H	-1.94650	-1.22872	1.62808
H	-2.01999	-3.00464	1.45567
H	1.57406	1.51023	3.04716
H	-4.94805	1.78716	1.20661
H	-3.63469	1.27265	2.23784

PBE0+D3(BJ)/Def2-TZVPP SCF energy:-1130.746051 a.u.

_S_-pi-O-frag1

Cartesian coordinates

ATOM	X	Y	Z
C	-0.46821	0.18122	-0.04180
O	-0.35089	1.37663	-0.01845
O	0.56581	-0.67127	-0.12319
C	1.88112	-0.16220	0.07077
H	1.82334	0.71155	0.73519
C	-1.76794	-0.56524	0.04951
H	-2.60174	0.10864	-0.17625
H	-1.77520	-1.43433	-0.62098
H	-1.86610	-0.93260	1.08254
H	2.34201	0.11484	-0.85428
H	2.48849	-0.93370	0.49597

PBE0+D3(BJ)/Def2-TZVPP SCF energy:-268.198127 a.u.

_S_-pi-O-frag2

Cartesian coordinates

ATOM	X	Y	Z
C	2.19100	-1.71691	0.00705
C	0.81975	-1.58523	0.03104
C	0.24859	-0.29525	0.03267
C	1.05619	0.85496	0.02641
C	2.46151	0.69336	-0.02659
C	3.01189	-0.57073	-0.03318
H	2.64581	-2.70971	0.00549

H	0.16356	-2.45554	0.04407
C	-1.16694	-0.06239	0.01405
C	0.37112	2.12956	0.02763
H	3.10337	1.57305	-0.07110
H	4.09703	-0.68611	-0.07723
N	-0.92989	2.22181	-0.01208
N	-1.70726	1.11736	-0.00987
O	-1.91940	-1.16559	0.00171
C	-3.34110	-1.06633	-0.02293
H	-3.61603	-0.13110	-0.53164
H	-3.73428	-1.04943	0.97208
H	-3.70126	-1.90945	-0.57459
H	0.94515	3.03251	0.03596

PBE0+D3(BJ)/Def2-TZVPP SCF energy:-532.068434 a.u.

_S_-pi-O-total

Cartesian coordinates

ATOM	X	Y	Z
C	2.73967	-0.74124	0.84911
O	1.62128	-0.73788	0.41034
O	3.84042	-0.59251	0.09495
C	3.68513	-0.16764	-1.25493
H	2.77343	0.44216	-1.32679
C	-3.29885	-1.25156	1.17267
C	-2.63797	-0.04757	1.06356
C	-1.68776	0.12934	0.03591
C	-1.39435	-0.90760	-0.86626
C	-2.10687	-2.12530	-0.75073
C	-3.03775	-2.28859	0.25310
H	-4.03673	-1.40146	1.96360
H	-2.83521	0.77538	1.75061
C	-0.98347	1.36047	-0.17994
C	-0.40548	-0.62292	-1.88362
H	-1.92126	-2.92759	-1.46467
H	-3.58501	-3.23036	0.33296
N	0.15836	0.54814	-1.99950
N	-0.12534	1.54677	-1.13558
O	-1.28737	2.34080	0.67446
C	-0.65852	3.61488	0.56052
H	-0.39618	3.78055	-0.49435
C	3.10230	-0.87499	2.30013
H	2.25291	-1.28022	2.86104
H	3.99274	-1.50340	2.43107
H	3.33940	0.13223	2.67564

H	3.62197	-0.99614	-1.92909
H	4.55068	0.39315	-1.53995
H	0.23383	3.64797	1.15003
H	-1.37226	4.34304	0.88496
H	-0.14060	-1.38670	-2.58461

PBE0+D3(BJ)/Def2-TZVPP SCF energy:-800.271423 a.u.

_S_-pi-pi-frag1

Cartesian coordinates

ATOM	X	Y	Z
C	0.07057	-2.34358	0.19006
C	1.26685	-1.62447	0.14369
C	-1.15536	-1.68589	0.11873
C	1.23744	-0.23935	-0.03640
H	2.20806	-2.16094	0.25618
C	-1.18755	-0.29747	-0.05367
H	-2.07218	-2.26890	0.19180
C	0.00964	0.42628	-0.19024
O	-2.32184	0.42672	-0.13821
O	2.33822	0.53803	-0.10116
C	3.59378	-0.07717	-0.08282
H	3.71803	-0.78118	-0.92498
H	4.33799	0.72354	-0.17595
H	3.77285	-0.62304	0.86036
C	-3.55413	-0.24203	-0.08381
H	-4.33061	0.52941	-0.15568
H	-3.67053	-0.94385	-0.92822
H	-3.68423	-0.79532	0.86166
O	0.00171	1.74147	-0.50844
C	-0.22670	2.64790	0.54946
H	-0.03077	3.65306	0.15333
H	-1.27408	2.59629	0.89147
H	0.44739	2.44838	1.39847
H	0.10619	-3.41245	0.22389

PBE0+D3(BJ)/Def2-TZVPP SCF energy:-575.388286 a.u.

_S_-pi-pi-frag2

Cartesian coordinates

ATOM	X	Y	Z
C	-1.24682	2.64275	0.29439
H	-0.40334	2.98715	-0.32056
C	-1.55169	1.19097	-0.01724
C	-0.50054	0.22567	0.01161
C	-2.84187	0.74030	-0.16544

C	0.87590	0.57852	0.02798
C	-0.87781	-1.14569	0.02752
C	-3.09471	-0.65035	-0.21443
H	-3.67436	1.44494	-0.19759
C	1.83796	-0.40606	0.06527
H	1.16382	1.62157	-0.08643
C	0.14374	-2.12837	0.16269
H	-4.12381	-1.00825	-0.33469
C	1.46175	-1.77301	0.18532
H	-0.16182	-3.17243	0.22663
H	2.25240	-2.52139	0.26274
N	-2.16527	-1.56126	-0.08040
O	3.16086	-0.17944	-0.04165
C	3.58970	1.15130	-0.24485
H	4.67568	1.10759	-0.38977
H	3.10661	1.59880	-1.12929
H	3.36006	1.78498	0.62655
H	-1.01121	2.68866	1.33711
H	-2.08767	3.27658	0.10429

PBE0+D3(BJ)/Def2-TZVPP SCF energy:-555.344092 a.u.

_S_-pi-pi-total

Cartesian coordinates

ATOM	X	Y	Z
C	0.64656	3.90951	-0.73782
H	1.23085	3.46408	-1.55540
C	-0.05541	2.81872	0.04647
C	0.69229	1.70394	0.53204
C	-1.36277	2.94820	0.45104
C	2.00412	1.39484	0.08158
C	0.07181	0.88352	1.51445
C	-1.90167	2.02605	1.37812
H	-1.97124	3.78286	0.09932
C	2.67142	0.30793	0.60089
H	2.43655	1.96145	-0.74055
C	0.83133	-0.17221	2.09413
H	-2.94495	2.12932	1.69828
C	2.09392	-0.45175	1.65629
H	0.36299	-0.76034	2.88298
H	2.67360	-1.27524	2.07701
N	-1.20982	1.05955	1.92475
O	3.86008	-0.13837	0.15283
C	-0.13247	-0.05143	-2.29713
C	-1.43831	0.22680	-1.88757

C	0.58591	-1.09408	-1.71633
C	-2.05294	-0.58392	-0.93006
H	-1.95505	1.08181	-2.32146
C	-0.02488	-1.90271	-0.75117
H	1.61401	-1.26633	-2.03118
C	-1.36745	-1.68750	-0.39488
O	0.58512	-2.93615	-0.13627
O	-3.31057	-0.39905	-0.47790
C	-4.09105	0.60330	-1.06217
H	-4.22185	0.44141	-2.14705
H	-5.07335	0.56124	-0.57538
H	-3.65832	1.60741	-0.90677
C	1.90308	-3.25319	-0.49847
H	2.20171	-4.10394	0.12623
H	1.97005	-3.55018	-1.55980
H	2.59607	-2.41416	-0.31744
O	-2.02322	-2.54625	0.41969
C	-1.80803	-2.39342	1.80652
H	-2.53445	-3.04240	2.31328
H	-0.78920	-2.71280	2.08329
H	-1.96410	-1.34879	2.12182
C	4.44420	0.52708	-0.94810
H	5.34895	-0.03632	-1.20554
H	3.75758	0.55686	-1.81038
H	4.71645	1.56358	-0.69281
H	1.27846	4.42552	-0.04548
H	-0.04666	4.60873	-1.15668
H	0.29141	0.50990	-3.10344

PBE0+D3(BJ)/Def2-TZVPP SCF energy:-1130.745240 a.u.

(*R*, *S*)-1-2

Cartesian coordinates

ATOM	X	Y	Z
C	0.24503	2.29002	0.74971
C	1.29859	3.51833	2.67695
C	-0.02261	3.20712	1.97358
H	-0.28483	1.33355	0.86861
H	-0.48623	4.14915	1.65929
H	-0.72887	2.72769	2.66607
C	2.58655	3.07747	0.59360
H	3.60896	2.68955	0.71289
H	2.50876	3.46983	-0.42503
C	2.25463	4.14300	1.65616
H	3.17235	4.49180	2.15335

H	1.77707	5.01489	1.18641
H	1.12291	4.21595	3.51003
C	1.92499	2.22098	3.20230
H	2.95597	2.46319	3.51561
C	1.99156	1.23429	2.00803
H	2.98359	0.77488	1.91811
H	1.26737	0.41186	2.12647
N	1.68920	1.90704	0.71777
C	1.21352	1.63089	4.42137
H	0.95322	2.44604	5.11752
H	0.25681	1.17565	4.10975
C	-0.30253	2.86550	-0.56820
H	0.02549	2.25197	-1.41425
O	0.23616	4.17247	-0.75149
C	0.26097	4.64897	-2.01527
O	-0.18269	4.04956	-2.95209
C	-1.82064	2.91214	-0.54644
C	-2.61289	1.82585	-1.03005
C	-2.47441	4.01398	-0.03914
C	-2.07192	0.67115	-1.66090
C	-4.02962	1.94960	-0.90115
C	-3.88310	4.02001	0.05170
H	-1.91403	4.89065	0.28794
C	-2.90842	-0.29696	-2.17776
H	-0.99569	0.55381	-1.74218
C	-4.85790	0.90239	-1.39488
H	-4.39219	4.89307	0.47708
C	-4.31841	-0.18267	-2.01985
H	-5.93278	0.99716	-1.24475
H	-4.94688	-0.99434	-2.38930
N	-4.63651	3.02710	-0.34582
O	-2.49534	-1.38346	-2.84952
C	-1.11929	-1.52285	-3.12363
H	-1.00835	-2.44506	-3.70487
H	-0.74664	-0.66826	-3.71512
H	-0.53526	-1.62895	-2.19868
Os	2.26748	0.25773	-1.07305
O	0.85233	-0.41580	-0.39167
O	2.70971	-0.95067	-2.23857
O	2.25662	1.68097	-2.01079
O	3.72120	0.05711	-0.12474
C	5.09299	-1.27874	-0.83686
C	4.49139	-1.98564	-1.85643
H	5.79528	-0.47822	-1.06806

H	5.13557	-1.70604	0.16420
H	4.74959	-1.75610	-2.89187
C	3.85876	-3.33747	-1.63529
H	2.97207	-3.42937	-2.27699
C	4.82507	-4.47787	-1.91510
H	4.31481	-5.44172	-1.78017
O	3.44513	-3.43514	-0.27014
C	2.14575	-3.31188	0.03463
C	1.83590	-3.00151	1.39895
C	0.47290	-2.78178	1.68807
C	-0.44185	-2.84926	0.57322
C	-1.87094	-2.45201	0.66547
C	-2.81367	-3.18933	-0.05085
C	-2.25936	-1.30676	1.37093
C	-4.15317	-2.80097	-0.04419
H	-2.46836	-4.03571	-0.64072
C	-3.60642	-0.93003	1.39941
H	-1.50515	-0.69080	1.85893
C	-4.56220	-1.68537	0.70252
N	1.23874	-3.51271	-0.87046
N	-0.05138	-3.26912	-0.60282
O	-5.12300	-3.41655	-0.75226
O	-5.85006	-1.26805	0.66904
O	-4.08191	0.15594	2.04007
C	-4.73890	-4.33825	-1.74077
H	-4.29229	-5.24823	-1.30416
H	-5.65274	-4.61797	-2.27911
H	-4.02214	-3.88467	-2.44577
C	-6.73797	-1.98053	1.49723
H	-6.79597	-3.04277	1.20602
H	-6.43933	-1.90896	2.55753
H	-7.72827	-1.52263	1.37640
C	-3.17768	1.10219	2.53169
H	-2.55413	0.69810	3.35097
H	-2.52032	1.48232	1.73131
H	-3.77466	1.93687	2.91832
C	0.09356	-2.58203	3.03798
C	2.80842	-2.96180	2.42110
H	3.85472	-3.12656	2.16278
C	1.04796	-2.57728	4.03173
H	0.74463	-2.43830	5.07137
C	2.41412	-2.75328	3.72362
H	-0.96084	-2.46394	3.28808
H	3.15587	-2.73682	4.52485

C	2.06324	0.59607	5.14915
H	1.51361	0.13123	5.98115
H	2.37892	-0.20664	4.46785
H	2.97291	1.05843	5.56354
C	0.92313	5.99789	-2.05774
H	1.97503	5.89961	-1.75172
H	0.87005	6.40042	-3.07477
H	0.43377	6.68278	-1.35137
H	5.19420	-4.42094	-2.94917
H	5.68739	-4.43373	-1.23343

PBE0+D3(BJ)/Def2-SVP Gibbs free energy:-2802.211107 a.u.

PBE0+D3(BJ)/Def2-SVP enthalpy:-2802.065804 a.u.

PBE0+D3(BJ)/Def2-SVP SCF energy:-2803.016744 a.u.

PBE0+D3(BJ)/Def2-TZVPP Gibbs free energy:-2803.064235 a.u.

PBE0+D3(BJ)/Def2-TZVPP enthalpy:-2802.918932 a.u.

PBE0+D3(BJ)/Def2-TZVPP SCF energy:-2803.869872 a.u.

Imaginary frequency: -415.2830 cm-1

(*R*, *S*)-1-3

Cartesian coordinates

ATOM	X	Y	Z
C	0.41946	2.26845	0.71786
C	1.21466	3.30487	2.85465
C	0.12841	3.35501	1.78065
H	-0.21522	1.38778	0.89589
H	0.13937	4.35122	1.31777
H	-0.87399	3.21064	2.20711
C	2.80069	2.85630	0.97927
H	3.79486	2.38912	1.02211
H	2.74511	3.42445	0.04612
C	2.53580	3.74222	2.21492
H	3.35360	3.65336	2.94677
H	2.47903	4.80125	1.92194
H	0.96137	3.98394	3.68150
C	1.36509	1.86607	3.36899
H	2.15443	1.86963	4.14019
C	1.84473	1.00328	2.17267
H	2.87441	0.65080	2.31211
H	1.21382	0.10925	2.05062
N	1.81327	1.75736	0.89420
C	0.09730	1.29242	4.00791
H	-0.66168	1.12127	3.22575
H	0.34088	0.29054	4.39040
C	0.05204	2.71480	-0.70466

H	0.40200	1.98168	-1.43538
O	0.71526	3.94323	-0.98690
C	0.91175	4.22818	-2.29347
O	0.47225	3.55656	-3.18260
C	-1.45327	2.84978	-0.85005
C	-2.24402	1.78973	-1.39234
C	-2.09767	3.99707	-0.44296
C	-1.70206	0.58479	-1.91565
C	-3.65462	1.99710	-1.44482
C	-3.50584	4.07972	-0.51842
H	-1.53015	4.84823	-0.06540
C	-2.52245	-0.36694	-2.48477
H	-0.63416	0.40391	-1.86484
C	-4.47207	0.98604	-2.02412
H	-4.01247	4.98693	-0.16878
C	-3.93114	-0.16446	-2.51878
H	-5.54337	1.17902	-2.07158
H	-4.54941	-0.94228	-2.97028
N	-4.26222	3.12046	-0.98907
O	-2.09154	-1.49684	-3.06074
C	-0.70828	-1.64994	-3.28991
H	-0.58270	-2.60923	-3.80412
H	-0.32718	-0.83269	-3.92723
H	-0.14294	-1.68938	-2.34970
Os	2.53962	0.15336	-0.85739
O	0.98764	-0.42535	-0.43187
O	3.02976	-1.04280	-2.01673
O	2.80822	1.61228	-1.69842
O	3.80818	-0.23287	0.27793
C	5.13125	-1.69514	-0.32648
C	4.55986	-2.32679	-1.41024
H	5.94313	-0.98311	-0.47505
H	5.01078	-2.11439	0.67082
H	4.96932	-2.15968	-2.40789
C	3.69733	-3.55621	-1.25885
H	2.86750	-3.50630	-1.97733
C	4.47896	-4.84477	-1.46170
H	3.80280	-5.70699	-1.37908
O	3.15491	-3.56286	0.06280
C	1.85072	-3.32298	0.25456
C	1.47431	-2.90664	1.57417
C	0.11043	-2.59834	1.75656
C	-0.73272	-2.67503	0.58422
C	-2.15237	-2.23375	0.60202

C	-3.11673	-3.03931	-0.00227
C	-2.51690	-1.01627	1.18876
C	-4.46423	-2.67121	0.04921
H	-2.78905	-3.94400	-0.51068
C	-3.86494	-0.64723	1.23974
H	-1.74522	-0.35829	1.58690
C	-4.85280	-1.51012	0.73735
N	0.99862	-3.50573	-0.70515
N	-0.29210	-3.16755	-0.54293
O	-5.46421	-3.37855	-0.51248
O	-6.16710	-1.25194	0.93276
O	-4.32289	0.50626	1.76964
C	-5.13553	-4.49991	-1.28702
H	-6.07750	-4.88366	-1.69762
H	-4.45870	-4.23599	-2.11825
H	-4.66283	-5.29303	-0.68193
C	-6.77104	-0.30819	0.07921
H	-7.81883	-0.22081	0.39485
H	-6.28583	0.67765	0.15898
H	-6.74278	-0.64872	-0.96979
C	-3.40468	1.46318	2.21315
H	-3.98535	2.34807	2.50003
H	-2.83804	1.10741	3.09231
H	-2.69593	1.75055	1.41659
C	-0.34452	-2.30479	3.06375
C	2.38251	-2.83689	2.65097
H	3.43415	-3.06054	2.47225
C	0.54548	-2.28084	4.11705
H	0.18104	-2.07490	5.12586
C	1.91946	-2.52065	3.90963
H	-1.40495	-2.11794	3.23432
H	2.61118	-2.47737	4.75338
C	-0.50125	2.13438	5.12724
H	-0.89424	3.09388	4.75733
H	-1.33450	1.60667	5.61471
H	0.24910	2.35996	5.90192
C	1.76684	5.45297	-2.45138
H	1.68395	5.82885	-3.47691
H	1.48244	6.22806	-1.72803
H	2.81300	5.17182	-2.25671
H	4.94101	-4.85896	-2.45931
H	5.27307	-4.94156	-0.70666

PBE0+D3(BJ)/Def2-SVP Gibbs free energy:-2802.213624 a.u.

PBE0+D3(BJ)/Def2-SVP enthalpy:-2802.066644 a.u.

PBE0+D3(BJ)/Def2-SVP SCF energy:-2803.017747 a.u.
PBE0+D3(BJ)/Def2-TZVPP Gibbs free energy:-2803.066307 a.u.
PBE0+D3(BJ)/Def2-TZVPP enthalpy:-2802.919327 a.u.
PBE0+D3(BJ)/Def2-TZVPP SCF energy:-2803.870430 a.u.
Imaginary frequency: -421.8919 cm-1

(*R*, *S*)-3-31

Cartesian coordinates

ATOM	X	Y	Z
C	-2.04395	-1.26566	0.85576
C	-0.50545	-2.80405	2.08291
C	-1.93669	-2.26321	2.03012
H	-2.44574	-1.78937	-0.02231
H	-2.17172	-1.76220	2.98190
H	-2.67638	-3.06688	1.90613
C	0.15808	-0.45083	1.59813
H	1.09705	-0.05446	1.19479
H	-0.34657	0.36623	2.12102
C	0.41368	-1.65882	2.52165
H	1.46646	-1.97797	2.46746
H	0.21082	-1.38901	3.56901
H	-0.43633	-3.63551	2.79881
C	-0.06662	-3.26315	0.68124
H	0.96492	-3.64542	0.76819
C	-0.04038	-1.99995	-0.21674
H	0.98900	-1.70395	-0.45394
H	-0.56269	-2.17873	-1.17022
N	-0.67995	-0.83364	0.43738
C	-0.91845	-4.38466	0.07560
H	-1.94104	-4.01906	-0.12236
H	-0.49773	-4.62750	-0.91521
C	-3.05432	-0.13794	1.11429
H	-3.11475	0.52331	0.24118
O	-2.61216	0.63609	2.22124
C	-2.93015	1.94555	2.22323
O	-3.69371	2.44122	1.44142
C	-4.43009	-0.72192	1.36715
C	-5.21475	-1.23324	0.28877
C	-4.93656	-0.81722	2.64226
C	-4.81371	-1.16152	-1.07719
C	-6.46044	-1.83775	0.62376
C	-6.19428	-1.42626	2.85429
H	-4.37146	-0.42895	3.49061
C	-5.62089	-1.68899	-2.06438

H	-3.87672	-0.67385	-1.34286
C	-7.26372	-2.37387	-0.42448
H	-6.59148	-1.50004	3.87342
C	-6.85885	-2.30731	-1.72652
H	-8.21200	-2.83415	-0.14275
H	-7.46558	-2.71174	-2.53849
N	-6.92800	-1.92770	1.89401
O	-5.33530	-1.66877	-3.37737
C	-4.13315	-1.07408	-3.79740
H	-3.25485	-1.58062	-3.36366
H	-4.10361	-1.16497	-4.88959
H	-4.08456	-0.00865	-3.51761
Os	-0.54970	1.06773	-1.20558
O	-1.84009	0.16720	-1.87773
O	-0.43430	2.42418	-2.26572
O	-0.96186	2.00361	0.19364
O	1.04635	0.47311	-1.22165
C	-1.29413	4.08279	-0.18663
C	-0.66824	4.34152	-1.37595
H	-2.37497	3.94945	-0.12859
H	-0.76164	4.22912	0.75120
H	-1.26359	4.51548	-2.27346
C	0.77062	4.79493	-1.45580
H	1.27698	4.25927	-2.27141
C	0.88921	6.29507	-1.67234
H	0.37160	6.58896	-2.59667
O	1.42779	4.47349	-0.22606
C	2.23246	3.40377	-0.16637
C	2.35547	2.77386	1.11631
C	3.15562	1.61475	1.15698
C	3.75959	1.20402	-0.08700
C	4.48108	-0.09453	-0.19192
C	3.95066	-1.05503	-1.05380
C	5.62004	-0.36736	0.56939
C	4.55361	-2.31357	-1.14355
H	3.07241	-0.79135	-1.64115
C	6.23162	-1.62284	0.47072
H	6.03398	0.40898	1.21242
C	5.68075	-2.61296	-0.36085
N	2.84558	2.98537	-1.23105
N	3.60882	1.87976	-1.19525
O	4.11707	-3.30740	-1.94318
O	6.23855	-3.84745	-0.40451
O	7.34727	-1.98091	1.13762

C	3.00857	-3.06933	-2.76868
H	2.10256	-2.83848	-2.18238
H	3.19114	-2.23997	-3.47308
H	2.83801	-3.99171	-3.33690
C	7.19027	-4.03029	-1.42783
H	8.03891	-3.33442	-1.31263
H	7.56080	-5.06027	-1.34515
H	6.73806	-3.89115	-2.42438
C	7.94634	-1.05528	2.00064
H	8.27682	-0.14748	1.46610
H	7.26701	-0.75778	2.81895
H	8.82367	-1.55131	2.43323
C	3.26193	0.90457	2.37874
C	1.68428	3.22573	2.27354
H	1.08091	4.13205	2.21385
C	2.59915	1.35345	3.49989
H	2.67949	0.79556	4.43526
C	1.80871	2.52231	3.45105
H	3.85456	-0.00999	2.41701
H	1.29360	2.86495	4.35106
C	-0.98227	-5.65184	0.91957
H	0.02792	-6.01885	1.16154
H	-1.51232	-5.48399	1.86951
H	-1.51197	-6.45513	0.38724
C	-2.17044	2.67210	3.29432
H	-2.18933	2.11224	4.23876
H	-1.12183	2.74536	2.96682
H	-2.58646	3.67626	3.43037
H	0.44146	6.84492	-0.83142
H	1.94729	6.57791	-1.76013

PBE0+D3(BJ)/Def2-SVP Gibbs free energy:-2802.204760 a.u.

PBE0+D3(BJ)/Def2-SVP enthalpy:-2802.055716 a.u.

PBE0+D3(BJ)/Def2-SVP SCF energy:-2803.006654 a.u.

PBE0+D3(BJ)/Def2-TZVPP Gibbs free energy:-2803.062680 a.u.

PBE0+D3(BJ)/Def2-TZVPP enthalpy:-2802.913636 a.u.

PBE0+D3(BJ)/Def2-TZVPP SCF energy:-2803.864574 a.u.

Imaginary frequency: -408.8332 cm-1

(*R*, *S*)-4a-10

Cartesian coordinates

ATOM	X	Y	Z
C	-3.35259	1.41351	-0.21327
C	-5.46401	2.48362	-1.01036
C	-4.25954	2.65809	-0.08497

H	-2.53204	1.63523	-0.90908
H	-4.61322	2.77180	0.95098
H	-3.68582	3.56513	-0.32231
C	-5.44164	0.09796	-0.26930
H	-5.87830	-0.77826	-0.76562
H	-5.29882	-0.15306	0.78498
C	-6.32756	1.35007	-0.44764
H	-7.16755	1.14415	-1.12896
H	-6.76233	1.65282	0.51687
H	-6.05057	3.41257	-1.05249
C	-4.98829	2.08324	-2.41718
H	-5.88692	1.96680	-3.04693
C	-4.29748	0.70105	-2.28681
H	-4.88705	-0.09314	-2.76212
H	-3.30822	0.70848	-2.76809
N	-4.10213	0.30365	-0.86957
C	-4.08075	3.11216	-3.10174
H	-3.12295	3.18898	-2.55836
H	-3.81926	2.71904	-4.09873
C	-2.65069	1.05884	1.10681
H	-1.98614	0.19582	0.97059
O	-3.65325	0.70091	2.05199
C	-3.33697	-0.15053	3.04099
O	-2.21148	-0.47311	3.30788
C	-1.83028	2.24101	1.58117
C	-0.59295	2.57701	0.95051
C	-2.27733	3.04074	2.60735
C	0.02183	1.77982	-0.05991
C	0.07005	3.74773	1.41948
C	-1.52686	4.17259	2.99585
H	-3.21058	2.80926	3.12204
C	1.25365	2.13892	-0.56572
H	-0.45530	0.85910	-0.39426
C	1.31282	4.11577	0.82860
H	-1.88975	4.80572	3.81378
C	1.88759	3.33787	-0.13211
H	1.79008	5.02737	1.19102
H	2.84812	3.59670	-0.57803
N	-0.40504	4.52659	2.42300
O	1.95827	1.42675	-1.46321
C	1.42181	0.21988	-1.95500
H	2.17836	-0.20763	-2.62248
H	1.21463	-0.49233	-1.14134
H	0.48488	0.39268	-2.50841

Os	-2.89400	-1.88938	-0.97559
O	-1.70068	-0.79519	-1.53608
O	-2.06426	-3.40401	-1.08468
O	-3.06662	-1.94615	0.75731
O	-4.33437	-2.24539	-1.81796
C	-2.14590	-3.59490	1.67486
C	-1.44844	-4.28242	0.70442
H	-3.11281	-3.97309	2.00715
H	-1.64746	-2.86919	2.31654
H	-1.86599	-5.21536	0.32058
C	0.03448	-4.10388	0.45670
H	0.56682	-4.78618	1.14743
C	0.45596	-4.44619	-0.97240
H	1.52487	-4.67188	-1.01368
O	0.32370	-2.76877	0.86943
C	1.56075	-2.26381	0.85547
C	1.72673	-0.99971	1.51597
C	2.98027	-0.37679	1.34527
C	3.94460	-1.08448	0.53548
C	5.24887	-0.48099	0.15121
C	5.27907	0.78827	-0.43457
C	6.42034	-1.22156	0.31147
C	6.50051	1.32180	-0.85974
H	4.34097	1.31618	-0.60784
C	7.64203	-0.68228	-0.10117
H	6.34955	-2.22203	0.73486
C	7.69145	0.60406	-0.66475
N	2.53544	-2.89999	0.28029
N	3.72988	-2.30081	0.10644
O	6.63521	2.51500	-1.47518
O	8.87965	1.14925	-1.02435
O	8.82803	-1.31568	0.00216
C	5.50730	3.33251	-1.61075
H	5.09656	3.61534	-0.62484
H	5.83734	4.24062	-2.12988
H	4.70834	2.85073	-2.20149
C	9.30848	0.82483	-2.32624
H	9.44964	-0.26328	-2.44204
H	8.58986	1.18135	-3.08417
H	10.27151	1.32749	-2.48501
C	8.85990	-2.60012	0.56177
H	9.90870	-2.92097	0.54950
H	8.49886	-2.60052	1.60484
H	8.25818	-3.31797	-0.02220

C	3.23121	0.83738	2.02603
C	0.74089	-0.42467	2.34077
H	-0.22830	-0.90458	2.45949
C	2.26367	1.38362	2.84352
H	2.46467	2.31946	3.36906
C	1.01505	0.75130	3.00387
H	4.19993	1.32540	1.91446
H	0.25046	1.19557	3.64419
C	-4.69704	4.49785	-3.25102
H	-5.67433	4.44612	-3.75718
H	-4.85273	4.98419	-2.27593
H	-4.04753	5.15715	-3.84488
C	-4.57091	-0.65704	3.72853
H	-4.99210	-1.45952	3.10338
H	-4.30826	-1.06874	4.70916
H	-5.32698	0.13254	3.82441
H	-0.12470	-5.31172	-1.32295
H	0.24643	-3.60899	-1.64934

PBE0+D3(BJ)/Def2-SVP Gibbs free energy:1.754868 a.u.

PBE0+D3(BJ)/Def2-SVP enthalpy:1.753838 a.u.

PBE0+D3(BJ)/Def2-SVP SCF energy:0.950136 a.u.

PBE0+D3(BJ)/Def2-TZVPP Gibbs free energy:1.754868 a.u.

PBE0+D3(BJ)/Def2-TZVPP enthalpy:1.753838 a.u.

PBE0+D3(BJ)/Def2-TZVPP SCF energy:0.950136 a.u.

Imaginary frequency: -71.7242 cm-1

(*R*, *S*)-4a-14

Cartesian coordinates

ATOM	X	Y	Z
C	-3.35248	1.41347	-0.21347
C	-5.46390	2.48345	-1.01073
C	-4.25940	2.65809	-0.08540
H	-2.53183	1.63511	-0.90918
H	-4.61306	2.77202	0.95054
H	-3.68567	3.56507	-0.32293
C	-5.44149	0.09780	-0.26963
H	-5.87812	-0.77830	-0.76619
H	-5.29879	-0.15345	0.78460
C	-6.32738	1.34996	-0.44781
H	-7.16755	1.14407	-1.12891
H	-6.76188	1.65278	0.51680
H	-6.05050	3.41238	-1.05295
C	-4.98822	2.08294	-2.41753
H	-5.88689	1.96627	-3.04718

C	-4.29716	0.70088	-2.28703
H	-4.88649	-0.09343	-2.76242
H	-3.30782	0.70850	-2.76815
N	-4.10193	0.30355	-0.86976
C	-4.08090	3.11190	-3.10231
H	-3.12307	3.18894	-2.55901
H	-3.81942	2.71868	-4.09926
C	-2.65075	1.05887	1.10672
H	-1.98609	0.19593	0.97059
O	-3.65343	0.70084	2.05174
C	-3.33705	-0.15034	3.04093
O	-2.21149	-0.47252	3.30801
C	-1.83051	2.24111	1.58122
C	-0.59315	2.57729	0.95073
C	-2.27780	3.04073	2.60739
C	0.02188	1.78029	-0.05969
C	0.06965	3.74807	1.41987
C	-1.52751	4.17263	2.99607
H	-3.21109	2.80912	3.12193
C	1.25370	2.13964	-0.56534
H	-0.45493	0.85947	-0.39426
C	1.31244	4.11633	0.82920
H	-1.89058	4.80567	3.81399
C	1.88743	3.33862	-0.13153
H	1.78954	5.02796	1.19177
H	2.84798	3.59763	-0.57732
N	-0.40566	4.52680	2.42339
O	1.95849	1.42770	-1.46288
C	1.42225	0.22082	-1.95490
H	2.17890	-0.20644	-2.62243
H	1.21516	-0.49158	-1.14139
H	0.48532	0.39356	-2.50834
Os	-2.89365	-1.88943	-0.97554
O	-1.70026	-0.79514	-1.53565
O	-2.06385	-3.40402	-1.08454
O	-3.06672	-1.94637	0.75731
O	-4.33378	-2.24543	-1.81831
C	-2.14601	-3.59504	1.67491
C	-1.44851	-4.28269	0.70459
H	-3.11295	-3.97316	2.00719
H	-1.64756	-2.86931	2.31657
H	-1.86613	-5.21559	0.32070
C	0.03447	-4.10439	0.45710
H	0.56663	-4.78634	1.14831

C	0.45621	-4.44751	-0.97172
H	0.24658	-3.61082	-1.64925
O	0.32366	-2.76909	0.86921
C	1.56069	-2.26408	0.85523
C	1.72660	-1.00002	1.51581
C	2.98008	-0.37698	1.34516
C	3.94447	-1.08454	0.53532
C	5.24873	-0.48098	0.15113
C	6.42020	-1.22158	0.31125
C	5.27891	0.78838	-0.43445
C	7.64188	-0.68223	-0.10133
H	6.34941	-2.22211	0.73447
C	6.50035	1.32197	-0.85955
H	4.34081	1.31631	-0.60763
C	7.69130	0.60419	-0.66471
N	2.53541	-2.90013	0.27995
N	3.72981	-2.30085	0.10617
O	8.82788	-1.31565	0.00187
O	8.87949	1.14944	-1.02425
O	6.63503	2.51526	-1.47482
C	8.85976	-2.60019	0.56126
H	8.25802	-3.31793	-0.02283
H	9.90856	-2.92104	0.54890
H	8.49874	-2.60077	1.60433
C	9.30830	0.82521	-2.32619
H	8.58966	1.18183	-3.08406
H	10.27132	1.32790	-2.48490
H	9.44947	-0.26288	-2.44214
C	5.50714	3.33282	-1.61019
H	5.09644	3.61546	-0.62421
H	5.83717	4.24102	-2.12916
H	4.70814	2.85116	-2.20099
C	3.23091	0.83717	2.02601
C	0.74071	-0.42514	2.34066
H	-0.22843	-0.90517	2.45931
C	2.26331	1.38326	2.84354
H	2.46423	2.31907	3.36915
C	1.01474	0.75082	3.00384
H	4.19958	1.32528	1.91449
H	0.25009	1.19496	3.64417
C	-4.69741	4.49747	-3.25174
H	-5.67474	4.44552	-3.75781
H	-4.85308	4.98395	-2.27672
H	-4.04805	5.15678	-3.84576

C	-4.57095	-0.65700	3.72844
H	-5.32651	0.13293	3.82553
H	-4.99294	-1.45851	3.10260
H	-4.30809	-1.06987	4.70852
H	1.52518	-4.67295	-1.01272
H	-0.12418	-5.31343	-1.32175

PBE0+D3(BJ)/Def2-SVP Gibbs free energy:1.754754 a.u.
PBE0+D3(BJ)/Def2-SVP enthalpy:1.753795 a.u.
PBE0+D3(BJ)/Def2-SVP SCF energy:0.950087 a.u.
PBE0+D3(BJ)/Def2-TZVPP Gibbs free energy:1.754754 a.u.
PBE0+D3(BJ)/Def2-TZVPP enthalpy:1.753795 a.u.
PBE0+D3(BJ)/Def2-TZVPP SCF energy:0.950087 a.u.
Imaginary frequency: -42.6299 cm-1

_(R, R)_-4a-15

Cartesian coordinates

ATOM	X	Y	Z
C	2.49898	1.60240	-0.89732
C	4.09143	2.77806	-2.40704
C	2.70095	2.85666	-1.77289
H	2.77757	1.84988	0.13424
H	1.93724	2.90250	-2.56512
H	2.57644	3.76183	-1.16125
C	3.52429	0.37583	-2.76340
H	4.16625	-0.49230	-2.96633
H	2.51401	0.12993	-3.10400
C	4.06766	1.65041	-3.44419
H	5.08096	1.48557	-3.84252
H	3.42933	1.93088	-4.29546
H	4.34737	3.73008	-2.89370
C	5.13707	2.42342	-1.33348
H	6.12015	2.38918	-1.83357
C	4.79860	0.99621	-0.82561
H	5.53143	0.25772	-1.17313
H	4.79713	0.95829	0.27528
N	3.46754	0.54235	-1.29207
C	5.24719	3.43541	-0.18633
H	4.31687	3.43868	0.40791
H	6.03198	3.07843	0.50209
C	1.03374	1.16572	-0.79219
H	0.94658	0.30163	-0.12102
O	0.56766	0.77651	-2.07725
C	-0.41550	-0.14462	-2.10087
O	-0.97718	-0.51709	-1.10815

C	0.19267	2.29207	-0.22392
C	0.28526	2.64697	1.15653
C	-0.63743	3.03560	-1.02986
C	1.12535	1.96835	2.08358
C	-0.50674	3.74410	1.60172
C	-1.36877	4.11250	-0.47953
H	-0.72927	2.80457	-2.09193
C	1.18727	2.38567	3.39543
H	1.68344	1.08799	1.76847
C	-0.43179	4.13080	2.97078
H	-2.01425	4.71224	-1.13205
C	0.39558	3.48009	3.84167
H	-1.04959	4.97222	3.28832
H	0.46956	3.77210	4.89067
N	-1.32058	4.45633	0.78181
O	1.93949	1.79892	4.34527
C	2.81364	0.76343	3.96539
H	3.53569	1.10707	3.20527
H	3.35330	0.46176	4.87062
H	2.27265	-0.10272	3.55274
Os	3.10468	-1.77019	-0.28665
O	2.80109	-0.78035	1.07863
O	2.84149	-3.36658	0.32173
O	1.81857	-1.83695	-1.45749
O	4.65461	-1.92101	-0.97811
C	0.77656	-3.61948	-1.58591
C	1.20486	-4.42792	-0.55673
H	1.17987	-3.77039	-2.58757
H	-0.16243	-3.07184	-1.49568
H	1.93551	-5.20984	-0.77561
C	0.43059	-4.59893	0.73079
H	-0.53052	-5.07410	0.47389
C	1.16430	-5.42694	1.76955
H	0.53806	-5.54800	2.66392
O	0.14084	-3.33184	1.32562
C	-1.03398	-2.73308	1.10354
C	-1.30150	-1.57187	1.89900
C	-2.49200	-0.88384	1.59533
C	-3.30189	-1.43663	0.53329
C	-4.53893	-0.75985	0.05911
C	-5.72202	-1.48279	-0.06512
C	-4.50025	0.59494	-0.30182
C	-6.87844	-0.85421	-0.53091
H	-5.75521	-2.54366	0.18303

C	-5.65404	1.22701	-0.76828
H	-3.55316	1.13169	-0.24157
C	-6.85864	0.50365	-0.86779
N	-1.86445	-3.21335	0.22945
N	-3.00873	-2.56522	-0.05422
O	-8.01491	-1.58484	-0.68608
O	-7.99025	1.12321	-1.29931
O	-5.71459	2.51626	-1.16130
C	-9.07689	-1.27267	0.19037
H	-9.42217	-0.23607	0.05534
H	-9.89684	-1.96362	-0.04447
H	-8.77569	-1.42198	1.24215
C	-8.16222	1.11440	-2.69941
H	-9.10906	1.62737	-2.91278
H	-7.34218	1.65329	-3.20300
H	-8.21430	0.08263	-3.08640
C	-4.55316	3.29374	-1.07498
H	-3.74496	2.89147	-1.71121
H	-4.81831	4.29854	-1.42685
H	-4.17792	3.36746	-0.03955
C	-2.83812	0.24077	2.38203
C	-0.47182	-1.15819	2.96082
H	0.43966	-1.72069	3.16593
C	-2.02058	0.63846	3.41883
H	-2.29358	1.50768	4.02080
C	-0.83612	-0.06613	3.71677
H	-3.76404	0.77890	2.17831
H	-0.20569	0.26390	4.54470
C	5.57358	4.85661	-0.62925
H	5.74802	5.51051	0.23750
H	6.48044	4.87991	-1.25455
H	4.75433	5.30109	-1.21471
C	-0.67369	-0.64172	-3.49069
H	-0.67128	0.18454	-4.21364
H	0.14208	-1.32992	-3.75912
H	-1.62745	-1.18035	-3.51401
H	1.38714	-6.42543	1.36621
H	2.10802	-4.94298	2.05313

PBE0+D3(BJ)/Def2-SVP Gibbs free energy:-2802.205331 a.u.

PBE0+D3(BJ)/Def2-SVP enthalpy:-2802.058846 a.u.

PBE0+D3(BJ)/Def2-SVP SCF energy:-2803.010034 a.u.

PBE0+D3(BJ)/Def2-TZVPP Gibbs free energy:-2803.061111 a.u.

PBE0+D3(BJ)/Def2-TZVPP enthalpy:-2802.914626 a.u.

PBE0+D3(BJ)/Def2-TZVPP SCF energy:-2803.865814 a.u.

Imaginary frequency: -379.4463 cm-1

(*R*, *R*)-4a-6

Cartesian coordinates

ATOM	X	Y	Z
C	2.19238	1.72094	-0.92364
C	3.66473	3.07197	-2.40337
C	2.26157	2.98933	-1.79752
H	2.42672	2.00121	0.10921
H	1.51207	2.94588	-2.60325
H	2.02127	3.87295	-1.18867
C	3.38700	0.62040	-2.76949
H	4.12964	-0.16667	-2.95905
H	2.41951	0.25956	-3.13083
C	3.79155	1.95090	-3.44041
H	4.82457	1.90539	-3.81931
H	3.14106	2.15727	-4.30359
H	3.82025	4.04785	-2.88527
C	4.72322	2.83720	-1.30895
H	5.71343	2.91986	-1.78902
C	4.54341	1.37838	-0.80924
H	5.36267	0.73008	-1.14429
H	4.52693	1.33683	0.29148
N	3.28209	0.77706	-1.30009
C	4.69110	3.85071	-0.15817
H	3.75527	3.74195	0.41729
H	5.49912	3.58612	0.54507
C	0.78825	1.11526	-0.84393
H	0.79600	0.23788	-0.18346
O	0.39997	0.69241	-2.14397
C	-0.44301	-0.35433	-2.20843
O	-0.95335	-0.83708	-1.23629
C	-0.20615	2.11100	-0.27756
C	-0.13922	2.51287	1.09272
C	-1.18291	2.66945	-1.06656
C	0.82396	2.00804	2.01080
C	-1.09032	3.47545	1.53625
C	-2.09574	3.59613	-0.51114
H	-1.27440	2.39514	-2.11749
C	0.87113	2.48773	3.30160
H	1.49000	1.20117	1.71015
C	-1.02095	3.93294	2.88327
H	-2.88946	4.00376	-1.14473
C	-0.06103	3.46855	3.73834

H	-1.75582	4.67570	3.19779
H	0.00849	3.81982	4.76938
N	-2.05944	3.99058	0.73582
O	1.74385	2.06490	4.23677
C	2.75973	1.17225	3.84918
H	3.38491	1.60206	3.04735
H	3.37860	0.99752	4.73717
H	2.35378	0.21382	3.48834
Os	3.16729	-1.57324	-0.29502
O	2.74390	-0.62321	1.06592
O	3.06745	-3.19123	0.30805
O	1.90665	-1.77693	-1.47862
O	4.73181	-1.55189	-0.97027
C	1.02884	-3.63328	-1.59258
C	1.54563	-4.40405	-0.57384
H	1.42909	-3.74507	-2.60061
H	0.04414	-3.17472	-1.48731
H	2.34740	-5.10792	-0.80868
C	0.80406	-4.66799	0.71927
H	-0.13244	-5.19052	0.46446
C	1.61197	-5.48631	1.70938
H	2.53398	-4.95629	1.98321
O	0.45538	-3.44559	1.37102
C	-0.74300	-2.88507	1.18306
C	-1.02170	-1.73912	1.99751
C	-2.22417	-1.06743	1.70982
C	-3.02459	-1.61098	0.63694
C	-4.21610	-0.88522	0.12544
C	-4.10678	0.46524	-0.20618
C	-5.42417	-1.56244	-0.07418
C	-5.21222	1.14728	-0.71432
H	-3.15919	0.99577	-0.10732
C	-6.53496	-0.88086	-0.57365
H	-5.46337	-2.62638	0.15579
C	-6.43856	0.49433	-0.86882
N	-1.58999	-3.38912	0.33750
N	-2.74246	-2.74954	0.06356
O	-5.09792	2.45223	-1.09116
O	-7.52829	1.18307	-1.29819
O	-7.73883	-1.44308	-0.80645
C	-5.59034	3.38833	-0.15374
H	-5.51888	4.37968	-0.62029
H	-6.64356	3.18326	0.09662
H	-4.97483	3.38831	0.76261

C	-7.71749	1.17228	-2.69536
H	-6.85956	1.63199	-3.21474
H	-7.86445	0.14486	-3.06876
H	-8.62087	1.75985	-2.90476
C	-7.90789	-2.80703	-0.53186
H	-7.74287	-3.03213	0.53607
H	-8.94514	-3.05270	-0.79028
H	-7.22894	-3.43447	-1.13508
C	-2.60558	0.01844	2.53440
C	-0.20246	-1.33652	3.07342
H	0.72714	-1.87530	3.25941
C	-1.81319	0.38697	3.59927
H	-2.11818	1.21905	4.23662
C	-0.60619	-0.28996	3.87183
H	-3.54011	0.54448	2.33899
H	0.01142	0.03266	4.71237
C	4.85573	5.30223	-0.59224
H	4.93395	5.96886	0.27884
H	5.76571	5.43523	-1.19907
H	4.00126	5.64843	-1.19346
C	-0.61569	-0.83740	-3.61666
H	0.27614	-1.42546	-3.88181
H	-1.50326	-1.47672	-3.67673
H	-0.68998	0.00347	-4.31830
H	1.02054	-5.67170	2.61631
H	1.88034	-6.45659	1.26682

PBE0+D3(BJ)/Def2-SVP Gibbs free energy:1.753776 a.u.

PBE0+D3(BJ)/Def2-SVP enthalpy:1.753633 a.u.

PBE0+D3(BJ)/Def2-SVP SCF energy:0.950851 a.u.

PBE0+D3(BJ)/Def2-TZVPP Gibbs free energy:1.753776 a.u.

PBE0+D3(BJ)/Def2-TZVPP enthalpy:1.753633 a.u.

PBE0+D3(BJ)/Def2-TZVPP SCF energy:0.950851 a.u.

Imaginary frequency: -125.9521 cm-1

(*R*, *R*)-4a-9

Cartesian coordinates

ATOM	X	Y	Z
C	2.44128	1.58388	-1.08900
C	4.00742	2.65599	-2.69792
C	2.61537	2.76129	-2.06955
H	2.72262	1.92477	-0.08513
H	1.85079	2.72157	-2.86131
H	2.47182	3.71296	-1.53881
C	3.48207	0.22498	-2.85208

H	4.13985	-0.64470	-2.98548
H	2.47527	-0.06728	-3.16566
C	3.99869	1.44840	-3.64054
H	5.01243	1.26834	-4.03100
H	3.35028	1.64642	-4.50724
H	4.24945	3.57509	-3.25255
C	5.06396	2.40913	-1.60616
H	6.04403	2.34341	-2.10879
C	4.74990	1.02626	-0.97380
H	5.49539	0.27168	-1.25278
H	4.74236	1.08014	0.12621
N	3.42697	0.51171	-1.39931
C	5.15847	3.54076	-0.57667
H	5.19899	4.50361	-1.11359
H	4.24028	3.57393	0.03505
C	0.98525	1.13175	-0.93391
H	0.92009	0.32856	-0.18843
O	0.51797	0.62448	-2.17640
C	-0.44768	-0.31335	-2.11436
O	-0.99641	-0.61108	-1.08965
C	0.12823	2.28877	-0.45895
C	0.22976	2.76860	0.88243
C	-0.72459	2.94157	-1.31794
C	1.09300	2.19111	1.85540
C	-0.57697	3.88758	1.23722
C	-1.46945	4.05093	-0.85745
H	-0.82361	2.61392	-2.35366
C	1.16317	2.72645	3.12313
H	1.66209	1.29484	1.61322
C	-0.49282	4.39774	2.56468
H	-2.13324	4.57776	-1.55308
C	0.35675	3.84283	3.47949
H	-1.12193	5.25380	2.81346
H	0.43801	4.22991	4.49669
N	-1.41330	4.50833	0.36701
O	1.93766	2.24075	4.11147
C	2.83053	1.19490	3.81238
H	3.53812	1.48737	3.01782
H	3.38502	0.98367	4.73411
H	2.30508	0.28508	3.48168
Os	3.11461	-1.71493	-0.20264
O	2.80366	-0.62101	1.07910
O	2.88833	-3.26004	0.53877
O	1.82050	-1.90343	-1.35101

O	4.66177	-1.89473	-0.89377
C	0.80928	-3.70896	-1.31623
C	1.26290	-4.41984	-0.22764
H	1.20475	-3.93731	-2.30631
H	-0.13781	-3.17057	-1.26161
H	2.00498	-5.20507	-0.38853
C	0.50655	-4.49328	1.07971
H	-0.44854	-5.00530	0.87718
C	1.26736	-5.21645	2.17573
H	0.65448	-5.26977	3.08582
O	0.20133	-3.18487	1.56755
C	-0.98591	-2.62617	1.30982
C	-1.26211	-1.40288	2.00281
C	-2.46833	-0.76389	1.65730
C	-3.28319	-1.42152	0.66106
C	-4.53776	-0.80888	0.14758
C	-5.71204	-1.55544	0.11369
C	-4.52354	0.50393	-0.34586
C	-6.88615	-0.98857	-0.38590
H	-5.73033	-2.58251	0.47809
C	-5.69453	1.07404	-0.84778
H	-3.58483	1.05858	-0.34827
C	-6.88534	0.32197	-0.87616
N	-1.81950	-3.19478	0.49359
N	-2.97851	-2.59254	0.17047
O	-8.03733	-1.71117	-0.35218
O	-8.02536	0.86784	-1.37881
O	-5.79028	2.33408	-1.32139
C	-8.56372	-2.09767	-1.60420
H	-7.83978	-2.71860	-2.16026
H	-9.46105	-2.69618	-1.40047
H	-8.83830	-1.22386	-2.21486
C	-8.76800	1.62827	-0.45146
H	-8.18639	2.49286	-0.09028
H	-9.66164	1.99364	-0.97401
H	-9.07933	1.01113	0.40841
C	-4.64193	3.13538	-1.31831
H	-3.84310	2.70191	-1.94525
H	-4.93348	4.10771	-1.73459
H	-4.24225	3.28888	-0.30067
C	-2.82383	0.42054	2.34558
C	-0.42531	-0.88192	3.01054
H	0.49966	-1.40725	3.25072
C	-2.00018	0.92220	3.33112

H	-2.28087	1.83641	3.85820
C	-0.79917	0.26677	3.67210
H	-3.76177	0.92263	2.10745
H	-0.16326	0.68018	4.45733
C	6.37014	3.42510	0.34244
H	6.40442	4.25521	1.06290
H	6.35549	2.48837	0.92163
H	7.30827	3.44139	-0.23430
C	-0.70412	-0.93087	-3.45534
H	-1.64793	-1.48645	-3.42658
H	-0.72107	-0.16895	-4.24560
H	0.12293	-1.62447	-3.67004
H	1.50241	-6.24235	1.85695
H	2.20604	-4.69447	2.40315

PBE0+D3(BJ)/Def2-SVP Gibbs free energy:1.752444 a.u.

PBE0+D3(BJ)/Def2-SVP enthalpy:1.753755 a.u.

PBE0+D3(BJ)/Def2-SVP SCF energy:0.950532 a.u.

PBE0+D3(BJ)/Def2-TZVPP Gibbs free energy:1.752444 a.u.

PBE0+D3(BJ)/Def2-TZVPP enthalpy:1.753755 a.u.

PBE0+D3(BJ)/Def2-TZVPP SCF energy:0.950532 a.u.

Imaginary frequency: -128.4670 cm-1

(*S*, *R*)-1-5

Cartesian coordinates

ATOM	X	Y	Z
C	0.61658	0.80332	2.73446
O	-0.02901	1.54002	2.03890
O	0.79508	-0.50261	2.47906
C	0.41175	-1.00235	1.20230
H	0.49948	-0.18921	0.46790
C	1.33004	-2.18597	0.83853
C	1.15349	-3.38834	1.79262
H	0.99404	-2.47427	-0.16561
C	3.49582	-1.87466	1.96260
C	3.33321	-3.00590	-0.15574
C	2.47823	-4.14781	1.87321
H	0.87045	-3.04235	2.79884
H	0.33338	-4.02837	1.43941
H	4.52298	-1.54250	1.75969
H	3.01514	-1.11437	2.58571
C	3.47425	-3.26567	2.63218
C	3.02651	-4.39611	0.45890
H	4.41187	-2.83074	-0.24602
H	2.90299	-2.90923	-1.16431

H	2.33862	-5.10624	2.39582
H	4.47410	-3.72691	2.61742
H	3.17560	-3.17701	3.68761
H	3.97866	-4.94368	0.56578
N	2.78123	-1.89758	0.66463
C	2.10426	-5.25409	-0.41419
H	1.18192	-4.69646	-0.65106
H	1.78536	-6.13503	0.16843
C	2.75840	-5.71674	-1.71187
H	3.65564	-6.32293	-1.50986
H	2.06727	-6.32774	-2.31041
H	3.06841	-4.86518	-2.33765
C	-1.00562	-1.53967	1.20811
C	-1.62136	-1.92372	-0.02109
C	-1.66356	-1.82709	2.38036
C	-1.10166	-1.56456	-1.29398
C	-2.80723	-2.70473	0.06227
C	-2.88058	-2.54646	2.34002
H	-1.23044	-1.54634	3.34169
C	-1.74296	-1.97800	-2.44035
H	-0.25466	-0.88428	-1.35522
C	-3.38476	-3.19141	-1.14483
H	-3.40518	-2.77317	3.27543
C	-2.86890	-2.84422	-2.36027
H	-4.26260	-3.83215	-1.06502
H	-3.32047	-3.19164	-3.29117
N	-3.41400	-3.00735	1.23792
O	-1.40264	-1.58828	-3.68339
Os	3.33859	0.18786	-0.57506
O	2.06853	-0.42453	-1.54019
O	4.86402	-0.56863	-0.47828
O	3.76585	1.65688	-1.39062
O	2.86568	0.97869	0.90905
C	-1.97534	6.07899	1.22514
C	-0.82868	5.59394	0.63509
C	-0.81950	4.28219	0.11597
C	-1.95515	3.45913	0.20601
C	-3.12953	3.99010	0.79148
C	-3.13209	5.27483	1.29182
H	-1.99571	7.09264	1.63086
H	0.07242	6.20205	0.55507
C	0.31346	3.70921	-0.55215
C	-1.84158	2.13163	-0.35857
H	-4.03204	3.38071	0.83570

H	-4.04347	5.67743	1.73913
C	-3.00252	1.20539	-0.27956
C	-3.53727	0.89051	0.97155
C	-3.59119	0.71972	-1.44494
C	-4.70751	0.13195	1.05513
H	-3.02387	1.23989	1.86636
C	-4.75905	-0.04706	-1.36345
H	-3.13276	0.95311	-2.40472
C	-5.35586	-0.29639	-0.11550
O	-5.40362	-0.55687	-2.43247
O	-5.30179	-0.21556	2.21544
C	-4.77878	0.28576	3.41142
H	-4.78645	1.39022	3.43149
H	-5.42163	-0.08901	4.21761
H	-3.74540	-0.06135	3.58825
C	-4.90318	-0.28394	-3.71458
H	-5.56296	-0.79994	-4.42283
H	-4.92236	0.79804	-3.93385
H	-3.87345	-0.65728	-3.84587
N	-0.76174	1.72254	-0.96628
N	0.32914	2.51366	-1.05689
O	1.37753	4.51077	-0.64523
C	2.56425	4.05861	-1.29250
H	2.28376	3.32081	-2.05800
C	3.49702	3.40358	-0.29651
C	3.10827	3.00344	0.96453
H	4.56045	3.56754	-0.47786
H	3.86385	2.87727	1.73975
H	2.07784	3.12568	1.30049
O	-6.56190	-0.90304	-0.02351
C	-6.57155	-2.31151	-0.11874
H	-7.58557	-2.63996	0.14495
H	-6.34675	-2.63695	-1.14839
H	-5.84521	-2.76344	0.57638
C	3.20282	5.27485	-1.94055
H	2.50702	5.73297	-2.65648
H	3.47389	6.02560	-1.18297
H	4.11205	4.97501	-2.48068
C	1.35759	1.20937	3.97578
H	0.98226	2.17372	4.33559
H	1.27572	0.44213	4.75653
H	2.42067	1.30931	3.70816
C	-0.34308	-0.66375	-3.81992
H	-0.28455	-0.41566	-4.88636

H	-0.52636	0.24816	-3.22773
H	0.61415	-1.09803	-3.49017

PBE0+D3(BJ)/Def2-SVP Gibbs free energy:-2802.215344 a.u.

PBE0+D3(BJ)/Def2-SVP enthalpy:-2802.072047 a.u.

PBE0+D3(BJ)/Def2-SVP SCF energy:-2803.023408 a.u.

PBE0+D3(BJ)/Def2-TZVPP Gibbs free energy:-2803.066753 a.u.

PBE0+D3(BJ)/Def2-TZVPP enthalpy:-2802.923456 a.u.

PBE0+D3(BJ)/Def2-TZVPP SCF energy:-2803.874817 a.u.

Imaginary frequency: -421.7833 cm-1

(*S*, *R*)-1-6

Cartesian coordinates

ATOM	X	Y	Z
C	1.62105	1.01815	2.76159
O	0.81334	1.71023	2.20324
O	1.73964	-0.30340	2.55138
C	0.99145	-0.86819	1.48203
H	0.85465	-0.09883	0.71020
C	1.75521	-2.07557	0.91327
C	1.88928	-3.23518	1.92244
H	1.12813	-2.40866	0.07716
C	4.16028	-1.73947	1.29654
C	3.35309	-2.93259	-0.62952
C	3.17263	-4.00572	1.60311
H	1.94089	-2.84759	2.95215
H	0.99893	-3.87728	1.87232
H	5.07191	-1.41548	0.77588
H	3.89208	-0.95921	2.01537
C	4.35671	-3.10994	1.98029
C	3.25022	-4.30100	0.09515
H	4.35125	-2.76796	-1.05397
H	2.63374	-2.86309	-1.46009
H	3.20644	-4.94724	2.17188
H	5.30072	-3.57931	1.66210
H	4.40946	-2.98818	3.07271
H	4.18702	-4.85660	-0.08236
N	3.07841	-1.79946	0.28662
C	2.10037	-5.17495	-0.41797
H	1.15011	-4.61572	-0.37205
H	1.97952	-6.03433	0.26324
C	2.31430	-5.68456	-1.83942
H	2.41192	-4.85665	-2.55907
H	3.22978	-6.29308	-1.90923
H	1.47042	-6.30725	-2.17014

C	-0.36756	-1.34243	1.95592
C	-1.35152	-1.76329	1.01088
C	-0.64758	-1.48059	3.29523
C	-1.20772	-1.58795	-0.39200
C	-2.52432	-2.38334	1.52757
C	-1.87346	-2.05727	3.69610
H	0.07766	-1.16984	4.04796
C	-2.18789	-2.04070	-1.24973
H	-0.35905	-1.03135	-0.78490
C	-3.49339	-2.87694	0.60968
H	-2.09856	-2.15580	4.76454
C	-3.32745	-2.72088	-0.73695
H	-4.38073	-3.35830	1.02385
H	-4.07276	-3.07563	-1.44963
N	-2.76921	-2.51382	2.85696
O	-2.16228	-1.87756	-2.58241
Os	3.20071	0.27790	-1.10990
O	1.68792	-0.34819	-1.59668
O	4.67266	-0.47423	-1.52750
O	3.30764	1.76713	-1.99816
O	3.25660	1.06316	0.45319
C	-1.83555	5.25207	2.41880
C	-0.79318	4.90705	1.58763
C	-0.92709	3.79644	0.72959
C	-2.10413	3.02281	0.71960
C	-3.17598	3.42535	1.55344
C	-3.03632	4.51378	2.38779
H	-1.74233	6.10880	3.08945
H	0.13607	5.47651	1.57154
C	0.08359	3.41766	-0.21457
C	-2.13494	1.90291	-0.19899
H	-4.11897	2.88145	1.52143
H	-3.87111	4.81417	3.02470
C	-3.35907	1.07197	-0.37848
C	-4.06653	0.54952	0.71037
C	-3.81485	0.85954	-1.68061
C	-5.26896	-0.13179	0.49361
H	-3.65727	0.64058	1.71582
C	-5.00299	0.15649	-1.89935
H	-3.22535	1.24876	-2.50864
C	-5.76447	-0.29136	-0.81053
O	-5.50854	-0.12249	-3.11771
O	-6.02127	-0.68327	1.46328
C	-5.63651	-0.52540	2.80484

H	-6.43916	-0.96519	3.40957
H	-4.69650	-1.05756	3.02359
H	-5.53453	0.54072	3.07427
C	-4.71546	0.13505	-4.24540
H	-5.27275	-0.24090	-5.11222
H	-4.53432	1.21504	-4.38398
H	-3.74869	-0.39058	-4.17790
N	-1.12753	1.63317	-0.98896
N	-0.01525	2.39253	-1.00175
O	1.14869	4.22312	-0.27379
C	2.08478	4.11599	-1.34529
H	1.65129	3.47740	-2.12860
C	3.36754	3.48669	-0.85876
C	3.53063	3.06439	0.44652
H	4.25269	3.72033	-1.45149
H	4.53550	2.94637	0.85210
H	2.71066	3.18445	1.15484
O	-6.96607	-0.88879	-1.01081
C	-6.91729	-2.27374	-1.25144
H	-6.35527	-2.50059	-2.17387
H	-6.46252	-2.81293	-0.40270
H	-7.95241	-2.61799	-1.37481
C	2.31887	5.52283	-1.87645
H	1.37244	5.95586	-2.22847
H	2.73299	6.17289	-1.09120
H	3.02382	5.49709	-2.71981
C	2.64630	1.49654	3.74803
H	3.61259	1.55276	3.22407
H	2.37266	2.49153	4.11599
H	2.75421	0.78985	4.58114
C	-1.07804	-1.16355	-3.14135
H	-0.99291	-0.15779	-2.69811
H	-0.12652	-1.69874	-2.98639
H	-1.27800	-1.09077	-4.21764

PBE0+D3(BJ)/Def2-SVP Gibbs free energy:-2803.020655 a.u.

PBE0+D3(BJ)/Def2-SVP enthalpy:0.000000 a.u.

PBE0+D3(BJ)/Def2-SVP SCF energy:0.000000 a.u.

PBE0+D3(BJ)/Def2-TZVPP Gibbs free energy:-2803.874233 a.u.

PBE0+D3(BJ)/Def2-TZVPP enthalpy:0.000000 a.u.

PBE0+D3(BJ)/Def2-TZVPP SCF energy:0.000000 a.u.

Imaginary frequency: 0.0000 cm-1

(*S*, *R*)-1-7

Cartesian coordinates

ATOM	X	Y	Z
C	-0.71813	0.70274	-2.72161
O	-0.09165	1.48735	-2.06240
O	-0.84418	-0.59910	-2.41688
C	-0.42164	-1.03786	-1.13033
H	-0.52481	-0.20206	-0.42391
C	-1.29048	-2.23870	-0.70712
C	-1.08431	-3.46978	-1.61805
H	-0.92938	-2.47653	0.30117
C	-3.48181	-2.04815	-1.80650
C	-3.25039	-3.09035	0.34751
C	-2.38025	-4.27994	-1.64640
H	-0.82883	-3.15323	-2.64122
H	-0.23551	-4.06533	-1.25340
H	-4.51607	-1.73930	-1.60240
H	-3.03384	-1.30111	-2.46859
C	-3.42510	-3.46506	-2.41598
C	-2.89048	-4.49096	-0.21087
H	-4.33526	-2.95608	0.43593
H	-2.82020	-2.94201	1.34994
H	-2.21784	-5.24683	-2.14406
H	-4.40670	-3.96082	-2.35695
H	-3.15488	-3.41186	-3.48142
H	-3.81671	-5.08768	-0.27475
N	-2.74821	-1.99487	-0.52020
C	-1.92341	-5.24809	0.70776
H	-2.40731	-5.34945	1.69394
H	-1.01873	-4.64235	0.88814
C	-1.52085	-6.62690	0.19873
H	-0.91802	-6.56361	-0.72007
H	-0.91975	-7.16378	0.94686
H	-2.40639	-7.24325	-0.02531
C	1.01545	-1.52021	-1.14513
C	1.66902	-1.84220	0.08228
C	1.66379	-1.81086	-2.32214
C	1.14950	-1.48137	1.35432
C	2.89612	-2.55748	-0.00055
C	2.91275	-2.47173	-2.28503
H	1.20048	-1.57778	-3.28197
C	1.82732	-1.83499	2.49971
H	0.26741	-0.84699	1.41369
C	3.53203	-2.96280	1.20735
H	3.42696	-2.70778	-3.22418

C	3.00905	-2.62371	2.42211
H	4.46230	-3.52692	1.13059
H	3.50376	-2.90618	3.35269
N	3.49207	-2.86601	-1.18014
O	1.47317	-1.44447	3.73896
Os	-3.35800	0.12149	0.64471
O	-2.04823	-0.40426	1.60780
O	-4.85653	-0.69256	0.61490
O	-3.81935	1.61151	1.40192
O	-2.94613	0.85946	-0.88458
C	1.73970	6.06698	-1.53786
C	0.61331	5.57327	-0.91675
C	0.65718	4.29780	-0.31555
C	1.82587	3.51793	-0.35477
C	2.97884	4.06032	-0.97143
C	2.92930	5.30954	-1.55285
H	1.71867	7.05282	-2.00714
H	-0.31199	6.14788	-0.87375
C	-0.45035	3.72523	0.39414
C	1.76634	2.22488	0.29218
H	3.90677	3.48856	-0.97325
H	3.82465	5.72134	-2.02360
C	2.96158	1.34165	0.28642
C	3.54979	0.97771	-0.92513
C	3.52660	0.92731	1.49093
C	4.73348	0.23484	-0.93630
H	3.07352	1.28310	-1.85564
C	4.69166	0.15688	1.48635
H	3.03605	1.20045	2.42377
C	5.32088	-0.18340	0.27069
O	5.29720	-0.29657	2.60014
O	5.38861	-0.10668	-2.07236
C	4.88530	0.33221	-3.30040
H	4.84839	1.43466	-3.35839
H	5.56956	-0.04170	-4.07225
H	3.87398	-0.06495	-3.49699
C	4.76484	0.05733	3.84766
H	4.77029	1.15180	3.99652
H	3.73566	-0.31726	3.98160
H	5.41186	-0.40446	4.60384
N	0.70132	1.81053	0.92405
N	-0.41907	2.56298	0.97020
O	-1.54240	4.49282	0.44890
C	-2.70032	4.04915	1.15153

H	-2.38178	3.36493	1.95121
C	-3.63769	3.31331	0.21899
C	-3.26830	2.86539	-1.03175
H	-4.70069	3.44957	0.42392
H	-4.03857	2.67404	-1.77868
H	-2.25223	3.01118	-1.40047
O	6.48413	-0.85770	0.35082
C	6.73502	-1.96308	-0.49596
H	5.80532	-2.47745	-0.78261
H	7.26458	-1.65145	-1.40856
H	7.37491	-2.65200	0.07411
C	-3.35664	5.28449	1.74388
H	-4.24459	4.99378	2.32305
H	-2.65694	5.80155	2.41445
H	-3.66560	5.98230	0.95103
C	-1.48993	1.03496	-3.96617
H	-1.15963	2.00132	-4.36285
H	-1.38425	0.24578	-4.72185
H	-2.55325	1.09887	-3.68879
C	0.36817	-0.57399	3.86748
H	0.49896	0.33494	3.25669
H	-0.56791	-1.06359	3.55486
H	0.30506	-0.30878	4.92953

PBE0+D3(BJ)/Def2-SVP Gibbs free energy:-2802.215300 a.u.

PBE0+D3(BJ)/Def2-SVP enthalpy:-2802.071992 a.u.

PBE0+D3(BJ)/Def2-SVP SCF energy:-2803.023359 a.u.

PBE0+D3(BJ)/Def2-TZVPP Gibbs free energy:-2803.125373 a.u.

PBE0+D3(BJ)/Def2-TZVPP enthalpy:-2802.982065 a.u.

PBE0+D3(BJ)/Def2-TZVPP SCF energy:-2803.933432 a.u.

Imaginary frequency: -424.0951 cm-1

(*S*, *S*)-1-9

Cartesian coordinates

ATOM	X	Y	Z
C	-2.10423	1.60911	-1.78431
O	-2.67136	1.54898	-0.72916
O	-0.81833	1.98049	-1.90716
C	-0.06607	2.15828	-0.69942
H	-0.14927	1.22312	-0.14219
C	1.41274	2.39382	-1.07167
C	1.58957	3.27268	-2.34200
H	1.85634	2.89413	-0.19936
C	1.68992	0.31429	-2.33493
C	3.60766	1.52187	-1.51509

C	2.34488	2.47140	-3.40940
H	0.61275	3.56220	-2.74674
H	2.12540	4.20002	-2.09610
H	2.36963	-0.54420	-2.43161
H	0.71271	-0.09175	-2.04487
C	1.61423	1.14095	-3.63726
C	3.76278	2.15734	-2.91450
H	4.22836	0.62284	-1.41460
H	3.91714	2.22160	-0.72403
H	2.38832	3.04724	-4.34655
H	2.08000	0.59112	-4.46908
H	0.56706	1.33309	-3.90906
H	4.18993	1.40299	-3.59920
N	2.20457	1.13721	-1.22202
C	4.71501	3.35340	-2.91440
H	4.33568	4.12978	-2.22778
H	4.71340	3.80646	-3.92014
C	6.14412	2.98834	-2.52417
H	6.19637	2.58261	-1.50186
H	6.55857	2.22780	-3.20486
H	6.80448	3.86686	-2.56192
C	-0.60694	3.29706	0.14439
C	-0.87393	3.12482	1.53847
C	-0.85163	4.52999	-0.41730
C	-0.71705	1.89040	2.23495
C	-1.38652	4.25607	2.23894
C	-1.33827	5.58662	0.38362
H	-0.69066	4.70169	-1.48240
C	-1.07707	1.78531	3.56256
H	-0.36981	1.00633	1.70776
C	-1.72575	4.11552	3.61634
H	-1.52051	6.56795	-0.06989
C	-1.58207	2.92069	4.25938
H	-2.11439	4.99800	4.12680
H	-1.85047	2.79610	5.30978
N	-1.59605	5.46461	1.66011
O	-1.02376	0.65853	4.28891
Os	2.38089	-0.26644	0.84198
O	0.74587	-0.55790	0.43652
O	2.94711	1.23610	1.41342
O	2.57203	-1.28718	2.23303
O	3.52772	-1.15547	-0.12817
C	1.08405	-3.11741	-3.21013
C	1.43283	-3.50354	-1.93425

C	0.51850	-3.31897	-0.87575
C	-0.75214	-2.75651	-1.10973
C	-1.07664	-2.34595	-2.42534
C	-0.17359	-2.52657	-3.45188
H	1.78665	-3.25391	-4.03484
H	2.40823	-3.94032	-1.72110
C	0.83692	-3.59413	0.49645
C	-1.60664	-2.57827	0.04342
H	-2.04230	-1.87634	-2.61415
H	-0.43102	-2.20164	-4.46257
C	-2.98204	-2.02679	-0.08128
C	-3.88351	-2.57164	-1.00449
C	-3.35892	-0.96399	0.73531
C	-5.16299	-2.02176	-1.12906
H	-3.58460	-3.42938	-1.60591
C	-4.63801	-0.41151	0.61425
H	-2.63393	-0.54468	1.42902
C	-5.54652	-0.94309	-0.31566
O	-5.08660	0.63145	1.32912
O	-6.09650	-2.45537	-2.00340
C	-5.79613	-3.54905	-2.82117
H	-4.93764	-3.34510	-3.48692
H	-5.57625	-4.45642	-2.23164
H	-6.68419	-3.73518	-3.43772
C	-4.19554	1.30145	2.18947
H	-4.76300	2.11920	2.64906
H	-3.82008	0.63813	2.98784
H	-3.34368	1.71488	1.63023
N	-1.19997	-2.85709	1.25407
N	0.02904	-3.35360	1.48112
O	2.06120	-4.08931	0.72729
C	2.63910	-3.98299	2.03026
H	1.87042	-3.61515	2.72339
C	3.76921	-2.98533	1.96835
C	4.46713	-2.73781	0.80656
H	4.21100	-2.71442	2.92893
H	5.42560	-2.22004	0.84460
H	4.24466	-3.30893	-0.09321
O	-6.78860	-0.41308	-0.44148
C	-6.84091	0.74846	-1.23580
H	-7.87318	1.12092	-1.20255
H	-6.16311	1.52875	-0.85132
H	-6.57873	0.52246	-2.28547
C	3.10612	-5.36499	2.46027

H	2.24497	-6.04400	2.53006
H	3.82571	-5.77571	1.73668
H	3.59079	-5.31546	3.44596
C	-2.71358	1.29139	-3.12067
H	-2.73624	2.19546	-3.74614
H	-2.10016	0.54201	-3.64059
H	-3.73077	0.91254	-2.97111
C	-0.45071	-0.50352	3.73229
H	-0.51792	-1.28226	4.49986
H	-0.98729	-0.85740	2.83766
H	0.60972	-0.34274	3.48164

PBE0+D3(BJ)/Def2-SVP Gibbs free energy:-2802.207400 a.u.

PBE0+D3(BJ)/Def2-SVP enthalpy:-2802.059012 a.u.

PBE0+D3(BJ)/Def2-SVP SCF energy:-2803.009656 a.u.

PBE0+D3(BJ)/Def2-TZVPP Gibbs free energy:-2803.061954 a.u.

PBE0+D3(BJ)/Def2-TZVPP enthalpy:-2802.913566 a.u.

PBE0+D3(BJ)/Def2-TZVPP SCF energy:-2803.864210 a.u.

Imaginary frequency: -417.2922 cm-1

(*S*, *R*)-1a-10

Cartesian coordinates

ATOM	X	Y	Z
C	2.06167	-3.85099	-2.27753
O	3.15746	-3.46371	-1.97431
O	0.92223	-3.28226	-1.84383
C	1.02697	-2.17322	-0.94269
H	2.00382	-1.71883	-1.11845
C	-0.06460	-1.12512	-1.26297
C	-1.34755	-1.69592	-1.92388
H	-0.32488	-0.65928	-0.30455
C	0.89189	-0.49208	-3.41800
C	-0.56620	1.01110	-2.24278
C	-1.55113	-1.01409	-3.27973
H	-1.26187	-2.78072	-2.06851
H	-2.21601	-1.51371	-1.27620
H	1.22278	0.39013	-3.98458
H	1.77908	-1.13007	-3.31263
C	-0.28223	-1.21320	-4.11758
C	-1.77171	0.49282	-3.06781
H	-0.11009	1.89679	-2.70210
H	-0.88744	1.31313	-1.23630
H	-2.41235	-1.46098	-3.79672
H	-0.42964	-0.81548	-5.13284
H	-0.07140	-2.28884	-4.21547

H	-1.75173	0.96869	-4.06456
N	0.48985	-0.01257	-2.08275
C	-3.09602	0.86771	-2.39719
H	-3.09266	1.95484	-2.21406
H	-3.13495	0.42005	-1.39326
C	-4.35044	0.47572	-3.16694
H	-4.44901	-0.61659	-3.26440
H	-5.25189	0.82981	-2.64475
H	-4.35484	0.90441	-4.18249
C	0.94621	-2.66981	0.48836
C	2.03685	-2.55639	1.40166
C	-0.22323	-3.23615	0.94588
C	3.32090	-2.05721	1.04531
C	1.79878	-2.96659	2.74735
C	-0.33398	-3.64389	2.29128
H	-1.07116	-3.36212	0.27320
C	4.30947	-1.92949	1.99503
H	3.53189	-1.82178	0.00467
C	2.84334	-2.81330	3.70602
H	-1.26766	-4.09509	2.64809
C	4.05665	-2.30168	3.34789
H	2.63402	-3.12690	4.73014
H	4.86656	-2.18243	4.06970
N	0.62711	-3.50097	3.16945
O	5.55046	-1.47669	1.75158
Os	2.45114	1.11650	-0.83467
O	3.32073	0.22666	-2.00388
O	1.55285	0.38310	0.40860
O	3.72044	1.88953	0.06566
O	1.76367	2.60795	-1.41651
C	0.99981	0.40337	3.84721
C	1.05260	1.51550	3.04007
C	-0.05351	1.83810	2.22648
C	-1.21472	1.03381	2.22625
C	-1.25667	-0.07976	3.10103
C	-0.16670	-0.38883	3.88541
H	1.86207	0.12415	4.45552
H	1.94590	2.13756	2.98785
C	-0.05678	2.95357	1.32649
C	-2.27363	1.43180	1.32706
H	-2.15819	-0.68819	3.16230
H	-0.19896	-1.26879	4.53032
C	-3.54235	0.67453	1.11582
C	-3.58124	-0.72238	1.00778

C	-4.69491	1.42668	0.87298
C	-4.78381	-1.35625	0.66285
H	-2.67145	-1.30672	1.14327
C	-5.88463	0.79784	0.50723
H	-4.61885	2.51031	0.92611
C	-5.93195	-0.59972	0.38383
O	-7.03430	1.44447	0.23272
O	-4.94711	-2.69066	0.55118
C	-3.84272	-3.53202	0.70516
H	-3.09547	-3.35998	-0.08776
H	-3.35717	-3.40670	1.68891
H	-4.21389	-4.56062	0.62169
C	-7.04347	2.84694	0.26332
H	-6.81059	3.23687	1.26918
H	-6.32647	3.27413	-0.45834
H	-8.05784	3.15854	-0.01349
N	-2.18506	2.52052	0.60163
N	-1.08501	3.28617	0.60464
O	1.07727	3.65742	1.23358
C	1.21535	4.62274	0.18339
H	0.38185	4.48719	-0.51668
C	2.51000	4.34305	-0.53785
C	3.65534	3.94681	0.11729
H	2.59034	4.77449	-1.53783
H	4.62485	4.02754	-0.37366
H	3.65717	3.85047	1.20280
O	-7.06630	-1.21712	-0.02325
C	-7.99783	-1.48478	0.99963
H	-8.85577	-1.98564	0.53283
H	-7.56765	-2.15221	1.76610
H	-8.34434	-0.55402	1.47967
C	1.15143	6.01960	0.78132
H	1.93134	6.15316	1.54547
H	1.29606	6.77793	-0.00199
H	0.16636	6.18053	1.24182
C	1.76460	-5.00659	-3.19120
H	1.21174	-4.64984	-4.07210
H	2.70242	-5.47722	-3.50439
H	1.12639	-5.73590	-2.67346
C	5.86554	-1.03454	0.45286
H	5.26093	-0.15632	0.17372
H	6.92355	-0.74783	0.47061
H	5.71893	-1.82973	-0.29708

PBE0+D3(BJ)/Def2-SVP Gibbs free energy:-2802.208313 a.u.

PBE0+D3(BJ)/Def2-SVP enthalpy:-2802.059287 a.u.
PBE0+D3(BJ)/Def2-SVP SCF energy:-2803.010499 a.u.
PBE0+D3(BJ)/Def2-TZVPP Gibbs free energy:-2803.062298 a.u.
PBE0+D3(BJ)/Def2-TZVPP enthalpy:-2802.913272 a.u.
PBE0+D3(BJ)/Def2-TZVPP SCF energy:-2803.864484 a.u.
Imaginary frequency: -428.2191 cm-1

(*S*, *R*)-1a-11

Cartesian coordinates

ATOM	X	Y	Z
C	-1.07089	-0.79269	2.99604
O	-2.01869	-0.06324	3.12132
O	-0.95485	-1.68374	1.98991
C	-1.90405	-1.62439	0.92954
H	-2.29862	-0.60493	0.90474
C	-1.19796	-1.93941	-0.40481
C	-0.42140	-3.28526	-0.39126
H	-2.00979	-1.98171	-1.14583
C	0.86123	-0.64904	0.00366
C	0.22914	-1.28846	-2.21665
C	1.06521	-3.02284	-0.66601
H	-0.52467	-3.77977	0.58343
H	-0.84394	-3.97533	-1.13577
H	1.53458	0.02896	-0.53341
H	0.52629	-0.11592	0.89673
C	1.56087	-1.97776	0.33716
C	1.24975	-2.43976	-2.07622
H	0.68491	-0.41457	-2.70085
H	-0.63623	-1.58950	-2.82706
H	1.63546	-3.95908	-0.56337
H	2.65111	-1.85805	0.27006
H	1.32337	-2.29239	1.36158
H	2.26471	-2.00589	-2.11895
N	-0.29410	-0.85600	-0.89726
C	1.14897	-3.46110	-3.20718
H	0.15084	-3.93284	-3.19600
H	1.87173	-4.27171	-3.01223
C	1.41596	-2.86361	-4.58550
H	2.40662	-2.38263	-4.62236
H	1.38608	-3.63465	-5.36921
H	0.66993	-2.09816	-4.84941
C	-3.05013	-2.57909	1.19121
C	-4.35114	-2.33006	0.65522
C	-2.87236	-3.70439	1.96327

C	-4.67947	-1.17273	-0.10809
C	-5.36631	-3.28717	0.94448
C	-3.95221	-4.59130	2.17478
H	-1.90548	-3.90909	2.42513
C	-5.97249	-0.96980	-0.54435
H	-3.90692	-0.44553	-0.34388
C	-6.68333	-3.05837	0.45035
H	-3.79833	-5.49198	2.78058
C	-6.98156	-1.93591	-0.26692
H	-7.44132	-3.80853	0.68090
H	-7.98909	-1.74166	-0.63853
N	-5.15057	-4.40358	1.68433
O	-6.39367	0.10752	-1.22927
Os	-1.37063	1.35903	-1.28217
O	-2.39785	0.31544	-2.16609
O	0.22650	1.71730	-1.76190
O	-2.16517	2.89089	-1.47557
O	-1.60366	1.31185	0.44339
C	2.12751	5.02773	-1.46347
C	1.27359	4.65059	-0.45167
C	1.60543	3.54597	0.35917
C	2.78310	2.80720	0.12753
C	3.66095	3.23676	-0.89573
C	3.33304	4.32611	-1.67347
H	1.87785	5.87770	-2.10143
H	0.34306	5.18539	-0.26336
C	0.78057	3.09628	1.44292
C	2.99702	1.65699	0.97154
H	4.59990	2.70810	-1.06059
H	4.01471	4.65056	-2.46240
C	4.02745	0.62254	0.68220
C	4.19673	0.14904	-0.61700
C	4.68637	-0.00818	1.74548
C	5.01075	-0.96141	-0.87194
H	3.64839	0.57168	-1.45996
C	5.52390	-1.09241	1.49744
H	4.50255	0.35139	2.75618
C	5.69435	-1.57932	0.18132
O	6.22143	-1.74943	2.44491
O	5.07894	-1.38151	-2.15628
C	5.05269	-2.76474	-2.44446
H	6.05327	-3.21657	-2.39950
H	4.39391	-3.30518	-1.74498
H	4.65100	-2.86196	-3.46236

C	6.07628	-1.35764	3.78423
H	6.71405	-2.02500	4.37610
H	6.40169	-0.31507	3.94295
H	5.03261	-1.46101	4.12677
N	2.21702	1.39449	1.98987
N	1.11786	2.12597	2.24141
O	-0.37344	3.74227	1.61170
C	-1.43996	3.11096	2.32938
H	-1.09058	2.14648	2.71521
C	-2.57306	2.86149	1.36511
C	-2.88992	3.71862	0.33335
H	-3.32584	2.15922	1.73325
H	-3.88505	3.70637	-0.10931
H	-2.27452	4.59811	0.14394
O	6.50625	-2.64661	-0.04475
C	7.88463	-2.34376	-0.08169
H	8.41472	-3.28233	-0.28902
H	8.11050	-1.61654	-0.88102
H	8.23057	-1.94264	0.88438
C	-1.84745	4.00258	3.48976
H	-1.00371	4.11686	4.18472
H	-2.15534	4.99700	3.13373
H	-2.68644	3.54750	4.03509
C	0.14557	-0.81708	3.87184
H	0.96402	-0.29685	3.34515
H	-0.06489	-0.28987	4.80855
H	0.46293	-1.84984	4.06815
C	-5.47473	1.13023	-1.52137
H	-5.03070	1.54669	-0.59921
H	-4.65951	0.77444	-2.17016
H	-6.03600	1.91988	-2.03459

PBE0+D3(BJ)/Def2-SVP Gibbs free energy:-2802.213812 a.u.

PBE0+D3(BJ)/Def2-SVP enthalpy:-2802.064962 a.u.

PBE0+D3(BJ)/Def2-SVP SCF energy:-2803.015574 a.u.

PBE0+D3(BJ)/Def2-TZVPP Gibbs free energy:-2803.066706 a.u.

PBE0+D3(BJ)/Def2-TZVPP enthalpy:-2802.917856 a.u.

PBE0+D3(BJ)/Def2-TZVPP SCF energy:-2803.868468 a.u.

Imaginary frequency: -387.9777 cm-1

(*S*, *R*)-1a-14

Cartesian coordinates

ATOM	X	Y	Z
C	-1.03473	-0.78623	2.99557
O	-1.99527	-0.07349	3.11868

O	-0.90028	-1.67420	1.98875
C	-1.84469	-1.62684	0.92352
H	-2.25307	-0.61282	0.89704
C	-1.12688	-1.93082	-0.40705
C	-0.33337	-3.26690	-0.38980
H	-1.93355	-1.98270	-1.15313
C	0.91371	-0.61619	0.01456
C	0.30122	-1.25743	-2.20979
C	1.15077	-2.98569	-0.65920
H	-0.43419	-3.76192	0.58494
H	-0.74426	-3.96270	-1.13549
H	1.58041	0.07147	-0.51777
H	0.56810	-0.09063	0.90779
C	1.63039	-1.93590	0.34692
C	1.33310	-2.39903	-2.06819
H	0.75128	-0.37714	-2.68771
H	-0.55734	-1.56473	-2.82663
H	1.73178	-3.91572	-0.55634
H	2.71882	-1.80001	0.28082
H	1.39548	-2.25553	1.37057
H	2.34388	-1.95512	-2.10838
N	-0.23411	-0.83557	-0.89204
C	1.24376	-3.42002	-3.20056
H	0.25002	-3.90102	-3.19184
H	1.97347	-4.22464	-3.00569
C	1.50770	-2.81825	-4.57757
H	1.48818	-3.58867	-5.36221
H	0.75386	-2.06086	-4.84232
H	2.49283	-2.32619	-4.61092
C	-2.97911	-2.59722	1.17739
C	-4.27895	-2.36677	0.63050
C	-2.79183	-3.71932	1.95181
C	-4.61705	-1.21441	-0.13606
C	-5.28280	-3.33810	0.91174
C	-3.86078	-4.62122	2.15507
H	-1.82587	-3.90974	2.42170
C	-5.90913	-1.03005	-0.58334
H	-3.85297	-0.47617	-0.36536
C	-6.59863	-3.12836	0.40614
H	-3.69944	-5.51915	2.76302
C	-6.90658	-2.01054	-0.31423
H	-7.34788	-3.88909	0.63059
H	-7.91356	-1.83077	-0.69453
N	-5.05744	-4.45085	1.65424

O	-6.33991	0.04097	-1.27188
Os	-1.33224	1.37182	-1.27525
O	-2.33806	0.32036	-2.17455
O	0.26642	1.74956	-1.73456
O	-2.14205	2.89511	-1.47049
O	-1.58266	1.31440	0.44754
C	2.12016	5.15914	-1.37808
C	1.25932	4.74827	-0.38525
C	1.59485	3.62826	0.40260
C	2.78275	2.90776	0.16527
C	3.66720	3.37162	-0.83701
C	3.33584	4.47625	-1.59149
H	1.86842	6.02213	-1.99750
H	0.32199	5.26973	-0.19298
C	0.76627	3.14610	1.46941
C	3.00018	1.73951	0.98317
H	4.61384	2.85714	-1.00335
H	4.02282	4.82773	-2.36403
C	4.02883	0.71125	0.66905
C	4.21004	0.28070	-0.64170
C	4.67321	0.03785	1.71415
C	4.99890	-0.84106	-0.93019
H	3.67789	0.73857	-1.47647
C	5.49631	-1.04864	1.43585
H	4.49417	0.37071	2.73455
C	5.66225	-1.51181	0.10779
O	6.19379	-1.72444	2.37316
O	5.06096	-1.18452	-2.23584
C	5.18694	-2.53091	-2.63305
H	4.51695	-3.18457	-2.04725
H	4.87687	-2.56888	-3.68580
H	6.21868	-2.89498	-2.53944
C	6.09376	-1.32444	3.71434
H	6.74416	-1.99452	4.28921
H	6.43609	-0.28513	3.85422
H	5.06071	-1.41389	4.09180
N	2.21760	1.44981	1.99223
N	1.11048	2.16589	2.25256
O	-0.39848	3.77247	1.64032
C	-1.45947	3.11436	2.34178
H	-1.09479	2.15381	2.72332
C	-2.57899	2.85113	1.36543
C	-2.89756	3.70636	0.33265
H	-3.32548	2.13757	1.72446

H	-3.88787	3.68094	-0.12021
H	-2.29276	4.59490	0.15176
O	6.49402	-2.54928	-0.16945
C	6.14271	-3.81554	0.34824
H	6.82165	-4.54501	-0.11221
H	6.25399	-3.85156	1.44102
H	5.10238	-4.07708	0.08320
C	-1.89548	3.98852	3.50514
H	-1.06065	4.11436	4.20881
H	-2.21998	4.97935	3.15391
H	-2.73008	3.51241	4.03914
C	0.17945	-0.79080	3.87491
H	0.51228	-1.81840	4.07288
H	0.99083	-0.25796	3.34979
H	-0.04189	-0.26648	4.81072
C	-5.43418	1.07838	-1.55358
H	-4.60690	0.73642	-2.19449
H	-6.00263	1.85976	-2.07151
H	-5.00629	1.50038	-0.62633

PBE0+D3(BJ)/Def2-SVP Gibbs free energy:-2802.213024 a.u.
PBE0+D3(BJ)/Def2-SVP enthalpy:-2802.064708 a.u.
PBE0+D3(BJ)/Def2-SVP SCF energy:-2803.015514 a.u.
PBE0+D3(BJ)/Def2-TZVPP Gibbs free energy:-2803.065594 a.u.
PBE0+D3(BJ)/Def2-TZVPP enthalpy:-2802.917278 a.u.
PBE0+D3(BJ)/Def2-TZVPP SCF energy:-2803.868084 a.u.
Imaginary frequency: -388.3684 cm-1

(*S*, *R*)-1a-15

Cartesian coordinates

ATOM	X	Y	Z
C	-2.16067	-4.17189	-1.96370
O	-1.75433	-3.63018	-2.95240
O	-1.69416	-3.89524	-0.72610
C	-0.77395	-2.81489	-0.62658
H	-1.07297	-2.06059	-1.36048
C	-0.83636	-2.22906	0.79352
C	-0.57470	-3.28755	1.89246
H	-0.04047	-1.47017	0.80765
C	-3.21589	-2.34463	1.42730
C	-1.76486	-0.65112	2.33167
C	-1.47350	-2.99496	3.09388
H	-0.80536	-4.29394	1.51569
H	0.48495	-3.28816	2.17627
H	-4.08312	-1.69138	1.59960

H	-3.42839	-2.93964	0.53432
C	-2.92302	-3.22982	2.65878
C	-1.31998	-1.53028	3.52642
H	-2.66504	-0.07393	2.56826
H	-0.98431	0.07211	2.05885
H	-1.21424	-3.66401	3.92864
H	-3.60792	-2.98976	3.48649
H	-3.07414	-4.29147	2.41135
H	-2.01743	-1.36278	4.36535
N	-2.07094	-1.45900	1.12084
C	0.08325	-1.17101	4.01971
H	0.78471	-1.19872	3.17015
H	0.42358	-1.94385	4.73123
C	0.16405	0.20553	4.67026
H	-0.51217	0.27342	5.53778
H	1.18552	0.41379	5.02081
H	-0.09881	0.99931	3.95546
C	0.64647	-3.25784	-0.93111
C	1.59630	-2.34984	-1.49368
C	1.08268	-4.52038	-0.59972
C	1.25029	-1.06628	-1.99206
C	2.95136	-2.78482	-1.57340
C	2.44809	-4.85542	-0.75267
H	0.38656	-5.26064	-0.20320
C	2.20953	-0.24592	-2.54213
H	0.22154	-0.72917	-1.94133
C	3.92780	-1.88259	-2.08541
H	2.78916	-5.85907	-0.47289
C	3.57431	-0.64789	-2.55127
H	4.96208	-2.22710	-2.11215
H	4.31269	0.04073	-2.96553
N	3.35713	-4.02167	-1.19168
O	1.94584	0.94261	-3.11662
Os	-2.77520	0.19766	-0.60554
O	-3.52891	-1.18972	-1.24985
O	-1.08019	0.42137	-0.59261
O	-3.26213	1.44461	-1.71423
O	-3.61440	0.90179	0.75070
C	0.39258	5.04586	-2.38612
C	-0.52559	4.73293	-1.40781
C	-0.18039	3.80613	-0.40200
C	1.09071	3.19722	-0.38086
C	2.00049	3.50871	-1.42000
C	1.65618	4.42149	-2.39363

H	0.13698	5.76463	-3.16737
H	-1.51602	5.18771	-1.39131
C	-1.09424	3.39599	0.62561
C	1.34513	2.26184	0.69633
H	2.95813	2.99459	-1.47156
H	2.36447	4.64447	-3.19429
C	2.66894	1.59588	0.84982
C	2.73912	0.22135	1.08465
C	3.84187	2.35487	0.77159
C	3.98743	-0.39874	1.20650
H	1.81438	-0.34788	1.15303
C	5.09099	1.73466	0.86702
H	3.77392	3.43403	0.64540
C	5.17028	0.35021	1.08677
O	6.26894	2.38324	0.77613
O	4.17305	-1.71363	1.43621
C	3.09257	-2.49481	1.86111
H	3.47567	-3.51254	1.99983
H	2.68431	-2.12301	2.81638
H	2.28640	-2.52715	1.11180
C	6.26383	3.76906	0.58396
H	5.77386	4.29946	1.41936
H	7.31358	4.08311	0.53402
H	5.76227	4.05373	-0.35852
N	0.42715	1.96282	1.57712
N	-0.79717	2.52641	1.53961
O	-2.30269	3.97073	0.58113
C	-3.38299	3.49471	1.38372
H	-2.98525	2.78395	2.12132
C	-4.38197	2.78226	0.50011
C	-4.41977	2.95289	-0.86763
H	-5.27026	2.41233	1.01553
H	-5.30196	2.65172	-1.43214
H	-3.73014	3.64342	-1.34965
O	6.38571	-0.22755	1.22500
C	6.79238	-1.06802	0.16777
H	6.14486	-1.95543	0.08513
H	6.79088	-0.52361	-0.79313
H	7.81823	-1.38762	0.39183
C	-3.99814	4.69388	2.08948
H	-4.85217	4.37826	2.70586
H	-3.25315	5.16651	2.74457
H	-4.34975	5.43694	1.35864
C	-3.25589	-5.19777	-1.90626

H	-3.41075	-5.62682	-2.90219
H	-3.02487	-5.98223	-1.17424
H	-4.18109	-4.69440	-1.58658
C	0.59814	1.29433	-3.33355
H	0.07338	0.51040	-3.90609
H	0.06250	1.46507	-2.38589
H	0.60628	2.22366	-3.91415

PBE0+D3(BJ)/Def2-SVP Gibbs free energy:-2802.211922 a.u.

PBE0+D3(BJ)/Def2-SVP enthalpy:-2802.066917 a.u.

PBE0+D3(BJ)/Def2-SVP SCF energy:-2803.018352 a.u.

PBE0+D3(BJ)/Def2-TZVPP Gibbs free energy:-2803.065305 a.u.

PBE0+D3(BJ)/Def2-TZVPP enthalpy:-2802.920300 a.u.

PBE0+D3(BJ)/Def2-TZVPP SCF energy:-2803.871735 a.u.

Imaginary frequency: -422.1561 cm-1

(*S*, *R*)-1a-16

Cartesian coordinates

ATOM	X	Y	Z
C	-1.03443	-0.78667	2.99554
O	-1.99491	-0.07386	3.11870
O	-0.90005	-1.67458	1.98865
C	-1.84450	-1.62707	0.92346
H	-2.25284	-0.61302	0.89712
C	-1.12679	-1.93094	-0.40719
C	-0.33321	-3.26698	-0.39009
H	-1.93353	-1.98282	-1.15320
C	0.91372	-0.61621	0.01438
C	0.30116	-1.25739	-2.20999
C	1.15090	-2.98567	-0.65953
H	-0.43396	-3.76209	0.58461
H	-0.74409	-3.96273	-1.13582
H	1.58036	0.07153	-0.51792
H	0.56811	-0.09075	0.90766
C	1.63051	-1.93590	0.34662
C	1.33313	-2.39892	-2.06849
H	0.75112	-0.37705	-2.68791
H	-0.55741	-1.56473	-2.82678
H	1.73197	-3.91567	-0.55675
H	2.71893	-1.79994	0.28047
H	1.39569	-2.25560	1.37027
H	2.34388	-1.95492	-2.10871
N	-0.23412	-0.83561	-0.89219
C	1.24383	-3.41985	-3.20092
H	0.25014	-3.90094	-3.19217

H	1.97362	-4.22441	-3.00614
C	1.50762	-2.81798	-4.57792
H	2.49270	-2.32582	-4.61130
H	1.48812	-3.58835	-5.36259
H	0.75368	-2.06065	-4.84257
C	-2.97895	-2.59743	1.17729
C	-4.27882	-2.36684	0.63055
C	-2.79165	-3.71968	1.95151
C	-4.61694	-1.21433	-0.13577
C	-5.28268	-3.33817	0.91172
C	-3.86062	-4.62156	2.15471
H	-1.82566	-3.91021	2.42129
C	-5.90905	-1.02984	-0.58291
H	-3.85285	-0.47607	-0.36497
C	-6.59855	-3.12830	0.40625
H	-3.69926	-5.51960	2.76250
C	-6.90651	-2.01034	-0.31390
H	-7.34781	-3.88903	0.63062
H	-7.91351	-1.83047	-0.69410
N	-5.05732	-4.45106	1.65401
O	-6.33984	0.04132	-1.27123
Os	-1.33238	1.37174	-1.27531
O	-2.33812	0.32028	-2.17471
O	0.26627	1.74960	-1.73456
O	-2.14227	2.89500	-1.47047
O	-1.58286	1.31420	0.44746
C	2.11978	5.15928	-1.37788
C	1.25898	4.74829	-0.38508
C	1.59458	3.62825	0.40271
C	2.78254	2.90786	0.16534
C	3.66695	3.37183	-0.83692
C	3.33550	4.47648	-1.59134
H	1.86798	6.02229	-1.99725
H	0.32161	5.26967	-0.19278
C	0.76602	3.14596	1.46947
C	3.00005	1.73958	0.98317
H	4.61362	2.85742	-1.00329
H	4.02246	4.82806	-2.36387
C	4.02882	0.71144	0.66904
C	4.21009	0.28090	-0.64170
C	4.67327	0.03812	1.71416
C	4.99909	-0.84078	-0.93015
H	3.67789	0.73867	-1.47648
C	5.49652	-1.04827	1.43589

H	4.49418	0.37097	2.73455
C	5.66252	-1.51142	0.10784
O	6.19408	-1.72396	2.37321
O	5.06121	-1.18433	-2.23577
C	5.18683	-2.53082	-2.63279
H	6.21852	-2.89508	-2.53935
H	4.51684	-3.18423	-2.04672
H	4.87651	-2.56893	-3.68546
C	6.09396	-1.32397	3.71440
H	6.43615	-0.28461	3.85429
H	5.06091	-1.41356	4.09182
H	6.74444	-1.99396	4.28928
N	2.21748	1.44975	1.99220
N	1.11029	2.16572	2.25256
O	-0.39876	3.77224	1.64044
C	-1.45973	3.11401	2.34183
H	-1.09501	2.15344	2.72327
C	-2.57924	2.85084	1.36545
C	-2.89781	3.70613	0.33271
H	-3.32572	2.13725	1.72443
H	-3.88811	3.68074	-0.12015
H	-2.29301	4.59469	0.15189
O	6.49443	-2.54877	-0.16943
C	6.14345	-3.81506	0.34841
H	5.10296	-4.07660	0.08401
H	6.82211	-4.54450	-0.11252
H	6.25542	-3.85113	1.44112
C	-1.89578	3.98804	3.50527
H	-1.06096	4.11385	4.20895
H	-2.22032	4.97890	3.15414
H	-2.73036	3.51184	4.03923
C	0.17975	-0.79138	3.87488
H	-0.04166	-0.26735	4.81084
H	0.51268	-1.81900	4.07257
H	0.99107	-0.25830	3.34994
C	-5.43409	1.07872	-1.55285
H	-6.00253	1.86023	-2.07060
H	-5.00608	1.50055	-0.62557
H	-4.60689	0.73682	-2.19390

PBE0+D3(BJ)/Def2-SVP Gibbs free energy:-2802.212989 a.u.

PBE0+D3(BJ)/Def2-SVP enthalpy:-2802.064704 a.u.

PBE0+D3(BJ)/Def2-SVP SCF energy:-2803.015514 a.u.

PBE0+D3(BJ)/Def2-TZVPP Gibbs free energy:-2803.065559 a.u.

PBE0+D3(BJ)/Def2-TZVPP enthalpy:-2802.917274 a.u.

PBE0+D3(BJ)/Def2-TZVPP SCF energy:-2803.868084 a.u.
Imaginary frequency: -388.3634 cm-1

(*S*, *R*)-1a-3

Cartesian coordinates

ATOM	X	Y	Z
C	1.47213	-4.11986	-2.22471
O	2.59843	-3.78122	-1.97912
O	0.38135	-3.47438	-1.77819
C	0.56973	-2.33658	-0.92807
H	1.56955	-1.95404	-1.13961
C	-0.46084	-1.23615	-1.26808
C	-1.77794	-1.75068	-1.90313
H	-0.68730	-0.73772	-0.31979
C	0.47374	-0.65975	-3.44979
C	-0.86582	0.92442	-2.22178
C	-1.98468	-1.04322	-3.24442
H	-1.73198	-2.83429	-2.06544
H	-2.62100	-1.56423	-1.22582
H	0.84241	0.20100	-4.02656
H	1.32182	-1.35138	-3.36330
C	-0.75613	-1.31187	-4.11975
C	-2.11507	0.47195	-3.02230
H	-0.37494	1.79028	-2.68377
H	-1.14092	1.22872	-1.20102
H	-2.89194	-1.42851	-3.73478
H	-0.90687	-0.90351	-5.13042
H	-0.60829	-2.39761	-4.22404
H	-2.08723	0.95350	-4.01623
N	0.13812	-0.15891	-2.10423
C	-3.43581	0.86934	-2.36021
H	-3.50150	0.40588	-1.36232
H	-4.26049	0.43790	-2.95406
C	-3.63680	2.37383	-2.21932
H	-3.53246	2.88606	-3.18974
H	-4.64496	2.58982	-1.83380
H	-2.92041	2.81088	-1.50683
C	0.47983	-2.74981	0.52904
C	1.60509	-2.67556	1.40505
C	-0.72845	-3.15172	1.05590
C	2.91745	-2.32520	0.97916
C	1.37187	-2.95127	2.78421
C	-0.83666	-3.41890	2.43804
H	-1.62653	-3.21631	0.43844

C	3.94363	-2.21887	1.89109
H	3.11527	-2.18925	-0.08183
C	2.45931	-2.83596	3.69947
H	-1.80733	-3.71547	2.85217
C	3.70355	-2.47163	3.27312
H	2.25601	-3.05346	4.74960
H	4.54489	-2.38154	3.96258
N	0.16151	-3.30859	3.27977
O	5.21140	-1.89798	1.58149
Os	2.21366	0.82267	-0.94239
O	2.95167	-0.23463	-2.06146
O	1.24333	0.29780	0.35320
O	3.59948	1.46768	-0.11341
O	1.72568	2.35822	-1.60888
C	1.66322	0.84974	3.81681
C	1.66796	1.93023	2.96384
C	0.49921	2.25034	2.24255
C	-0.67160	1.47382	2.38216
C	-0.66523	0.40642	3.31117
C	0.48539	0.09662	4.00214
H	2.57196	0.56917	4.35251
H	2.56722	2.52545	2.80796
C	0.44395	3.30025	1.26925
C	-1.77469	1.81805	1.51948
H	-1.56854	-0.17918	3.47765
H	0.48408	-0.75345	4.68659
C	-3.01279	1.00235	1.38504
C	-2.95902	-0.38959	1.32216
C	-4.22974	1.65979	1.16341
C	-4.10563	-1.12254	1.00860
H	-2.02535	-0.93519	1.45046
C	-5.37699	0.93362	0.85358
H	-4.23648	2.74793	1.18277
C	-5.31950	-0.47371	0.77104
O	-6.57752	1.48181	0.58132
O	-3.99726	-2.47227	0.85716
C	-4.70496	-3.26708	1.78790
H	-5.79063	-3.10886	1.70704
H	-4.46759	-4.31319	1.55549
H	-4.37868	-3.04573	2.81897
C	-6.71819	2.87584	0.66639
H	-7.76519	3.09885	0.42883
H	-6.49359	3.24444	1.68171
H	-6.06646	3.39838	-0.05444

N	-1.73006	2.85218	0.71609
N	-0.62726	3.60367	0.59928
O	1.57920	3.97134	1.03922
C	1.73155	4.67165	-0.19989
H	0.79629	4.57649	-0.76630
C	2.85103	4.02135	-0.97338
C	3.94839	3.46950	-0.34911
H	2.91585	4.29681	-2.02790
H	4.85987	3.27430	-0.91361
H	4.03932	3.52292	0.73522
O	-6.42515	-1.19017	0.44022
C	-6.68046	-1.24882	-0.94781
H	-7.58035	-1.86165	-1.08572
H	-6.86246	-0.24286	-1.36004
H	-5.83695	-1.71608	-1.48452
C	1.99846	6.13828	0.10038
H	2.16212	6.69663	-0.83279
H	1.13395	6.57445	0.62031
H	2.89025	6.24968	0.73478
C	1.08095	-5.29893	-3.07122
H	0.48232	-4.95981	-3.92862
H	1.98128	-5.81437	-3.42170
H	0.45543	-5.98454	-2.48273
C	5.51884	-1.58132	0.24490
H	4.97774	-0.67976	-0.08542
H	6.59684	-1.38372	0.21285
H	5.28315	-2.41488	-0.43769

PBE0+D3(BJ)/Def2-SVP Gibbs free energy:-2802.213528 a.u.

PBE0+D3(BJ)/Def2-SVP enthalpy:-2802.066600 a.u.

PBE0+D3(BJ)/Def2-SVP SCF energy:-2803.017887 a.u.

PBE0+D3(BJ)/Def2-TZVPP Gibbs free energy:-2803.063818 a.u.

PBE0+D3(BJ)/Def2-TZVPP enthalpy:-2802.916890 a.u.

PBE0+D3(BJ)/Def2-TZVPP SCF energy:-2803.868177 a.u.

Imaginary frequency: -418.8669 cm-1

(*S*, *R*)-1a-4

Cartesian coordinates

ATOM	X	Y	Z
C	-1.08507	-4.66087	-1.62628
O	-0.81451	-4.15425	-2.67806
O	-0.59445	-4.22220	-0.44677
C	0.13173	-3.00053	-0.47993
H	-0.32759	-2.36579	-1.24503
C	0.04816	-2.32572	0.89667

C	0.62534	-3.20064	2.03108
H	0.67358	-1.43036	0.79469
C	-2.16381	-2.89338	1.81890
C	-1.08312	-0.81936	2.38112
C	-0.16562	-2.93007	3.31242
H	0.53957	-4.26751	1.77638
H	1.69458	-2.98246	2.15423
H	-3.15591	-2.45352	1.99347
H	-2.26941	-3.64619	1.03243
C	-1.57647	-3.49204	3.11529
C	-0.26913	-1.41689	3.55866
H	-2.07611	-0.48199	2.70364
H	-0.58733	0.06190	1.95410
H	0.32327	-3.41814	4.16935
H	-2.20461	-3.24161	3.98429
H	-1.54253	-4.58999	3.04668
H	-0.85043	-1.27281	4.48580
N	-1.29946	-1.81105	1.29150
C	1.09336	-0.74679	3.76534
H	1.68405	-0.80199	2.83692
H	1.65442	-1.33062	4.51646
C	1.00314	0.70892	4.21111
H	0.48661	1.32953	3.46253
H	0.46108	0.79905	5.16679
H	2.00778	1.13541	4.35256
C	1.58334	-3.22484	-0.85989
C	2.34031	-2.19649	-1.49752
C	2.22373	-4.40288	-0.55179
C	1.78004	-0.96005	-1.91745
C	3.72541	-2.44568	-1.71274
C	3.60216	-4.54722	-0.82941
H	1.67580	-5.22470	-0.08868
C	2.57038	-0.00224	-2.51407
H	0.72687	-0.75650	-1.74331
C	4.51855	-1.42070	-2.30381
H	4.10709	-5.48601	-0.57304
C	3.96362	-0.23254	-2.68860
H	5.57999	-1.63027	-2.44890
H	4.55760	0.55564	-3.15489
N	4.33626	-3.60756	-1.37053
O	2.11723	1.17678	-2.97717
Os	-2.53861	-0.62043	-0.48899
O	-2.83920	-2.23097	-0.96039
O	-1.00673	0.14500	-0.57060

O	-3.40077	0.26481	-1.71331
O	-3.56597	-0.08371	0.81176
C	-1.39868	5.26314	-2.56406
C	-2.21376	4.66055	-1.63070
C	-1.63611	3.89451	-0.59599
C	-0.23641	3.74007	-0.51082
C	0.57737	4.34892	-1.49519
C	0.00168	5.10123	-2.49625
H	-1.83582	5.85931	-3.36758
H	-3.29856	4.76263	-1.67232
C	-2.40583	3.20822	0.40282
C	0.25951	2.91990	0.56616
H	1.65603	4.18971	-1.47194
H	0.63470	5.56513	-3.25572
C	1.68780	2.52454	0.69363
C	1.94995	1.16764	0.74851
C	2.73972	3.44677	0.79639
C	3.25005	0.67566	0.92254
H	1.13181	0.45934	0.65087
C	4.04035	2.97167	0.95150
H	2.52951	4.51554	0.79004
C	4.31358	1.58232	1.01456
O	5.12726	3.76485	1.06679
O	3.32555	-0.66705	0.99641
C	4.49369	-1.37245	1.35314
H	4.90445	-1.02091	2.31019
H	4.18273	-2.42194	1.43844
H	5.26679	-1.30532	0.57453
C	4.95831	5.15450	1.03787
H	4.32320	5.50626	1.86922
H	5.95717	5.59473	1.14419
H	4.51710	5.49480	0.08433
N	-0.54118	2.38560	1.44976
N	-1.87229	2.52988	1.37085
O	-3.73629	3.30109	0.29676
C	-4.57329	2.46857	1.10552
H	-3.98297	2.09963	1.95526
C	-5.06223	1.29076	0.29779
C	-5.05958	1.28205	-1.08020
H	-5.73304	0.61722	0.83412
H	-5.69870	0.58703	-1.62369
H	-4.69077	2.14954	-1.62602
O	5.59745	1.16740	1.19622
C	6.42176	1.23196	0.05233

H	7.40874	0.85311	0.34808
H	6.02003	0.60201	-0.76027
H	6.52746	2.26738	-0.30803
C	-5.72629	3.33131	1.59642
H	-6.29035	3.74625	0.74804
H	-6.41302	2.73489	2.21391
H	-5.34237	4.16099	2.20624
C	-2.02187	-5.81569	-1.41867
H	-2.16419	-6.35077	-2.36393
H	-1.65124	-6.49202	-0.63774
H	-2.98951	-5.40793	-1.08857
C	0.72410	1.36953	-3.04664
H	0.24062	0.56434	-3.62657
H	0.26336	1.40639	-2.04765
H	0.56355	2.32810	-3.55312

PBE0+D3(BJ)/Def2-SVP Gibbs free energy:-2802.215415 a.u.

PBE0+D3(BJ)/Def2-SVP enthalpy:-2802.070659 a.u.

PBE0+D3(BJ)/Def2-SVP SCF energy:-2803.021996 a.u.

PBE0+D3(BJ)/Def2-TZVPP Gibbs free energy:-2803.064582 a.u.

PBE0+D3(BJ)/Def2-TZVPP enthalpy:-2802.919826 a.u.

PBE0+D3(BJ)/Def2-TZVPP SCF energy:-2803.871163 a.u.

Imaginary frequency: -417.7165 cm-1

(*S*, *R*)-1a-5

Cartesian coordinates

ATOM	X	Y	Z
C	-0.63697	4.93334	-1.47470
O	-1.11593	5.18834	-0.40695
O	0.13117	3.84504	-1.70049
C	0.28328	2.92974	-0.62111
H	-0.67637	2.88386	-0.09523
C	0.64759	1.54569	-1.19069
C	1.90982	1.56553	-2.09203
H	0.83561	0.91961	-0.30751
C	-0.85878	1.52000	-3.15738
C	0.03608	-0.51577	-2.24764
C	1.55685	1.00439	-3.47202
H	2.29105	2.58887	-2.20412
H	2.71495	0.97652	-1.62783
H	-1.56371	0.84079	-3.65929
H	-1.41182	2.43151	-2.90855
C	0.37674	1.80476	-4.03362
C	1.12641	-0.46328	-3.34483
H	-0.82082	-1.13265	-2.54696

H	0.43552	-0.95935	-1.32730
H	2.42656	1.08713	-4.14142
H	0.18074	1.51534	-5.07698
H	0.61352	2.87841	-4.02311
H	0.67142	-0.75099	-4.30944
N	-0.46912	0.84248	-1.89989
C	2.27821	-1.43700	-3.08456
H	2.67351	-1.28372	-2.06518
H	3.10406	-1.19437	-3.77639
C	1.87913	-2.89915	-3.25532
H	2.73645	-3.56424	-3.07413
H	1.08722	-3.17871	-2.54373
H	1.51286	-3.08797	-4.27781
C	1.33483	3.42804	0.35414
C	1.29988	3.06256	1.73531
C	2.35948	4.24302	-0.06923
C	0.28587	2.24570	2.30552
C	2.35029	3.54890	2.56619
C	3.35279	4.66344	0.84428
H	2.40900	4.57485	-1.10722
C	0.30267	1.94139	3.65147
H	-0.50544	1.85328	1.67630
C	2.35571	3.18836	3.94466
H	4.17195	5.30124	0.49274
C	1.36686	2.40887	4.47447
H	3.17533	3.56708	4.55719
H	1.35253	2.13935	5.53190
N	3.36209	4.32881	2.10928
O	-0.64298	1.22877	4.28405
Os	-2.45247	0.52321	-0.45334
O	-2.67809	2.14727	-0.92408
O	-1.16230	-0.05450	0.51743
O	-3.78871	0.31609	0.64090
O	-3.04000	-0.62394	-1.62302
C	-1.98544	-2.47422	4.18025
C	-2.59321	-2.72304	2.96862
C	-1.80043	-2.85077	1.80987
C	-0.39770	-2.71630	1.87973
C	0.20091	-2.45391	3.13337
C	-0.58422	-2.33457	4.26052
H	-2.58953	-2.37744	5.08478
H	-3.67514	-2.82889	2.88578
C	-2.33928	-3.10839	0.50516
C	0.32903	-2.89188	0.64790

H	1.28518	-2.35768	3.20202
H	-0.11777	-2.12668	5.22516
C	1.80018	-2.70428	0.52346
C	2.40505	-1.49354	0.90174
C	2.54934	-3.70502	-0.07934
C	3.76413	-1.30056	0.64515
H	1.80061	-0.70671	1.35558
C	3.90886	-3.51370	-0.34769
H	2.08352	-4.63823	-0.39438
C	4.52100	-2.30706	0.00580
O	4.54382	-4.50819	-1.00625
O	4.44843	-0.17742	0.92949
C	3.75363	0.91617	1.46712
H	4.48459	1.71775	1.62391
H	2.97833	1.27496	0.77211
H	3.27711	0.67500	2.43274
C	5.86916	-4.85758	-0.66730
H	5.99865	-5.90603	-0.96807
H	6.60981	-4.23450	-1.18821
H	6.04093	-4.76916	0.41710
N	-0.26792	-3.22556	-0.46595
N	-1.60233	-3.32514	-0.54029
O	-3.67353	-3.12629	0.39507
C	-4.26519	-3.01970	-0.90481
H	-3.48187	-3.17817	-1.65702
C	-4.84131	-1.63581	-1.06754
C	-5.30337	-0.90859	0.00578
H	-5.17151	-1.37951	-2.07579
H	-5.96169	-0.05450	-0.15184
H	-5.28578	-1.34561	1.00331
O	5.82810	-2.07221	-0.29231
C	6.02059	-1.32012	-1.47081
H	5.59569	-1.83990	-2.34770
H	5.56208	-0.32129	-1.38505
H	7.10357	-1.20437	-1.60834
C	-5.32791	-4.10136	-1.02431
H	-4.86356	-5.09392	-0.94062
H	-6.08327	-3.99391	-0.23179
H	-5.83307	-4.03434	-1.99860
C	-0.80016	5.73463	-2.73545
H	0.18073	5.95054	-3.18079
H	-1.37488	5.14691	-3.46649
H	-1.33154	6.66591	-2.51275
C	-1.80164	0.86159	3.57074

H	-2.29461	1.74387	3.12726
H	-1.57385	0.13702	2.77305
H	-2.48059	0.39801	4.29488

PBE0+D3(BJ)/Def2-SVP Gibbs free energy:-2802.216031 a.u.

PBE0+D3(BJ)/Def2-SVP enthalpy:-2802.070311 a.u.

PBE0+D3(BJ)/Def2-SVP SCF energy:-2803.021235 a.u.

PBE0+D3(BJ)/Def2-TZVPP Gibbs free energy:-2803.066050 a.u.

PBE0+D3(BJ)/Def2-TZVPP enthalpy:-2802.920330 a.u.

PBE0+D3(BJ)/Def2-TZVPP SCF energy:-2803.871254 a.u.

Imaginary frequency: -389.3396 cm-1

(*S*, *R*)-1a-6

Cartesian coordinates

ATOM	X	Y	Z
C	-2.17942	-3.73553	2.41026
O	-3.25903	-3.34511	2.05693
O	-1.01804	-3.20657	1.98420
C	-1.07730	-2.13925	1.03003
H	-2.05645	-1.67084	1.14556
C	0.00987	-1.08529	1.34304
C	1.26439	-1.63150	2.07499
H	0.30678	-0.66545	0.37404
C	-1.03332	-0.35665	3.42650
C	0.50820	1.07651	2.26170
C	1.39763	-0.92219	3.42625
H	1.18076	-2.71439	2.23582
H	2.16133	-1.45215	1.46684
H	-1.35252	0.55493	3.95192
H	-1.93868	-0.96559	3.30601
C	0.08364	-1.09726	4.19558
C	1.63497	0.57919	3.20158
H	0.04648	2.00087	2.62941
H	0.90466	1.30176	1.26133
H	2.23479	-1.35485	3.99461
H	0.17994	-0.69589	5.21547
H	-0.15283	-2.16803	4.28473
H	1.51839	1.07558	4.18175
N	-0.56202	0.06611	2.09372
C	3.03421	0.91977	2.68475
H	3.17825	0.48922	1.68014
H	3.77749	0.43146	3.33785
C	3.31203	2.41761	2.62319
H	2.63790	2.92306	1.91666
H	3.20165	2.88467	3.61562

H	4.33720	2.60594	2.27145
C	-0.94591	-2.70556	-0.37100
C	-2.00093	-2.63042	-1.32914
C	0.23375	-3.30518	-0.75435
C	-3.29021	-2.09733	-1.04885
C	-1.71945	-3.11672	-2.64065
C	0.38777	-3.78755	-2.07055
H	1.05616	-3.40209	-0.04589
C	-4.24135	-2.00992	-2.04051
H	-3.53544	-1.80149	-0.03152
C	-2.72626	-3.00523	-3.64434
H	1.32837	-4.26645	-2.36828
C	-3.94462	-2.46016	-3.36031
H	-2.48405	-3.37756	-4.64117
H	-4.72583	-2.37136	-4.11728
N	-0.53988	-3.68550	-2.98932
O	-5.48338	-1.52762	-1.86963
Os	-2.45551	1.17085	0.72164
O	-3.38498	0.35192	1.89613
O	-1.53221	0.35993	-0.45335
O	-3.67451	1.92882	-0.25734
O	-1.75596	2.67318	1.26053
C	-0.85472	0.21389	-3.85105
C	-0.90791	1.36727	-3.10386
C	0.17263	1.70053	-2.26101
C	1.30890	0.86621	-2.17241
C	1.35209	-0.29451	-2.98312
C	0.28684	-0.61285	-3.79726
H	-1.69842	-0.07159	-4.48206
H	-1.78392	2.01532	-3.12098
C	0.16977	2.85750	-1.41448
C	2.34727	1.28709	-1.25985
H	2.23536	-0.93220	-2.97065
H	0.31887	-1.52649	-4.39349
C	3.60125	0.52450	-0.99098
C	3.61534	-0.86270	-0.79436
C	4.77302	1.26770	-0.82595
C	4.81498	-1.49891	-0.44466
H	2.69207	-1.43519	-0.88068
C	5.96484	0.63607	-0.46882
H	4.71835	2.34656	-0.95324
C	5.98828	-0.75189	-0.25833
O	7.13990	1.27089	-0.29458
O	4.95367	-2.82635	-0.24891

C	3.82651	-3.64916	-0.31347
H	4.17536	-4.67719	-0.15787
H	3.09995	-3.39776	0.47797
H	3.32327	-3.58816	-1.29433
C	7.17740	2.66775	-0.41573
H	6.50653	3.15754	0.31060
H	8.21060	2.97150	-0.20865
H	6.90338	2.99926	-1.43220
N	2.26138	2.41305	-0.59550
N	1.17767	3.19911	-0.66871
O	-0.94894	3.59056	-1.39877
C	-1.10512	4.60410	-0.39758
H	-0.30132	4.48247	0.33890
C	-2.43156	4.38179	0.28483
C	-3.56014	3.98116	-0.39635
H	-2.53990	4.85794	1.26161
H	-4.54532	4.10325	0.05305
H	-3.52344	3.83708	-1.47595
O	7.12768	-1.36878	0.13549
C	7.99691	-1.73439	-0.91149
H	7.51164	-2.44374	-1.60391
H	8.33834	-0.85009	-1.47577
H	8.86701	-2.22280	-0.45407
C	-0.98858	5.97146	-1.05273
H	-1.73652	6.08692	-1.85103
H	-1.14562	6.76626	-0.30900
H	0.01617	6.09215	-1.48194
C	-1.92961	-4.84887	3.38821
H	-1.26206	-5.59774	2.94049
H	-1.42743	-4.44928	4.28124
H	-2.88221	-5.30810	3.67243
C	-5.83868	-1.00433	-0.61216
H	-5.73565	-1.75588	0.18832
H	-5.22880	-0.12104	-0.36170
H	-6.88968	-0.70170	-0.68752

PBE0+D3(BJ)/Def2-SVP Gibbs free energy:-2802.208992 a.u.

PBE0+D3(BJ)/Def2-SVP enthalpy:-2802.060655 a.u.

PBE0+D3(BJ)/Def2-SVP SCF energy:-2803.011790 a.u.

PBE0+D3(BJ)/Def2-TZVPP Gibbs free energy:-2803.062688 a.u.

PBE0+D3(BJ)/Def2-TZVPP enthalpy:-2802.914351 a.u.

PBE0+D3(BJ)/Def2-TZVPP SCF energy:-2803.865486 a.u.

Imaginary frequency: -427.2379 cm-1

(*S*, *S*)-2a-3

Cartesian coordinates

ATOM	X	Y	Z
C	-0.94384	-4.98912	-1.31212
O	-0.27865	-5.31255	-0.36973
O	-1.46092	-3.75090	-1.46272
C	-1.13068	-2.78876	-0.46543
H	-0.08434	-2.95475	-0.18265
C	-1.29855	-1.37394	-1.05107
C	-2.68546	-1.12780	-1.70311
H	-1.16894	-0.70109	-0.19270
C	-0.22534	-1.77577	-3.23469
C	-0.48766	0.45238	-2.36941
C	-2.49258	-0.74244	-3.17394
H	-3.30938	-2.02872	-1.64530
H	-3.22345	-0.33561	-1.16098
H	0.49965	-1.30111	-3.91200
H	0.16094	-2.77464	-3.00203
C	-1.63861	-1.82057	-3.85339
C	-1.73271	0.58682	-3.27111
H	0.40822	0.85079	-2.85792
H	-0.61454	1.01670	-1.43442
H	-3.47167	-0.66046	-3.67025
H	-1.58778	-1.63927	-4.93760
H	-2.09265	-2.80968	-3.70021
H	-1.40089	0.69432	-4.31960
N	-0.22025	-0.96516	-1.99800
C	-2.55406	1.82794	-2.93135
H	-2.84958	1.79673	-1.86979
H	-3.49025	1.81019	-3.51568
C	-1.80281	3.12727	-3.20454
H	-1.52145	3.20531	-4.26726
H	-2.42671	3.99796	-2.95437
H	-0.88375	3.19255	-2.60136
C	-2.01545	-2.97035	0.75497
C	-1.57471	-2.59018	2.05984
C	-3.29189	-3.46988	0.62906
C	-0.25719	-2.13975	2.34069
C	-2.51719	-2.69257	3.12422
C	-4.13951	-3.53735	1.75799
H	-3.66018	-3.81035	-0.33949
C	0.11639	-1.81961	3.62916
H	0.46322	-2.06237	1.53454
C	-2.11532	-2.29851	4.43322
H	-5.15994	-3.92046	1.64217

C	-0.84066	-1.87393	4.68203
H	-2.85987	-2.36972	5.22763
H	-0.51449	-1.59376	5.68515
N	-3.78425	-3.14764	2.95586
O	1.35913	-1.46920	3.99689
Os	2.05212	-0.90640	-0.98333
O	1.99078	-2.58304	-1.28272
O	1.07357	-0.17714	0.21609
O	3.62543	-0.68210	-0.27860
O	2.42079	0.01944	-2.41975
C	2.50740	1.64178	4.42223
C	3.20810	1.72427	3.23920
C	2.52105	2.03385	2.04757
C	1.13359	2.27374	2.05525
C	0.43854	2.19406	3.28470
C	1.11720	1.87413	4.44103
H	3.02501	1.38850	5.34938
H	4.28218	1.54140	3.19799
C	3.16254	2.11612	0.76626
C	0.53206	2.62537	0.79027
H	-0.63276	2.39360	3.31307
H	0.57251	1.80389	5.38452
C	-0.93139	2.86018	0.65435
C	-1.84869	1.88713	1.06817
C	-1.36804	4.04273	0.06004
C	-3.21921	2.11378	0.89182
H	-1.48404	0.95583	1.50323
C	-2.73515	4.26431	-0.12693
H	-0.62332	4.75976	-0.28097
C	-3.66835	3.31212	0.31047
O	-3.25176	5.35513	-0.72766
O	-4.18661	1.24349	1.23628
C	-3.82637	0.07080	1.91885
H	-4.75509	-0.44925	2.17913
H	-3.21592	-0.59334	1.28660
H	-3.26748	0.28773	2.84504
C	-2.38606	6.39361	-1.10127
H	-1.83760	6.79718	-0.23294
H	-1.65743	6.06705	-1.86333
H	-3.01402	7.18591	-1.52631
N	1.23452	2.77007	-0.30178
N	2.55367	2.49604	-0.31874
O	4.45202	1.78180	0.73324
C	5.13909	1.81293	-0.51971

H	4.78512	2.69799	-1.06792
C	4.90695	0.59249	-1.39817
C	4.26027	0.71617	-2.61408
H	5.59833	-0.23878	-1.25101
H	4.46115	0.00104	-3.41235
H	3.85940	1.68696	-2.90933
O	-4.99570	3.54372	0.16562
C	-5.54809	3.05166	-1.03301
H	-6.61651	3.30323	-1.02555
H	-5.07529	3.52022	-1.91353
H	-5.43884	1.95565	-1.10408
C	6.61260	1.95223	-0.17937
H	6.78515	2.86687	0.40457
H	6.95213	1.08912	0.41266
H	7.21247	2.00320	-1.09916
C	-1.31210	-5.86089	-2.47978
H	-2.40241	-5.87036	-2.61643
H	-0.86672	-5.45061	-3.39795
H	-0.94209	-6.87751	-2.31022
C	2.37769	-1.49086	3.02463
H	3.31274	-1.25339	3.54356
H	2.46511	-2.48709	2.55749
H	2.20005	-0.73693	2.24305

PBE0+D3(BJ)/Def2-SVP Gibbs free energy:-2802.214468 a.u.

PBE0+D3(BJ)/Def2-SVP enthalpy:-2802.070573 a.u.

PBE0+D3(BJ)/Def2-SVP SCF energy:-2803.022284 a.u.

PBE0+D3(BJ)/Def2-TZVPP Gibbs free energy:-2803.065570 a.u.

PBE0+D3(BJ)/Def2-TZVPP enthalpy:-2802.921675 a.u.

PBE0+D3(BJ)/Def2-TZVPP SCF energy:-2803.873386 a.u.

Imaginary frequency: -454.4792 cm-1

(*S*, *S*)-2a-4

Cartesian coordinates

ATOM	X	Y	Z
C	-1.11299	-4.87531	-1.40622
O	-0.44355	-5.25607	-0.48869
O	-1.59010	-3.61564	-1.50176
C	-1.20992	-2.70162	-0.47731
H	-0.16517	-2.91269	-0.21838
C	-1.33493	-1.26330	-1.01220
C	-2.72491	-0.93503	-1.62169
H	-1.15764	-0.62469	-0.13592
C	-0.33369	-1.62885	-3.23857
C	-0.48489	0.57136	-2.28222

C	-2.55403	-0.50533	-3.08440
H	-3.38642	-1.80979	-1.57846
H	-3.21369	-0.14076	-1.03708
H	0.39254	-1.15647	-3.91602
H	0.01712	-2.65082	-3.05571
C	-1.76230	-1.59367	-3.82121
C	-1.73432	0.79201	-3.15631
H	0.41432	0.96392	-2.76980
H	-0.58034	1.10126	-1.32521
H	-3.54111	-0.37463	-3.55207
H	-1.73069	-1.37776	-4.89982
H	-2.25390	-2.56778	-3.68935
H	-1.41508	0.91770	-4.20659
N	-0.26397	-0.86760	-1.97240
C	-2.46036	2.07184	-2.74777
H	-1.70804	2.87402	-2.66490
H	-2.86442	1.95596	-1.72881
C	-3.56386	2.51227	-3.70051
H	-3.18687	2.60766	-4.73148
H	-4.40379	1.80018	-3.71854
H	-3.96688	3.49178	-3.40255
C	-2.07792	-2.90102	0.75264
C	-1.60363	-2.58421	2.06279
C	-3.36807	-3.36543	0.63185
C	-0.27858	-2.14788	2.33248
C	-2.52296	-2.72713	3.14281
C	-4.19246	-3.47085	1.77491
H	-3.76313	-3.65389	-0.34309
C	0.12406	-1.88322	3.62542
H	0.42248	-2.02941	1.51372
C	-2.08848	-2.39839	4.45927
H	-5.22322	-3.82669	1.66386
C	-0.80744	-1.98792	4.69739
H	-2.81523	-2.50296	5.26635
H	-0.45704	-1.75380	5.70410
N	-3.80104	-3.15300	2.98253
O	1.37287	-1.54270	3.98056
Os	2.03889	-0.92994	-1.03037
O	1.90107	-2.59746	-1.35124
O	1.12986	-0.18514	0.21411
O	3.64307	-0.77227	-0.37945
O	2.39305	-0.00041	-2.46870
C	2.76962	1.60029	4.36651
C	3.42671	1.65463	3.15709

C	2.70555	1.98191	1.99071
C	1.32686	2.26473	2.04668
C	0.67948	2.21855	3.30382
C	1.39042	1.88173	4.43607
H	3.31371	1.33510	5.27506
H	4.49207	1.43749	3.07587
C	3.30557	2.04444	0.68864
C	0.68935	2.62011	0.79855
H	-0.38118	2.45890	3.37438
H	0.88003	1.83844	5.40012
C	-0.77658	2.86565	0.70624
C	-1.68382	1.93636	1.22914
C	-1.23298	4.00851	0.04941
C	-3.05834	2.18884	1.14200
H	-1.31064	1.02240	1.69216
C	-2.60667	4.24725	-0.06152
H	-0.49866	4.68198	-0.38912
C	-3.52489	3.36165	0.52447
O	-3.14551	5.29411	-0.71662
O	-4.01573	1.37083	1.61848
C	-3.63209	0.15882	2.21312
H	-2.97410	0.31259	3.08544
H	-4.54785	-0.34266	2.54603
H	-3.11407	-0.49319	1.49211
C	-2.28892	6.21553	-1.33424
H	-1.63030	6.71620	-0.60383
H	-1.66261	5.73799	-2.10799
H	-2.92945	6.96880	-1.80873
N	1.36150	2.75027	-0.31433
N	2.67306	2.44777	-0.37404
O	4.58185	1.66920	0.61282
C	5.22997	1.68135	-0.66091
H	4.88996	2.58001	-1.19573
C	4.92876	0.47266	-1.53432
C	4.24482	0.62301	-2.72674
H	5.59615	-0.38193	-1.41273
H	4.39320	-0.09596	-3.53308
H	3.87155	1.60922	-3.00668
O	-4.85101	3.63676	0.48782
C	-5.57809	2.97053	-0.51788
H	-5.24743	3.28205	-1.52236
H	-5.48240	1.87543	-0.42613
H	-6.63296	3.24562	-0.39002
C	6.71727	1.76927	-0.36638

H	7.04537	0.89289	0.21243
H	7.28964	1.80329	-1.30429
H	6.93895	2.67532	0.21438
C	-1.53088	-5.68981	-2.59833
H	-1.08851	-5.26004	-3.50901
H	-1.19219	-6.72371	-2.47370
H	-2.62318	-5.65842	-2.71440
C	2.37285	-1.52994	2.98878
H	2.44466	-2.50685	2.47986
H	2.18432	-0.74459	2.24132
H	3.31889	-1.31940	3.49918

PBE0+D3(BJ)/Def2-SVP Gibbs free energy:-2802.213715 a.u.

PBE0+D3(BJ)/Def2-SVP enthalpy:-2802.067707 a.u.

PBE0+D3(BJ)/Def2-SVP SCF energy:-2803.019260 a.u.

PBE0+D3(BJ)/Def2-TZVPP Gibbs free energy:-2803.066298 a.u.

PBE0+D3(BJ)/Def2-TZVPP enthalpy:-2802.920290 a.u.

PBE0+D3(BJ)/Def2-TZVPP SCF energy:-2803.871843 a.u.

Imaginary frequency: -455.0764 cm-1

(*S*, *S*)-2a-7

Cartesian coordinates

ATOM	X	Y	Z
C	0.28408	5.11948	-1.06014
O	-0.36687	5.31851	-0.07441
O	0.93287	3.95902	-1.29678
C	0.76275	2.91773	-0.33994
H	-0.28056	2.94727	-0.00280
C	1.05896	1.56216	-1.00857
C	2.43955	1.49948	-1.71530
H	1.03318	0.83551	-0.18516
C	-0.12922	1.95707	-3.13412
C	0.39904	-0.26946	-2.40031
C	2.23595	1.17200	-3.19885
H	2.96804	2.45725	-1.62512
H	3.07556	0.74094	-1.23426
H	-0.82072	1.43890	-3.81437
H	-0.61342	2.89476	-2.83889
C	1.24903	2.18740	-3.78998
C	1.61540	-0.22324	-3.34772
H	-0.46676	-0.73847	-2.87978
H	0.62218	-0.86331	-1.50292
H	3.19970	1.22154	-3.72850
H	1.17944	2.06074	-4.88087
H	1.60058	3.21002	-3.59306

H	1.25682	-0.30821	-4.38939
N	-0.00369	1.08974	-1.94277
C	2.56536	-1.39384	-3.10527
H	2.89563	-1.38763	-2.05317
H	3.47292	-1.25345	-3.71823
C	1.92808	-2.74328	-3.42485
H	1.63050	-2.79553	-4.48484
H	2.62303	-3.57150	-3.22389
H	1.03059	-2.91565	-2.81046
C	1.67919	3.14345	0.84927
C	1.35588	2.64186	2.14731
C	2.87397	3.80991	0.69788
C	0.12804	1.99597	2.45317
C	2.32529	2.81950	3.17701
C	3.75902	3.93313	1.79278
H	3.14733	4.24291	-0.26524
C	-0.13649	1.56259	3.73600
H	-0.61071	1.85399	1.67208
C	2.04219	2.31116	4.47759
H	4.71527	4.45112	1.65583
C	0.85118	1.70075	4.75246
H	2.80634	2.44612	5.24463
H	0.61344	1.32888	5.75061
N	3.51290	3.44599	2.98232
O	-1.29866	1.02072	4.13233
Os	-2.21856	0.72268	-0.86840
O	-2.34906	2.41273	-1.04128
O	-1.12530	0.02386	0.24817
O	-3.73239	0.27052	-0.14369
O	-2.53415	-0.12725	-2.36369
C	-2.22806	-2.27210	4.33220
C	-2.94479	-2.34477	3.15801
C	-2.25745	-2.47816	1.93436
C	-0.85184	-2.55404	1.89946
C	-0.13931	-2.48644	3.11950
C	-0.82023	-2.33781	4.30898
H	-2.74748	-2.15554	5.28534
H	-4.03352	-2.28717	3.14932
C	-2.91816	-2.53385	0.66150
C	-0.24663	-2.73378	0.60030
H	0.94832	-2.55966	3.11446
H	-0.26311	-2.27538	5.24569
C	1.23111	-2.78443	0.42486
C	2.03931	-1.73736	0.90024

C	1.80121	-3.85998	-0.24135
C	3.41935	-1.78929	0.70160
H	1.57572	-0.88807	1.40385
C	3.18399	-3.91329	-0.45271
H	1.18257	-4.66981	-0.62734
C	4.00030	-2.88005	0.01774
O	3.65176	-4.96377	-1.16490
O	4.28714	-0.83780	1.09414
C	3.79771	0.26179	1.81726
H	3.11401	0.87194	1.20651
H	3.26878	-0.04940	2.73408
H	4.66168	0.87500	2.09704
C	4.85028	-5.60563	-0.78337
H	4.78777	-6.63222	-1.16895
H	5.73836	-5.11183	-1.20256
H	4.95443	-5.63745	0.31271
N	-0.95559	-2.87092	-0.48796
N	-2.29751	-2.75116	-0.46083
O	-4.23813	-2.35258	0.67888
C	-4.95274	-2.36658	-0.55878
H	-4.50797	-3.15086	-1.18813
C	-4.89982	-1.05733	-1.33112
C	-4.28963	-1.00064	-2.57076
H	-5.67612	-0.32930	-1.09060
H	-4.59991	-0.24749	-3.29575
H	-3.79710	-1.89098	-2.96455
O	5.34483	-2.89622	-0.19416
C	5.76536	-2.14542	-1.31198
H	5.48328	-1.08450	-1.20979
H	6.85958	-2.22014	-1.35895
H	5.33290	-2.54530	-2.24605
C	-6.38690	-2.71897	-0.20408
H	-6.42679	-3.69376	0.30146
H	-6.81366	-1.95841	0.46686
H	-7.00266	-2.76844	-1.11338
C	0.49170	6.08135	-2.19624
H	1.56560	6.22501	-2.37972
H	0.05181	5.66435	-3.11405
H	0.01592	7.03863	-1.95869
C	-2.35884	0.95864	3.20701
H	-3.22770	0.57149	3.75005
H	-2.59806	1.95836	2.80483
H	-2.12265	0.27934	2.37407

PBE0+D3(BJ)/Def2-SVP Gibbs free energy:-2802.213020 a.u.

PBE0+D3(BJ)/Def2-SVP enthalpy:-2802.068459 a.u.

PBE0+D3(BJ)/Def2-SVP SCF energy:-2803.019954 a.u.

PBE0+D3(BJ)/Def2-TZVPP Gibbs free energy:-2803.062581 a.u.

PBE0+D3(BJ)/Def2-TZVPP enthalpy:-2802.918020 a.u.

PBE0+D3(BJ)/Def2-TZVPP SCF energy:-2803.869515 a.u.

Imaginary frequency: -454.7582 cm-1

(*S*, *R*)-3-6

Cartesian coordinates

ATOM	X	Y	Z
C	-0.65641	0.83423	-2.72799
O	-0.02015	1.57306	-2.02662
O	-0.81462	-0.47713	-2.48548
C	-0.41539	-0.98449	-1.21684
H	-0.50790	-0.18046	-0.47299
C	-1.31525	-2.18347	-0.85904
C	-1.12953	-3.37467	-1.82472
H	-0.96956	-2.47793	0.13971
C	-3.49288	-1.89412	-1.96540
C	-3.29967	-3.03519	0.14475
C	-2.44377	-4.15334	-1.89753
H	-0.86165	-3.01615	-2.83081
H	-0.29628	-4.00473	-1.48444
H	-4.52241	-1.57491	-1.75365
H	-3.02550	-1.12533	-2.58818
C	-3.45964	-3.28149	-2.64203
C	-2.97585	-4.41737	-0.47961
H	-4.38024	-2.87593	0.24263
H	-2.86499	-2.93788	1.15139
H	-2.29517	-5.10678	-2.42672
H	-4.45288	-3.75653	-2.61901
H	-3.17346	-3.18370	-3.70011
H	-3.91999	-4.97961	-0.58128
N	-2.76862	-1.91508	-0.67285
C	-2.03257	-5.26512	0.38107
H	-1.11720	-4.69427	0.61353
H	-1.70454	-6.13762	-0.20914
C	-2.66804	-5.74553	1.68157
H	-3.55713	-6.36497	1.48375
H	-1.96198	-6.34862	2.27062
H	-2.98629	-4.90268	2.31495
C	1.01062	-1.49823	-1.24042
C	1.64244	-1.89533	-0.02364
C	1.66528	-1.74650	-2.42360

C	1.11672	-1.58506	1.25916
C	2.85233	-2.63648	-0.12886
C	2.89783	-2.43834	-2.40600
H	1.21800	-1.45605	-3.37535
C	1.77047	-2.01418	2.39265
H	0.25052	-0.93143	1.34052
C	3.46283	-3.11814	1.06405
H	3.41720	-2.64039	-3.35023
C	2.93295	-2.82798	2.28849
H	4.37964	-3.70124	0.96933
H	3.40838	-3.17009	3.20903
N	3.45445	-2.90089	-1.31601
O	1.40964	-1.67893	3.64623
Os	-3.34290	0.16002	0.58135
O	-4.85831	-0.61797	0.50117
O	-2.89960	0.95750	-0.90840
O	-3.77862	1.62384	1.40176
O	-2.05424	-0.43776	1.53080
C	1.91887	6.06869	-1.27831
C	0.77383	5.57411	-0.69309
C	0.78146	4.27306	-0.14775
C	1.93293	3.46904	-0.20675
C	3.10573	4.01186	-0.78452
C	3.09152	5.28591	-1.31145
H	1.92585	7.07416	-1.70434
H	-0.13896	6.16721	-0.63618
C	-0.34759	3.69634	0.52363
C	1.83570	2.15054	0.38188
H	4.02128	3.42082	-0.79967
H	4.00207	5.69739	-1.75242
C	3.00948	1.23947	0.35169
C	3.60929	0.91968	-0.86664
C	3.54327	0.75224	1.54319
C	4.77335	0.14694	-0.89558
H	3.15722	1.28175	-1.78886
C	4.68789	-0.04781	1.51922
H	3.04397	0.99249	2.48043
C	5.32791	-0.34684	0.29830
O	5.26264	-0.57077	2.61879
O	5.43914	-0.15521	-2.03663
C	4.96894	0.35724	-3.24915
H	5.65656	0.00370	-4.02759
H	3.95121	-0.00221	-3.48170
H	4.96182	1.46182	-3.25264

C	4.72031	-0.26204	3.87401
H	4.75215	0.82364	4.07491
H	3.67979	-0.61489	3.97523
H	5.34322	-0.77625	4.61649
N	0.75326	1.73406	0.98212
N	-0.34998	2.50986	1.04875
O	-1.42230	4.48635	0.59976
C	-2.59752	4.04218	1.27268
H	-2.30415	3.31475	2.04338
C	-3.54492	3.37337	0.30073
C	-3.17578	2.97448	-0.96657
H	-4.60594	3.52603	0.50424
H	-3.94407	2.83533	-1.72697
H	-2.15367	3.11373	-1.32084
O	6.46975	-1.05812	0.36401
C	6.70911	-2.12095	-0.53883
H	7.32055	-2.85660	0.00336
H	5.77248	-2.59438	-0.87013
H	7.26364	-1.77314	-1.42306
C	-3.22729	5.26657	1.91461
H	-3.51062	6.00702	1.15142
H	-4.12786	4.97370	2.47283
H	-2.52077	5.73456	2.61348
C	-1.40677	1.24108	-3.96346
H	-1.31183	0.48465	-4.75327
H	-2.47116	1.31811	-3.69361
H	-1.04950	2.21619	-4.31253
C	0.32450	-0.78915	3.80638
H	-0.61874	-1.23807	3.45641
H	0.25289	-0.57833	4.88002
H	0.48565	0.14702	3.24609

PBE0+D3(BJ)/Def2-SVP Gibbs free energy:-2802.214763 a.u.

PBE0+D3(BJ)/Def2-SVP enthalpy:-2802.071768 a.u.

PBE0+D3(BJ)/Def2-SVP SCF energy:-2803.023336 a.u.

PBE0+D3(BJ)/Def2-TZVPP Gibbs free energy:-2803.066113 a.u.

PBE0+D3(BJ)/Def2-TZVPP enthalpy:-2802.923118 a.u.

PBE0+D3(BJ)/Def2-TZVPP SCF energy:-2803.874686 a.u.

Imaginary frequency: -421.7862 cm-1

(*S*, *R*)-3-7

Cartesian coordinates

ATOM	X	Y	Z
C	-0.82791	0.70388	-2.73846
O	-0.26555	1.53127	-2.07458

O	-0.86767	-0.60323	-2.43054
C	-0.42113	-1.01205	-1.14266
H	-0.56366	-0.17903	-0.44026
C	-1.22711	-2.25367	-0.71193
C	-0.96645	-3.47241	-1.62567
H	-0.84802	-2.47343	0.29385
C	-3.43321	-2.16755	-1.79560
C	-3.13628	-3.20278	0.35327
C	-2.22221	-4.34354	-1.64946
H	-0.73097	-3.14227	-2.64928
H	-0.08846	-4.02717	-1.26579
H	-4.48031	-1.91239	-1.58291
H	-3.02876	-1.39617	-2.45778
C	-3.30908	-3.57762	-2.41109
C	-2.71312	-4.58289	-0.21165
H	-4.22576	-3.12122	0.44956
H	-2.70669	-3.03636	1.35312
H	-2.01663	-5.30004	-2.15124
H	-4.26488	-4.12128	-2.35036
H	-3.04607	-3.50699	-3.47729
H	-3.60992	-5.22338	-0.27166
N	-2.69396	-2.08198	-0.51451
C	-1.70486	-5.29454	0.69903
H	-2.17720	-5.42185	1.68782
H	-0.82976	-4.64569	0.87561
C	-1.23873	-6.65077	0.18363
H	-0.64640	-6.55572	-0.73925
H	-0.60679	-7.15935	0.92615
H	-2.09454	-7.30929	-0.03594
C	1.03819	-1.42332	-1.15954
C	1.70482	-1.72527	0.06678
C	1.70313	-1.67152	-2.33568
C	1.17633	-1.38068	1.33973
C	2.95277	-2.40176	-0.01961
C	2.98314	-2.26985	-2.30041
H	1.23652	-1.44719	-3.29545
C	1.86472	-1.71411	2.48482
H	0.27697	-0.77137	1.39884
C	3.57872	-2.82056	1.18828
H	3.51492	-2.45277	-3.24121
C	3.05258	-2.49219	2.40515
H	4.50073	-3.39585	1.11034
H	3.54133	-2.78637	3.33568
N	3.57393	-2.66183	-1.19969

O	1.51239	-1.32144	3.72396
Os	-3.39382	-0.00231	0.66424
O	-4.85454	-0.88211	0.64068
O	-3.02666	0.75982	-0.86380
O	-3.91089	1.46277	1.43392
O	-2.05409	-0.47251	1.61503
C	1.40043	6.12252	-1.66399
C	0.32404	5.58677	-0.99116
C	0.44756	4.31868	-0.38599
C	1.64563	3.58775	-0.47147
C	2.74577	4.17257	-1.14315
C	2.61802	5.41430	-1.72873
H	1.31739	7.10297	-2.13770
H	-0.62236	6.12163	-0.91092
C	-0.60709	3.70474	0.36823
C	1.66621	2.29956	0.18766
H	3.69530	3.64010	-1.18891
H	3.47303	5.85702	-2.24411
C	2.90014	1.47018	0.15546
C	3.49620	1.07183	1.35634
C	3.49300	1.13819	-1.05525
C	4.71261	0.39492	1.33031
H	3.00082	1.31216	2.29533
C	4.71655	0.45497	-1.09607
H	3.02260	1.39599	-2.00473
C	5.35809	0.11140	0.10377
O	5.20607	0.23996	-2.33555
O	5.37789	0.00696	2.43960
C	4.82896	0.30518	3.69664
H	4.74253	1.39492	3.85008
H	3.83688	-0.15666	3.83477
H	5.52113	-0.10390	4.44283
C	6.13021	-0.78866	-2.61218
H	7.14332	-0.53426	-2.27231
H	5.81496	-1.73946	-2.15352
H	6.13019	-0.90397	-3.70475
N	0.64594	1.84721	0.86526
N	-0.50470	2.54858	0.94848
O	-1.72795	4.42591	0.45937
C	-2.85046	3.93040	1.18464
H	-2.48785	3.24238	1.96179
C	-3.78724	3.18174	0.26138
C	-3.42726	2.75205	-0.99832
H	-4.84953	3.28801	0.48641

H	-4.20511	2.53904	-1.73152
H	-2.42344	2.93096	-1.38558
O	6.61807	-0.40367	0.09352
C	6.78849	-1.73805	0.52112
H	6.48517	-1.86581	1.57058
H	6.21114	-2.43196	-0.11353
H	7.85866	-1.96357	0.42366
C	-3.53110	5.13056	1.82002
H	-2.83146	5.65709	2.48324
H	-3.88389	5.83374	1.05054
H	-4.39329	4.79801	2.41540
C	-1.60752	0.97981	-3.99172
H	-1.45377	0.19002	-4.73826
H	-2.67486	0.99043	-3.72234
H	-1.32688	1.95850	-4.39635
C	0.38961	-0.47363	3.85417
H	0.49652	0.43293	3.23539
H	-0.53779	-0.98559	3.55219
H	0.32899	-0.20124	4.91455

PBE0+D3(BJ)/Def2-SVP Gibbs free energy:-2802.213977 a.u.

PBE0+D3(BJ)/Def2-SVP enthalpy:-2802.071533 a.u.

PBE0+D3(BJ)/Def2-SVP SCF energy:-2803.023114 a.u.

PBE0+D3(BJ)/Def2-TZVPP Gibbs free energy:-2803.063517 a.u.

PBE0+D3(BJ)/Def2-TZVPP enthalpy:-2802.921073 a.u.

PBE0+D3(BJ)/Def2-TZVPP SCF energy:-2803.872654 a.u.

Imaginary frequency: -420.1716 cm-1

(*S*, *R*)-3a-11

Cartesian coordinates

ATOM	X	Y	Z
C	0.15307	4.92518	-0.20950
O	-0.16982	4.87884	0.94374
O	0.65790	3.86776	-0.87920
C	0.70808	2.63099	-0.17041
H	-0.19639	2.57745	0.44188
C	0.73701	1.47950	-1.18837
C	1.87216	1.63815	-2.23051
H	0.91043	0.57390	-0.59073
C	-0.87080	2.24744	-2.89990
C	-0.44829	-0.08643	-2.57533
C	1.33216	1.28951	-3.61722
H	2.23334	2.67485	-2.25091
H	2.72645	1.00455	-1.95349
H	-1.83924	1.97130	-3.34138

H	-1.00861	3.20556	-2.39096
C	0.24238	2.30926	-3.96957
C	0.71349	-0.11519	-3.59750
H	-1.41514	-0.28803	-3.05264
H	-0.30683	-0.86502	-1.81684
H	2.14191	1.34527	-4.35968
H	-0.16551	2.09231	-4.96865
H	0.67779	3.31928	-4.00886
H	0.30041	-0.31102	-4.60237
N	-0.56628	1.22034	-1.88051
C	1.69360	-1.24127	-3.26287
H	1.11854	-2.17766	-3.18535
H	2.09700	-1.07926	-2.24951
C	2.84153	-1.42458	-4.24577
H	2.47033	-1.61959	-5.26487
H	3.48735	-0.53291	-4.29772
H	3.46917	-2.27722	-3.94523
C	1.91206	2.59025	0.75585
C	1.84675	1.95579	2.03367
C	3.10381	3.17229	0.38981
C	0.66632	1.36126	2.55413
C	3.03036	1.94939	2.82389
C	4.21996	3.09832	1.25265
H	3.19464	3.70345	-0.55818
C	0.65734	0.78725	3.80677
H	-0.23458	1.34815	1.95365
C	2.99769	1.31678	4.09986
H	5.16843	3.55322	0.94388
C	1.85114	0.74902	4.58053
H	3.91971	1.31718	4.68373
H	1.81192	0.27843	5.56454
N	4.19698	2.51137	2.42182
O	-0.43204	0.25338	4.38833
Os	-2.46588	0.95412	-0.30797
O	-1.22806	0.02925	0.43173
O	-3.29293	0.12452	-1.59679
O	-3.74909	0.73328	0.84688
O	-2.45192	2.65157	-0.47174
C	-2.30600	-2.95844	3.83225
C	-2.94777	-2.89726	2.61367
C	-2.18323	-2.84630	1.43065
C	-0.77342	-2.84547	1.48518
C	-0.13964	-2.89959	2.74718
C	-0.89766	-2.95530	3.89760

H	-2.88857	-3.00191	4.75480
H	-4.03581	-2.89441	2.54308
C	-2.75786	-2.78546	0.11658
C	-0.08064	-2.82251	0.22134
H	0.94941	-2.90458	2.80378
H	-0.40297	-2.98930	4.86963
C	1.39323	-2.67103	0.09313
C	2.05828	-1.61250	0.72237
C	2.09403	-3.50609	-0.77864
C	3.40665	-1.37961	0.45367
H	1.54002	-0.92663	1.39643
C	3.43706	-3.27154	-1.06266
H	1.58398	-4.31712	-1.29863
C	4.10126	-2.19199	-0.45849
O	4.04836	-4.03962	-2.00117
O	4.02960	-0.31783	1.02766
C	5.03404	-0.63057	1.97574
H	4.60610	-1.20678	2.81386
H	5.85352	-1.20482	1.51544
H	5.41016	0.32769	2.35473
C	5.16236	-4.80021	-1.58200
H	5.48791	-5.38697	-2.45027
H	5.98854	-4.15796	-1.24306
H	4.88007	-5.49242	-0.76985
N	-0.71624	-2.89604	-0.91839
N	-2.05733	-2.87152	-0.97180
O	-4.08775	-2.64356	0.04850
C	-4.68754	-2.22693	-1.18322
H	-3.93558	-2.30743	-1.97935
C	-5.12759	-0.78881	-1.05820
C	-5.43114	-0.21608	0.15679
H	-5.50809	-0.33742	-1.97623
H	-6.00790	0.70762	0.19859
H	-5.38580	-0.81684	1.06400
O	5.39975	-1.93377	-0.77563
C	5.59858	-0.77462	-1.56444
H	5.04959	-0.85466	-2.51655
H	5.27528	0.13179	-1.02783
H	6.67393	-0.71117	-1.77305
C	-5.85412	-3.15934	-1.47229
H	-6.36483	-2.85716	-2.39792
H	-5.48916	-4.18849	-1.59645
H	-6.58289	-3.13745	-0.64853
C	0.03607	6.12103	-1.11201

H	0.95061	6.25103	-1.70559
H	-0.79819	5.95700	-1.81060
H	-0.16645	7.01496	-0.51244
C	-1.66562	0.36046	3.71720
H	-1.91767	1.41440	3.50803
H	-1.66217	-0.20149	2.76907
H	-2.42382	-0.06479	4.38417

PBE0+D3(BJ)/Def2-SVP Gibbs free energy:-2802.216319 a.u.

PBE0+D3(BJ)/Def2-SVP enthalpy:-2802.071120 a.u.

PBE0+D3(BJ)/Def2-SVP SCF energy:-2803.022563 a.u.

PBE0+D3(BJ)/Def2-TZVPP Gibbs free energy:-2803.063944 a.u.

PBE0+D3(BJ)/Def2-TZVPP enthalpy:-2802.918745 a.u.

PBE0+D3(BJ)/Def2-TZVPP SCF energy:-2803.870188 a.u.

Imaginary frequency: -405.0882 cm-1

(*S*, *R*)-3a-12

Cartesian coordinates

ATOM	X	Y	Z
C	-0.91217	4.82164	-1.63724
O	-1.42179	5.11054	-0.59260
O	-0.09733	3.75511	-1.79323
C	0.07075	2.90329	-0.66550
H	-0.89544	2.84516	-0.15111
C	0.49529	1.50846	-1.15689
C	1.77890	1.52085	-2.02943
H	0.68279	0.93527	-0.23812
C	-0.95267	1.31907	-3.15927
C	-0.02585	-0.62118	-2.09690
C	1.48868	0.85186	-3.37672
H	2.11907	2.55002	-2.20463
H	2.59602	1.00327	-1.50491
H	-1.62797	0.59068	-3.63196
H	-1.53265	2.23143	-2.98696
C	0.29954	1.57441	-4.02217
C	1.09424	-0.61673	-3.15860
H	-0.84801	-1.28996	-2.37986
H	0.36171	-0.98411	-1.13861
H	2.37249	0.92695	-4.02725
H	0.13933	1.20734	-5.04704
H	0.50693	2.65275	-4.08266
H	0.68228	-0.99046	-4.11300
N	-0.58115	0.73524	-1.85094
C	2.22226	-1.56115	-2.74325
H	1.76320	-2.52154	-2.45938

H	2.68913	-1.18662	-1.81713
C	3.29624	-1.79848	-3.79583
H	2.86213	-2.17457	-4.73621
H	3.85678	-0.87985	-4.03322
H	4.01878	-2.54876	-3.43983
C	1.09079	3.48610	0.29633
C	1.06567	3.17068	1.69022
C	2.08183	4.32712	-0.15518
C	0.08744	2.32945	2.28851
C	2.09041	3.73448	2.50434
C	3.05133	4.82574	0.74428
H	2.12248	4.61857	-1.20562
C	0.11663	2.07447	3.64481
H	-0.68299	1.87706	1.67259
C	2.10817	3.42600	3.89516
H	3.84366	5.48468	0.37092
C	1.15515	2.62127	4.45152
H	2.90756	3.86460	4.49435
H	1.15012	2.38951	5.51794
N	3.06875	4.54083	2.02122
O	-0.79256	1.33902	4.30433
Os	-2.59180	0.38096	-0.45495
O	-1.31311	-0.07658	0.59194
O	-3.03761	-0.83603	-1.61736
O	-3.96236	0.10359	0.57762
O	-2.88913	1.97425	-0.98507
C	-1.86496	-2.46612	4.14141
C	-2.42837	-2.78226	2.92444
C	-1.59828	-2.92918	1.79448
C	-0.20414	-2.74414	1.89551
C	0.34908	-2.41870	3.15590
C	-0.47153	-2.28068	4.25520
H	-2.49800	-2.35342	5.02400
H	-3.50335	-2.92676	2.81595
C	-2.09250	-3.25853	0.48849
C	0.56038	-2.93356	0.68739
H	1.42750	-2.28984	3.25384
H	-0.03928	-2.02396	5.22405
C	2.01693	-2.64597	0.58818
C	2.51988	-1.39756	0.99086
C	2.84969	-3.56944	-0.02978
C	3.86038	-1.08861	0.75281
H	1.85105	-0.66602	1.44628
C	4.19100	-3.26136	-0.28258

H	2.46176	-4.52855	-0.37191
C	4.70292	-2.01737	0.10398
O	4.91193	-4.18372	-0.95820
O	4.44426	0.08266	1.06716
C	3.64814	1.11858	1.57954
H	2.85884	1.40414	0.86592
H	3.17496	0.84814	2.53904
H	4.30569	1.98060	1.74054
C	6.25182	-4.44554	-0.59713
H	6.39446	-4.37038	0.49251
H	6.46299	-5.47495	-0.91718
H	6.95579	-3.75898	-1.08803
N	0.00753	-3.33715	-0.42730
N	-1.32163	-3.49702	-0.52794
O	-3.42258	-3.31976	0.35370
C	-4.00305	-3.29817	-0.95393
H	-3.19483	-3.36228	-1.69376
C	-4.73898	-1.99350	-1.13065
C	-5.30808	-1.31980	-0.07364
H	-5.07724	-1.78147	-2.14685
H	-6.06180	-0.55343	-0.25260
H	-5.25291	-1.73866	0.93023
O	5.99383	-1.68127	-0.16368
C	6.15962	-0.83681	-1.28276
H	5.65372	0.13036	-1.13110
H	7.23710	-0.66069	-1.39679
H	5.77010	-1.31082	-2.20002
C	-4.92073	-4.50394	-1.08705
H	-5.41599	-4.50111	-2.06878
H	-4.33639	-5.43022	-0.99523
H	-5.69484	-4.49183	-0.30559
C	-1.08234	5.55093	-2.94005
H	-0.10257	5.77875	-3.38183
H	-1.62422	4.90633	-3.64787
H	-1.64991	6.47257	-2.77366
C	-1.93366	0.89182	3.60918
H	-2.47558	1.73519	3.14710
H	-1.67292	0.16050	2.82854
H	-2.58262	0.41227	4.35063

PBE0+D3(BJ)/Def2-SVP Gibbs free energy:-2802.214803 a.u.

PBE0+D3(BJ)/Def2-SVP enthalpy:-2802.069074 a.u.

PBE0+D3(BJ)/Def2-SVP SCF energy:-2803.020322 a.u.

PBE0+D3(BJ)/Def2-TZVPP Gibbs free energy:-2803.066068 a.u.

PBE0+D3(BJ)/Def2-TZVPP enthalpy:-2802.920339 a.u.

PBE0+D3(BJ)/Def2-TZVPP SCF energy:-2803.871587 a.u.

Imaginary frequency: -405.5882 cm-1

(*S*, *R*)-3a-1

Cartesian coordinates

ATOM	X	Y	Z
C	-1.00075	-4.85371	-1.00060
O	-0.64343	-5.10272	0.11562
O	-1.33460	-3.61254	-1.41214
C	-1.17286	-2.55628	-0.46914
H	-0.26391	-2.76937	0.10352
C	-1.03122	-1.22864	-1.23160
C	-2.19740	-0.97231	-2.22027
H	-1.03899	-0.45571	-0.45324
C	0.45509	-1.95704	-3.07196
C	0.28615	0.35710	-2.45028
C	-1.62912	-0.69991	-3.61390
H	-2.86100	-1.84432	-2.27303
H	-2.80931	-0.13100	-1.86582
H	1.37998	-1.64246	-3.57800
H	0.62779	-2.96654	-2.68548
C	-0.75972	-1.89457	-4.01955
C	-0.75095	0.55722	-3.58172
H	1.30558	0.58900	-2.78348
H	0.07550	1.02556	-1.60615
H	-2.45162	-0.56703	-4.33352
H	-0.42745	-1.78969	-5.06341
H	-1.34650	-2.82212	-3.94821
H	-0.21746	0.61028	-4.54766
N	0.28159	-1.03591	-1.92712
C	-1.54519	1.85425	-3.42754
H	-2.04045	1.86713	-2.44144
H	-2.35227	1.86335	-4.18094
C	-0.69267	3.10921	-3.57154
H	-1.32156	4.00961	-3.50693
H	0.06370	3.17107	-2.77376
H	-0.17840	3.13076	-4.54610
C	-2.35099	-2.50334	0.48738
C	-2.20412	-2.01707	1.82210
C	-3.60377	-2.90334	0.08449
C	-0.95568	-1.64166	2.38746
C	-3.37875	-1.93468	2.62102
C	-4.70194	-2.77675	0.96375
H	-3.75960	-3.31992	-0.91141

C	-0.87563	-1.20381	3.69224
H	-0.05709	-1.69983	1.78528
C	-3.26839	-1.43708	3.95097
H	-5.69925	-3.08082	0.62525
C	-2.05803	-1.07867	4.47437
H	-4.18523	-1.37053	4.53911
H	-1.96007	-0.71628	5.49921
N	-4.60722	-2.30328	2.17968
O	0.27090	-0.88973	4.32186
Os	2.21314	-1.21001	-0.38179
O	1.11910	-0.21205	0.47799
O	3.15740	-0.36645	-1.57858
O	3.51144	-1.31477	0.77378
O	1.96024	-2.85975	-0.73318
C	2.62599	2.05030	4.17003
C	3.28010	2.07062	2.95662
C	2.54260	2.29758	1.77708
C	1.14549	2.48842	1.82947
C	0.49672	2.45180	3.08414
C	1.22981	2.23782	4.23209
H	3.18823	1.87959	5.09046
H	4.35815	1.92201	2.88868
C	3.12979	2.33963	0.46809
C	0.48300	2.74042	0.57552
H	-0.58266	2.59929	3.13639
H	0.72357	2.20267	5.19809
C	-0.99474	2.80209	0.41915
C	-1.79828	1.74990	0.86173
C	-1.55226	3.85681	-0.31167
C	-3.15766	1.73748	0.53682
H	-1.38869	0.91026	1.42751
C	-2.90769	3.84870	-0.63793
H	-0.89350	4.64768	-0.66653
C	-3.71742	2.77307	-0.22145
O	-3.52172	4.79808	-1.36879
O	-3.92767	0.68726	0.92442
C	-4.91472	0.98198	1.89890
H	-5.42543	0.03785	2.12308
H	-4.44129	1.35954	2.82139
H	-5.63502	1.72513	1.52368
C	-2.82058	5.97399	-1.67353
H	-3.52639	6.63282	-2.19366
H	-2.46190	6.47936	-0.76025
H	-1.95860	5.78319	-2.33526

N	1.14803	2.88877	-0.54002
N	2.47155	2.68087	-0.59672
O	4.42617	2.01652	0.37483
C	4.96229	1.65460	-0.90307
H	4.25414	1.97181	-1.67971
C	5.13124	0.15649	-0.96420
C	5.32675	-0.60324	0.16762
H	5.42260	-0.24110	-1.93792
H	5.72910	-1.61287	0.08743
H	5.39417	-0.11634	1.13954
O	-5.03109	2.75791	-0.56544
C	-5.34731	1.86348	-1.61187
H	-6.41801	1.97922	-1.82314
H	-4.77332	2.10555	-2.52263
H	-5.14514	0.81940	-1.31903
C	6.28506	2.38563	-1.07485
H	6.98087	2.12597	-0.26331
H	6.75033	2.11376	-2.03321
H	6.11719	3.47166	-1.06479
C	-1.12796	-5.83723	-2.13018
H	-0.38503	-5.59798	-2.90533
H	-0.95534	-6.85156	-1.75522
H	-2.12310	-5.76323	-2.58987
C	1.48689	-1.09187	3.64127
H	1.57731	-0.42458	2.76928
H	2.28739	-0.86227	4.35339
H	1.59070	-2.13852	3.30679

PBE0+D3(BJ)/Def2-SVP Gibbs free energy:-2802.217605 a.u.

PBE0+D3(BJ)/Def2-SVP enthalpy:-2802.074007 a.u.

PBE0+D3(BJ)/Def2-SVP SCF energy:-2803.025286 a.u.

PBE0+D3(BJ)/Def2-TZVPP Gibbs free energy:-2803.066319 a.u.

PBE0+D3(BJ)/Def2-TZVPP enthalpy:-2802.922721 a.u.

PBE0+D3(BJ)/Def2-TZVPP SCF energy:-2803.874000 a.u.

Imaginary frequency: -394.7232 cm-1

(*S*, *R*)-3a-4

Cartesian coordinates

ATOM	X	Y	Z
C	1.06701	4.76615	-1.05684
O	0.67965	5.03696	0.04416
O	1.39526	3.51414	-1.43927
C	1.18837	2.47517	-0.48546
H	0.26292	2.70829	0.05161
C	1.05484	1.13806	-1.23281

C	2.24273	0.85954	-2.18938
H	1.03580	0.37558	-0.44394
C	-0.37648	1.85264	-3.12121
C	-0.24667	-0.45378	-2.46067
C	1.70701	0.56674	-3.59251
H	2.91111	1.72787	-2.24185
H	2.84196	0.02190	-1.80581
H	-1.29303	1.54146	-3.64421
H	-0.54639	2.86967	-2.75354
C	0.85901	1.76212	-4.03929
C	0.81750	-0.68292	-3.56182
H	-1.25937	-0.67981	-2.81750
H	-0.06748	-1.11166	-1.60089
H	2.54650	0.41545	-4.28875
H	0.55014	1.64162	-5.08860
H	1.45310	2.68527	-3.97071
H	0.30850	-0.75047	-4.53988
N	-0.24092	0.94708	-1.95921
C	1.59986	-1.98098	-3.36097
H	2.08768	-1.95808	-2.37195
H	2.41307	-2.01878	-4.10817
C	0.74452	-3.23857	-3.46306
H	-0.04443	-3.24506	-2.69521
H	0.26955	-3.31597	-4.45449
H	1.35643	-4.14034	-3.30875
C	2.33169	2.42701	0.51365
C	2.12837	1.99357	1.85931
C	3.60637	2.78734	0.14216
C	0.85518	1.65372	2.39094
C	3.27140	1.92558	2.70445
C	4.66983	2.67457	1.06504
H	3.80573	3.16560	-0.86125
C	0.72079	1.26370	3.70648
H	-0.01904	1.69675	1.75300
C	3.10414	1.48408	4.04826
H	5.68452	2.94676	0.75167
C	1.87081	1.15896	4.53837
H	3.99705	1.43093	4.67345
H	1.72962	0.83766	5.57182
N	4.52191	2.25392	2.29501
O	-0.45115	0.97876	4.30145
Os	-2.21281	1.17175	-0.46720
O	-1.14932	0.18595	0.44320
O	-3.13335	0.30853	-1.66820

O	-3.53993	1.31558	0.65057
O	-1.93479	2.81056	-0.84987
C	-2.79134	-1.97962	4.16472
C	-3.40907	-2.02145	2.93307
C	-2.63839	-2.27629	1.78063
C	-1.24450	-2.47481	1.87807
C	-0.63413	-2.42002	3.15131
C	-1.39921	-2.17658	4.27222
H	-3.37932	-1.78597	5.06427
H	-4.48363	-1.86799	2.82987
C	-3.18755	-2.33995	0.45626
C	-0.54574	-2.74630	0.64801
H	0.44141	-2.57766	3.24023
H	-0.92194	-2.12704	5.25227
C	0.93544	-2.80298	0.52892
C	1.73278	-1.75368	1.00386
C	1.51732	-3.82491	-0.21533
C	3.09095	-1.73343	0.70189
H	1.31652	-0.92622	1.58241
C	2.87496	-3.80524	-0.54252
H	0.90692	-4.63528	-0.61258
C	3.67507	-2.73833	-0.09413
O	3.32320	-4.84368	-1.28115
O	3.86292	-0.71095	1.16094
C	4.74738	-1.05157	2.21394
H	5.45830	-1.83295	1.90045
H	5.28771	-0.13418	2.47772
H	4.18121	-1.40181	3.09377
C	4.28893	-4.64845	-2.28912
H	4.21409	-5.51543	-2.95868
H	4.07622	-3.73625	-2.87270
H	5.30678	-4.58711	-1.88025
N	-1.17844	-2.90621	-0.48474
N	-2.49915	-2.70001	-0.58279
O	-4.47978	-2.01520	0.31859
C	-4.97644	-1.68220	-0.98296
H	-4.25073	-2.02822	-1.73055
C	-5.12703	-0.18474	-1.09079
C	-5.34522	0.60922	0.01318
H	-5.38777	0.18768	-2.08294
H	-5.73349	1.62040	-0.10735
H	-5.44661	0.15176	0.99632
O	5.00418	-2.69920	-0.39121
C	5.42329	-1.61995	-1.20510

H	5.23175	-0.65036	-0.72121
H	6.50154	-1.74404	-1.36714
H	4.90801	-1.64406	-2.18177
C	-6.30247	-2.40332	-1.17087
H	-6.73798	-2.15375	-2.14907
H	-6.14753	-3.49036	-1.12569
H	-7.01727	-2.11327	-0.38658
C	1.24206	5.73038	-2.19657
H	0.51641	5.49197	-2.98819
H	1.07636	6.75293	-1.84131
H	2.24791	5.63300	-2.62762
C	-1.63790	1.14944	3.56298
H	-1.69142	0.44503	2.71743
H	-2.46711	0.94833	4.25036
H	-1.72852	2.18061	3.18020

PBE0+D3(BJ)/Def2-SVP Gibbs free energy:-2802.216336 a.u.

PBE0+D3(BJ)/Def2-SVP enthalpy:-2802.072426 a.u.

PBE0+D3(BJ)/Def2-SVP SCF energy:-2803.023344 a.u.

PBE0+D3(BJ)/Def2-TZVPP Gibbs free energy:-2803.062821 a.u.

PBE0+D3(BJ)/Def2-TZVPP enthalpy:-2802.918911 a.u.

PBE0+D3(BJ)/Def2-TZVPP SCF energy:-2803.869829 a.u.

Imaginary frequency: -394.6318 cm-1

(*S*, *R*)-3a-6

Cartesian coordinates

ATOM	X	Y	Z
C	2.06091	-3.85138	-2.27729
O	3.15680	-3.46387	-1.97472
O	0.92161	-3.28262	-1.84326
C	1.02663	-2.17335	-0.94245
H	2.00344	-1.71903	-1.11862
C	-0.06499	-1.12527	-1.26268
C	-1.34808	-1.69612	-1.92328
H	-0.32508	-0.65930	-0.30428
C	0.89117	-0.49256	-3.41796
C	-0.56670	1.01081	-2.24274
C	-1.55183	-1.01452	-3.27922
H	-1.26248	-2.78095	-2.06773
H	-2.21641	-1.51374	-1.27549
H	1.22191	0.38958	-3.98473
H	1.77841	-1.13050	-3.31266
C	-0.28305	-1.21383	-4.11721
C	-1.77232	0.49245	-3.06754
H	-0.11065	1.89642	-2.70227

H	-0.88781	1.31304	-1.23628
H	-2.41316	-1.46148	-3.79600
H	-0.43060	-0.81635	-5.13254
H	-0.07225	-2.28949	-4.21489
H	-1.75240	0.96812	-4.06440
N	0.48935	-0.01286	-2.08271
C	-3.09655	0.86752	-2.39689
H	-3.09299	1.95462	-2.21360
H	-3.13558	0.41973	-1.39301
C	-4.35105	0.47586	-3.16669
H	-5.25244	0.83001	-2.64443
H	-4.35539	0.90474	-4.18216
H	-4.44980	-0.61641	-3.26434
C	0.94630	-2.66958	0.48875
C	2.03728	-2.55601	1.40162
C	-0.22301	-3.23573	0.94682
C	3.32123	-2.05698	1.04470
C	1.79966	-2.96588	2.74750
C	-0.33330	-3.64315	2.29235
H	-1.07119	-3.36183	0.27448
C	4.31015	-1.92908	1.99404
H	3.53185	-1.82180	0.00393
C	2.84459	-2.81244	3.70575
H	-1.26689	-4.09420	2.64960
C	4.05780	-2.30097	3.34707
H	2.63563	-3.12580	4.73002
H	4.86798	-2.18160	4.06855
N	0.62811	-3.50009	3.17014
O	5.55106	-1.47637	1.75002
Os	2.45084	1.11626	-0.83488
O	1.55249	0.38313	0.40851
O	1.76354	2.60778	-1.41681
O	3.72029	1.88921	0.06532
O	3.32025	0.22623	-2.00408
C	1.00014	0.40404	3.84755
C	1.05290	1.51600	3.04017
C	-0.05323	1.83843	2.22655
C	-1.21444	1.03414	2.22653
C	-1.25637	-0.07924	3.10155
C	-0.16638	-0.38815	3.88596
H	1.86241	0.12497	4.45591
H	1.94620	2.13804	2.98778
C	-0.05653	2.95373	1.32636
C	-2.27336	1.43193	1.32727

H	-2.15789	-0.68765	3.16298
H	-0.19861	-1.26797	4.53106
C	-3.54207	0.67461	1.11613
C	-3.58100	-0.72232	1.00845
C	-4.69458	1.42674	0.87293
C	-4.78356	-1.35624	0.66353
H	-2.67125	-1.30666	1.14419
C	-5.88426	0.79785	0.50714
H	-4.61849	2.51038	0.92578
C	-5.93162	-0.59974	0.38410
O	-7.03385	1.44446	0.23221
O	-4.94692	-2.69067	0.55224
C	-3.84255	-3.53205	0.70630
H	-3.35679	-3.40644	1.68991
H	-4.21381	-4.56065	0.62328
H	-3.09547	-3.36033	-0.08685
C	-7.04295	2.84693	0.26242
H	-8.05725	3.15851	-0.01468
H	-6.81025	3.23714	1.26822
H	-6.32578	3.27389	-0.45921
N	-2.18481	2.52051	0.60163
N	-1.08479	3.28619	0.60449
O	1.07750	3.65757	1.23330
C	1.21555	4.62272	0.18295
H	0.38198	4.48711	-0.51703
C	2.51011	4.34283	-0.53839
C	3.65550	3.94653	0.11666
H	2.59037	4.77414	-1.53842
H	4.62495	4.02712	-0.37441
H	3.65747	3.85032	1.20220
O	-7.06593	-1.21720	-0.02301
C	-7.99763	-1.48459	0.99978
H	-8.85551	-1.98553	0.53296
H	-7.56759	-2.15186	1.76647
H	-8.34419	-0.55371	1.47954
C	1.15179	6.01968	0.78066
H	1.29635	6.77787	-0.00279
H	0.16679	6.18073	1.24127
H	1.93182	6.15332	1.54468
C	1.76354	-5.00749	-3.19021
H	2.70135	-5.47700	-3.50512
H	1.12739	-5.73754	-2.67096
H	1.20837	-4.65173	-4.07005
C	5.86564	-1.03441	0.45112

H	6.92363	-0.74760	0.46845
H	5.71883	-1.82973	-0.29863
H	5.26086	-0.15629	0.17205

PBE0+D3(BJ)/Def2-SVP Gibbs free energy:-2802.208335 a.u.
PBE0+D3(BJ)/Def2-SVP enthalpy:-2802.059295 a.u.
PBE0+D3(BJ)/Def2-SVP SCF energy:-2803.010499 a.u.
PBE0+D3(BJ)/Def2-TZVPP Gibbs free energy:-2803.062317 a.u.
PBE0+D3(BJ)/Def2-TZVPP enthalpy:-2802.913277 a.u.
PBE0+D3(BJ)/Def2-TZVPP SCF energy:-2803.864481 a.u.
Imaginary frequency: -428.2563 cm-1

(*S*, *R*)-3a-8

Cartesian coordinates

ATOM	X	Y	Z
C	2.06148	-3.85094	-2.27763
O	3.15733	-3.46362	-1.97465
O	0.92212	-3.28224	-1.84369
C	1.02694	-2.17316	-0.94263
H	2.00374	-1.71872	-1.11856
C	-0.06473	-1.12513	-1.26281
C	-1.34770	-1.69599	-1.92364
H	-0.32497	-0.65932	-0.30438
C	0.89160	-0.49193	-3.41789
C	-0.56659	1.01104	-2.24250
C	-1.55135	-1.01420	-3.27951
H	-1.26200	-2.78079	-2.06824
H	-2.21613	-1.51377	-1.27593
H	1.22226	0.39037	-3.98447
H	1.77893	-1.12974	-3.31262
C	-0.28245	-1.21325	-4.11738
C	-1.77201	0.49270	-3.06762
H	-0.11062	1.89688	-2.70167
H	-0.88792	1.31282	-1.23598
H	-2.41256	-1.46116	-3.79647
H	-0.42992	-0.81564	-5.13267
H	-0.07151	-2.28887	-4.21517
H	-1.75199	0.96855	-4.06438
N	0.48959	-0.01252	-2.08259
C	-3.09638	0.86753	-2.39709
H	-3.09313	1.95467	-2.21404
H	-3.13530	0.41994	-1.39312
C	-4.35075	0.47534	-3.16683
H	-5.25223	0.82956	-2.64480
H	-4.35508	0.90379	-4.18249

H	-4.44930	-0.61698	-3.26403
C	0.94643	-2.66966	0.48847
C	2.03730	-2.55638	1.40150
C	-0.22300	-3.23580	0.94628
C	3.32130	-2.05727	1.04487
C	1.79951	-2.96660	2.74724
C	-0.33348	-3.64353	2.29170
H	-1.07111	-3.36157	0.27381
C	4.31011	-1.92969	1.99436
H	3.53204	-1.82178	0.00420
C	2.84435	-2.81352	3.70565
H	-1.26716	-4.09456	2.64874
C	4.05762	-2.30200	3.34724
H	2.63526	-3.12717	4.72980
H	4.86772	-2.18289	4.06885
N	0.62786	-3.50082	3.16962
O	5.55105	-1.47689	1.75062
Os	2.45095	1.11658	-0.83466
O	1.55282	0.38310	0.40869
O	1.76332	2.60793	-1.41650
O	3.72032	1.88967	0.06556
O	3.32044	0.22673	-2.00394
C	0.99994	0.40378	3.84737
C	1.05262	1.51585	3.04014
C	-0.05351	1.83827	2.22652
C	-1.21465	1.03387	2.22636
C	-1.25647	-0.07967	3.10118
C	-0.16647	-0.38858	3.88559
H	1.86219	0.12478	4.45578
H	1.94584	2.13802	2.98790
C	-0.05690	2.95370	1.32648
C	-2.27362	1.43175	1.32719
H	-2.15793	-0.68818	3.16249
H	-0.19862	-1.26850	4.53055
C	-3.54230	0.67438	1.11600
C	-3.58113	-0.72254	1.00808
C	-4.69488	1.42646	0.87303
C	-4.78366	-1.35649	0.66314
H	-2.67132	-1.30684	1.14366
C	-5.88454	0.79754	0.50722
H	-4.61886	2.51010	0.92605
C	-5.93181	-0.60003	0.38395
O	-7.03421	1.44411	0.23252
O	-4.94691	-2.69092	0.55160

C	-3.84252	-3.53224	0.70583
H	-3.09518	-3.36033	-0.08704
H	-3.35708	-3.40678	1.68962
H	-4.21368	-4.56084	0.62246
C	-7.04342	2.84658	0.26297
H	-6.81067	3.23663	1.26882
H	-6.32634	3.27372	-0.45865
H	-8.05776	3.15813	-0.01400
N	-2.18516	2.52046	0.60173
N	-1.08518	3.28620	0.60467
O	1.07710	3.65761	1.23352
C	1.21511	4.62293	0.18332
H	0.38157	4.48736	-0.51671
C	2.50974	4.34325	-0.53798
C	3.65512	3.94708	0.11711
H	2.58998	4.77460	-1.53800
H	4.62460	4.02777	-0.37390
H	3.65700	3.85073	1.20262
O	-7.06609	-1.21752	-0.02316
C	-7.99785	-1.48484	0.99959
H	-8.85570	-1.98582	0.53276
H	-7.56786	-2.15204	1.76637
H	-8.34445	-0.55392	1.47926
C	1.15120	6.01979	0.78124
H	0.16616	6.18070	1.24180
H	1.93117	6.15337	1.54533
H	1.29577	6.77811	-0.00208
C	1.76421	-5.00648	-3.19131
H	1.21194	-4.64943	-4.07247
H	2.70191	-5.47759	-3.50410
H	1.12531	-5.73539	-2.67387
C	5.86575	-1.03449	0.45190
H	5.26102	-0.15625	0.17309
H	6.92375	-0.74774	0.46940
H	5.71896	-1.82955	-0.29815

PBE0+D3(BJ)/Def2-SVP Gibbs free energy:-2802.208339 a.u.

PBE0+D3(BJ)/Def2-SVP enthalpy:-2802.059290 a.u.

PBE0+D3(BJ)/Def2-SVP SCF energy:-2803.010499 a.u.

PBE0+D3(BJ)/Def2-TZVPP Gibbs free energy:-2803.062325 a.u.

PBE0+D3(BJ)/Def2-TZVPP enthalpy:-2802.913276 a.u.

PBE0+D3(BJ)/Def2-TZVPP SCF energy:-2803.864485 a.u.

Imaginary frequency: -428.1209 cm-1

(*S*, *R*)-4-2

Cartesian coordinates

ATOM	X	Y	Z
C	0.82538	-1.32415	4.11517
O	1.97345	-0.97226	4.11849
O	0.01876	-1.21968	3.04506
C	0.57993	-0.65940	1.85185
H	1.61996	-0.98614	1.80619
C	-0.16536	-1.20359	0.61617
C	-1.66282	-1.53480	0.85679
H	-0.08761	-0.42306	-0.14847
C	0.52437	-3.52734	0.93564
C	-0.20663	-2.74070	-1.21924
C	-1.89369	-3.02157	0.56973
H	-1.95084	-1.32026	1.89387
H	-2.29042	-0.91496	0.20422
H	0.96107	-4.37490	0.38656
H	1.20898	-3.31282	1.76709
C	-0.91124	-3.83906	1.41790
C	-1.60911	-3.31526	-0.91014
H	0.40755	-3.45409	-1.78117
H	-0.28364	-1.82345	-1.82481
H	-2.93200	-3.29222	0.81682
H	-1.12340	-4.91469	1.32220
H	-1.02768	-3.57158	2.47834
H	-1.57738	-4.41410	-1.02561
N	0.53010	-2.37774	0.01261
C	-2.66459	-2.77742	-1.87475
H	-2.66142	-1.67767	-1.82511
H	-3.66283	-3.11240	-1.54138
C	-2.42690	-3.21206	-3.31755
H	-3.20465	-2.82043	-3.99107
H	-1.46116	-2.84163	-3.69426
H	-2.42544	-4.30982	-3.41395
C	0.52557	0.85483	1.90192
C	1.70015	1.65937	1.78763
C	-0.68746	1.49656	2.01437
C	3.02187	1.13126	1.78522
C	1.51202	3.06831	1.67420
C	-0.74630	2.90631	1.96618
H	-1.60787	0.92004	2.11739
C	4.10396	1.96380	1.61170
H	3.17190	0.06849	1.95616
C	2.65455	3.89581	1.46637
H	-1.71325	3.41438	2.06737

C	3.91163	3.36283	1.42385
H	2.48358	4.96811	1.35891
H	4.79497	3.98596	1.27403
N	0.29991	3.66900	1.77413
O	5.38742	1.55375	1.59142
Os	2.91092	-1.75562	-0.72482
O	3.22334	-2.18438	0.90068
O	2.10783	-0.22377	-0.92385
O	4.45711	-1.15956	-1.25300
O	2.54339	-2.91080	-1.91963
C	-0.89515	5.59433	-0.88655
C	-0.10466	4.60533	-1.42737
C	-0.62913	3.30580	-1.58075
C	-1.94207	3.00482	-1.16714
C	-2.74878	4.04813	-0.65540
C	-2.22700	5.31657	-0.51687
H	-0.49450	6.60011	-0.74845
H	0.92290	4.79978	-1.73393
C	0.11359	2.22598	-2.16960
C	-2.36190	1.63231	-1.31165
H	-3.78281	3.84284	-0.37642
H	-2.85236	6.11703	-0.11604
C	-3.65361	1.12288	-0.77564
C	-4.00869	1.38086	0.55219
C	-4.43303	0.27440	-1.56141
C	-5.12624	0.75421	1.11086
H	-3.37732	2.02580	1.15836
C	-5.55720	-0.34705	-1.00931
H	-4.12161	0.07713	-2.58560
C	-5.91519	-0.10355	0.32753
O	-6.35414	-1.20521	-1.67411
O	-5.51547	0.90151	2.39274
C	-4.78087	1.75279	3.22762
H	-5.27522	1.73705	4.20630
H	-3.73753	1.41163	3.35124
H	-4.77150	2.79062	2.85074
C	-6.11476	-1.43570	-3.03720
H	-5.12866	-1.89845	-3.20737
H	-6.89532	-2.12588	-3.37893
H	-6.17884	-0.50308	-3.62345
N	-1.60110	0.73087	-1.87835
N	-0.36967	1.02993	-2.33165
O	1.34187	2.53521	-2.58445
C	2.28386	1.51303	-2.93695

H	1.72764	0.58798	-3.14367
C	3.18439	1.32043	-1.73265
C	4.47874	0.84470	-1.77845
H	2.89011	1.91032	-0.86295
H	5.14798	1.05725	-0.94456
H	4.94731	0.55775	-2.71977
O	-7.01293	-0.68934	0.86204
C	-6.79746	-1.98763	1.36628
H	-6.04750	-1.98086	2.17629
H	-7.75454	-2.34185	1.77028
H	-6.46743	-2.67666	0.57031
C	2.99943	1.98200	-4.19145
H	2.26880	2.14873	-4.99490
H	3.54635	2.91823	-4.00795
H	3.71259	1.21894	-4.53263
C	0.09808	-1.95089	5.27238
H	0.73762	-1.93280	6.16118
H	-0.84105	-1.41429	5.46408
H	-0.15932	-2.99063	5.02254
C	5.66399	0.19641	1.85493
H	6.75584	0.09995	1.86925
H	5.25578	-0.11455	2.83065
H	5.25436	-0.45937	1.06989

PBE0+D3(BJ)/Def2-SVP Gibbs free energy:-2802.210741 a.u.

PBE0+D3(BJ)/Def2-SVP enthalpy:-2802.063645 a.u.

PBE0+D3(BJ)/Def2-SVP SCF energy:-2803.015124 a.u.

PBE0+D3(BJ)/Def2-TZVPP Gibbs free energy:-2803.065267 a.u.

PBE0+D3(BJ)/Def2-TZVPP enthalpy:-2802.918171 a.u.

PBE0+D3(BJ)/Def2-TZVPP SCF energy:-2803.869650 a.u.

Imaginary frequency: -424.0300 cm-1

(*S*, *S*)-4-6

Cartesian coordinates

ATOM	X	Y	Z
C	0.97041	-1.31876	4.11751
O	2.11883	-0.96881	4.08930
O	0.13271	-1.21029	3.07242
C	0.65578	-0.65184	1.86080
H	1.70080	-0.95791	1.79621
C	-0.10405	-1.22354	0.64713
C	-1.58571	-1.58984	0.93022
H	-0.06479	-0.44692	-0.12430
C	0.64933	-3.52833	0.96225
C	-0.15544	-2.77032	-1.17914

C	-1.78705	-3.08352	0.65670
H	-1.84987	-1.37698	1.97382
H	-2.24666	-0.98923	0.29256
H	1.09261	-4.36807	0.40647
H	1.34953	-3.29288	1.77475
C	-0.76447	-3.87239	1.48272
C	-1.53736	-3.37357	-0.83035
H	0.46063	-3.47332	-1.75209
H	-0.26769	-1.85902	-1.78779
H	-2.81137	-3.37788	0.93269
H	-0.95318	-4.95318	1.39677
H	-0.86038	-3.60326	2.54494
H	-1.48723	-4.47149	-0.94824
N	0.60432	-2.38401	0.03328
C	-2.64107	-2.85316	-1.74880
H	-2.66299	-1.75534	-1.68724
H	-3.61518	-3.21349	-1.37562
C	-2.47257	-3.25879	-3.20813
H	-2.44378	-4.35456	-3.32385
H	-3.30510	-2.87760	-3.81839
H	-1.54181	-2.85259	-3.63368
C	0.57102	0.86143	1.89633
C	1.73197	1.68494	1.77958
C	-0.65386	1.48333	1.99615
C	3.06160	1.17747	1.77918
C	1.52148	3.08978	1.65636
C	-0.73577	2.89153	1.93647
H	-1.56594	0.89310	2.09835
C	4.13067	2.02513	1.60022
H	3.22845	0.11802	1.95450
C	2.65151	3.93400	1.44674
H	-1.71209	3.38345	2.02660
C	3.91692	3.42041	1.40755
H	2.46414	5.00294	1.33326
H	4.79040	4.05658	1.25464
N	0.29889	3.67058	1.74630
O	5.41950	1.63226	1.57818
Os	2.96503	-1.72361	-0.73878
O	3.30441	-2.15576	0.88058
O	2.15254	-0.19460	-0.91785
O	4.50279	-1.11837	-1.28216
O	2.58785	-2.87338	-1.93567
C	-0.97457	5.54772	-0.92151
C	-0.15263	4.57747	-1.44912

C	-0.64038	3.26349	-1.60035
C	-1.94893	2.92931	-1.19888
C	-2.78808	3.95314	-0.70000
C	-2.30194	5.23584	-0.56290
H	-0.60245	6.56461	-0.78499
H	0.87247	4.79775	-1.74626
C	0.13635	2.20065	-2.17576
C	-2.32989	1.54554	-1.34060
H	-3.81923	3.72237	-0.43042
H	-2.95235	6.02129	-0.17224
C	-3.62348	1.01003	-0.83716
C	-4.02268	1.25396	0.48673
C	-4.36490	0.15201	-1.63708
C	-5.15574	0.62569	0.99676
H	-3.42060	1.90624	1.11566
C	-5.50240	-0.48980	-1.13249
H	-4.05626	-0.06388	-2.65955
C	-5.90611	-0.25553	0.18790
O	-6.16273	-1.30758	-1.98012
O	-5.61843	0.79419	2.25296
C	-4.90503	1.62502	3.12715
H	-4.85608	2.66494	2.75882
H	-5.44234	1.61180	4.08288
H	-3.87655	1.25848	3.29162
C	-6.66818	-2.54483	-1.52400
H	-6.00889	-2.98958	-0.76174
H	-7.67728	-2.44939	-1.09849
H	-6.70189	-3.20695	-2.39974
N	-1.53719	0.65954	-1.88690
N	-0.31055	0.98963	-2.32966
O	1.35754	2.54077	-2.58603
C	2.32137	1.54122	-2.94415
H	1.78512	0.60683	-3.16198
C	3.22245	1.35594	-1.74008
C	4.51834	0.88551	-1.78886
H	2.92592	1.94482	-0.87064
H	5.18786	1.09954	-0.95556
H	4.98731	0.60562	-2.73206
O	-7.01213	-0.86037	0.69954
C	-8.17480	-0.06058	0.69423
H	-8.98609	-0.66782	1.11624
H	-8.04234	0.84041	1.31509
H	-8.44544	0.24151	-0.33264
C	3.03110	2.03801	-4.19153

H	3.55821	2.98320	-3.99623
H	3.76072	1.29342	-4.53853
H	2.29959	2.19835	-4.99549
C	0.27686	-1.94940	5.29326
H	-0.66147	-1.42034	5.50805
H	0.02171	-2.99137	5.05032
H	0.93854	-1.92624	6.16560
C	5.71255	0.27762	1.83854
H	5.31323	-0.03921	2.81605
H	5.30518	-0.38207	1.05533
H	6.80546	0.19314	1.84638

PBE0+D3(BJ)/Def2-SVP Gibbs free energy:-2802.210179 a.u.

PBE0+D3(BJ)/Def2-SVP enthalpy:-2802.060973 a.u.

PBE0+D3(BJ)/Def2-SVP SCF energy:-2803.012017 a.u.

PBE0+D3(BJ)/Def2-TZVPP Gibbs free energy:-2803.063533 a.u.

PBE0+D3(BJ)/Def2-TZVPP enthalpy:-2802.914327 a.u.

PBE0+D3(BJ)/Def2-TZVPP SCF energy:-2803.865371 a.u.

Imaginary frequency: -425.8799 cm-1

(*S*, *S*)-4a-12

Cartesian coordinates

ATOM	X	Y	Z
C	1.00221	4.83537	1.04984
O	0.43973	4.63513	2.08896
O	1.42609	3.84731	0.23517
C	1.12601	2.51092	0.63334
H	0.11677	2.51540	1.06070
C	1.17426	1.59955	-0.60362
C	2.49216	1.73932	-1.41034
H	1.09658	0.58050	-0.20382
C	-0.03689	3.02890	-2.20957
C	0.13710	0.65836	-2.54312
C	2.16192	2.04080	-2.87359
H	3.10664	2.55701	-1.01459
H	3.08897	0.82147	-1.31500
H	-0.87018	2.97076	-2.92500
H	-0.29304	3.80598	-1.48098
C	1.30407	3.31196	-2.91988
C	1.35170	0.88241	-3.47189
H	-0.79597	0.62364	-3.11548
H	0.21305	-0.30303	-2.01504
H	3.09105	2.20047	-3.44060
H	1.13142	3.61703	-3.96309
H	1.83550	4.13377	-2.41759

H	0.98982	1.20953	-4.46305
N	0.00116	1.72530	-1.51624
C	2.12539	-0.42097	-3.66275
H	1.41146	-1.20681	-3.96012
H	2.51206	-0.74917	-2.68558
C	3.26207	-0.36822	-4.67399
H	4.03880	0.35865	-4.38675
H	3.74105	-1.35511	-4.75635
H	2.89875	-0.08205	-5.67397
C	2.11771	2.02716	1.67730
C	1.74635	1.07878	2.67838
C	3.42412	2.45778	1.65715
C	0.41243	0.63186	2.87701
C	2.77650	0.58965	3.53085
C	4.36179	1.91320	2.56222
H	3.74799	3.21538	0.94274
C	0.11207	-0.26310	3.88163
H	-0.37668	1.00876	2.23698
C	2.44202	-0.37857	4.52042
H	5.40415	2.25050	2.52310
C	1.15236	-0.79711	4.69290
H	3.25143	-0.75333	5.14924
H	0.88107	-1.51867	5.46557
N	4.06605	1.00327	3.45438
O	-1.12941	-0.67800	4.18669
Os	-2.20829	1.23616	-0.43997
O	-1.18055	0.05065	0.23829
O	-2.70296	1.07448	-2.11245
O	-3.74227	0.70496	0.18520
O	-2.10456	2.86471	0.04751
C	-2.64599	-3.57361	3.27664
C	-3.34157	-3.09829	2.18692
C	-2.64404	-2.79286	1.00017
C	-1.24967	-2.96949	0.91517
C	-0.55966	-3.46501	2.04627
C	-1.24994	-3.75446	3.20335
H	-3.17266	-3.80597	4.20430
H	-4.42032	-2.94386	2.22065
C	-3.28302	-2.28786	-0.18115
C	-0.63725	-2.65805	-0.35499
H	0.51785	-3.62238	1.99686
H	-0.70916	-4.12735	4.07516
C	0.84005	-2.64582	-0.52940
C	1.63989	-1.92581	0.36602

C	1.43258	-3.28228	-1.62087
C	3.01719	-1.85112	0.17388
H	1.20746	-1.39293	1.21547
C	2.80966	-3.20636	-1.82420
H	0.82248	-3.81756	-2.34890
C	3.61619	-2.49253	-0.92265
O	3.33748	-3.75492	-2.94930
O	3.77257	-1.11061	1.02849
C	4.59846	-1.85217	1.90767
H	5.36853	-2.41270	1.35353
H	5.06584	-1.12668	2.58475
H	3.99216	-2.55516	2.50452
C	4.23910	-4.82725	-2.76985
H	5.13394	-4.51512	-2.21083
H	3.75160	-5.66512	-2.24171
H	4.53400	-5.16378	-3.77189
N	-1.33856	-2.32733	-1.40596
N	-2.66763	-2.12677	-1.31614
O	-4.57862	-1.99597	-0.06438
C	-5.26578	-1.43887	-1.18652
H	-4.85881	-1.90941	-2.09269
C	-5.10979	0.06623	-1.32267
C	-4.56189	0.60075	-2.47562
H	-5.78484	0.67589	-0.72067
H	-4.80947	1.62086	-2.77180
H	-4.22962	-0.07004	-3.26932
O	4.96032	-2.41955	-1.12767
C	5.43680	-1.15983	-1.56132
H	5.23113	-0.37768	-0.81313
H	6.52147	-1.25779	-1.69514
H	4.98012	-0.87945	-2.52401
C	-6.72823	-1.80885	-1.00462
H	-7.32544	-1.42531	-1.84404
H	-6.84216	-2.90099	-0.96140
H	-7.12022	-1.37875	-0.07073
C	1.32285	6.18103	0.46127
H	1.04141	6.96786	1.16903
H	2.39400	6.24577	0.22501
H	0.76735	6.31068	-0.47912
C	-2.21017	-0.15429	3.45266
H	-3.12101	-0.58159	3.88637
H	-2.25350	0.94601	3.52956
H	-2.14912	-0.44083	2.39134

PBE0+D3(BJ)/Def2-SVP Gibbs free energy:-2802.214239 a.u.

PBE0+D3(BJ)/Def2-SVP enthalpy:-2802.066922 a.u.

PBE0+D3(BJ)/Def2-SVP SCF energy:-2803.017659 a.u.

PBE0+D3(BJ)/Def2-TZVPP Gibbs free energy:-2803.062200 a.u.

PBE0+D3(BJ)/Def2-TZVPP enthalpy:-2802.914883 a.u.

PBE0+D3(BJ)/Def2-TZVPP SCF energy:-2803.865620 a.u.

Imaginary frequency: -457.0442 cm-1

(*S*, *S*)-4a-2

Cartesian coordinates

ATOM	X	Y	Z
C	0.34495	5.13971	-1.12133
O	-0.28086	5.34164	-0.11979
O	0.97711	3.97449	-1.37648
C	0.82122	2.93306	-0.41709
H	-0.21428	2.96961	-0.05877
C	1.09711	1.57612	-1.09206
C	2.45029	1.51992	-1.85074
H	1.10500	0.85204	-0.26603
C	-0.17144	1.95993	-3.17175
C	0.39688	-0.26497	-2.45098
C	2.19196	1.18780	-3.32460
H	2.97377	2.48216	-1.78553
H	3.11231	0.76908	-1.39419
H	-0.88844	1.44000	-3.82385
H	-0.64595	2.89639	-2.85708
C	1.17826	2.19460	-3.88187
C	1.57702	-0.21263	-3.44475
H	-0.48391	-0.74098	-2.89572
H	0.65756	-0.85259	-1.55882
H	3.13511	1.24037	-3.88927
H	1.06688	2.06177	-4.96857
H	1.53158	3.22008	-3.70430
H	1.17787	-0.30461	-4.47113
N	0.00219	1.09458	-1.98449
C	2.55834	-1.36416	-3.24010
H	2.94568	-1.34467	-2.20856
H	3.43014	-1.21276	-3.89858
C	1.93925	-2.73019	-3.51900
H	1.58420	-2.79875	-4.56022
H	2.67863	-3.52834	-3.35821
H	1.08387	-2.92762	-2.85383
C	1.76479	3.14862	0.75263
C	1.45457	2.66431	2.06059
C	2.97320	3.78260	0.57334

C	0.21164	2.06560	2.39863
C	2.45182	2.81468	3.06770
C	3.88515	3.88392	1.64845
H	3.23837	4.20404	-0.39709
C	-0.04017	1.64975	3.68935
H	-0.55002	1.95047	1.63594
C	2.18062	2.32199	4.37686
H	4.85225	4.37441	1.48860
C	0.97487	1.75614	4.68215
H	2.96581	2.43439	5.12612
H	0.74664	1.39868	5.68778
N	3.65299	3.40492	2.84417
O	-1.21355	1.15485	4.11458
Os	-2.17493	0.74650	-0.83467
O	-1.05222	0.05955	0.25895
O	-2.54570	-0.13787	-2.29649
O	-3.66970	0.33069	-0.04963
O	-2.30131	2.43371	-1.03863
C	-2.07628	-2.07840	4.44073
C	-2.84110	-2.17503	3.29925
C	-2.20647	-2.36223	2.05427
C	-0.80501	-2.46609	1.96599
C	-0.04252	-2.37348	3.15378
C	-0.67195	-2.17375	4.36377
H	-2.55425	-1.91910	5.40918
H	-3.92805	-2.09587	3.33191
C	-2.91890	-2.44832	0.81141
C	-0.25659	-2.70043	0.65089
H	1.04253	-2.46739	3.10652
H	-0.07648	-2.09224	5.27513
C	1.20971	-2.77747	0.40984
C	2.05135	-1.73462	0.81422
C	1.71857	-3.87641	-0.27981
C	3.41899	-1.80271	0.52286
H	1.62860	-0.87338	1.33281
C	3.08233	-3.94102	-0.57802
H	1.02876	-4.65578	-0.59862
C	3.93508	-2.89388	-0.19712
O	3.66553	-4.95429	-1.24932
O	4.32042	-0.86303	0.86650
C	3.89155	0.23387	1.63057
H	3.41786	-0.07902	2.57697
H	4.77933	0.83414	1.85918
H	3.17895	0.85937	1.07069

C	2.87484	-6.04306	-1.64521
H	3.54498	-6.74728	-2.15285
H	2.41187	-6.54782	-0.77960
H	2.08007	-5.73677	-2.34682
N	-1.01182	-2.86701	-0.40215
N	-2.34885	-2.72172	-0.32549
O	-4.23284	-2.23650	0.87134
C	-4.98747	-2.26508	-0.34222
H	-4.59708	-3.09147	-0.95369
C	-4.90454	-0.98983	-1.16813
C	-4.31989	-1.00075	-2.42125
H	-5.65339	-0.22850	-0.94349
H	-4.62945	-0.27327	-3.17213
H	-3.85128	-1.91696	-2.78370
O	5.24315	-2.92330	-0.54935
C	6.10884	-3.45798	0.42501
H	6.08453	-2.86181	1.35286
H	5.84966	-4.50535	0.65731
H	7.12400	-3.42693	0.00860
C	-6.42268	-2.54089	0.07050
H	-6.48734	-3.49267	0.61574
H	-6.79473	-1.73701	0.72326
H	-7.06928	-2.59860	-0.81663
C	0.53396	6.10338	-2.25916
H	0.07497	7.06494	-2.00630
H	1.60430	6.23570	-2.46982
H	0.06598	5.69436	-3.16659
C	-2.29025	1.11145	3.20779
H	-3.16072	0.75612	3.76984
H	-2.50842	2.11193	2.79580
H	-2.08770	0.41391	2.38111

PBE0+D3(BJ)/Def2-SVP Gibbs free energy:1.759059 a.u.

PBE0+D3(BJ)/Def2-SVP enthalpy:1.759059 a.u.

PBE0+D3(BJ)/Def2-SVP SCF energy:0.951782 a.u.

PBE0+D3(BJ)/Def2-TZVPP Gibbs free energy:1.759059 a.u.

PBE0+D3(BJ)/Def2-TZVPP enthalpy:1.759059 a.u.

PBE0+D3(BJ)/Def2-TZVPP SCF energy:0.951782 a.u.

Imaginary frequency: -454.6429 cm-1
